# Supplementary material for: Human Tumor–Derived Matrix Improves the Predictability of Head and Neck Cancer Drug Testing
Source: Cancers (Basel). 2019 Dec 30;12(1):92. doi: 10.3390/cancers12010092 (PMC7017272; doi:10.3390/cancers12010092)
Supplement: Supplementary file 1 [file cancers-12-00092-s001.zip › cancers-664648-supplement-final/Supplementary Figure 2.pdf]

Supplementary Figure 2: Bar plots of DSS values of drugs for each cell line cultured in different conditions.

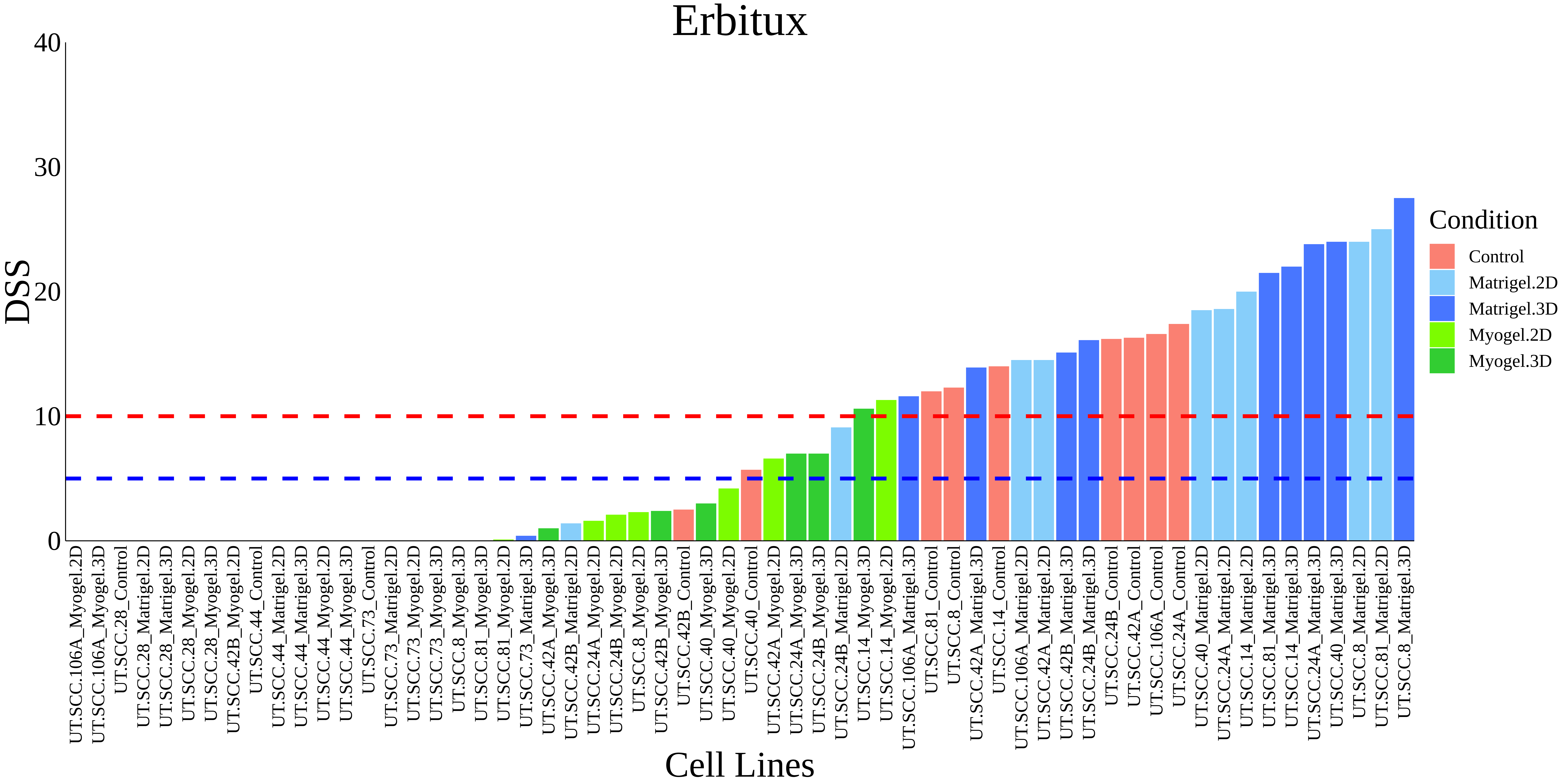

Erbitux
DSS
40
30
20
10
0
Cell Lines
Condition
Control
Matrigel.2D
Matrigel.3D
Myogel.2D
Myogel.3D
UT.SCC.106A_Myogel.2D
UT.SCC.106A_Myogel.3D
UT.SCC.28_Control
UT.SCC.28_Matrigel.2D
UT.SCC.28_Matrigel.3D
UT.SCC.28_Myogel.2D
UT.SCC.28_Myogel.3D
UT.SCC.42B_Myogel.2D
UT.SCC.44_Control
UT.SCC.44_Matrigel.2D
UT.SCC.44_Matrigel.3D
UT.SCC.44_Myogel.2D
UT.SCC.44_Myogel.3D
UT.SCC.73_Control
UT.SCC.73_Matrigel.2D
UT.SCC.73_Myogel.2D
UT.SCC.73_Myogel.3D
UT.SCC.8_Myogel.3D
UT.SCC.81_Myogel.3D
UT.SCC.81_Myogel.2D
UT.SCC.73_Matrigel.3D
UT.SCC.42A_Myogel.3D
UT.SCC.42B_Matrigel.2D
UT.SCC.24A_Myogel.2D
UT.SCC.24B_Myogel.2D
UT.SCC.8_Myogel.2D
UT.SCC.42B_Myogel.3D
UT.SCC.42B_Control
UT.SCC.40_Myogel.3D
UT.SCC.40_Myogel.2D
UT.SCC.40_Control
UT.SCC.42A_Myogel.2D
UT.SCC.24A_Myogel.3D
UT.SCC.24B_Myogel.3D
UT.SCC.24B_Matrigel.2D
UT.SCC.14_Myogel.3D
UT.SCC.14_Myogel.2D
UT.SCC.106A_Matrigel.3D
UT.SCC.81_Control
UT.SCC.8_Control
UT.SCC.42A_Matrigel.3D
UT.SCC.14_Control
UT.SCC.106A_Matrigel.2D
UT.SCC.42A_Matrigel.2D
UT.SCC.42B_Matrigel.3D
UT.SCC.24B_Matrigel.3D
UT.SCC.24B_Control
UT.SCC.42A_Control
UT.SCC.106A_Control
UT.SCC.24A_Control
UT.SCC.40_Matrigel.2D
UT.SCC.24A_Matrigel.2D
UT.SCC.14_Matrigel.2D
UT.SCC.81_Matrigel.3D
UT.SCC.14_Matrigel.3D
UT.SCC.24A_Matrigel.3D
UT.SCC.40_Matrigel.3D
UT.SCC.8_Matrigel.2D
UT.SCC.81_Matrigel.2D
UT.SCC.8_Matrigel.3D

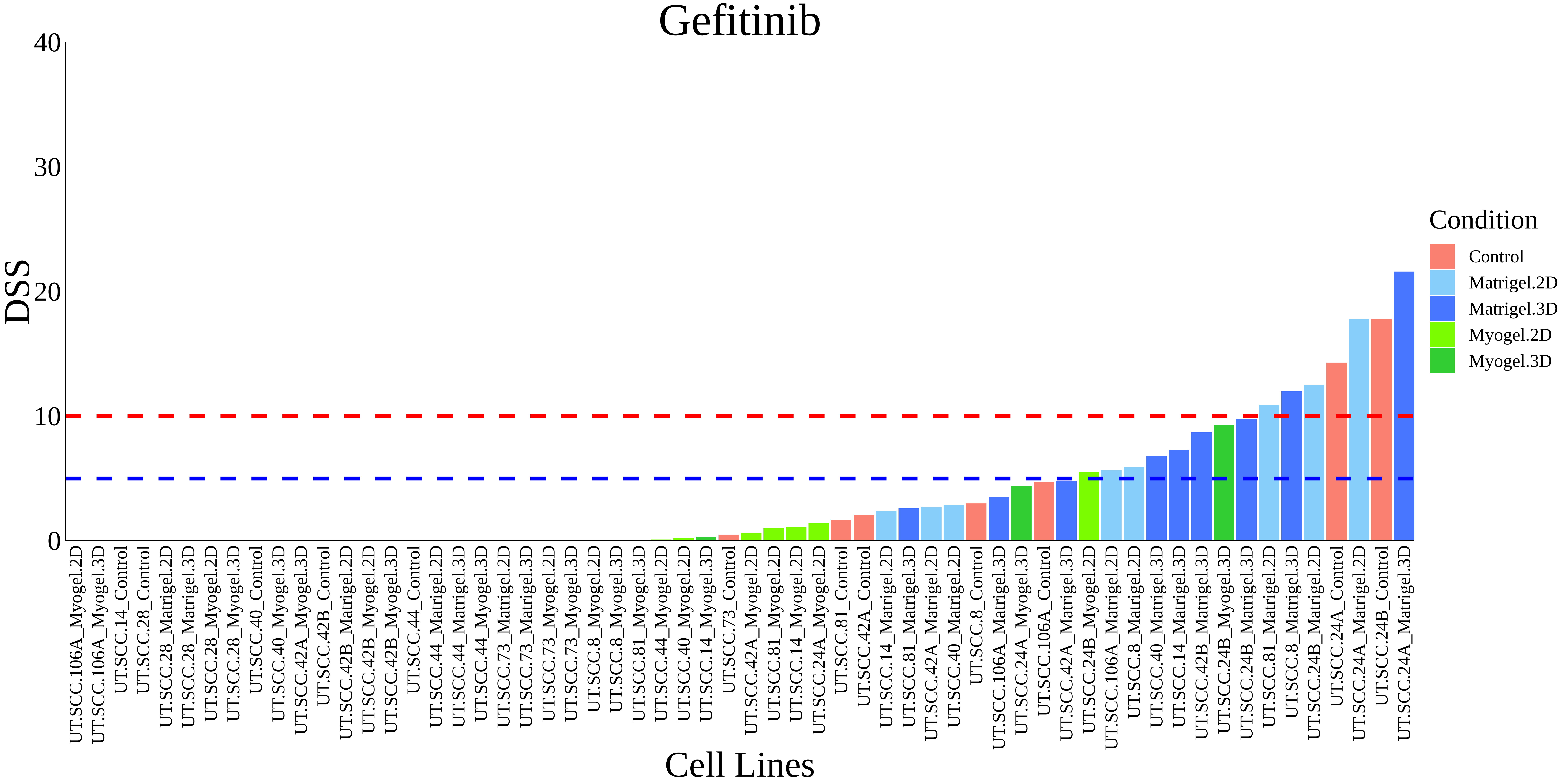

Gefitinib
DSS
0
10
20
30
40
Cell Lines
Condition
Control
Matrigel.2D
Matrigel.3D
Myogel.2D
Myogel.3D
UT.SCC.106A_Myogel.2D
UT.SCC.106A_Myogel.3D
UT.SCC.14_Control
UT.SCC.28_Control
UT.SCC.28_Matrigel.2D
UT.SCC.28_Matrigel.3D
UT.SCC.28_Myogel.2D
UT.SCC.28_Myogel.3D
UT.SCC.40_Control
UT.SCC.40_Myogel.3D
UT.SCC.42A_Myogel.3D
UT.SCC.42B_Control
UT.SCC.42B_Matrigel.2D
UT.SCC.42B_Myogel.2D
UT.SCC.42B_Myogel.3D
UT.SCC.44_Control
UT.SCC.44_Matrigel.2D
UT.SCC.44_Matrigel.3D
UT.SCC.44_Myogel.3D
UT.SCC.73_Matrigel.2D
UT.SCC.73_Matrigel.3D
UT.SCC.73_Myogel.2D
UT.SCC.73_Myogel.3D
UT.SCC.8_Myogel.2D
UT.SCC.8_Myogel.3D
UT.SCC.81_Myogel.3D
UT.SCC.44_Myogel.2D
UT.SCC.40_Myogel.2D
UT.SCC.14_Myogel.3D
UT.SCC.73_Control
UT.SCC.42A_Myogel.2D
UT.SCC.81_Myogel.2D
UT.SCC.14_Myogel.2D
UT.SCC.24A_Myogel.2D
UT.SCC.81_Control
UT.SCC.42A_Control
UT.SCC.14_Matrigel.2D
UT.SCC.81_Matrigel.3D
UT.SCC.42A_Matrigel.2D
UT.SCC.40_Matrigel.2D
UT.SCC.8_Control
UT.SCC.106A_Matrigel.3D
UT.SCC.24A_Myogel.3D
UT.SCC.106A_Control
UT.SCC.42A_Matrigel.3D
UT.SCC.24B_Myogel.2D
UT.SCC.106A_Matrigel.2D
UT.SCC.8_Matrigel.2D
UT.SCC.40_Matrigel.3D
UT.SCC.14_Matrigel.3D
UT.SCC.42B_Matrigel.3D
UT.SCC.24B_Myogel.3D
UT.SCC.24B_Matrigel.3D
UT.SCC.81_Matrigel.2D
UT.SCC.8_Matrigel.3D
UT.SCC.24B_Matrigel.2D
UT.SCC.24A_Control
UT.SCC.24A_Matrigel.2D
UT.SCC.24B_Control
UT.SCC.24A_Matrigel.3D

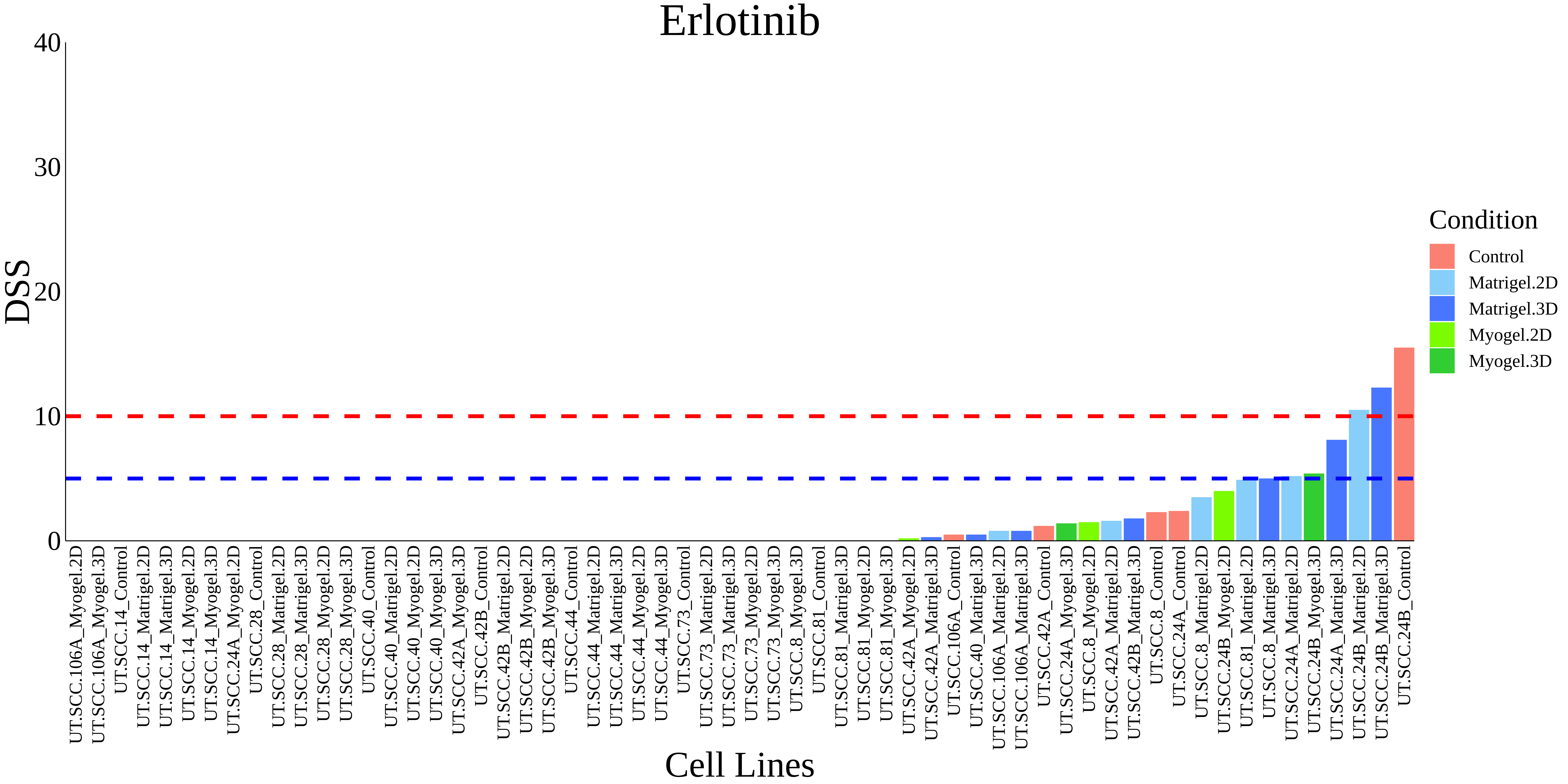

Erlotinib
40
30
20
10
0
DSS
Cell Lines
Condition
Control
Matrigel.2D
Matrigel.3D
Myogel.2D
Myogel.3D
UT.SCC.106A_Myogel.2D
UT.SCC.106A_Myogel.3D
UT.SCC.14_Control
UT.SCC.14_Matrigel.2D
UT.SCC.14_Matrigel.3D
UT.SCC.14_Myogel.2D
UT.SCC.14_Myogel.3D
UT.SCC.24A_Myogel.2D
UT.SCC.28_Control
UT.SCC.28_Matrigel.2D
UT.SCC.28_Matrigel.3D
UT.SCC.28_Myogel.2D
UT.SCC.28_Myogel.3D
UT.SCC.40_Control
UT.SCC.40_Matrigel.2D
UT.SCC.40_Myogel.2D
UT.SCC.40_Myogel.3D
UT.SCC.42A_Myogel.3D
UT.SCC.42B_Control
UT.SCC.42B_Matrigel.2D
UT.SCC.42B_Myogel.2D
UT.SCC.42B_Myogel.3D
UT.SCC.44_Control
UT.SCC.44_Matrigel.2D
UT.SCC.44_Matrigel.3D
UT.SCC.44_Myogel.2D
UT.SCC.44_Myogel.3D
UT.SCC.73_Control
UT.SCC.73_Matrigel.2D
UT.SCC.73_Matrigel.3D
UT.SCC.73_Myogel.2D
UT.SCC.73_Myogel.3D
UT.SCC.8_Myogel.3D
UT.SCC.81_Control
UT.SCC.81_Matrigel.3D
UT.SCC.81_Myogel.2D
UT.SCC.81_Myogel.3D
UT.SCC.42A_Myogel.2D
UT.SCC.42A_Matrigel.3D
UT.SCC.106A_Control
UT.SCC.40_Matrigel.3D
UT.SCC.106A_Matrigel.2D
UT.SCC.106A_Matrigel.3D
UT.SCC.42A_Control
UT.SCC.24A_Myogel.3D
UT.SCC.8_Myogel.2D
UT.SCC.42A_Matrigel.2D
UT.SCC.42B_Matrigel.3D
UT.SCC.8_Control
UT.SCC.24A_Control
UT.SCC.8_Matrigel.2D
UT.SCC.24B_Myogel.2D
UT.SCC.81_Matrigel.2D
UT.SCC.8_Matrigel.3D
UT.SCC.24A_Matrigel.2D
UT.SCC.24B_Myogel.3D
UT.SCC.24A_Matrigel.3D
UT.SCC.24B_Matrigel.2D
UT.SCC.24B_Matrigel.3D
UT.SCC.24B_Control

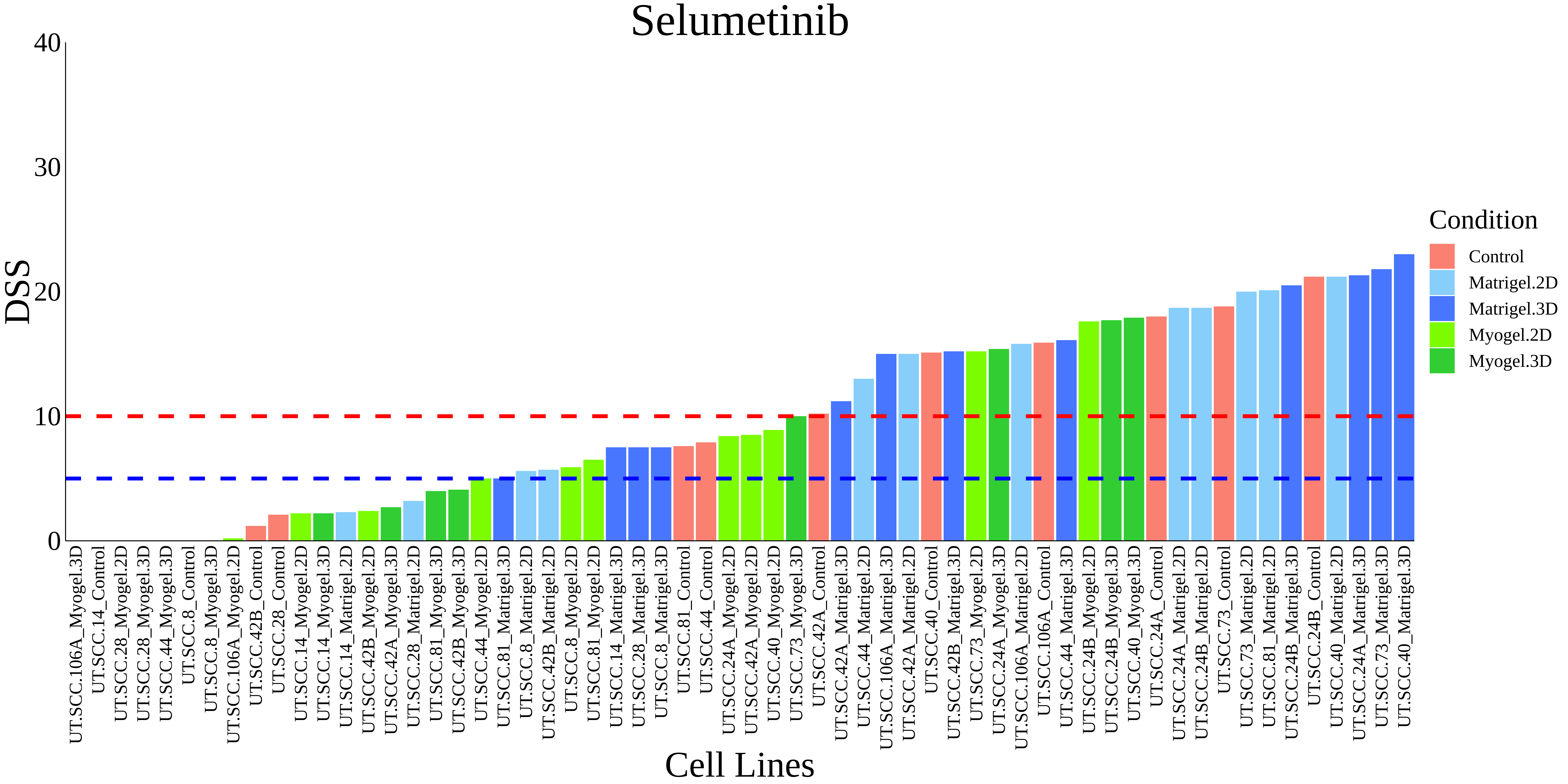

Selumetinib
DSS
0
10
20
30
40
Cell Lines
Condition
Control
Matrigel.2D
Matrigel.3D
Myogel.2D
Myogel.3D
UT.SCC.106A_Myogel.3D
UT.SCC.14_Control
UT.SCC.28_Myogel.2D
UT.SCC.28_Myogel.3D
UT.SCC.44_Myogel.3D
UT.SCC.8_Control
UT.SCC.8_Myogel.3D
UT.SCC.106A_Myogel.2D
UT.SCC.42B_Control
UT.SCC.28_Control
UT.SCC.14_Myogel.2D
UT.SCC.14_Myogel.3D
UT.SCC.14_Matrigel.2D
UT.SCC.42B_Myogel.2D
UT.SCC.42A_Myogel.3D
UT.SCC.28_Matrigel.2D
UT.SCC.81_Myogel.3D
UT.SCC.42B_Myogel.3D
UT.SCC.44_Myogel.2D
UT.SCC.81_Matrigel.3D
UT.SCC.8_Matrigel.2D
UT.SCC.42B_Matrigel.2D
UT.SCC.8_Myogel.2D
UT.SCC.81_Myogel.2D
UT.SCC.14_Matrigel.3D
UT.SCC.28_Matrigel.3D
UT.SCC.8_Matrigel.3D
UT.SCC.81_Control
UT.SCC.44_Control
UT.SCC.24A_Myogel.2D
UT.SCC.42A_Myogel.2D
UT.SCC.40_Myogel.2D
UT.SCC.73_Myogel.3D
UT.SCC.42A_Control
UT.SCC.42A_Matrigel.3D
UT.SCC.44_Matrigel.2D
UT.SCC.106A_Matrigel.3D
UT.SCC.42A_Matrigel.2D
UT.SCC.40_Control
UT.SCC.42B_Matrigel.3D
UT.SCC.73_Myogel.2D
UT.SCC.24A_Myogel.3D
UT.SCC.106A_Matrigel.2D
UT.SCC.106A_Control
UT.SCC.44_Matrigel.3D
UT.SCC.24B_Myogel.2D
UT.SCC.24B_Myogel.3D
UT.SCC.40_Myogel.3D
UT.SCC.24A_Control
UT.SCC.24A_Matrigel.2D
UT.SCC.24B_Matrigel.2D
UT.SCC.73_Control
UT.SCC.73_Matrigel.2D
UT.SCC.81_Matrigel.2D
UT.SCC.24B_Matrigel.3D
UT.SCC.24B_Control
UT.SCC.40_Matrigel.2D
UT.SCC.24A_Matrigel.3D
UT.SCC.73_Matrigel.3D
UT.SCC.40_Matrigel.3D

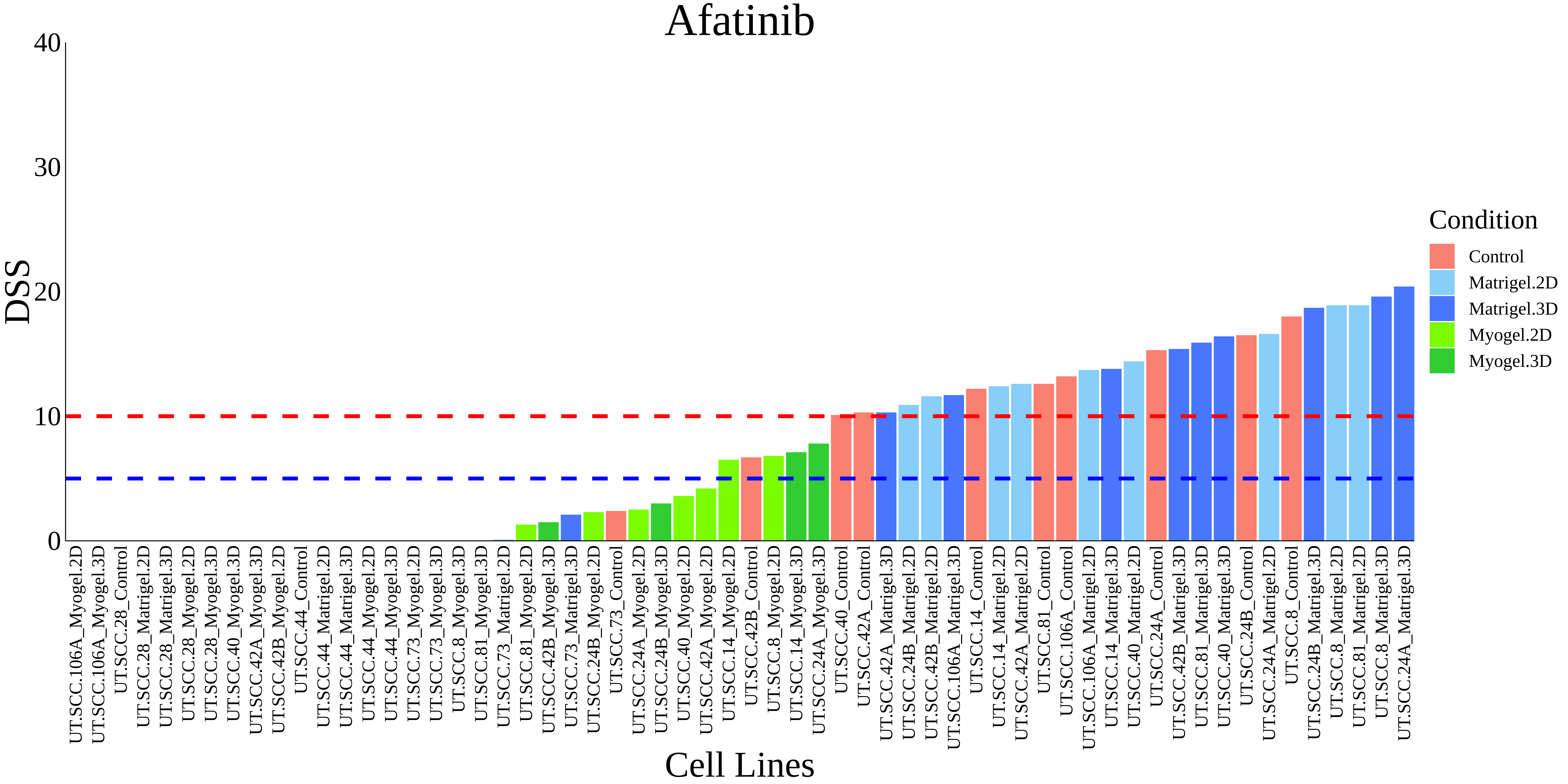

Afatinib
DSS
0
10
20
30
40
Cell Lines
Condition
Control
Matrigel.2D
Matrigel.3D
Myogel.2D
Myogel.3D
UT.SCC.106A_Myogel.2D
UT.SCC.106A_Myogel.3D
UT.SCC.28_Control
UT.SCC.28_Matrigel.2D
UT.SCC.28_Matrigel.3D
UT.SCC.28_Myogel.2D
UT.SCC.28_Myogel.3D
UT.SCC.40_Myogel.3D
UT.SCC.42A_Myogel.3D
UT.SCC.42B_Myogel.2D
UT.SCC.44_Control
UT.SCC.44_Matrigel.2D
UT.SCC.44_Matrigel.3D
UT.SCC.44_Myogel.2D
UT.SCC.44_Myogel.3D
UT.SCC.73_Myogel.2D
UT.SCC.73_Myogel.3D
UT.SCC.8_Myogel.3D
UT.SCC.81_Myogel.3D
UT.SCC.73_Matrigel.2D
UT.SCC.81_Myogel.2D
UT.SCC.42B_Myogel.3D
UT.SCC.73_Matrigel.3D
UT.SCC.24B_Myogel.2D
UT.SCC.73_Control
UT.SCC.24A_Myogel.2D
UT.SCC.24B_Myogel.3D
UT.SCC.40_Myogel.2D
UT.SCC.42A_Myogel.2D
UT.SCC.14_Myogel.2D
UT.SCC.42B_Control
UT.SCC.8_Myogel.2D
UT.SCC.14_Myogel.3D
UT.SCC.24A_Myogel.3D
UT.SCC.40_Control
UT.SCC.42A_Control
UT.SCC.42A_Matrigel.3D
UT.SCC.24B_Matrigel.2D
UT.SCC.42B_Matrigel.2D
UT.SCC.106A_Matrigel.3D
UT.SCC.14_Control
UT.SCC.14_Matrigel.2D
UT.SCC.42A_Matrigel.2D
UT.SCC.81_Control
UT.SCC.106A_Control
UT.SCC.106A_Matrigel.2D
UT.SCC.14_Matrigel.3D
UT.SCC.40_Matrigel.2D
UT.SCC.24A_Control
UT.SCC.42B_Matrigel.3D
UT.SCC.81_Matrigel.3D
UT.SCC.40_Matrigel.3D
UT.SCC.24B_Control
UT.SCC.24A_Matrigel.2D
UT.SCC.8_Control
UT.SCC.24B_Matrigel.3D
UT.SCC.8_Matrigel.2D
UT.SCC.81_Matrigel.2D
UT.SCC.8_Matrigel.3D
UT.SCC.24A_Matrigel.3D

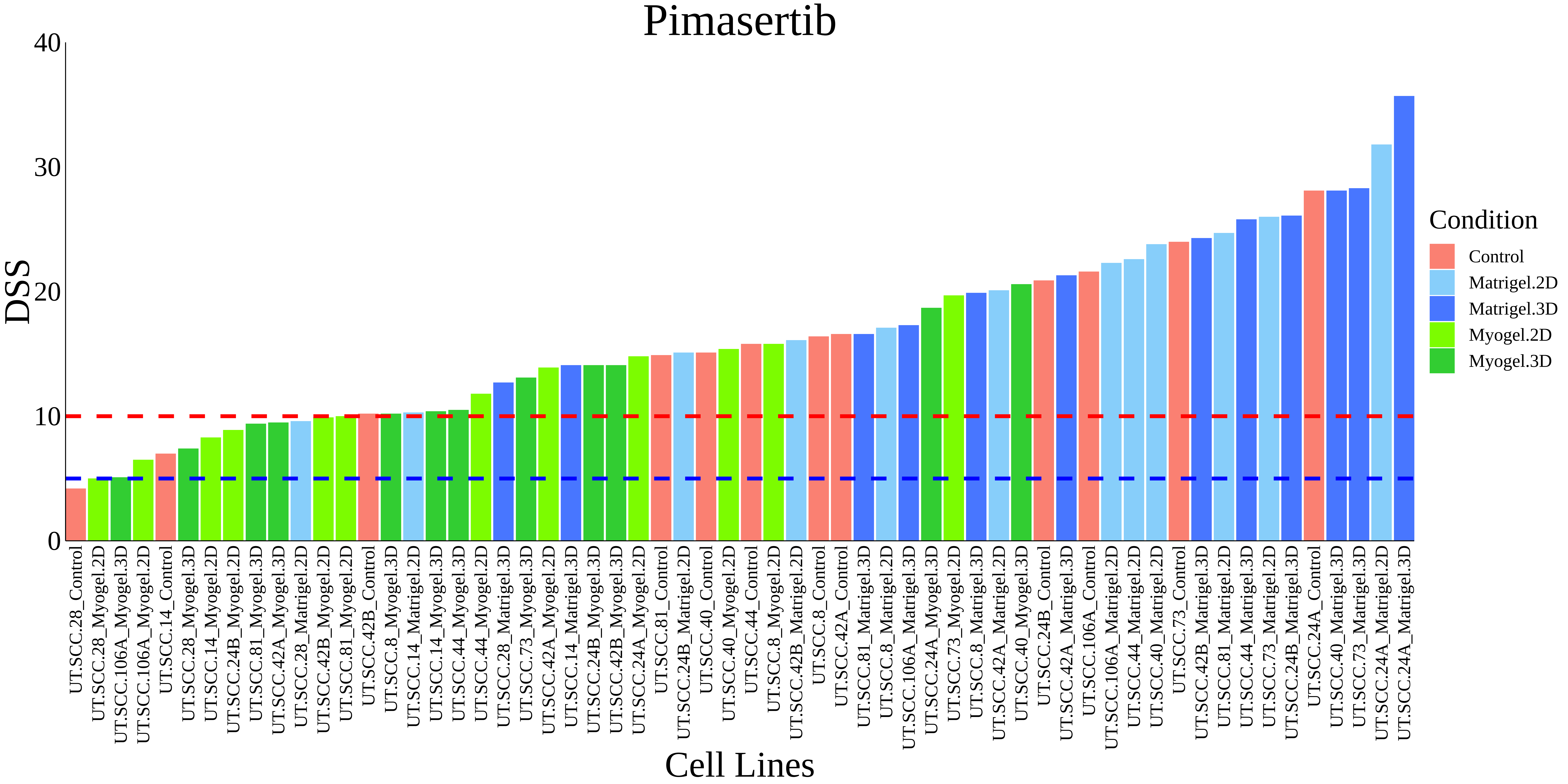
Pimasertib
DSS
0
10
20
30
40
Cell Lines
Condition
Control
Matrigel.2D
Matrigel.3D
Myogel.2D
Myogel.3D
UT.SCC.28_Control
UT.SCC.28_Myogel.2D
UT.SCC.106A_Myogel.3D
UT.SCC.106A_Myogel.2D
UT.SCC.14_Control
UT.SCC.28_Myogel.3D
UT.SCC.14_Myogel.2D
UT.SCC.24B_Myogel.2D
UT.SCC.81_Myogel.3D
UT.SCC.42A_Myogel.3D
UT.SCC.28_Matrigel.2D
UT.SCC.42B_Myogel.2D
UT.SCC.81_Myogel.2D
UT.SCC.42B_Control
UT.SCC.8_Myogel.3D
UT.SCC.14_Matrigel.2D
UT.SCC.14_Myogel.3D
UT.SCC.44_Myogel.3D
UT.SCC.44_Myogel.2D
UT.SCC.28_Matrigel.3D
UT.SCC.73_Myogel.3D
UT.SCC.42A_Myogel.2D
UT.SCC.14_Matrigel.3D
UT.SCC.24B_Myogel.3D
UT.SCC.42B_Myogel.3D
UT.SCC.24A_Myogel.2D
UT.SCC.81_Control
UT.SCC.24B_Matrigel.2D
UT.SCC.40_Control
UT.SCC.40_Myogel.2D
UT.SCC.44_Control
UT.SCC.8_Myogel.2D
UT.SCC.42B_Matrigel.2D
UT.SCC.8_Control
UT.SCC.42A_Control
UT.SCC.81_Matrigel.3D
UT.SCC.8_Matrigel.2D
UT.SCC.106A_Matrigel.3D
UT.SCC.24A_Myogel.3D
UT.SCC.73_Myogel.2D
UT.SCC.8_Matrigel.3D
UT.SCC.42A_Matrigel.2D
UT.SCC.40_Myogel.3D
UT.SCC.24B_Control
UT.SCC.42A_Matrigel.3D
UT.SCC.106A_Control
UT.SCC.106A_Matrigel.2D
UT.SCC.44_Matrigel.2D
UT.SCC.40_Matrigel.2D
UT.SCC.73_Control
UT.SCC.42B_Matrigel.3D
UT.SCC.81_Matrigel.2D
UT.SCC.44_Matrigel.3D
UT.SCC.73_Matrigel.2D
UT.SCC.24B_Matrigel.3D
UT.SCC.24A_Control
UT.SCC.40_Matrigel.3D
UT.SCC.73_Matrigel.3D
UT.SCC.24A_Matrigel.2D
UT.SCC.24A_Matrigel.3D

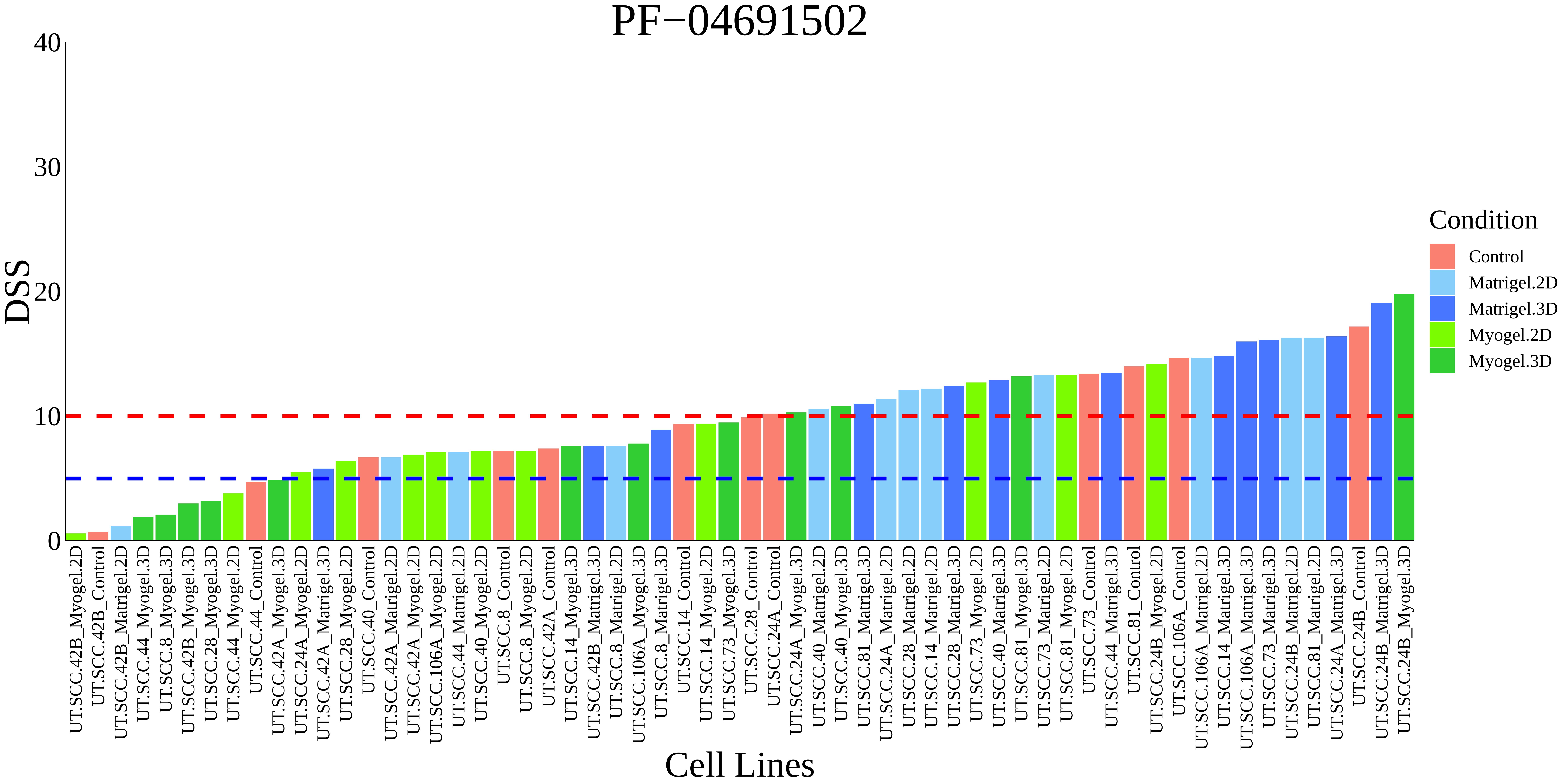
PF−04691502
DSS
Cell Lines
Condition
Control
Matrigel.2D
Matrigel.3D
Myogel.2D
Myogel.3D
0
10
20
30
40
UT.SCC.42B_Myogel.2D
UT.SCC.42B_Control
UT.SCC.42B_Matrigel.2D
UT.SCC.44_Myogel.3D
UT.SCC.8_Myogel.3D
UT.SCC.42B_Myogel.3D
UT.SCC.28_Myogel.3D
UT.SCC.44_Myogel.2D
UT.SCC.44_Control
UT.SCC.42A_Myogel.3D
UT.SCC.24A_Myogel.2D
UT.SCC.42A_Matrigel.3D
UT.SCC.28_Myogel.2D
UT.SCC.40_Control
UT.SCC.42A_Matrigel.2D
UT.SCC.42A_Myogel.2D
UT.SCC.106A_Myogel.2D
UT.SCC.44_Matrigel.2D
UT.SCC.40_Myogel.2D
UT.SCC.8_Control
UT.SCC.8_Myogel.2D
UT.SCC.42A_Control
UT.SCC.14_Myogel.3D
UT.SCC.42B_Matrigel.3D
UT.SCC.8_Matrigel.2D
UT.SCC.106A_Myogel.3D
UT.SCC.8_Matrigel.3D
UT.SCC.14_Control
UT.SCC.14_Myogel.2D
UT.SCC.73_Myogel.3D
UT.SCC.28_Control
UT.SCC.24A_Control
UT.SCC.24A_Myogel.3D
UT.SCC.40_Matrigel.2D
UT.SCC.40_Myogel.3D
UT.SCC.81_Matrigel.3D
UT.SCC.24A_Matrigel.2D
UT.SCC.28_Matrigel.2D
UT.SCC.14_Matrigel.2D
UT.SCC.28_Matrigel.3D
UT.SCC.73_Myogel.2D
UT.SCC.40_Matrigel.3D
UT.SCC.81_Myogel.3D
UT.SCC.73_Matrigel.2D
UT.SCC.81_Myogel.2D
UT.SCC.73_Control
UT.SCC.44_Matrigel.3D
UT.SCC.81_Control
UT.SCC.24B_Myogel.2D
UT.SCC.106A_Control
UT.SCC.106A_Matrigel.2D
UT.SCC.14_Matrigel.3D
UT.SCC.106A_Matrigel.3D
UT.SCC.73_Matrigel.3D
UT.SCC.24B_Matrigel.2D
UT.SCC.81_Matrigel.2D
UT.SCC.24A_Matrigel.3D
UT.SCC.24B_Control
UT.SCC.24B_Matrigel.3D
UT.SCC.24B_Myogel.3D

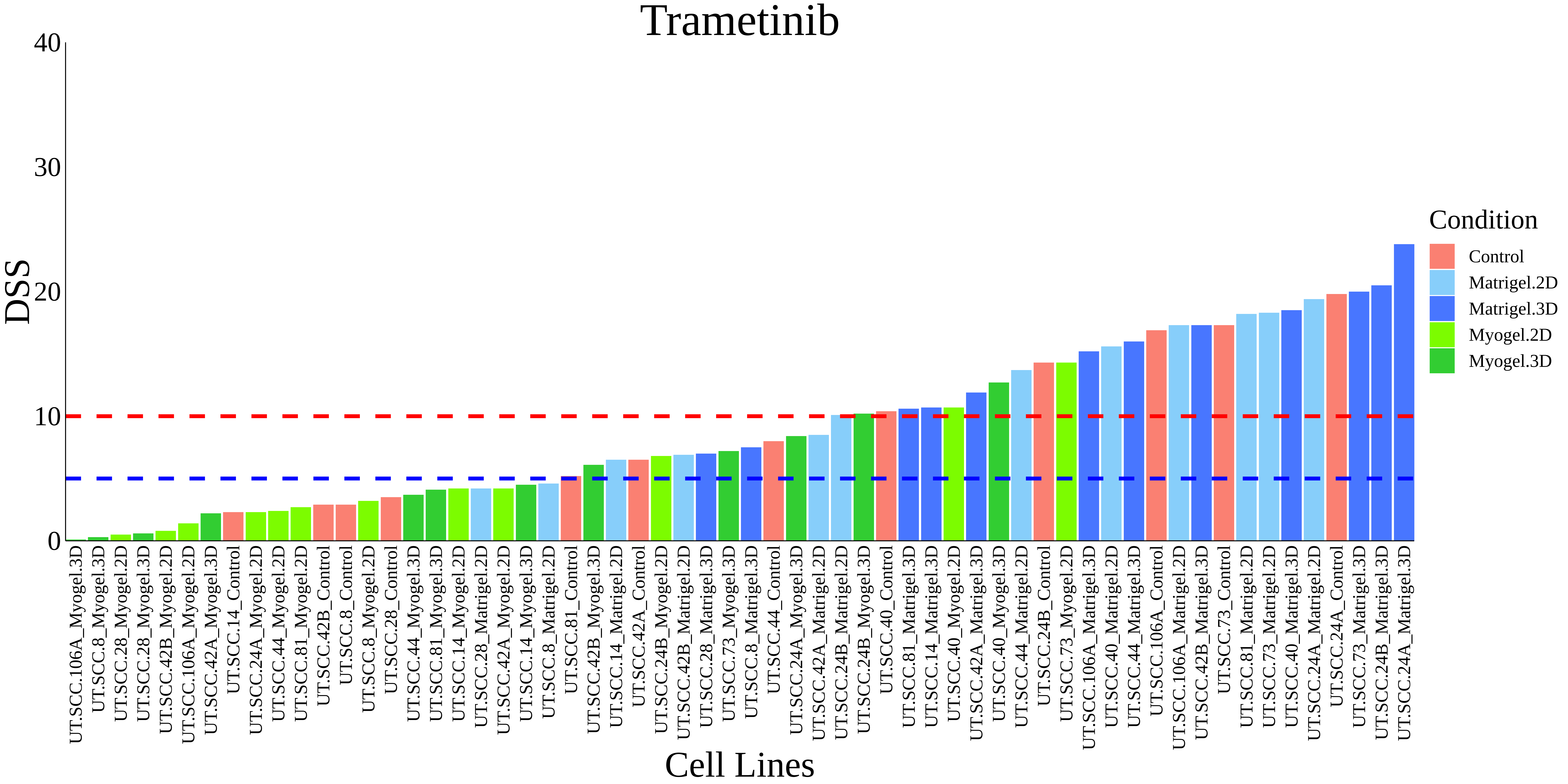
Trametinib
DSS
Cell Lines
Condition
Control
Matrigel.2D
Matrigel.3D
Myogel.2D
Myogel.3D
0
10
20
30
40
UT.SCC.106A_Myogel.3D
UT.SCC.8_Myogel.3D
UT.SCC.28_Myogel.2D
UT.SCC.28_Myogel.3D
UT.SCC.42B_Myogel.2D
UT.SCC.106A_Myogel.2D
UT.SCC.42A_Myogel.3D
UT.SCC.14_Control
UT.SCC.24A_Myogel.2D
UT.SCC.44_Myogel.2D
UT.SCC.81_Myogel.2D
UT.SCC.42B_Control
UT.SCC.8_Control
UT.SCC.8_Myogel.2D
UT.SCC.28_Control
UT.SCC.44_Myogel.3D
UT.SCC.81_Myogel.3D
UT.SCC.14_Myogel.2D
UT.SCC.28_Matrigel.2D
UT.SCC.42A_Myogel.2D
UT.SCC.14_Myogel.3D
UT.SCC.8_Matrigel.2D
UT.SCC.81_Control
UT.SCC.42B_Myogel.3D
UT.SCC.14_Matrigel.2D
UT.SCC.42A_Control
UT.SCC.24B_Myogel.2D
UT.SCC.42B_Matrigel.2D
UT.SCC.28_Matrigel.3D
UT.SCC.73_Myogel.3D
UT.SCC.8_Matrigel.3D
UT.SCC.44_Control
UT.SCC.24A_Myogel.3D
UT.SCC.42A_Matrigel.2D
UT.SCC.24B_Matrigel.2D
UT.SCC.24B_Myogel.3D
UT.SCC.40_Control
UT.SCC.81_Matrigel.3D
UT.SCC.14_Matrigel.3D
UT.SCC.40_Myogel.2D
UT.SCC.42A_Matrigel.3D
UT.SCC.40_Myogel.3D
UT.SCC.44_Matrigel.2D
UT.SCC.24B_Control
UT.SCC.73_Myogel.2D
UT.SCC.106A_Matrigel.3D
UT.SCC.40_Matrigel.2D
UT.SCC.44_Matrigel.3D
UT.SCC.106A_Control
UT.SCC.106A_Matrigel.2D
UT.SCC.42B_Matrigel.3D
UT.SCC.73_Control
UT.SCC.81_Matrigel.2D
UT.SCC.73_Matrigel.2D
UT.SCC.40_Matrigel.3D
UT.SCC.24A_Matrigel.2D
UT.SCC.24A_Control
UT.SCC.73_Matrigel.3D
UT.SCC.24B_Matrigel.3D
UT.SCC.24A_Matrigel.3D

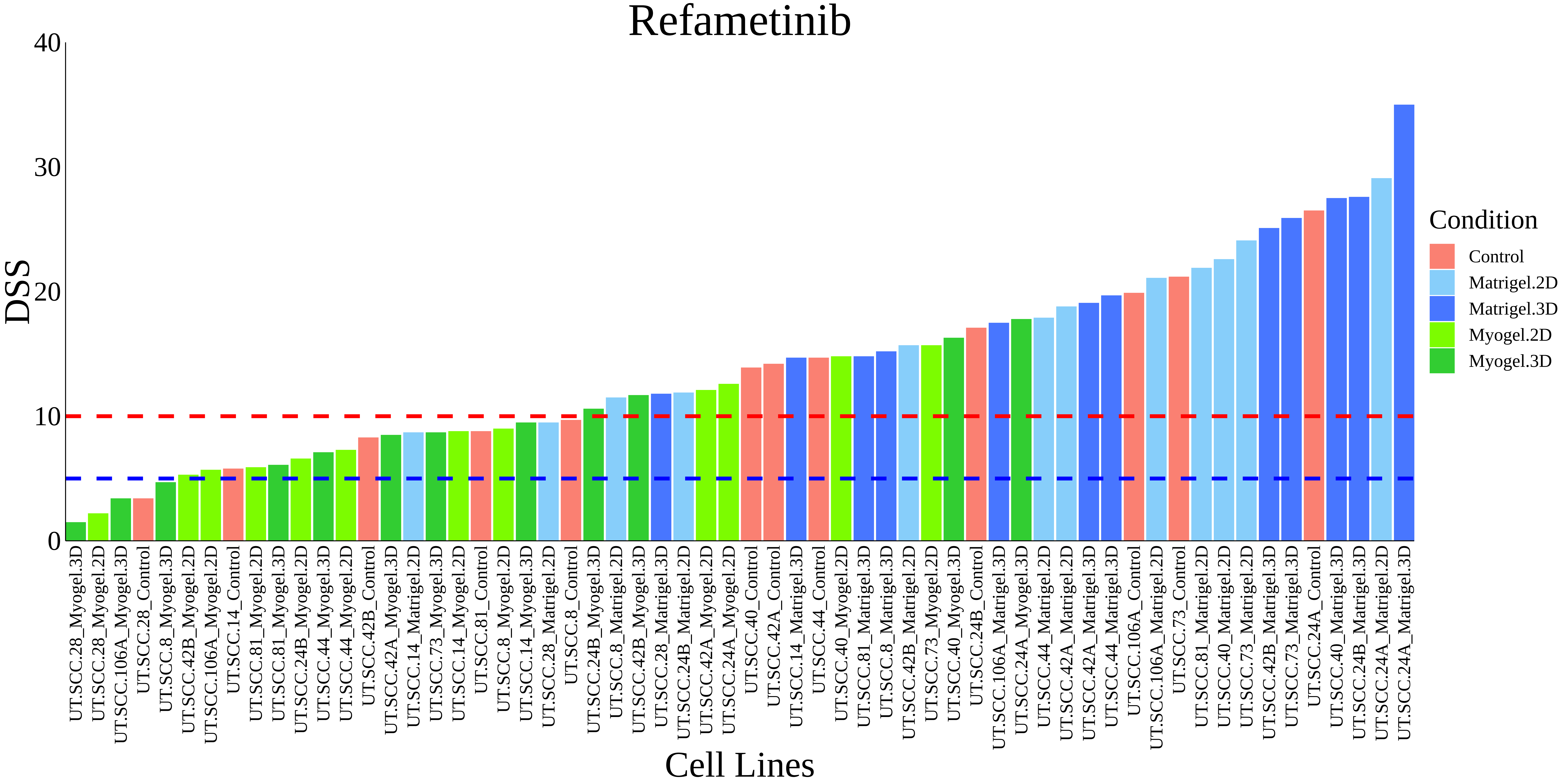

Refametinib
DSS
0
10
20
30
40
Cell Lines
Condition
Control
Matrigel.2D
Matrigel.3D
Myogel.2D
Myogel.3D
UT.SCC.28_Myogel.3D
UT.SCC.28_Myogel.2D
UT.SCC.106A_Myogel.3D
UT.SCC.28_Control
UT.SCC.8_Myogel.3D
UT.SCC.42B_Myogel.2D
UT.SCC.106A_Myogel.2D
UT.SCC.14_Control
UT.SCC.81_Myogel.2D
UT.SCC.81_Myogel.3D
UT.SCC.24B_Myogel.2D
UT.SCC.44_Myogel.3D
UT.SCC.44_Myogel.2D
UT.SCC.42B_Control
UT.SCC.42A_Myogel.3D
UT.SCC.14_Matrigel.2D
UT.SCC.73_Myogel.3D
UT.SCC.14_Myogel.2D
UT.SCC.81_Control
UT.SCC.8_Myogel.2D
UT.SCC.14_Myogel.3D
UT.SCC.28_Matrigel.2D
UT.SCC.8_Control
UT.SCC.24B_Myogel.3D
UT.SCC.8_Matrigel.2D
UT.SCC.42B_Myogel.3D
UT.SCC.28_Matrigel.3D
UT.SCC.24B_Matrigel.2D
UT.SCC.42A_Myogel.2D
UT.SCC.24A_Myogel.2D
UT.SCC.40_Control
UT.SCC.42A_Control
UT.SCC.14_Matrigel.3D
UT.SCC.44_Control
UT.SCC.40_Myogel.2D
UT.SCC.81_Matrigel.3D
UT.SCC.8_Matrigel.3D
UT.SCC.42B_Matrigel.2D
UT.SCC.73_Myogel.2D
UT.SCC.40_Myogel.3D
UT.SCC.24B_Control
UT.SCC.106A_Matrigel.3D
UT.SCC.24A_Myogel.3D
UT.SCC.44_Matrigel.2D
UT.SCC.42A_Matrigel.2D
UT.SCC.42A_Matrigel.3D
UT.SCC.44_Matrigel.3D
UT.SCC.106A_Control
UT.SCC.106A_Matrigel.2D
UT.SCC.73_Control
UT.SCC.81_Matrigel.2D
UT.SCC.40_Matrigel.2D
UT.SCC.73_Matrigel.2D
UT.SCC.42B_Matrigel.3D
UT.SCC.73_Matrigel.3D
UT.SCC.24A_Control
UT.SCC.40_Matrigel.3D
UT.SCC.24B_Matrigel.3D
UT.SCC.24A_Matrigel.2D
UT.SCC.24A_Matrigel.3D

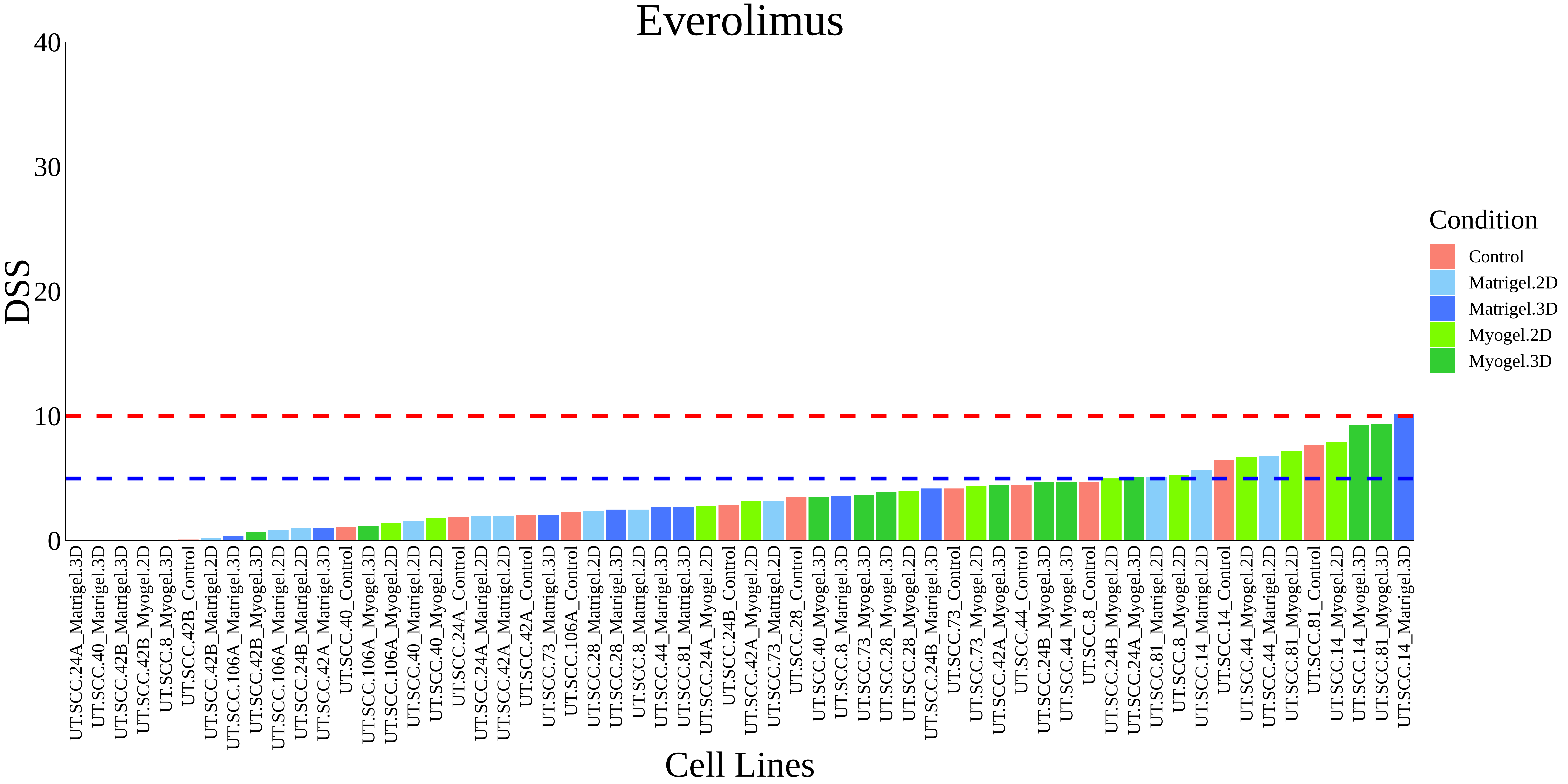

Everolimus
DSS
40
30
20
10
0
Condition
Control
Matrigel.2D
Matrigel.3D
Myogel.2D
Myogel.3D
Cell Lines
UT.SCC.24A_Matrigel.3D
UT.SCC.40_Matrigel.3D
UT.SCC.42B_Matrigel.3D
UT.SCC.42B_Myogel.2D
UT.SCC.8_Myogel.3D
UT.SCC.42B_Control
UT.SCC.42B_Matrigel.2D
UT.SCC.106A_Matrigel.3D
UT.SCC.42B_Myogel.3D
UT.SCC.106A_Matrigel.2D
UT.SCC.24B_Matrigel.2D
UT.SCC.42A_Matrigel.3D
UT.SCC.40_Control
UT.SCC.106A_Myogel.3D
UT.SCC.106A_Myogel.2D
UT.SCC.40_Matrigel.2D
UT.SCC.40_Myogel.2D
UT.SCC.24A_Control
UT.SCC.24A_Matrigel.2D
UT.SCC.42A_Matrigel.2D
UT.SCC.42A_Control
UT.SCC.73_Matrigel.3D
UT.SCC.106A_Control
UT.SCC.28_Matrigel.2D
UT.SCC.28_Matrigel.3D
UT.SCC.8_Matrigel.2D
UT.SCC.44_Matrigel.3D
UT.SCC.81_Matrigel.3D
UT.SCC.24A_Myogel.2D
UT.SCC.24B_Control
UT.SCC.42A_Myogel.2D
UT.SCC.73_Matrigel.2D
UT.SCC.28_Control
UT.SCC.40_Myogel.3D
UT.SCC.8_Matrigel.3D
UT.SCC.73_Myogel.3D
UT.SCC.28_Myogel.3D
UT.SCC.28_Myogel.2D
UT.SCC.24B_Matrigel.3D
UT.SCC.73_Control
UT.SCC.73_Myogel.2D
UT.SCC.42A_Myogel.3D
UT.SCC.44_Control
UT.SCC.24B_Myogel.3D
UT.SCC.44_Myogel.3D
UT.SCC.8_Control
UT.SCC.24B_Myogel.2D
UT.SCC.24A_Myogel.3D
UT.SCC.81_Matrigel.2D
UT.SCC.8_Myogel.2D
UT.SCC.14_Matrigel.2D
UT.SCC.14_Control
UT.SCC.44_Myogel.2D
UT.SCC.44_Matrigel.2D
UT.SCC.81_Myogel.2D
UT.SCC.81_Control
UT.SCC.14_Myogel.2D
UT.SCC.14_Myogel.3D
UT.SCC.81_Myogel.3D
UT.SCC.14_Matrigel.3D

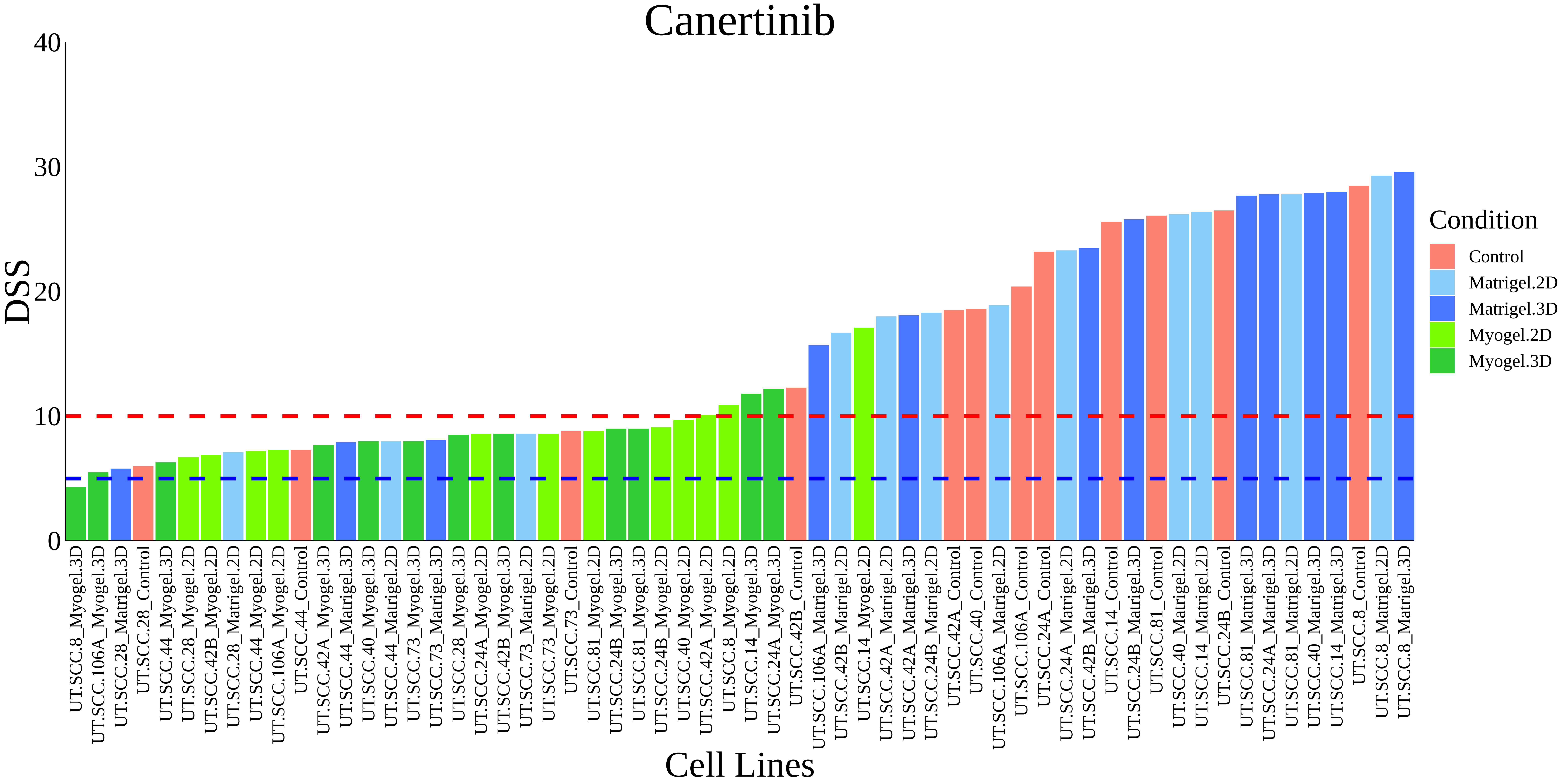

Canertinib
DSS
Cell Lines
Condition
Control
Matrigel.2D
Matrigel.3D
Myogel.2D
Myogel.3D
0
10
20
30
40
UT.SCC.8_Myogel.3D
UT.SCC.106A_Myogel.3D
UT.SCC.28_Matrigel.3D
UT.SCC.28_Control
UT.SCC.44_Myogel.3D
UT.SCC.28_Myogel.2D
UT.SCC.42B_Myogel.2D
UT.SCC.28_Matrigel.2D
UT.SCC.44_Myogel.2D
UT.SCC.106A_Myogel.2D
UT.SCC.44_Control
UT.SCC.42A_Myogel.3D
UT.SCC.44_Matrigel.3D
UT.SCC.40_Myogel.3D
UT.SCC.44_Matrigel.2D
UT.SCC.73_Myogel.3D
UT.SCC.73_Matrigel.3D
UT.SCC.28_Myogel.3D
UT.SCC.24A_Myogel.2D
UT.SCC.42B_Myogel.3D
UT.SCC.73_Matrigel.2D
UT.SCC.73_Myogel.2D
UT.SCC.73_Control
UT.SCC.81_Myogel.2D
UT.SCC.24B_Myogel.3D
UT.SCC.81_Myogel.3D
UT.SCC.24B_Myogel.2D
UT.SCC.40_Myogel.2D
UT.SCC.42A_Myogel.2D
UT.SCC.8_Myogel.2D
UT.SCC.14_Myogel.3D
UT.SCC.24A_Myogel.3D
UT.SCC.42B_Control
UT.SCC.106A_Matrigel.3D
UT.SCC.42B_Matrigel.2D
UT.SCC.14_Myogel.2D
UT.SCC.42A_Matrigel.2D
UT.SCC.42A_Matrigel.3D
UT.SCC.24B_Matrigel.2D
UT.SCC.42A_Control
UT.SCC.40_Control
UT.SCC.106A_Matrigel.2D
UT.SCC.106A_Control
UT.SCC.24A_Control
UT.SCC.24A_Matrigel.2D
UT.SCC.42B_Matrigel.3D
UT.SCC.14_Control
UT.SCC.24B_Matrigel.3D
UT.SCC.81_Control
UT.SCC.40_Matrigel.2D
UT.SCC.14_Matrigel.2D
UT.SCC.24B_Control
UT.SCC.81_Matrigel.3D
UT.SCC.24A_Matrigel.3D
UT.SCC.81_Matrigel.2D
UT.SCC.40_Matrigel.3D
UT.SCC.14_Matrigel.3D
UT.SCC.8_Control
UT.SCC.8_Matrigel.2D
UT.SCC.8_Matrigel.3D

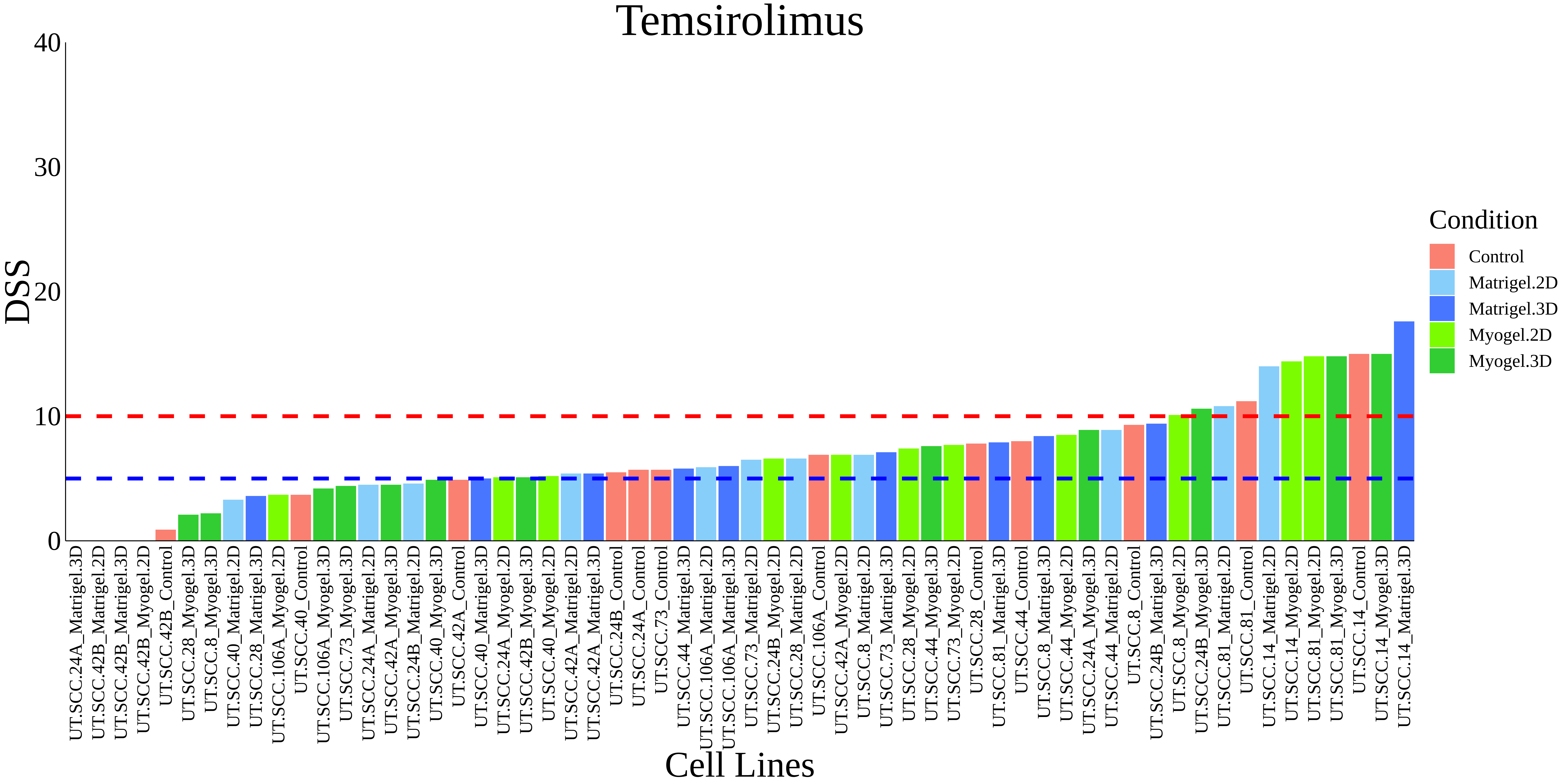

Temsirolimus
DSS
0
10
20
30
40
Cell Lines
Condition
Control
Matrigel.2D
Matrigel.3D
Myogel.2D
Myogel.3D
UT.SCC.24A_Matrigel.3D
UT.SCC.42B_Matrigel.2D
UT.SCC.42B_Matrigel.3D
UT.SCC.42B_Myogel.2D
UT.SCC.42B_Control
UT.SCC.28_Myogel.3D
UT.SCC.8_Myogel.3D
UT.SCC.40_Matrigel.2D
UT.SCC.28_Matrigel.3D
UT.SCC.106A_Myogel.2D
UT.SCC.40_Control
UT.SCC.106A_Myogel.3D
UT.SCC.73_Myogel.3D
UT.SCC.24A_Matrigel.2D
UT.SCC.42A_Myogel.3D
UT.SCC.24B_Matrigel.2D
UT.SCC.40_Myogel.3D
UT.SCC.42A_Control
UT.SCC.40_Matrigel.3D
UT.SCC.24A_Myogel.2D
UT.SCC.42B_Myogel.3D
UT.SCC.40_Myogel.2D
UT.SCC.42A_Matrigel.2D
UT.SCC.42A_Matrigel.3D
UT.SCC.24B_Control
UT.SCC.24A_Control
UT.SCC.73_Control
UT.SCC.44_Matrigel.3D
UT.SCC.106A_Matrigel.2D
UT.SCC.106A_Matrigel.3D
UT.SCC.73_Matrigel.2D
UT.SCC.24B_Myogel.2D
UT.SCC.28_Matrigel.2D
UT.SCC.106A_Control
UT.SCC.42A_Myogel.2D
UT.SCC.8_Matrigel.2D
UT.SCC.73_Matrigel.3D
UT.SCC.28_Myogel.2D
UT.SCC.44_Myogel.3D
UT.SCC.73_Myogel.2D
UT.SCC.28_Control
UT.SCC.81_Matrigel.3D
UT.SCC.44_Control
UT.SCC.8_Matrigel.3D
UT.SCC.44_Myogel.2D
UT.SCC.24A_Myogel.3D
UT.SCC.44_Matrigel.2D
UT.SCC.8_Control
UT.SCC.24B_Matrigel.3D
UT.SCC.8_Myogel.2D
UT.SCC.24B_Myogel.3D
UT.SCC.81_Matrigel.2D
UT.SCC.81_Control
UT.SCC.14_Matrigel.2D
UT.SCC.14_Myogel.2D
UT.SCC.81_Myogel.2D
UT.SCC.81_Myogel.3D
UT.SCC.14_Control
UT.SCC.14_Myogel.3D
UT.SCC.14_Matrigel.3D

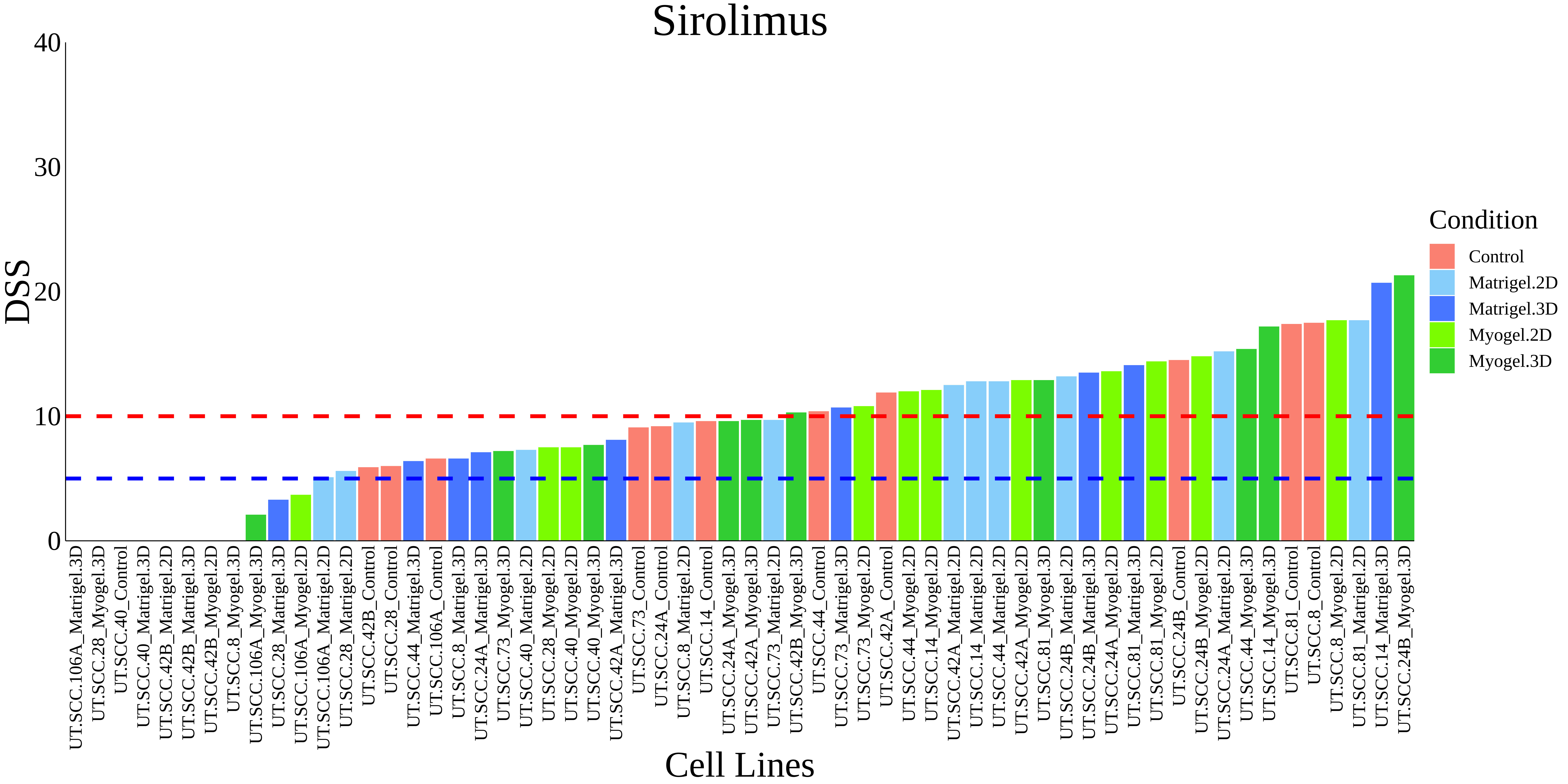
Sirolimus
DSS
40
30
20
10
0
Cell Lines
Condition
Control
Matrigel.2D
Matrigel.3D
Myogel.2D
Myogel.3D
UT.SCC.106A_Matrigel.3D
UT.SCC.28_Myogel.3D
UT.SCC.40_Control
UT.SCC.40_Matrigel.3D
UT.SCC.42B_Matrigel.2D
UT.SCC.42B_Matrigel.3D
UT.SCC.42B_Myogel.2D
UT.SCC.8_Myogel.3D
UT.SCC.106A_Myogel.3D
UT.SCC.28_Matrigel.3D
UT.SCC.106A_Myogel.2D
UT.SCC.106A_Matrigel.2D
UT.SCC.28_Matrigel.2D
UT.SCC.42B_Control
UT.SCC.28_Control
UT.SCC.44_Matrigel.3D
UT.SCC.106A_Control
UT.SCC.8_Matrigel.3D
UT.SCC.24A_Matrigel.3D
UT.SCC.73_Myogel.3D
UT.SCC.40_Matrigel.2D
UT.SCC.28_Myogel.2D
UT.SCC.40_Myogel.2D
UT.SCC.40_Myogel.3D
UT.SCC.42A_Matrigel.3D
UT.SCC.73_Control
UT.SCC.24A_Control
UT.SCC.8_Matrigel.2D
UT.SCC.14_Control
UT.SCC.24A_Myogel.3D
UT.SCC.42A_Myogel.3D
UT.SCC.73_Matrigel.2D
UT.SCC.42B_Myogel.3D
UT.SCC.44_Control
UT.SCC.73_Matrigel.3D
UT.SCC.73_Myogel.2D
UT.SCC.42A_Control
UT.SCC.44_Myogel.2D
UT.SCC.14_Myogel.2D
UT.SCC.42A_Matrigel.2D
UT.SCC.14_Matrigel.2D
UT.SCC.44_Matrigel.2D
UT.SCC.42A_Myogel.2D
UT.SCC.81_Myogel.3D
UT.SCC.24B_Matrigel.2D
UT.SCC.24B_Matrigel.3D
UT.SCC.24A_Myogel.2D
UT.SCC.81_Matrigel.3D
UT.SCC.81_Myogel.2D
UT.SCC.24B_Control
UT.SCC.24B_Myogel.2D
UT.SCC.24A_Matrigel.2D
UT.SCC.44_Myogel.3D
UT.SCC.14_Myogel.3D
UT.SCC.81_Control
UT.SCC.8_Control
UT.SCC.8_Myogel.2D
UT.SCC.81_Matrigel.2D
UT.SCC.14_Matrigel.3D
UT.SCC.24B_Myogel.3D

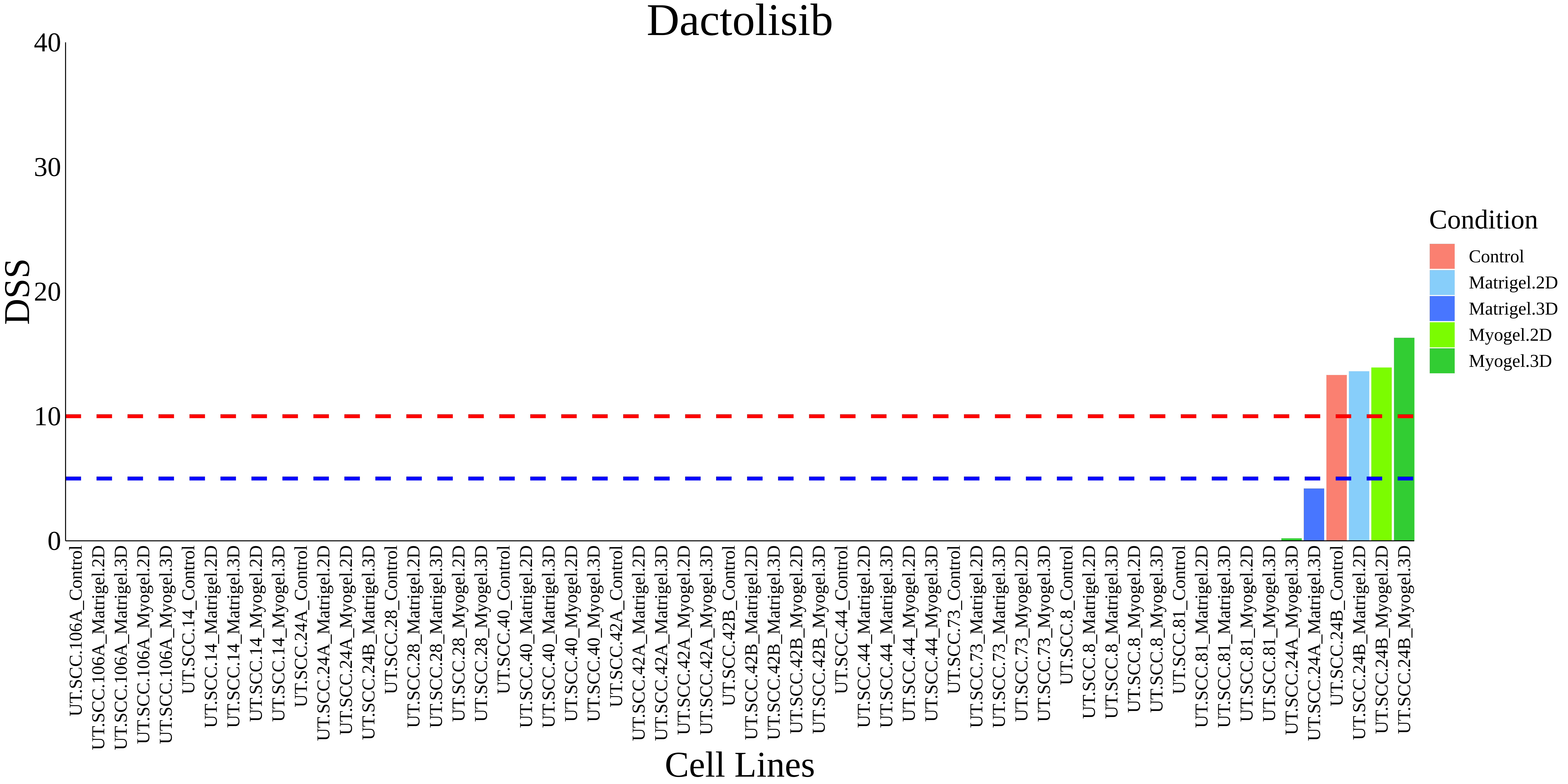

Dactolisib
DSS
40
30
20
10
0
Cell Lines
Condition
Control
Matrigel.2D
Matrigel.3D
Myogel.2D
Myogel.3D
UT.SCC.106A_Control
UT.SCC.106A_Matrigel.2D
UT.SCC.106A_Matrigel.3D
UT.SCC.106A_Myogel.2D
UT.SCC.106A_Myogel.3D
UT.SCC.14_Control
UT.SCC.14_Matrigel.2D
UT.SCC.14_Matrigel.3D
UT.SCC.14_Myogel.2D
UT.SCC.14_Myogel.3D
UT.SCC.24A_Control
UT.SCC.24A_Matrigel.2D
UT.SCC.24A_Myogel.2D
UT.SCC.24B_Matrigel.3D
UT.SCC.28_Control
UT.SCC.28_Matrigel.2D
UT.SCC.28_Matrigel.3D
UT.SCC.28_Myogel.2D
UT.SCC.28_Myogel.3D
UT.SCC.40_Control
UT.SCC.40_Matrigel.2D
UT.SCC.40_Matrigel.3D
UT.SCC.40_Myogel.2D
UT.SCC.40_Myogel.3D
UT.SCC.42A_Control
UT.SCC.42A_Matrigel.2D
UT.SCC.42A_Matrigel.3D
UT.SCC.42A_Myogel.2D
UT.SCC.42A_Myogel.3D
UT.SCC.42B_Control
UT.SCC.42B_Matrigel.2D
UT.SCC.42B_Matrigel.3D
UT.SCC.42B_Myogel.2D
UT.SCC.42B_Myogel.3D
UT.SCC.44_Control
UT.SCC.44_Matrigel.2D
UT.SCC.44_Matrigel.3D
UT.SCC.44_Myogel.2D
UT.SCC.44_Myogel.3D
UT.SCC.73_Control
UT.SCC.73_Matrigel.2D
UT.SCC.73_Matrigel.3D
UT.SCC.73_Myogel.2D
UT.SCC.73_Myogel.3D
UT.SCC.8_Control
UT.SCC.8_Matrigel.2D
UT.SCC.8_Matrigel.3D
UT.SCC.8_Myogel.2D
UT.SCC.8_Myogel.3D
UT.SCC.81_Control
UT.SCC.81_Matrigel.2D
UT.SCC.81_Matrigel.3D
UT.SCC.81_Myogel.2D
UT.SCC.81_Myogel.3D
UT.SCC.24A_Myogel.3D
UT.SCC.24A_Matrigel.3D
UT.SCC.24B_Control
UT.SCC.24B_Matrigel.2D
UT.SCC.24B_Myogel.2D
UT.SCC.24B_Myogel.3D

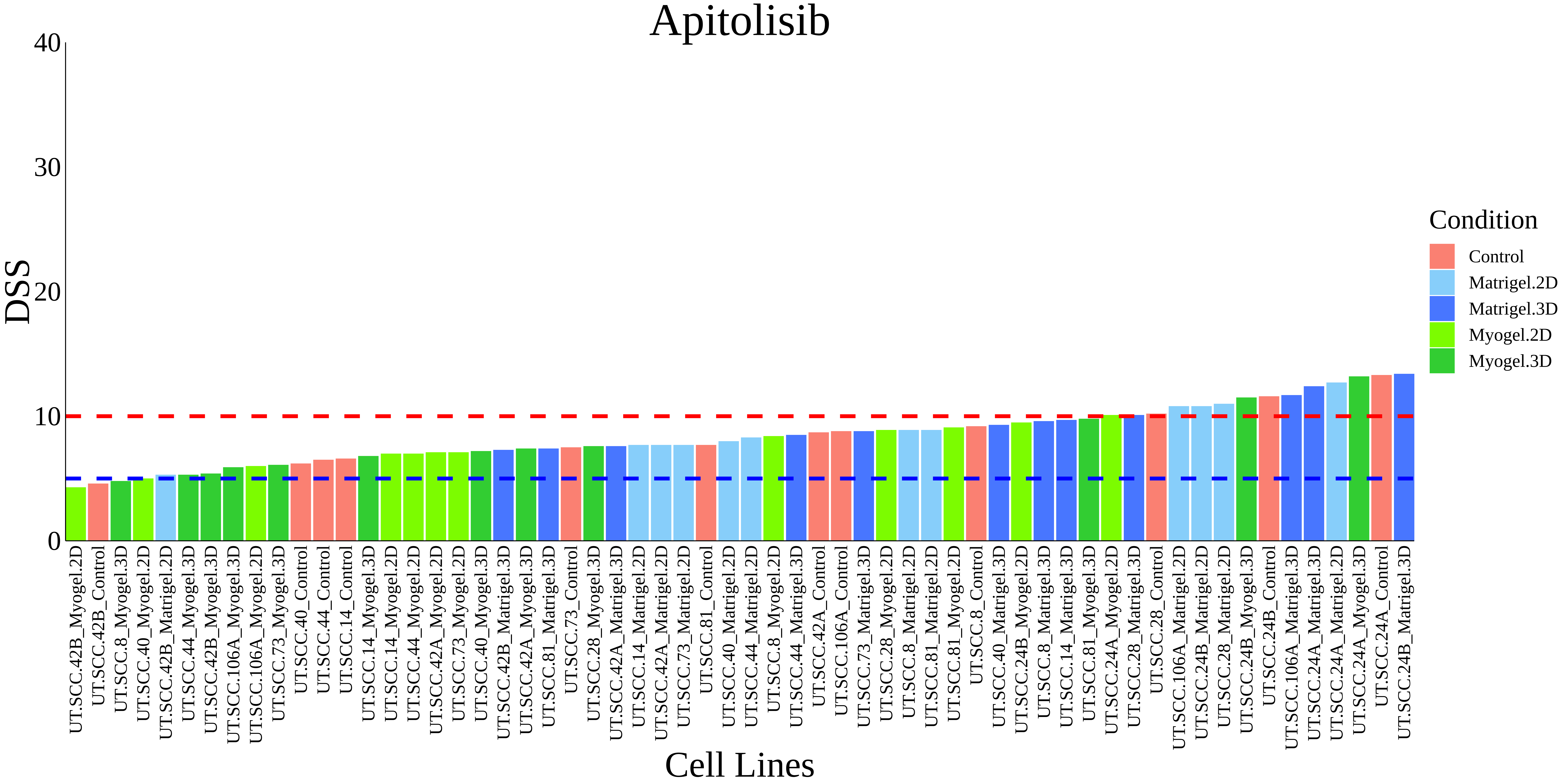

Apitolisib
DSS
0
10
20
30
40
Cell Lines
Condition
Control
Matrigel.2D
Matrigel.3D
Myogel.2D
Myogel.3D
UT.SCC.42B_Myogel.2D
UT.SCC.42B_Control
UT.SCC.8_Myogel.3D
UT.SCC.40_Myogel.2D
UT.SCC.42B_Matrigel.2D
UT.SCC.44_Myogel.3D
UT.SCC.42B_Myogel.3D
UT.SCC.106A_Myogel.3D
UT.SCC.106A_Myogel.2D
UT.SCC.73_Myogel.3D
UT.SCC.40_Control
UT.SCC.44_Control
UT.SCC.14_Control
UT.SCC.14_Myogel.3D
UT.SCC.14_Myogel.2D
UT.SCC.44_Myogel.2D
UT.SCC.42A_Myogel.2D
UT.SCC.73_Myogel.2D
UT.SCC.40_Myogel.3D
UT.SCC.42B_Matrigel.3D
UT.SCC.42A_Myogel.3D
UT.SCC.81_Matrigel.3D
UT.SCC.73_Control
UT.SCC.28_Myogel.3D
UT.SCC.42A_Matrigel.3D
UT.SCC.14_Matrigel.2D
UT.SCC.42A_Matrigel.2D
UT.SCC.73_Matrigel.2D
UT.SCC.81_Control
UT.SCC.40_Matrigel.2D
UT.SCC.44_Matrigel.2D
UT.SCC.8_Myogel.2D
UT.SCC.44_Matrigel.3D
UT.SCC.42A_Control
UT.SCC.106A_Control
UT.SCC.73_Matrigel.3D
UT.SCC.28_Myogel.2D
UT.SCC.8_Matrigel.2D
UT.SCC.81_Matrigel.2D
UT.SCC.81_Myogel.2D
UT.SCC.8_Control
UT.SCC.40_Matrigel.3D
UT.SCC.24B_Myogel.2D
UT.SCC.8_Matrigel.3D
UT.SCC.14_Matrigel.3D
UT.SCC.81_Myogel.3D
UT.SCC.24A_Myogel.2D
UT.SCC.28_Matrigel.3D
UT.SCC.28_Control
UT.SCC.106A_Matrigel.2D
UT.SCC.24B_Matrigel.2D
UT.SCC.28_Matrigel.2D
UT.SCC.24B_Myogel.3D
UT.SCC.24B_Control
UT.SCC.106A_Matrigel.3D
UT.SCC.24A_Matrigel.3D
UT.SCC.24A_Matrigel.2D
UT.SCC.24A_Myogel.3D
UT.SCC.24A_Control
UT.SCC.24B_Matrigel.3D

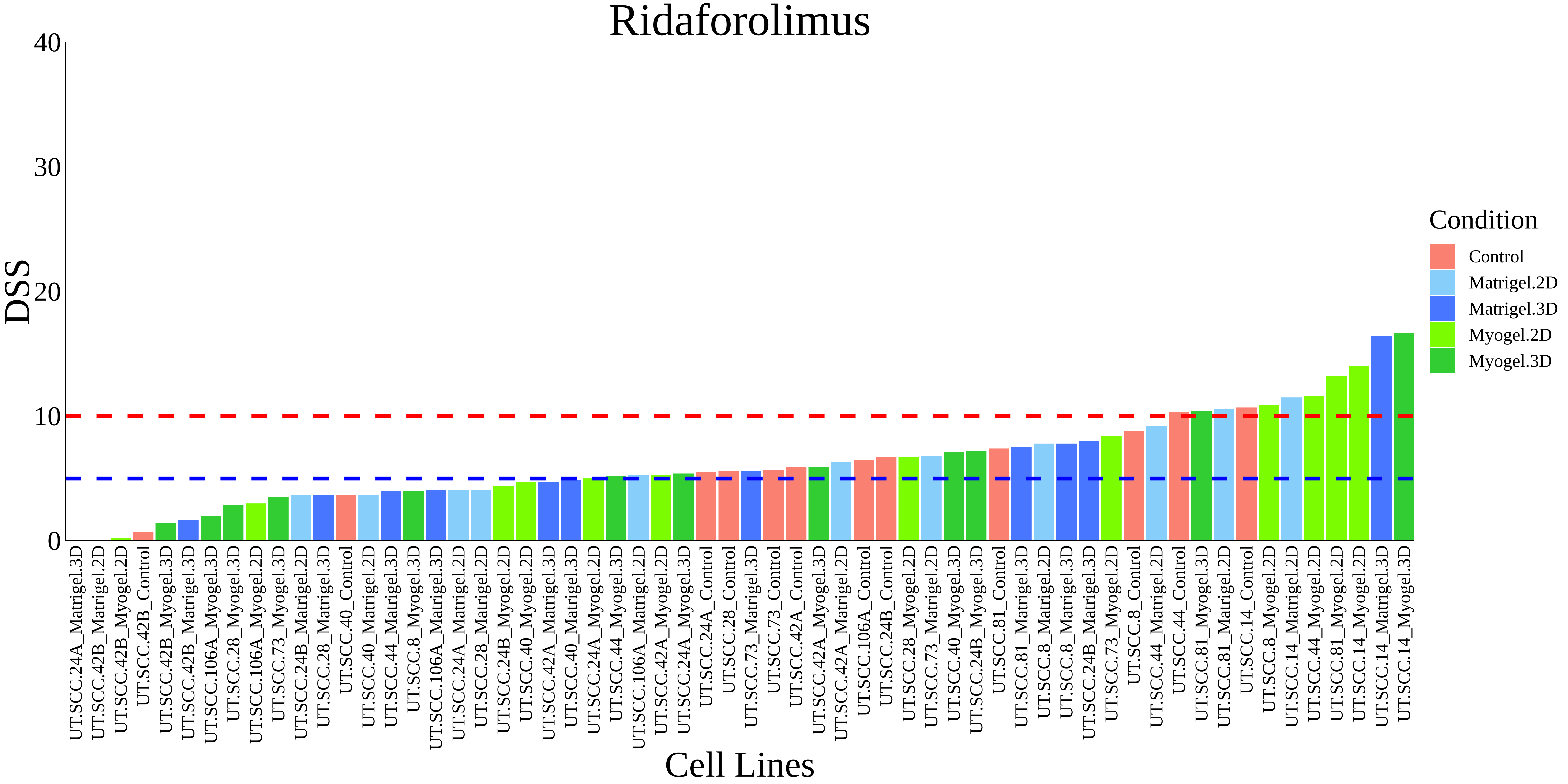

Ridaforolimus
DSS
0
10
20
30
40
UT.SCC.24A_Matrigel.3D
UT.SCC.42B_Matrigel.2D
UT.SCC.42B_Myogel.2D
UT.SCC.42B_Control
UT.SCC.42B_Myogel.3D
UT.SCC.42B_Matrigel.3D
UT.SCC.106A_Myogel.3D
UT.SCC.28_Myogel.3D
UT.SCC.106A_Myogel.2D
UT.SCC.73_Myogel.3D
UT.SCC.24B_Matrigel.2D
UT.SCC.28_Matrigel.3D
UT.SCC.40_Control
UT.SCC.40_Matrigel.2D
UT.SCC.44_Matrigel.3D
UT.SCC.8_Myogel.3D
UT.SCC.106A_Matrigel.3D
UT.SCC.24A_Matrigel.2D
UT.SCC.28_Matrigel.2D
UT.SCC.24B_Myogel.2D
UT.SCC.40_Myogel.2D
UT.SCC.42A_Matrigel.3D
UT.SCC.40_Matrigel.3D
UT.SCC.24A_Myogel.2D
UT.SCC.44_Myogel.3D
UT.SCC.106A_Matrigel.2D
UT.SCC.42A_Myogel.2D
UT.SCC.24A_Myogel.3D
UT.SCC.24A_Control
UT.SCC.28_Control
UT.SCC.73_Matrigel.3D
UT.SCC.73_Control
UT.SCC.42A_Control
UT.SCC.42A_Myogel.3D
UT.SCC.42A_Matrigel.2D
UT.SCC.106A_Control
UT.SCC.24B_Control
UT.SCC.28_Myogel.2D
UT.SCC.73_Matrigel.2D
UT.SCC.40_Myogel.3D
UT.SCC.24B_Myogel.3D
UT.SCC.81_Control
UT.SCC.81_Matrigel.3D
UT.SCC.8_Matrigel.2D
UT.SCC.8_Matrigel.3D
UT.SCC.24B_Matrigel.3D
UT.SCC.73_Myogel.2D
UT.SCC.8_Control
UT.SCC.44_Matrigel.2D
UT.SCC.44_Control
UT.SCC.81_Myogel.3D
UT.SCC.81_Matrigel.2D
UT.SCC.14_Control
UT.SCC.8_Myogel.2D
UT.SCC.14_Matrigel.2D
UT.SCC.44_Myogel.2D
UT.SCC.81_Myogel.2D
UT.SCC.14_Myogel.2D
UT.SCC.14_Matrigel.3D
UT.SCC.14_Myogel.3D
Cell Lines
Condition
Control
Matrigel.2D
Matrigel.3D
Myogel.2D
Myogel.3D

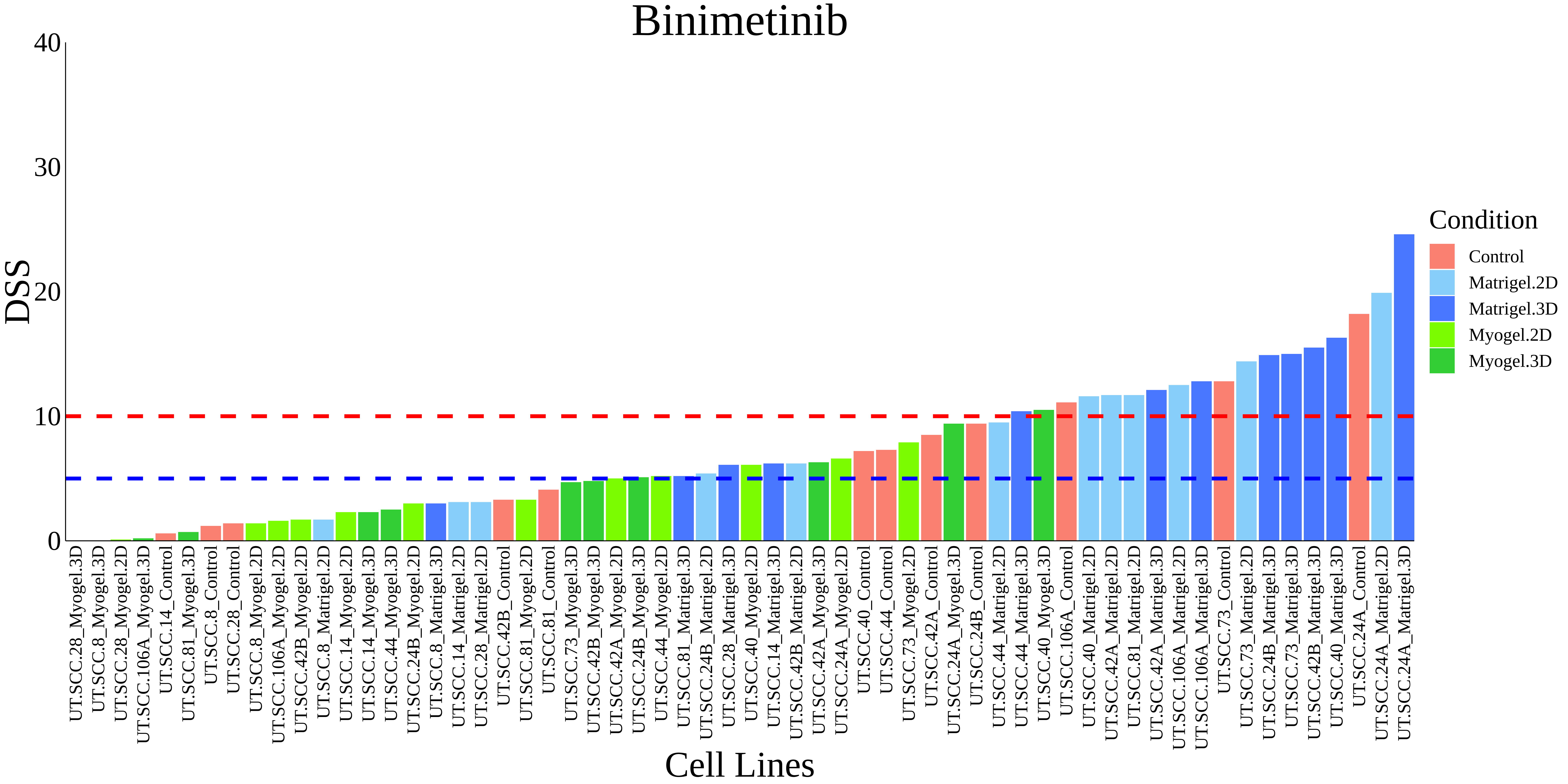

Binimetinib
DSS
Cell Lines
Condition
Control
Matrigel.2D
Matrigel.3D
Myogel.2D
Myogel.3D
0
10
20
30
40
UT.SCC.28_Myogel.3D
UT.SCC.8_Myogel.3D
UT.SCC.28_Myogel.2D
UT.SCC.106A_Myogel.3D
UT.SCC.14_Control
UT.SCC.81_Myogel.3D
UT.SCC.8_Control
UT.SCC.28_Control
UT.SCC.8_Myogel.2D
UT.SCC.106A_Myogel.2D
UT.SCC.42B_Myogel.2D
UT.SCC.8_Matrigel.2D
UT.SCC.14_Myogel.2D
UT.SCC.14_Myogel.3D
UT.SCC.44_Myogel.3D
UT.SCC.24B_Myogel.2D
UT.SCC.8_Matrigel.3D
UT.SCC.14_Matrigel.2D
UT.SCC.28_Matrigel.2D
UT.SCC.42B_Control
UT.SCC.81_Myogel.2D
UT.SCC.81_Control
UT.SCC.73_Myogel.3D
UT.SCC.42B_Myogel.3D
UT.SCC.42A_Myogel.2D
UT.SCC.24B_Myogel.3D
UT.SCC.44_Myogel.2D
UT.SCC.81_Matrigel.3D
UT.SCC.24B_Matrigel.2D
UT.SCC.28_Matrigel.3D
UT.SCC.40_Myogel.2D
UT.SCC.14_Matrigel.3D
UT.SCC.42B_Matrigel.2D
UT.SCC.42A_Myogel.3D
UT.SCC.24A_Myogel.2D
UT.SCC.40_Control
UT.SCC.44_Control
UT.SCC.73_Myogel.2D
UT.SCC.42A_Control
UT.SCC.24A_Myogel.3D
UT.SCC.24B_Control
UT.SCC.44_Matrigel.2D
UT.SCC.44_Matrigel.3D
UT.SCC.40_Myogel.3D
UT.SCC.106A_Control
UT.SCC.40_Matrigel.2D
UT.SCC.42A_Matrigel.2D
UT.SCC.81_Matrigel.2D
UT.SCC.42A_Matrigel.3D
UT.SCC.106A_Matrigel.2D
UT.SCC.106A_Matrigel.3D
UT.SCC.73_Control
UT.SCC.73_Matrigel.2D
UT.SCC.24B_Matrigel.3D
UT.SCC.73_Matrigel.3D
UT.SCC.42B_Matrigel.3D
UT.SCC.40_Matrigel.3D
UT.SCC.24A_Control
UT.SCC.24A_Matrigel.2D
UT.SCC.24A_Matrigel.3D

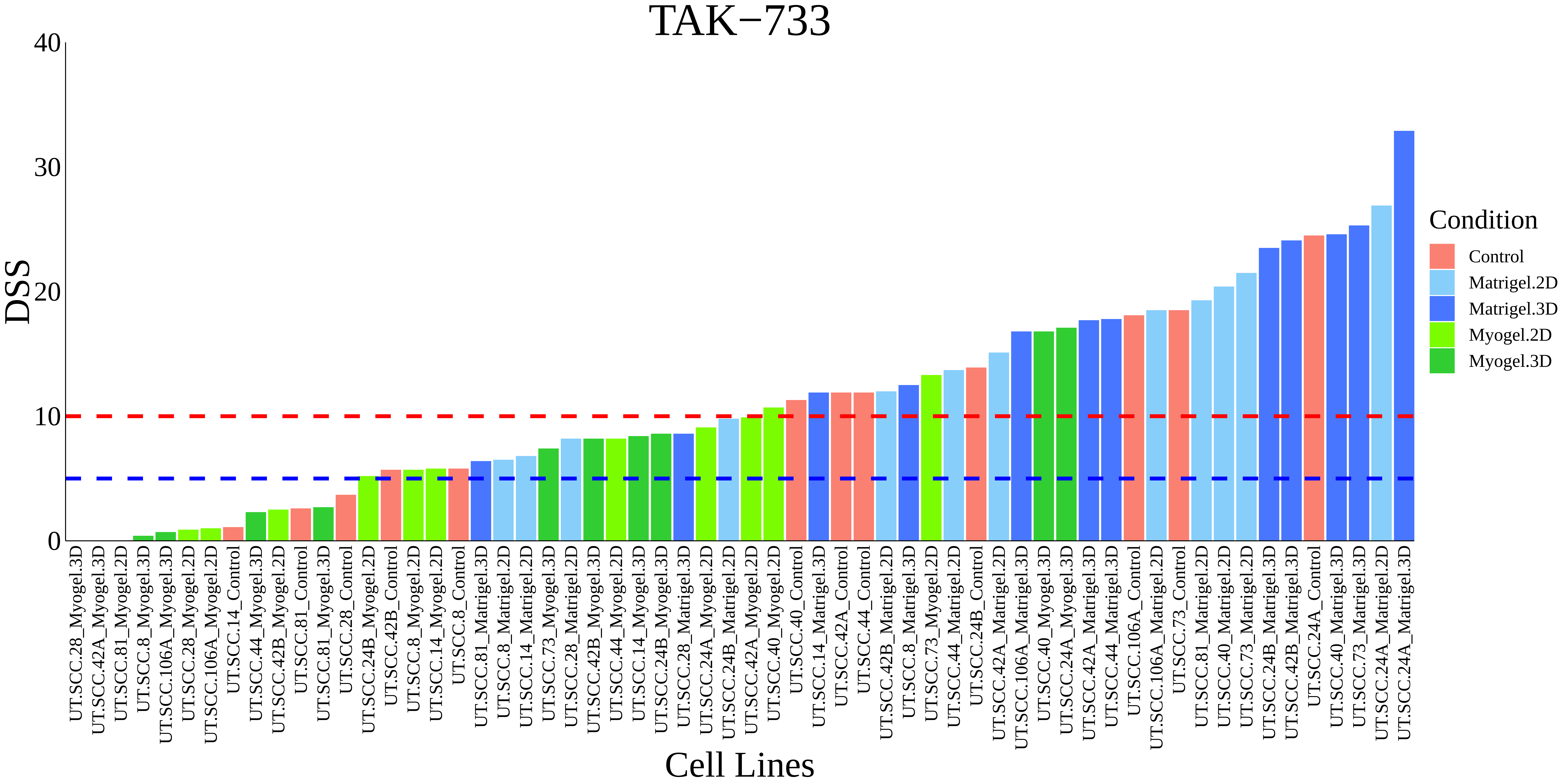
TAK−733
DSS
40
30
20
10
0
Cell Lines
Condition
Control
Matrigel.2D
Matrigel.3D
Myogel.2D
Myogel.3D
UT.SCC.28_Myogel.3D
UT.SCC.42A_Myogel.3D
UT.SCC.81_Myogel.2D
UT.SCC.8_Myogel.3D
UT.SCC.106A_Myogel.3D
UT.SCC.28_Myogel.2D
UT.SCC.106A_Myogel.2D
UT.SCC.14_Control
UT.SCC.44_Myogel.3D
UT.SCC.42B_Myogel.2D
UT.SCC.81_Control
UT.SCC.81_Myogel.3D
UT.SCC.28_Control
UT.SCC.24B_Myogel.2D
UT.SCC.42B_Control
UT.SCC.8_Myogel.2D
UT.SCC.14_Myogel.2D
UT.SCC.8_Control
UT.SCC.81_Matrigel.3D
UT.SCC.8_Matrigel.2D
UT.SCC.14_Matrigel.2D
UT.SCC.73_Myogel.3D
UT.SCC.28_Matrigel.2D
UT.SCC.42B_Myogel.3D
UT.SCC.44_Myogel.2D
UT.SCC.14_Myogel.3D
UT.SCC.24B_Myogel.3D
UT.SCC.28_Matrigel.3D
UT.SCC.24A_Myogel.2D
UT.SCC.24B_Matrigel.2D
UT.SCC.42A_Myogel.2D
UT.SCC.40_Myogel.2D
UT.SCC.40_Control
UT.SCC.14_Matrigel.3D
UT.SCC.42A_Control
UT.SCC.44_Control
UT.SCC.42B_Matrigel.2D
UT.SCC.8_Matrigel.3D
UT.SCC.73_Myogel.2D
UT.SCC.44_Matrigel.2D
UT.SCC.24B_Control
UT.SCC.42A_Matrigel.2D
UT.SCC.106A_Matrigel.3D
UT.SCC.40_Myogel.3D
UT.SCC.24A_Myogel.3D
UT.SCC.42A_Matrigel.3D
UT.SCC.44_Matrigel.3D
UT.SCC.106A_Control
UT.SCC.106A_Matrigel.2D
UT.SCC.73_Control
UT.SCC.81_Matrigel.2D
UT.SCC.40_Matrigel.2D
UT.SCC.73_Matrigel.2D
UT.SCC.24B_Matrigel.3D
UT.SCC.42B_Matrigel.3D
UT.SCC.24A_Control
UT.SCC.40_Matrigel.3D
UT.SCC.73_Matrigel.3D
UT.SCC.24A_Matrigel.2D
UT.SCC.24A_Matrigel.3D

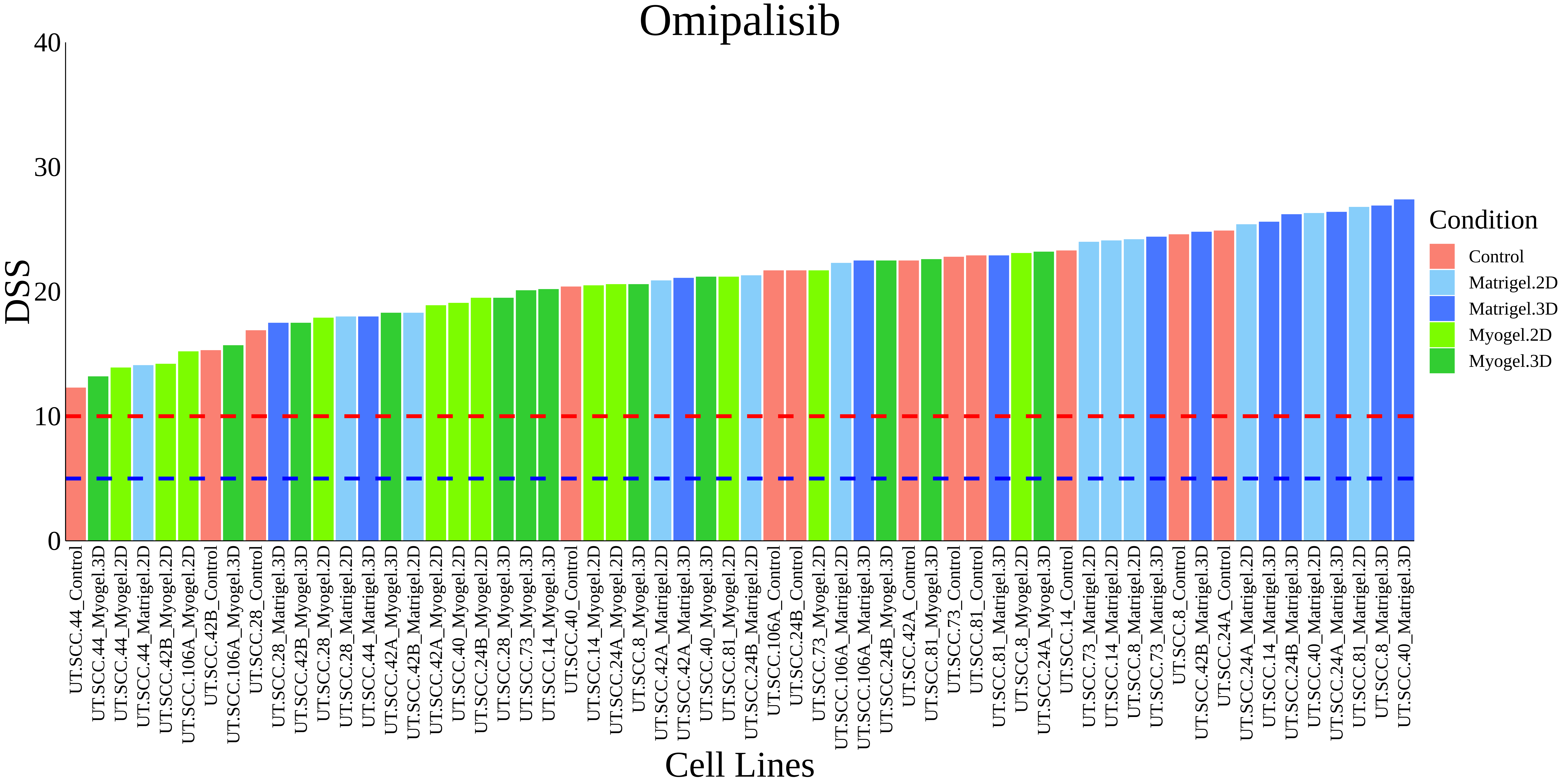
Omipalisib
DSS
Cell Lines
Condition
Control
Matrigel.2D
Matrigel.3D
Myogel.2D
Myogel.3D
0
10
20
30
40
UT.SCC.44_Control
UT.SCC.44_Myogel.3D
UT.SCC.44_Myogel.2D
UT.SCC.44_Matrigel.2D
UT.SCC.42B_Myogel.2D
UT.SCC.106A_Myogel.2D
UT.SCC.42B_Control
UT.SCC.106A_Myogel.3D
UT.SCC.28_Control
UT.SCC.28_Matrigel.3D
UT.SCC.42B_Myogel.3D
UT.SCC.28_Myogel.2D
UT.SCC.28_Matrigel.2D
UT.SCC.44_Matrigel.3D
UT.SCC.42A_Myogel.3D
UT.SCC.42B_Matrigel.2D
UT.SCC.42A_Myogel.2D
UT.SCC.40_Myogel.2D
UT.SCC.24B_Myogel.2D
UT.SCC.28_Myogel.3D
UT.SCC.73_Myogel.3D
UT.SCC.14_Myogel.3D
UT.SCC.40_Control
UT.SCC.14_Myogel.2D
UT.SCC.24A_Myogel.2D
UT.SCC.8_Myogel.3D
UT.SCC.42A_Matrigel.2D
UT.SCC.42A_Matrigel.3D
UT.SCC.40_Myogel.3D
UT.SCC.81_Myogel.2D
UT.SCC.24B_Matrigel.2D
UT.SCC.106A_Control
UT.SCC.24B_Control
UT.SCC.73_Myogel.2D
UT.SCC.106A_Matrigel.2D
UT.SCC.106A_Matrigel.3D
UT.SCC.24B_Myogel.3D
UT.SCC.42A_Control
UT.SCC.81_Myogel.3D
UT.SCC.73_Control
UT.SCC.81_Control
UT.SCC.81_Matrigel.3D
UT.SCC.8_Myogel.2D
UT.SCC.24A_Myogel.3D
UT.SCC.14_Control
UT.SCC.73_Matrigel.2D
UT.SCC.14_Matrigel.2D
UT.SCC.8_Matrigel.2D
UT.SCC.73_Matrigel.3D
UT.SCC.8_Control
UT.SCC.42B_Matrigel.3D
UT.SCC.24A_Control
UT.SCC.24A_Matrigel.2D
UT.SCC.14_Matrigel.3D
UT.SCC.24B_Matrigel.3D
UT.SCC.40_Matrigel.2D
UT.SCC.24A_Matrigel.3D
UT.SCC.81_Matrigel.2D
UT.SCC.8_Matrigel.3D
UT.SCC.40_Matrigel.3D
