# Supplementary material for: Human Tumor–Derived Matrix Improves the Predictability of Head and Neck Cancer Drug Testing
Source: Cancers (Basel). 2019 Dec 30;12(1):92. doi: 10.3390/cancers12010092 (PMC7017272; doi:10.3390/cancers12010092)

Supplementary Figure 3: Cells were cultured in 2D plastic wells, on top or embedded in Matrigel or Myogel. Within Matrigel, UT-SCC-8 cells formed isolated round-shaped spheroids, whereas within Myogel cells were stellate-shaped.

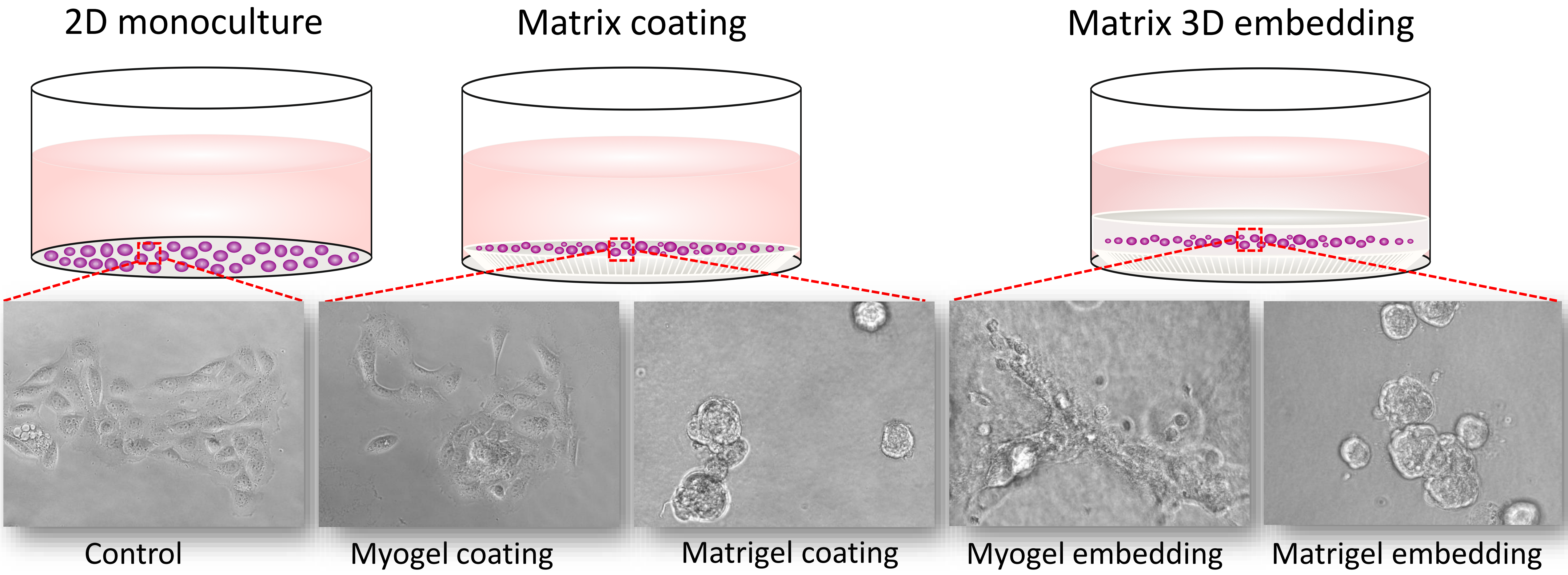

Supplement: Supplementary file 1 [file cancers-12-00092-s001.zip › cancers-664648-supplement-final/Supplementary Figure 3.pdf]
