# Supplementary material for: Human Tumor–Derived Matrix Improves the Predictability of Head and Neck Cancer Drug Testing
Source: Cancers (Basel). 2019 Dec 30;12(1):92. doi: 10.3390/cancers12010092 (PMC7017272; doi:10.3390/cancers12010092)
Supplement: Supplementary file 1 [file cancers-12-00092-s001.zip › cancers-664648-supplement-final/Supplementary Figure 4.pdf]

Supplementary Figure 4. Cell density and morphology of two representarive cell lines, **UT-SCC-40** (**A**) and **UT-SCC81** (**B**), cultured using different matrices, after 72 hours of cetuximab and omipaliseb treatment. UT-SCC-40 cells responded to cetuximab treatment on Matrigel but showed resistance on Myogel and on Plastic (**A**). UT-SCC81 cells responded to cetuximab treatment on Matrigel and plastic but showed resisistance on Myogel (**B**). Both cell lines were highly responsive to Omipalisib in all conditions (**A,B**). Cell density images were in line with viability data (dose-response curves shown in Supplementary Figure 6). Images were taken by IncuCyte Live-Cell Imaging System using objective 4x. Luminescence readout representing number of viable cells in the control wells in different matrices (**C**).

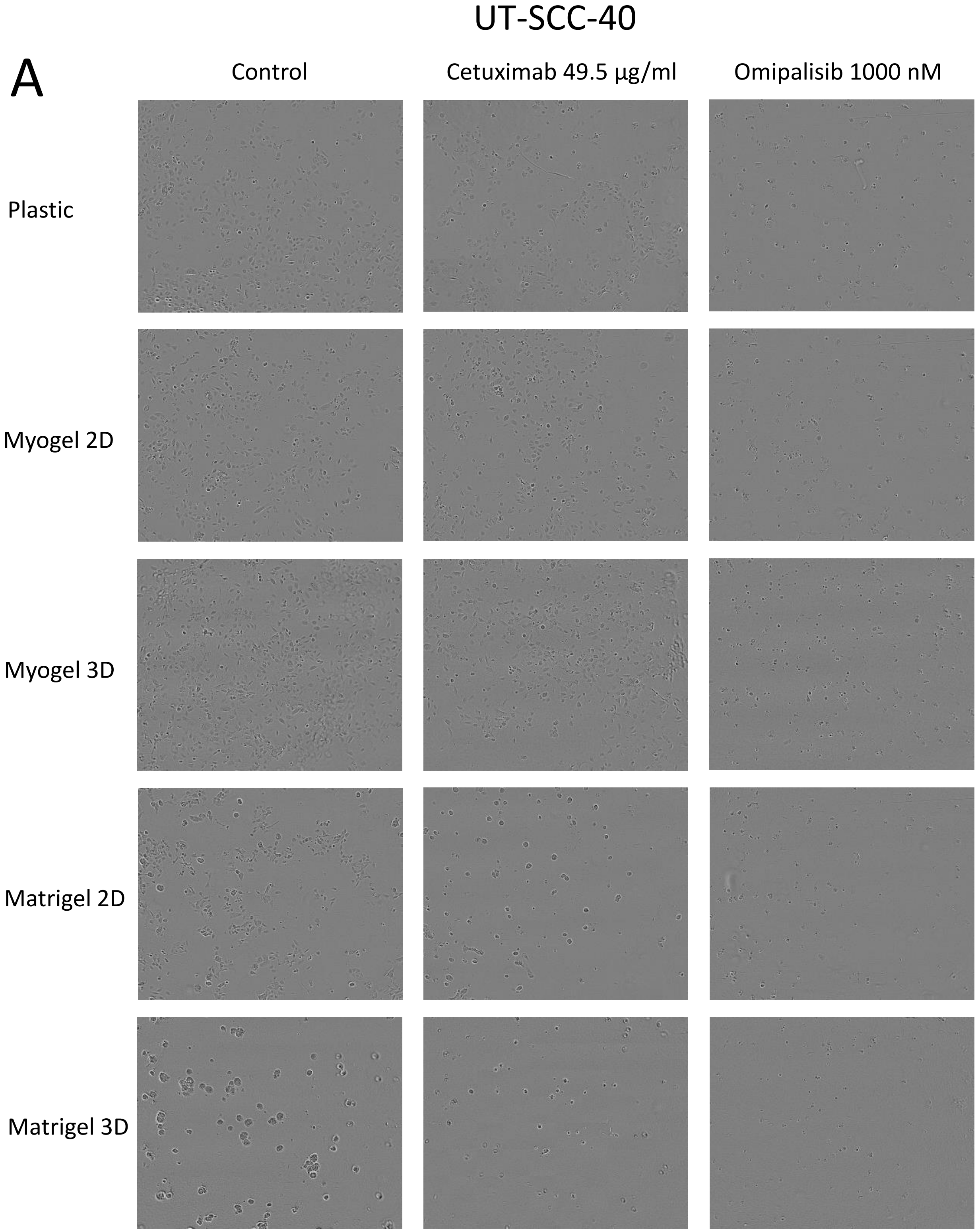

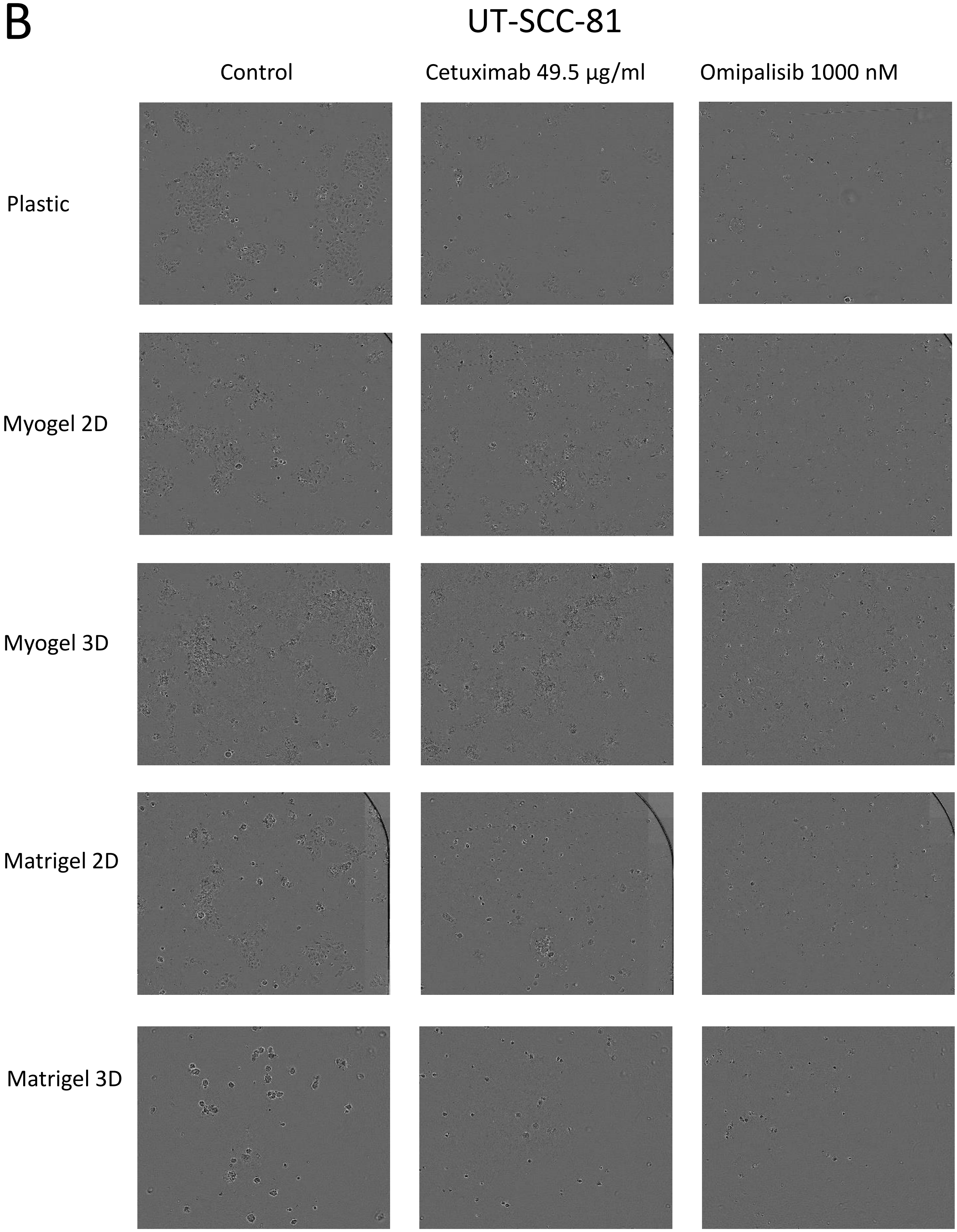
B
UT-SCC-81
Control
Cetuximab 49.5 µg/ml
Omipalisib 1000 nM
Plastic
Myogel 2D
Myogel 3D
Matrigel 2D
Matrigel 3D

C

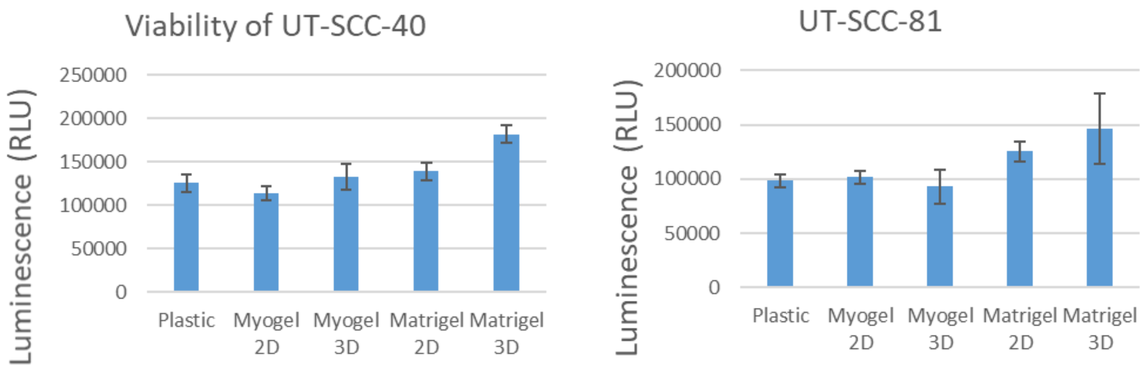
Viability of UT-SCC-40
Luminescence (RLU)
250000
200000
150000
100000
50000
0
Plastic
Myogel 2D
Myogel 3D
Matrigel 2D
Matrigel 3D
UT-SCC-81
Luminescence (RLU)
200000
150000
100000
50000
0
Plastic
Myogel 2D
Myogel 3D
Matrigel 2D
Matrigel 3D
