# Supplementary material for: Human Tumor–Derived Matrix Improves the Predictability of Head and Neck Cancer Drug Testing
Source: Cancers (Basel). 2019 Dec 30;12(1):92. doi: 10.3390/cancers12010092 (PMC7017272; doi:10.3390/cancers12010092)
Supplement: Supplementary file 1 [file cancers-12-00092-s001.zip › cancers-664648-supplement-final/Supplementary Figure 5.pdf]

Supplementary Figure 5. Immunoblot analysis of EGFR (**A**), p-ERK1/2 (**B**) and ERK1/2 (**C**) in HSNCC cell lines growing on plastic, Matrigel and Myogel. β-Actin was used as the control to normalize the quantities of the proteins. Immunoblot figures, densitometry readings and normalized values are presented in following pages.

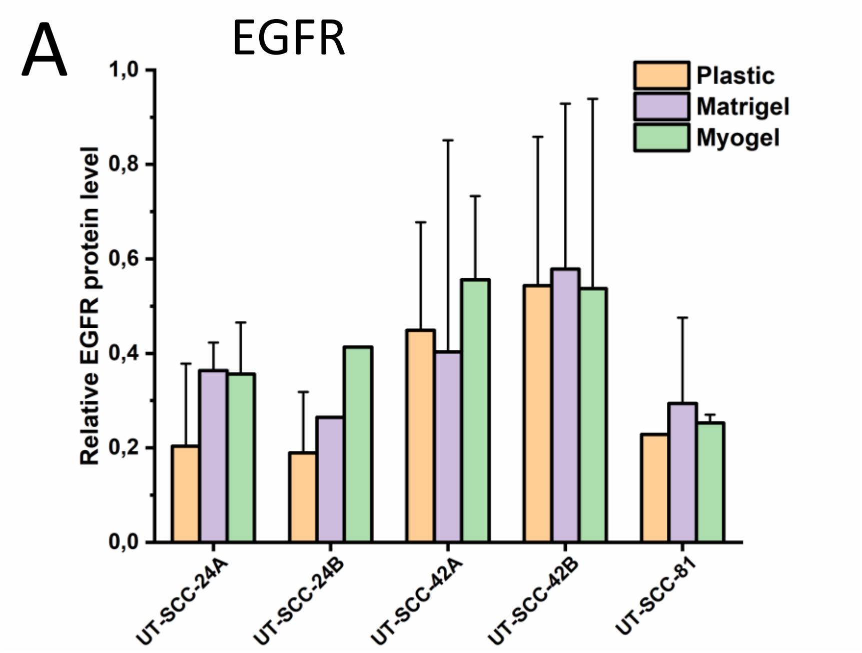

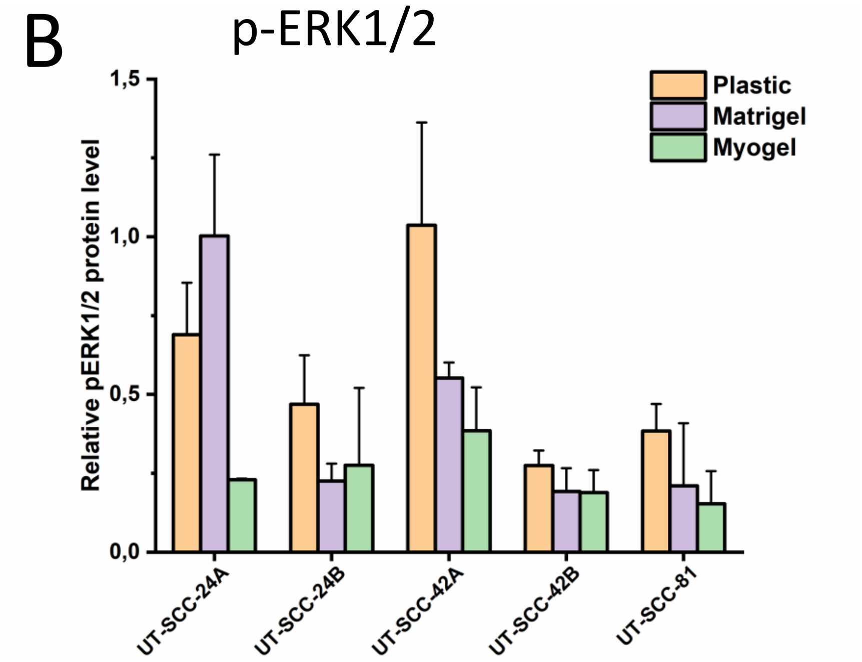

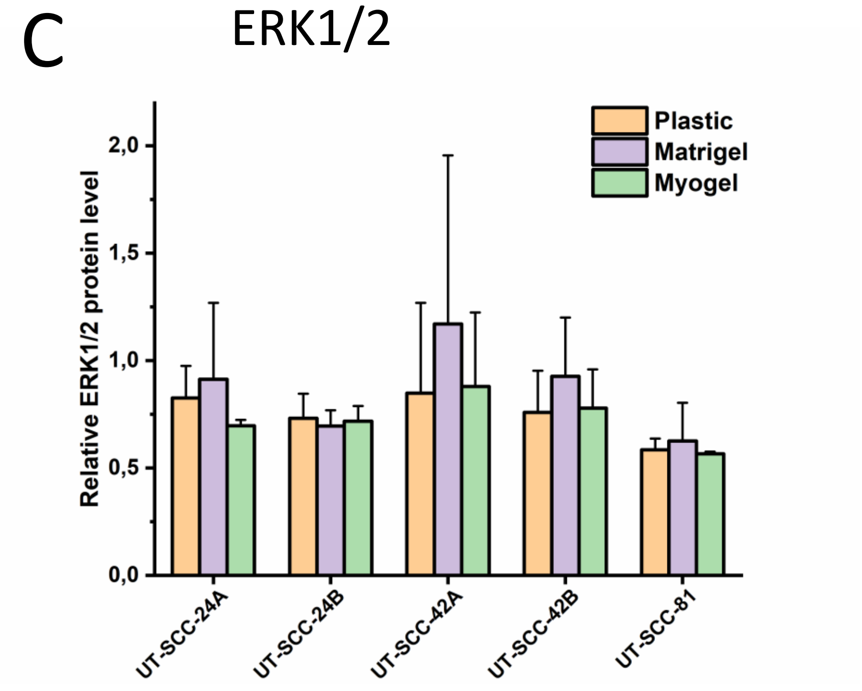

Immunoblot analysis of EGFR in UTSCC-24A, UTSCC-24B, UTSCC-42A, UTSCC-42B, and UTSCC-81 cell lines growing on plastic, Matrigel and Myogel. β-Actin was used as the control to normalize the quantities of the proteins. Immunoblot figures, densitometry readings and normalized values are presented.

UTSCC-24A

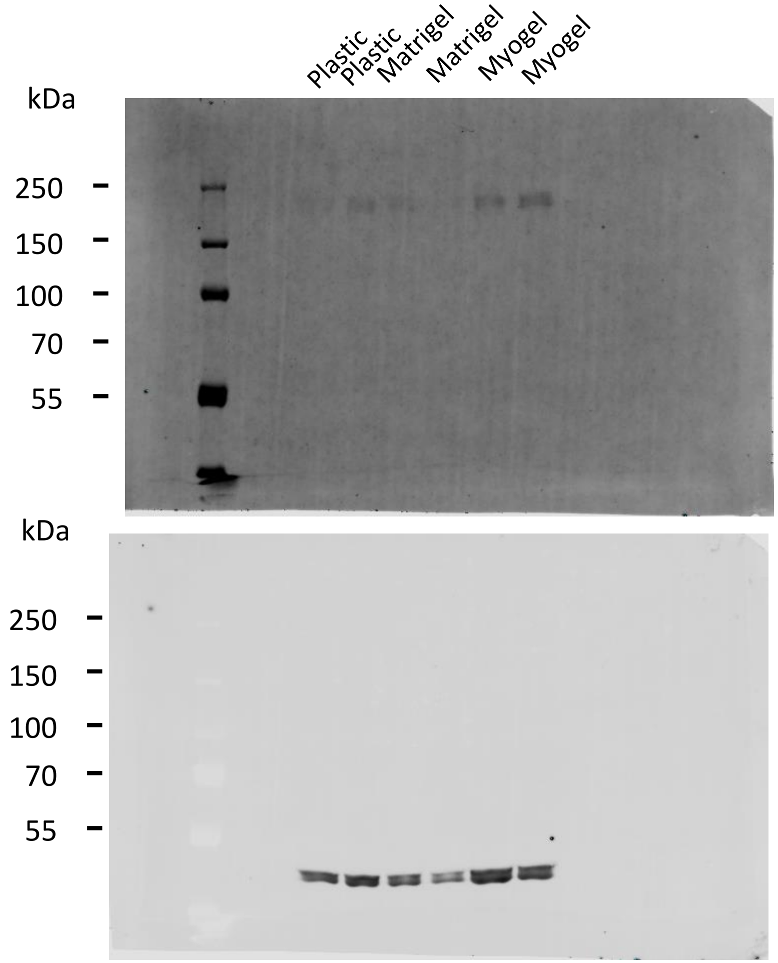

Experiment 1

	Densitometry readings					
	UTSCC-24A					
	plastic	plastic	matrigel	matrigel	myogel	myogel
EGFR	7901,7	4115,9	3884,9	2609,3	9010,6	10612,1
β-Actin	14039,1	16619,9	12628,6	7797,2	24937,2	17805,9
Normalized value	0,56	0,25	0,31	0,33	0,36	0,60

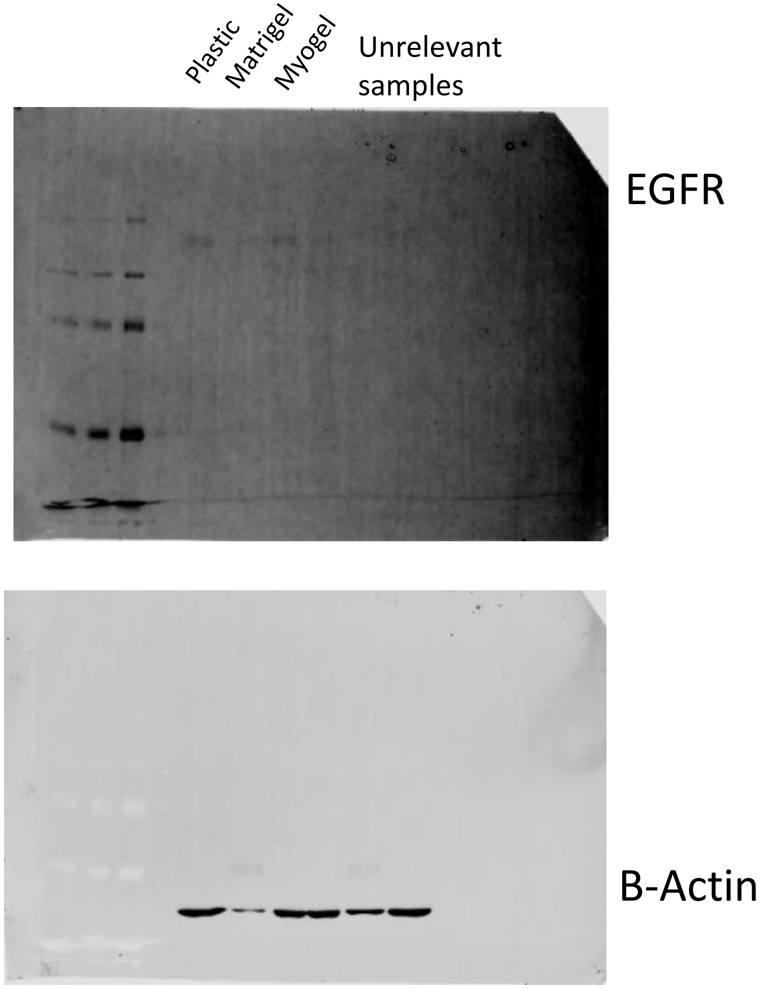

Experiment 2

	Densitometry readings		
	UTSCC-24A		
	plastic	matrigel	myogel
EGFR	2952,9	1571,9	4881,3
β-Actin	26193,4	3876,2	18177,1
Normalized value	0,11	0,41	0,27

UTSCC-24B

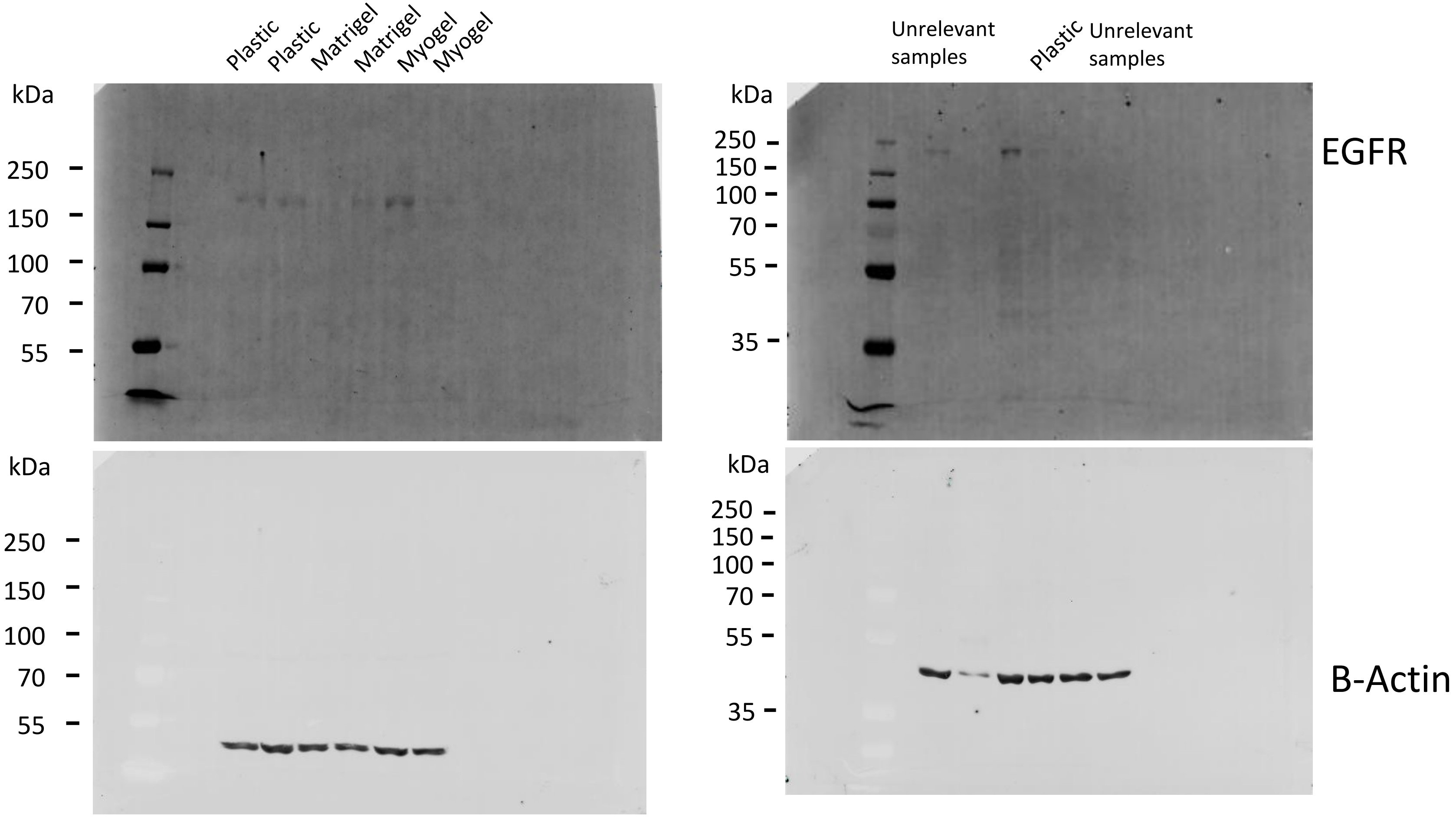

Experiment 1

Experiment 2

	Densitometry readings					
	UTSCC-24B					
	plastic	plastic	matrigel	matrigel	myogel	myogel
EGFR	4880,0	5538,9	3134,6	4759,9	11523,6	2830,2
β-Actin	13297,4	17976,5	15098,8	14803,3	17984,3	15271,2
Normalized value	0,37	0,31	0,21	0,32	0,64	0,19

	Densitometry readings		
	UTSCC-24B		
	Plastic		
EGFR	2246,4		
β-Actin	15543,5		
Normalized value	0,14		

UTSCC-42A

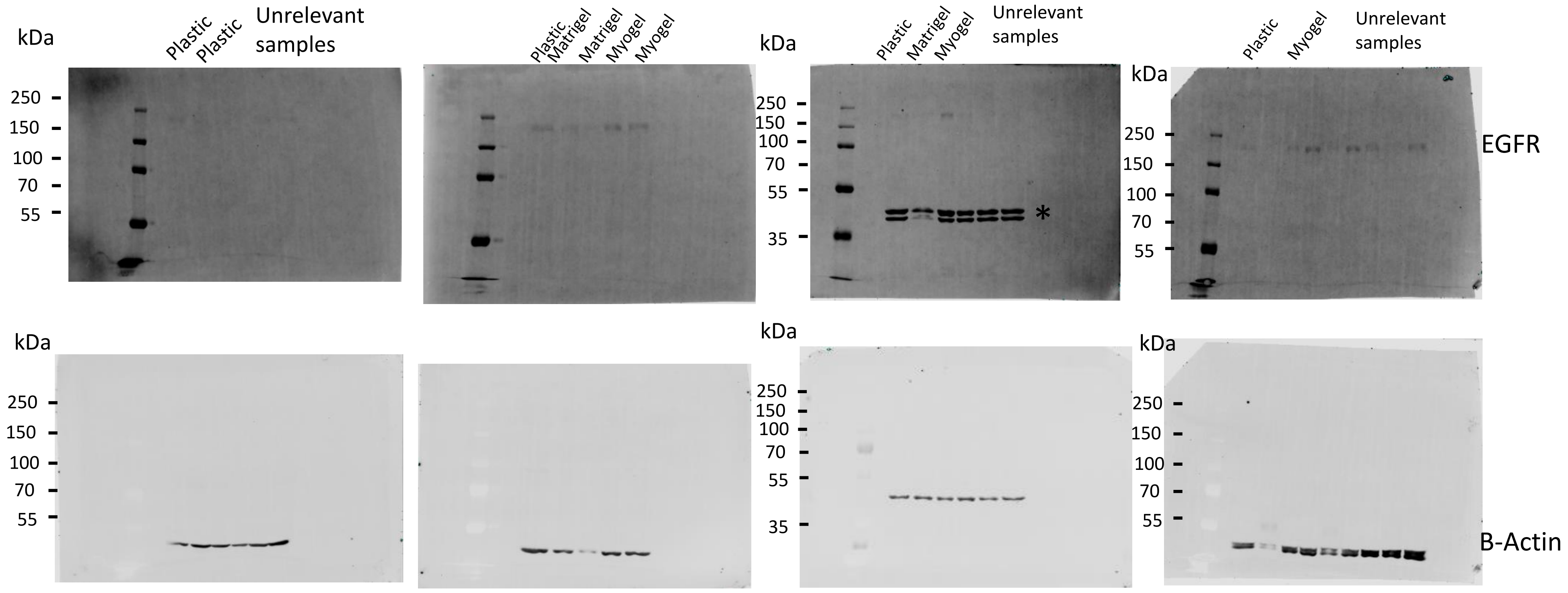

Experiment 1

	Densitometry readings	
	UTSCC-42A	
	plastic	plastic
EGFR	5753,2	8781,2
β-Actin	6906,4	9970,0
Normalized value	0,83	0,88

Experiment 1

	Densitometry readings				
	UTSCC-42A				
	plastic	matrigel	matrigel	myogel	myogel
EGFR	12792,1	8055,7	2805,9	15170,1	3088,9
β-Actin	30212,3	16259,6	14643,4	19744,1	21022,8
Normalized value	0,42	0,53	0,91	0,82	0,70

Experiment 2

	Densitometry readings		
	UTSCC-42A		
	plastic	matrigel	myogel
EGFR	4294,0	1307,4	5311,6
β-Actin	13839,6	15140,1	11709,6
Normalized value	0,31	0,09	0,45

Experiment 3

	Densitometry readings		
	UTSCC-42A		
	plastic		myogel
EGFR	2465,95		2973,539
β-Actin	7617,15		6539,91
Normalized value	0,32		0,45

* The membrane was first incubated with ERK1/2 antibody

UTSCC-42B

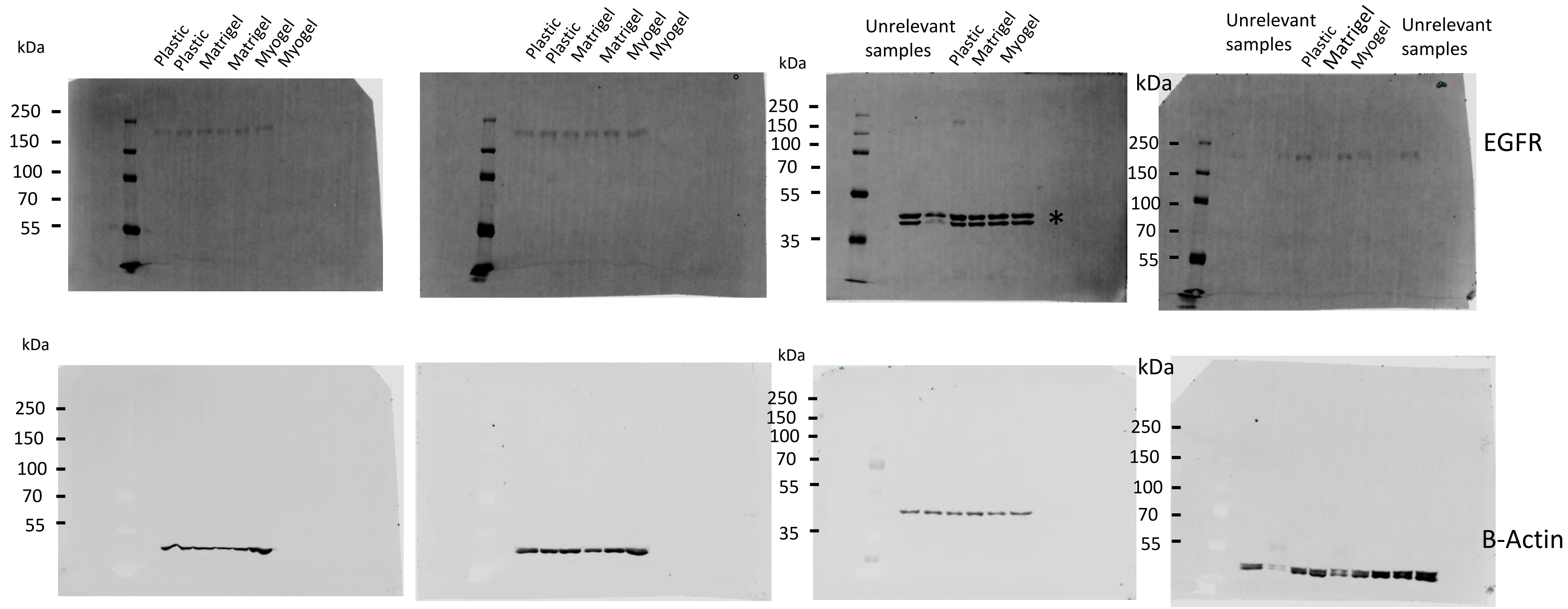

Experiment 1

	Densitometry readings					
	UTSCC-42B					
	plastic	plastic	matrigel	matrigel	myogel	myogel
EGFR	2961,0	9235,2	11553,0	4366,2	9185,6	10712,3
β-Actin	10961,8	8239,4	10030,3	5042,4	8132,2	25512,9
Normalized value	0,27	1,12	1,15	0,87	1,13	0,42

Experiment 1

	Densitometry readings					
	UTSCC-42B					
	plastic	plastic	matrigel	matrigel	myogel	myogel
EGFR	12405,2	10016,4	11559,3	9554,7	11107,1	14440,1
β-Actin	15943,0	8626,0	13414,2	9262,3	10031,2	15988,1
Normalized value	0,78	1,16	0,86	1,03	1,11	0,90

Experiment 2

	Densitometry readings		
	UTSCC-42B		
	plastic	matrigel	myogel
EGFR	3141,2	4814,3	1464,8
β-Actin	15174,6	11072,8	14592,6
Normalized value	0,21	0,43	0,10

Experiment 3

	Densitometry readings		
	UTSCC-42B		
	plastic	matrigel	myogel
EGFR	5044,8	1421,3	4766,3
β-Actin	8552,0	4407,7	7664,8
Normalized value	0,59	0,32	0,62

* The membrane was first incubated with ERK1/2 antibody

UTSCC-81

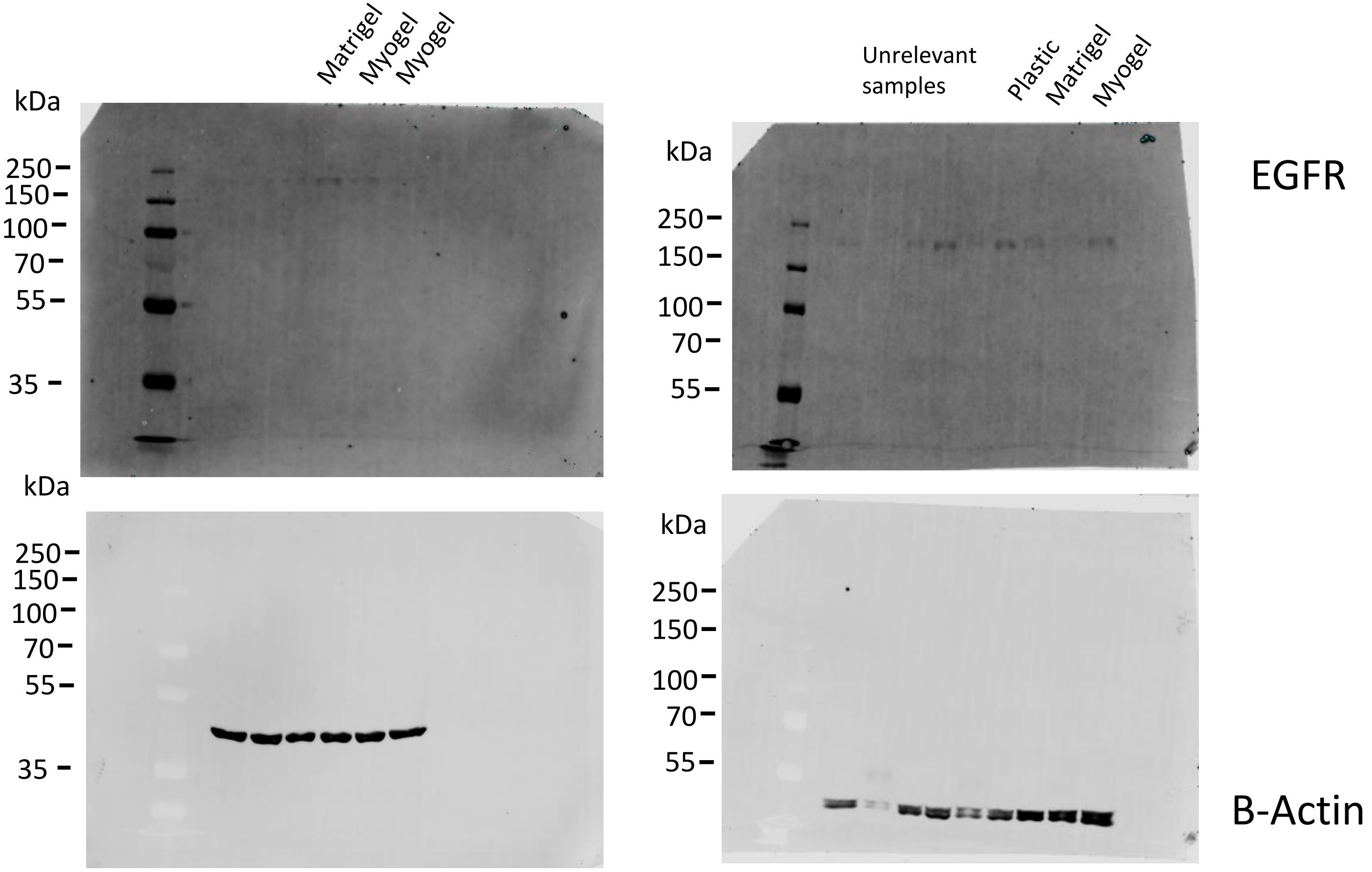

Experiment 1

	Densitometry readings		
	UTSCC-81		
	matrigel	myogel	myogel
EGFR	7394,6	4599,9	4595,4
β-Actin	17513,0	16317,9	18505,6
Normalized value	0,42	0,28	0,25

Experiment 2

	Densitometry readings		
	UTSCC-81		
	plastic	matrigel	myogel
EGFR	2368,4	2025,9	4157,4
β-Actin	10371,8	12237,2	17299,7
Normalized value	0,23	0,17	0,24

Immunoblot analysis of p-ERK1/2 in UTSCC-24A, UTSCC-24B, UTSCC-42A, UTSCC-42B, and UTSCC-81 cell lines growing on plastic, Matrigel and Myogel. β-Actin was used as the control to normalize the quantities of the proteins. Immunoblot figures, densitometry readings and normalized values are presented.

UTSCC-24A

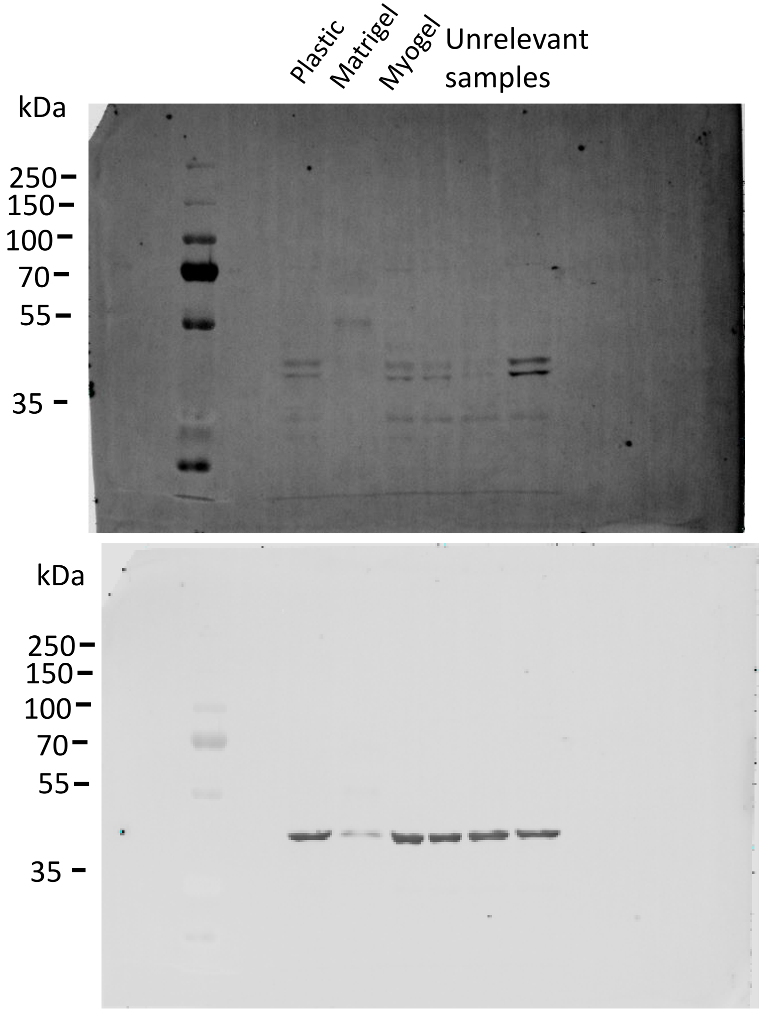

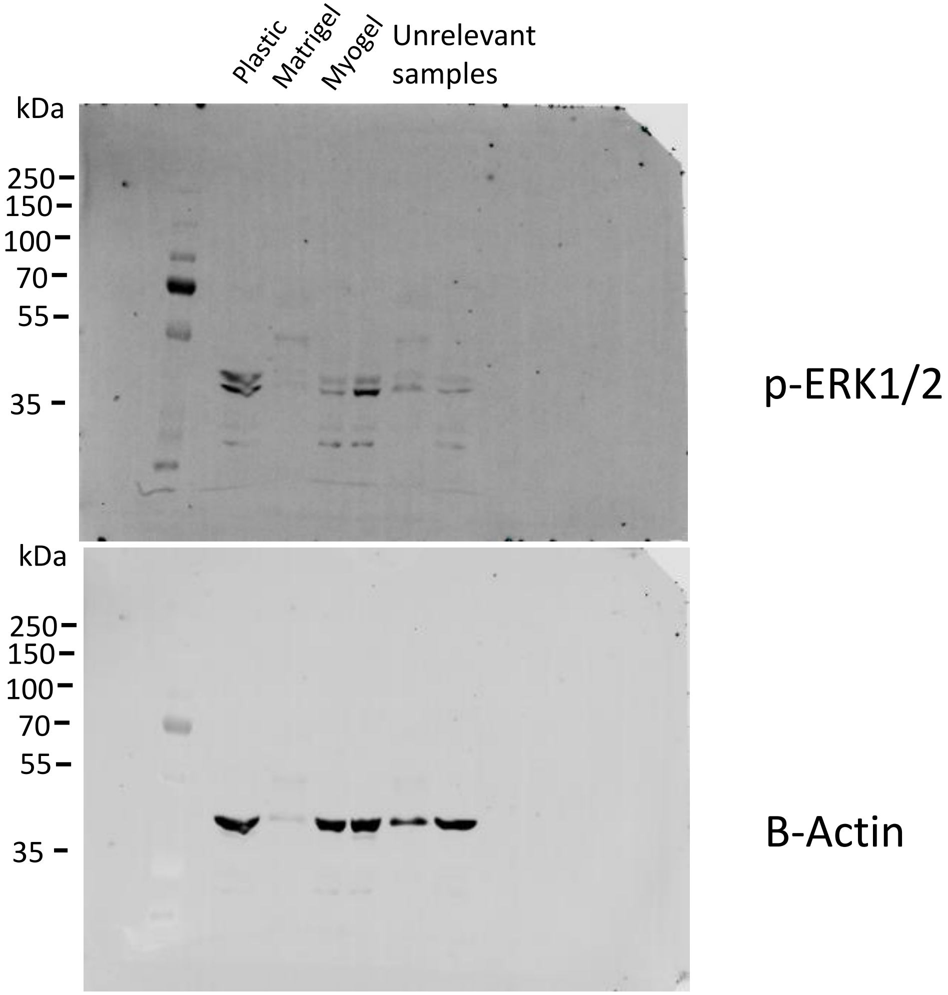

Experiment 1

	Densitometry readings		
	UTSCC-24A		
	plastic	matrigel	myogel
p-ERK1/2	6409	1417	3735
β-Actin	18906	3116	16894
Normalized value	0,34	0,45	0,22

Experiment 2

	Densitometry readings		
	UTSCC-24A		
	plastic	matrigel	myogel
p-ERK1/2	18646,8	612,4	3607,6
β-Actin	23136,2	516,9	15535,9
Normalized value	0,81	1,18	0,23

UTSCC-24B

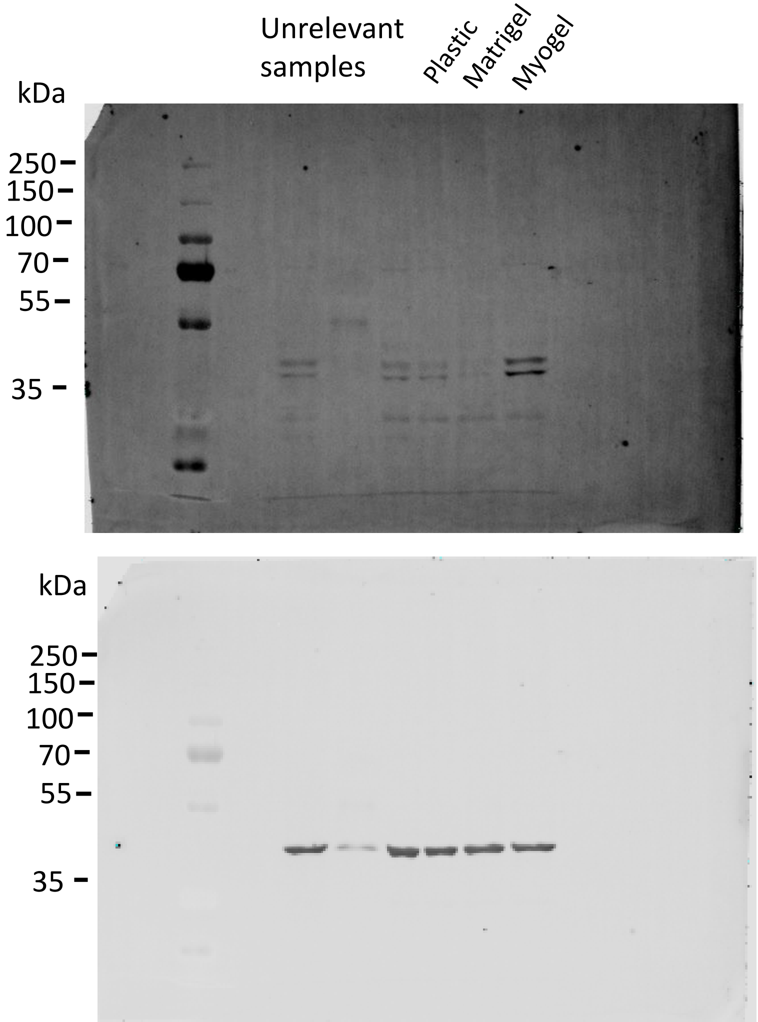

Experiment 1

	Densitometry readings		
	UTSCC-24B		
	plastic	matrigel	myogel
p-ERK1/2	2022,6	1868,1	13495,8
β-Actin	14438,8	17257,6	16974,2
Normalized value	0,14	0,11	0,80

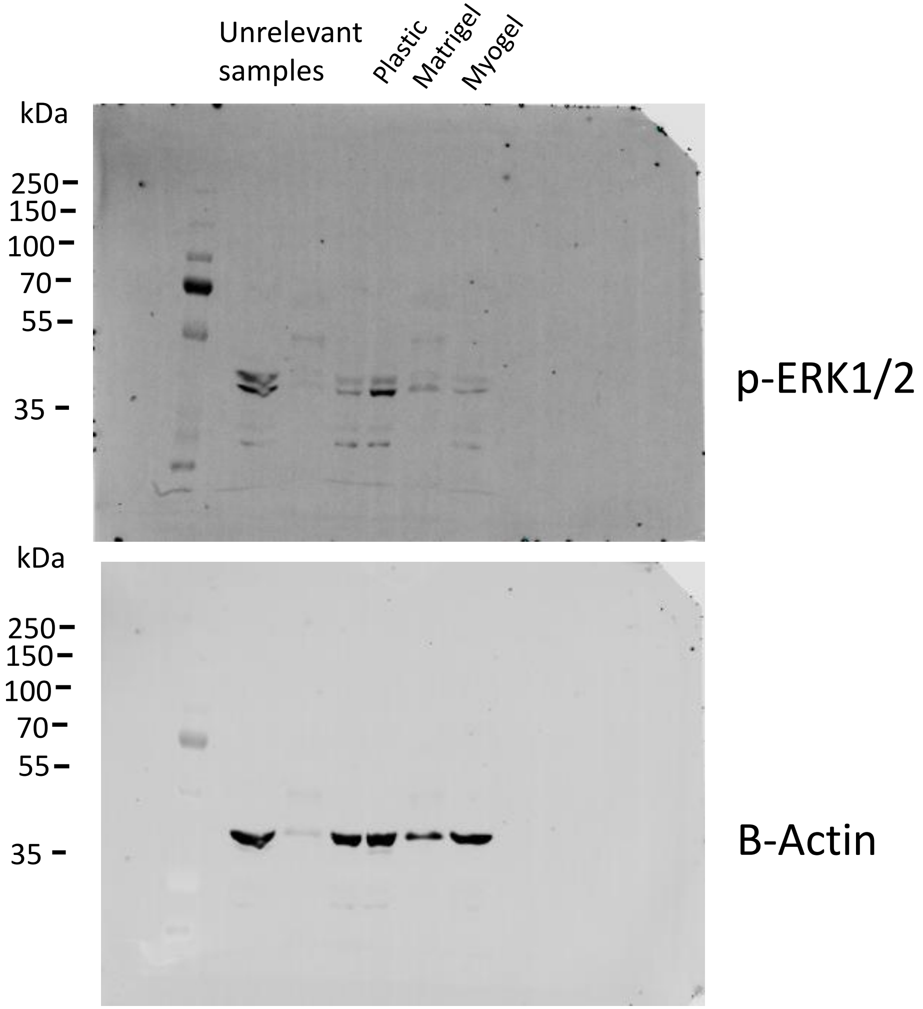

Experiment 2

	Densitometry readings		
	UTSCC-24B		
	plastic	matrigel	myogel
p-ERK1/2	9904,8	2486,9	1865,3
β-Actin	17109,6	9396,2	18146,1
Normalized value	0,58	0,26	0,10

UTSCC-42A

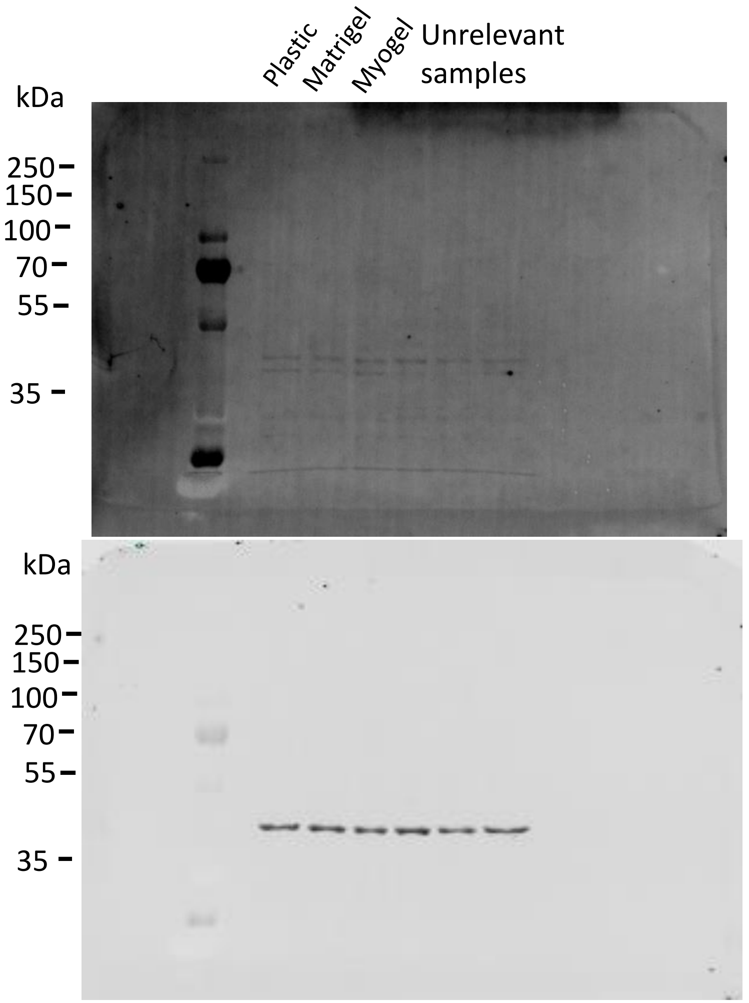

Experiment 1

	Densitometry readings		
	UTSCC-42A		
	plastic	matrigel	myogel
p-ERK1/2	4795,3	6788,0	7934,4
β-Actin	13839,6	15140,1	11709,6
Normalized value	0,35	0,45	0,68

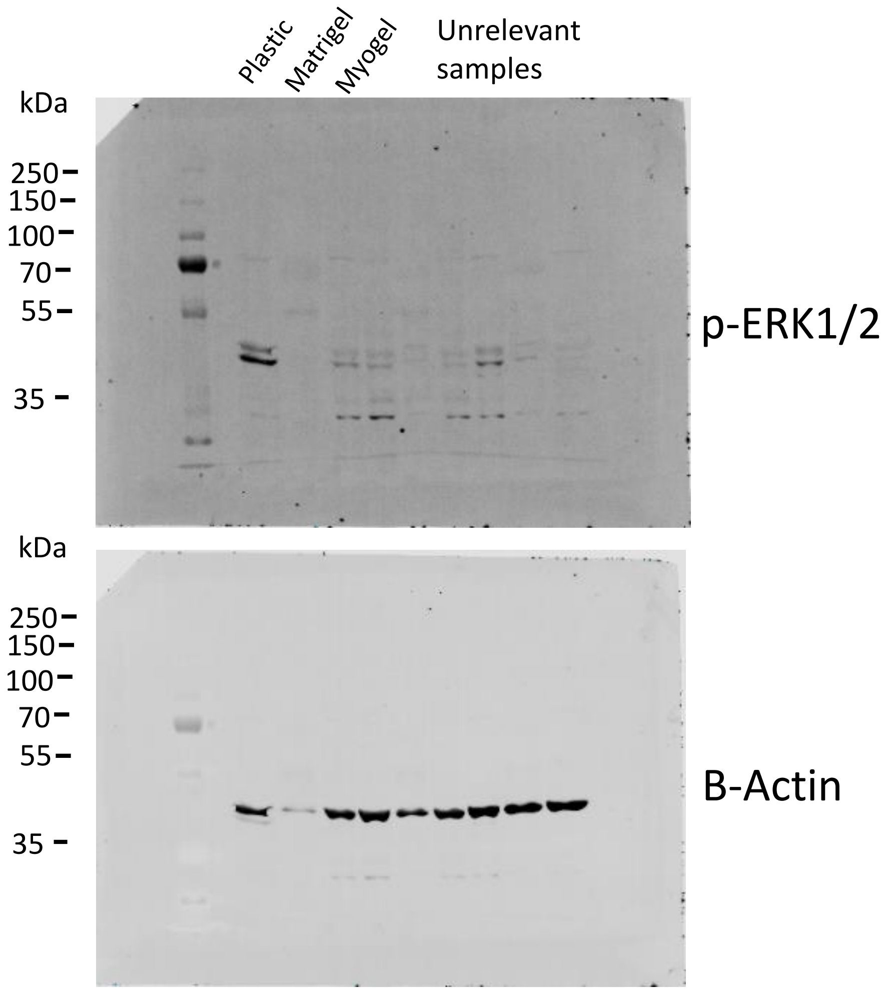

Experiment 2

	Densitometry readings		
	UTSCC-42A		
	plastic	matrigel	myogel
p-ERK1/2	13445,4	613,4	2757,7
β-Actin	10614,0	1045,2	9608,5
Normalized value	1,27	0,59	0,29

UTSCC-42B

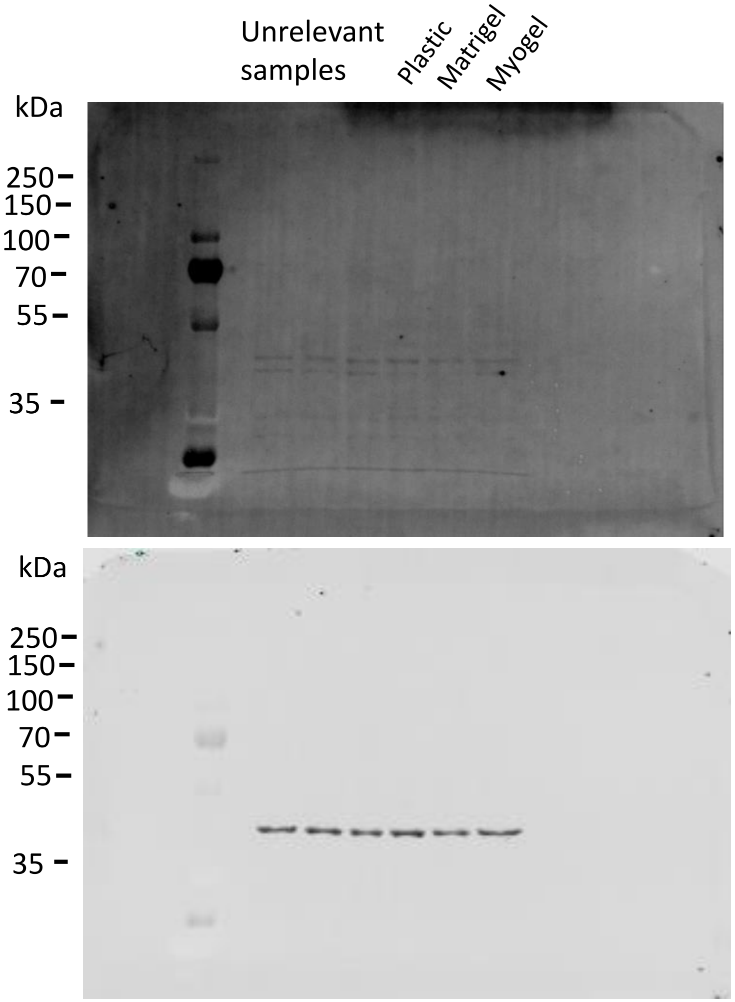

Experiment 1

	Densitometry readings		
	UTSCC-42B		
	plastic	matrigel	myogel
p-ERK1/2	5694,6	3857,9	4953,3
β-Actin	15174,6	11072,8	14592,6
Normalized value	0,38	0,35	0,34

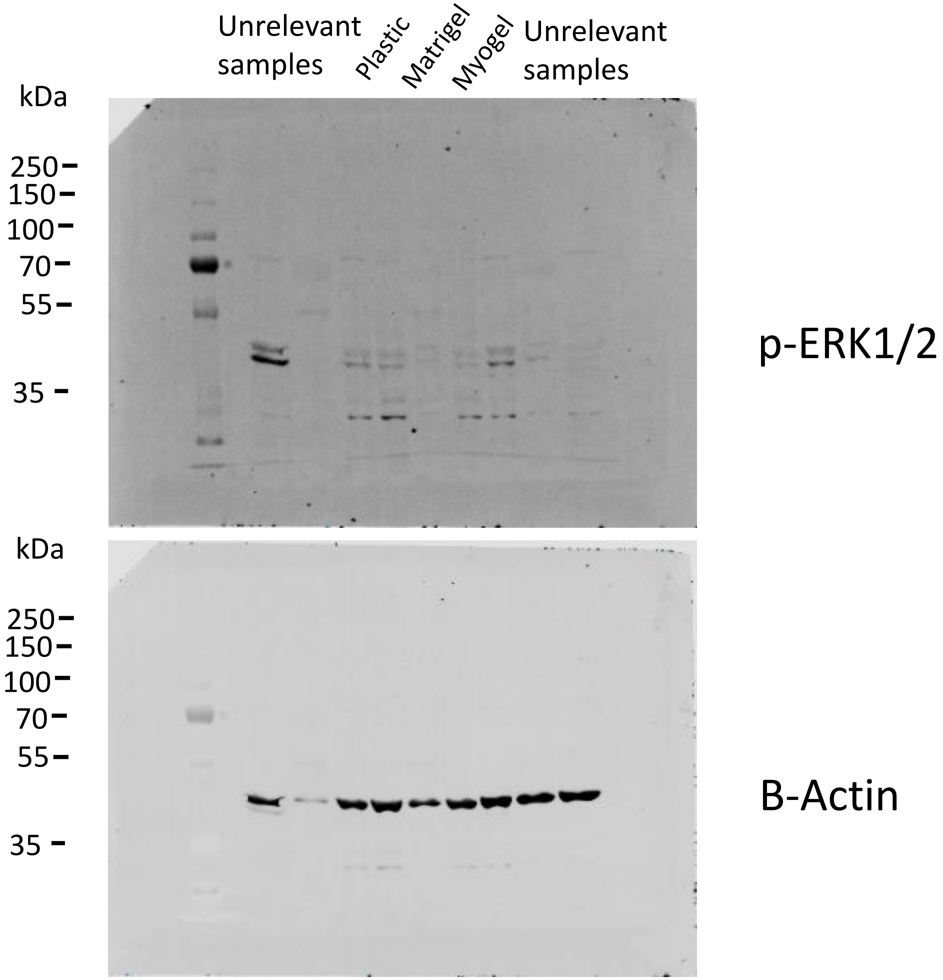

Experiment 2

	Densitometry readings		
	UTSCC-42B		
	plastic	matrigel	myogel
p-ERK1/2	2815,8	1005,0	1413,6
β-Actin	11657,0	7172,8	10163,8
Normalize d value	0,24	0,14	0,14

UTSCC-81

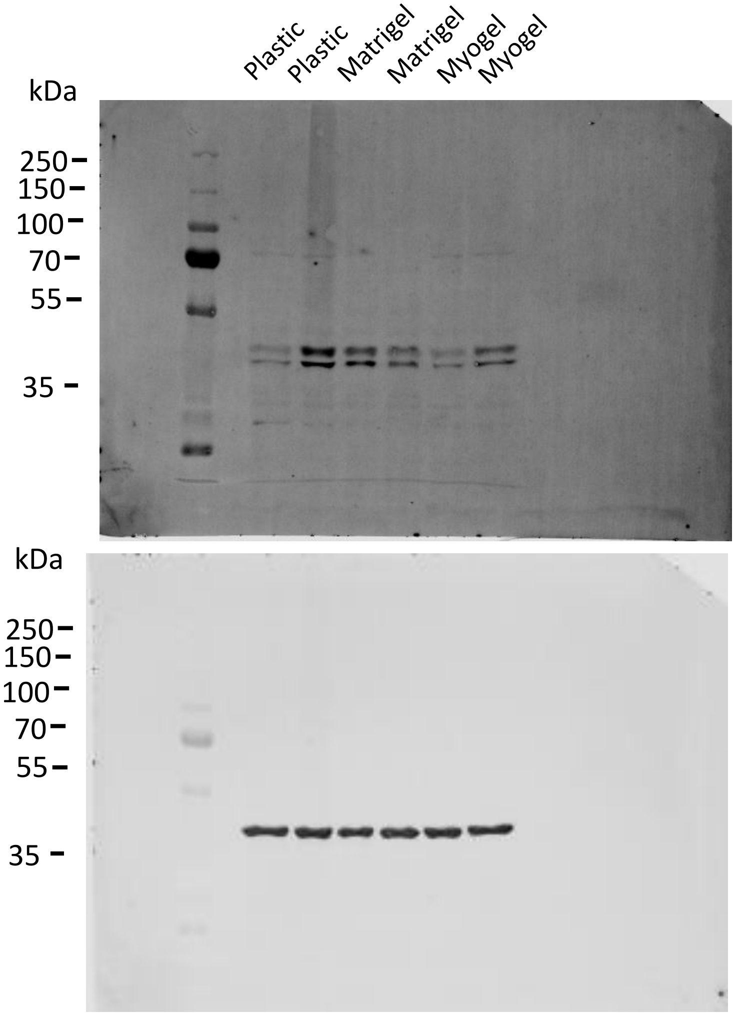

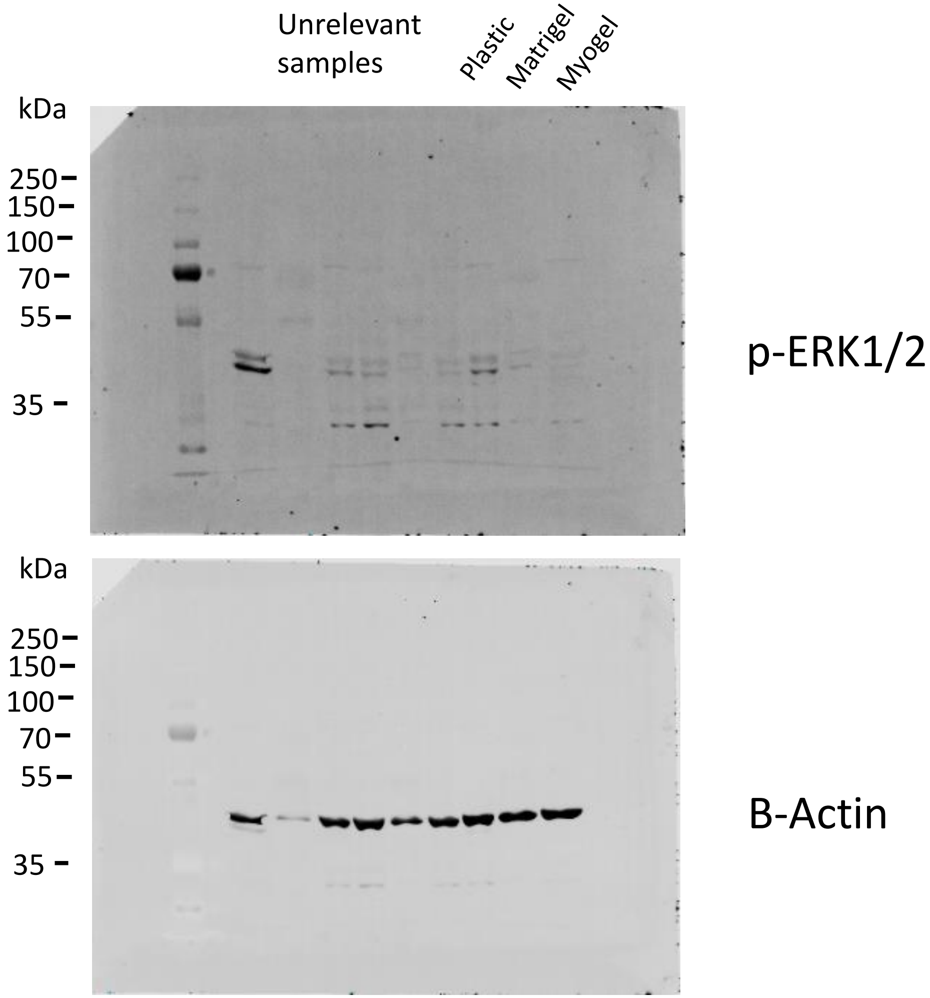

Experiment 1

	Densitometry readings					
	UTSCC-81					
	plastic	plastic	matrigel	matrigel	myogel	myogel
p-ERK1/2	4373,0	15863,3	9339,7	7335,7	3297,1	8186,4
β-Actin	21445,1	19691,9	16005,9	18417,2	18044,8	19642,8
Normalized value	0,20	0,81	0,58	0,40	0,18	0,42

Experiment 2

	Densitometry readings		
	UTSCC-81		
	plastic	matrigel	myogel
p-ERK1/2	4286,0	967,2	1236,2
β-Actin	13212,0	13836,9	15331,2
Normalized value	0,32	0,07	0,08

Immunoblot analysis of ERK1/2 in UTSCC-24A, UTSCC-24B, UTSCC-42A, UTSCC-42B, and UTSCC-81 cell lines growing on plastic, Matrigel and Myogel. β-Actin was used as the control to normalize the quantities of the proteins. Immunoblot figures, densitometry readings and normalized values are presented.

UTSCC-24A

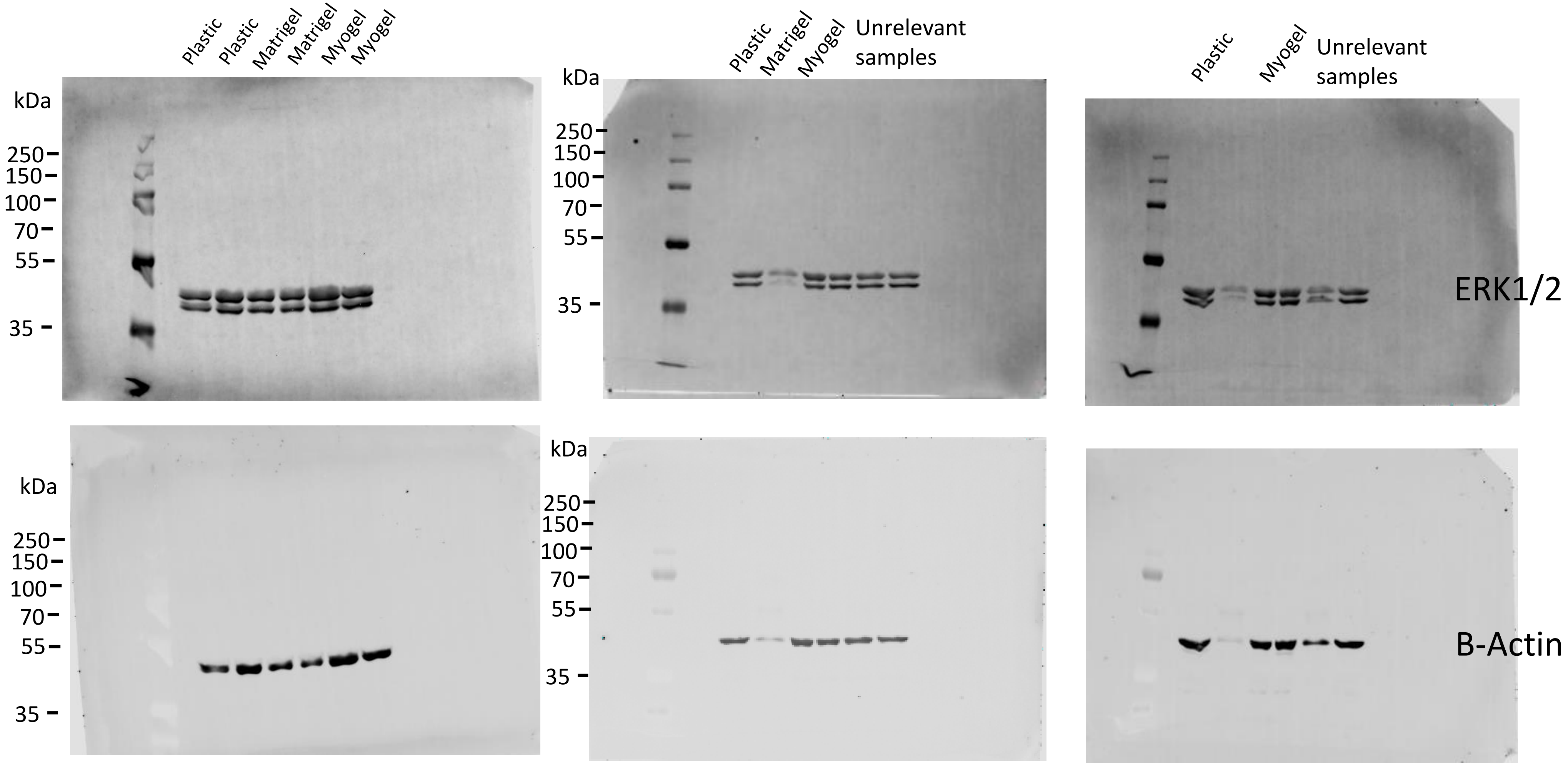

Experiment 1

	Densitometry readings					
	UTSCC-24A					
	plastic	plastic	matrigel	matrigel	myogel	myogel
ERK	14803,2	17700,6	14737,7	14197,8	17960,5	13890,8
β-Actin	12130,6	18201,2	12092,4	9537,9	21432,3	16521,2
Normalized value	1,22	0,97	1,22	1,49	0,84	0,84

Experiment 2

	Densitometry readings		
	UTSCC-24A		
	plastic	matrigel	myogel
ERK1/2	14558,0	3817,3	12282,0
β-Actin	18906,3	3115,9	16894,5
Normalized value	0,77	1,23	0,73

Experiment 3

	Densitometry readings		
	UTSCC-24A		
	plastic		myogel
ERK1/2	24431,7		15906,3
β-Actin	23136,2		15535,9
Normalized value	1,06		1,02

UTSCC-24B

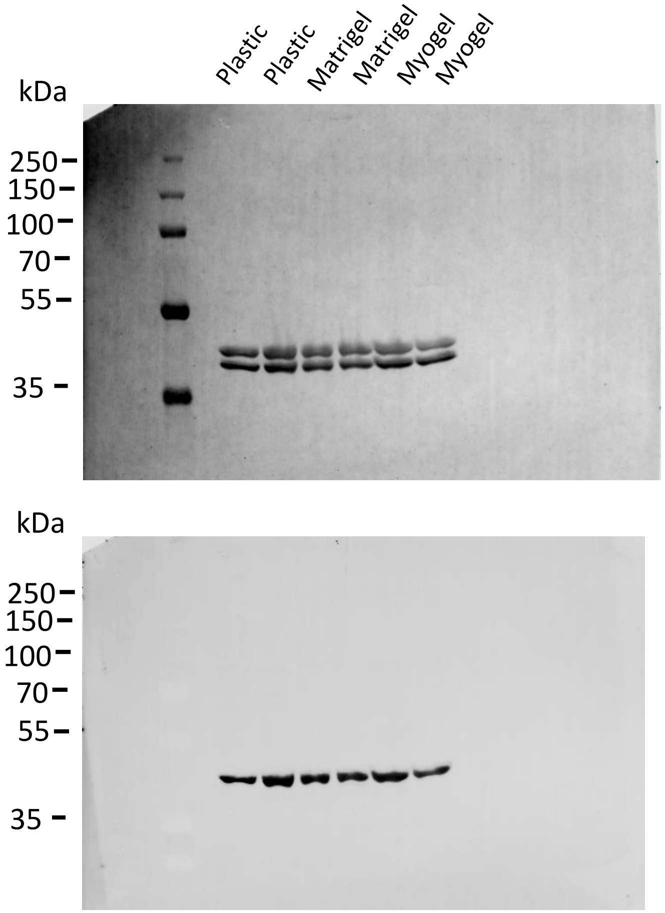

Experiment 1

	Densitometry readings					
	UTSCC-24B					
	plastic	plastic	matrigel	matrigel	myogel	myogel
ERK	10958,2	14003,8	10540,1	7800,1	8788,2	8322,0
β-Actin	13452,5	18790,1	14120,2	11813,7	15713,3	10299,8
Normalized value	0,81	0,75	0,75	0,66	0,56	0,81

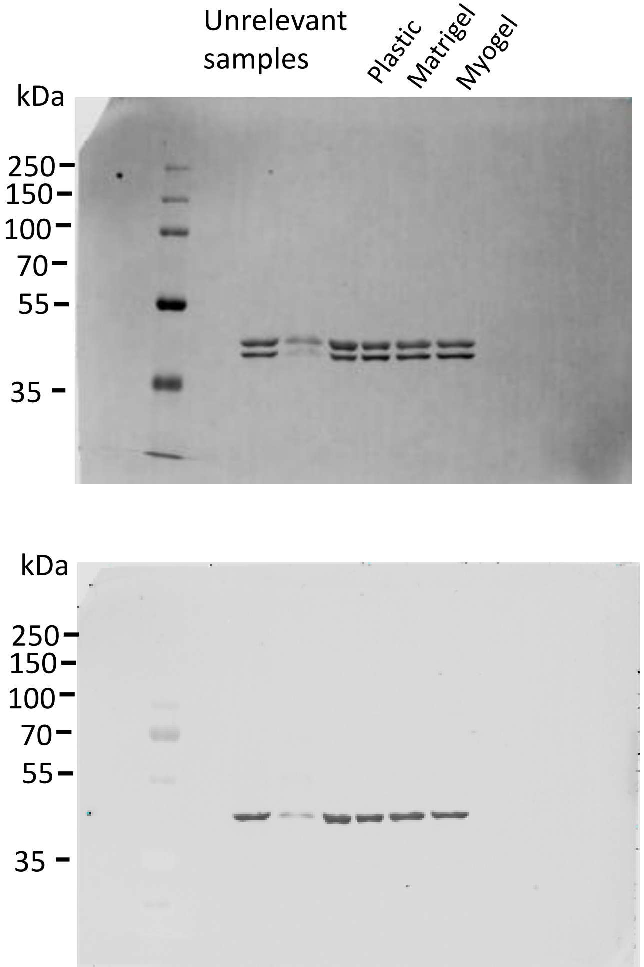

Experiment 2

	Densitometry readings		
	UTSCC-24B		
	plastic	matrigel	myogel
ERK1/2	10950,0	12319,1	13547,1
β-Actin	14438,8	17257,6	16974,2
Normalized value	0,76	0,71	0,80

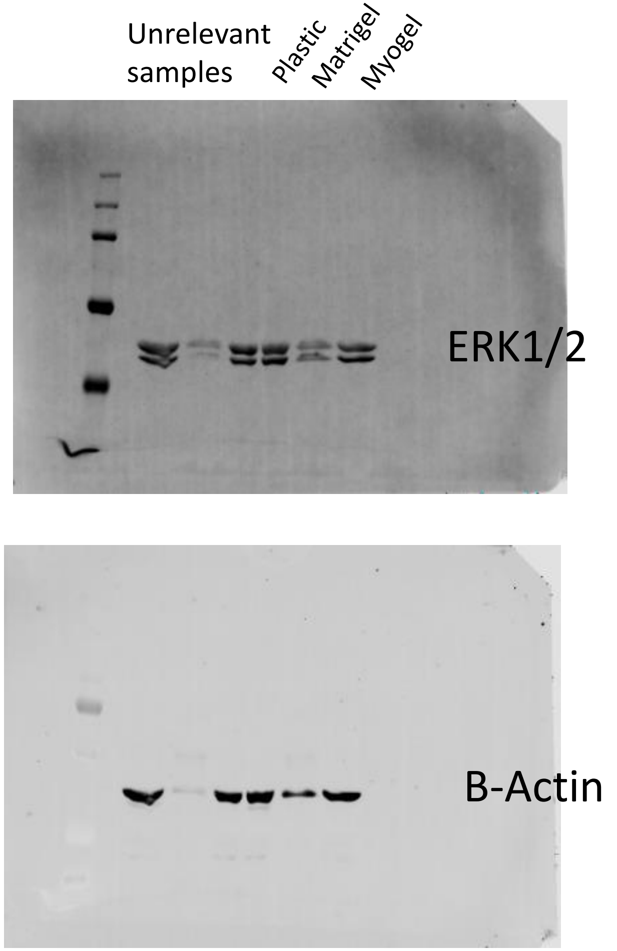

Experiment 3

	Densitometry readings		
	UTSCC-24B		
	plastic	matrigel	myogel
ERK1/2	16424,3	8837,7	17344,2
β-Actin	17109,6	9396,2	18146,1
Normalized value	0,96	0,94	0,96

UTSCC-42A

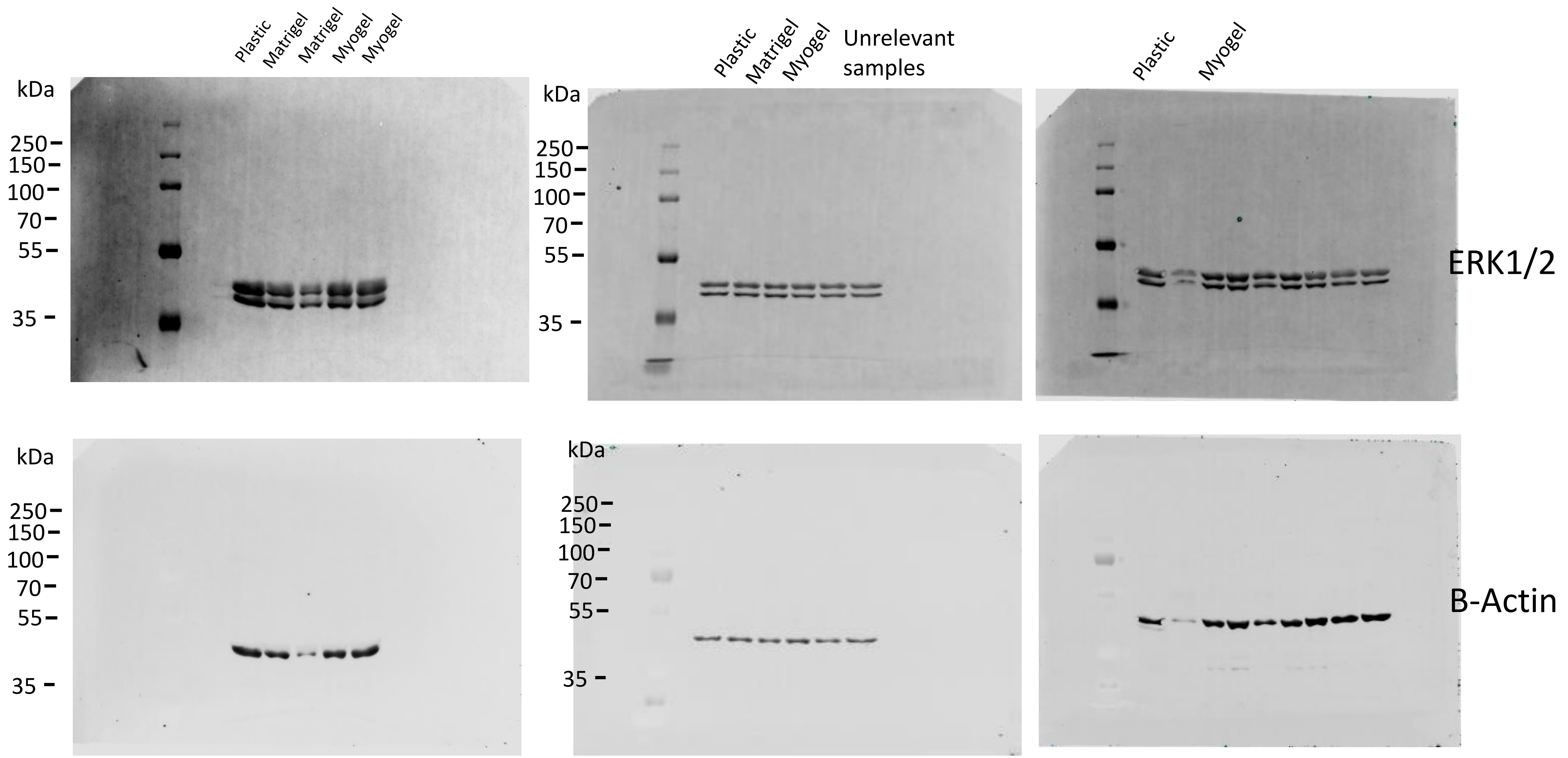

Experiment 1

	Densitometry readings				
	UTSCC-42A				
	plastic	matrigel	matrigel	myogel	myogel
ERK	14257,5	9439,9	7022,6	14122,0	15066,1
β-Actin	24447,7	14963,2	4111,0	18660,4	24913,0
Normalized value	0,58	0,63	1,71	0,76	0,60

Experiment 2

	Densitometry readings		
	UTSCC-42A		
	plastic	matrigel	myogel
ERK1/2	17102,6	17142,6	14428,1
β-Actin	13839,6	15140,1	11709,6
Normalized value	1,24	1,13	1,23

Experiment 3

	Densitometry readings		
	UTSCC-42A		
	plastic	matrigel	myogel
ERK1/2	11915,6	3672,2	11465,8
β-Actin	10614,0	1045,2	9608,5
Normalized value	1,45	2,07	1,15

UTSCC-42B

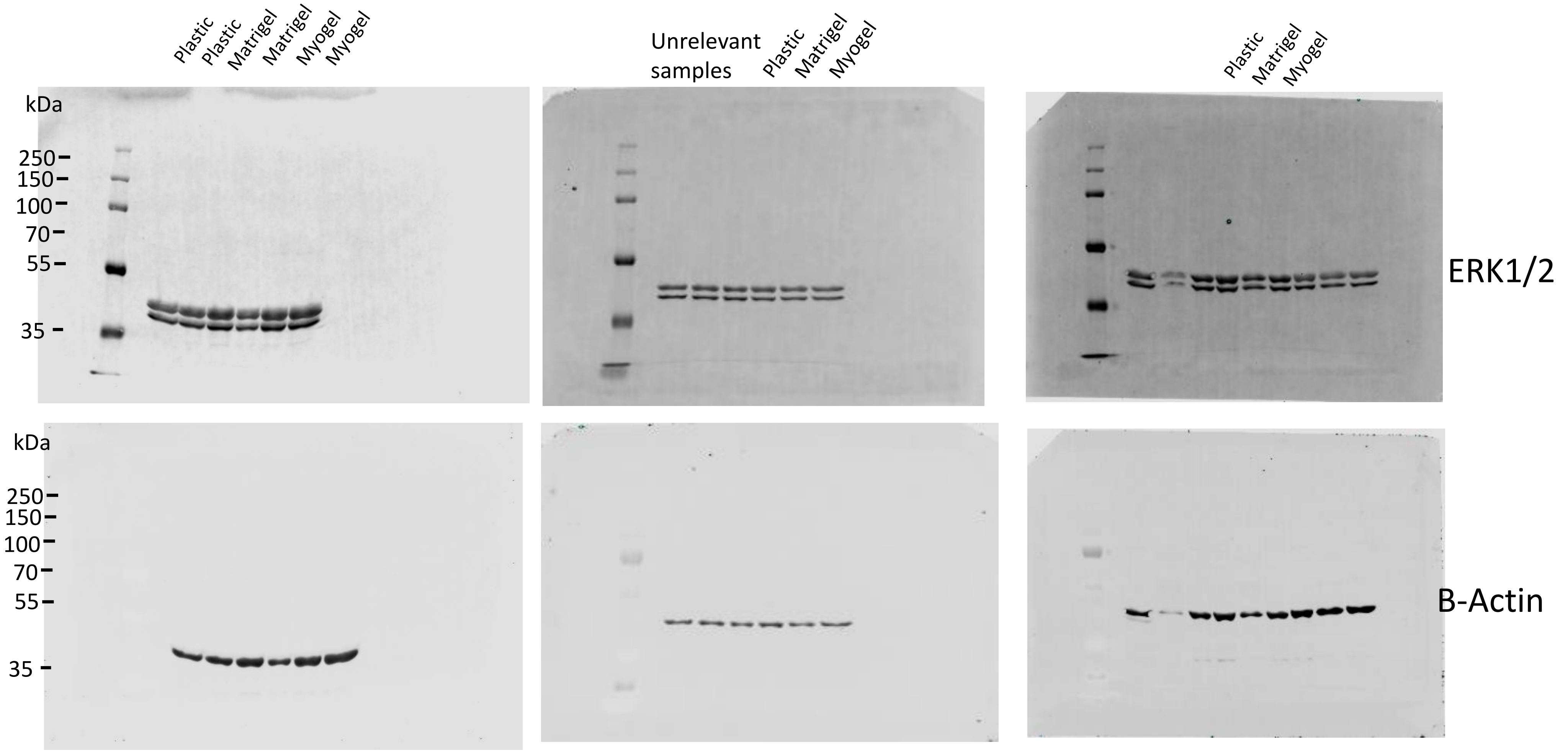

Experiment 1

	Densitometry readings					
	UTSCC-42B					
	plastic	plastic	matrigel	matrigel	myogel	myogel
ERK	8981,5	8560,7	15189,1	7883,8	12998,5	16786,3
β-Actin	13637,7	14339,6	19790,4	9868,6	18300,5	23912,7
Normalized value	0,66	0,60	0,77	0,80	0,71	0,70

Experiment 2

	Densitometry readings		
	UTSCC-42B		
	plastic	matrigel	myogel
ERK1/2	12486,6	12160,8	12652,5
β-Actin	15174,6	11072,8	14592,6
Normalized value	0,82	1,10	0,87

Experiment 3

	Densitometry readings		
	UTSCC-42B		
	plastic	matrigel	myogel
ERK1/2	12327,1	8182,8	9407,7
β-Actin	11657,0	7172,8	10163,8
Normalized value	1,06	1,14	0,93

UTSCC-81

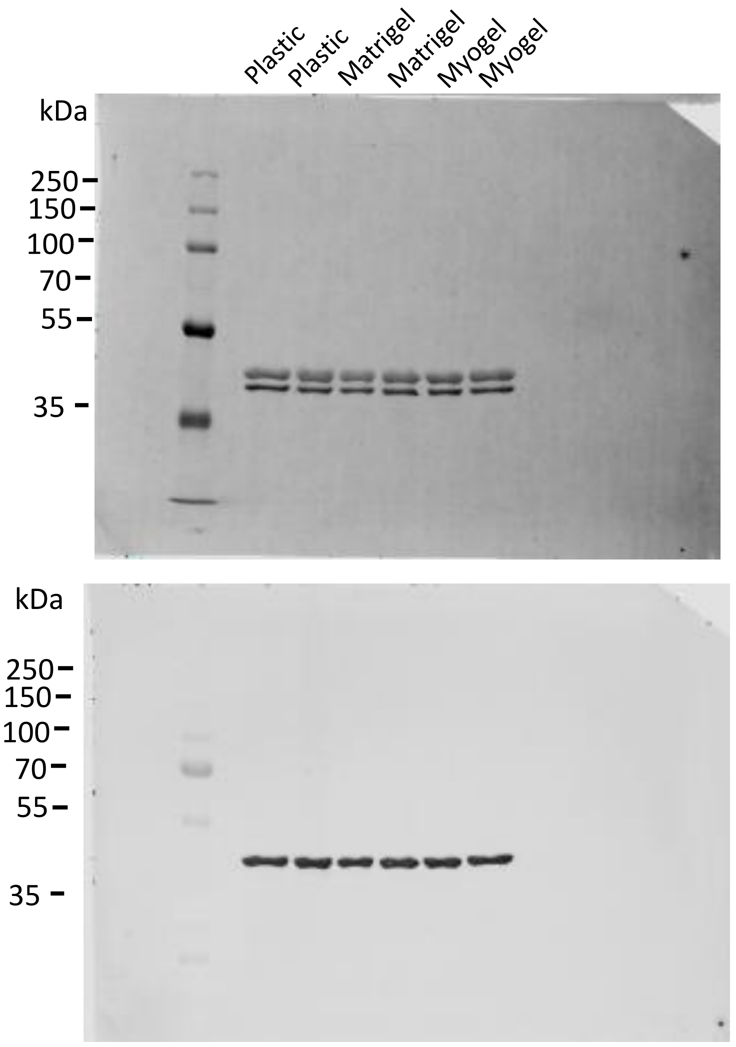

Experiment 1

	Densitometry readings					
	UTSCC-81					
	plastic	plastic	matrigel	matrigel	myogel	myogel
ERK1/2	15270,0	12180,5	9298,3	12323,5	11857,2	13850,7
β-Actin	21445,1	19691,9	16005,9	18417,2	18044,8	19642,8
Normalized value	0,71	0,62	0,58	0,67	0,66	0,71

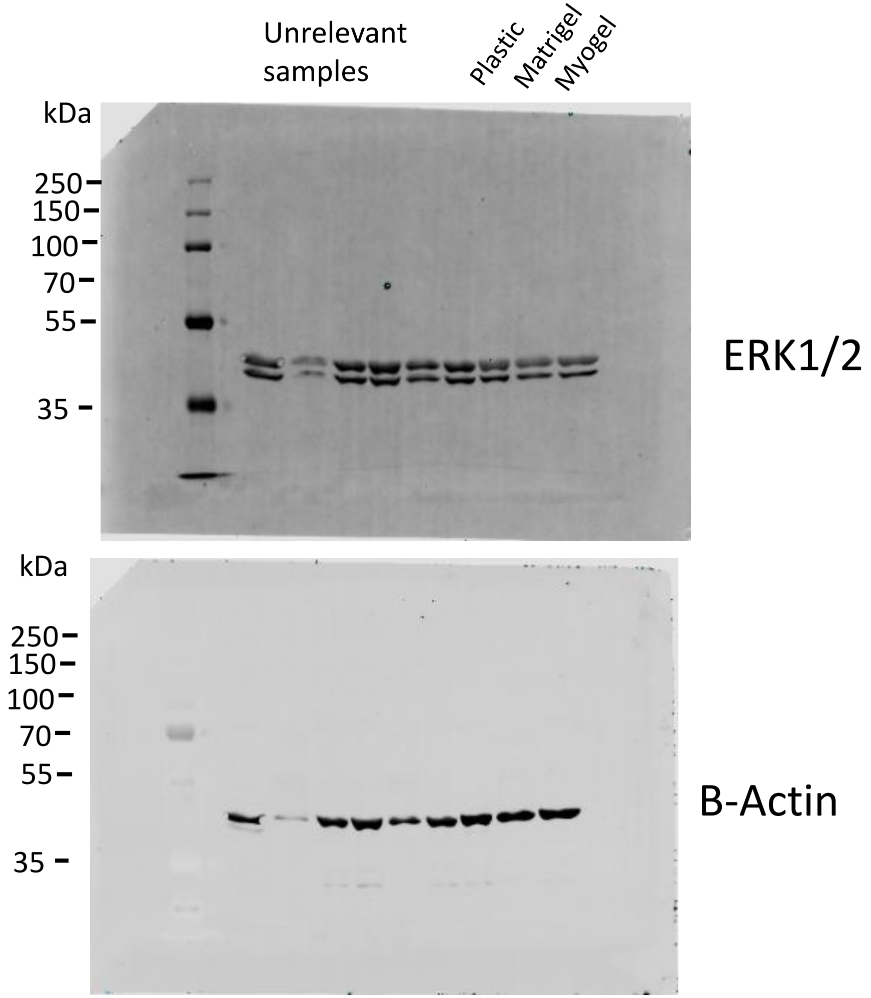

Experiment 2

	Densitometry readings		
	UTSCC-81		
	plastic	matrigel	myogel
ERK1/2	7628,5	7317,5	8213,9
β-Actin	13212,0	13836,9	15331,2
Normalized value	0,58	0,53	0,54
