# Supplementary material for: Human Tumor–Derived Matrix Improves the Predictability of Head and Neck Cancer Drug Testing
Source: Cancers (Basel). 2019 Dec 30;12(1):92. doi: 10.3390/cancers12010092 (PMC7017272; doi:10.3390/cancers12010092)
Supplement: Supplementary file 1 [file cancers-12-00092-s001.zip › cancers-664648-supplement-final/Supplementary Figure 6.pdf]

Supplementary Figure 6: Dose response curves of drugs for each conditions.

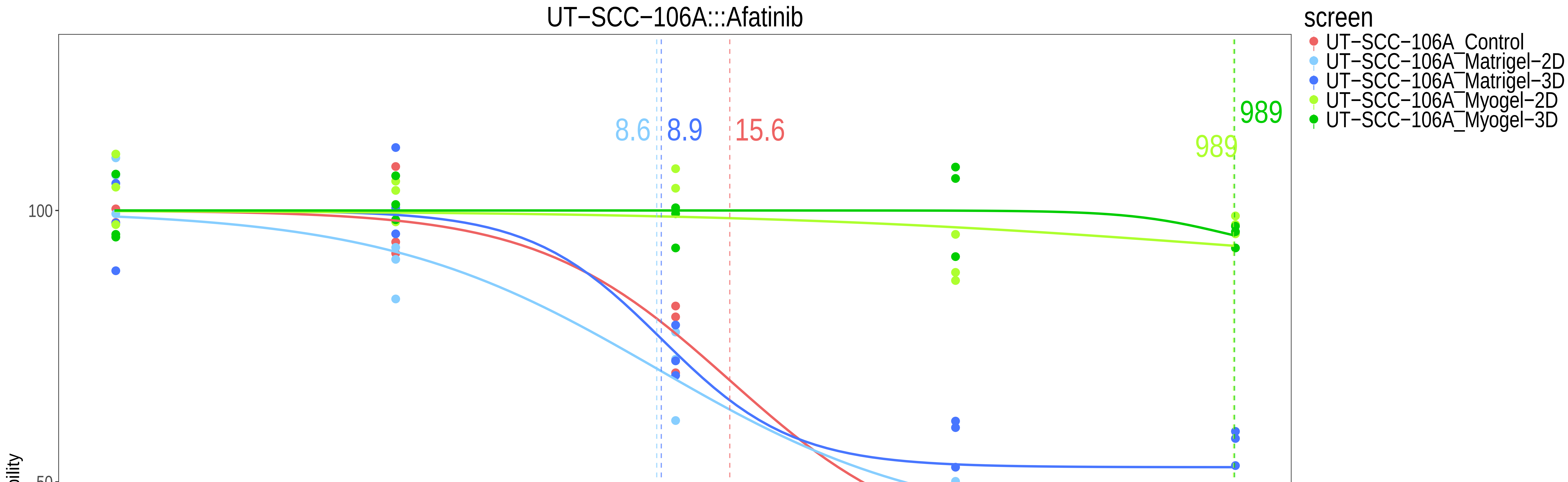

	screen	drug_name	DSS	EC50
1	UT−SCC−106A_Control	Afatinib	13.2	15.6
2	UT−SCC−106A_Matrigel−2D	Afatinib	13.7	8.6
3	UT−SCC−106A_Matrigel−3D	Afatinib	11.7	8.9
4	UT−SCC−106A_Myogel−2D	Afatinib	0.0	989.0
5	UT−SCC−106A_Myogel−3D	Afatinib	0.0	989.0

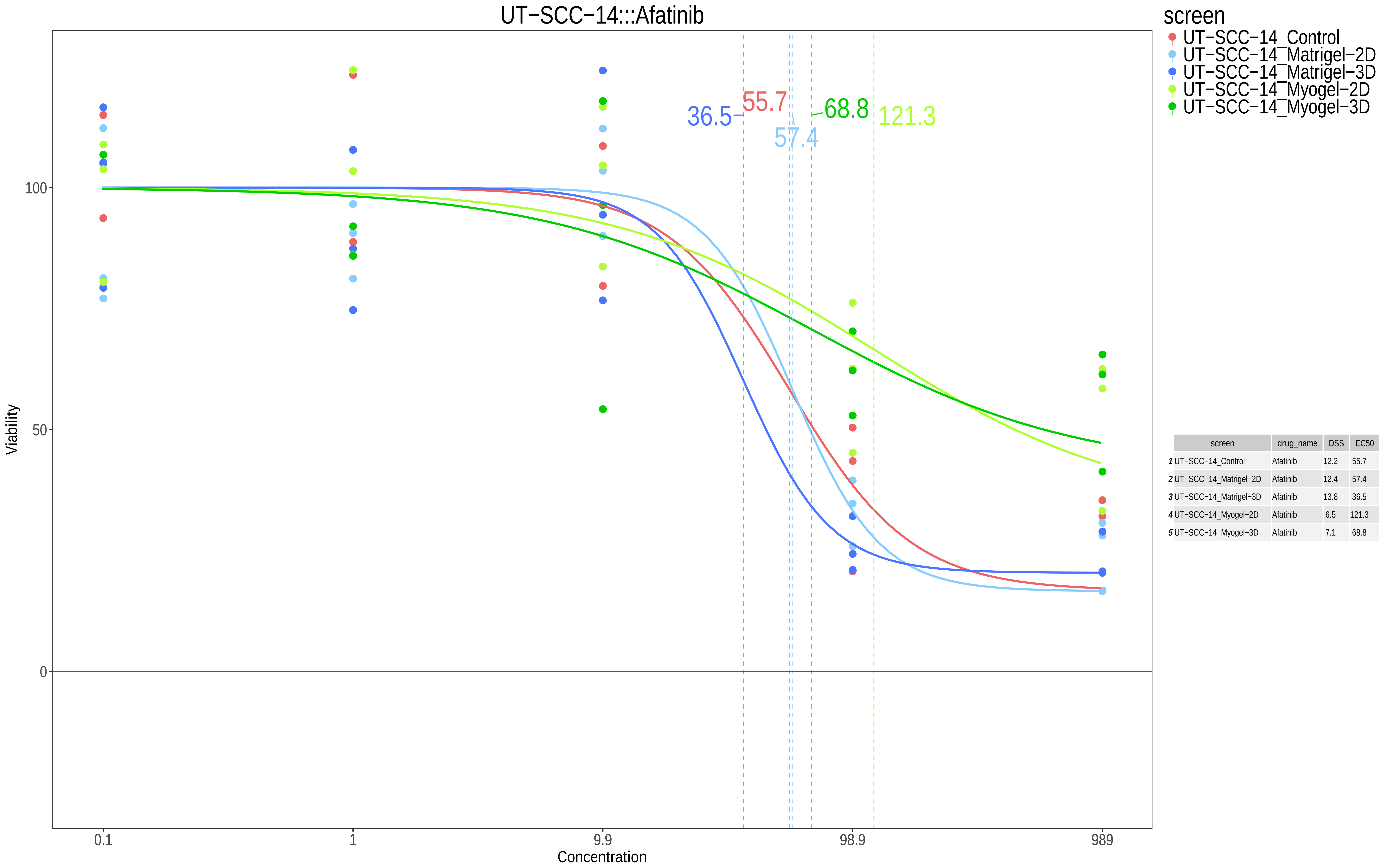

	screen	drug_name	DSS	EC50
1	UT−SCC−14_Control	Afatinib	12.2	55.7
2	UT−SCC−14_Matrigel−2D	Afatinib	12.4	57.4
3	UT−SCC−14_Matrigel−3D	Afatinib	13.8	36.5
4	UT−SCC−14_Myogel−2D	Afatinib	6.5	121.3
5	UT−SCC−14_Myogel−3D	Afatinib	7.1	68.8

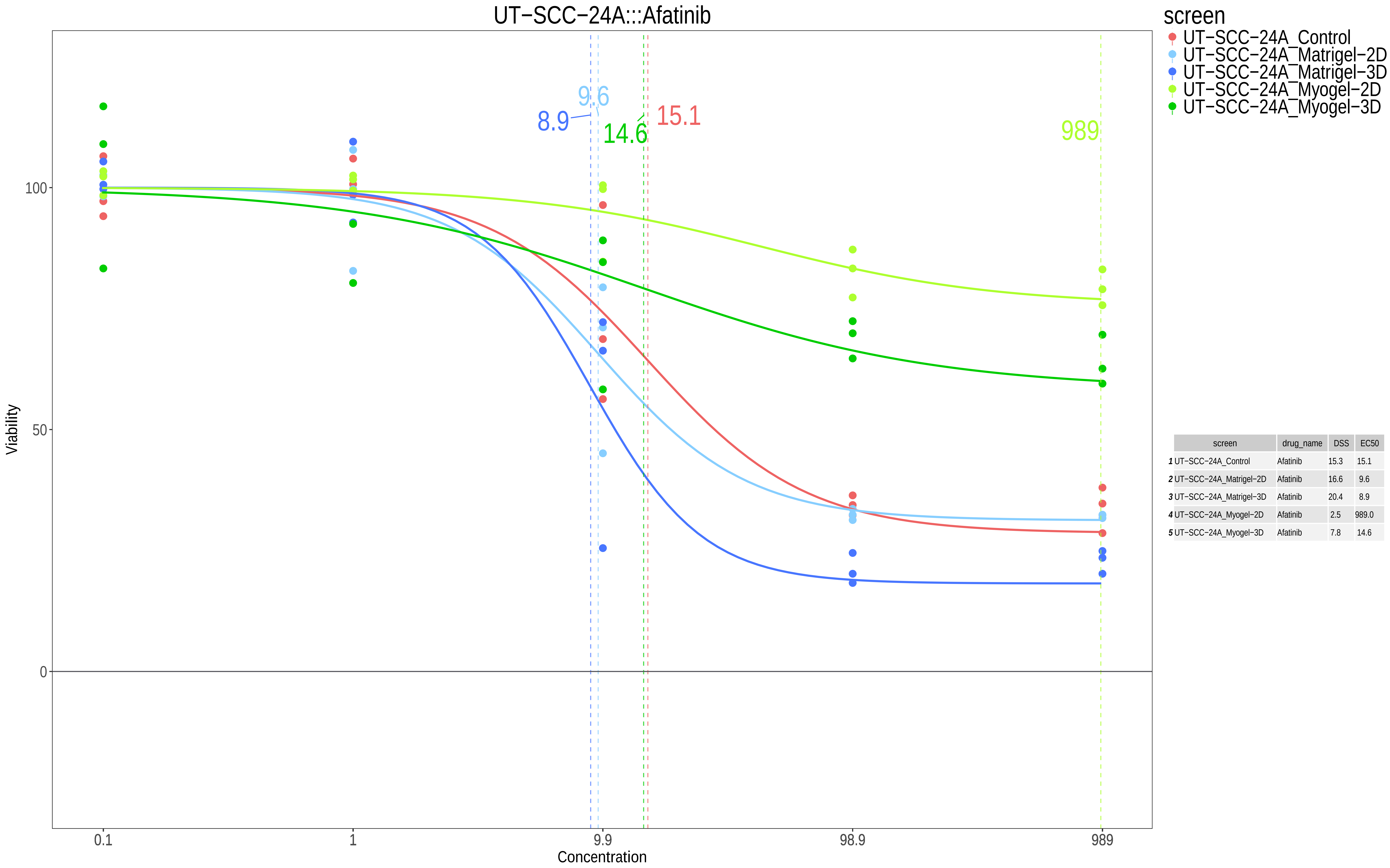

	screen	drug_name	DSS	EC50
1	UT−SCC−24A_Control	Afatinib	15.3	15.1
2	UT−SCC−24A_Matrigel−2D	Afatinib	16.6	9.6
3	UT−SCC−24A_Matrigel−3D	Afatinib	20.4	8.9
4	UT−SCC−24A_Myogel−2D	Afatinib	2.5	989.0
5	UT−SCC−24A_Myogel−3D	Afatinib	7.8	14.6

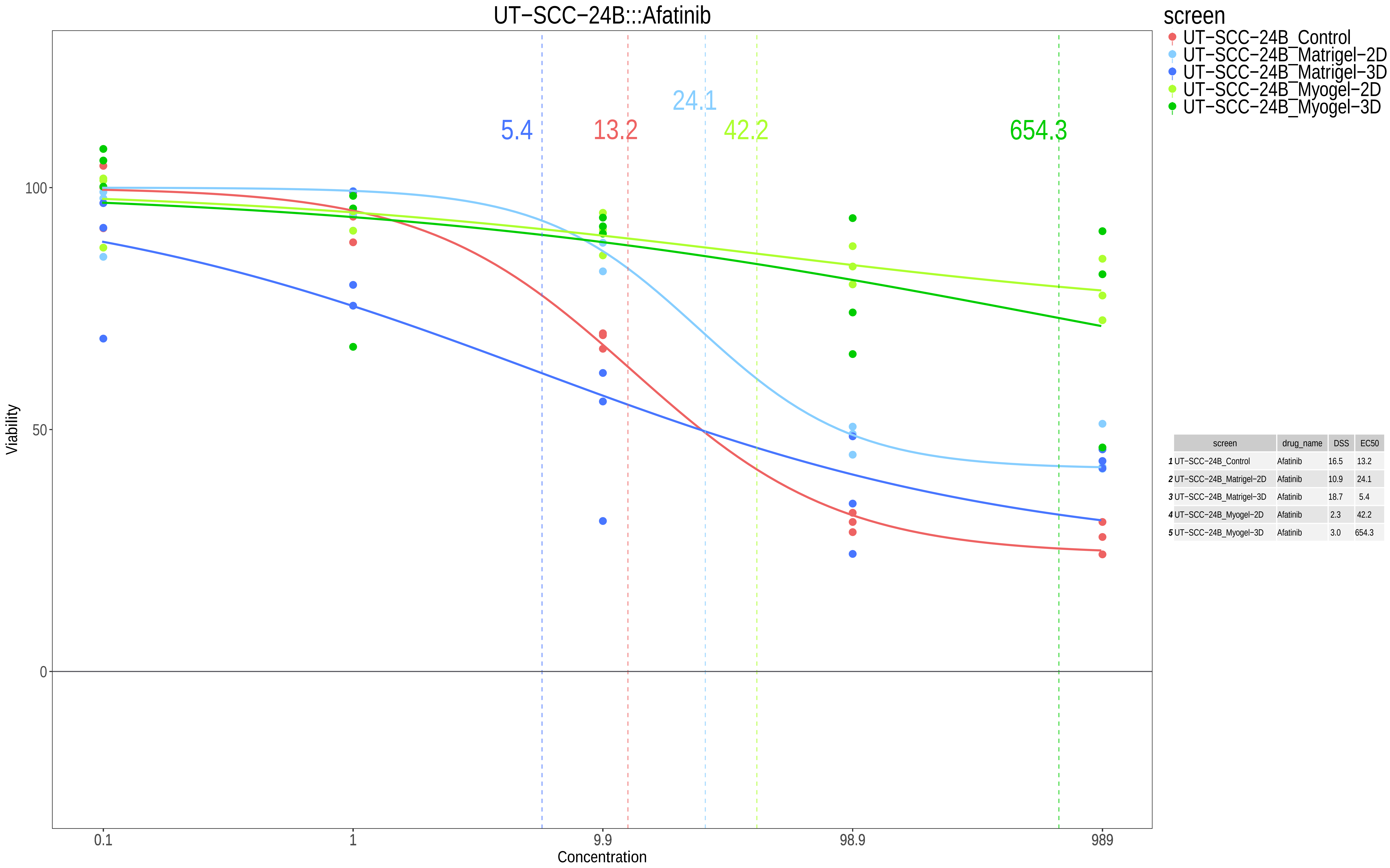

	screen	drug_name	DSS	EC50
1	UT−SCC−24B_Control	Afatinib	16.5	13.2
2	UT−SCC−24B_Matrigel−2D	Afatinib	10.9	24.1
3	UT−SCC−24B_Matrigel−3D	Afatinib	18.7	5.4
4	UT−SCC−24B_Myogel−2D	Afatinib	2.3	42.2
5	UT−SCC−24B_Myogel−3D	Afatinib	3.0	654.3

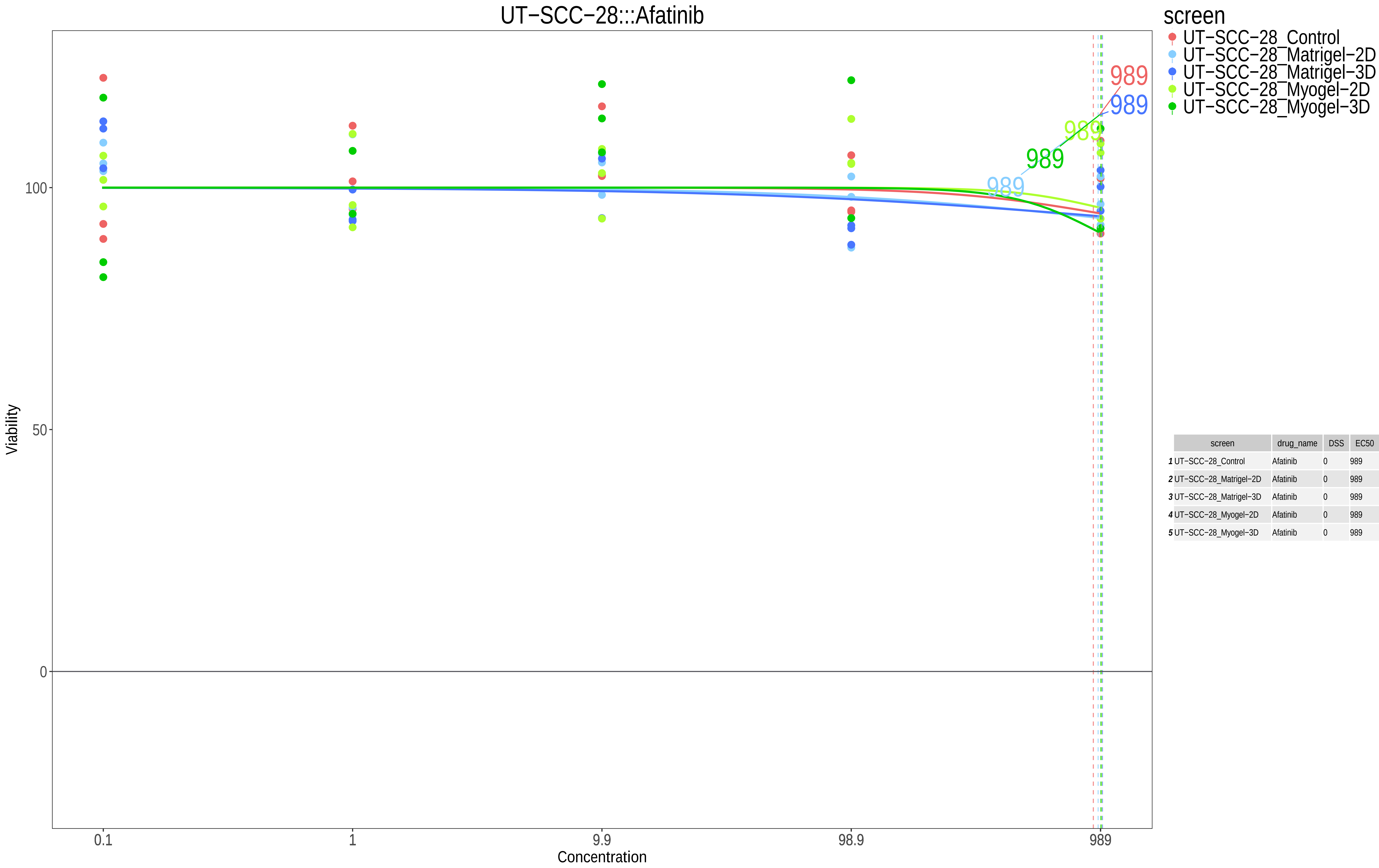

	screen	drug_name	DSS	EC50
1	UT−SCC−28_Control	Afatinib	0	989
2	UT−SCC−28_Matrigel−2D	Afatinib	0	989
3	UT−SCC−28_Matrigel−3D	Afatinib	0	989
4	UT−SCC−28_Myogel−2D	Afatinib	0	989
5	UT−SCC−28_Myogel−3D	Afatinib	0	989

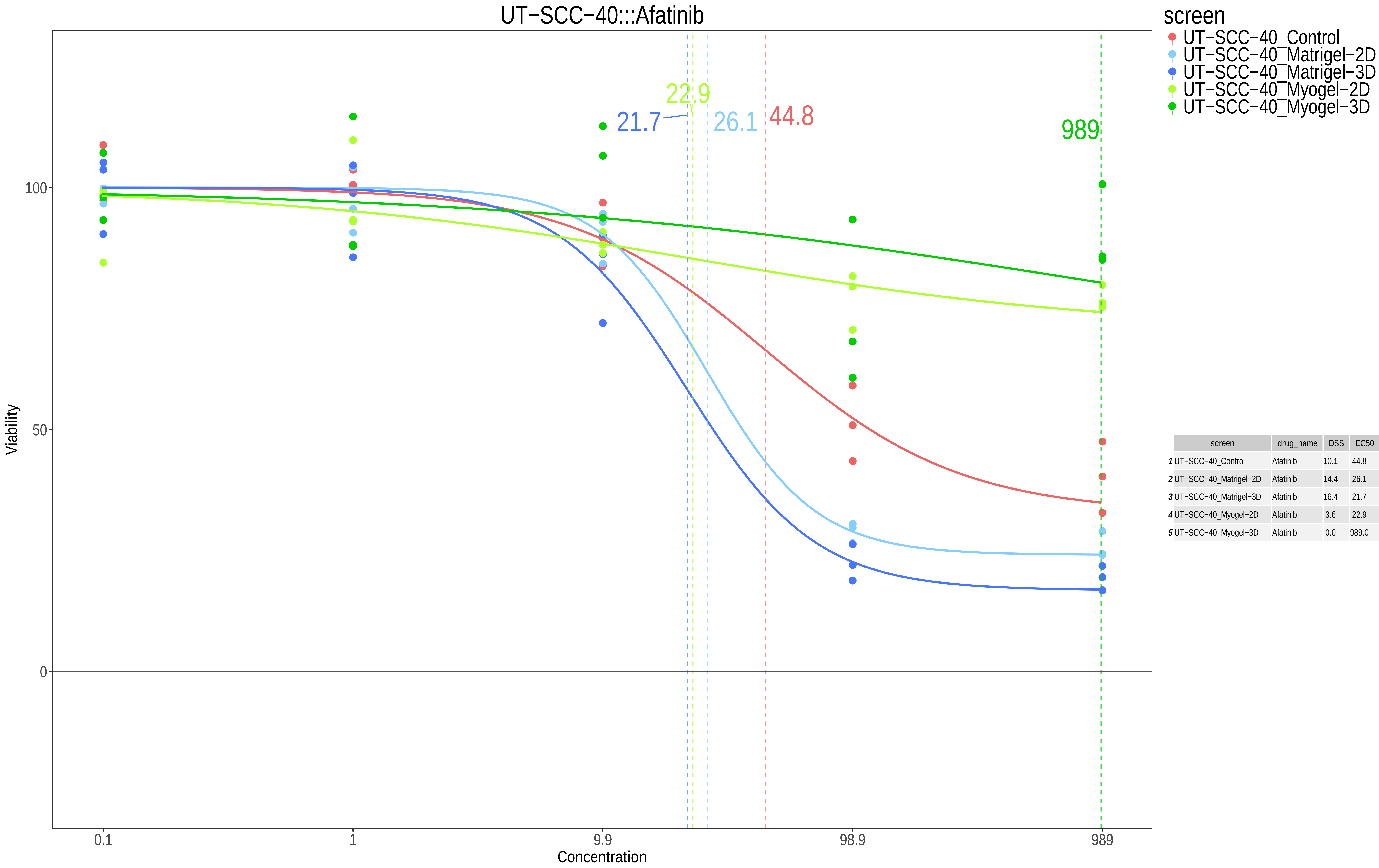

	screen	drug_name	DSS	EC50
1	UT−SCC−40_Control	Afatinib	10.1	44.8
2	UT−SCC−40_Matrigel−2D	Afatinib	14.4	26.1
3	UT−SCC−40_Matrigel−3D	Afatinib	16.4	21.7
4	UT−SCC−40_Myogel−2D	Afatinib	3.6	22.9
5	UT−SCC−40_Myogel−3D	Afatinib	0.0	989.0

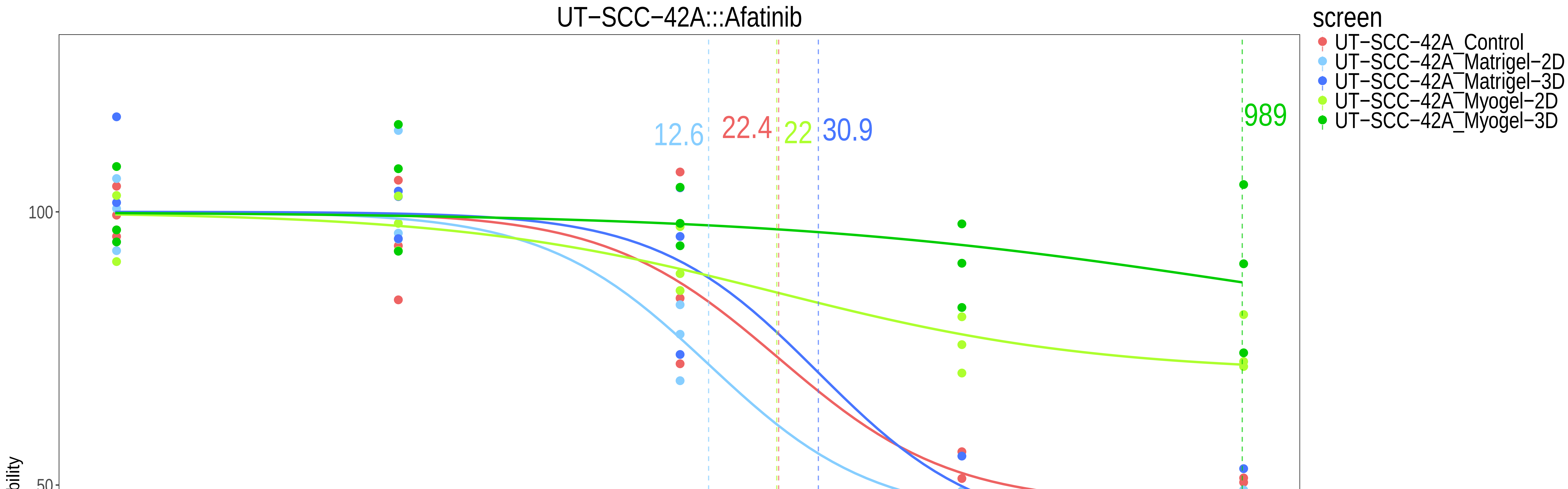

	screen	drug_name	DSS	EC50
1	UT−SCC−42A_Control	Afatinib	10.3	22.4
2	UT−SCC−42A_Matrigel−2D	Afatinib	12.6	12.6
3	UT−SCC−42A_Matrigel−3D	Afatinib	10.3	30.9
4	UT−SCC−42A_Myogel−2D	Afatinib	4.2	22.0
5	UT−SCC−42A_Myogel−3D	Afatinib	0.0	989.0

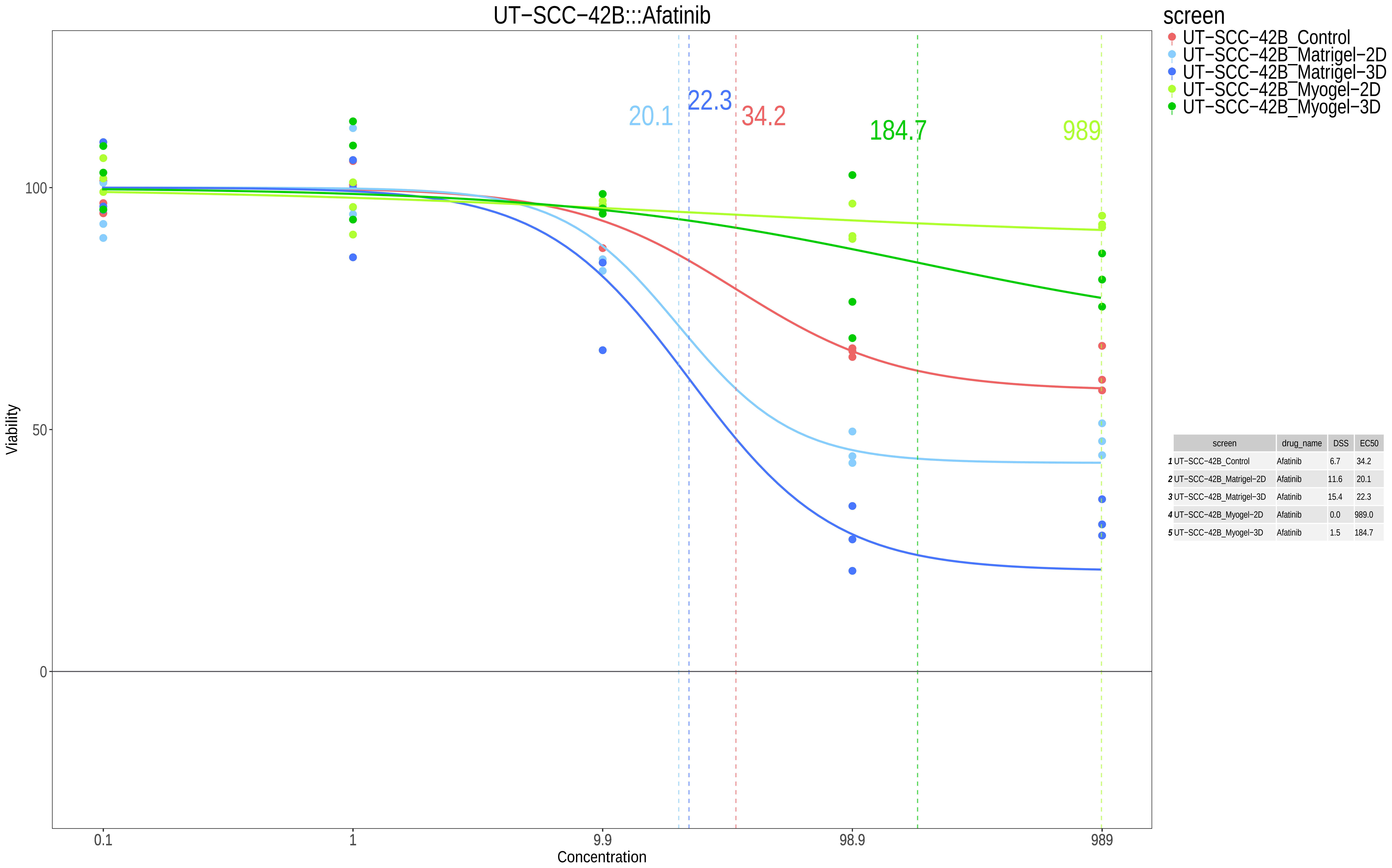

	screen	drug_name	DSS	EC50
1	UT−SCC−42B_Control	Afatinib	6.7	34.2
2	UT−SCC−42B_Matrigel−2D	Afatinib	11.6	20.1
3	UT−SCC−42B_Matrigel−3D	Afatinib	15.4	22.3
4	UT−SCC−42B_Myogel−2D	Afatinib	0.0	989.0
5	UT−SCC−42B_Myogel−3D	Afatinib	1.5	184.7

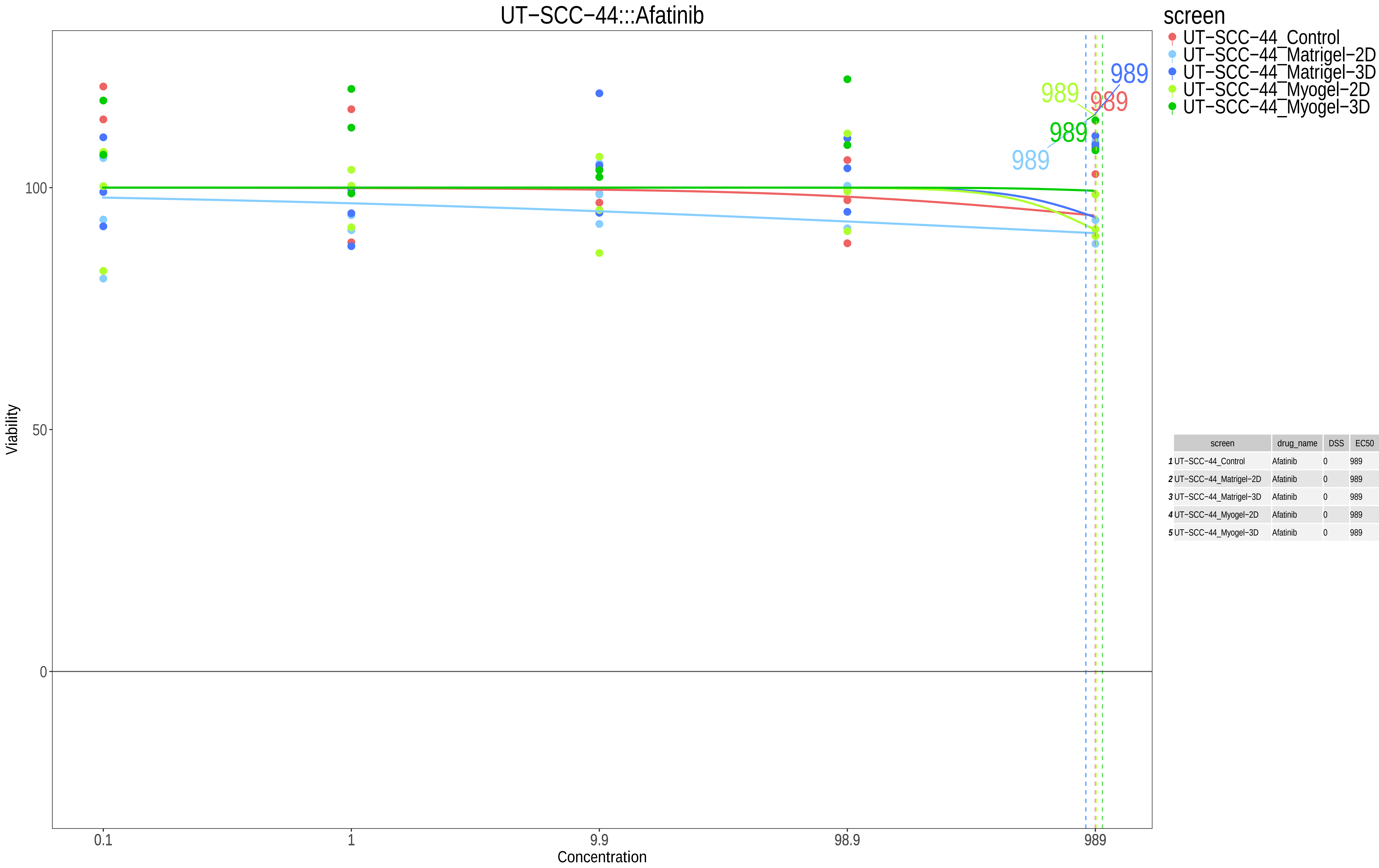

	screen	drug_name	DSS	EC50
1	UT−SCC−44_Control	Afatinib	0	989
2	UT−SCC−44_Matrigel−2D	Afatinib	0	989
3	UT−SCC−44_Matrigel−3D	Afatinib	0	989
4	UT−SCC−44_Myogel−2D	Afatinib	0	989
5	UT−SCC−44_Myogel−3D	Afatinib	0	989

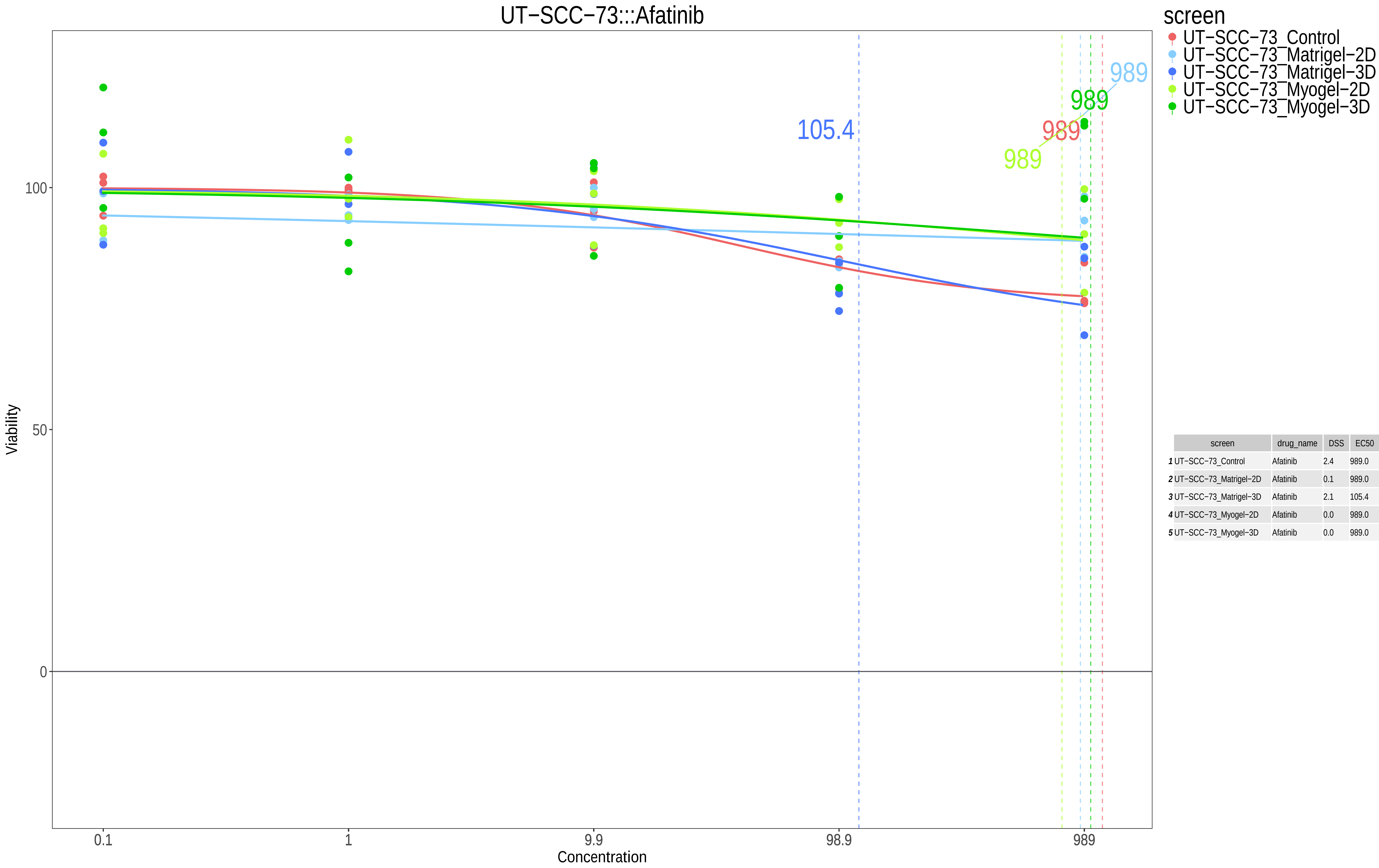

	screen	drug_name	DSS	EC50
1	UT−SCC−73_Control	Afatinib	2.4	989.0
2	UT−SCC−73_Matrigel−2D	Afatinib	0.1	989.0
3	UT−SCC−73_Matrigel−3D	Afatinib	2.1	105.4
4	UT−SCC−73_Myogel−2D	Afatinib	0.0	989.0
5	UT−SCC−73_Myogel−3D	Afatinib	0.0	989.0

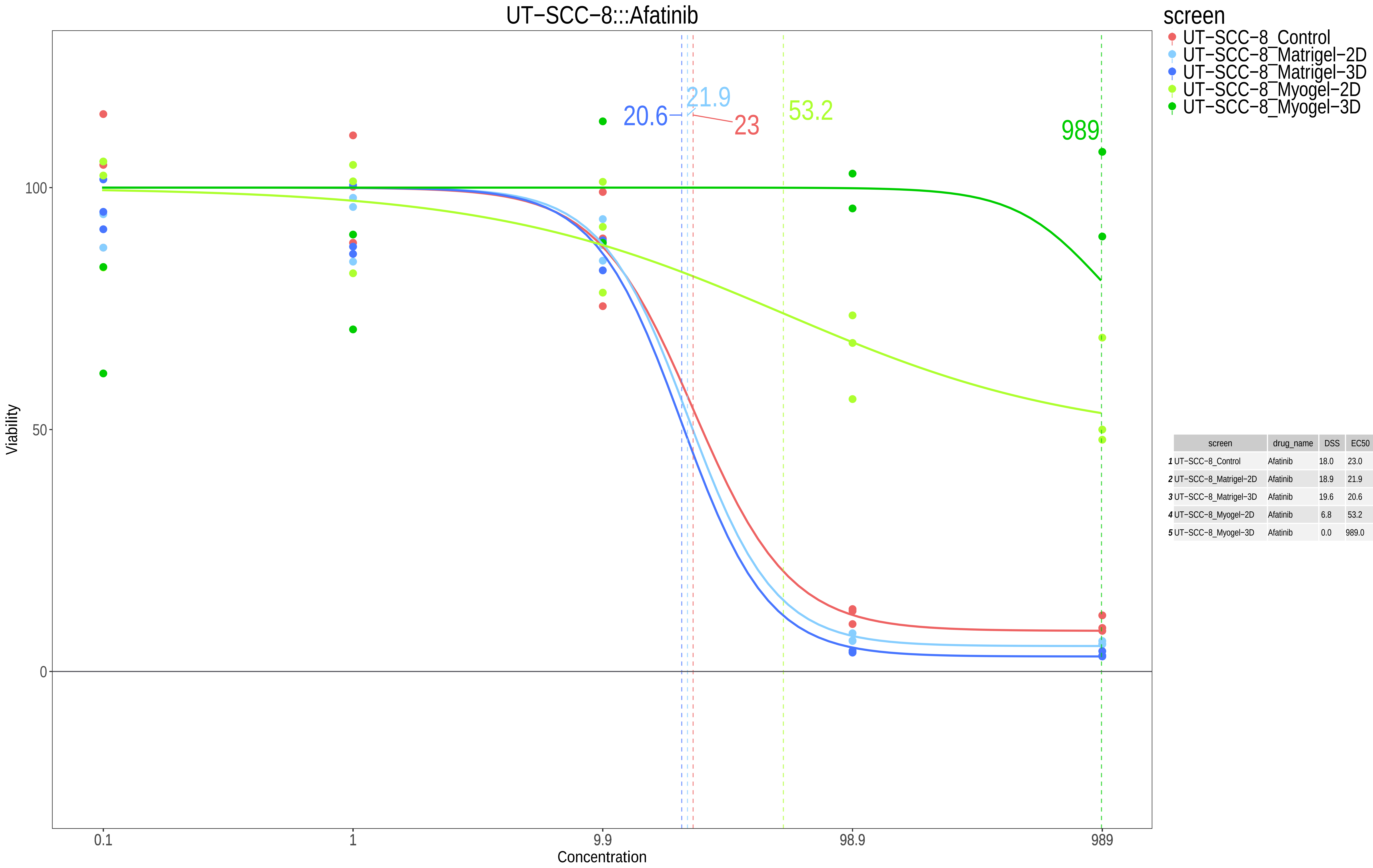

	screen	drug_name	DSS	EC50
1	UT−SCC−8_Control	Afatinib	18.0	23.0
2	UT−SCC−8_Matrigel−2D	Afatinib	18.9	21.9
3	UT−SCC−8_Matrigel−3D	Afatinib	19.6	20.6
4	UT−SCC−8_Myogel−2D	Afatinib	6.8	53.2
5	UT−SCC−8_Myogel−3D	Afatinib	0.0	989.0

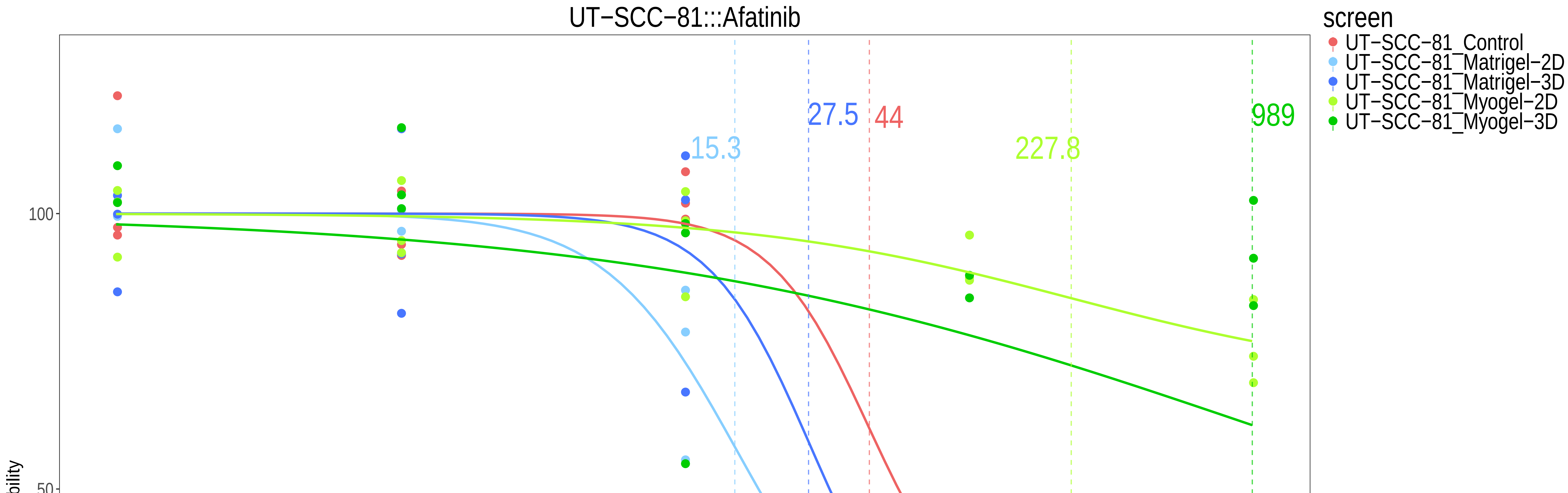

	screen	drug_name	DSS	EC50
1	UT−SCC−81_Control	Afatinib	12.6	44.0
2	UT−SCC−81_Matrigel−2D	Afatinib	18.9	15.3
3	UT−SCC−81_Matrigel−3D	Afatinib	15.9	27.5
4	UT−SCC−81_Myogel−2D	Afatinib	1.3	227.8
5	UT−SCC−81_Myogel−3D	Afatinib	0.0	989.0

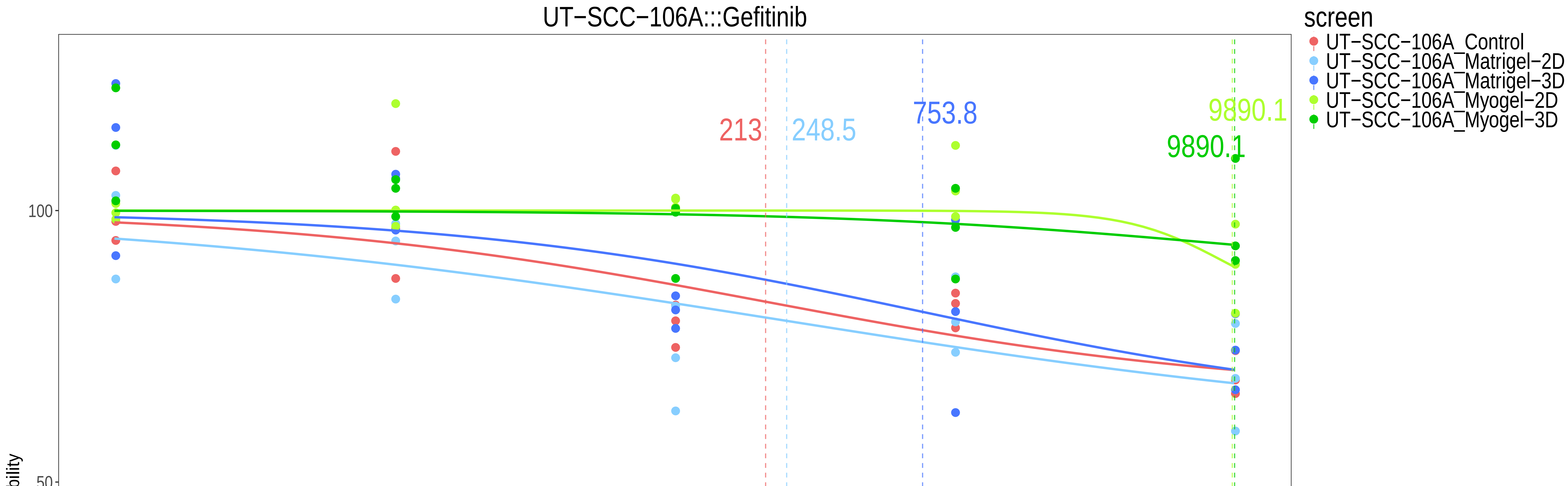

	screen	drug_name	DSS	EC50
1	UT−SCC−106A_Control	Gefitinib	4.7	213.0
2	UT−SCC−106A_Matrigel−2D	Gefitinib	5.7	248.5
3	UT−SCC−106A_Matrigel−3D	Gefitinib	3.5	753.8
4	UT−SCC−106A_Myogel−2D	Gefitinib	0.0	9890.1
5	UT−SCC−106A_Myogel−3D	Gefitinib	0.0	9890.1

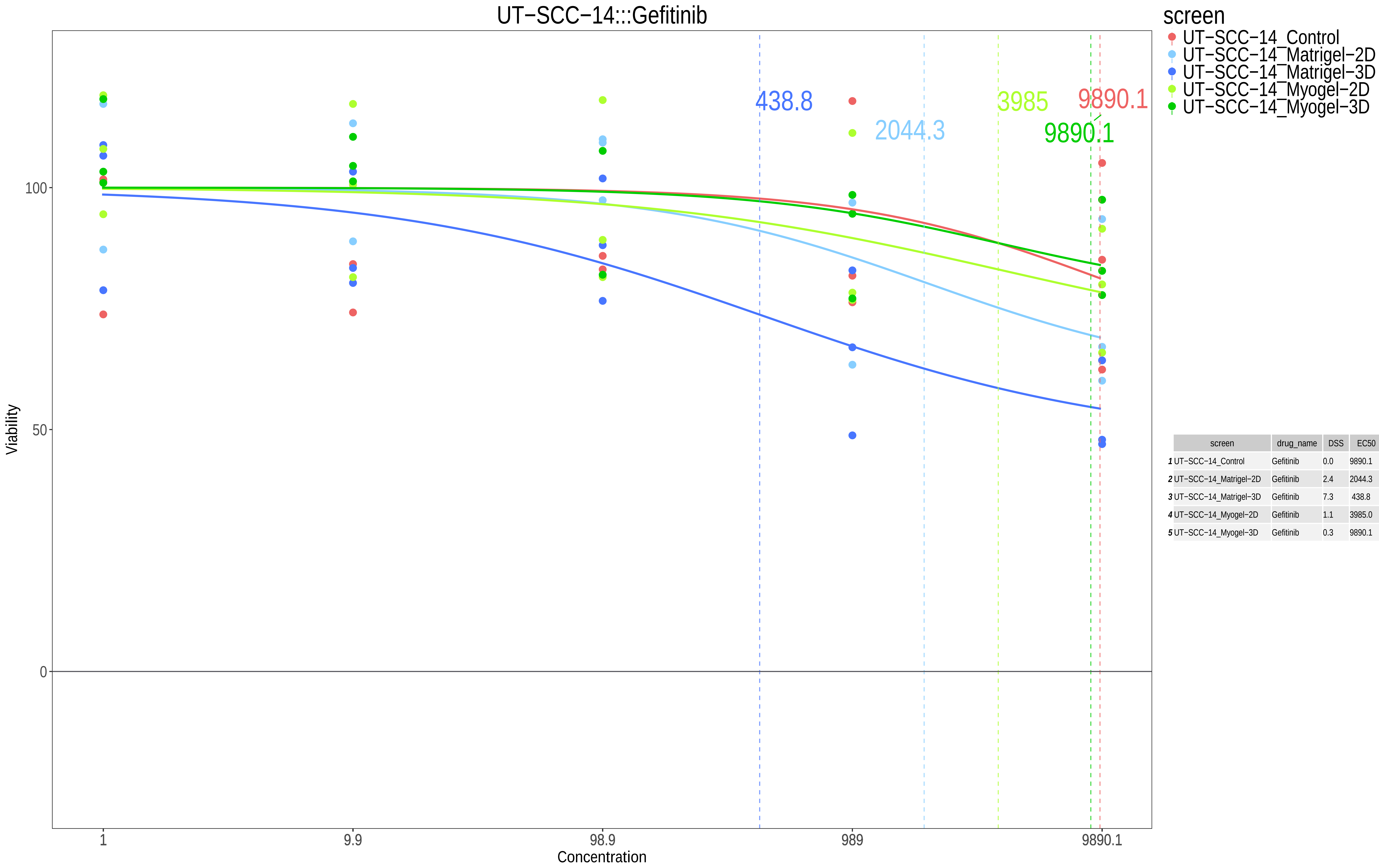

	screen	drug_name	DSS	EC50
1	UT−SCC−14_Control	Gefitinib	0.0	9890.1
2	UT−SCC−14_Matrigel−2D	Gefitinib	2.4	2044.3
3	UT−SCC−14_Matrigel−3D	Gefitinib	7.3	438.8
4	UT−SCC−14_Myogel−2D	Gefitinib	1.1	3985.0
5	UT−SCC−14_Myogel−3D	Gefitinib	0.3	9890.1

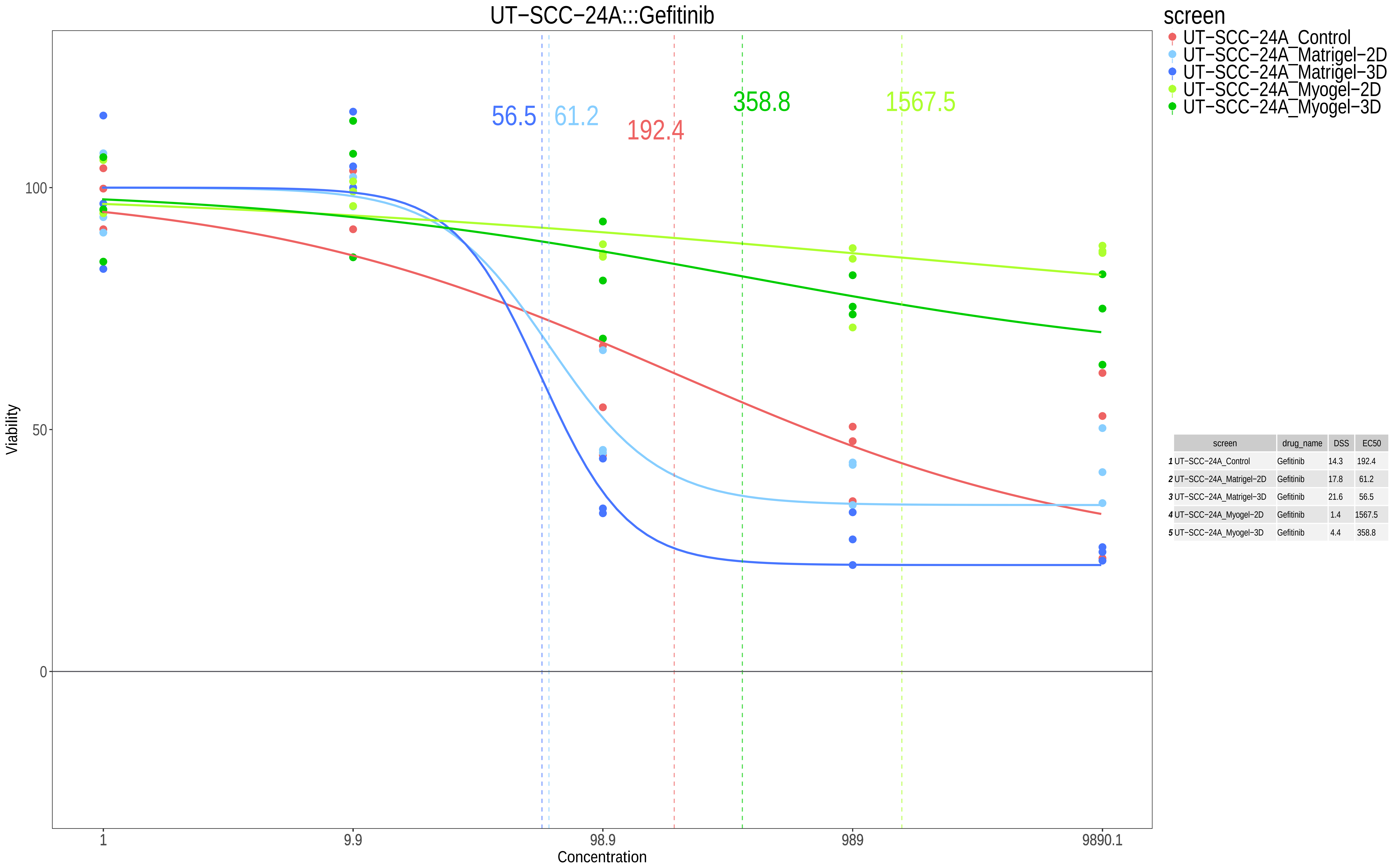

	screen	drug_name	DSS	EC50
1	UT−SCC−24A_Control	Gefitinib	14.3	192.4
2	UT−SCC−24A_Matrigel−2D	Gefitinib	17.8	61.2
3	UT−SCC−24A_Matrigel−3D	Gefitinib	21.6	56.5
4	UT−SCC−24A_Myogel−2D	Gefitinib	1.4	1567.5
5	UT−SCC−24A_Myogel−3D	Gefitinib	4.4	358.8

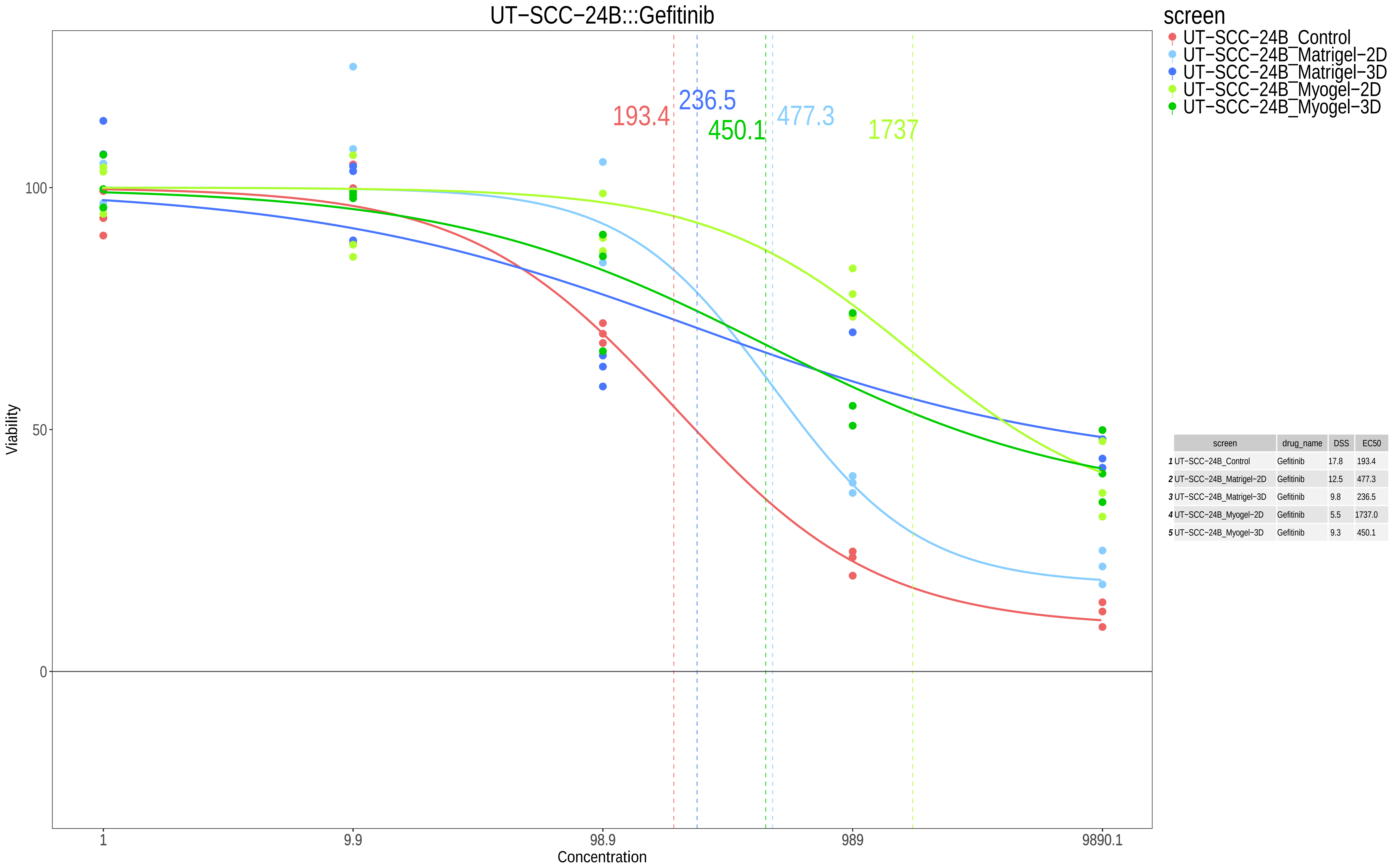

	screen	drug_name	DSS	EC50
1	UT−SCC−24B_Control	Gefitinib	17.8	193.4
2	UT−SCC−24B_Matrigel−2D	Gefitinib	12.5	477.3
3	UT−SCC−24B_Matrigel−3D	Gefitinib	9.8	236.5
4	UT−SCC−24B_Myogel−2D	Gefitinib	5.5	1737.0
5	UT−SCC−24B_Myogel−3D	Gefitinib	9.3	450.1

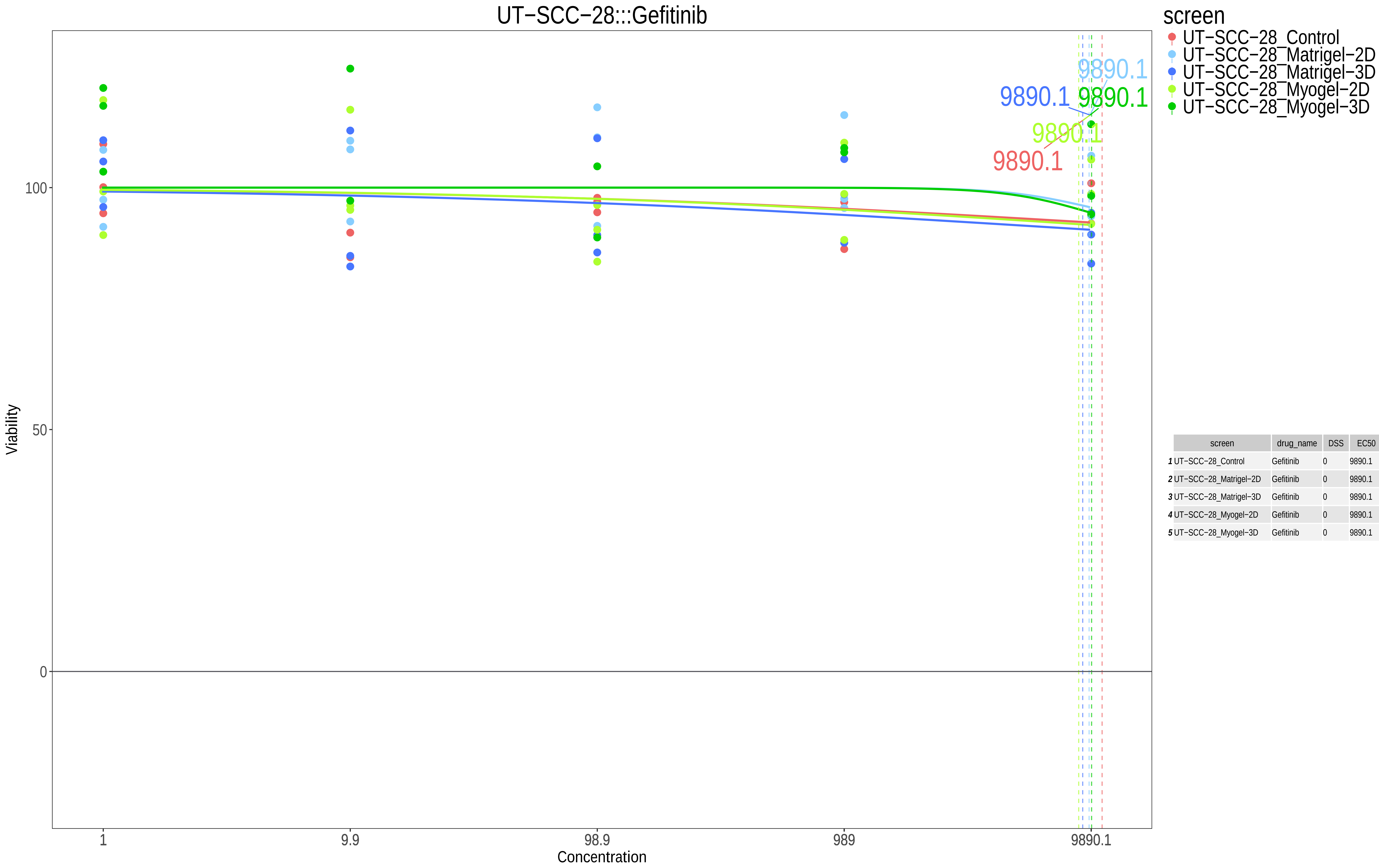

	screen	drug_name	DSS	EC50
1	UT−SCC−28_Control	Gefitinib	0	9890.1
2	UT−SCC−28_Matrigel−2D	Gefitinib	0	9890.1
3	UT−SCC−28_Matrigel−3D	Gefitinib	0	9890.1
4	UT−SCC−28_Myogel−2D	Gefitinib	0	9890.1
5	UT−SCC−28_Myogel−3D	Gefitinib	0	9890.1

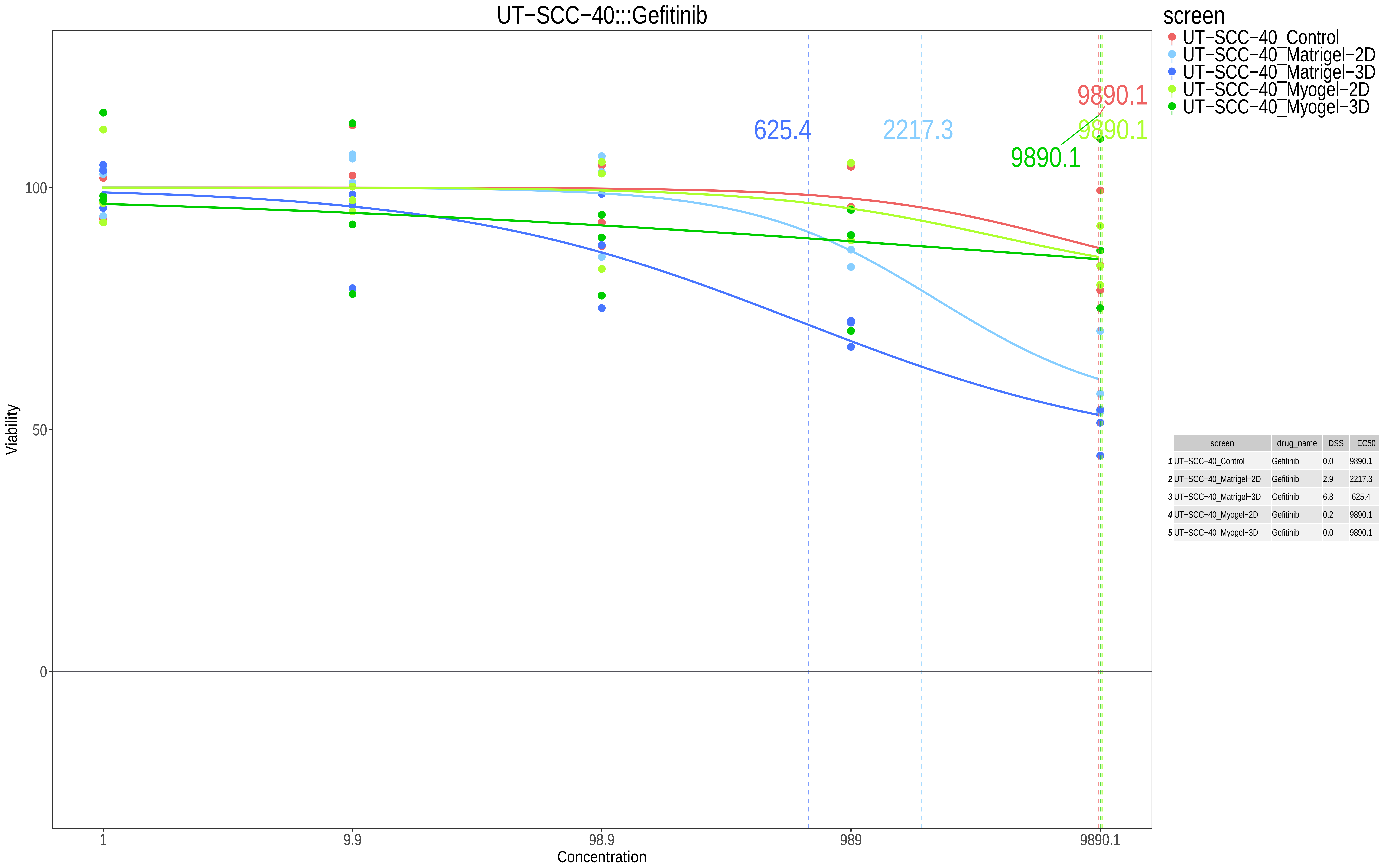

	screen	drug_name	DSS	EC50
1	UT−SCC−40_Control	Gefitinib	0.0	9890.1
2	UT−SCC−40_Matrigel−2D	Gefitinib	2.9	2217.3
3	UT−SCC−40_Matrigel−3D	Gefitinib	6.8	625.4
4	UT−SCC−40_Myogel−2D	Gefitinib	0.2	9890.1
5	UT−SCC−40_Myogel−3D	Gefitinib	0.0	9890.1

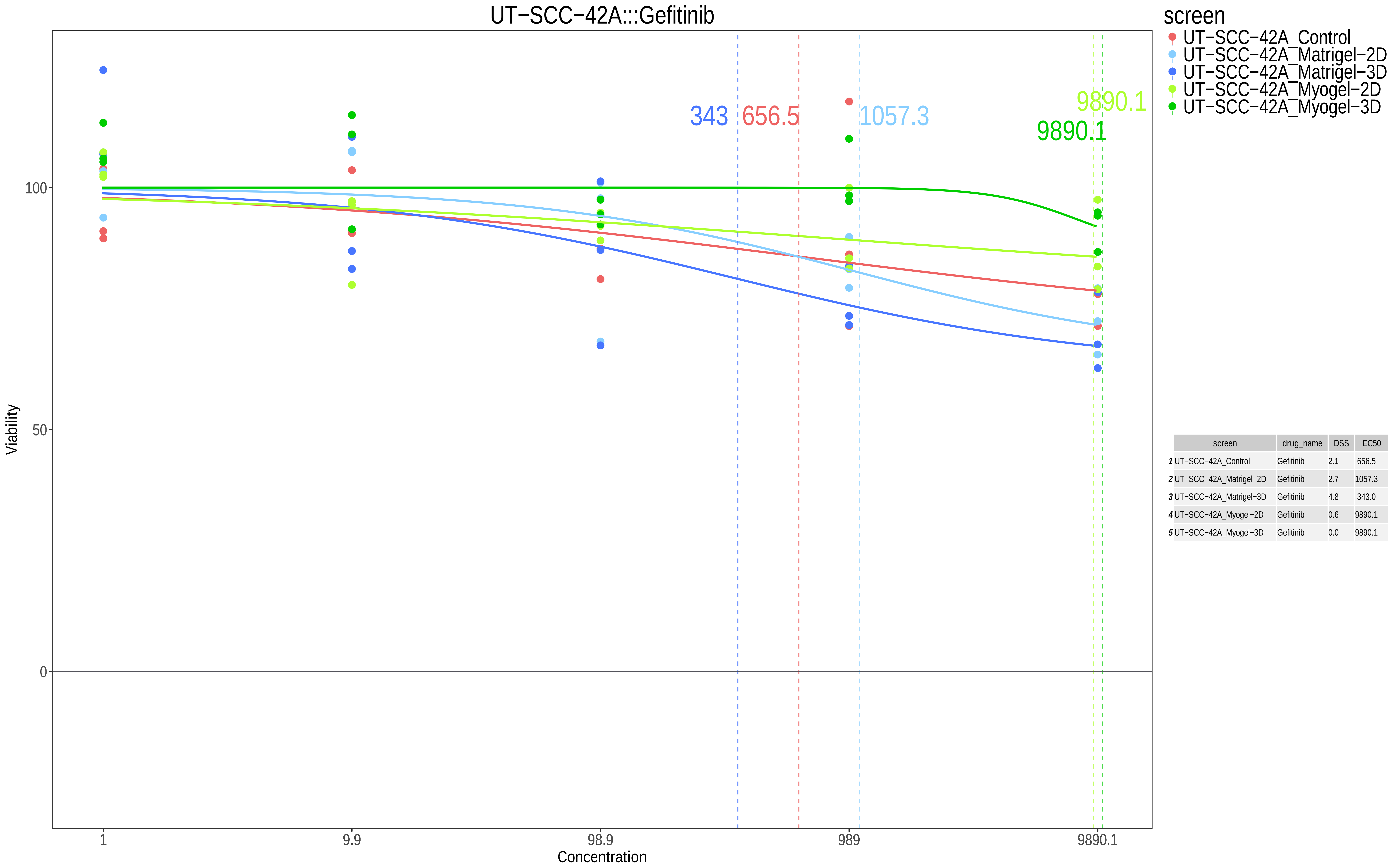

	screen	drug_name	DSS	EC50
1	UT−SCC−42A_Control	Gefitinib	2.1	656.5
2	UT−SCC−42A_Matrigel−2D	Gefitinib	2.7	1057.3
3	UT−SCC−42A_Matrigel−3D	Gefitinib	4.8	343.0
4	UT−SCC−42A_Myogel−2D	Gefitinib	0.6	9890.1
5	UT−SCC−42A_Myogel−3D	Gefitinib	0.0	9890.1

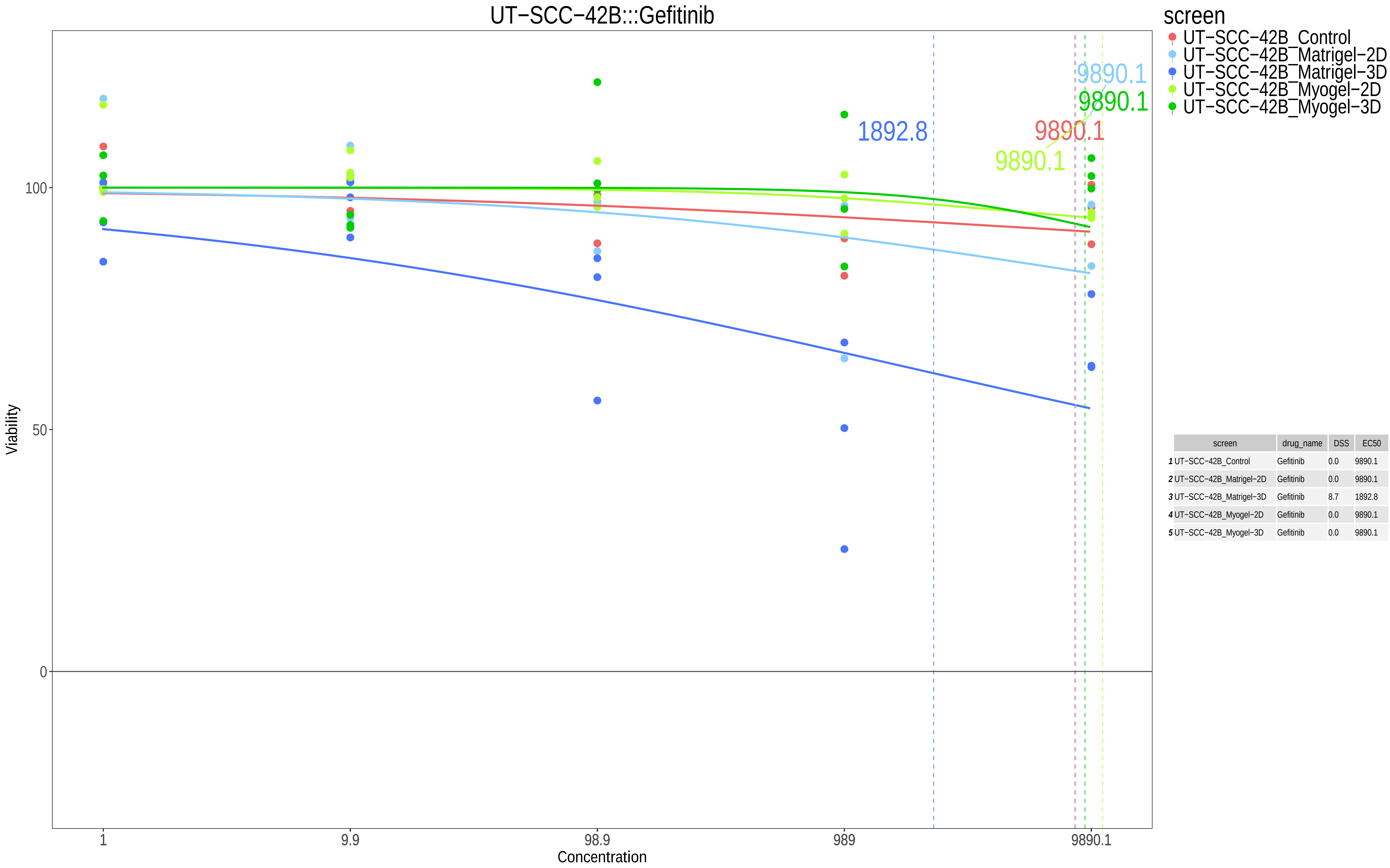

	screen	drug_name	DSS	EC50
1	UT−SCC−42B_Control	Gefitinib	0.0	9890.1
2	UT−SCC−42B_Matrigel−2D	Gefitinib	0.0	9890.1
3	UT−SCC−42B_Matrigel−3D	Gefitinib	8.7	1892.8
4	UT−SCC−42B_Myogel−2D	Gefitinib	0.0	9890.1
5	UT−SCC−42B_Myogel−3D	Gefitinib	0.0	9890.1

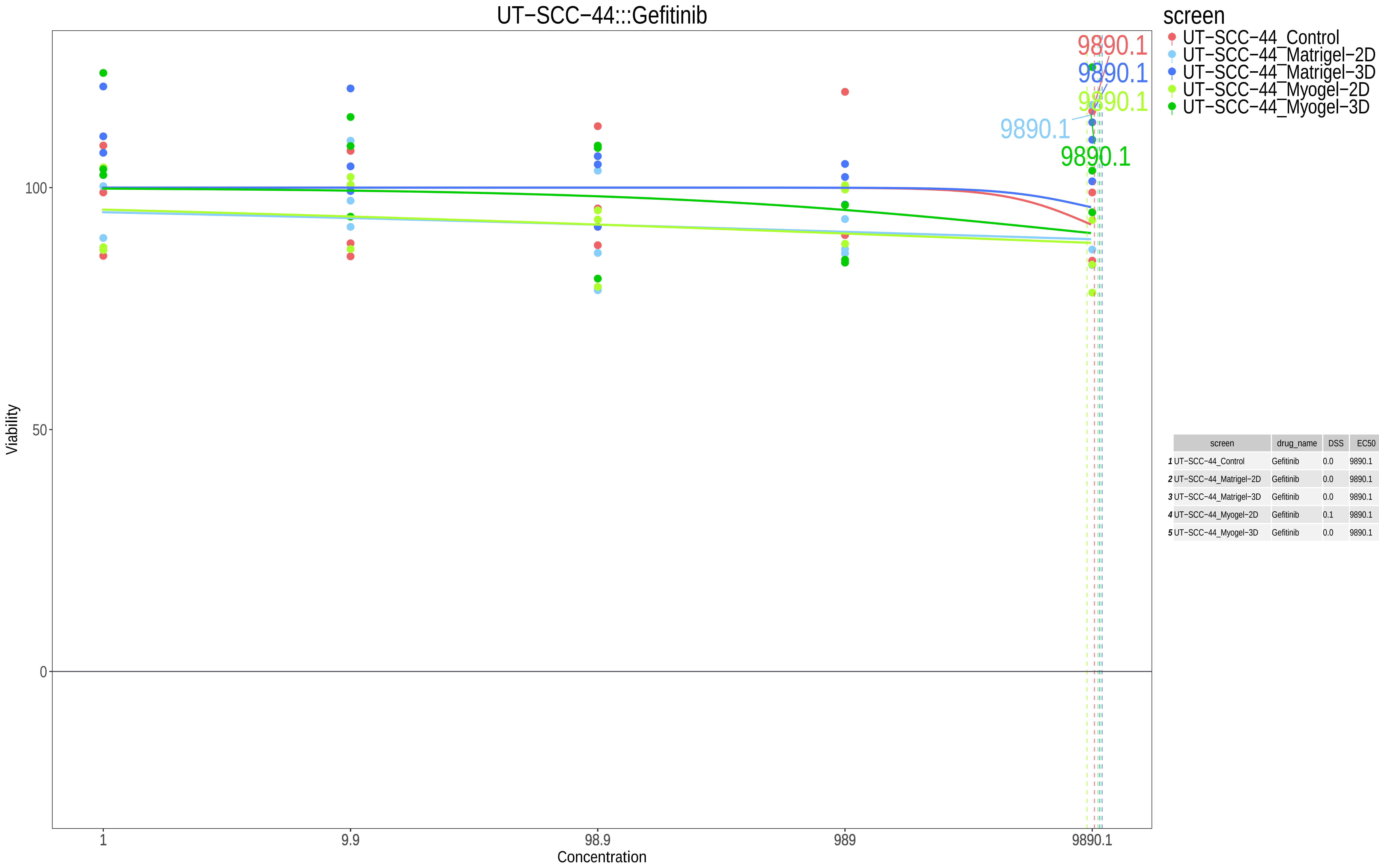

	screen	drug_name	DSS	EC50
1	UT−SCC−44_Control	Gefitinib	0.0	9890.1
2	UT−SCC−44_Matrigel−2D	Gefitinib	0.0	9890.1
3	UT−SCC−44_Matrigel−3D	Gefitinib	0.0	9890.1
4	UT−SCC−44_Myogel−2D	Gefitinib	0.1	9890.1
5	UT−SCC−44_Myogel−3D	Gefitinib	0.0	9890.1

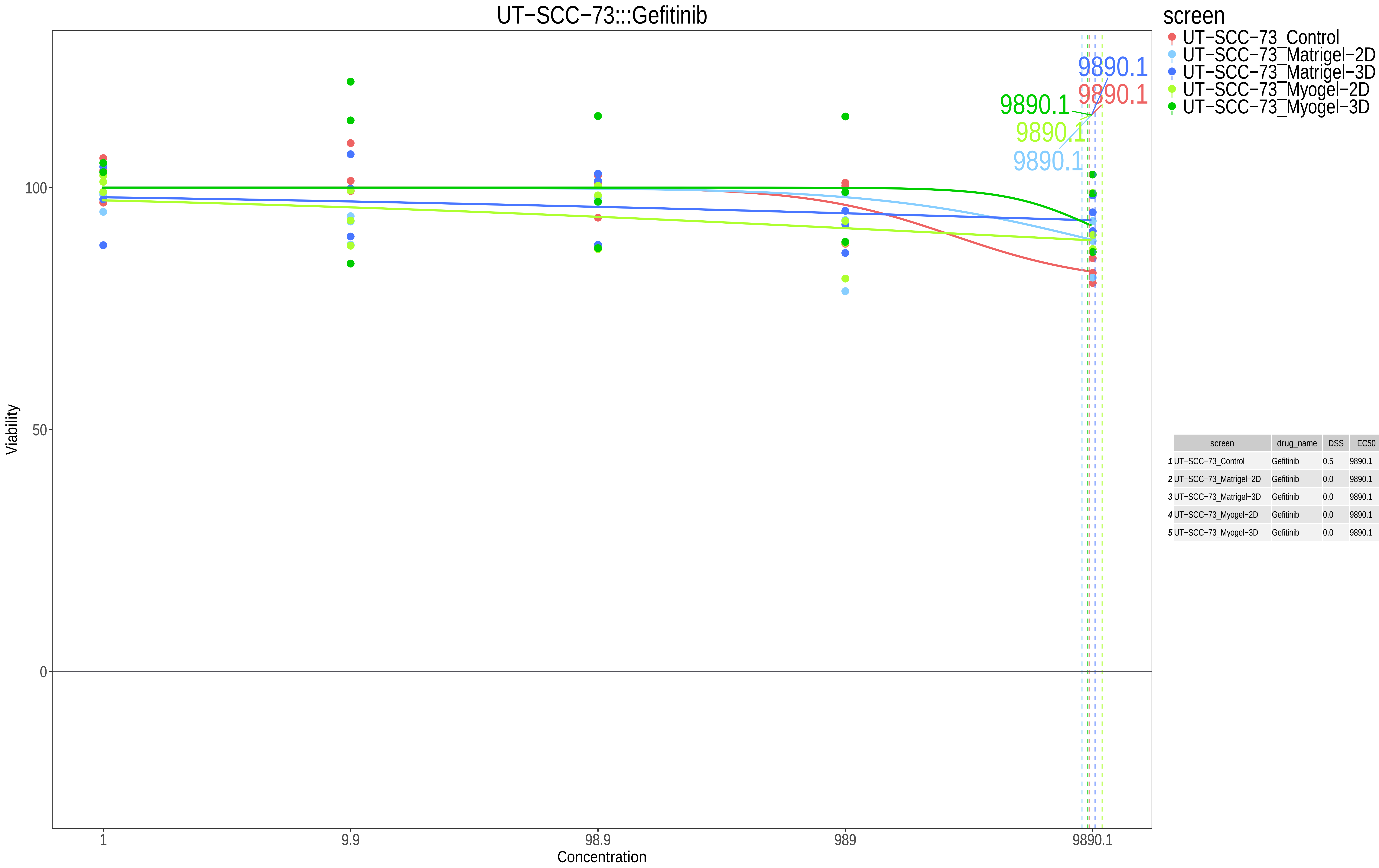

	screen	drug_name	DSS	EC50
1	UT−SCC−73_Control	Gefitinib	0.5	9890.1
2	UT−SCC−73_Matrigel−2D	Gefitinib	0.0	9890.1
3	UT−SCC−73_Matrigel−3D	Gefitinib	0.0	9890.1
4	UT−SCC−73_Myogel−2D	Gefitinib	0.0	9890.1
5	UT−SCC−73_Myogel−3D	Gefitinib	0.0	9890.1

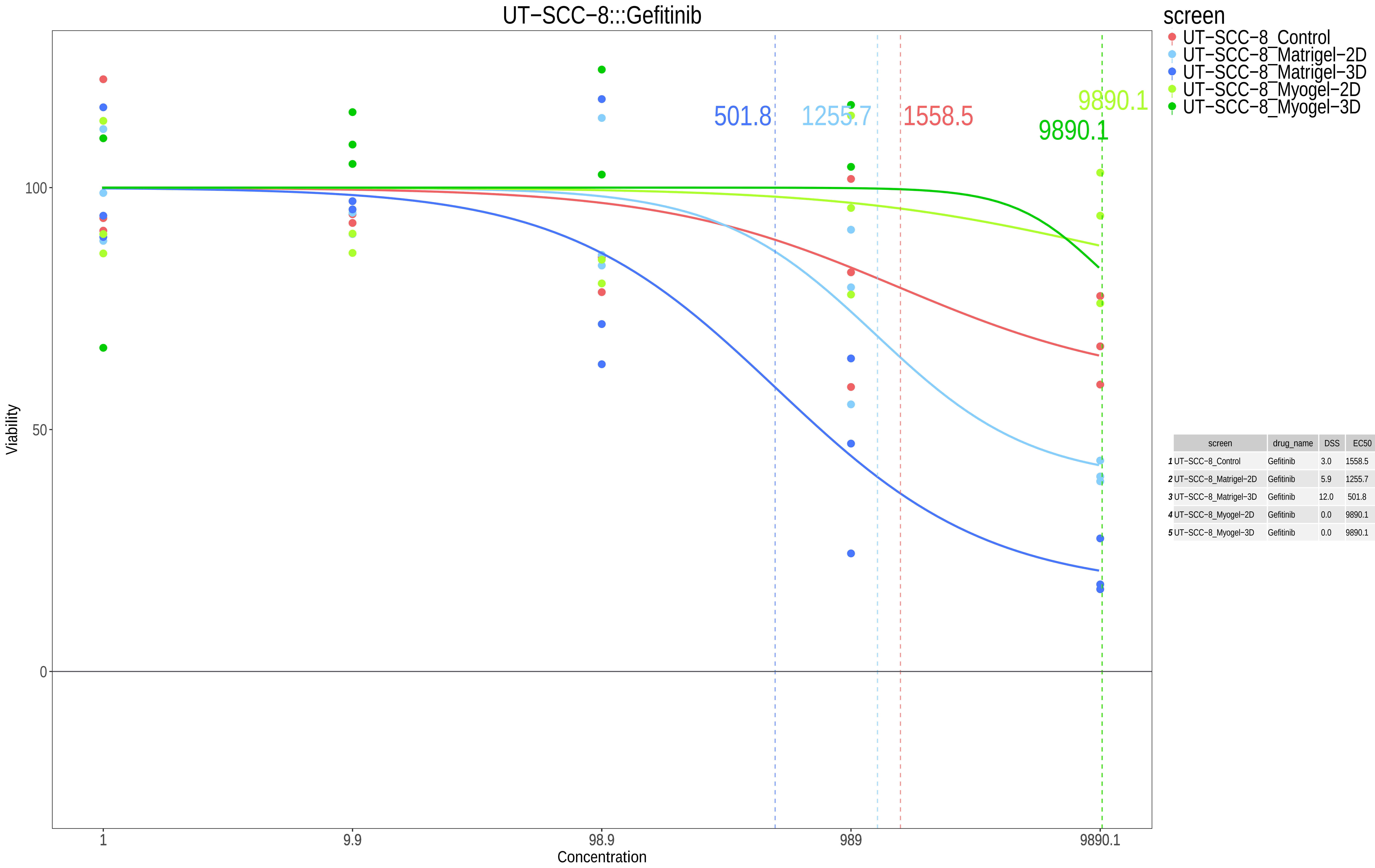

	screen	drug_name	DSS	EC50
1	UT−SCC−8_Control	Gefitinib	3.0	1558.5
2	UT−SCC−8_Matrigel−2D	Gefitinib	5.9	1255.7
3	UT−SCC−8_Matrigel−3D	Gefitinib	12.0	501.8
4	UT−SCC−8_Myogel−2D	Gefitinib	0.0	9890.1
5	UT−SCC−8_Myogel−3D	Gefitinib	0.0	9890.1

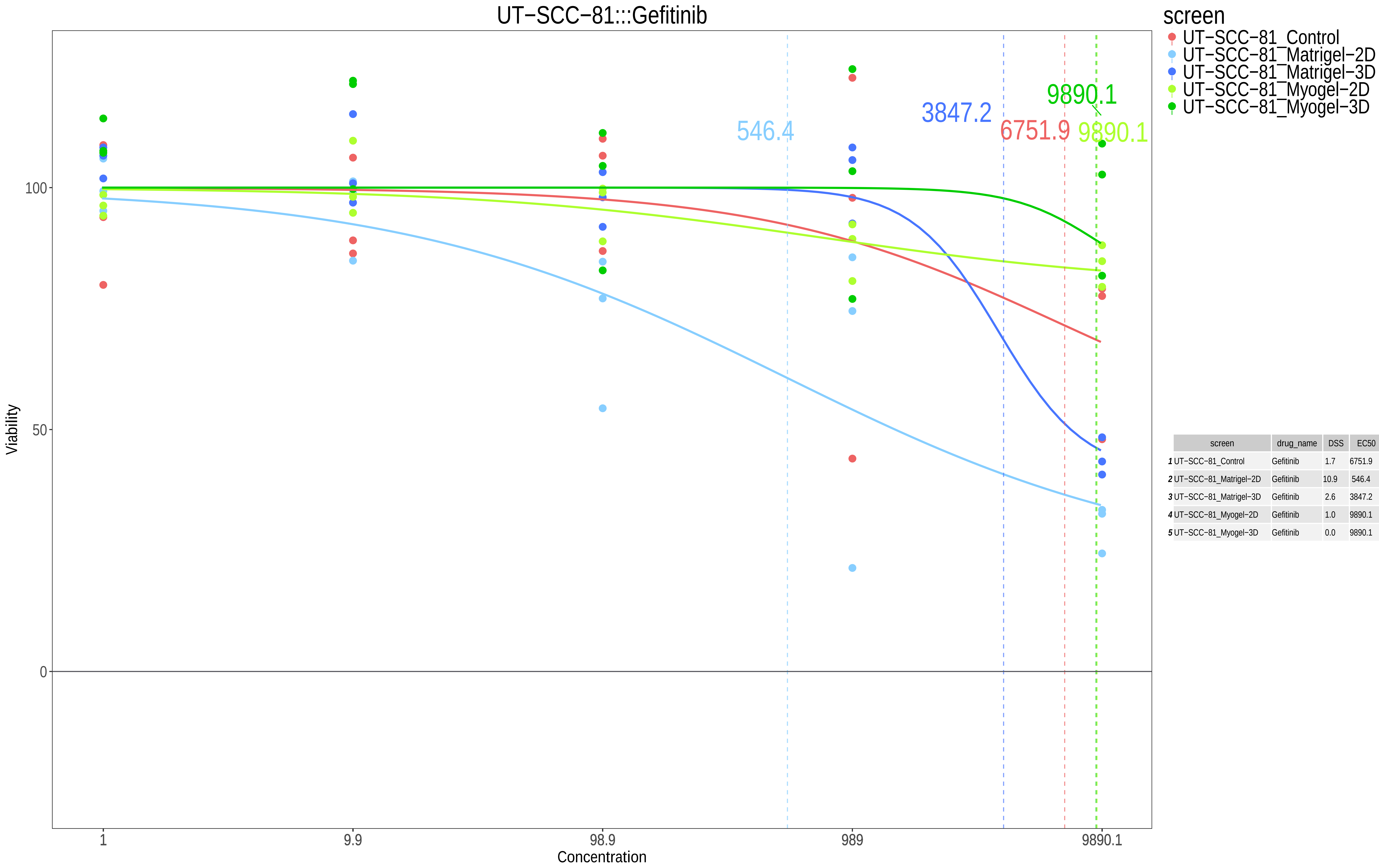

	screen	drug_name	DSS	EC50
1	UT−SCC−81_Control	Gefitinib	1.7	6751.9
2	UT−SCC−81_Matrigel−2D	Gefitinib	10.9	546.4
3	UT−SCC−81_Matrigel−3D	Gefitinib	2.6	3847.2
4	UT−SCC−81_Myogel−2D	Gefitinib	1.0	9890.1
5	UT−SCC−81_Myogel−3D	Gefitinib	0.0	9890.1

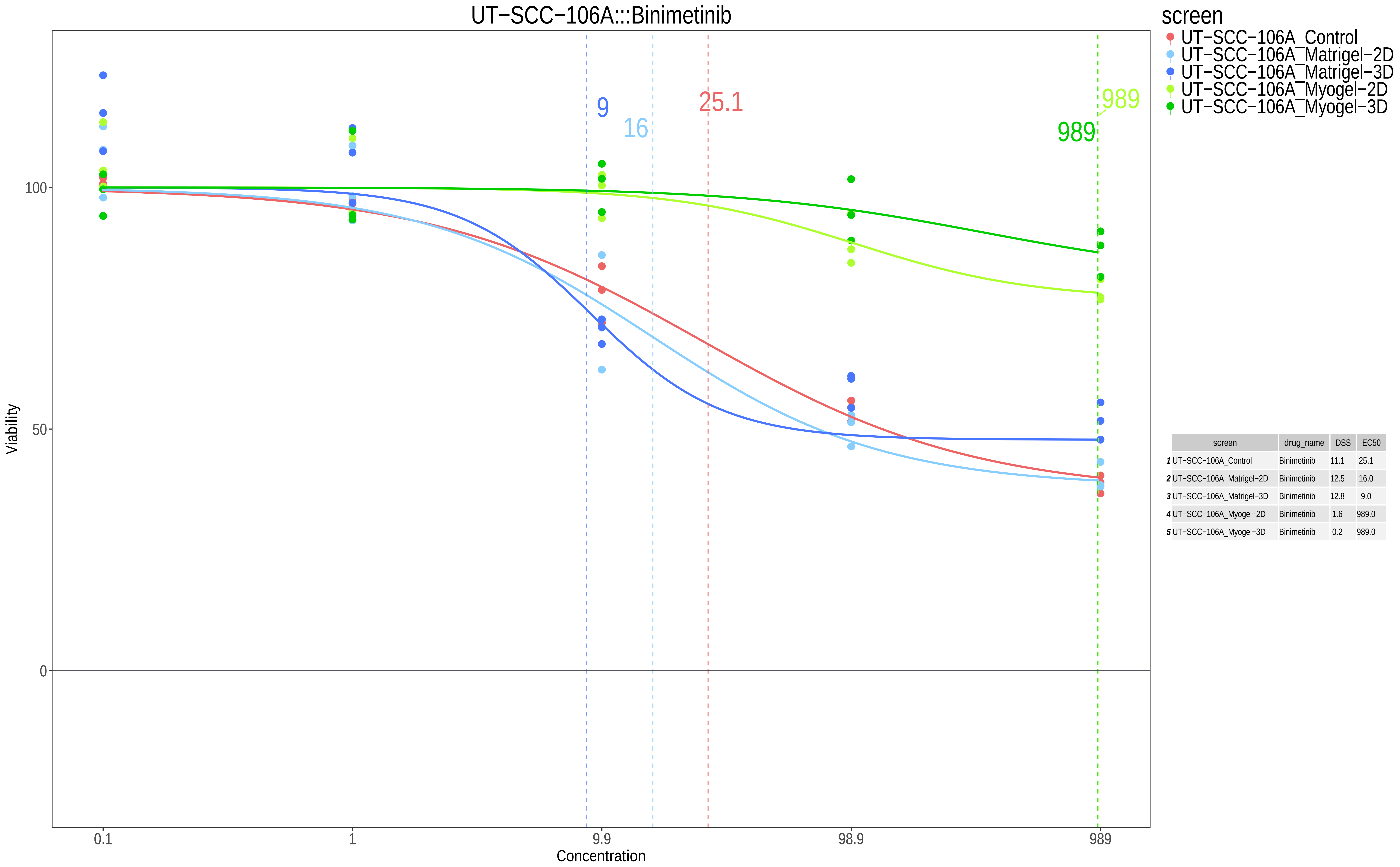

	screen	drug_name	DSS	EC50
1	UT−SCC−106A_Control	Binimetinib	11.1	25.1
2	UT−SCC−106A_Matrigel−2D	Binimetinib	12.5	16.0
3	UT−SCC−106A_Matrigel−3D	Binimetinib	12.8	9.0
4	UT−SCC−106A_Myogel−2D	Binimetinib	1.6	989.0
5	UT−SCC−106A_Myogel−3D	Binimetinib	0.2	989.0

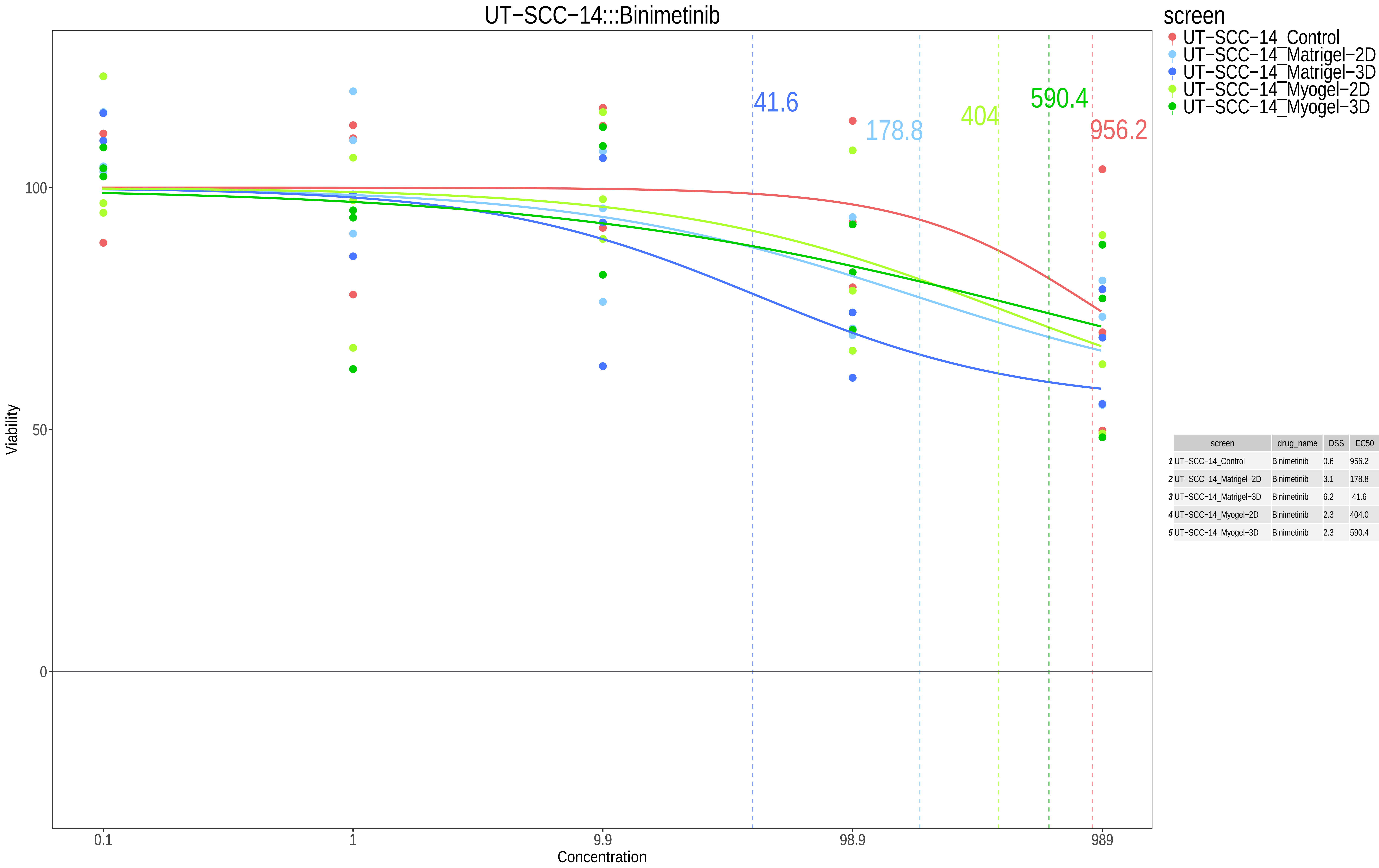

	screen	drug_name	DSS	EC50
1	UT−SCC−14_Control	Binimetinib	0.6	956.2
2	UT−SCC−14_Matrigel−2D	Binimetinib	3.1	178.8
3	UT−SCC−14_Matrigel−3D	Binimetinib	6.2	41.6
4	UT−SCC−14_Myogel−2D	Binimetinib	2.3	404.0
5	UT−SCC−14_Myogel−3D	Binimetinib	2.3	590.4

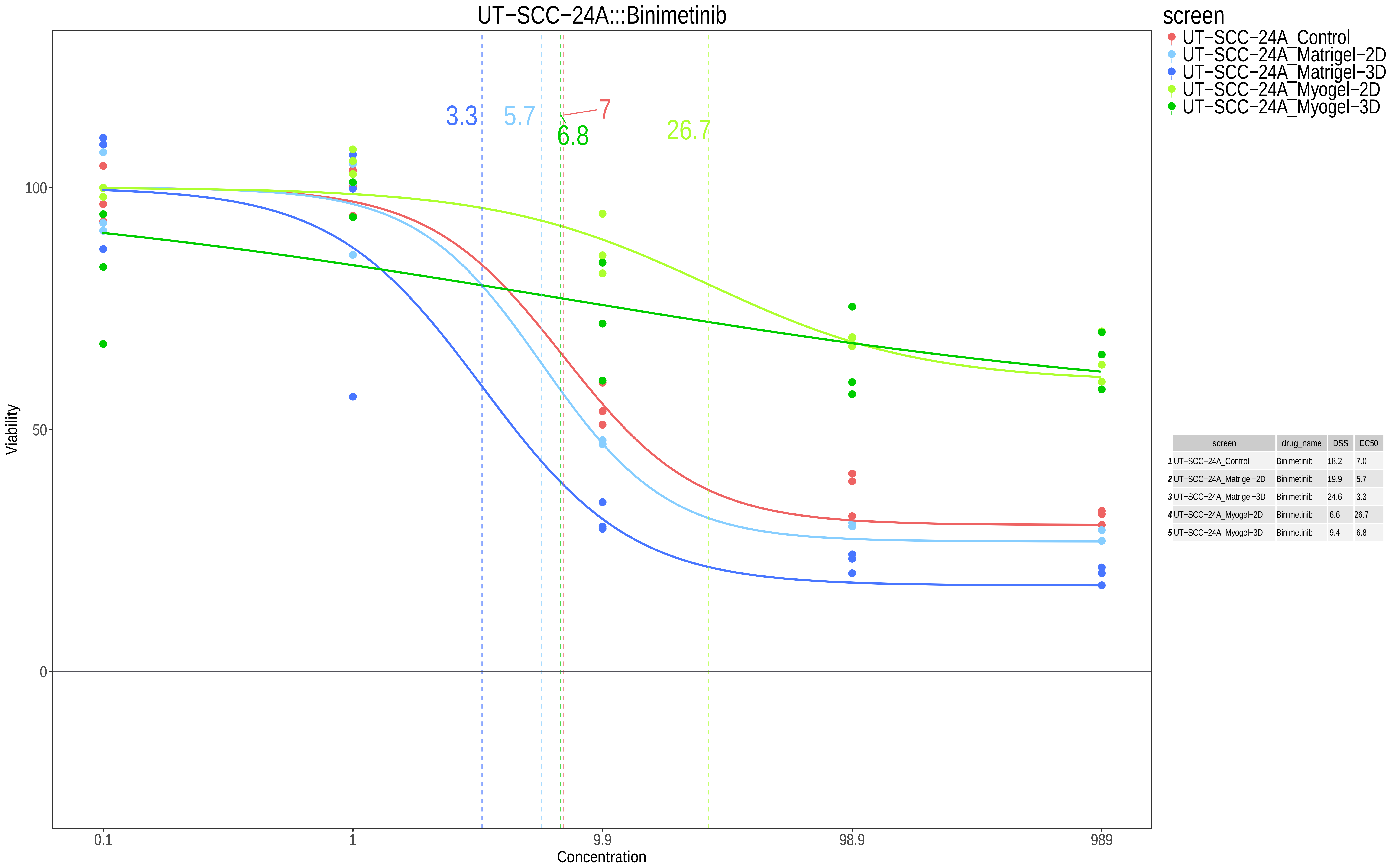

	screen	drug_name	DSS	EC50
1	UT−SCC−24A_Control	Binimetinib	18.2	7.0
2	UT−SCC−24A_Matrigel−2D	Binimetinib	19.9	5.7
3	UT−SCC−24A_Matrigel−3D	Binimetinib	24.6	3.3
4	UT−SCC−24A_Myogel−2D	Binimetinib	6.6	26.7
5	UT−SCC−24A_Myogel−3D	Binimetinib	9.4	6.8

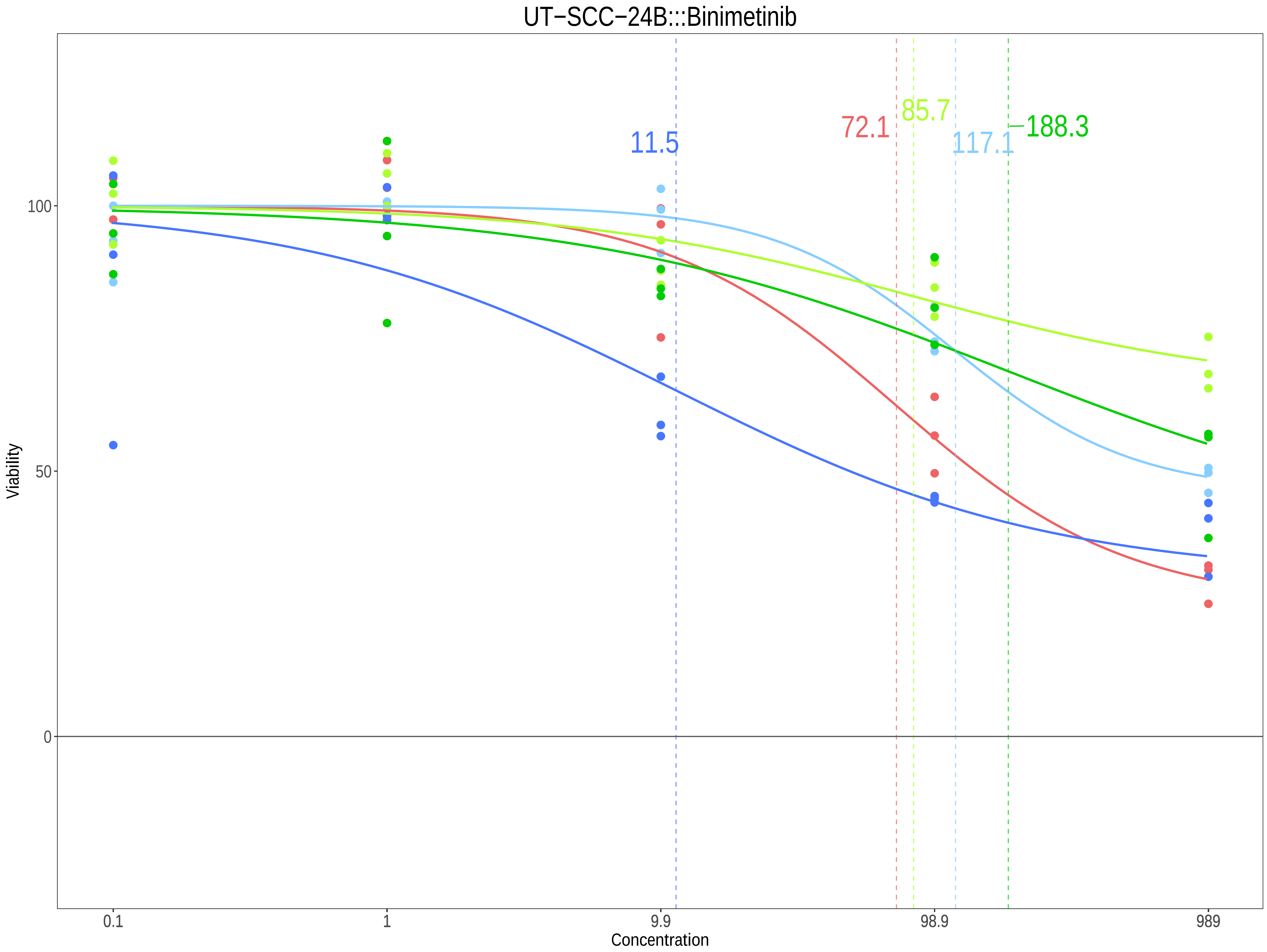

	screen	drug_name	DSS	EC50
1	UT−SCC−24B_Control	Binimetinib	9.4	72.1
2	UT−SCC−24B_Matrigel−2D	Binimetinib	5.4	117.1
3	UT−SCC−24B_Matrigel−3D	Binimetinib	14.9	11.5
4	UT−SCC−24B_Myogel−2D	Binimetinib	3.0	85.7
5	UT−SCC−24B_Myogel−3D	Binimetinib	5.1	188.3

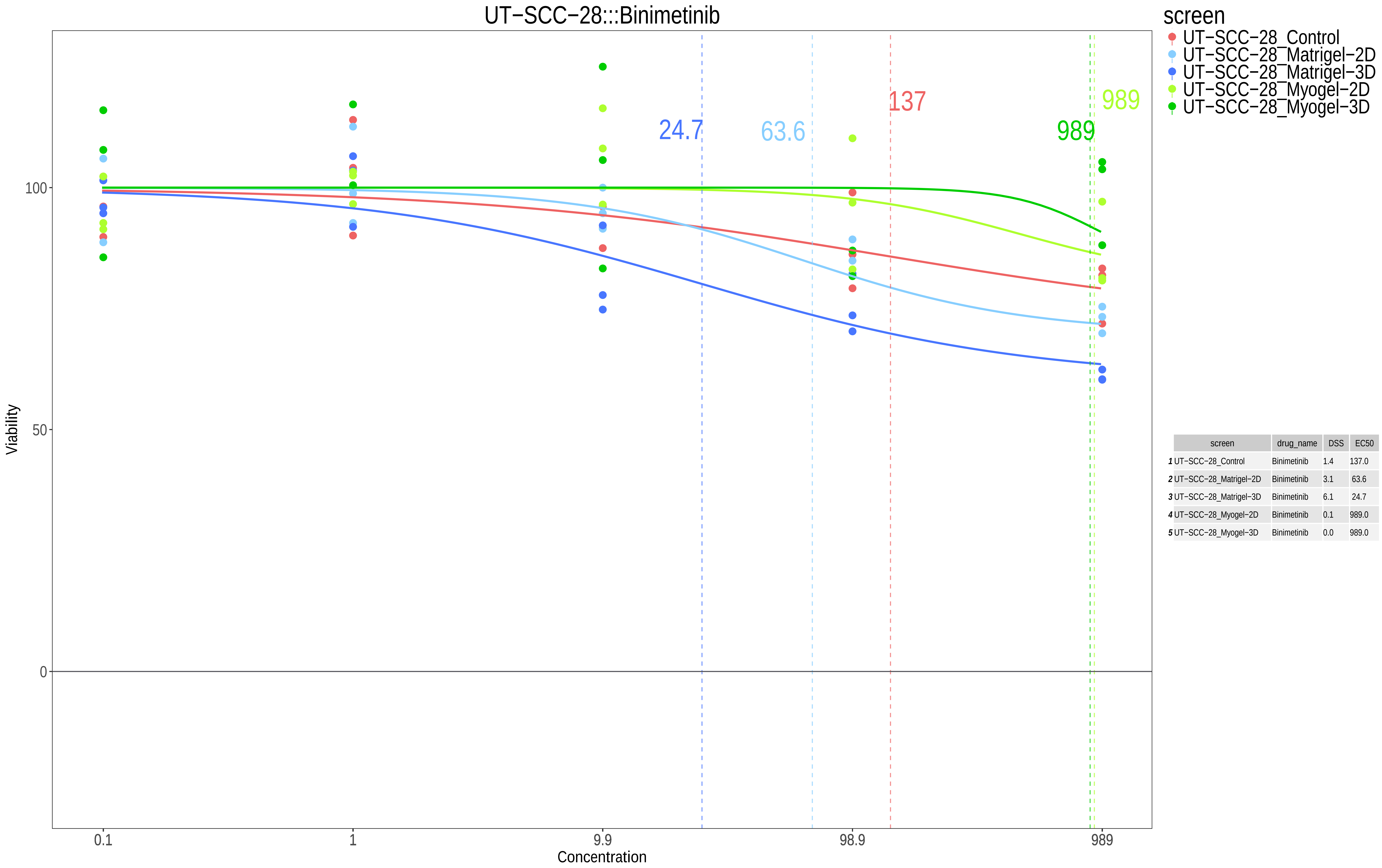

	screen	drug_name	DSS	EC50
1	UT−SCC−28_Control	Binimetinib	1.4	137.0
2	UT−SCC−28_Matrigel−2D	Binimetinib	3.1	63.6
3	UT−SCC−28_Matrigel−3D	Binimetinib	6.1	24.7
4	UT−SCC−28_Myogel−2D	Binimetinib	0.1	989.0
5	UT−SCC−28_Myogel−3D	Binimetinib	0.0	989.0

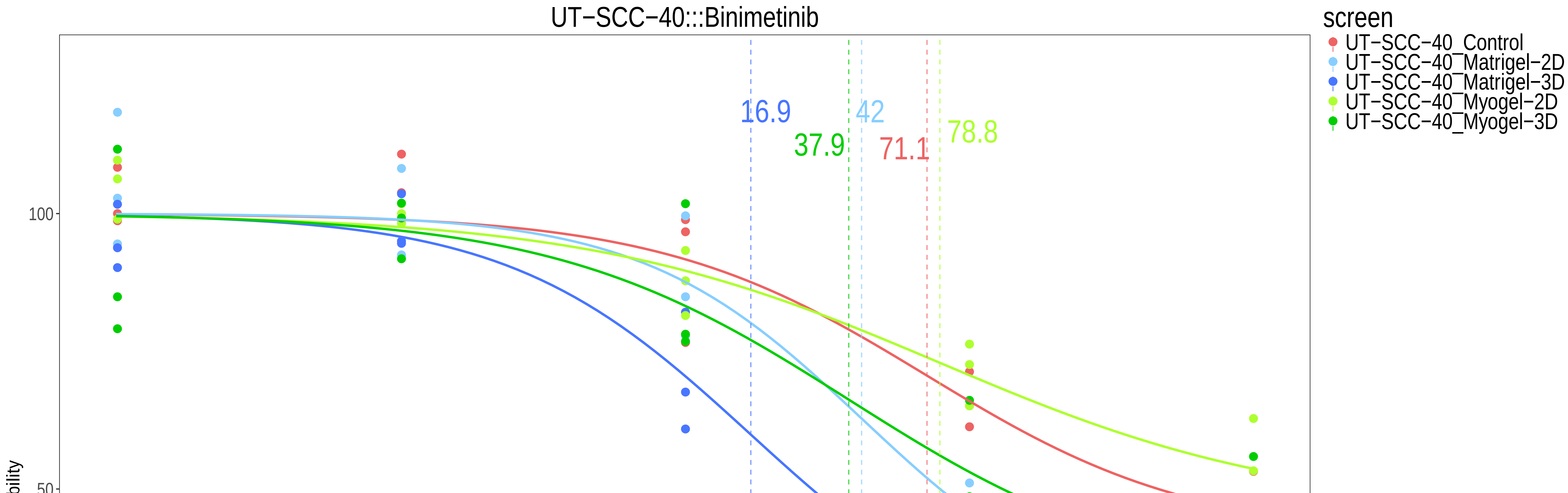

	screen	drug_name	DSS	EC50
1	UT−SCC−40_Control	Binimetinib	7.2	71.1
2	UT−SCC−40_Matrigel−2D	Binimetinib	11.6	42.0
3	UT−SCC−40_Matrigel−3D	Binimetinib	16.3	16.9
4	UT−SCC−40_Myogel−2D	Binimetinib	6.1	78.8
5	UT−SCC−40_Myogel−3D	Binimetinib	10.5	37.9

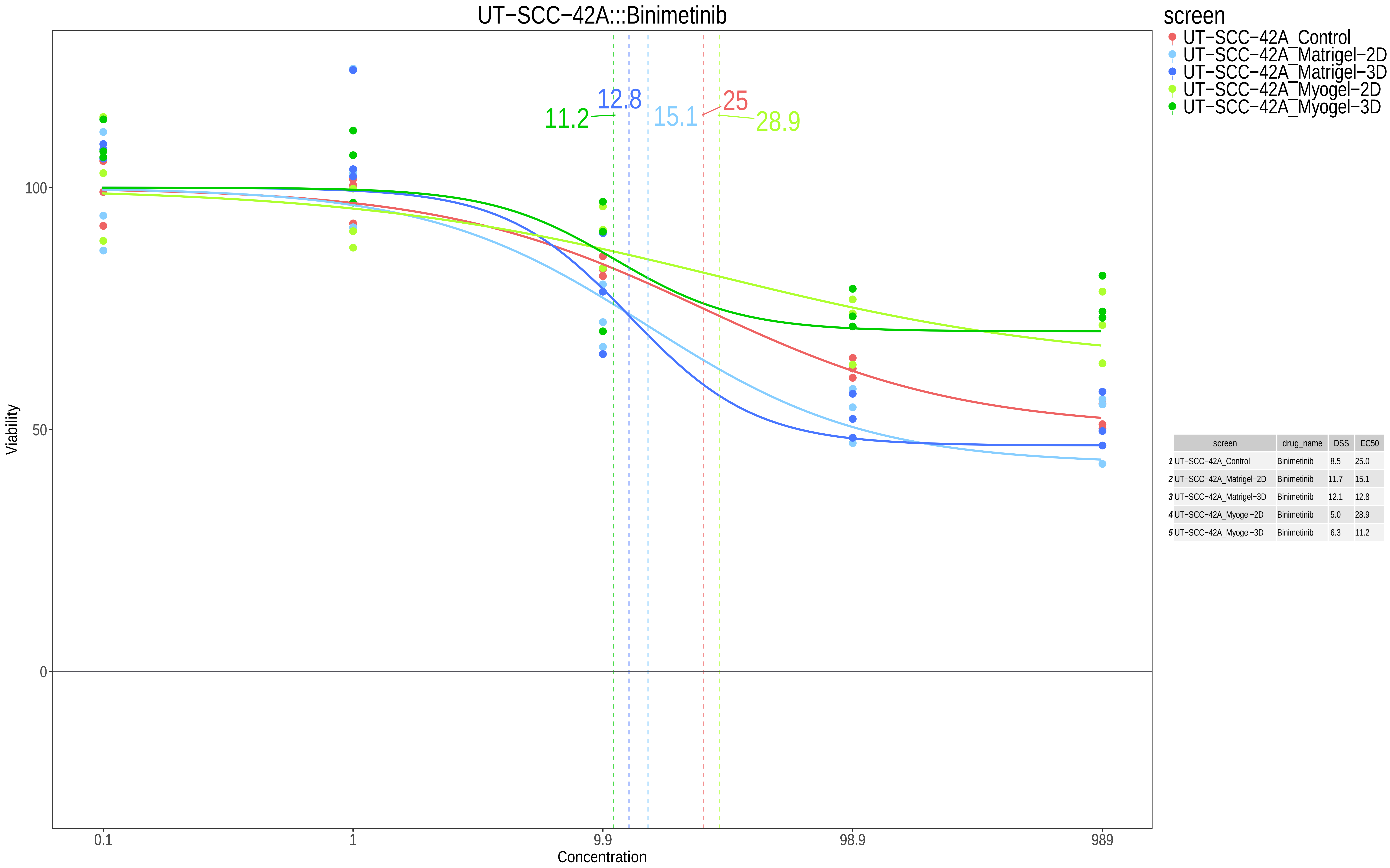

	screen	drug_name	DSS	EC50
1	UT−SCC−42A_Control	Binimetinib	8.5	25.0
2	UT−SCC−42A_Matrigel−2D	Binimetinib	11.7	15.1
3	UT−SCC−42A_Matrigel−3D	Binimetinib	12.1	12.8
4	UT−SCC−42A_Myogel−2D	Binimetinib	5.0	28.9
5	UT−SCC−42A_Myogel−3D	Binimetinib	6.3	11.2

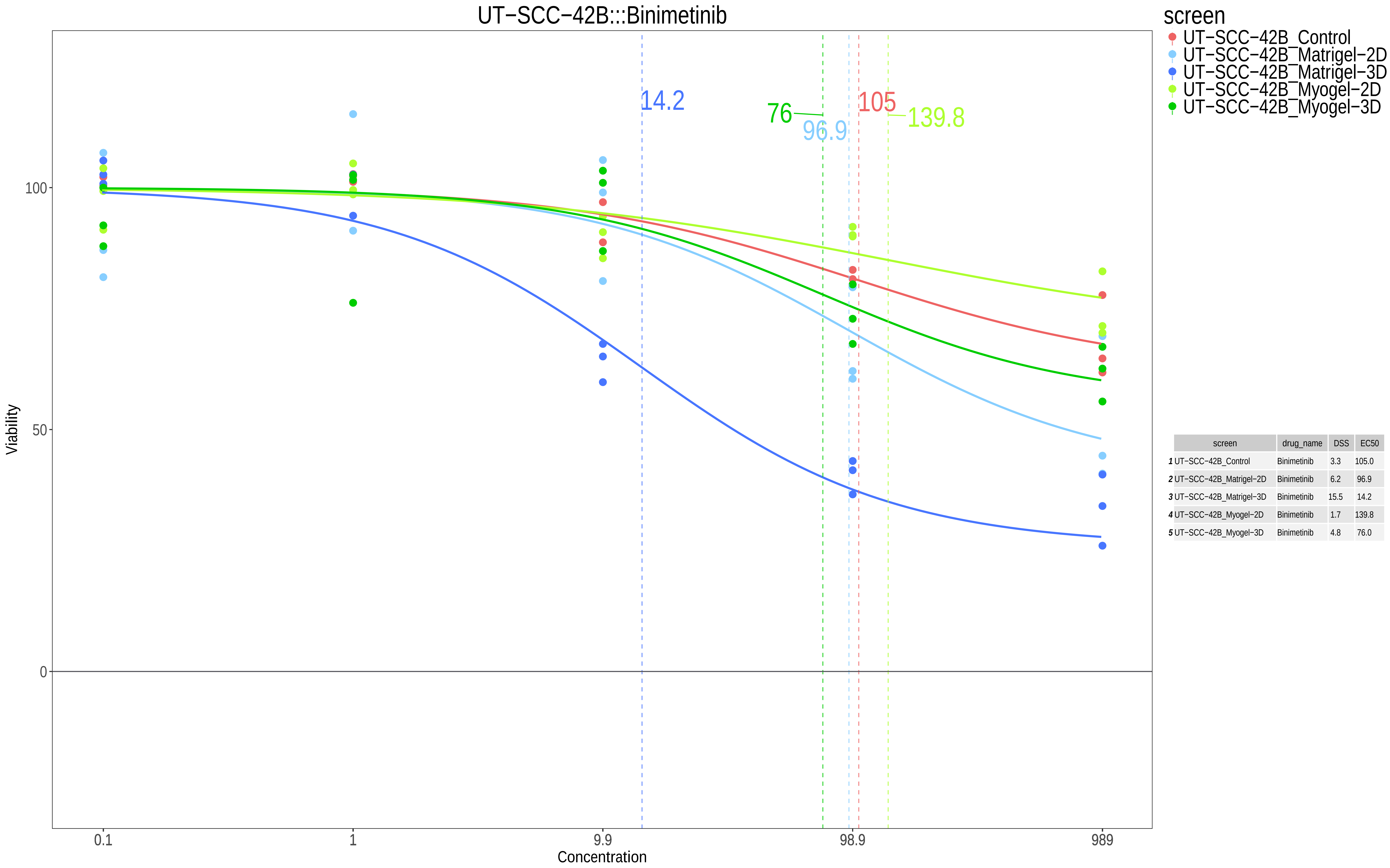

	screen	drug_name	DSS	EC50
1	UT−SCC−42B_Control	Binimetinib	3.3	105.0
2	UT−SCC−42B_Matrigel−2D	Binimetinib	6.2	96.9
3	UT−SCC−42B_Matrigel−3D	Binimetinib	15.5	14.2
4	UT−SCC−42B_Myogel−2D	Binimetinib	1.7	139.8
5	UT−SCC−42B_Myogel−3D	Binimetinib	4.8	76.0

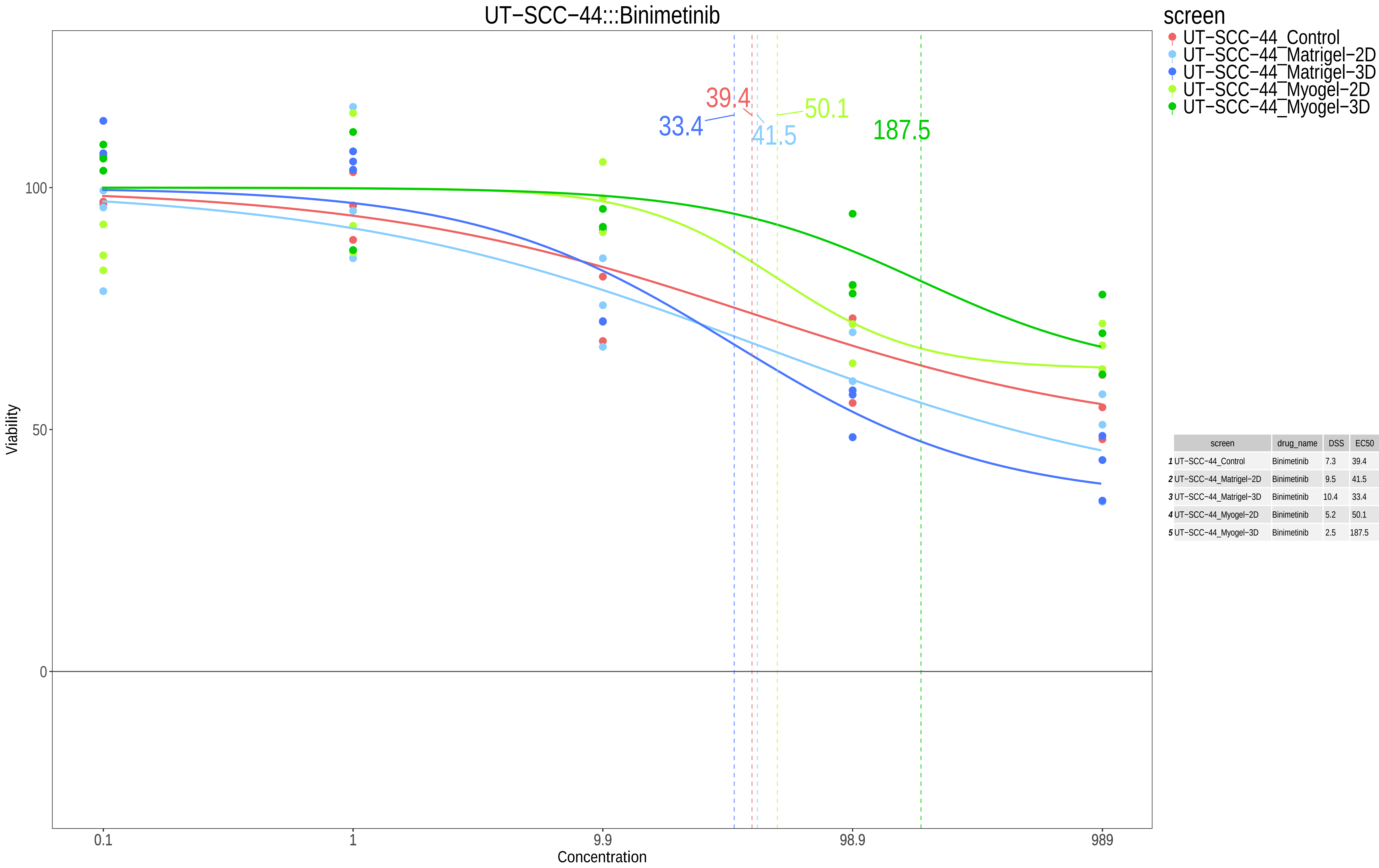

	screen	drug_name	DSS	EC50
1	UT−SCC−44_Control	Binimetinib	7.3	39.4
2	UT−SCC−44_Matrigel−2D	Binimetinib	9.5	41.5
3	UT−SCC−44_Matrigel−3D	Binimetinib	10.4	33.4
4	UT−SCC−44_Myogel−2D	Binimetinib	5.2	50.1
5	UT−SCC−44_Myogel−3D	Binimetinib	2.5	187.5

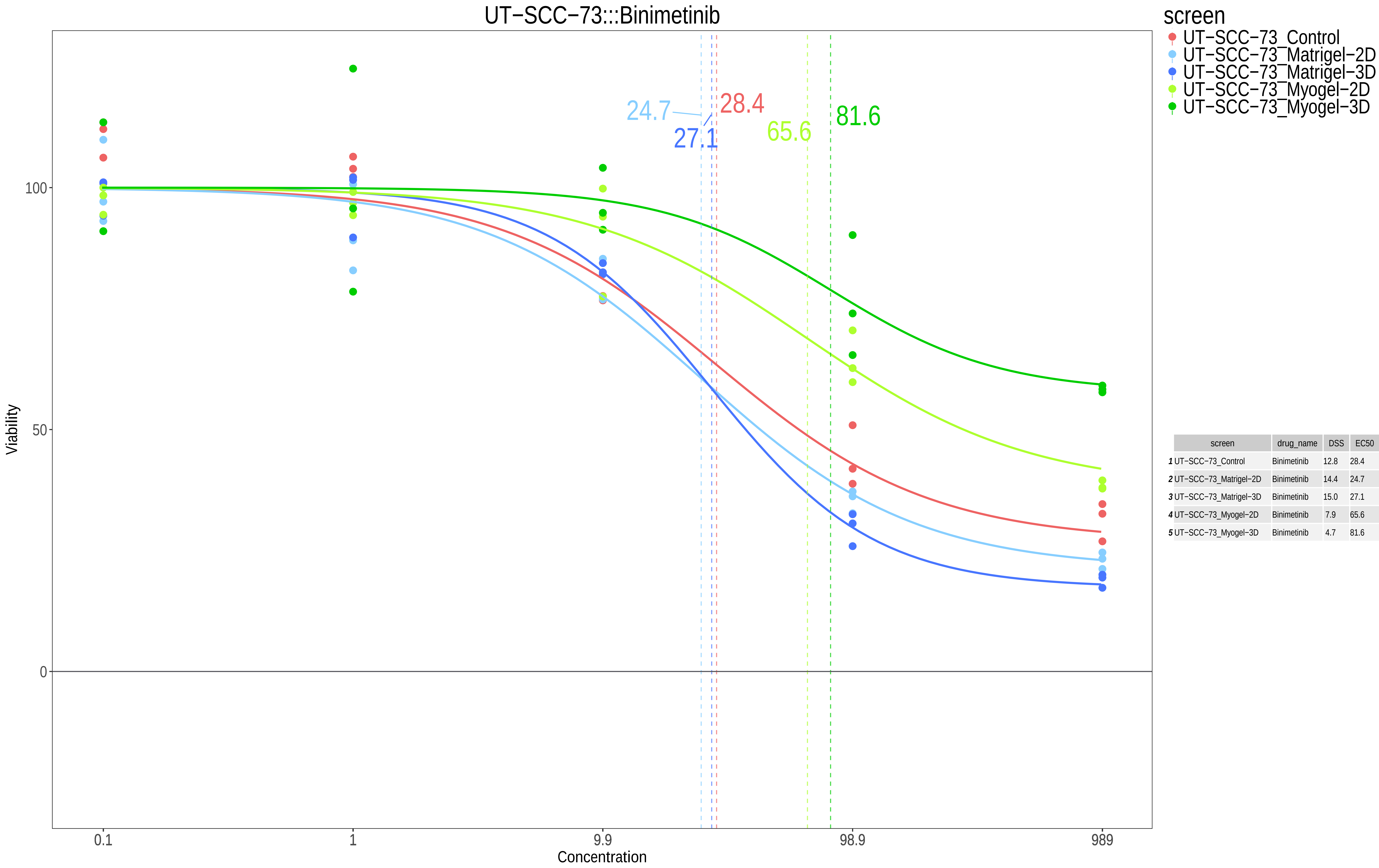

	screen	drug_name	DSS	EC50
1	UT−SCC−73_Control	Binimetinib	12.8	28.4
2	UT−SCC−73_Matrigel−2D	Binimetinib	14.4	24.7
3	UT−SCC−73_Matrigel−3D	Binimetinib	15.0	27.1
4	UT−SCC−73_Myogel−2D	Binimetinib	7.9	65.6
5	UT−SCC−73_Myogel−3D	Binimetinib	4.7	81.6

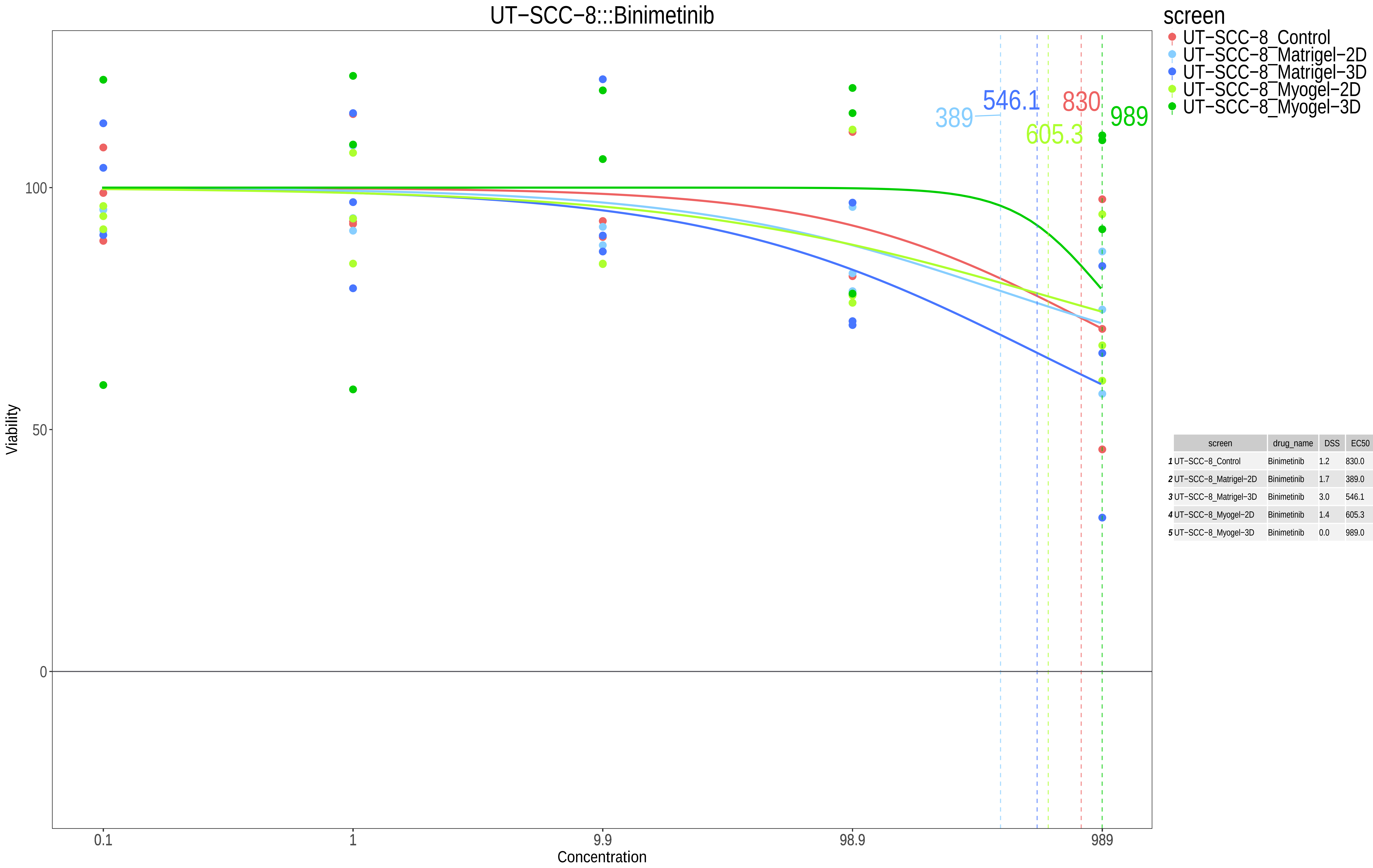

	screen	drug_name	DSS	EC50
1	UT−SCC−8_Control	Binimetinib	1.2	830.0
2	UT−SCC−8_Matrigel−2D	Binimetinib	1.7	389.0
3	UT−SCC−8_Matrigel−3D	Binimetinib	3.0	546.1
4	UT−SCC−8_Myogel−2D	Binimetinib	1.4	605.3
5	UT−SCC−8_Myogel−3D	Binimetinib	0.0	989.0

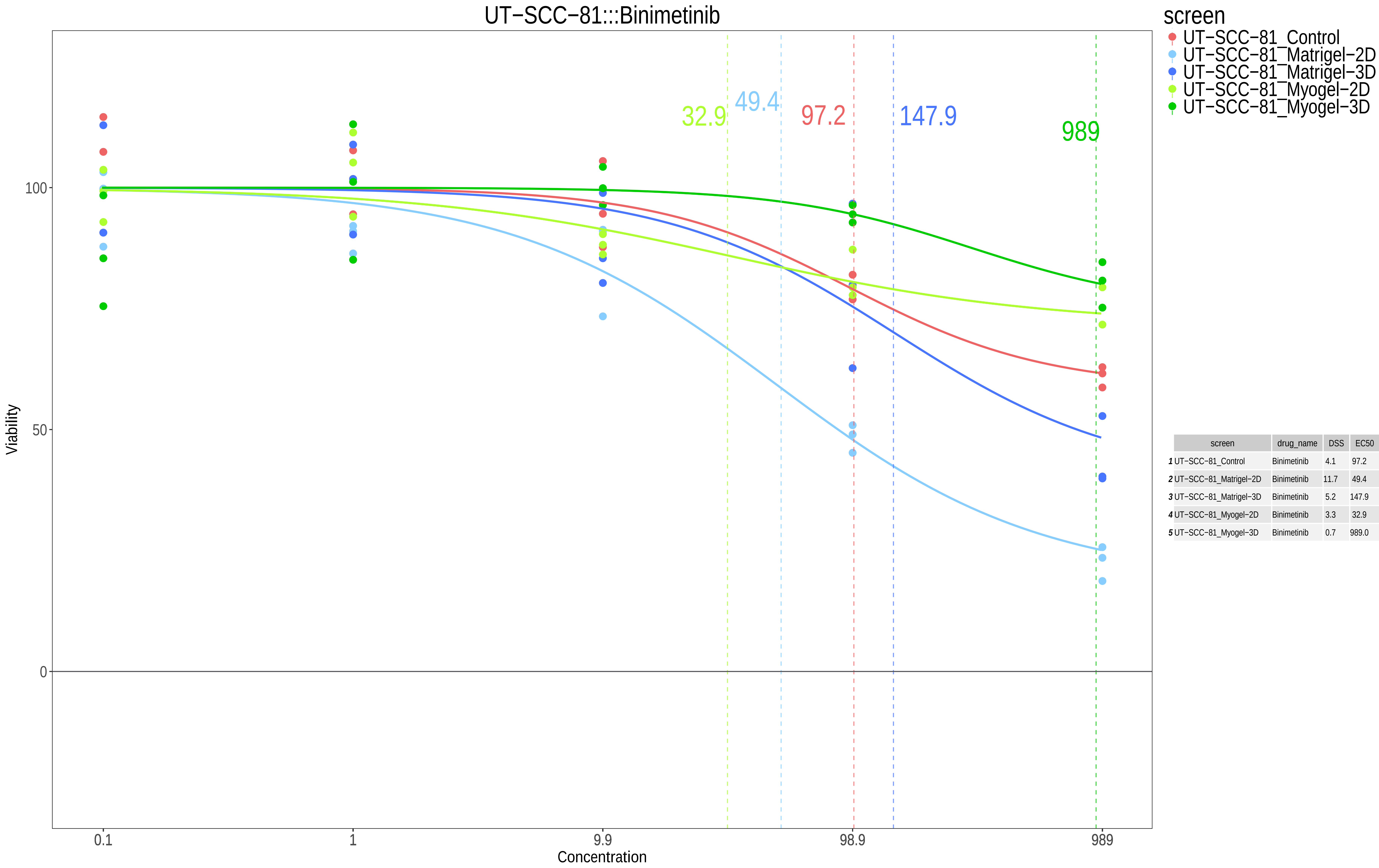

	screen	drug_name	DSS	EC50
1	UT−SCC−81_Control	Binimetinib	4.1	97.2
2	UT−SCC−81_Matrigel−2D	Binimetinib	11.7	49.4
3	UT−SCC−81_Matrigel−3D	Binimetinib	5.2	147.9
4	UT−SCC−81_Myogel−2D	Binimetinib	3.3	32.9
5	UT−SCC−81_Myogel−3D	Binimetinib	0.7	989.0

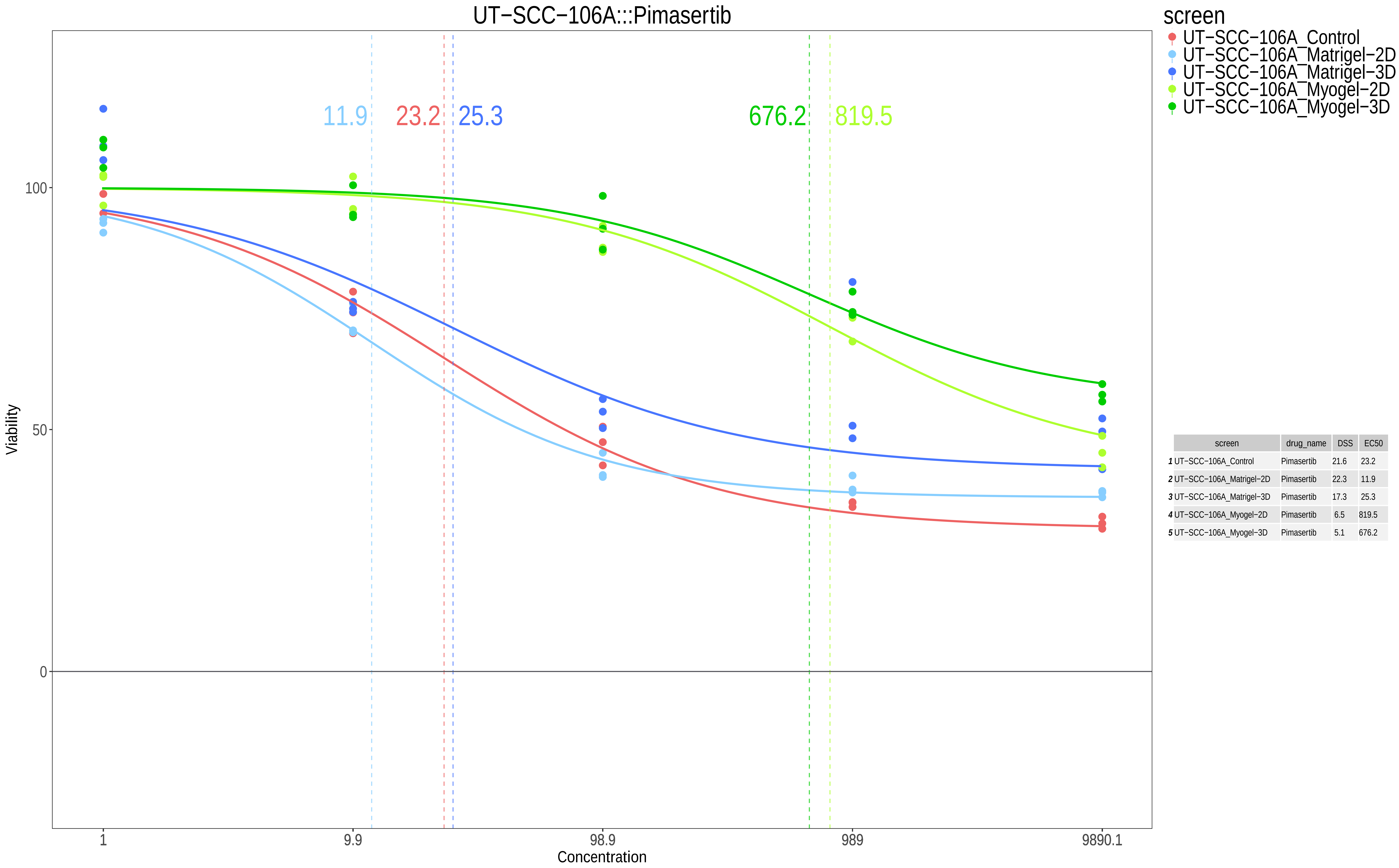

	screen	drug_name	DSS	EC50
1	UT−SCC−106A_Control	Pimasertib	21.6	23.2
2	UT−SCC−106A_Matrigel−2D	Pimasertib	22.3	11.9
3	UT−SCC−106A_Matrigel−3D	Pimasertib	17.3	25.3
4	UT−SCC−106A_Myogel−2D	Pimasertib	6.5	819.5
5	UT−SCC−106A_Myogel−3D	Pimasertib	5.1	676.2

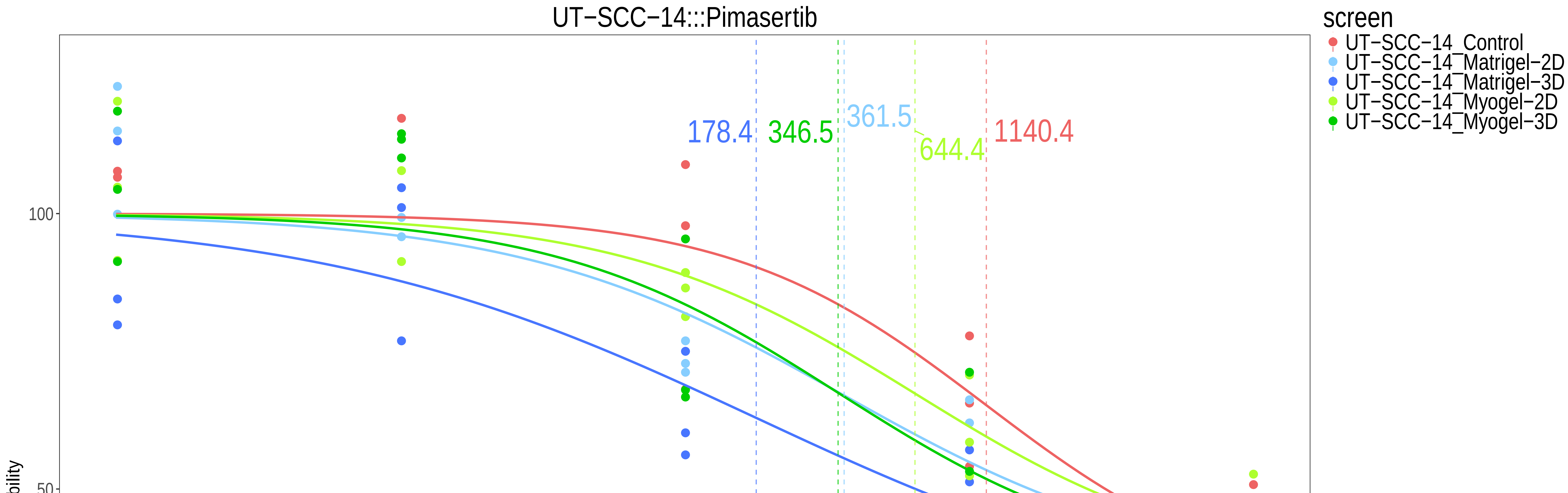

	screen	drug_name	DSS	EC50
1	UT−SCC−14_Control	Pimasertib	7.0	1140.4
2	UT−SCC−14_Matrigel−2D	Pimasertib	10.3	361.5
3	UT−SCC−14_Matrigel−3D	Pimasertib	14.1	178.4
4	UT−SCC−14_Myogel−2D	Pimasertib	8.3	644.4
5	UT−SCC−14_Myogel−3D	Pimasertib	10.4	346.5

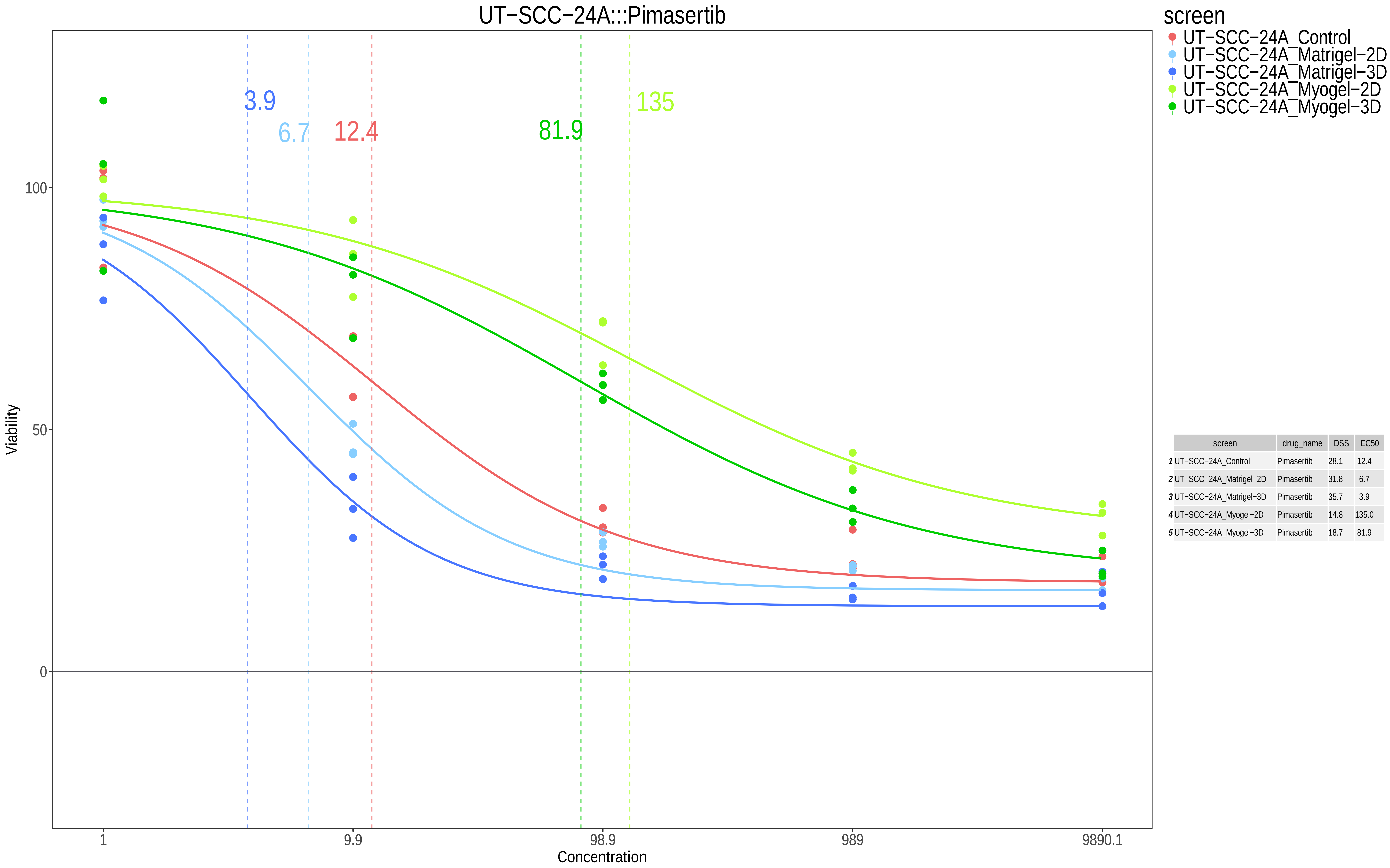

	screen	drug_name	DSS	EC50
1	UT−SCC−24A_Control	Pimasertib	28.1	12.4
2	UT−SCC−24A_Matrigel−2D	Pimasertib	31.8	6.7
3	UT−SCC−24A_Matrigel−3D	Pimasertib	35.7	3.9
4	UT−SCC−24A_Myogel−2D	Pimasertib	14.8	135.0
5	UT−SCC−24A_Myogel−3D	Pimasertib	18.7	81.9

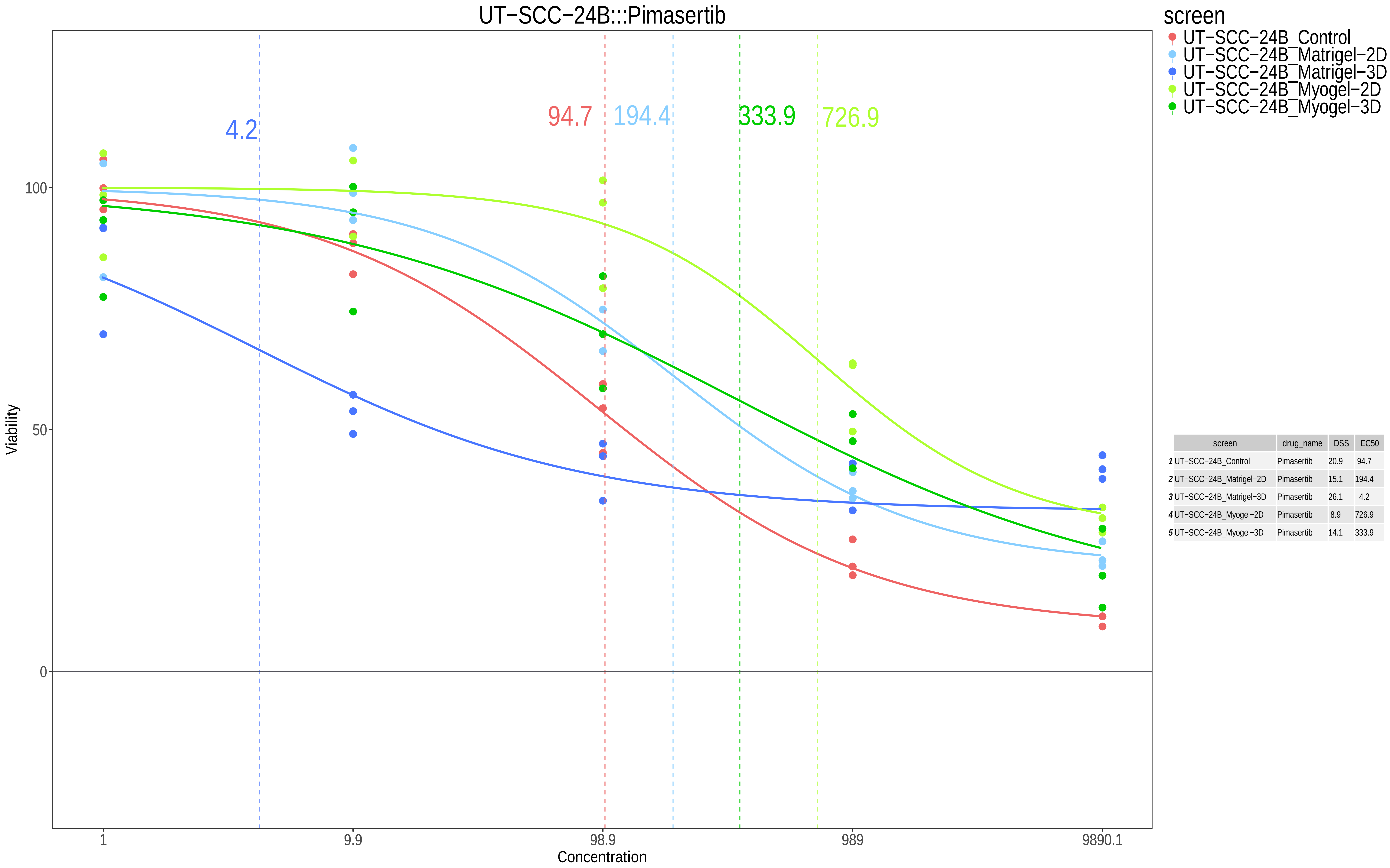

	screen	drug_name	DSS	EC50
1	UT−SCC−24B_Control	Pimasertib	20.9	94.7
2	UT−SCC−24B_Matrigel−2D	Pimasertib	15.1	194.4
3	UT−SCC−24B_Matrigel−3D	Pimasertib	26.1	4.2
4	UT−SCC−24B_Myogel−2D	Pimasertib	8.9	726.9
5	UT−SCC−24B_Myogel−3D	Pimasertib	14.1	333.9

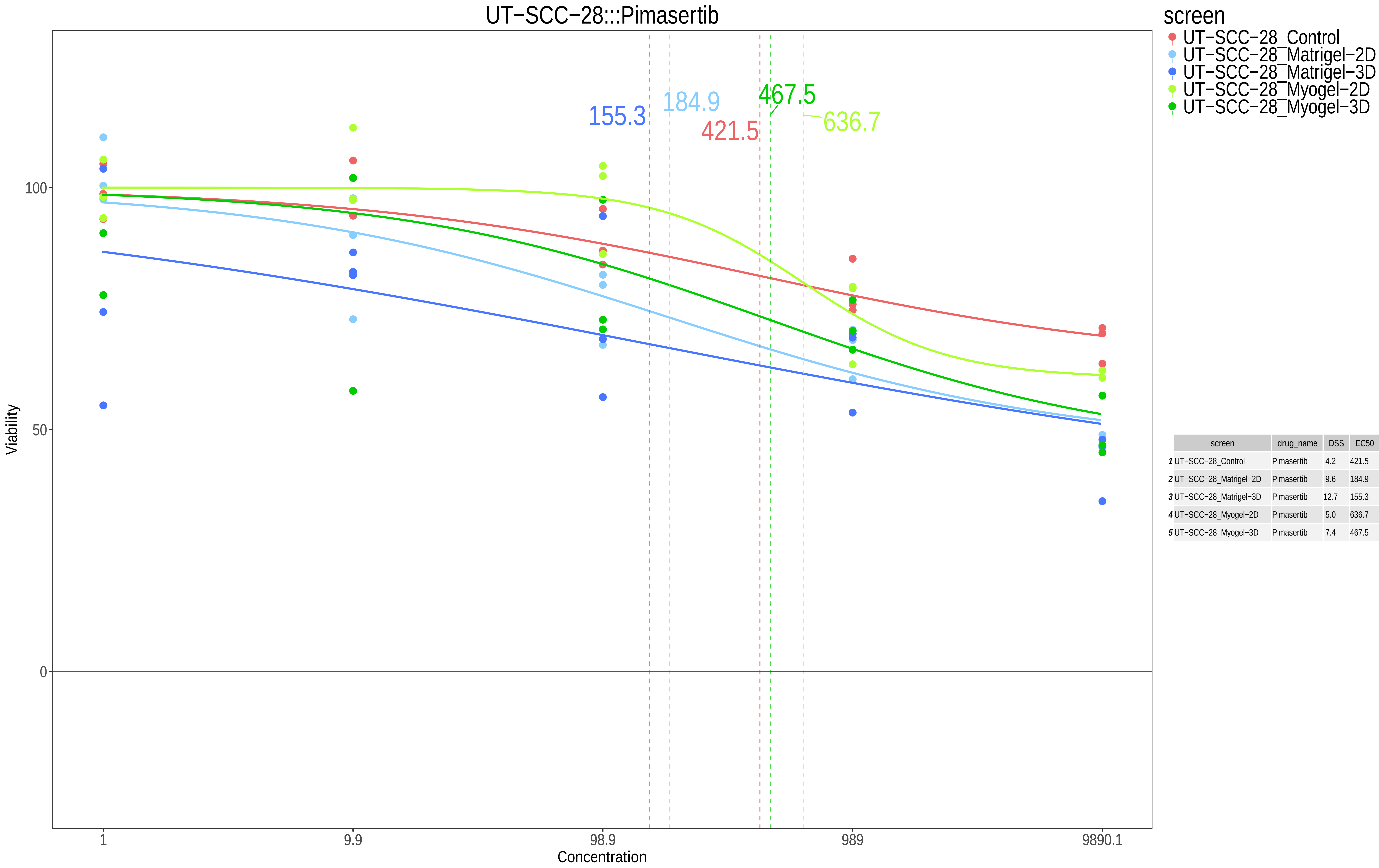

	screen	drug_name	DSS	EC50
1	UT−SCC−28_Control	Pimasertib	4.2	421.5
2	UT−SCC−28_Matrigel−2D	Pimasertib	9.6	184.9
3	UT−SCC−28_Matrigel−3D	Pimasertib	12.7	155.3
4	UT−SCC−28_Myogel−2D	Pimasertib	5.0	636.7
5	UT−SCC−28_Myogel−3D	Pimasertib	7.4	467.5

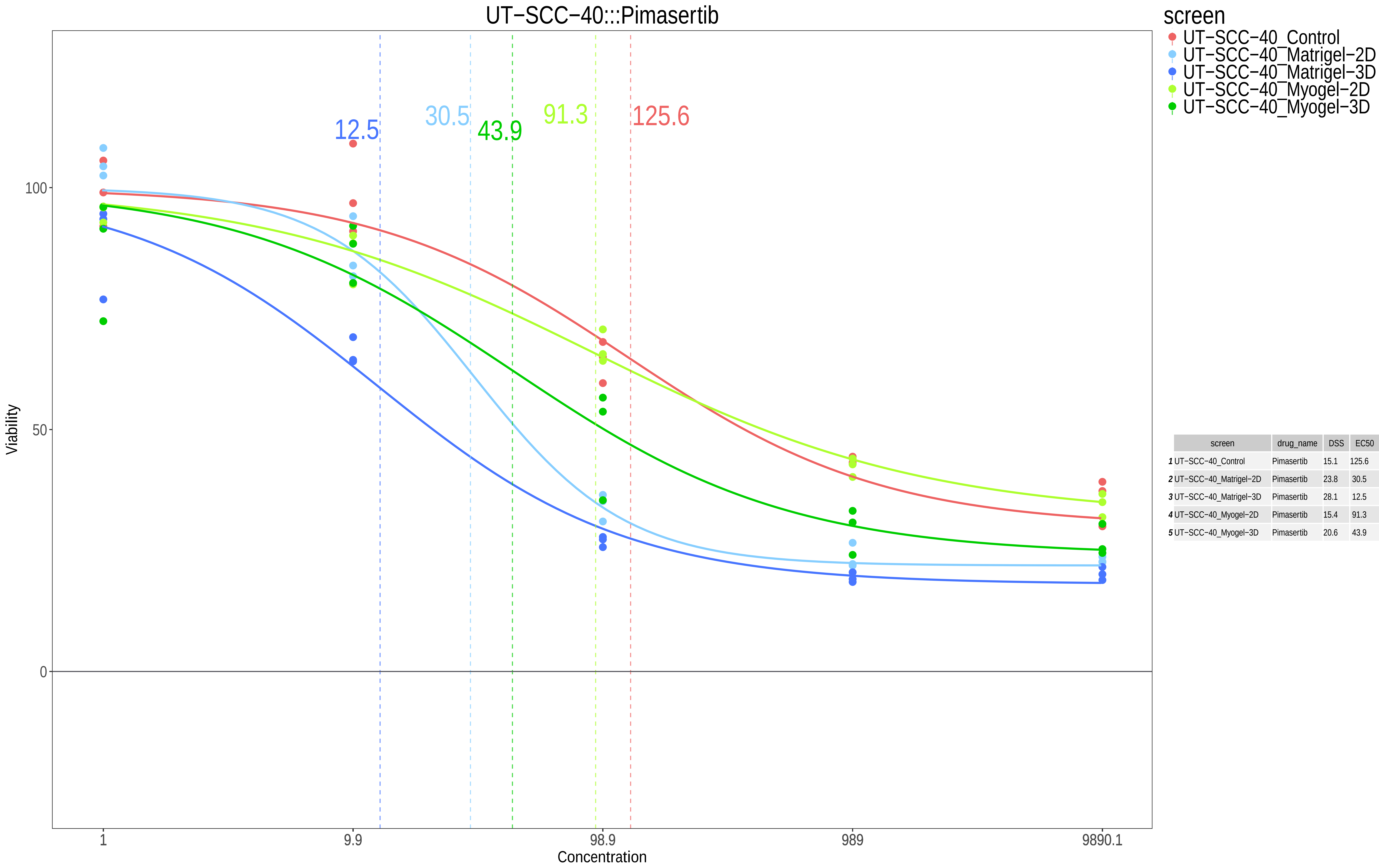

	screen	drug_name	DSS	EC50
1	UT−SCC−40_Control	Pimasertib	15.1	125.6
2	UT−SCC−40_Matrigel−2D	Pimasertib	23.8	30.5
3	UT−SCC−40_Matrigel−3D	Pimasertib	28.1	12.5
4	UT−SCC−40_Myogel−2D	Pimasertib	15.4	91.3
5	UT−SCC−40_Myogel−3D	Pimasertib	20.6	43.9

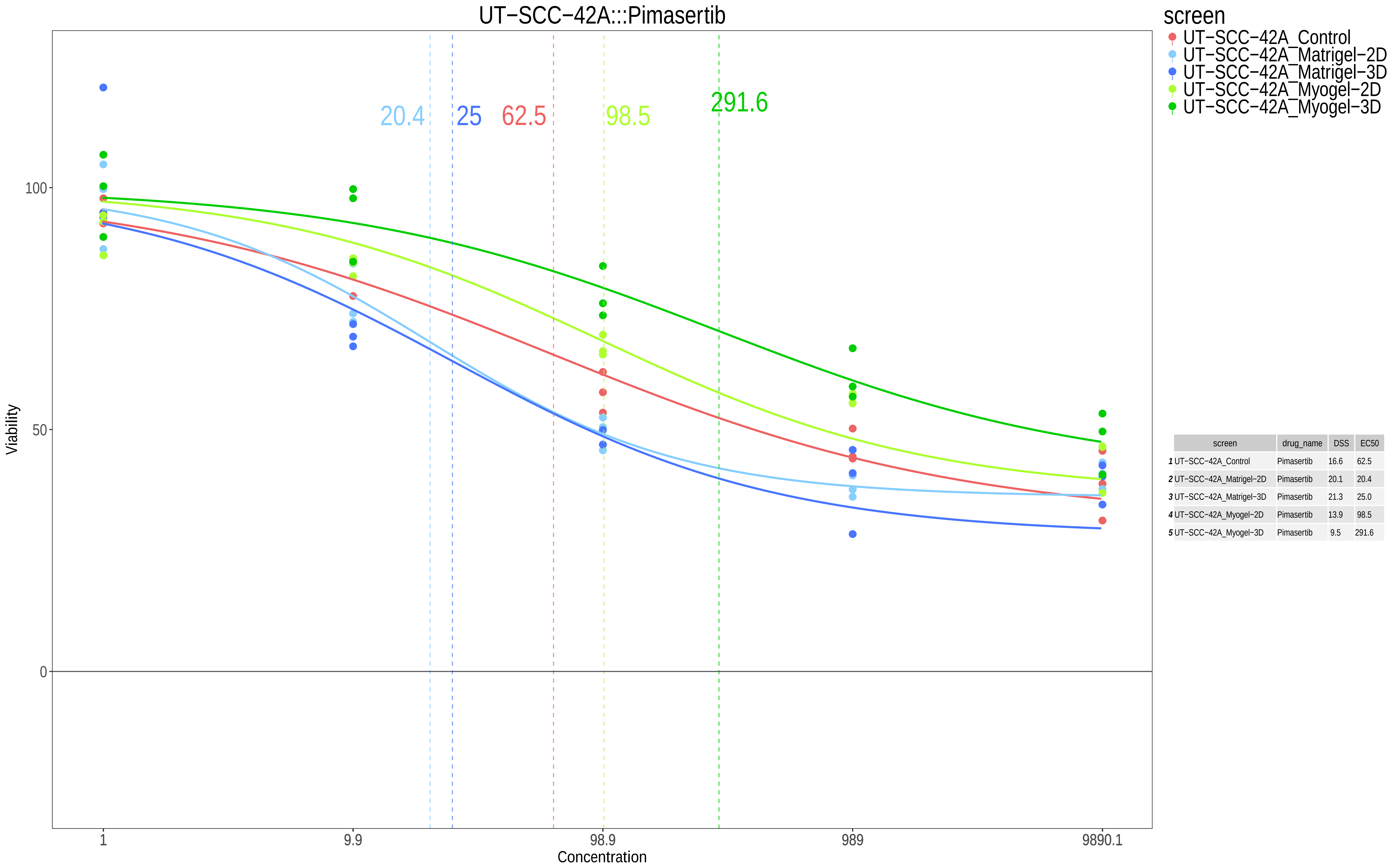

	screen	drug_name	DSS	EC50
1	UT−SCC−42A_Control	Pimasertib	16.6	62.5
2	UT−SCC−42A_Matrigel−2D	Pimasertib	20.1	20.4
3	UT−SCC−42A_Matrigel−3D	Pimasertib	21.3	25.0
4	UT−SCC−42A_Myogel−2D	Pimasertib	13.9	98.5
5	UT−SCC−42A_Myogel−3D	Pimasertib	9.5	291.6

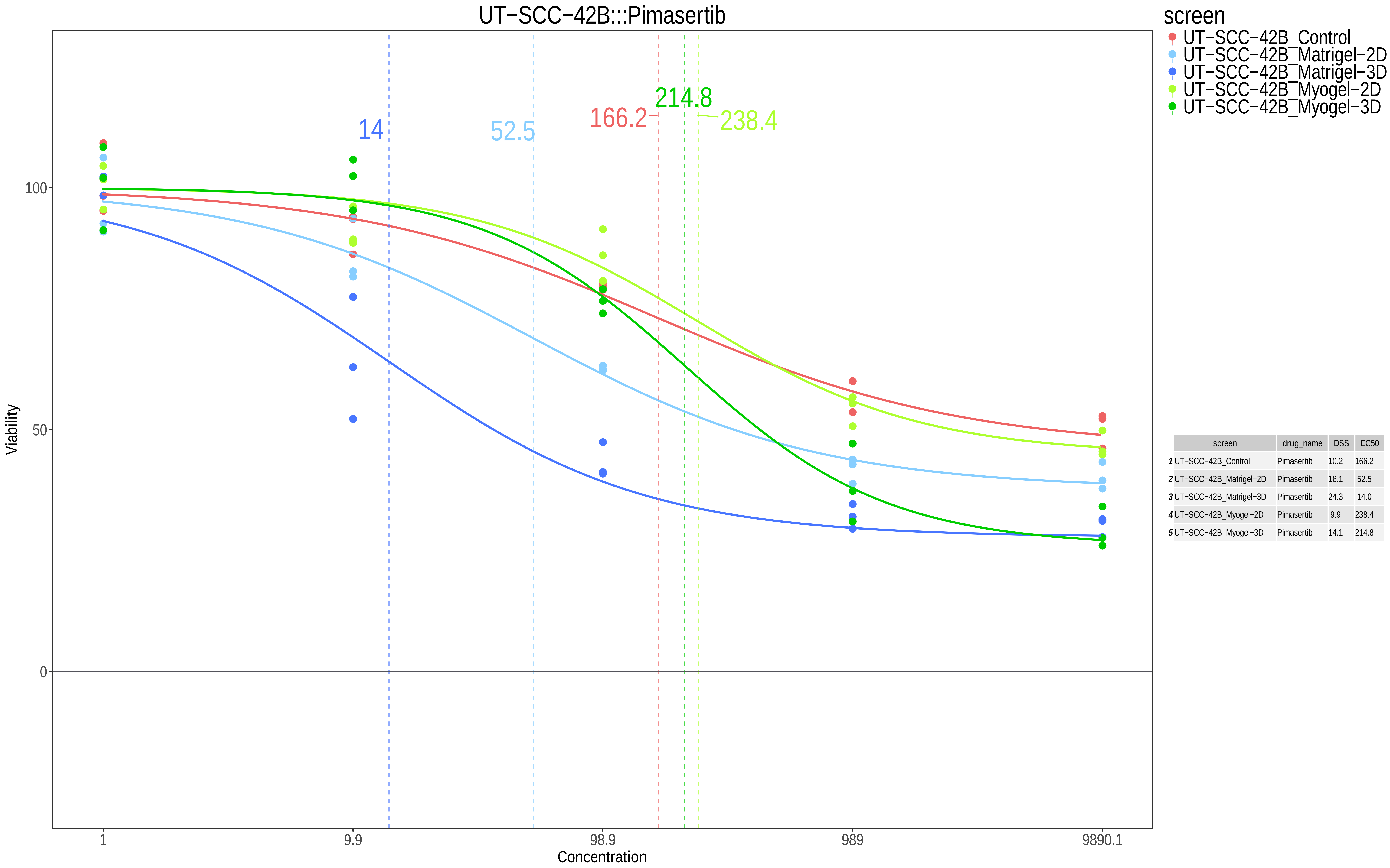

	screen	drug_name	DSS	EC50
1	UT−SCC−42B_Control	Pimasertib	10.2	166.2
2	UT−SCC−42B_Matrigel−2D	Pimasertib	16.1	52.5
3	UT−SCC−42B_Matrigel−3D	Pimasertib	24.3	14.0
4	UT−SCC−42B_Myogel−2D	Pimasertib	9.9	238.4
5	UT−SCC−42B_Myogel−3D	Pimasertib	14.1	214.8

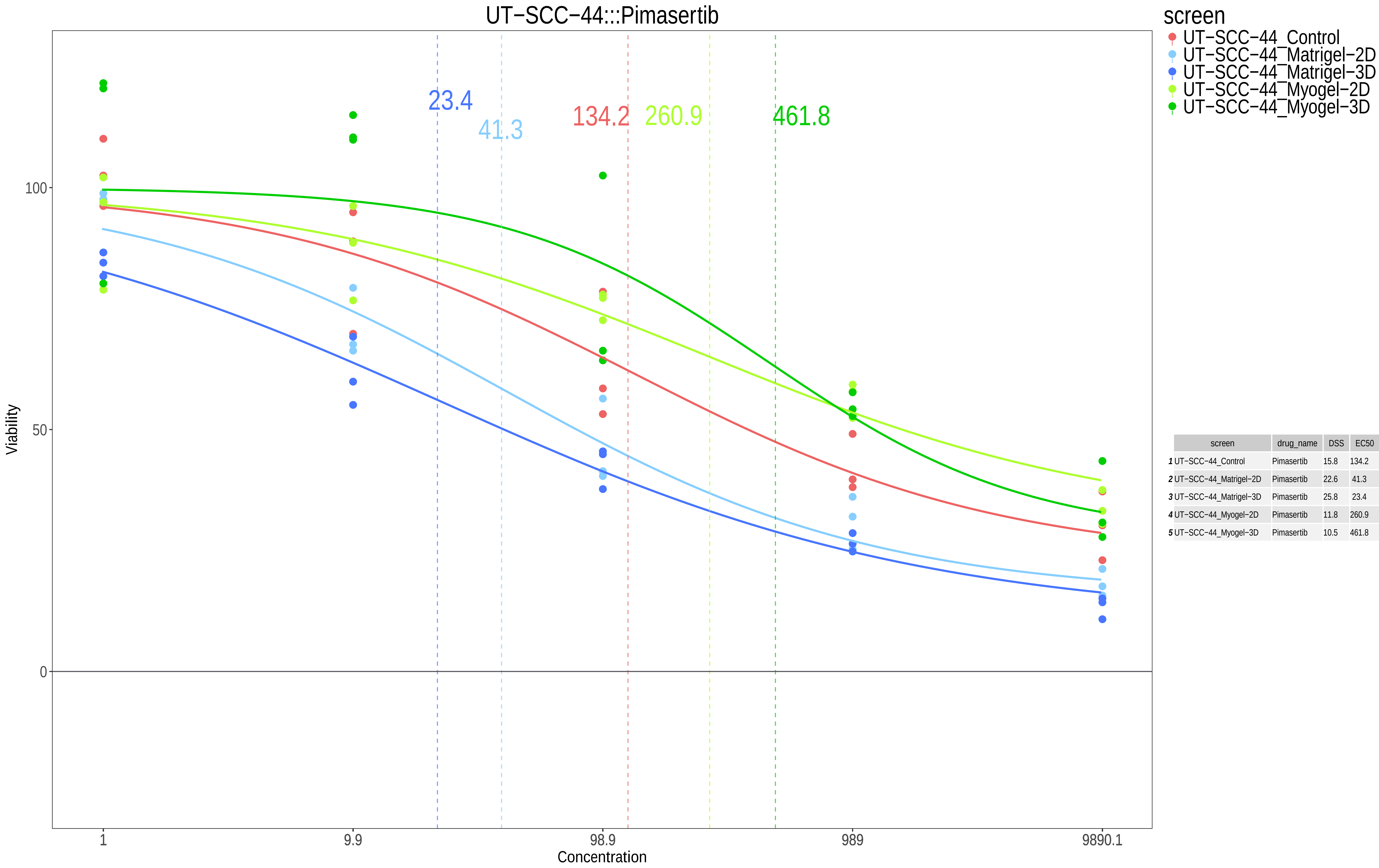

	screen	drug_name	DSS	EC50
1	UT−SCC−44_Control	Pimasertib	15.8	134.2
2	UT−SCC−44_Matrigel−2D	Pimasertib	22.6	41.3
3	UT−SCC−44_Matrigel−3D	Pimasertib	25.8	23.4
4	UT−SCC−44_Myogel−2D	Pimasertib	11.8	260.9
5	UT−SCC−44_Myogel−3D	Pimasertib	10.5	461.8

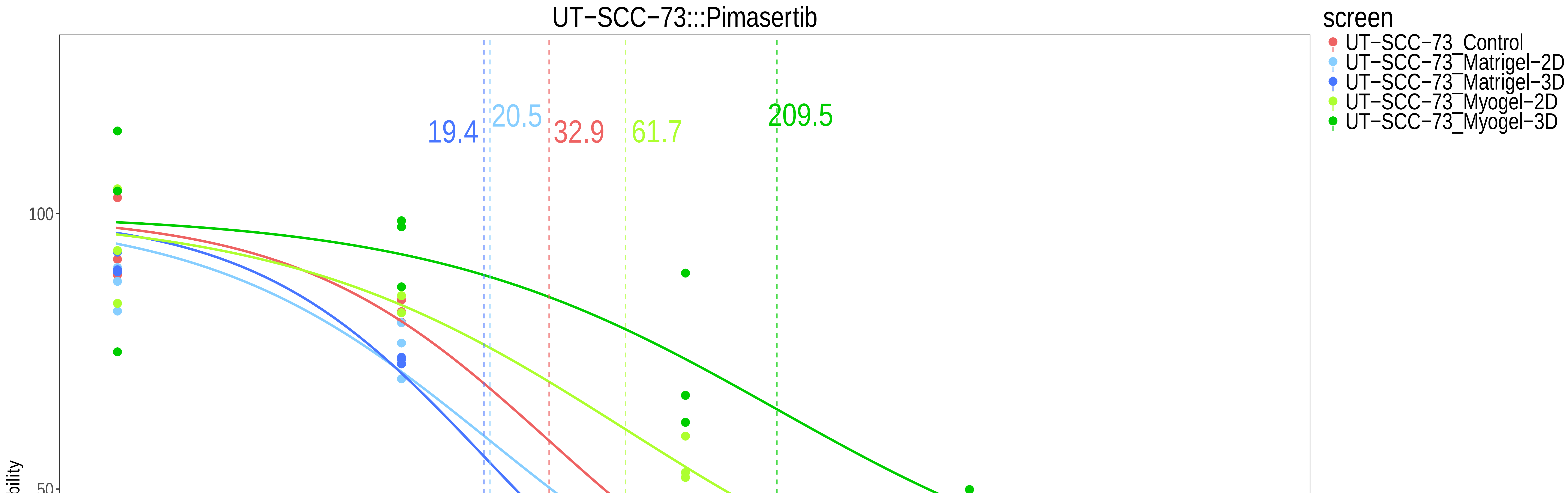

	screen	drug_name	DSS	EC50
1	UT−SCC−73_Control	Pimasertib	24.0	32.9
2	UT−SCC−73_Matrigel−2D	Pimasertib	26.0	20.5
3	UT−SCC−73_Matrigel−3D	Pimasertib	28.3	19.4
4	UT−SCC−73_Myogel−2D	Pimasertib	19.7	61.7
5	UT−SCC−73_Myogel−3D	Pimasertib	13.1	209.5

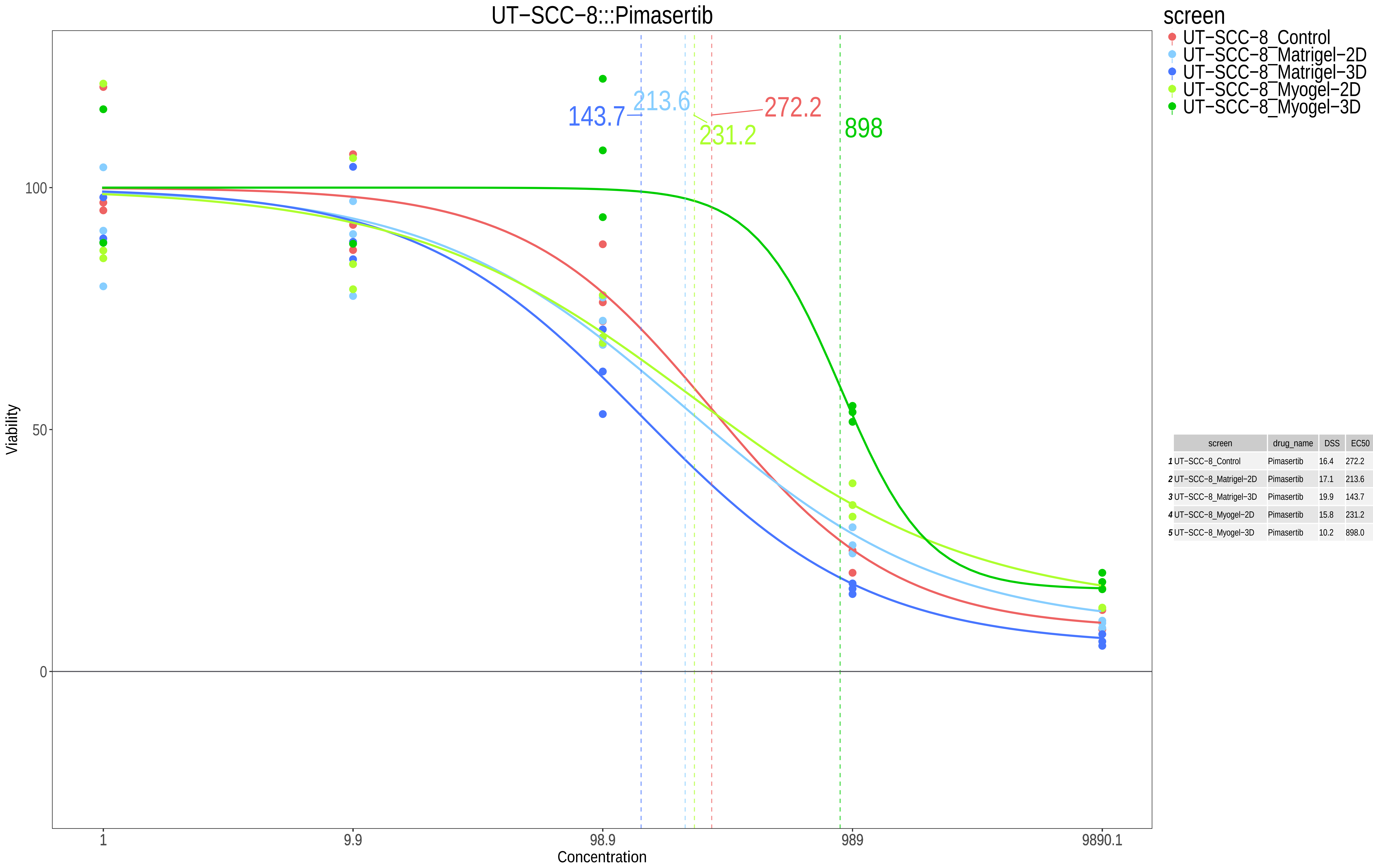

	screen	drug_name	DSS	EC50
1	UT−SCC−8_Control	Pimasertib	16.4	272.2
2	UT−SCC−8_Matrigel−2D	Pimasertib	17.1	213.6
3	UT−SCC−8_Matrigel−3D	Pimasertib	19.9	143.7
4	UT−SCC−8_Myogel−2D	Pimasertib	15.8	231.2
5	UT−SCC−8_Myogel−3D	Pimasertib	10.2	898.0

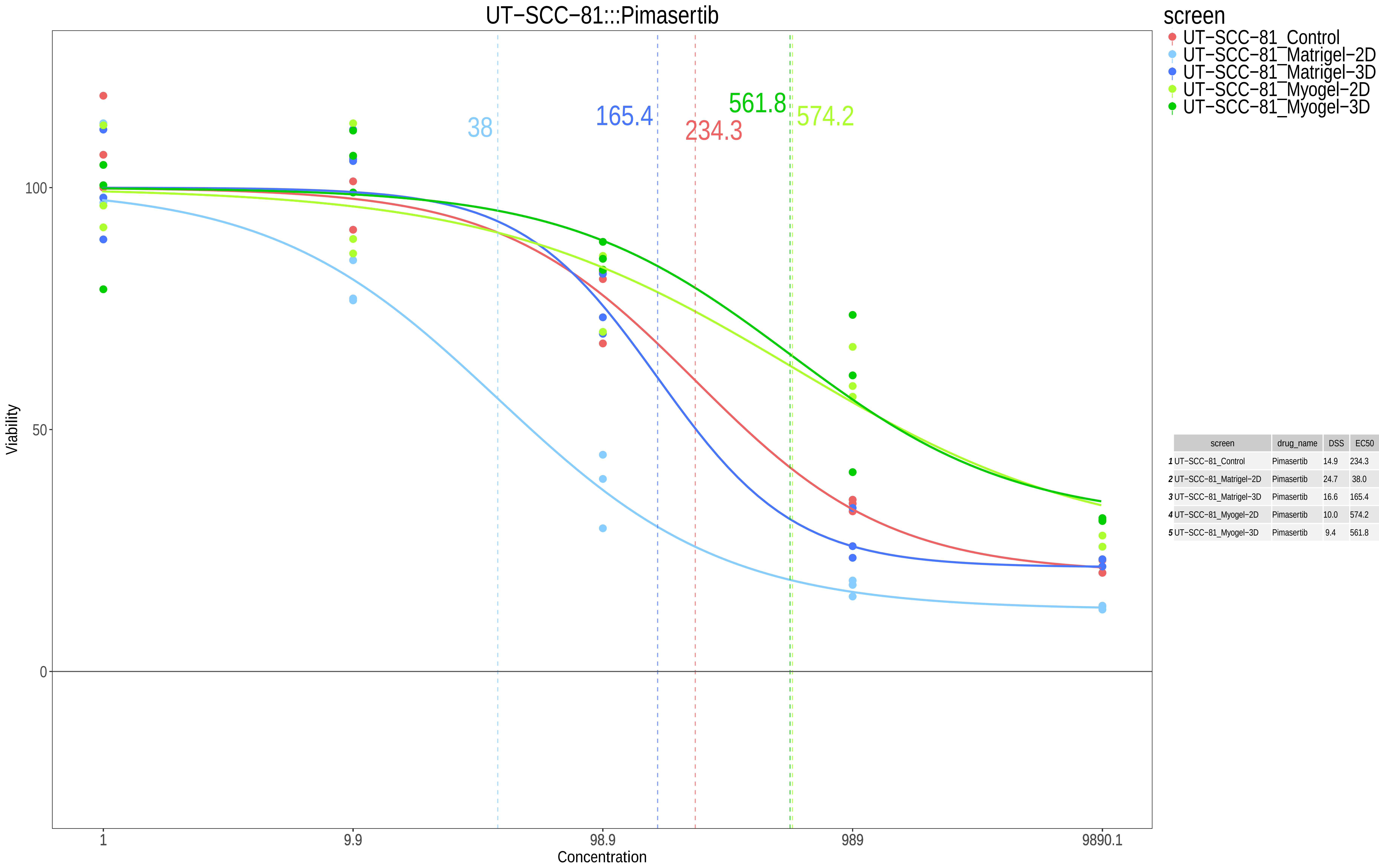

	screen	drug_name	DSS	EC50
1	UT−SCC−81_Control	Pimasertib	14.9	234.3
2	UT−SCC−81_Matrigel−2D	Pimasertib	24.7	38.0
3	UT−SCC−81_Matrigel−3D	Pimasertib	16.6	165.4
4	UT−SCC−81_Myogel−2D	Pimasertib	10.0	574.2
5	UT−SCC−81_Myogel−3D	Pimasertib	9.4	561.8

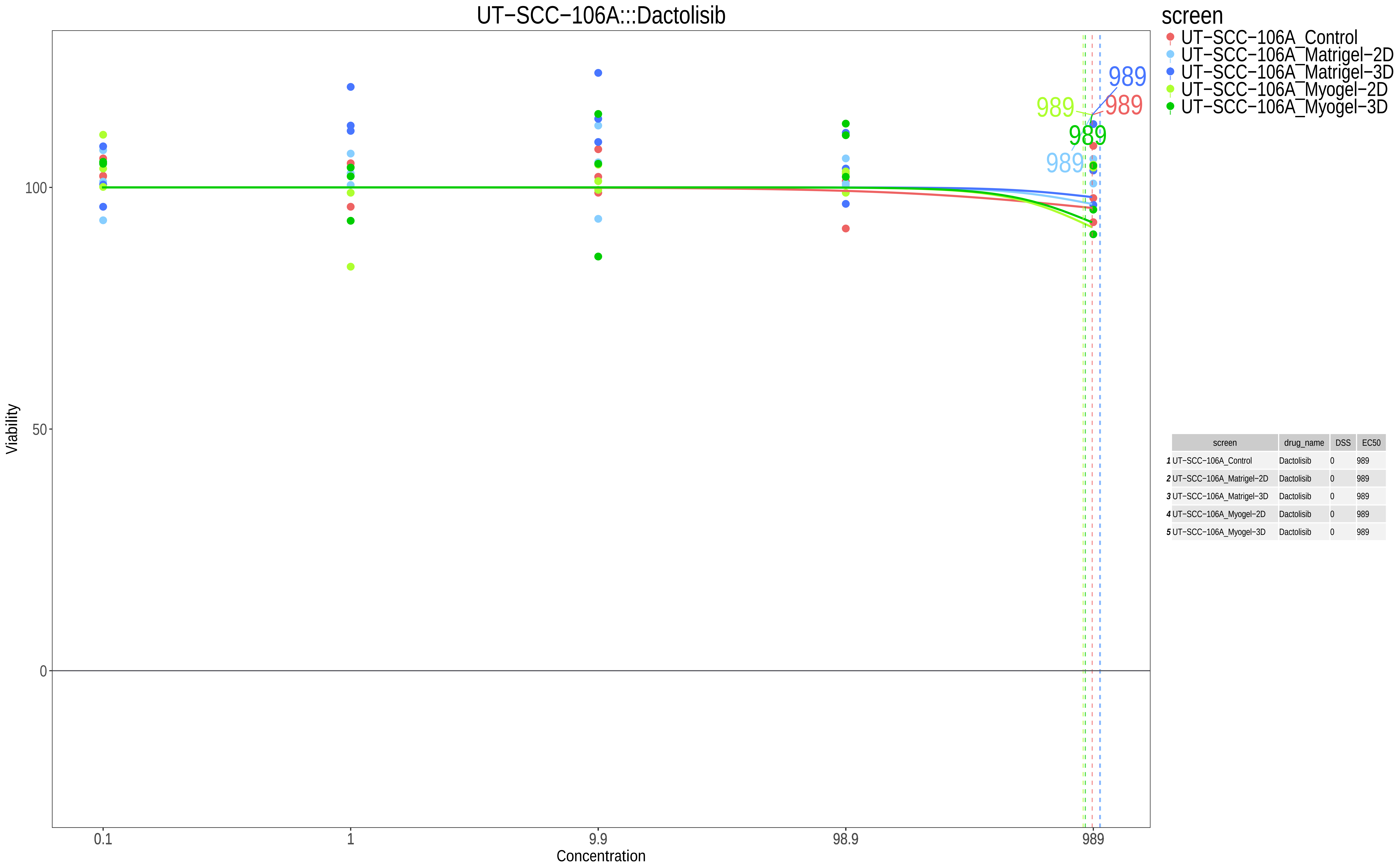

	screen	drug_name	DSS	EC50
1	UT−SCC−106A_Control	Dactolisib	0	989
2	UT−SCC−106A_Matrigel−2D	Dactolisib	0	989
3	UT−SCC−106A_Matrigel−3D	Dactolisib	0	989
4	UT−SCC−106A_Myogel−2D	Dactolisib	0	989
5	UT−SCC−106A_Myogel−3D	Dactolisib	0	989

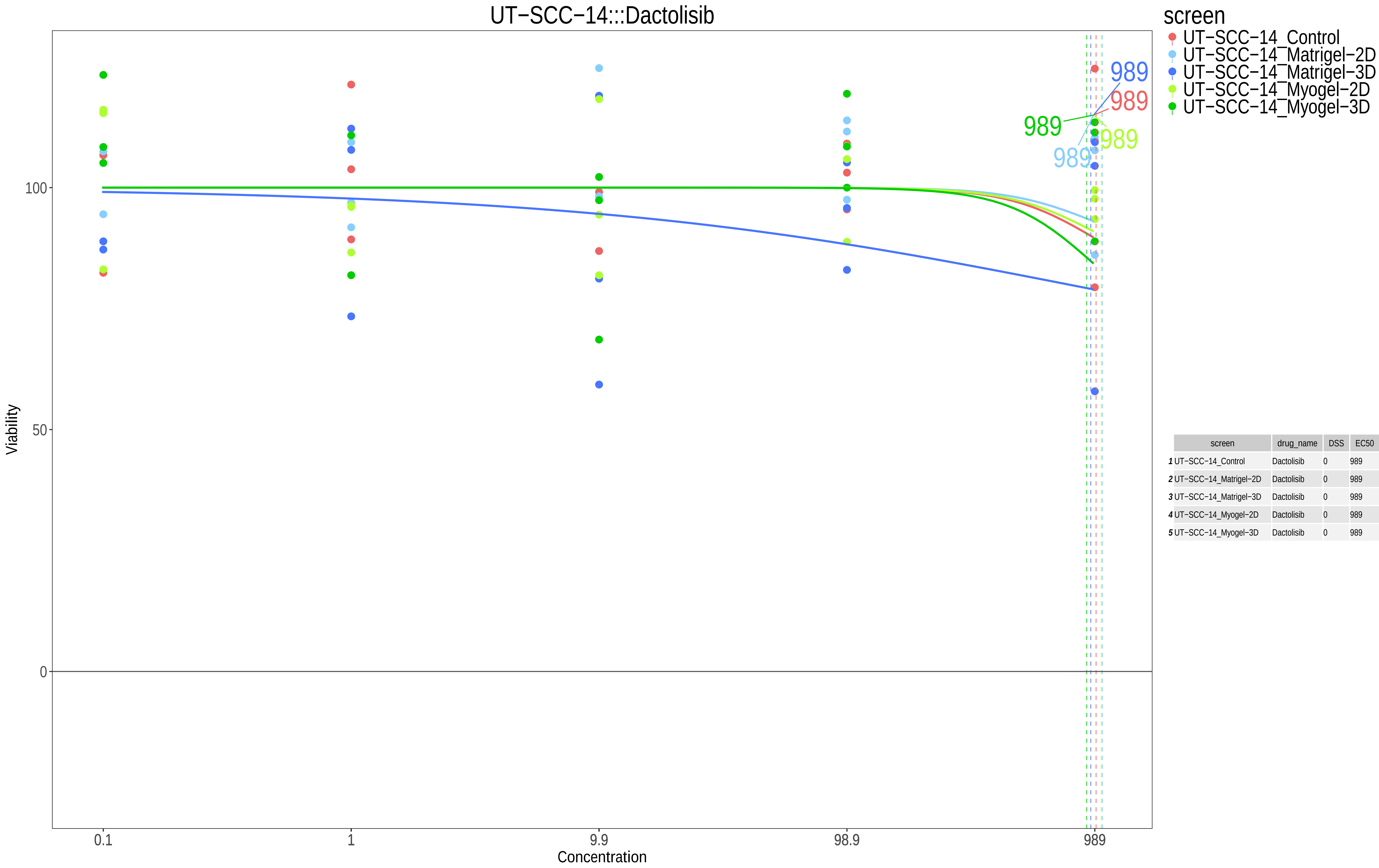

	screen	drug_name	DSS	EC50
1	UT−SCC−14_Control	Dactolisib	0	989
2	UT−SCC−14_Matrigel−2D	Dactolisib	0	989
3	UT−SCC−14_Matrigel−3D	Dactolisib	0	989
4	UT−SCC−14_Myogel−2D	Dactolisib	0	989
5	UT−SCC−14_Myogel−3D	Dactolisib	0	989

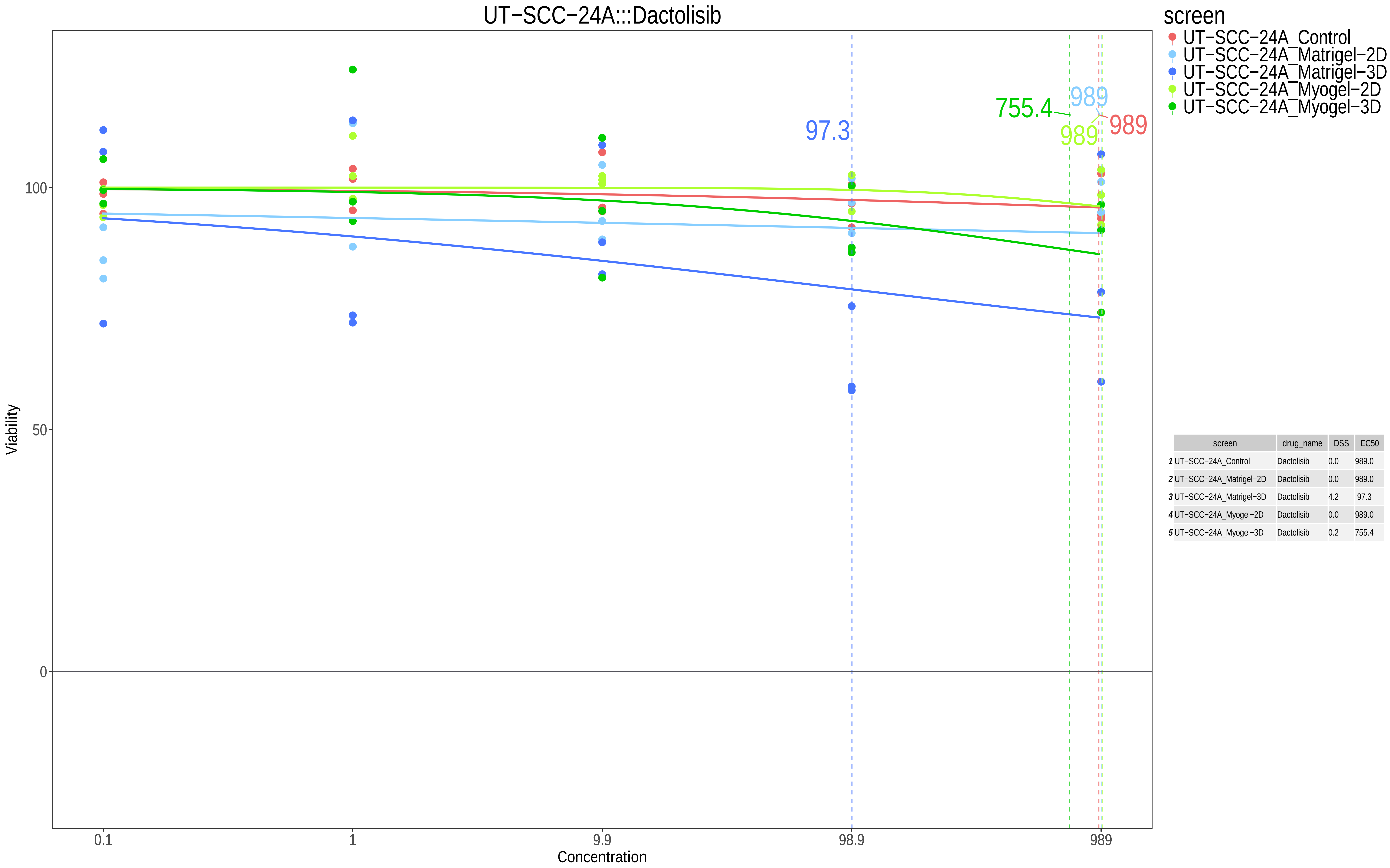

	screen	drug_name	DSS	EC50
1	UT−SCC−24A_Control	Dactolisib	0.0	989.0
2	UT−SCC−24A_Matrigel−2D	Dactolisib	0.0	989.0
3	UT−SCC−24A_Matrigel−3D	Dactolisib	4.2	97.3
4	UT−SCC−24A_Myogel−2D	Dactolisib	0.0	989.0
5	UT−SCC−24A_Myogel−3D	Dactolisib	0.2	755.4

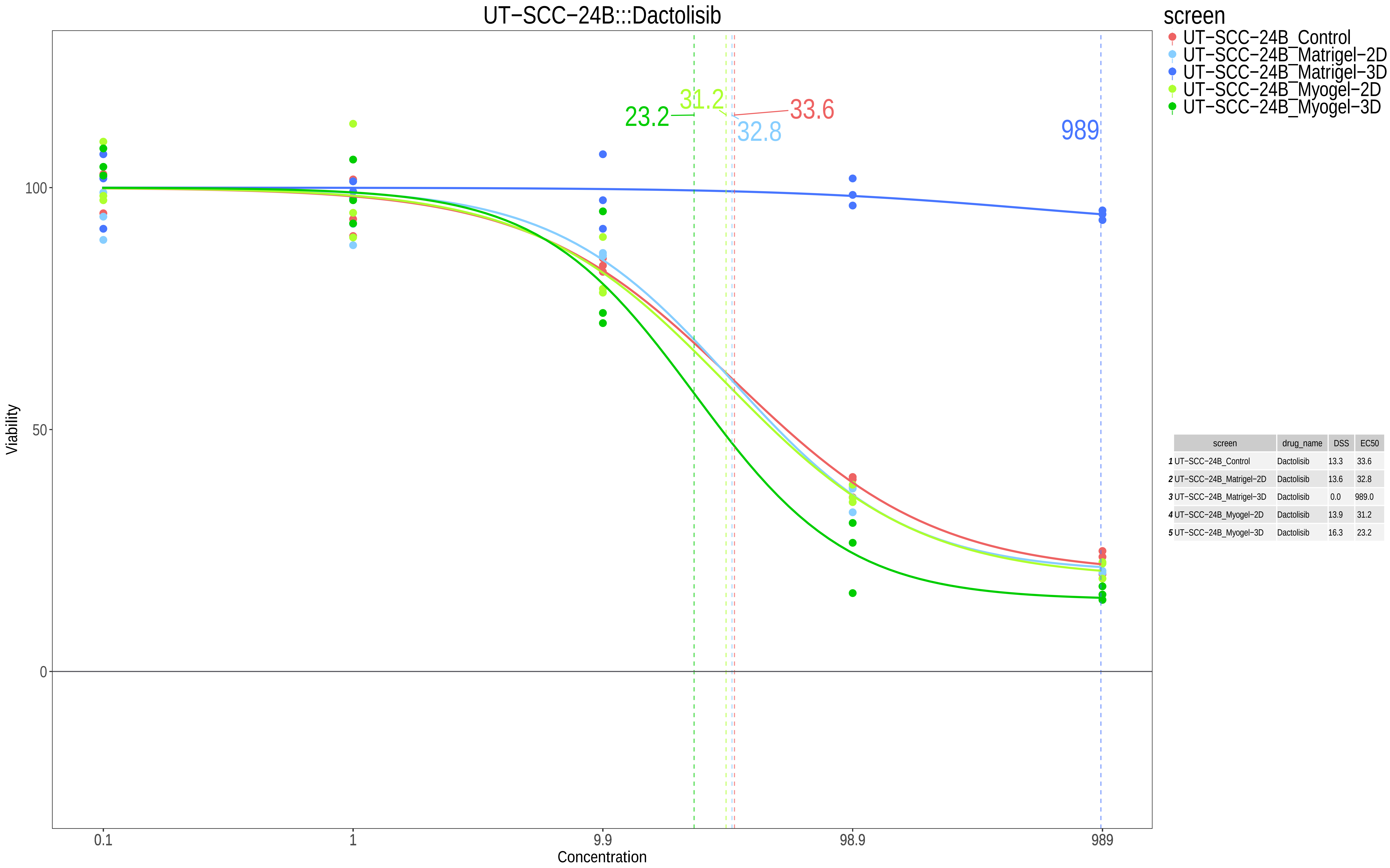

	screen	drug_name	DSS	EC50
1	UT−SCC−24B_Control	Dactolisib	13.3	33.6
2	UT−SCC−24B_Matrigel−2D	Dactolisib	13.6	32.8
3	UT−SCC−24B_Matrigel−3D	Dactolisib	0.0	989.0
4	UT−SCC−24B_Myogel−2D	Dactolisib	13.9	31.2
5	UT−SCC−24B_Myogel−3D	Dactolisib	16.3	23.2

UT−SCC−28:::Dactolisib

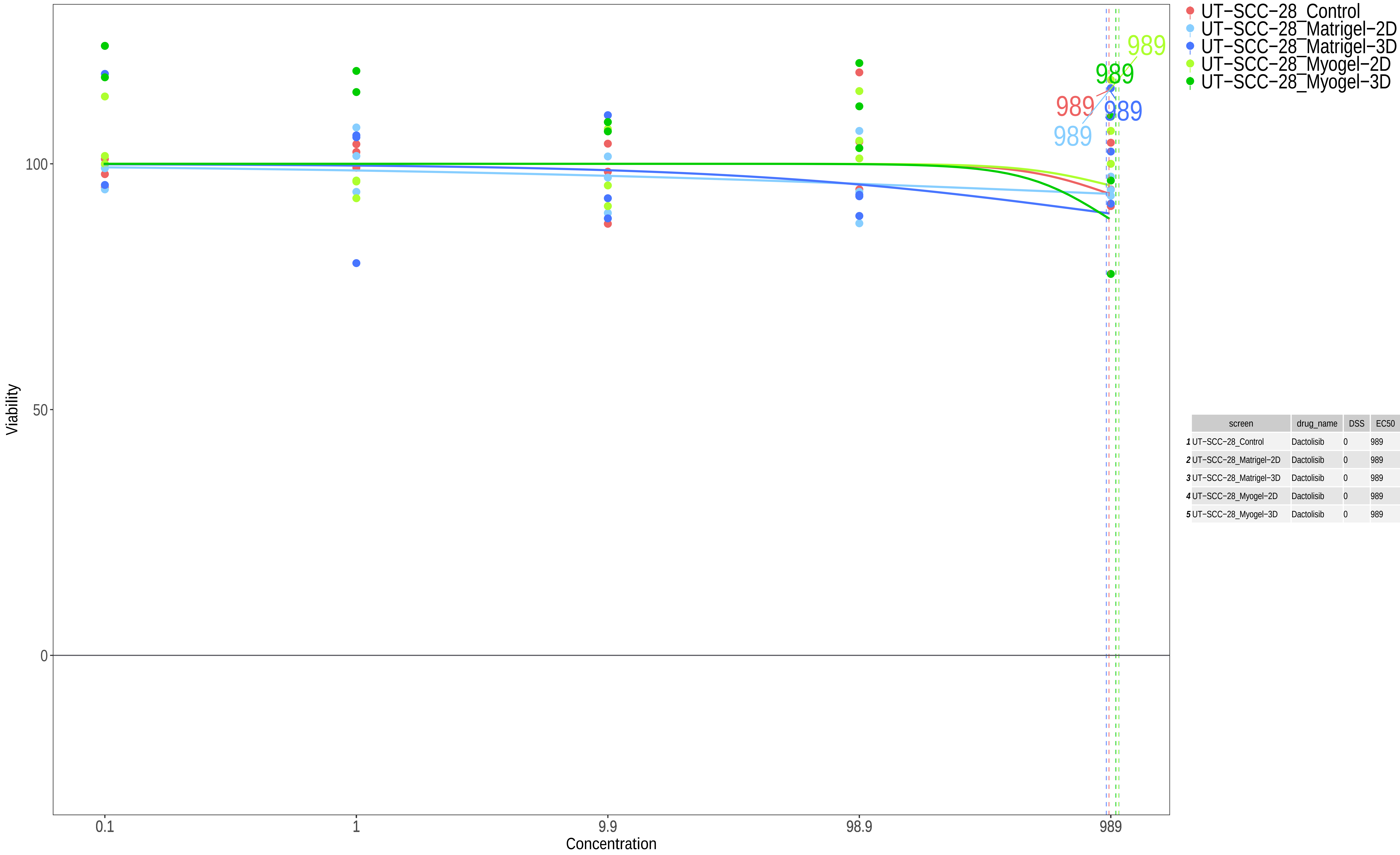

	screen	drug_name	DSS	EC50
1	UT−SCC−28_Control	Dactolisib	0	989
2	UT−SCC−28_Matrigel−2D	Dactolisib	0	989
3	UT−SCC−28_Matrigel−3D	Dactolisib	0	989
4	UT−SCC−28_Myogel−2D	Dactolisib	0	989
5	UT−SCC−28_Myogel−3D	Dactolisib	0	989

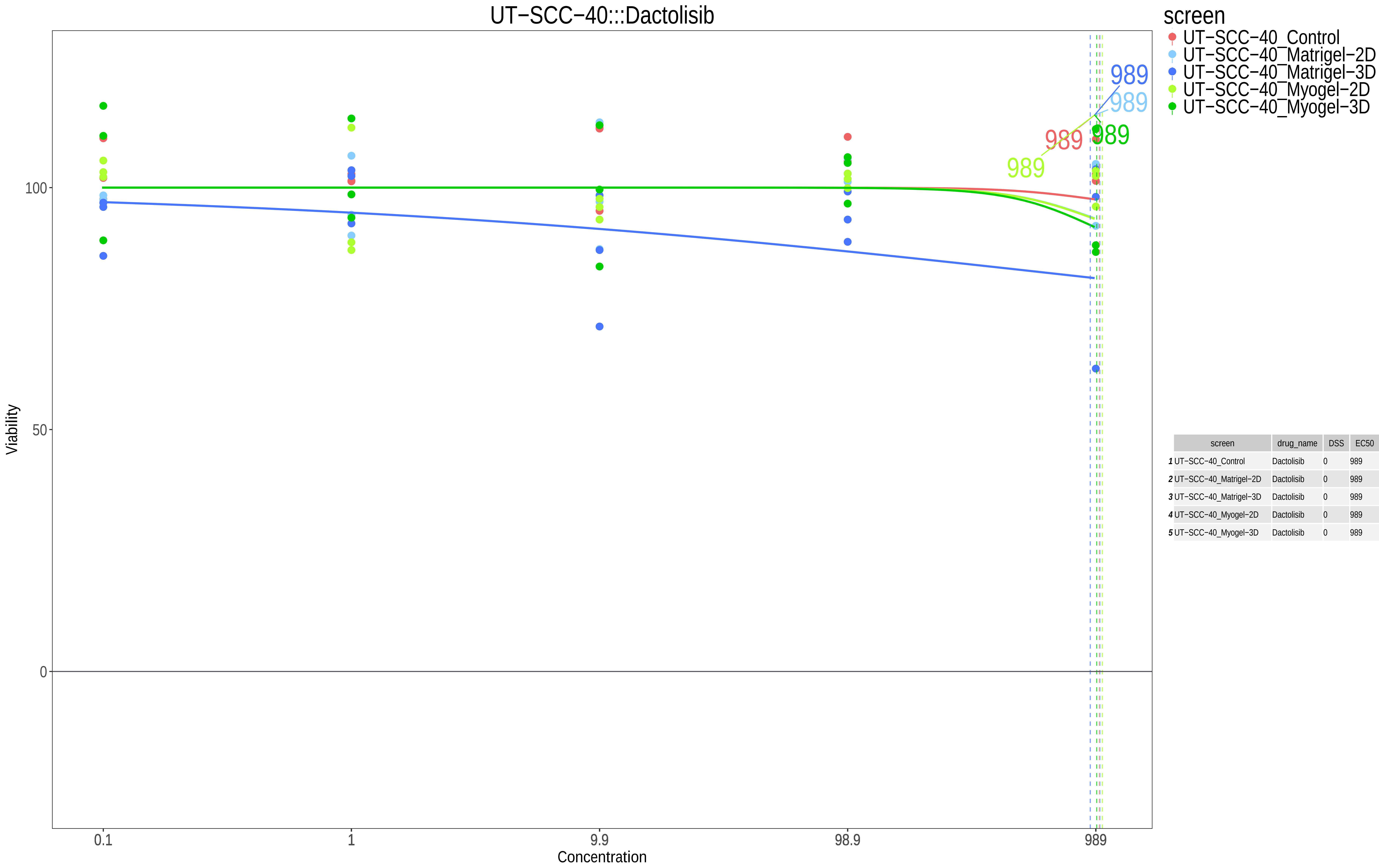

	screen	drug_name	DSS	EC50
1	UT−SCC−40_Control	Dactolisib	0	989
2	UT−SCC−40_Matrigel−2D	Dactolisib	0	989
3	UT−SCC−40_Matrigel−3D	Dactolisib	0	989
4	UT−SCC−40_Myogel−2D	Dactolisib	0	989
5	UT−SCC−40_Myogel−3D	Dactolisib	0	989

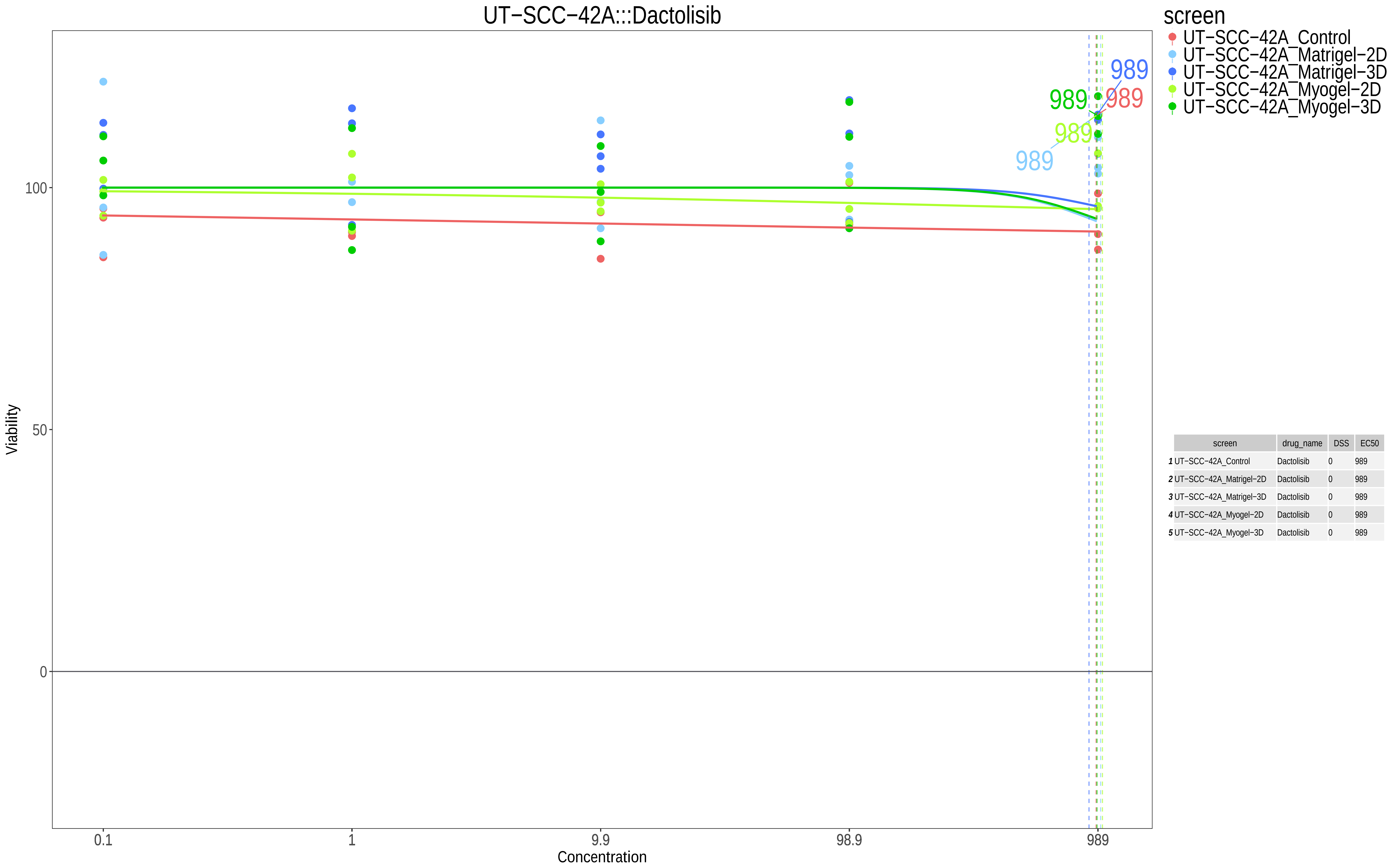

	screen	drug_name	DSS	EC50
1	UT−SCC−42A_Control	Dactolisib	0	989
2	UT−SCC−42A_Matrigel−2D	Dactolisib	0	989
3	UT−SCC−42A_Matrigel−3D	Dactolisib	0	989
4	UT−SCC−42A_Myogel−2D	Dactolisib	0	989
5	UT−SCC−42A_Myogel−3D	Dactolisib	0	989

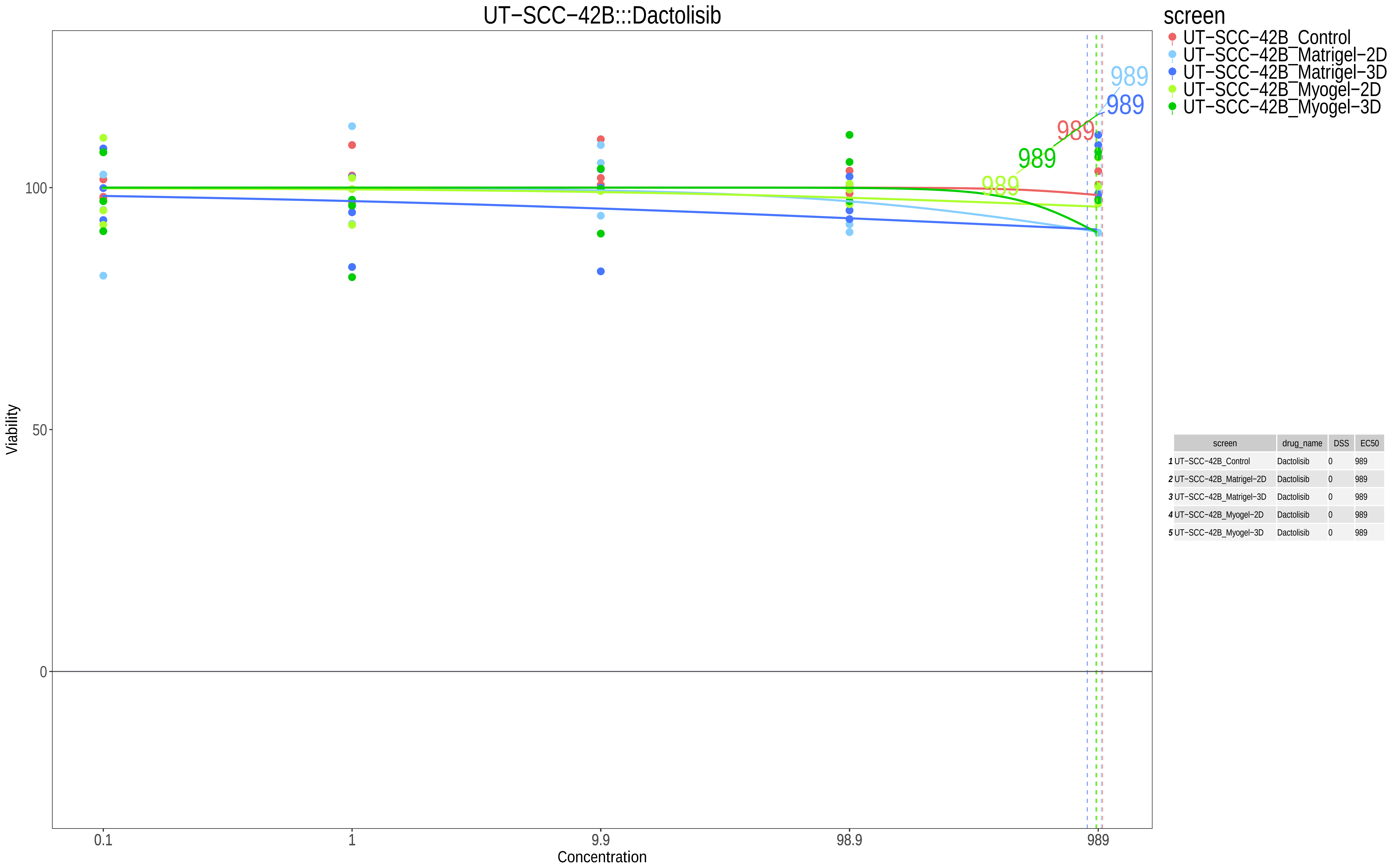

	screen	drug_name	DSS	EC50
1	UT−SCC−42B_Control	Dactolisib	0	989
2	UT−SCC−42B_Matrigel−2D	Dactolisib	0	989
3	UT−SCC−42B_Matrigel−3D	Dactolisib	0	989
4	UT−SCC−42B_Myogel−2D	Dactolisib	0	989
5	UT−SCC−42B_Myogel−3D	Dactolisib	0	989

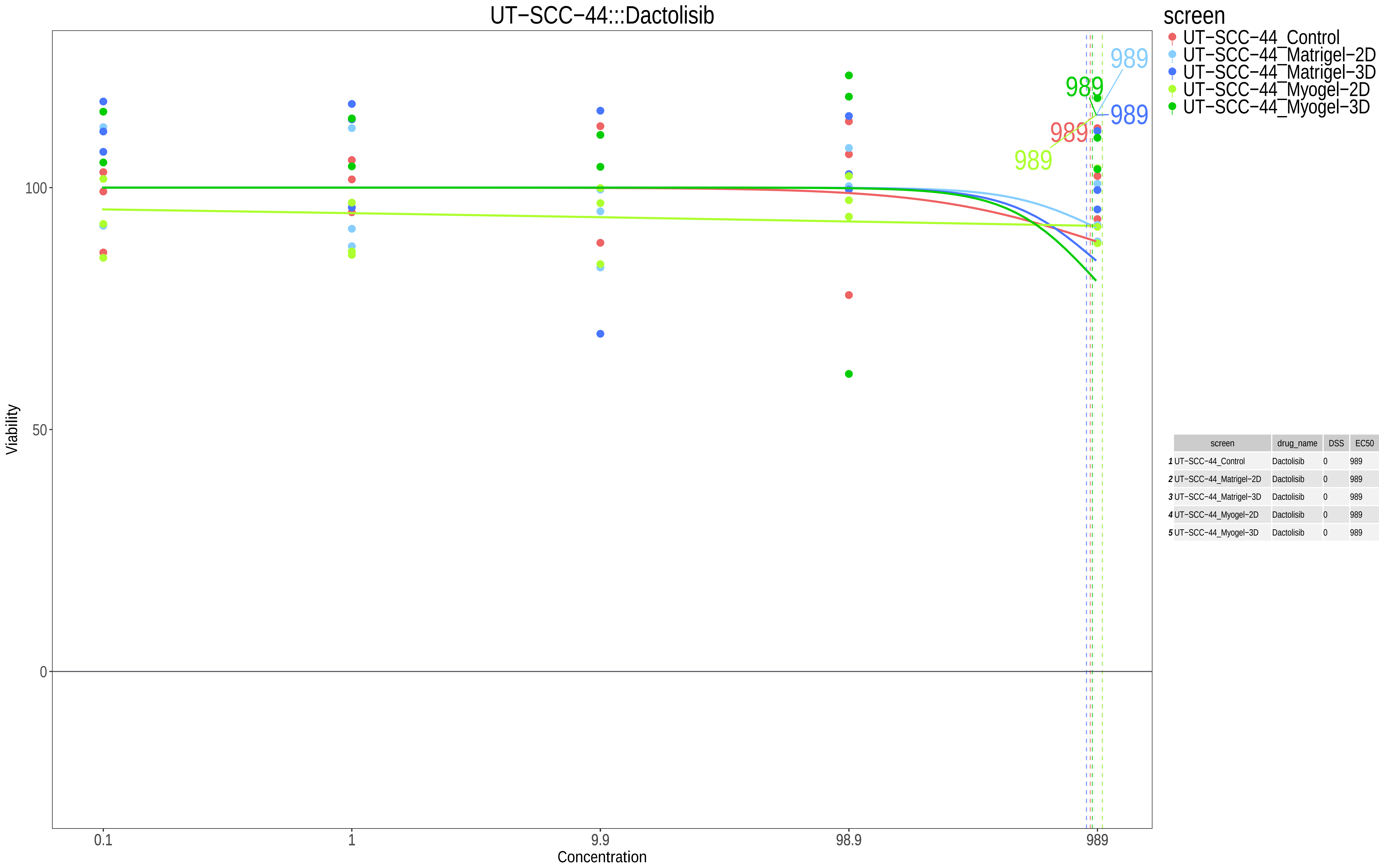

	screen	drug_name	DSS	EC50
1	UT−SCC−44_Control	Dactolisib	0	989
2	UT−SCC−44_Matrigel−2D	Dactolisib	0	989
3	UT−SCC−44_Matrigel−3D	Dactolisib	0	989
4	UT−SCC−44_Myogel−2D	Dactolisib	0	989
5	UT−SCC−44_Myogel−3D	Dactolisib	0	989

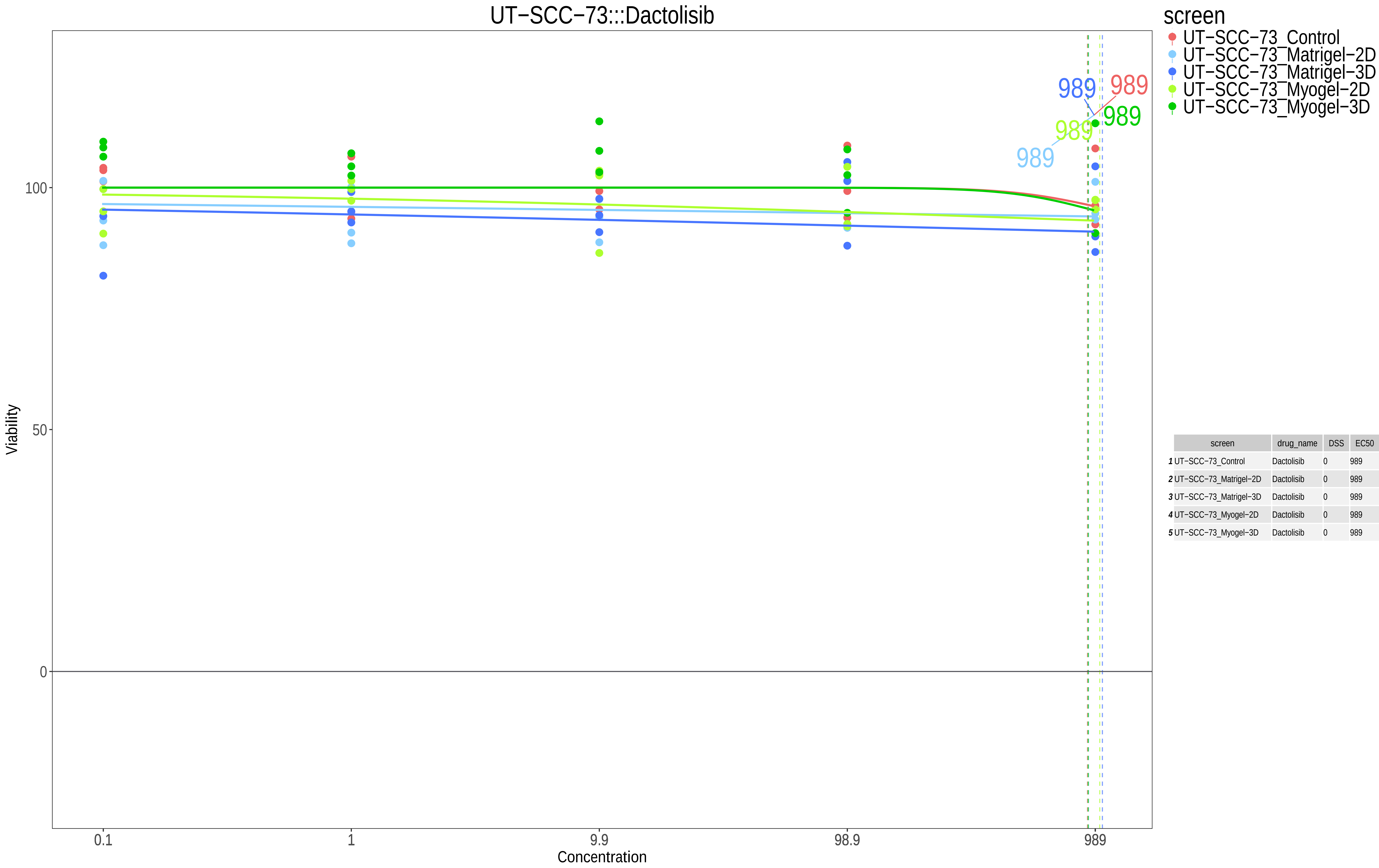

	screen	drug_name	DSS	EC50
1	UT−SCC−73_Control	Dactolisib	0	989
2	UT−SCC−73_Matrigel−2D	Dactolisib	0	989
3	UT−SCC−73_Matrigel−3D	Dactolisib	0	989
4	UT−SCC−73_Myogel−2D	Dactolisib	0	989
5	UT−SCC−73_Myogel−3D	Dactolisib	0	989

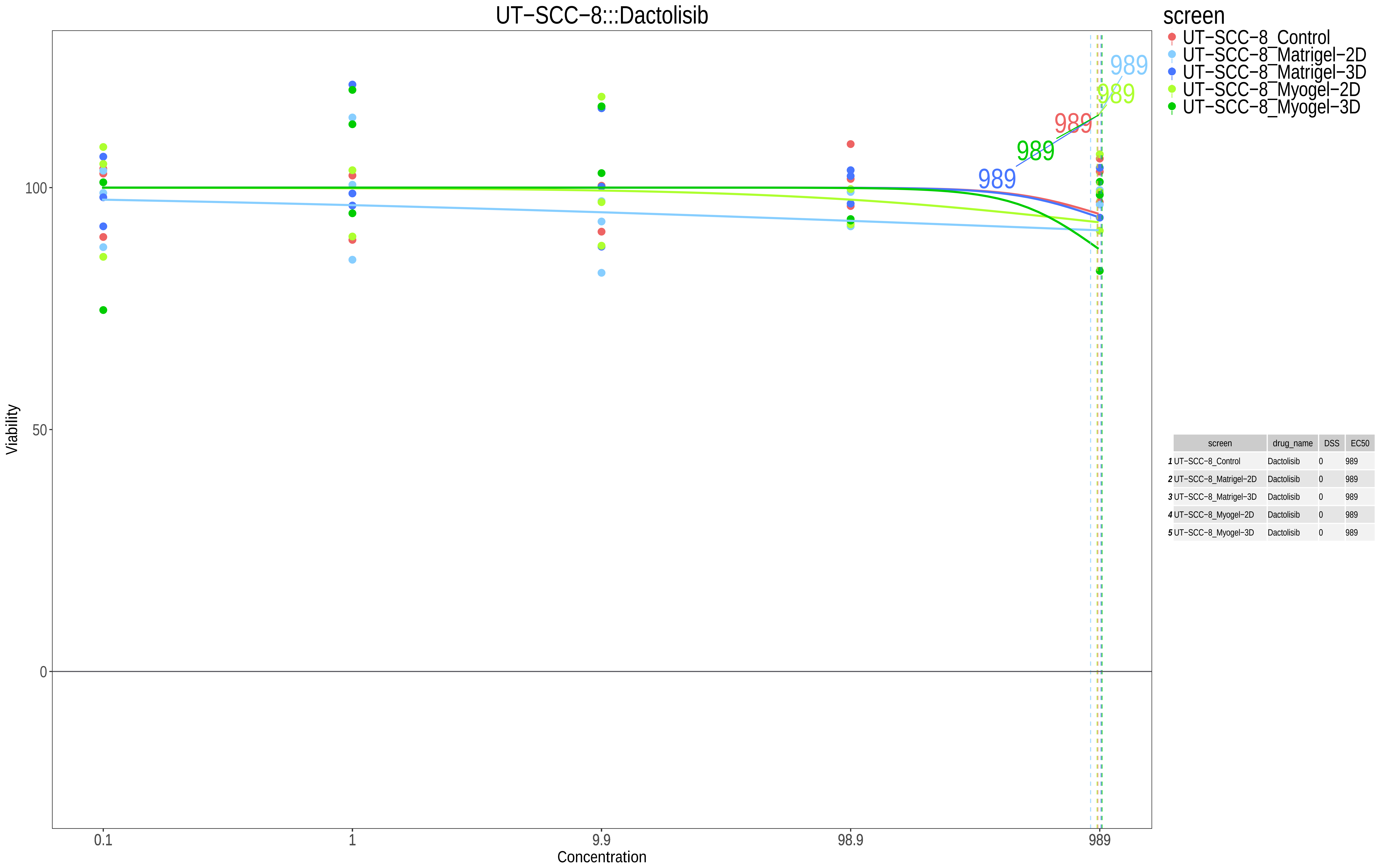

	screen	drug_name	DSS	EC50
1	UT−SCC−8_Control	Dactolisib	0	989
2	UT−SCC−8_Matrigel−2D	Dactolisib	0	989
3	UT−SCC−8_Matrigel−3D	Dactolisib	0	989
4	UT−SCC−8_Myogel−2D	Dactolisib	0	989
5	UT−SCC−8_Myogel−3D	Dactolisib	0	989

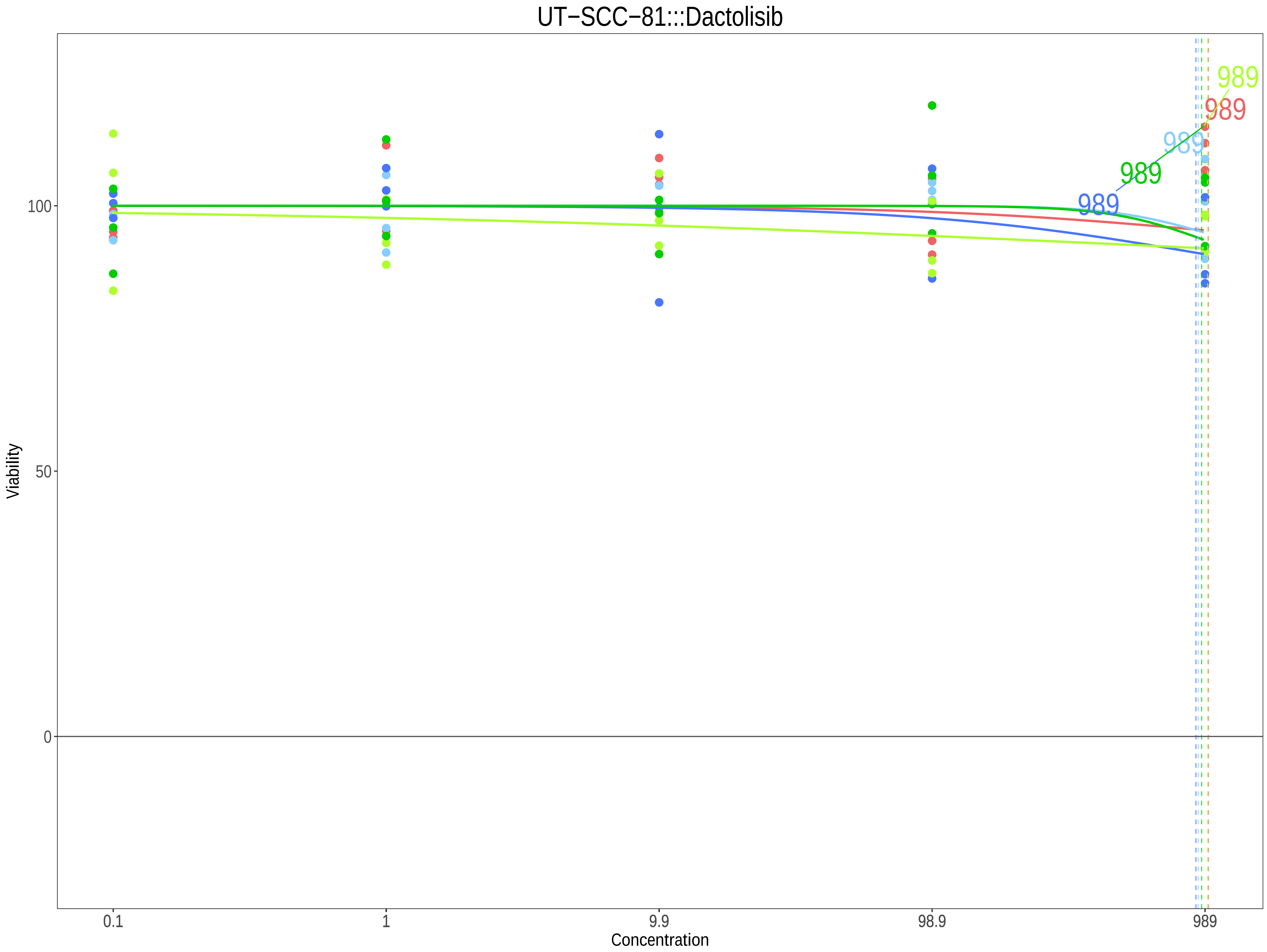

	screen	drug_name	DSS	EC50
1	UT−SCC−81_Control	Dactolisib	0	989
2	UT−SCC−81_Matrigel−2D	Dactolisib	0	989
3	UT−SCC−81_Matrigel−3D	Dactolisib	0	989
4	UT−SCC−81_Myogel−2D	Dactolisib	0	989
5	UT−SCC−81_Myogel−3D	Dactolisib	0	989

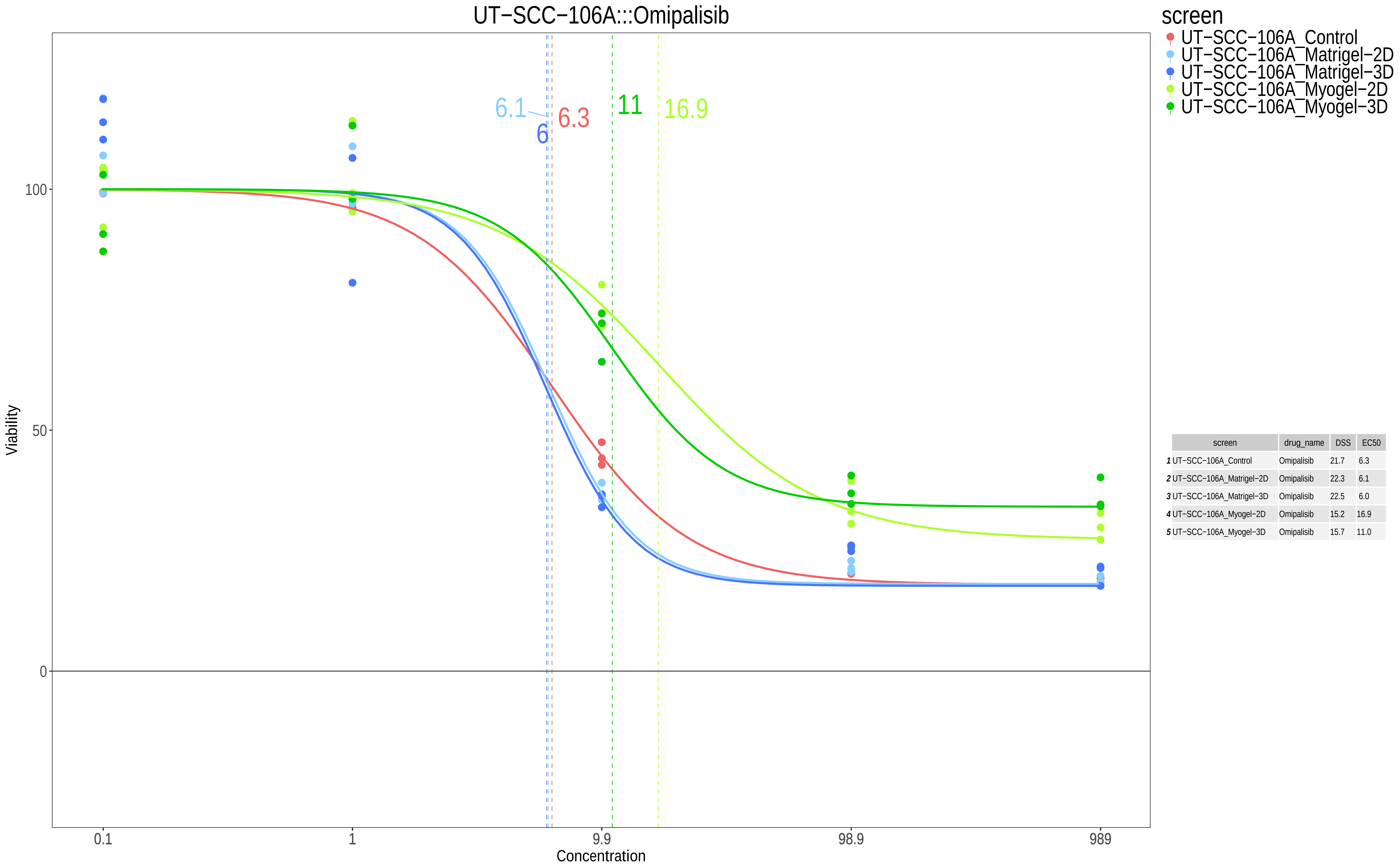

	screen	drug_name	DSS	EC50
1	UT−SCC−106A_Control	Omipalisib	21.7	6.3
2	UT−SCC−106A_Matrigel−2D	Omipalisib	22.3	6.1
3	UT−SCC−106A_Matrigel−3D	Omipalisib	22.5	6.0
4	UT−SCC−106A_Myogel−2D	Omipalisib	15.2	16.9
5	UT−SCC−106A_Myogel−3D	Omipalisib	15.7	11.0

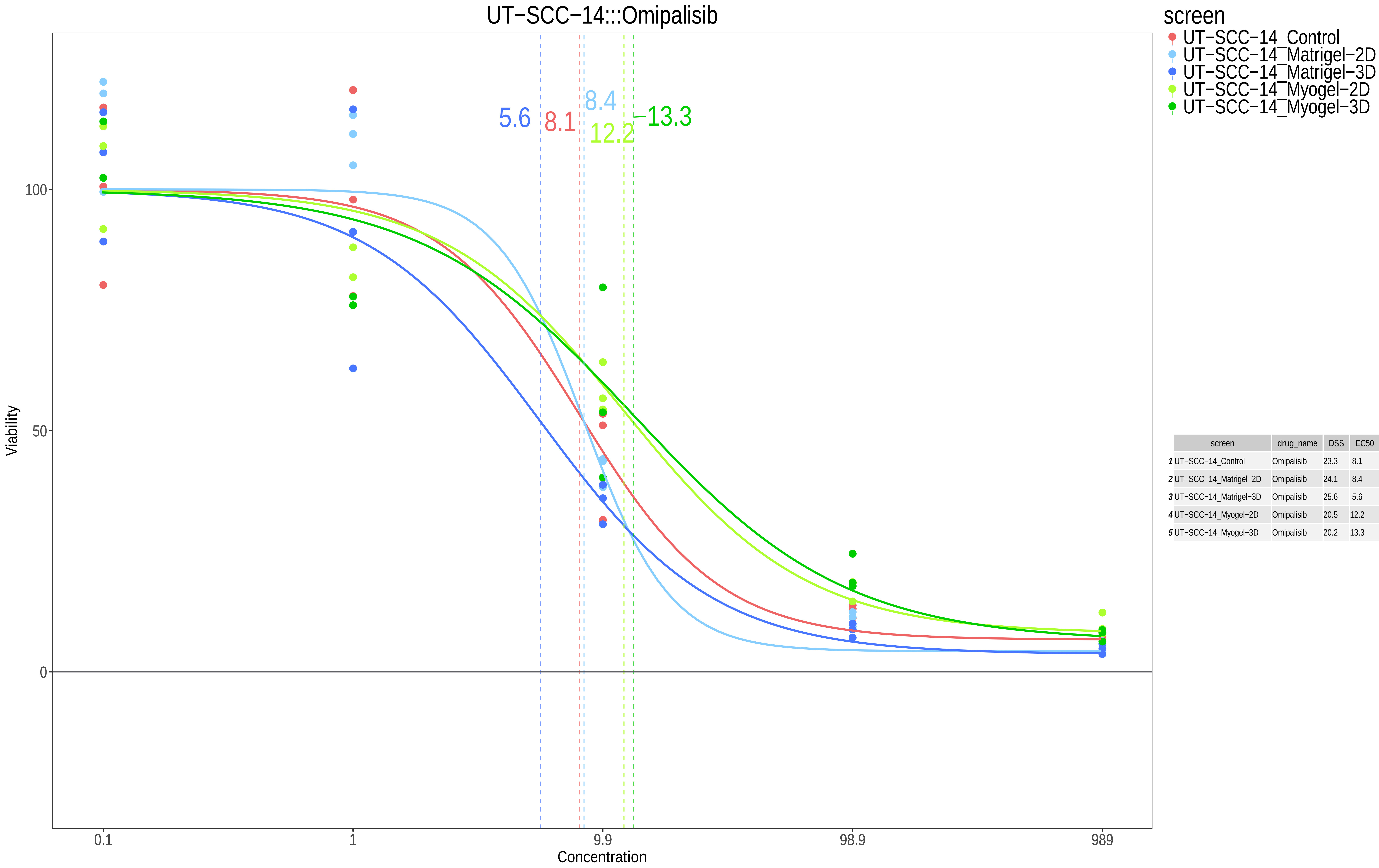

	screen	drug_name	DSS	EC50
1	UT−SCC−14_Control	Omipalisib	23.3	8.1
2	UT−SCC−14_Matrigel−2D	Omipalisib	24.1	8.4
3	UT−SCC−14_Matrigel−3D	Omipalisib	25.6	5.6
4	UT−SCC−14_Myogel−2D	Omipalisib	20.5	12.2
5	UT−SCC−14_Myogel−3D	Omipalisib	20.2	13.3

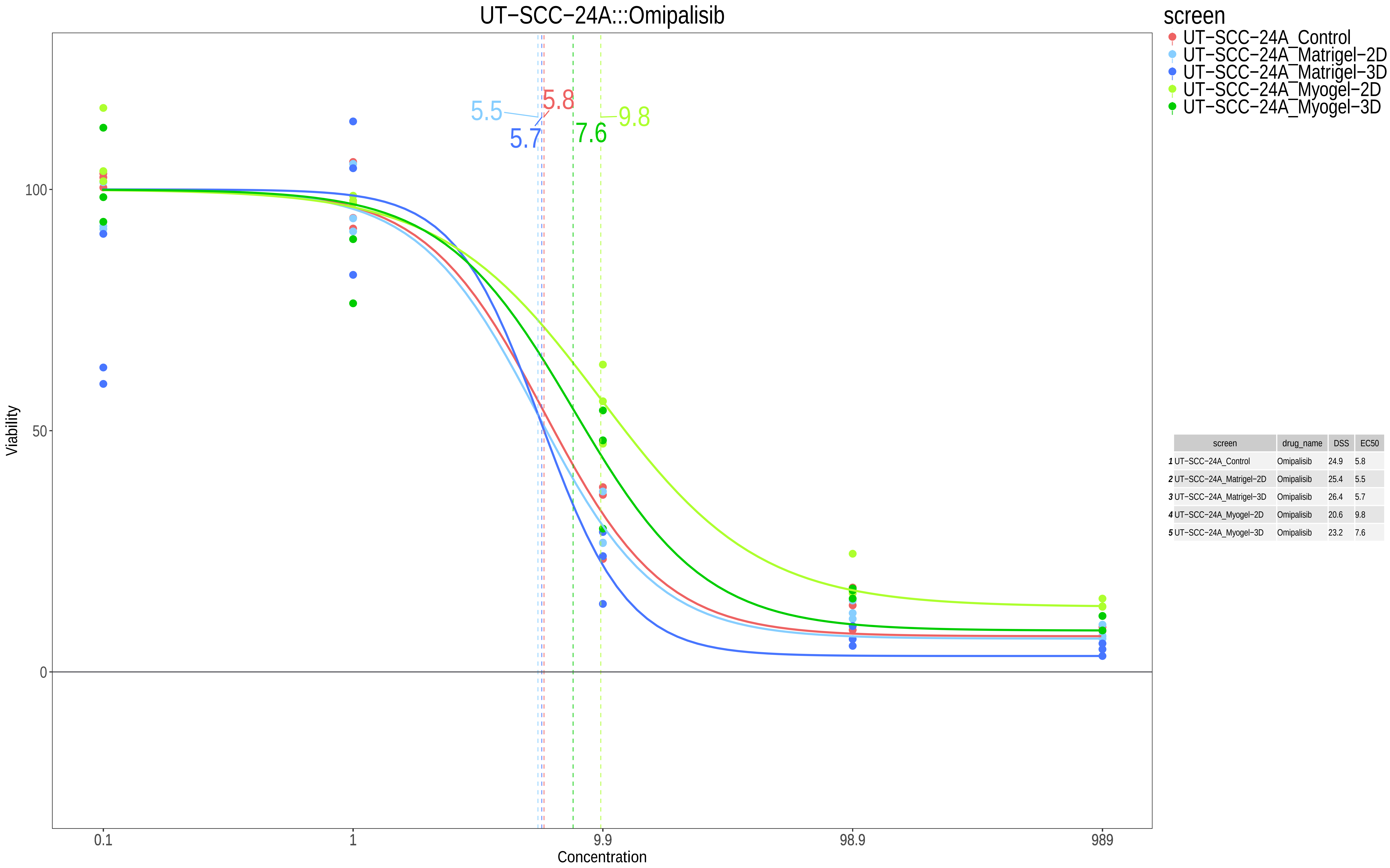

	screen	drug_name	DSS	EC50
1	UT−SCC−24A_Control	Omipalisib	24.9	5.8
2	UT−SCC−24A_Matrigel−2D	Omipalisib	25.4	5.5
3	UT−SCC−24A_Matrigel−3D	Omipalisib	26.4	5.7
4	UT−SCC−24A_Myogel−2D	Omipalisib	20.6	9.8
5	UT−SCC−24A_Myogel−3D	Omipalisib	23.2	7.6

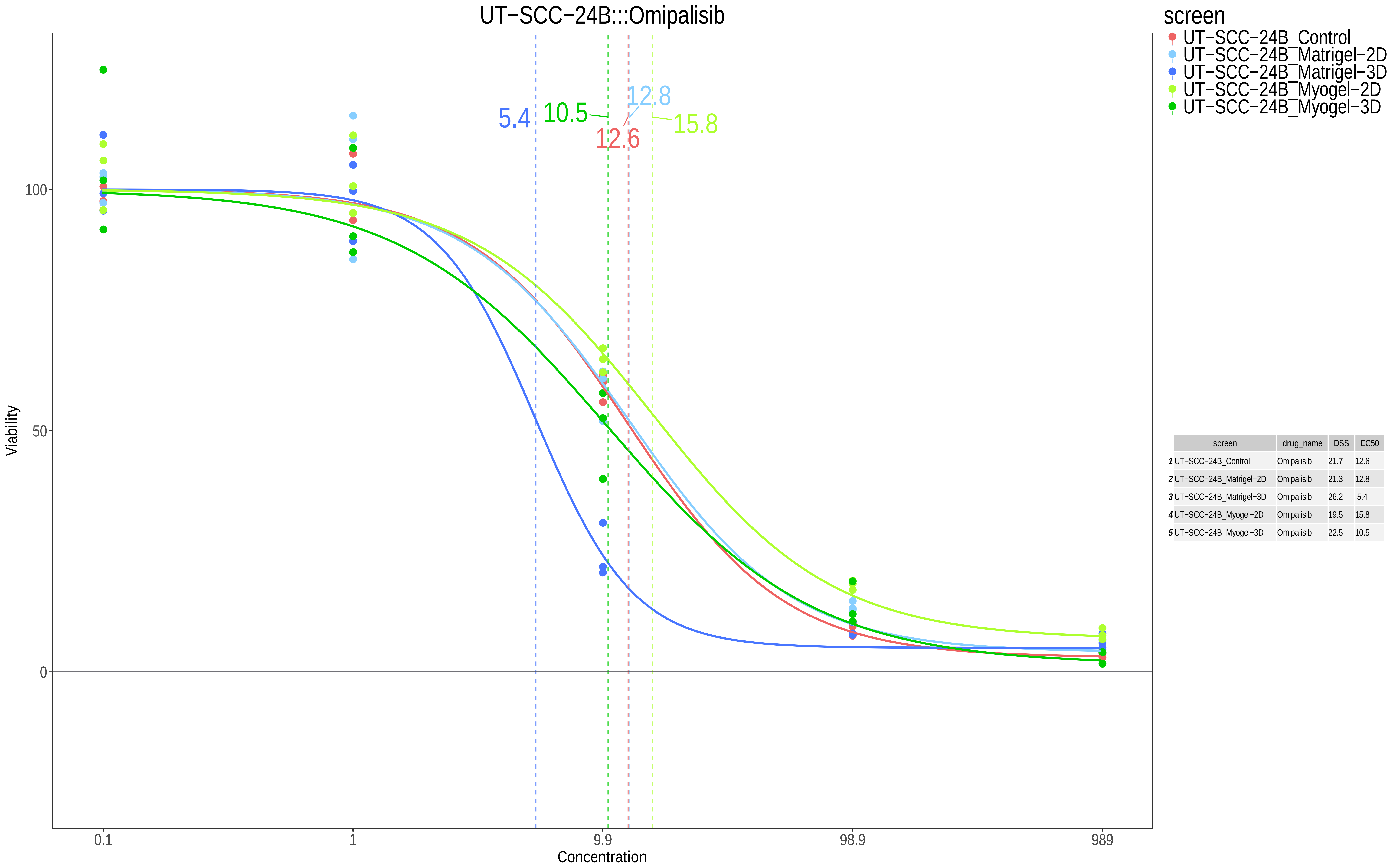

	screen	drug_name	DSS	EC50
1	UT−SCC−24B_Control	Omipalisib	21.7	12.6
2	UT−SCC−24B_Matrigel−2D	Omipalisib	21.3	12.8
3	UT−SCC−24B_Matrigel−3D	Omipalisib	26.2	5.4
4	UT−SCC−24B_Myogel−2D	Omipalisib	19.5	15.8
5	UT−SCC−24B_Myogel−3D	Omipalisib	22.5	10.5

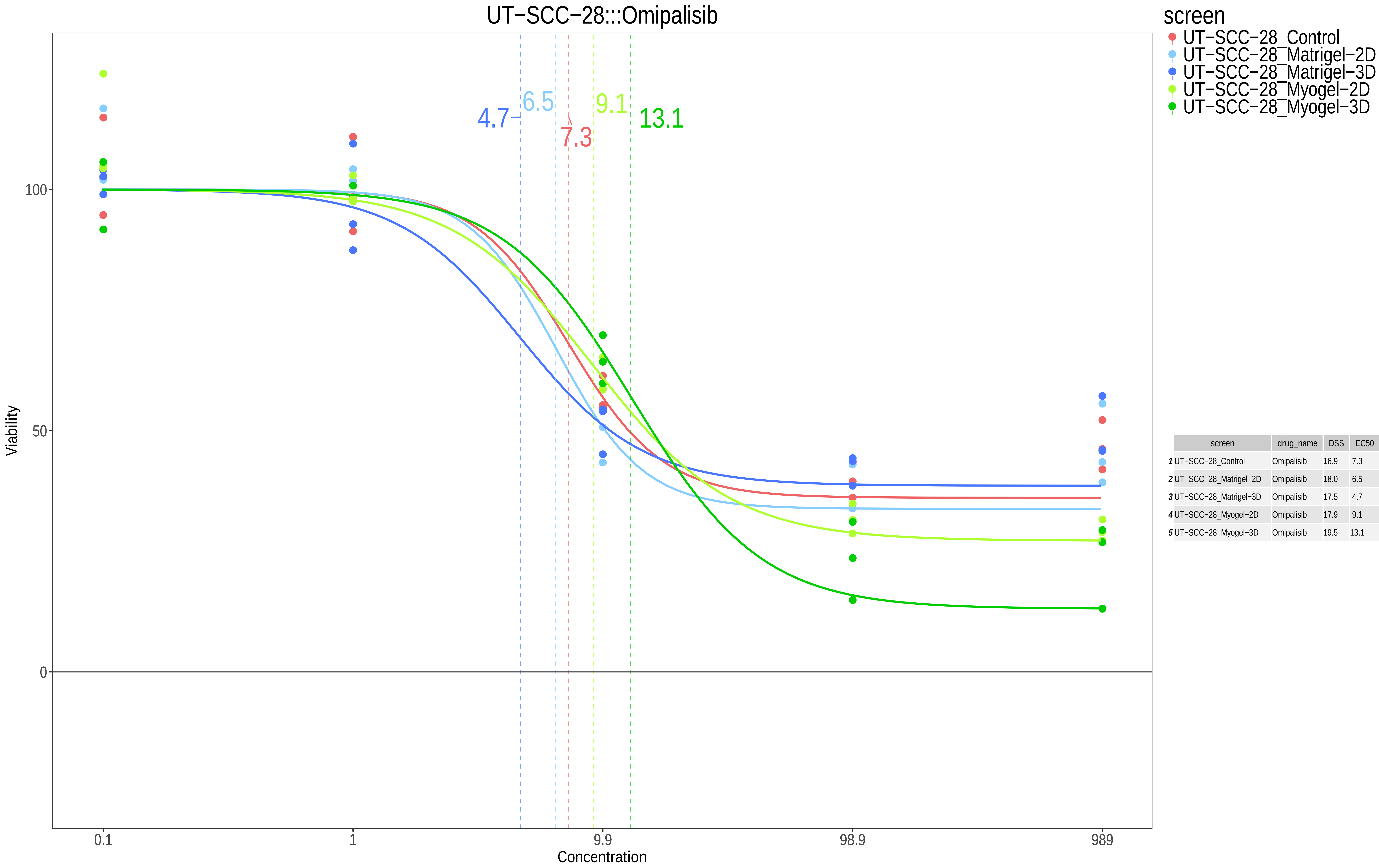

	screen	drug_name	DSS	EC50
1	UT−SCC−28_Control	Omipalisib	16.9	7.3
2	UT−SCC−28_Matrigel−2D	Omipalisib	18.0	6.5
3	UT−SCC−28_Matrigel−3D	Omipalisib	17.5	4.7
4	UT−SCC−28_Myogel−2D	Omipalisib	17.9	9.1
5	UT−SCC−28_Myogel−3D	Omipalisib	19.5	13.1

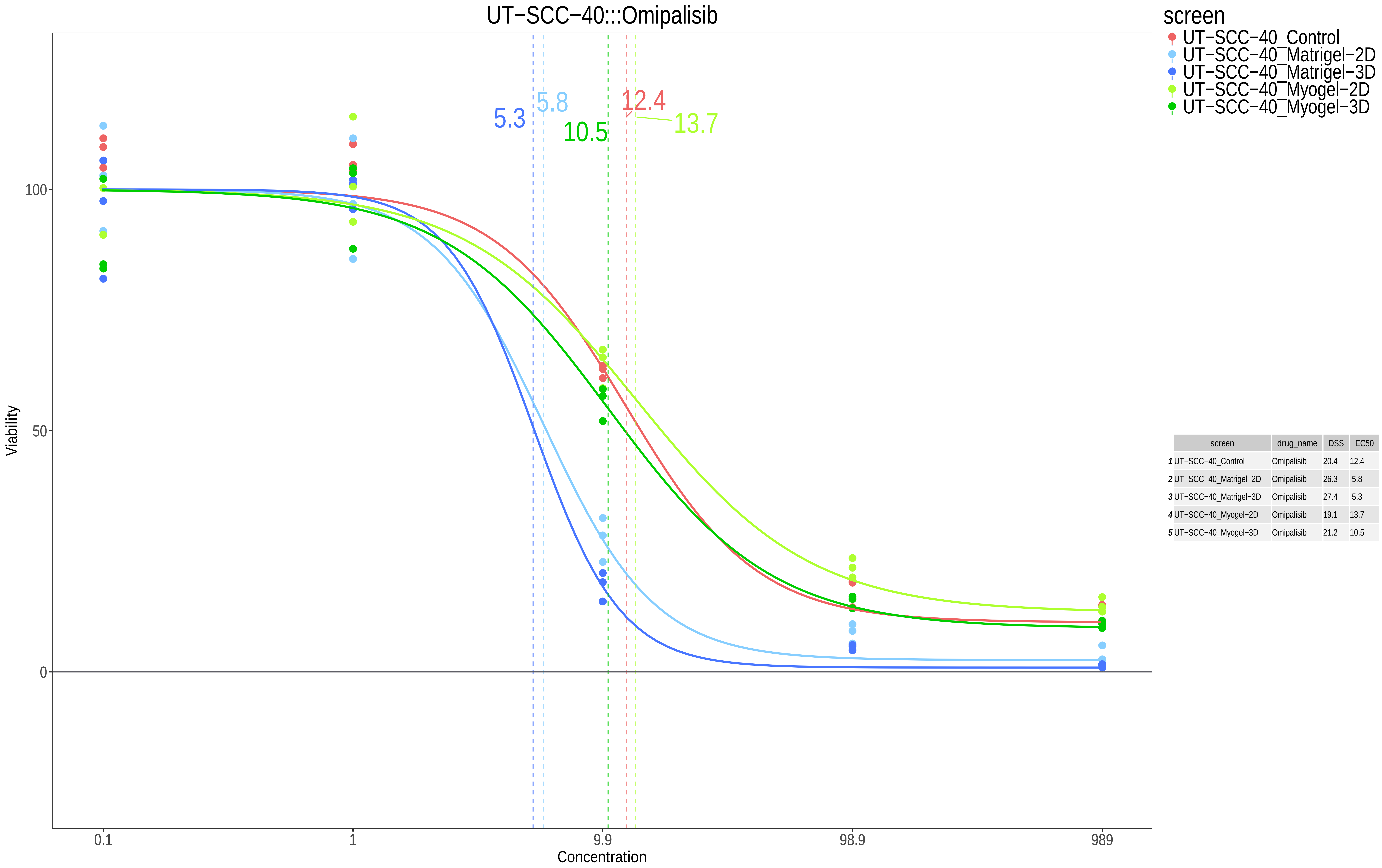

	screen	drug_name	DSS	EC50
1	UT−SCC−40_Control	Omipalisib	20.4	12.4
2	UT−SCC−40_Matrigel−2D	Omipalisib	26.3	5.8
3	UT−SCC−40_Matrigel−3D	Omipalisib	27.4	5.3
4	UT−SCC−40_Myogel−2D	Omipalisib	19.1	13.7
5	UT−SCC−40_Myogel−3D	Omipalisib	21.2	10.5

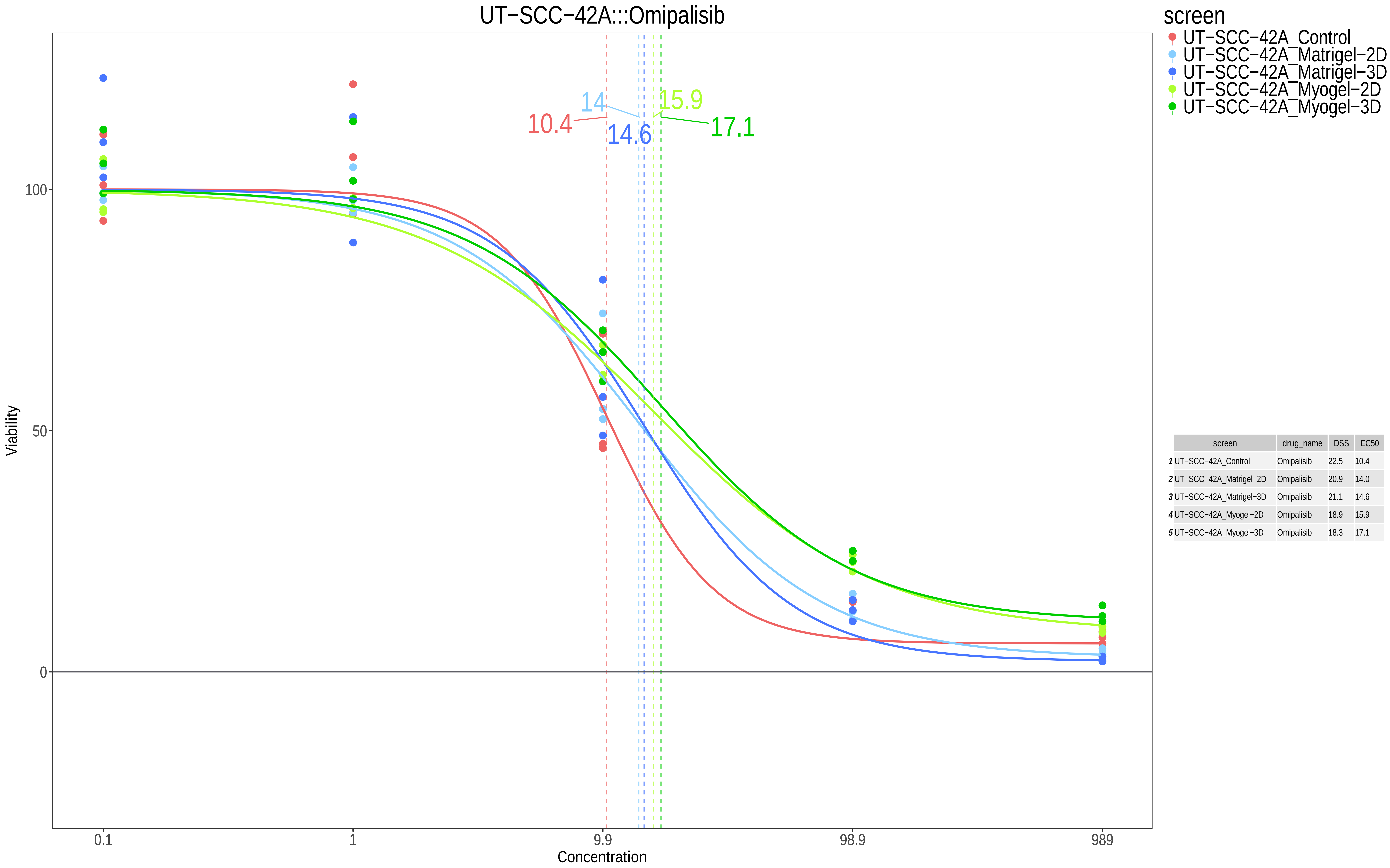

	screen	drug_name	DSS	EC50
1	UT−SCC−42A_Control	Omipalisib	22.5	10.4
2	UT−SCC−42A_Matrigel−2D	Omipalisib	20.9	14.0
3	UT−SCC−42A_Matrigel−3D	Omipalisib	21.1	14.6
4	UT−SCC−42A_Myogel−2D	Omipalisib	18.9	15.9
5	UT−SCC−42A_Myogel−3D	Omipalisib	18.3	17.1

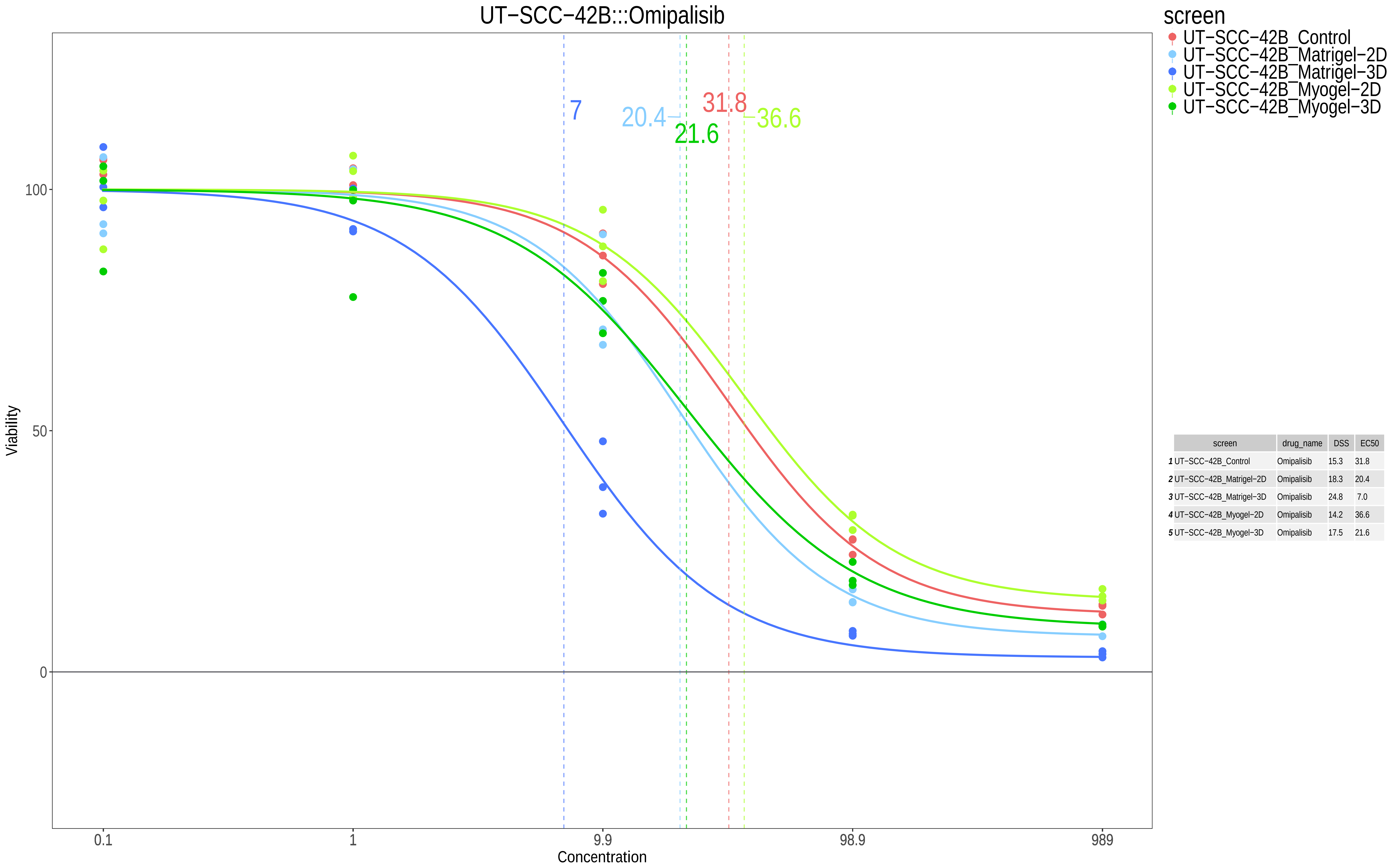

	screen	drug_name	DSS	EC50
1	UT−SCC−42B_Control	Omipalisib	15.3	31.8
2	UT−SCC−42B_Matrigel−2D	Omipalisib	18.3	20.4
3	UT−SCC−42B_Matrigel−3D	Omipalisib	24.8	7.0
4	UT−SCC−42B_Myogel−2D	Omipalisib	14.2	36.6
5	UT−SCC−42B_Myogel−3D	Omipalisib	17.5	21.6

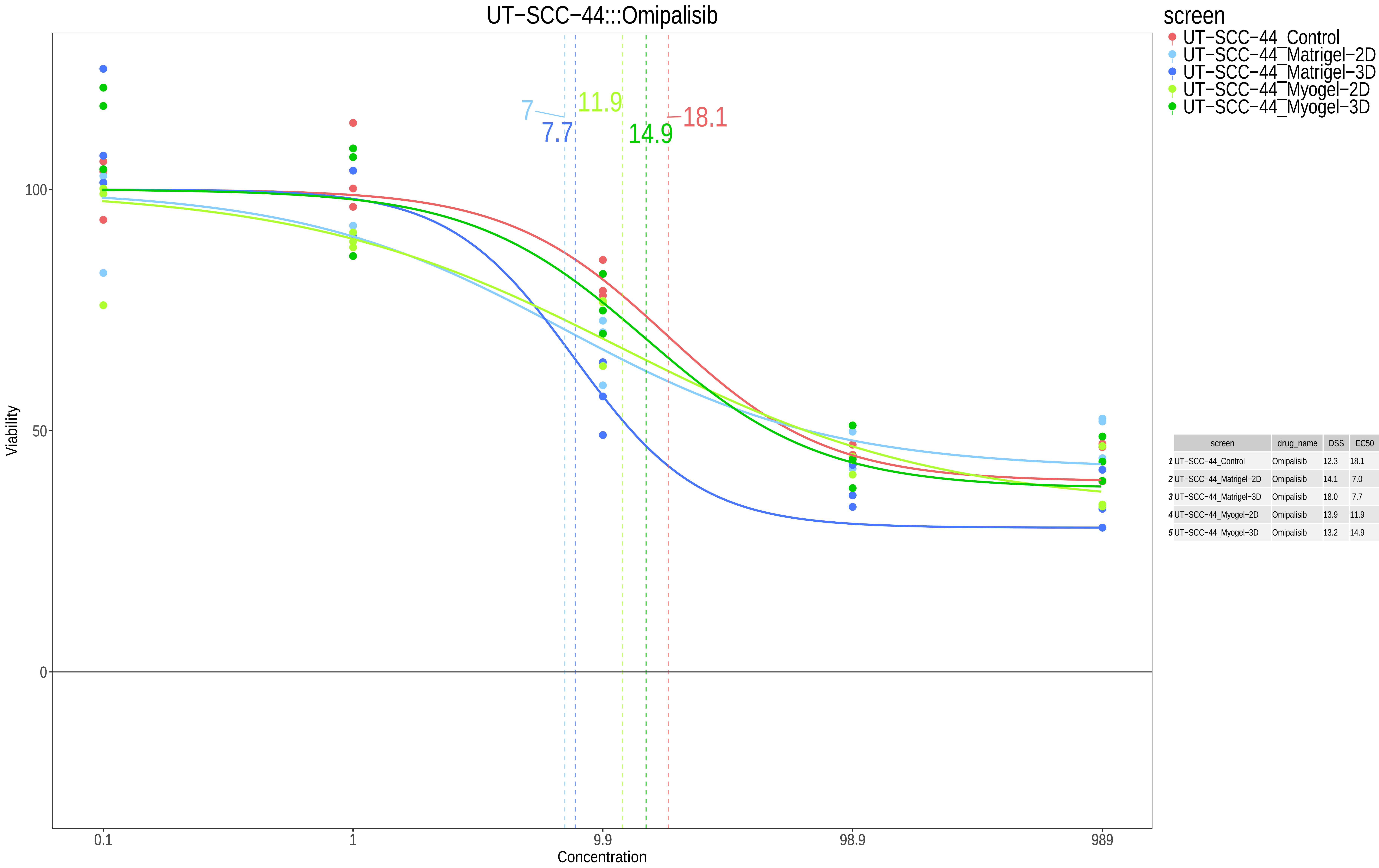

	screen	drug_name	DSS	EC50
1	UT−SCC−44_Control	Omipalisib	12.3	18.1
2	UT−SCC−44_Matrigel−2D	Omipalisib	14.1	7.0
3	UT−SCC−44_Matrigel−3D	Omipalisib	18.0	7.7
4	UT−SCC−44_Myogel−2D	Omipalisib	13.9	11.9
5	UT−SCC−44_Myogel−3D	Omipalisib	13.2	14.9

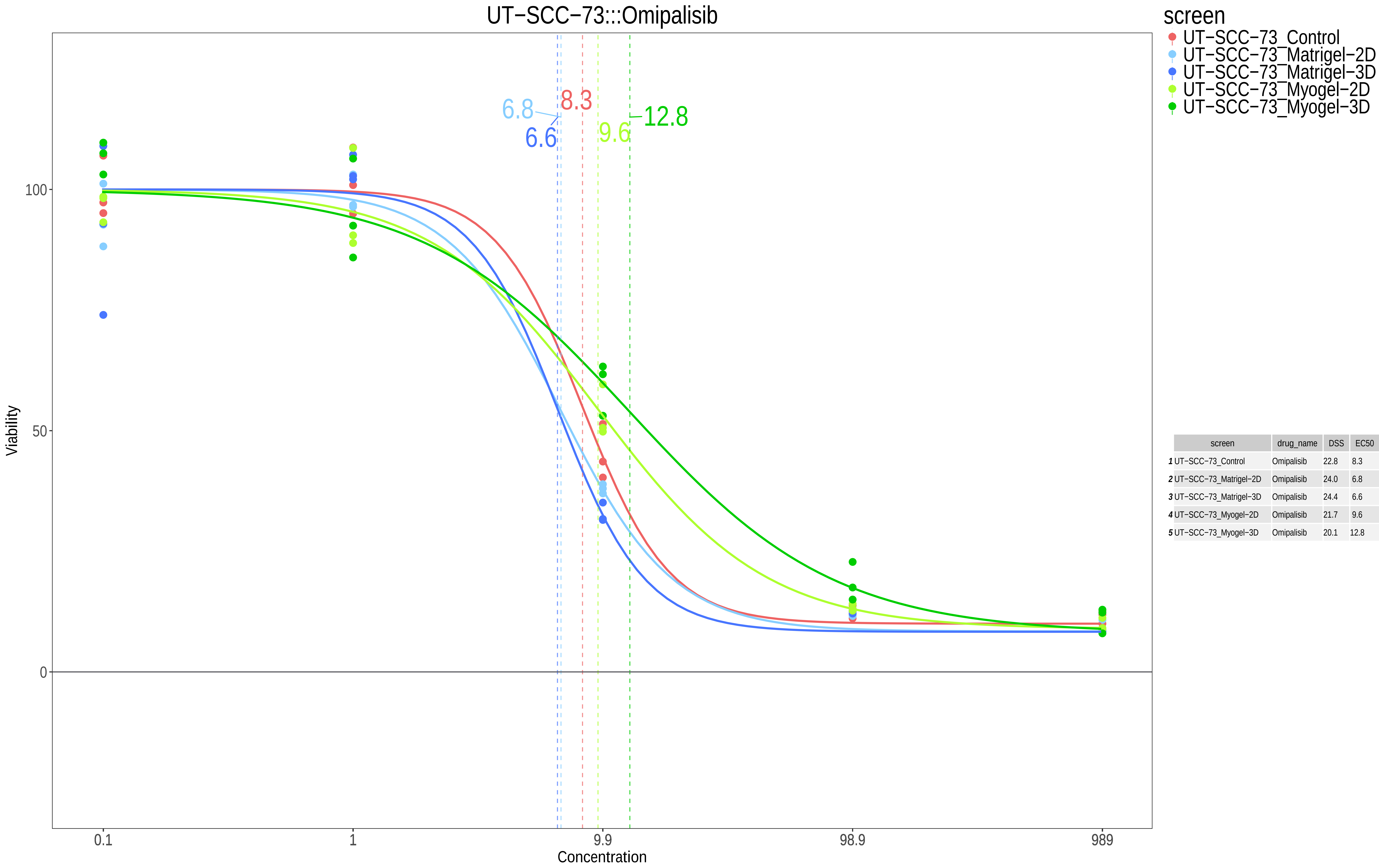

	screen	drug_name	DSS	EC50
1	UT−SCC−73_Control	Omipalisib	22.8	8.3
2	UT−SCC−73_Matrigel−2D	Omipalisib	24.0	6.8
3	UT−SCC−73_Matrigel−3D	Omipalisib	24.4	6.6
4	UT−SCC−73_Myogel−2D	Omipalisib	21.7	9.6
5	UT−SCC−73_Myogel−3D	Omipalisib	20.1	12.8

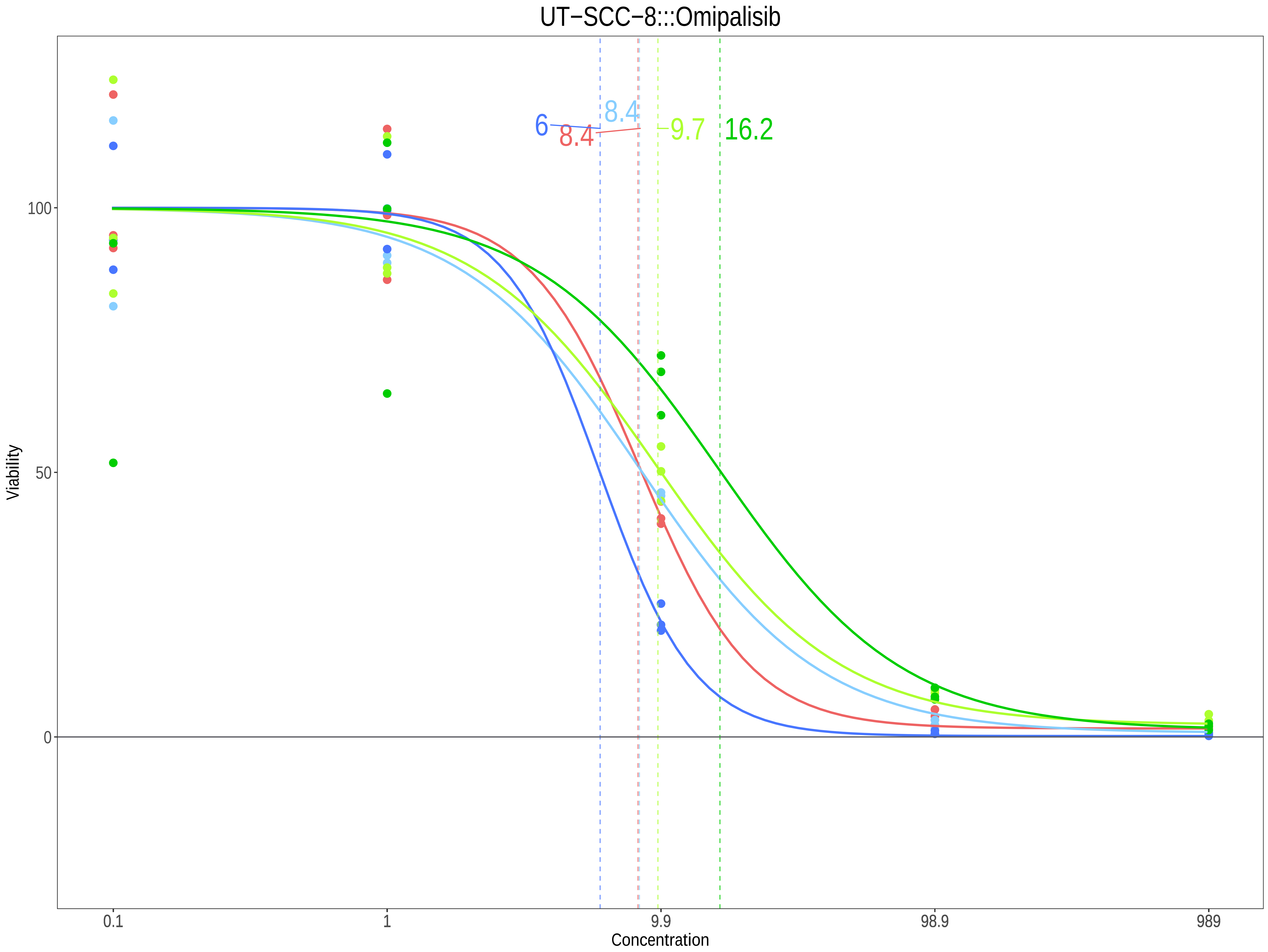

	screen	drug_name	DSS	EC50
1	UT−SCC−8_Control	Omipalisib	24.6	8.4
2	UT−SCC−8_Matrigel−2D	Omipalisib	24.2	8.4
3	UT−SCC−8_Matrigel−3D	Omipalisib	26.9	6.0
4	UT−SCC−8_Myogel−2D	Omipalisib	23.1	9.7
5	UT−SCC−8_Myogel−3D	Omipalisib	20.6	16.2

UT−SCC−81:::Omipalisib

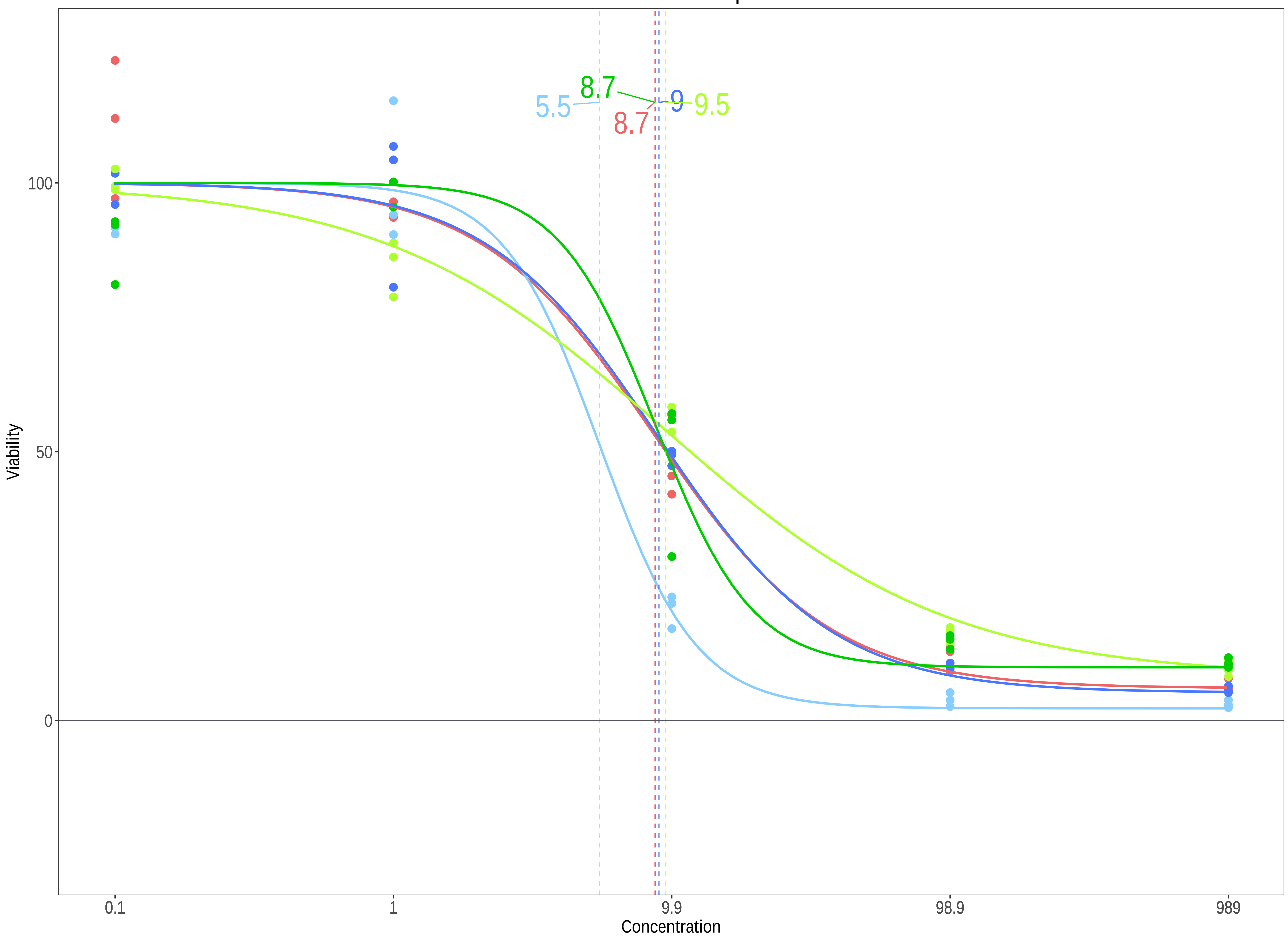

	screen	drug_name	DSS	EC50
1	UT−SCC−81_Control	Omipalisib	22.9	8.7
2	UT−SCC−81_Matrigel−2D	Omipalisib	26.8	5.5
3	UT−SCC−81_Matrigel−3D	Omipalisib	22.9	9.0
4	UT−SCC−81_Myogel−2D	Omipalisib	21.2	9.5
5	UT−SCC−81_Myogel−3D	Omipalisib	22.6	8.7

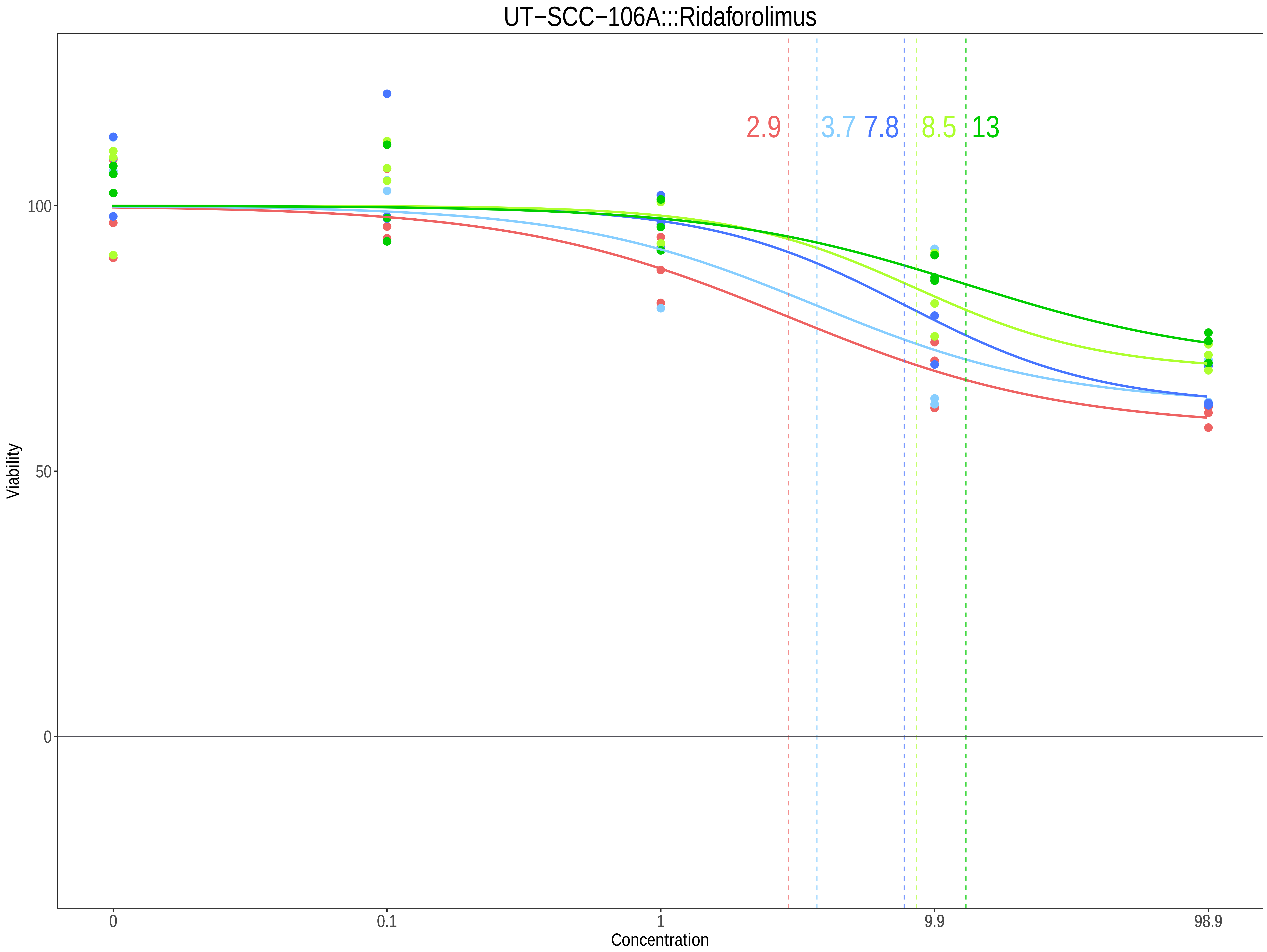

	screen	drug_name	DSS	EC50
1	UT−SCC−106A_Control	Ridaforolimus	6.5	2.9
2	UT−SCC−106A_Matrigel−2D	Ridaforolimus	5.3	3.7
3	UT−SCC−106A_Matrigel−3D	Ridaforolimus	4.1	7.8
4	UT−SCC−106A_Myogel−2D	Ridaforolimus	3.0	8.5
5	UT−SCC−106A_Myogel−3D	Ridaforolimus	2.0	13.0

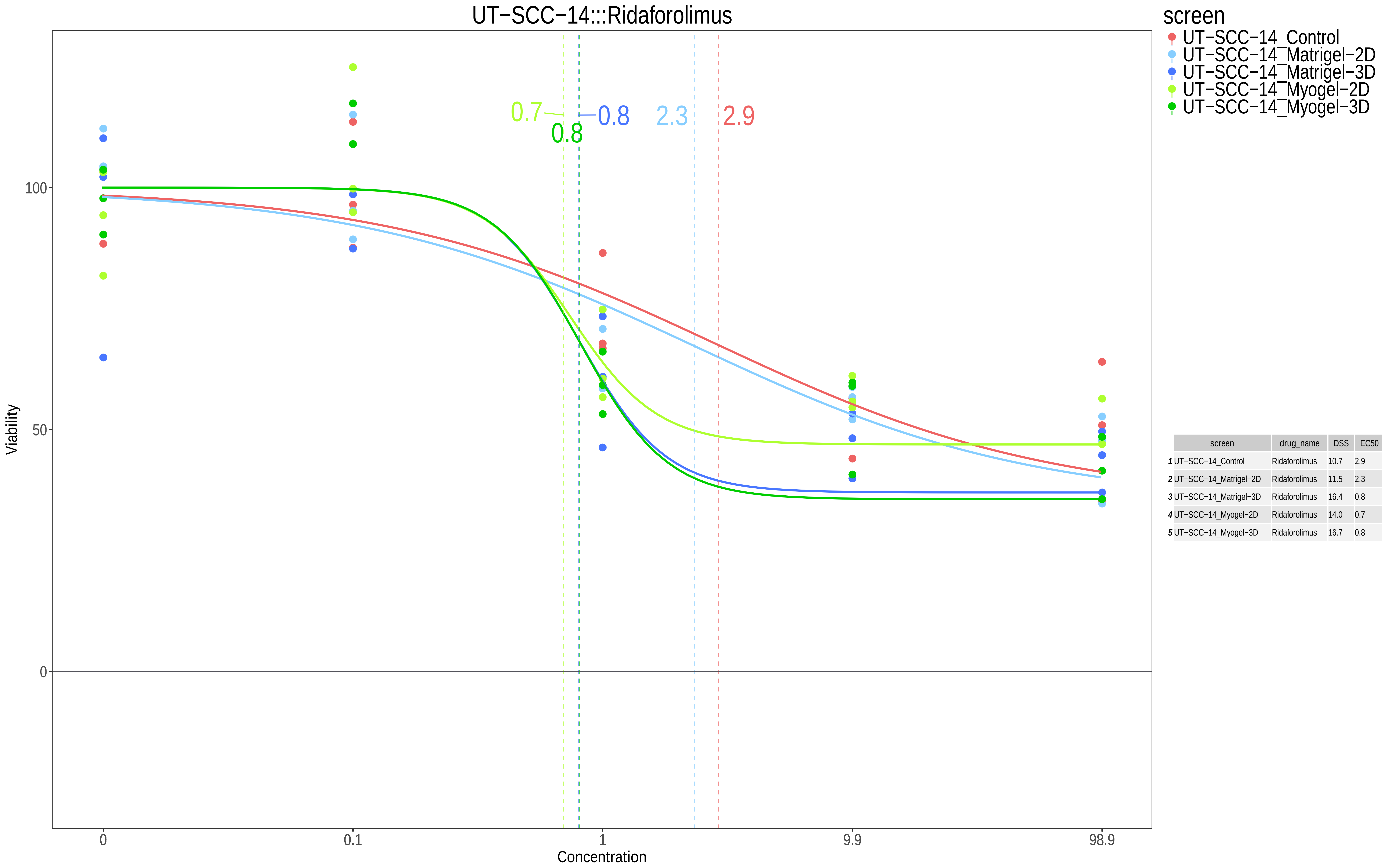

	screen	drug_name	DSS	EC50
1	UT−SCC−14_Control	Ridaforolimus	10.7	2.9
2	UT−SCC−14_Matrigel−2D	Ridaforolimus	11.5	2.3
3	UT−SCC−14_Matrigel−3D	Ridaforolimus	16.4	0.8
4	UT−SCC−14_Myogel−2D	Ridaforolimus	14.0	0.7
5	UT−SCC−14_Myogel−3D	Ridaforolimus	16.7	0.8

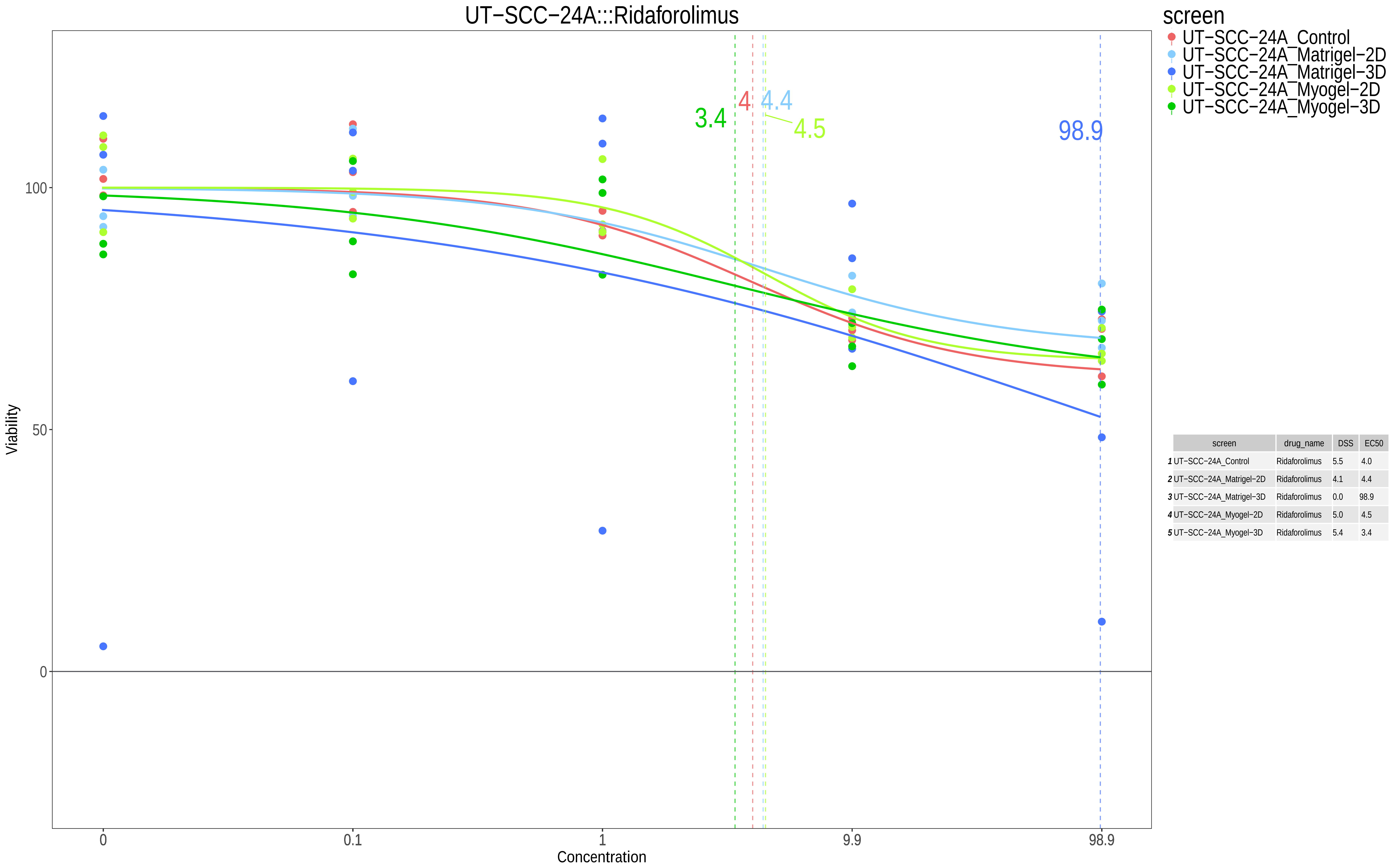

	screen	drug_name	DSS	EC50
1	UT−SCC−24A_Control	Ridaforolimus	5.5	4.0
2	UT−SCC−24A_Matrigel−2D	Ridaforolimus	4.1	4.4
3	UT−SCC−24A_Matrigel−3D	Ridaforolimus	0.0	98.9
4	UT−SCC−24A_Myogel−2D	Ridaforolimus	5.0	4.5
5	UT−SCC−24A_Myogel−3D	Ridaforolimus	5.4	3.4

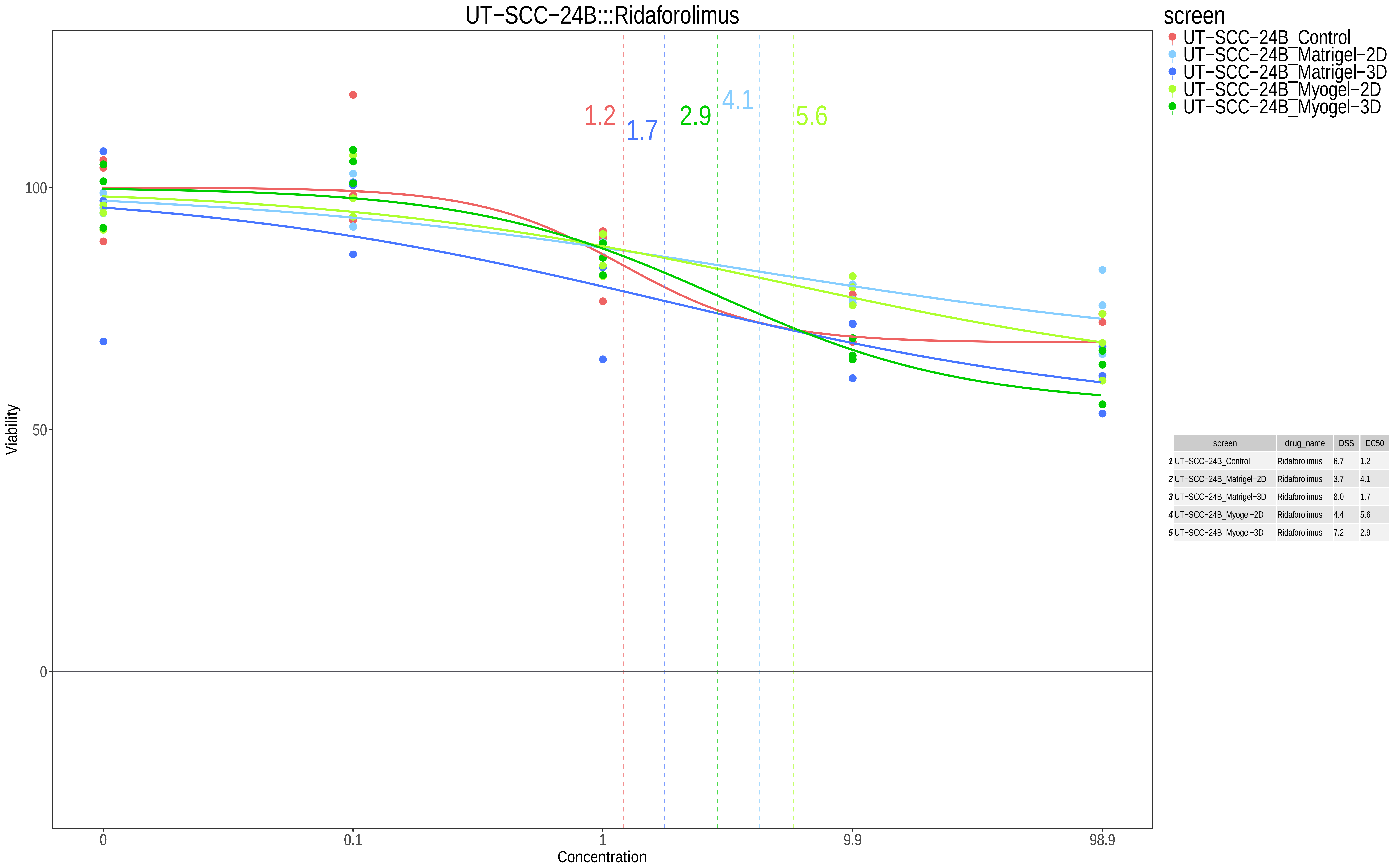

	screen	drug_name	DSS	EC50
1	UT−SCC−24B_Control	Ridaforolimus	6.7	1.2
2	UT−SCC−24B_Matrigel−2D	Ridaforolimus	3.7	4.1
3	UT−SCC−24B_Matrigel−3D	Ridaforolimus	8.0	1.7
4	UT−SCC−24B_Myogel−2D	Ridaforolimus	4.4	5.6
5	UT−SCC−24B_Myogel−3D	Ridaforolimus	7.2	2.9

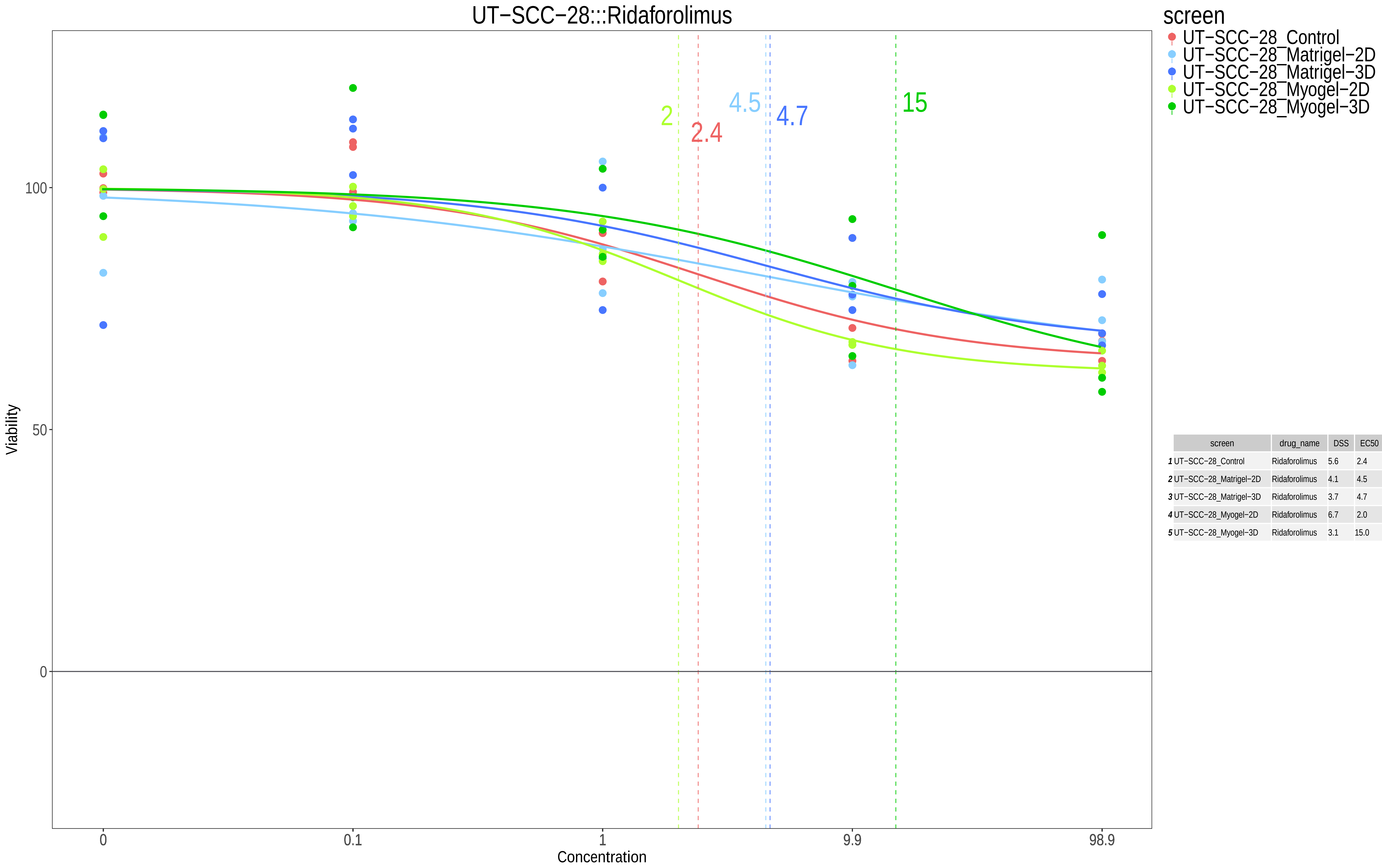

	screen	drug_name	DSS	EC50
1	UT−SCC−28_Control	Ridaforolimus	5.6	2.4
2	UT−SCC−28_Matrigel−2D	Ridaforolimus	4.1	4.5
3	UT−SCC−28_Matrigel−3D	Ridaforolimus	3.7	4.7
4	UT−SCC−28_Myogel−2D	Ridaforolimus	6.7	2.0
5	UT−SCC−28_Myogel−3D	Ridaforolimus	3.1	15.0

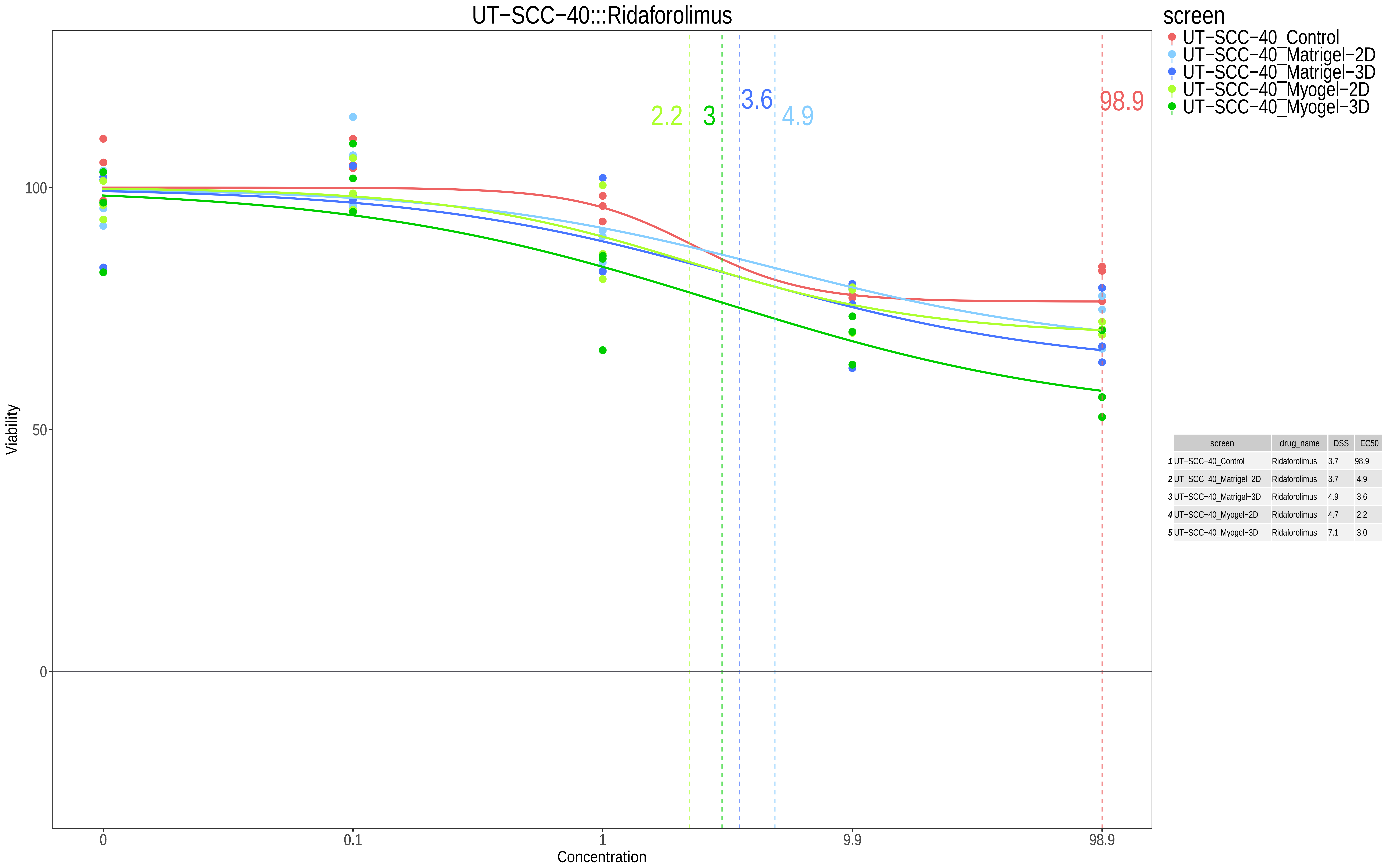

	screen	drug_name	DSS	EC50
1	UT−SCC−40_Control	Ridaforolimus	3.7	98.9
2	UT−SCC−40_Matrigel−2D	Ridaforolimus	3.7	4.9
3	UT−SCC−40_Matrigel−3D	Ridaforolimus	4.9	3.6
4	UT−SCC−40_Myogel−2D	Ridaforolimus	4.7	2.2
5	UT−SCC−40_Myogel−3D	Ridaforolimus	7.1	3.0

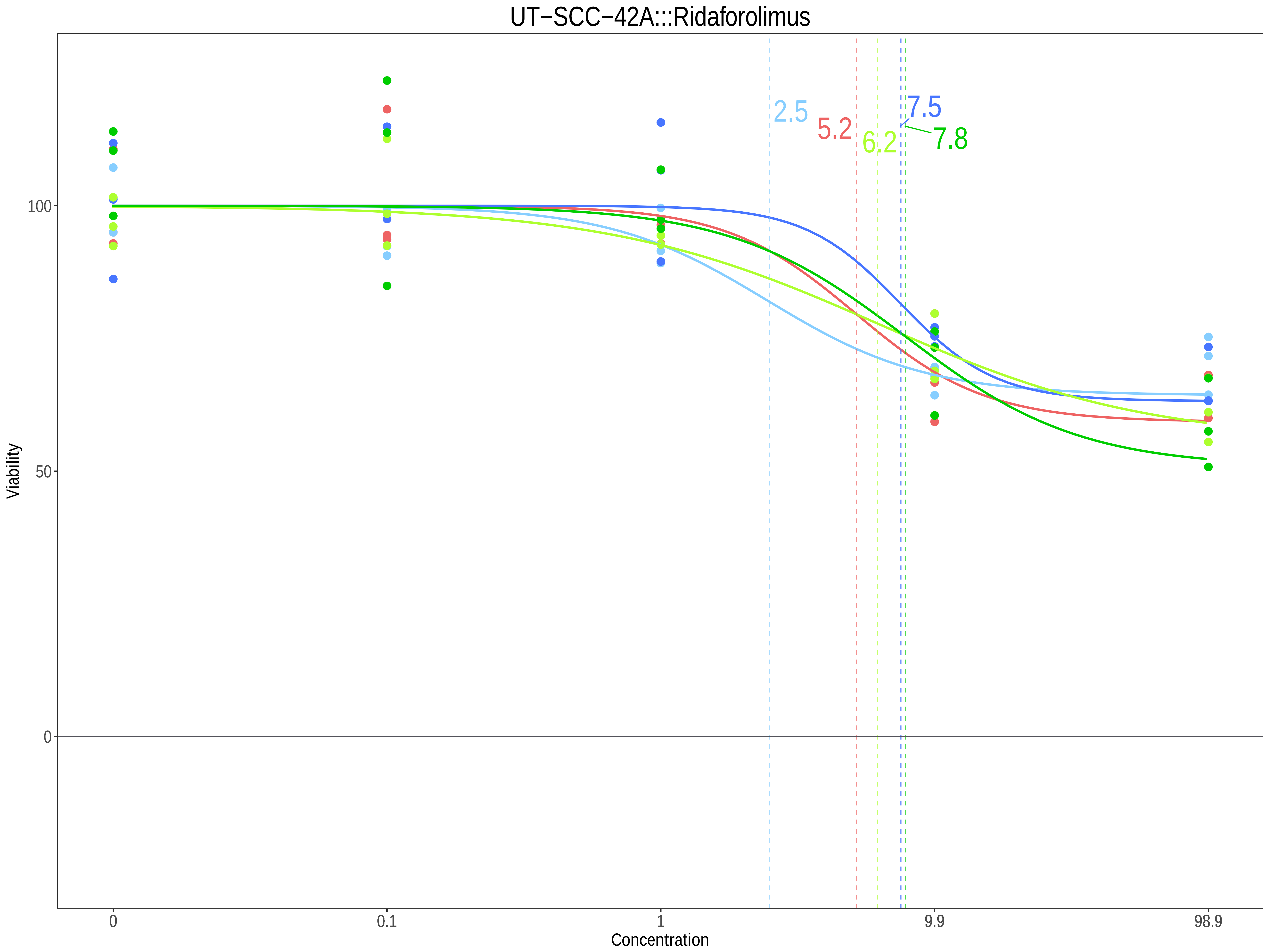

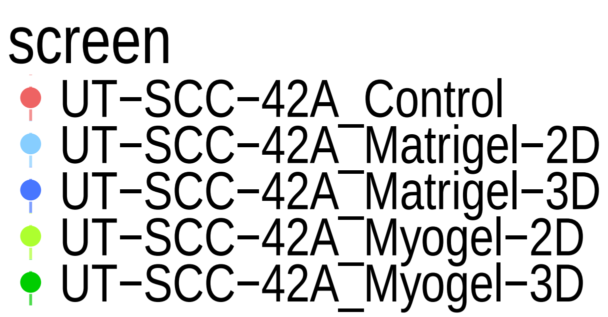

	screen	drug_name	DSS	EC50
1	UT−SCC−42A_Control	Ridaforolimus	5.9	5.2
2	UT−SCC−42A_Matrigel−2D	Ridaforolimus	6.3	2.5
3	UT−SCC−42A_Matrigel−3D	Ridaforolimus	4.7	7.5
4	UT−SCC−42A_Myogel−2D	Ridaforolimus	5.3	6.2
5	UT−SCC−42A_Myogel−3D	Ridaforolimus	5.9	7.8

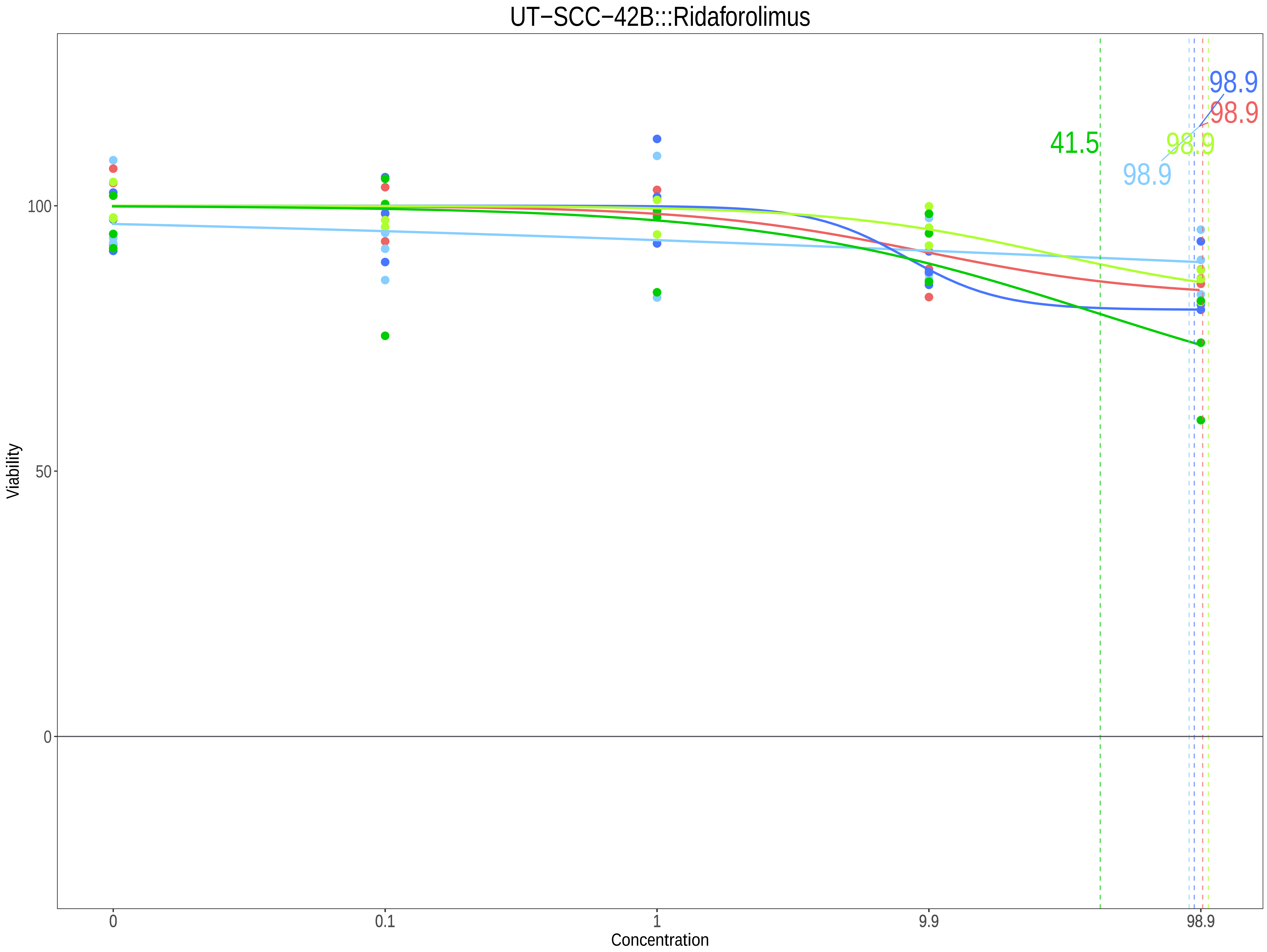

	screen	drug_name	DSS	EC50
1	UT−SCC−42B_Control	Ridaforolimus	0.7	98.9
2	UT−SCC−42B_Matrigel−2D	Ridaforolimus	0.0	98.9
3	UT−SCC−42B_Matrigel−3D	Ridaforolimus	1.7	98.9
4	UT−SCC−42B_Myogel−2D	Ridaforolimus	0.2	98.9
5	UT−SCC−42B_Myogel−3D	Ridaforolimus	1.4	41.5

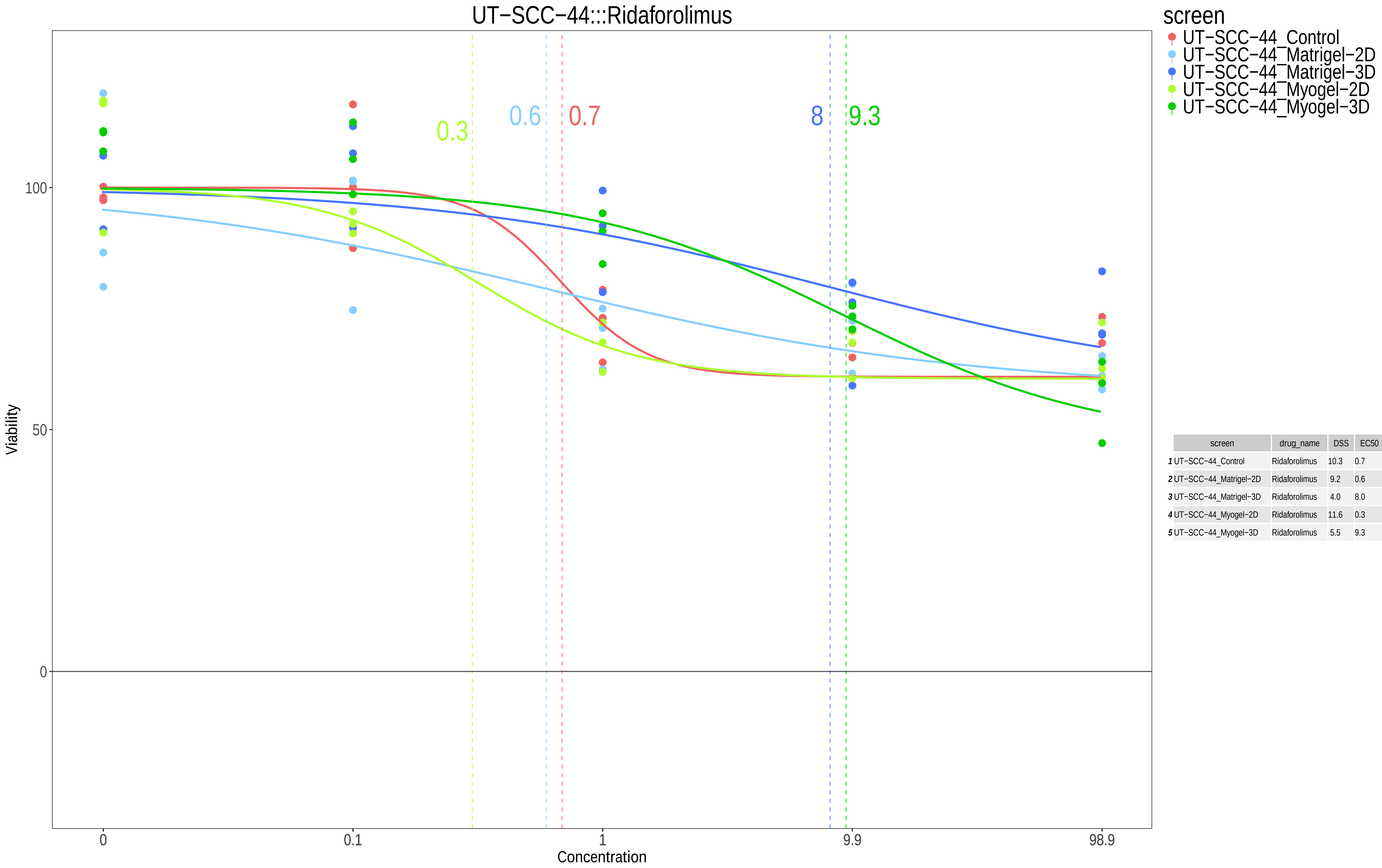

	screen	drug_name	DSS	EC50
1	UT−SCC−44_Control	Ridaforolimus	10.3	0.7
2	UT−SCC−44_Matrigel−2D	Ridaforolimus	9.2	0.6
3	UT−SCC−44_Matrigel−3D	Ridaforolimus	4.0	8.0
4	UT−SCC−44_Myogel−2D	Ridaforolimus	11.6	0.3
5	UT−SCC−44_Myogel−3D	Ridaforolimus	5.5	9.3

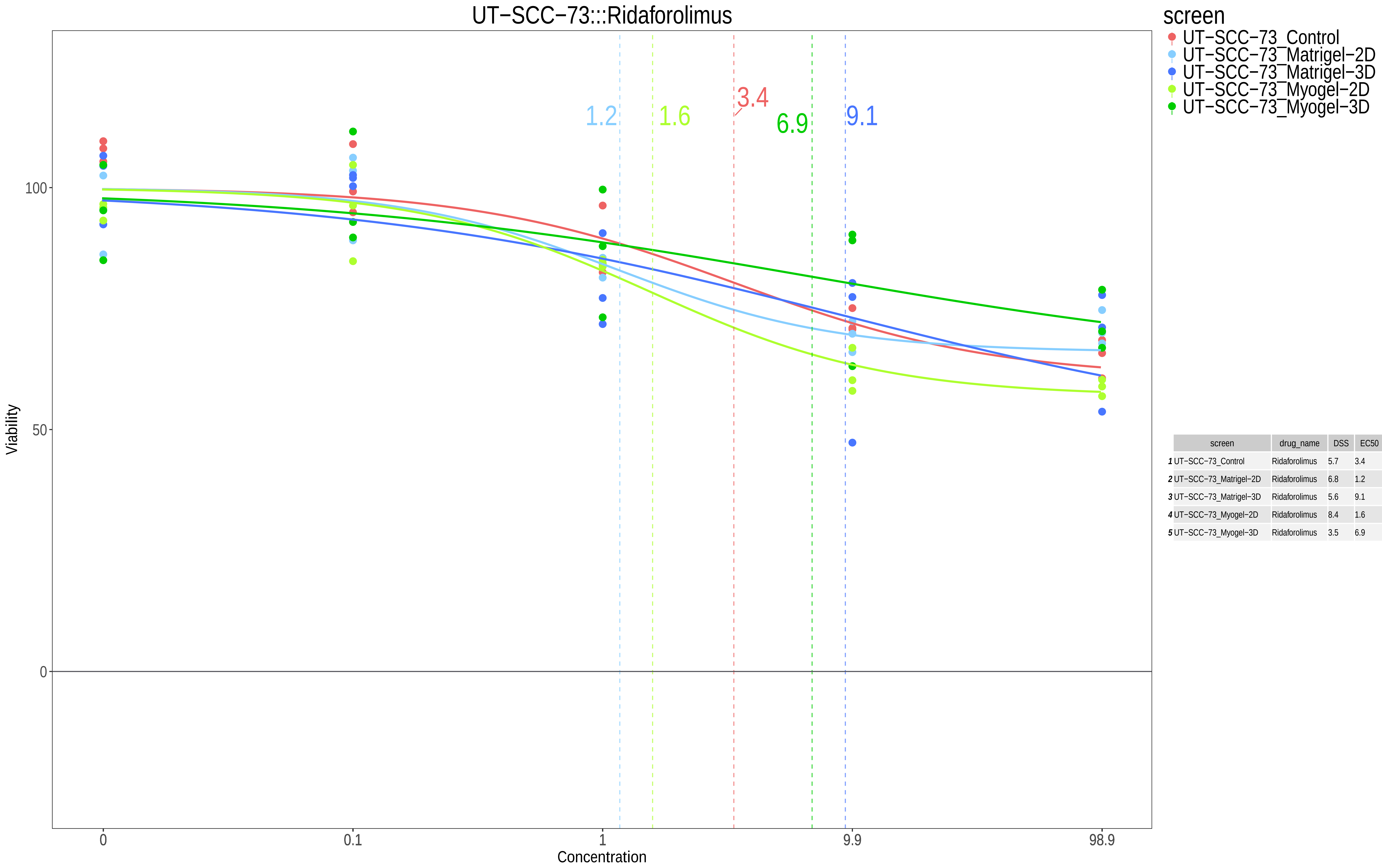

	screen	drug_name	DSS	EC50
1	UT−SCC−73_Control	Ridaforolimus	5.7	3.4
2	UT−SCC−73_Matrigel−2D	Ridaforolimus	6.8	1.2
3	UT−SCC−73_Matrigel−3D	Ridaforolimus	5.6	9.1
4	UT−SCC−73_Myogel−2D	Ridaforolimus	8.4	1.6
5	UT−SCC−73_Myogel−3D	Ridaforolimus	3.5	6.9

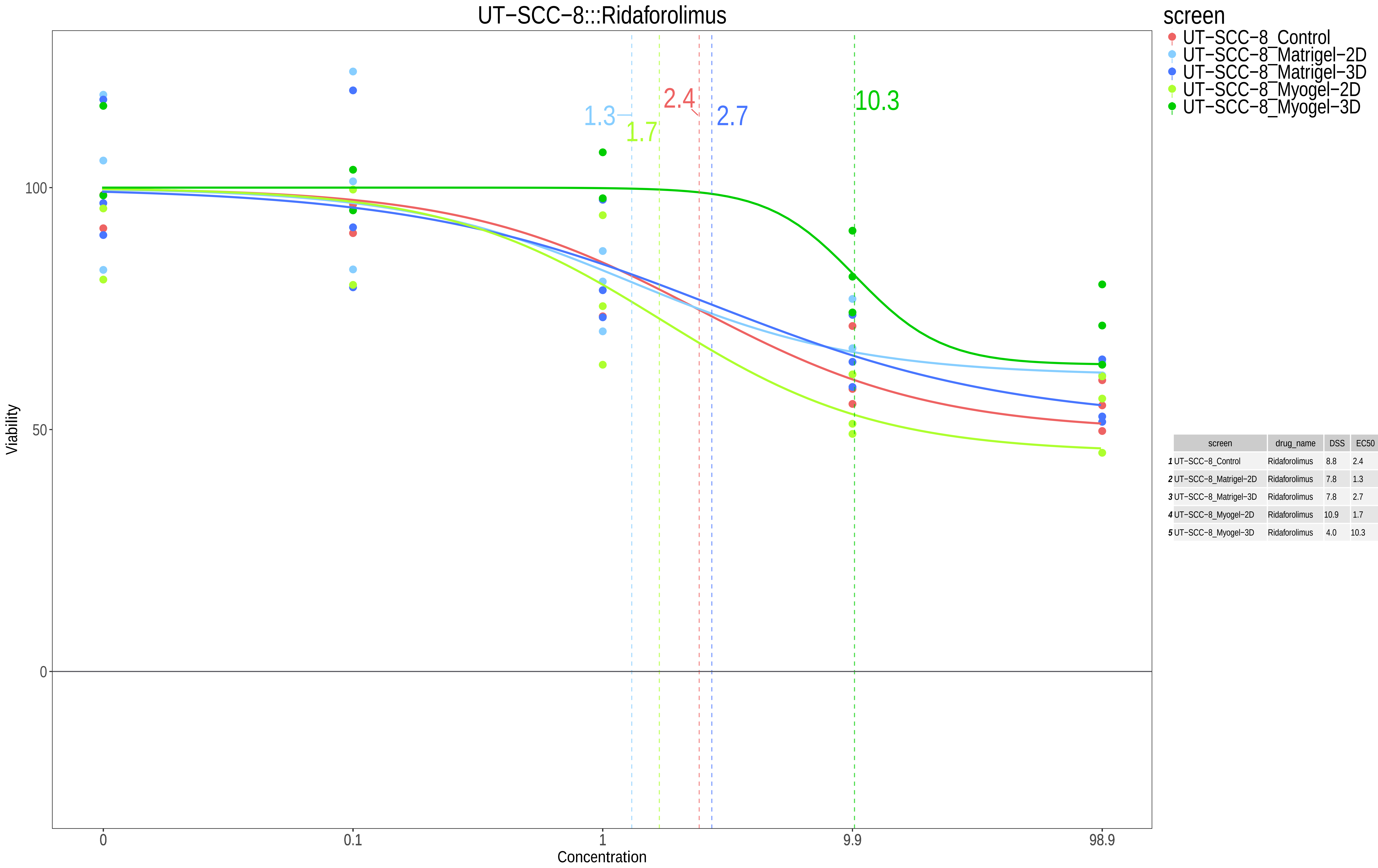

	screen	drug_name	DSS	EC50
1	UT−SCC−8_Control	Ridaforolimus	8.8	2.4
2	UT−SCC−8_Matrigel−2D	Ridaforolimus	7.8	1.3
3	UT−SCC−8_Matrigel−3D	Ridaforolimus	7.8	2.7
4	UT−SCC−8_Myogel−2D	Ridaforolimus	10.9	1.7
5	UT−SCC−8_Myogel−3D	Ridaforolimus	4.0	10.3

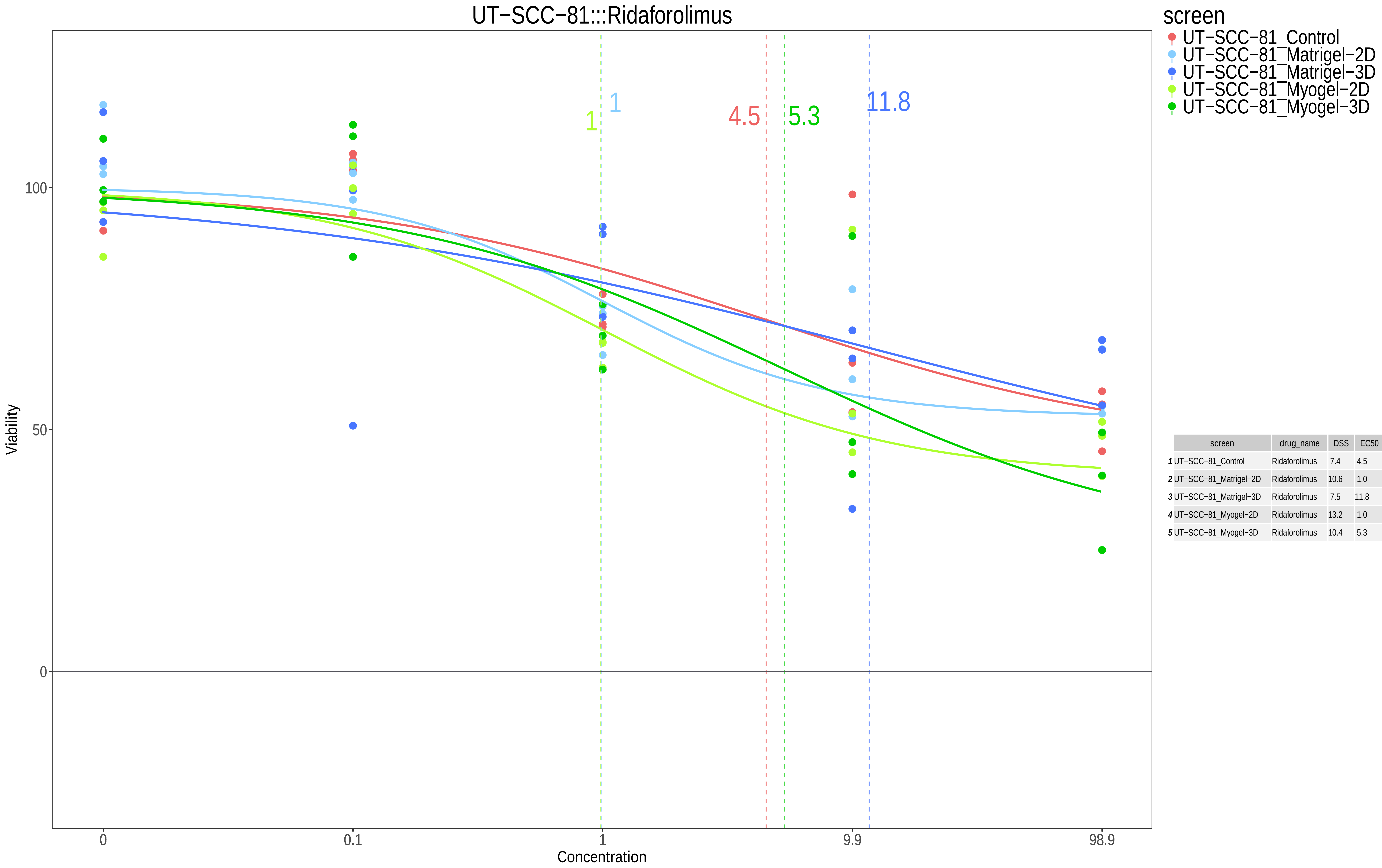

	screen	drug_name	DSS	EC50
1	UT−SCC−81_Control	Ridaforolimus	7.4	4.5
2	UT−SCC−81_Matrigel−2D	Ridaforolimus	10.6	1.0
3	UT−SCC−81_Matrigel−3D	Ridaforolimus	7.5	11.8
4	UT−SCC−81_Myogel−2D	Ridaforolimus	13.2	1.0
5	UT−SCC−81_Myogel−3D	Ridaforolimus	10.4	5.3

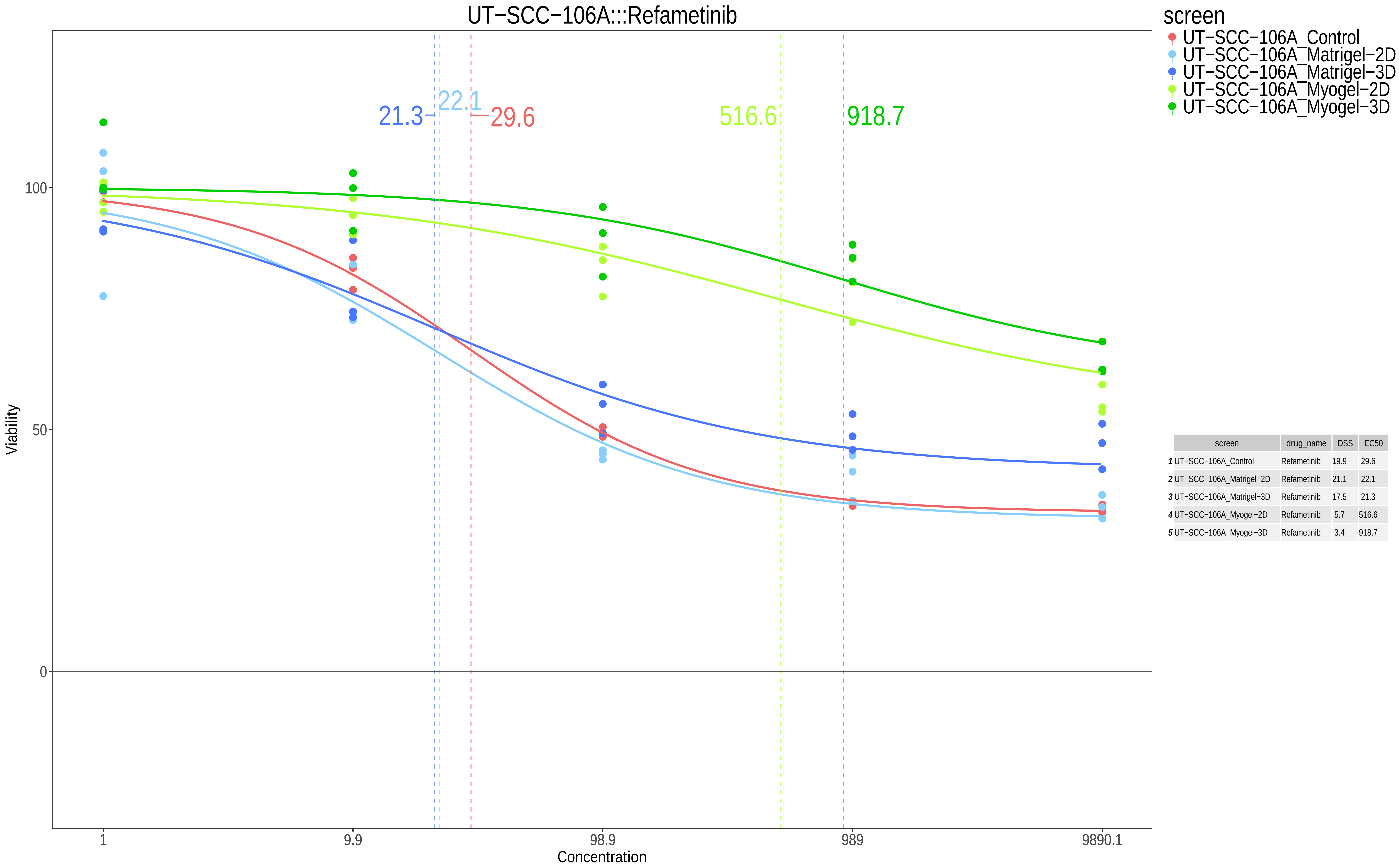

	screen	drug_name	DSS	EC50
1	UT−SCC−106A_Control	Refametinib	19.9	29.6
2	UT−SCC−106A_Matrigel−2D	Refametinib	21.1	22.1
3	UT−SCC−106A_Matrigel−3D	Refametinib	17.5	21.3
4	UT−SCC−106A_Myogel−2D	Refametinib	5.7	516.6
5	UT−SCC−106A_Myogel−3D	Refametinib	3.4	918.7

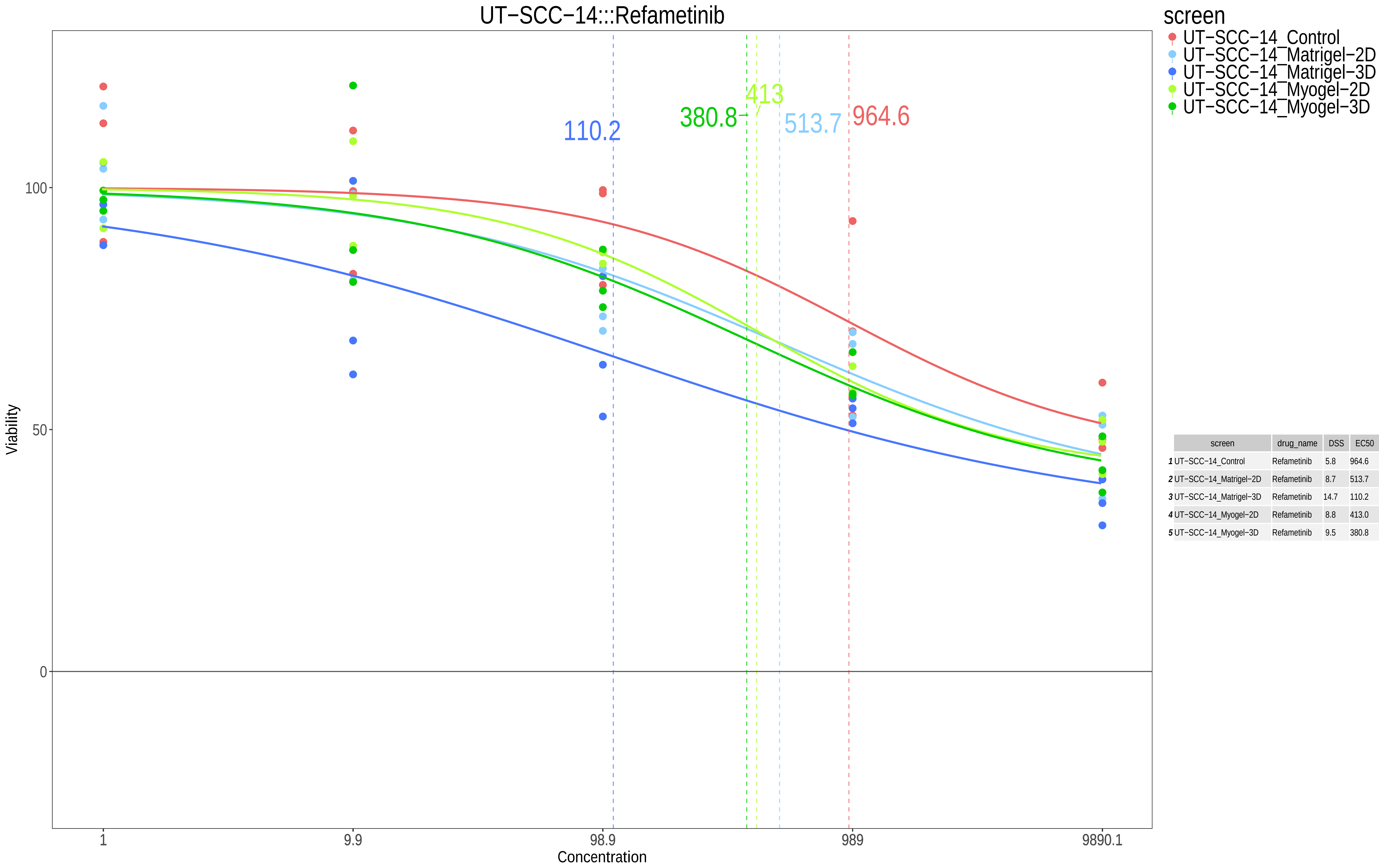

	screen	drug_name	DSS	EC50
1	UT−SCC−14_Control	Refametinib	5.8	964.6
2	UT−SCC−14_Matrigel−2D	Refametinib	8.7	513.7
3	UT−SCC−14_Matrigel−3D	Refametinib	14.7	110.2
4	UT−SCC−14_Myogel−2D	Refametinib	8.8	413.0
5	UT−SCC−14_Myogel−3D	Refametinib	9.5	380.8

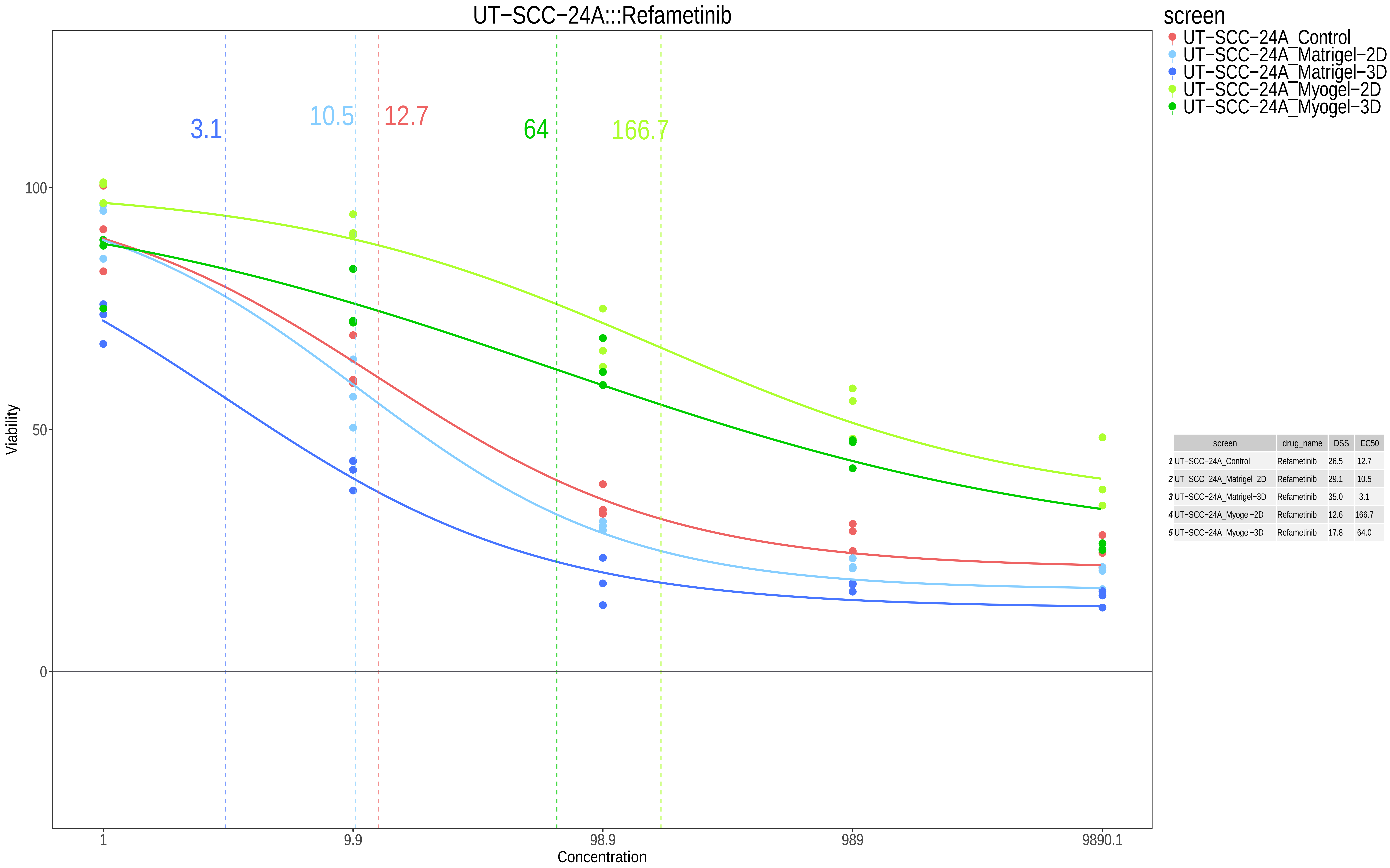

	screen	drug_name	DSS	EC50
1	UT−SCC−24A_Control	Refametinib	26.5	12.7
2	UT−SCC−24A_Matrigel−2D	Refametinib	29.1	10.5
3	UT−SCC−24A_Matrigel−3D	Refametinib	35.0	3.1
4	UT−SCC−24A_Myogel−2D	Refametinib	12.6	166.7
5	UT−SCC−24A_Myogel−3D	Refametinib	17.8	64.0

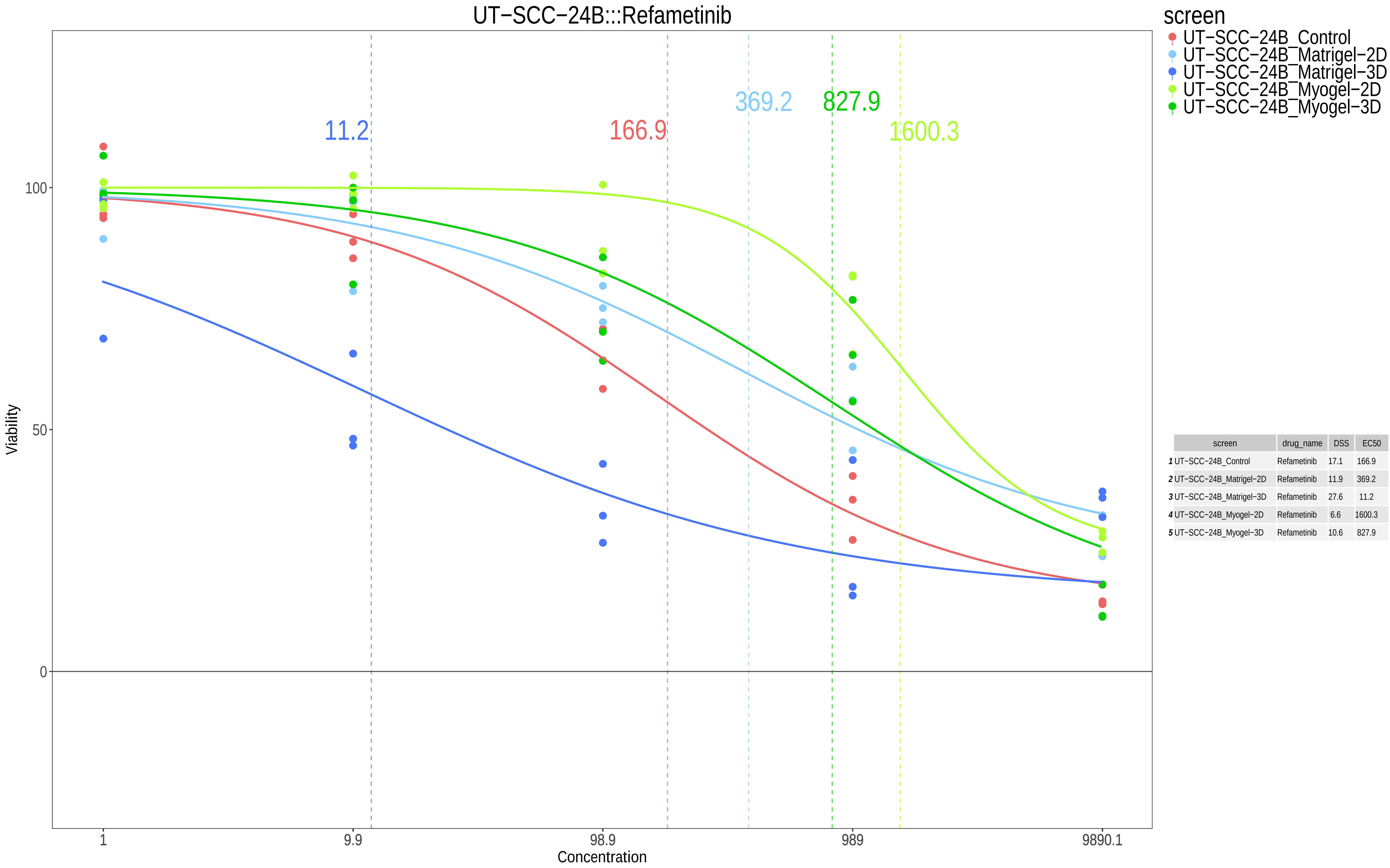

	screen	drug_name	DSS	EC50
1	UT−SCC−24B_Control	Refametinib	17.1	166.9
2	UT−SCC−24B_Matrigel−2D	Refametinib	11.9	369.2
3	UT−SCC−24B_Matrigel−3D	Refametinib	27.6	11.2
4	UT−SCC−24B_Myogel−2D	Refametinib	6.6	1600.3
5	UT−SCC−24B_Myogel−3D	Refametinib	10.6	827.9

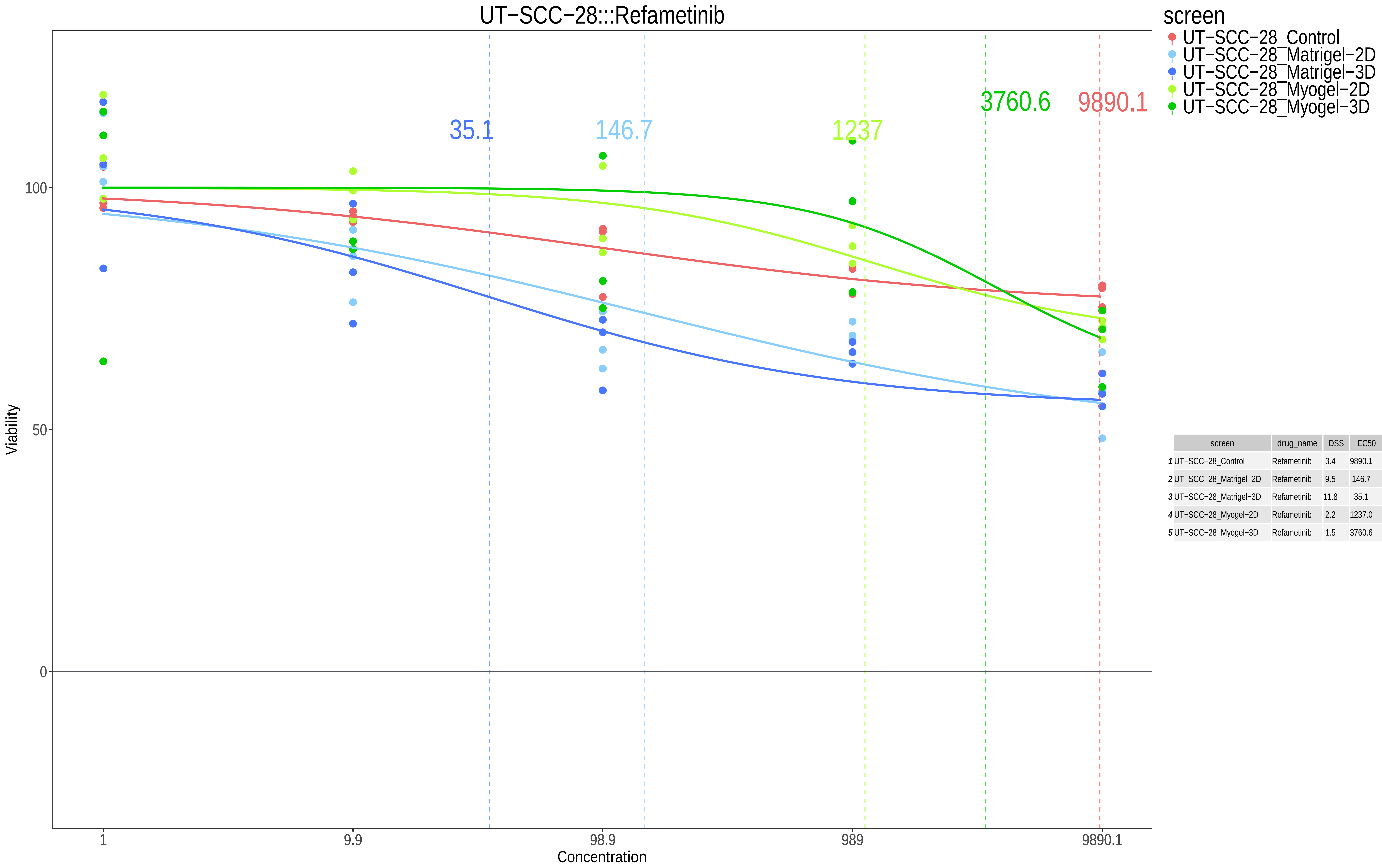

	screen	drug_name	DSS	EC50
1	UT−SCC−28_Control	Refametinib	3.4	9890.1
2	UT−SCC−28_Matrigel−2D	Refametinib	9.5	146.7
3	UT−SCC−28_Matrigel−3D	Refametinib	11.8	35.1
4	UT−SCC−28_Myogel−2D	Refametinib	2.2	1237.0
5	UT−SCC−28_Myogel−3D	Refametinib	1.5	3760.6

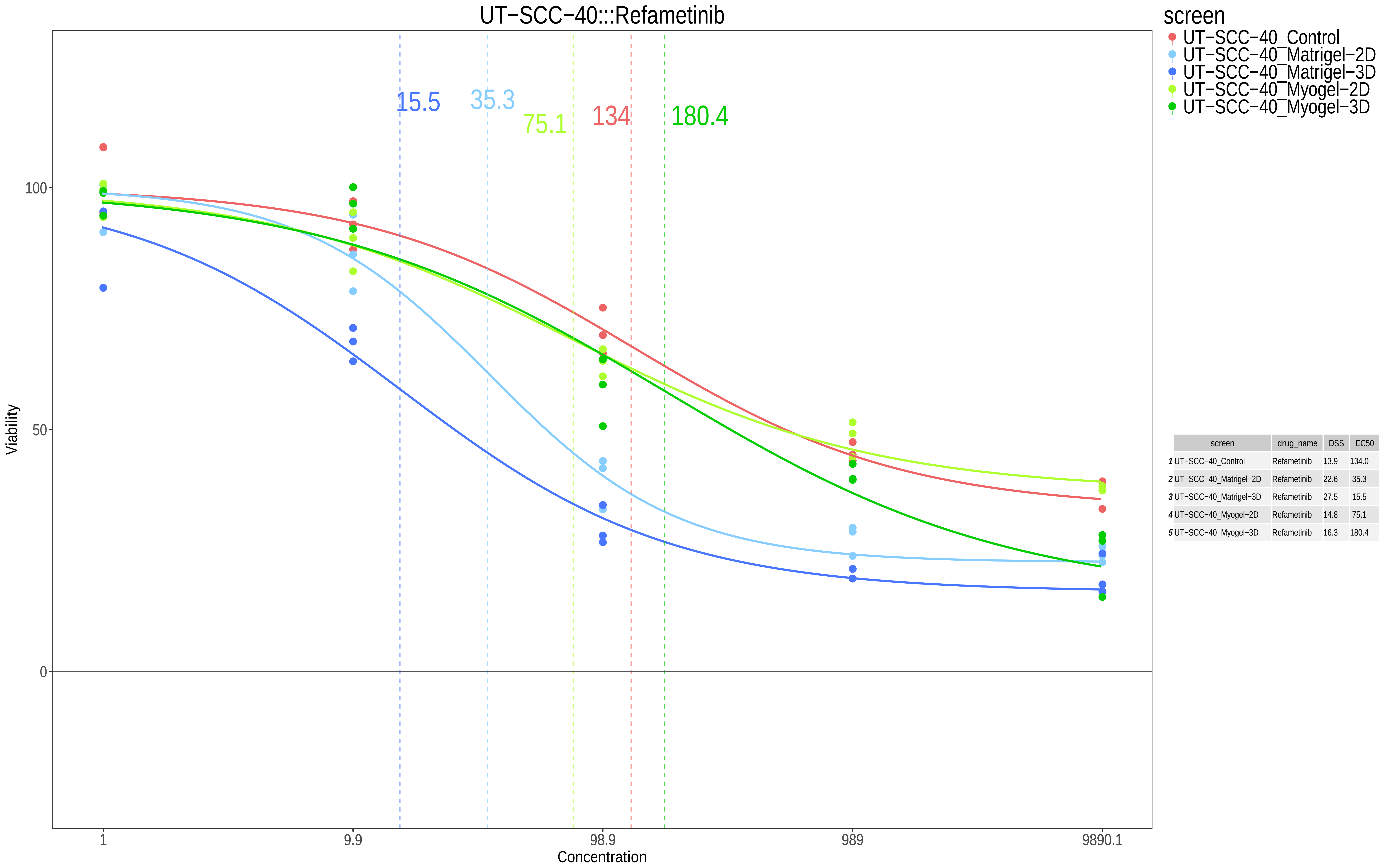

	screen	drug_name	DSS	EC50
1	UT−SCC−40_Control	Refametinib	13.9	134.0
2	UT−SCC−40_Matrigel−2D	Refametinib	22.6	35.3
3	UT−SCC−40_Matrigel−3D	Refametinib	27.5	15.5
4	UT−SCC−40_Myogel−2D	Refametinib	14.8	75.1
5	UT−SCC−40_Myogel−3D	Refametinib	16.3	180.4

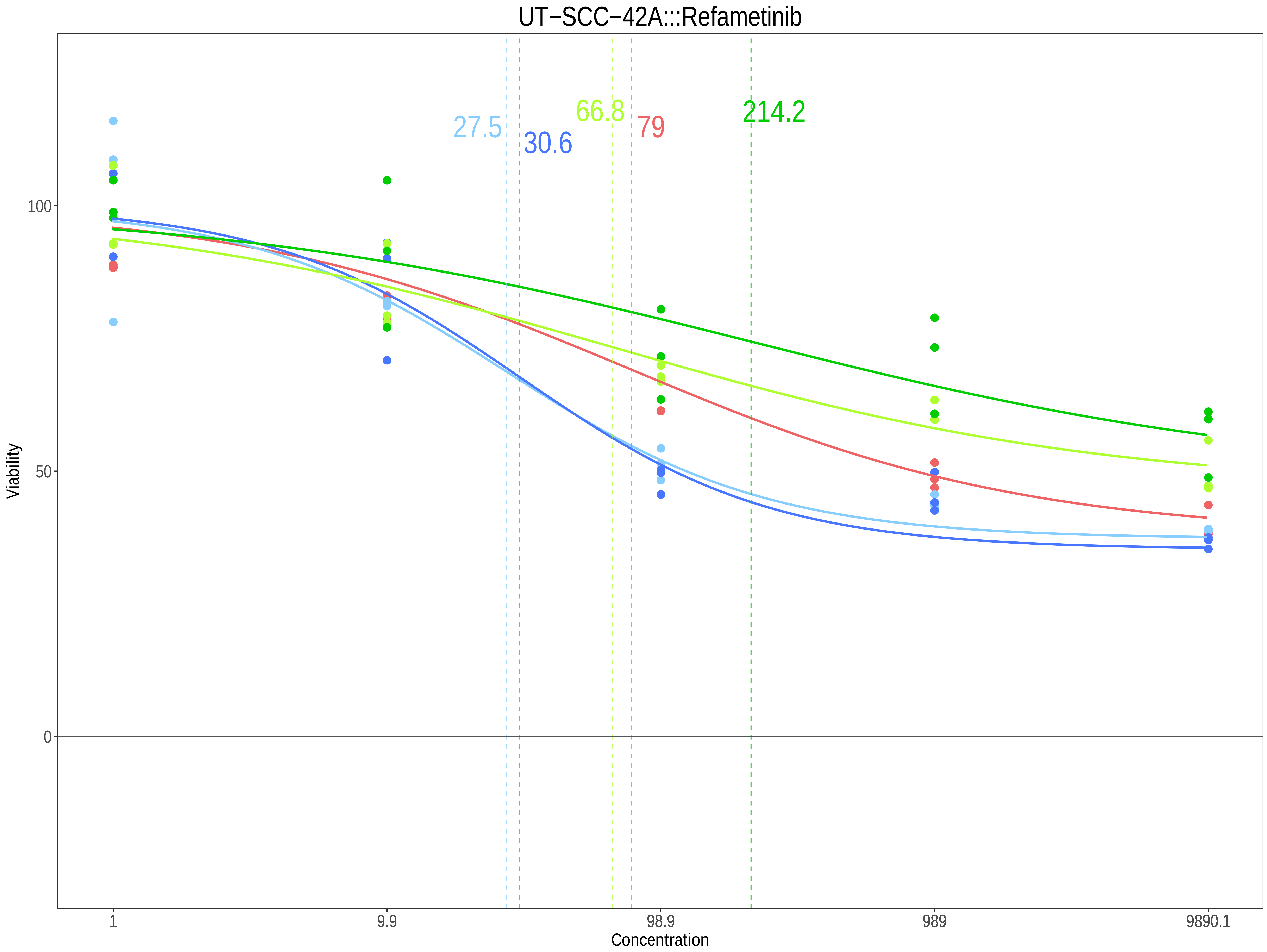

	screen	drug_name	DSS	EC50
1	UT−SCC−42A_Control	Refametinib	14.2	79.0
2	UT−SCC−42A_Matrigel−2D	Refametinib	18.8	27.5
3	UT−SCC−42A_Matrigel−3D	Refametinib	19.1	30.6
4	UT−SCC−42A_Myogel−2D	Refametinib	12.1	66.8
5	UT−SCC−42A_Myogel−3D	Refametinib	8.5	214.2

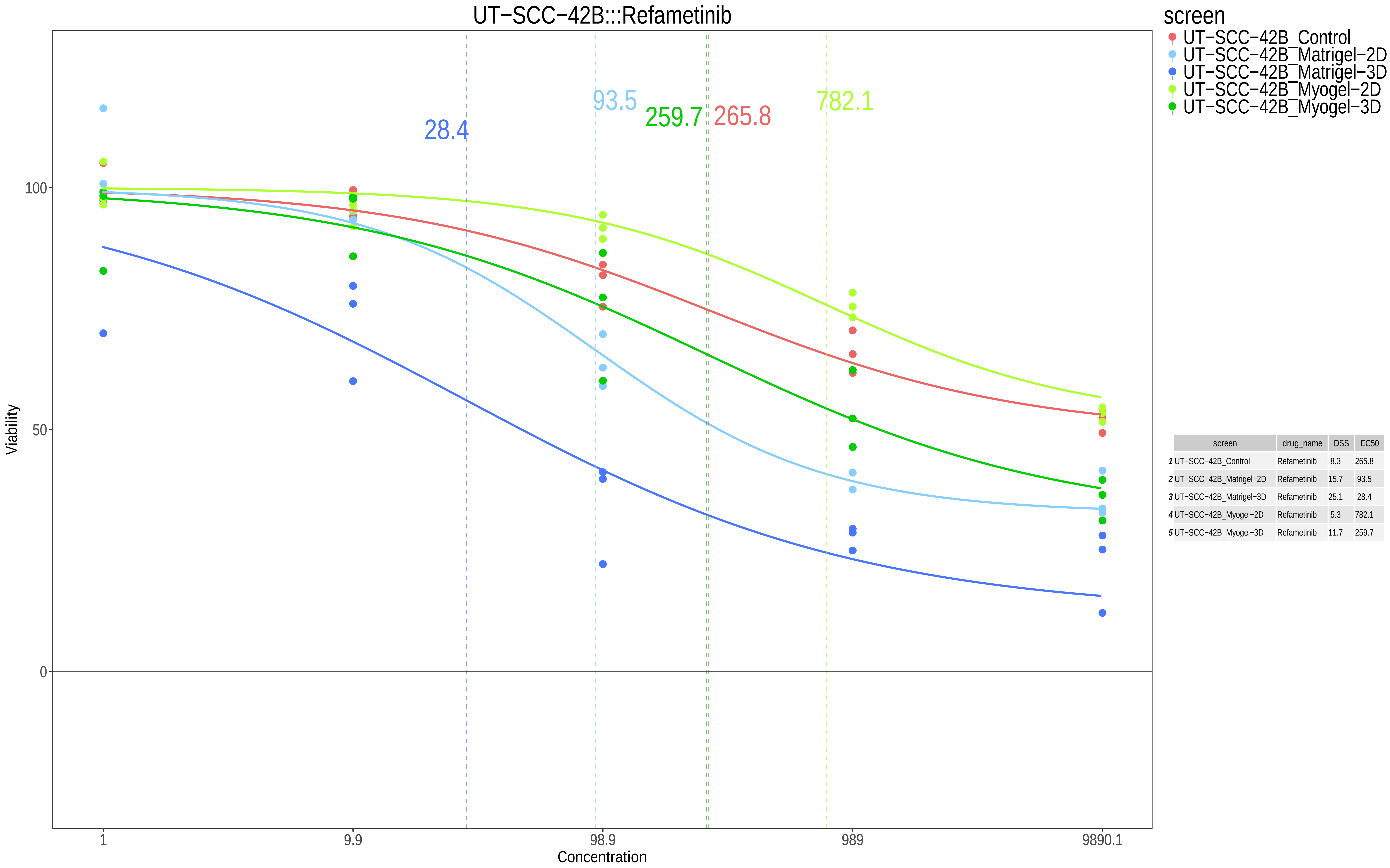

	screen	drug_name	DSS	EC50
1	UT−SCC−42B_Control	Refametinib	8.3	265.8
2	UT−SCC−42B_Matrigel−2D	Refametinib	15.7	93.5
3	UT−SCC−42B_Matrigel−3D	Refametinib	25.1	28.4
4	UT−SCC−42B_Myogel−2D	Refametinib	5.3	782.1
5	UT−SCC−42B_Myogel−3D	Refametinib	11.7	259.7

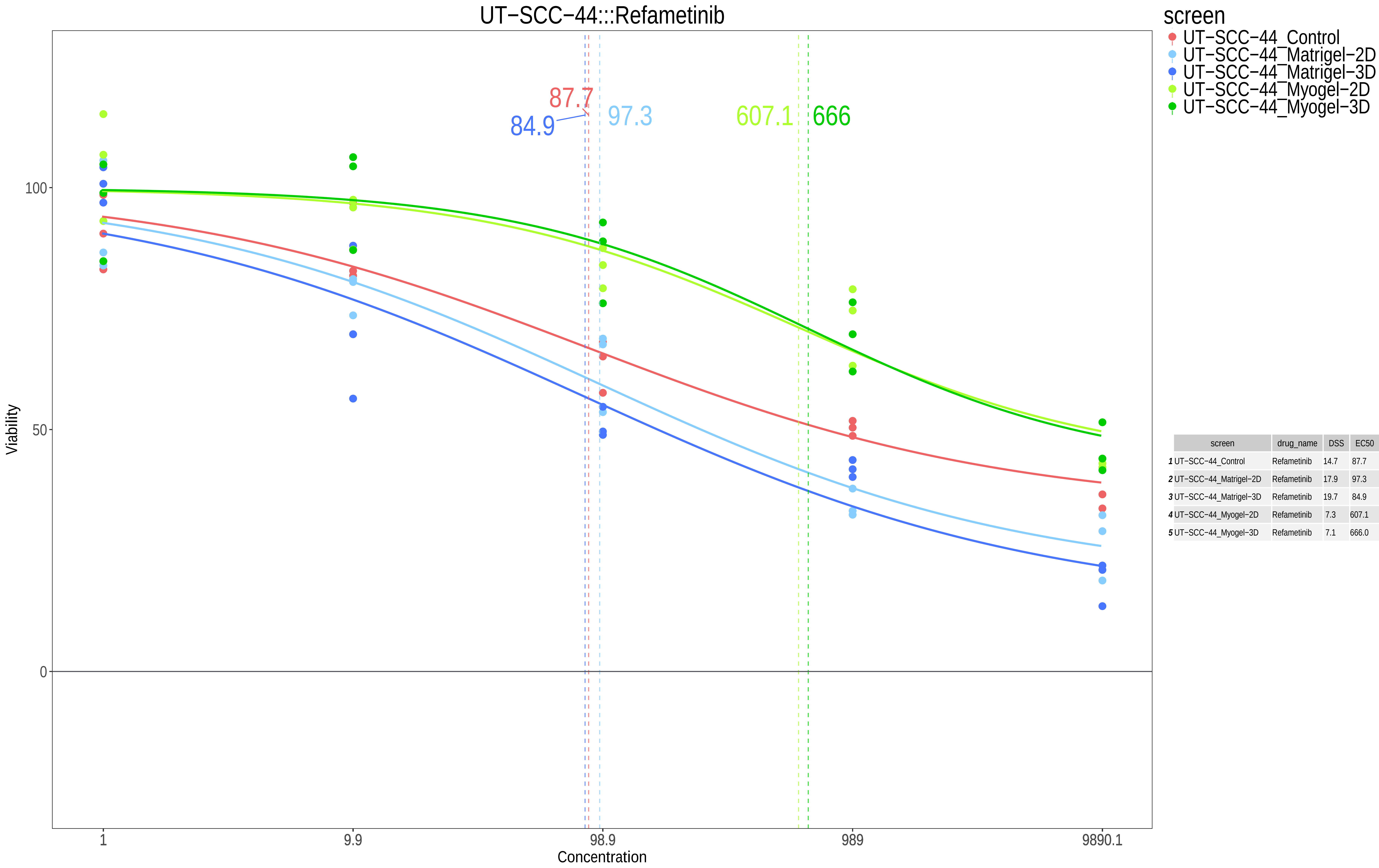

	screen	drug_name	DSS	EC50
1	UT−SCC−44_Control	Refametinib	14.7	87.7
2	UT−SCC−44_Matrigel−2D	Refametinib	17.9	97.3
3	UT−SCC−44_Matrigel−3D	Refametinib	19.7	84.9
4	UT−SCC−44_Myogel−2D	Refametinib	7.3	607.1
5	UT−SCC−44_Myogel−3D	Refametinib	7.1	666.0

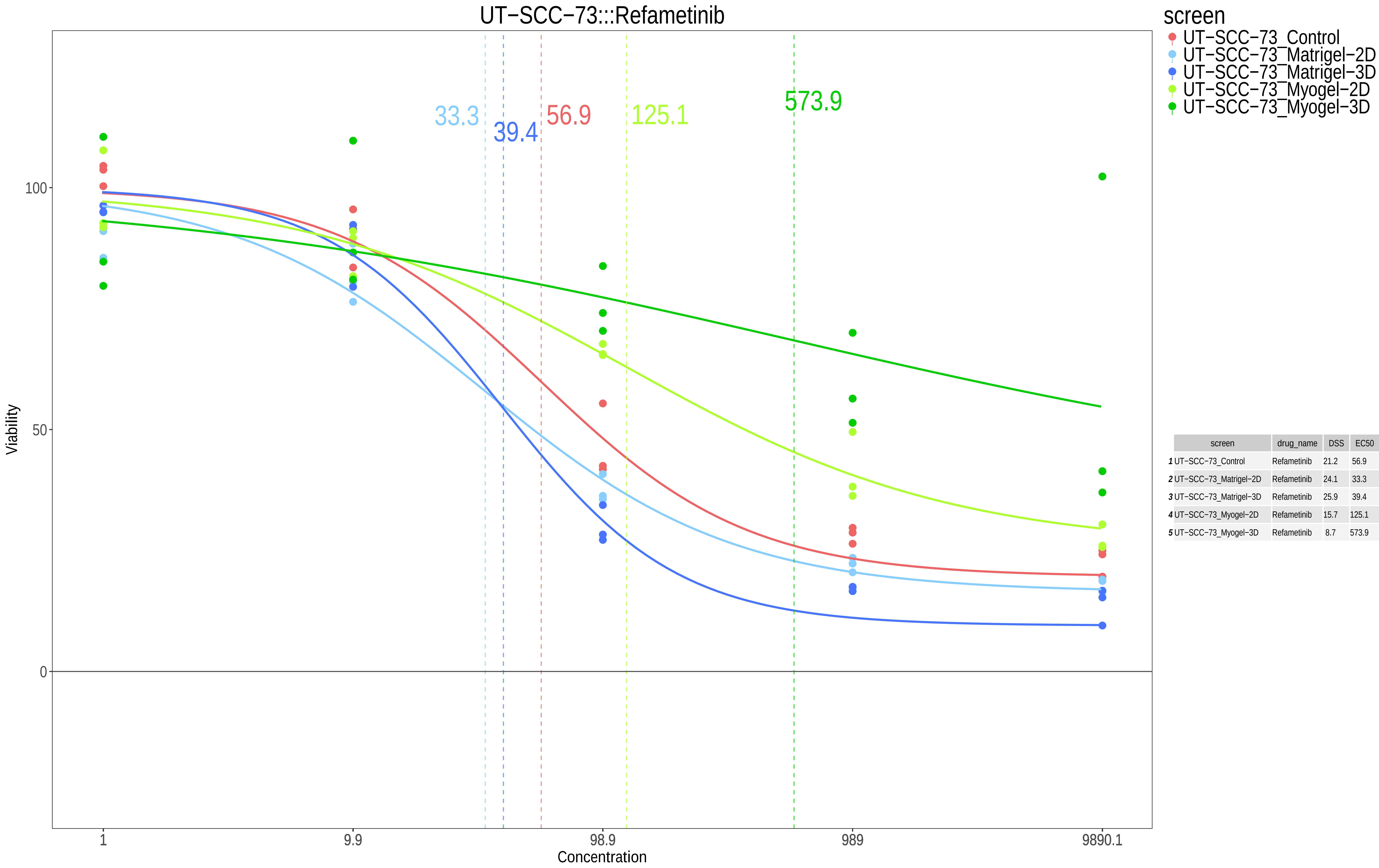

	screen	drug_name	DSS	EC50
1	UT−SCC−73_Control	Refametinib	21.2	56.9
2	UT−SCC−73_Matrigel−2D	Refametinib	24.1	33.3
3	UT−SCC−73_Matrigel−3D	Refametinib	25.9	39.4
4	UT−SCC−73_Myogel−2D	Refametinib	15.7	125.1
5	UT−SCC−73_Myogel−3D	Refametinib	8.7	573.9

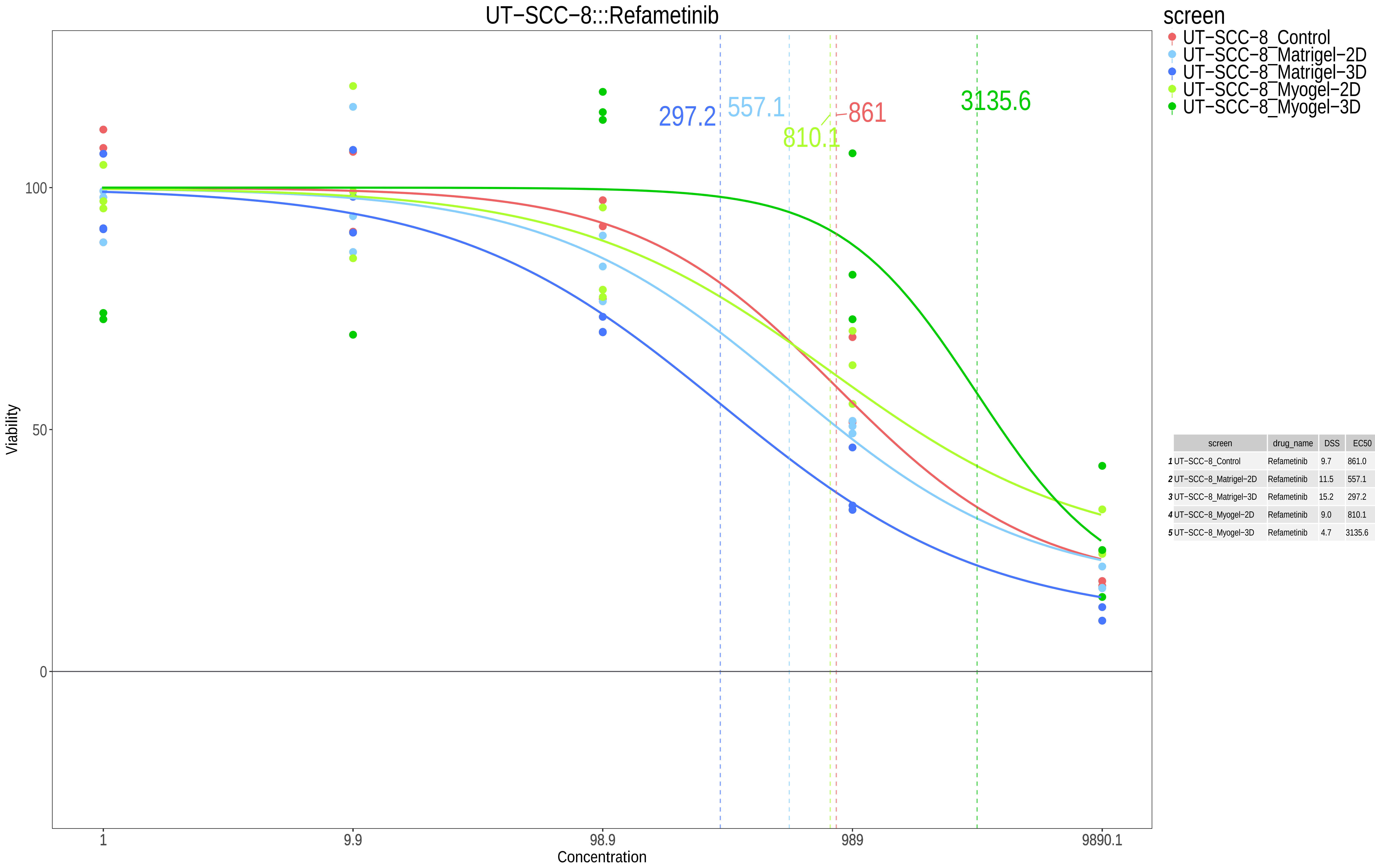

	screen	drug_name	DSS	EC50
1	UT−SCC−8_Control	Refametinib	9.7	861.0
2	UT−SCC−8_Matrigel−2D	Refametinib	11.5	557.1
3	UT−SCC−8_Matrigel−3D	Refametinib	15.2	297.2
4	UT−SCC−8_Myogel−2D	Refametinib	9.0	810.1
5	UT−SCC−8_Myogel−3D	Refametinib	4.7	3135.6

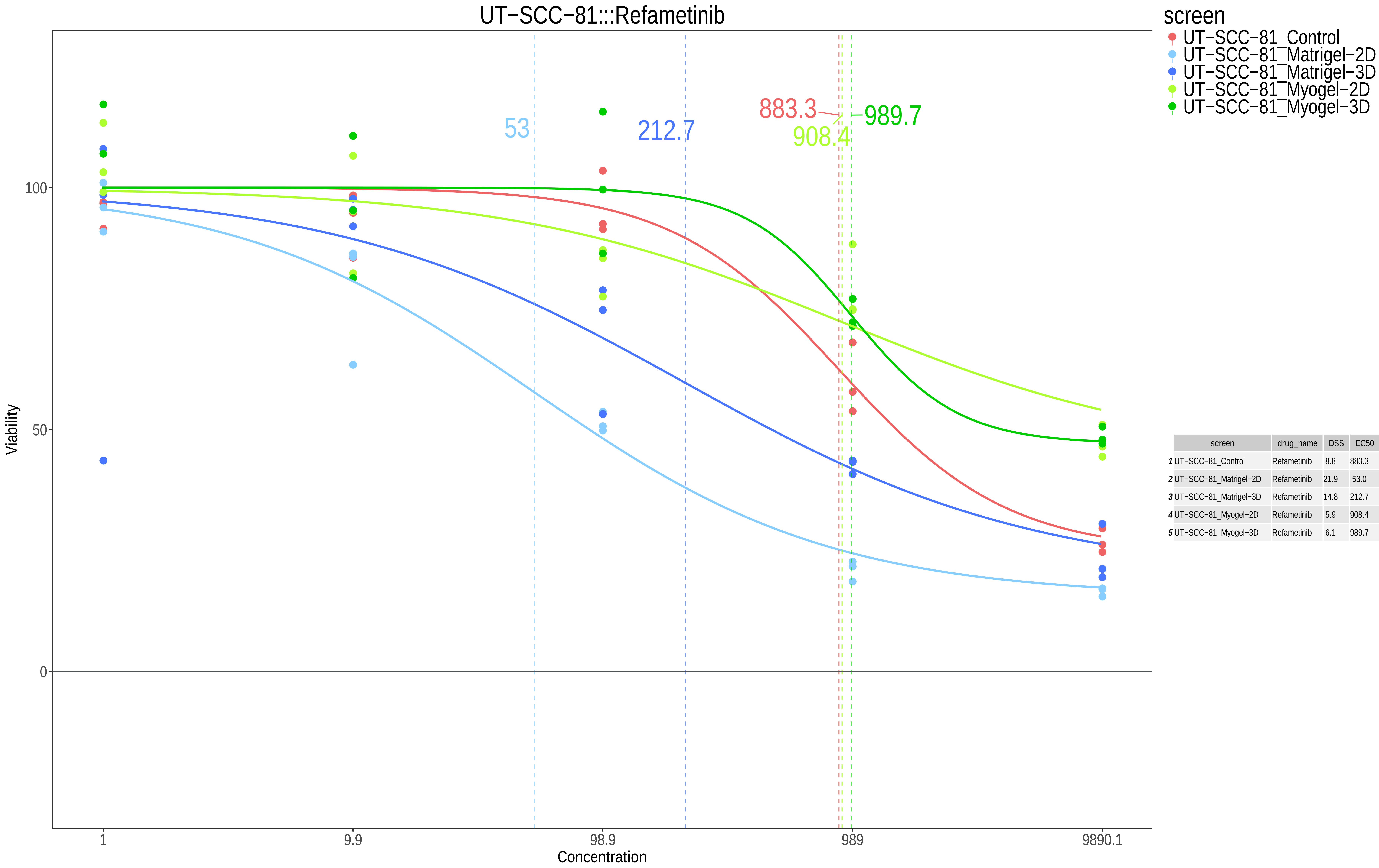

	screen	drug_name	DSS	EC50
1	UT−SCC−81_Control	Refametinib	8.8	883.3
2	UT−SCC−81_Matrigel−2D	Refametinib	21.9	53.0
3	UT−SCC−81_Matrigel−3D	Refametinib	14.8	212.7
4	UT−SCC−81_Myogel−2D	Refametinib	5.9	908.4
5	UT−SCC−81_Myogel−3D	Refametinib	6.1	989.7

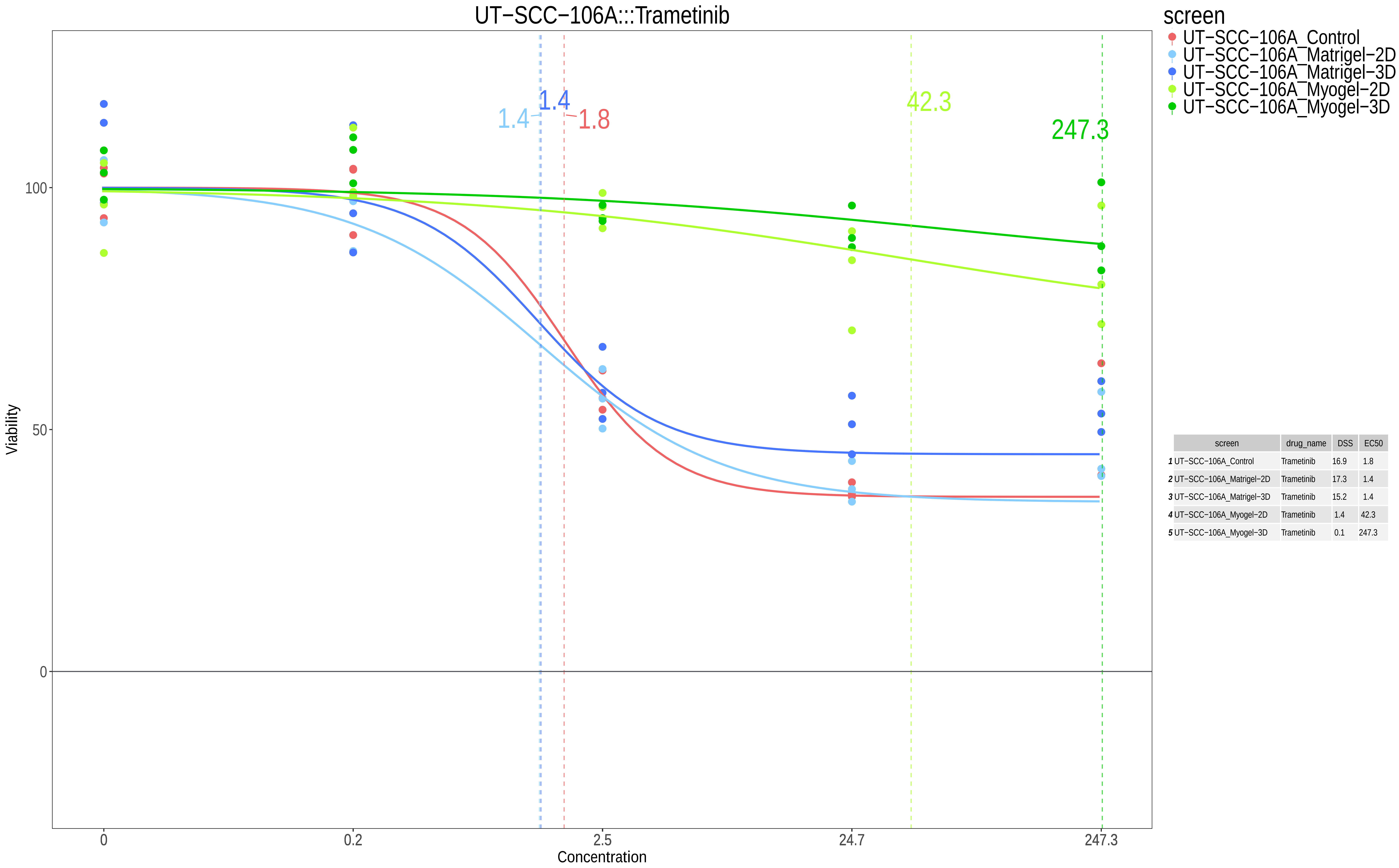

	screen	drug_name	DSS	EC50
1	UT−SCC−106A_Control	Trametinib	16.9	1.8
2	UT−SCC−106A_Matrigel−2D	Trametinib	17.3	1.4
3	UT−SCC−106A_Matrigel−3D	Trametinib	15.2	1.4
4	UT−SCC−106A_Myogel−2D	Trametinib	1.4	42.3
5	UT−SCC−106A_Myogel−3D	Trametinib	0.1	247.3

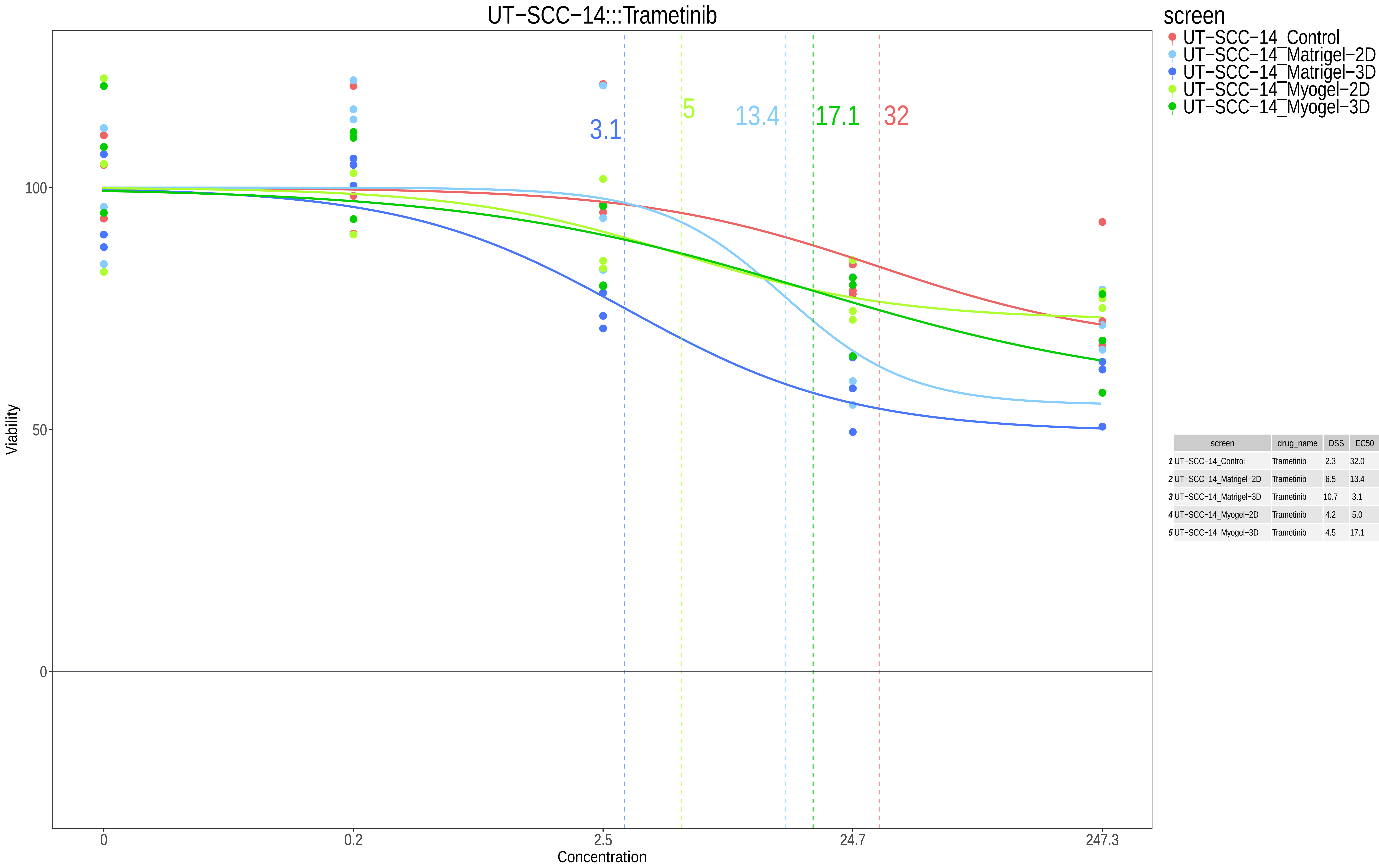

	screen	drug_name	DSS	EC50
1	UT−SCC−14_Control	Trametinib	2.3	32.0
2	UT−SCC−14_Matrigel−2D	Trametinib	6.5	13.4
3	UT−SCC−14_Matrigel−3D	Trametinib	10.7	3.1
4	UT−SCC−14_Myogel−2D	Trametinib	4.2	5.0
5	UT−SCC−14_Myogel−3D	Trametinib	4.5	17.1

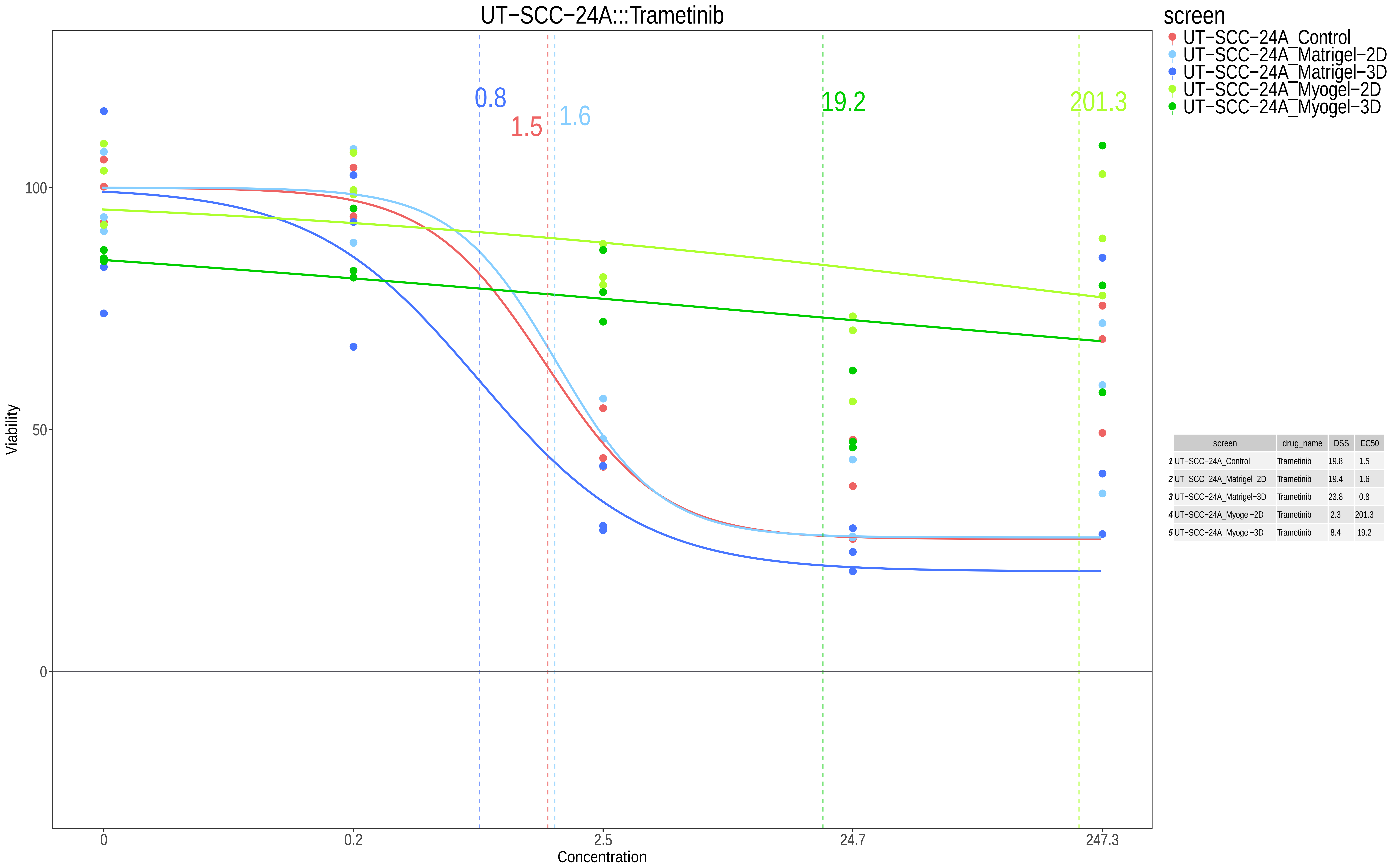

	screen	drug_name	DSS	EC50
1	UT−SCC−24A_Control	Trametinib	19.8	1.5
2	UT−SCC−24A_Matrigel−2D	Trametinib	19.4	1.6
3	UT−SCC−24A_Matrigel−3D	Trametinib	23.8	0.8
4	UT−SCC−24A_Myogel−2D	Trametinib	2.3	201.3
5	UT−SCC−24A_Myogel−3D	Trametinib	8.4	19.2

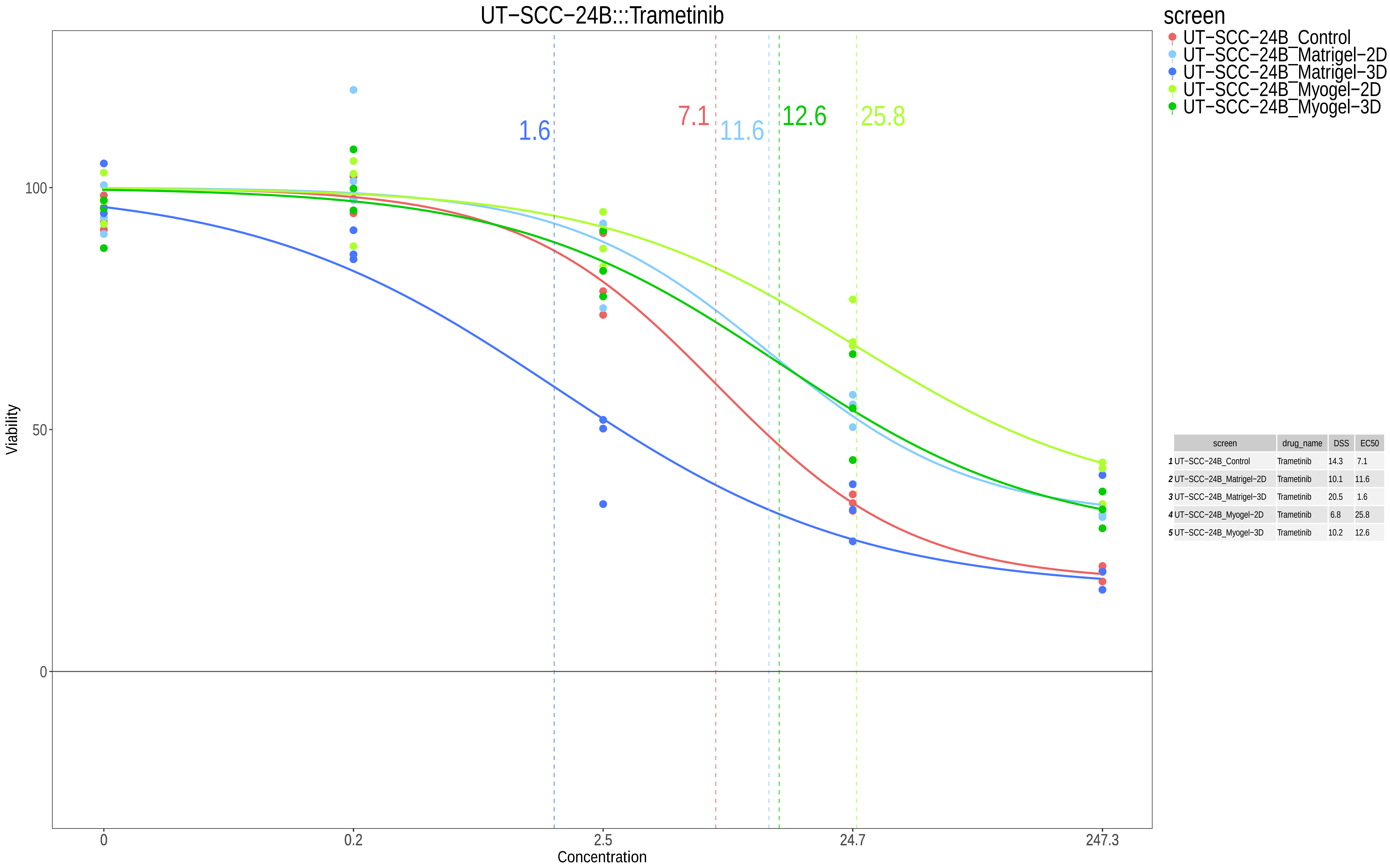

	screen	drug_name	DSS	EC50
1	UT−SCC−24B_Control	Trametinib	14.3	7.1
2	UT−SCC−24B_Matrigel−2D	Trametinib	10.1	11.6
3	UT−SCC−24B_Matrigel−3D	Trametinib	20.5	1.6
4	UT−SCC−24B_Myogel−2D	Trametinib	6.8	25.8
5	UT−SCC−24B_Myogel−3D	Trametinib	10.2	12.6

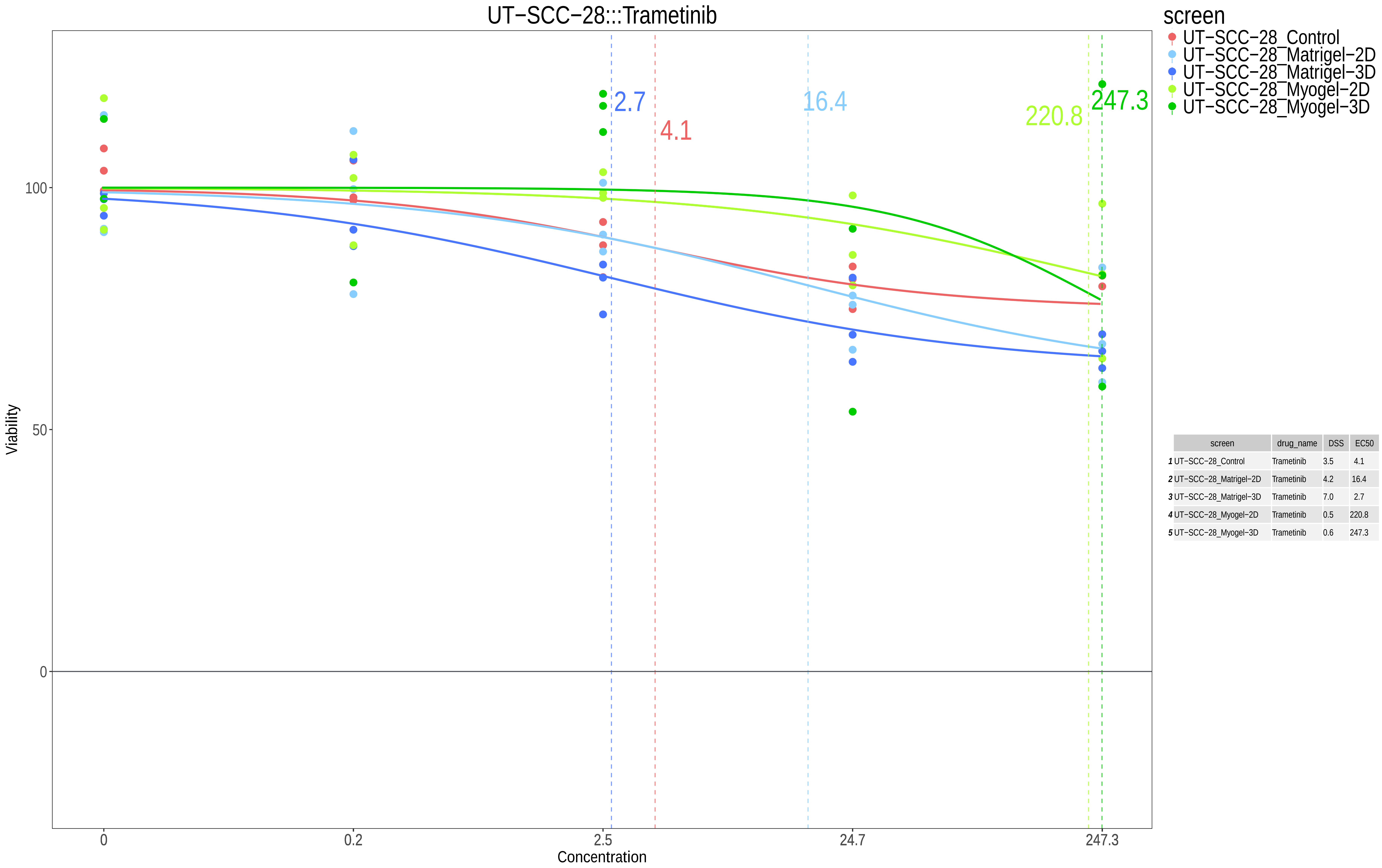

	screen	drug_name	DSS	EC50
1	UT−SCC−28_Control	Trametinib	3.5	4.1
2	UT−SCC−28_Matrigel−2D	Trametinib	4.2	16.4
3	UT−SCC−28_Matrigel−3D	Trametinib	7.0	2.7
4	UT−SCC−28_Myogel−2D	Trametinib	0.5	220.8
5	UT−SCC−28_Myogel−3D	Trametinib	0.6	247.3

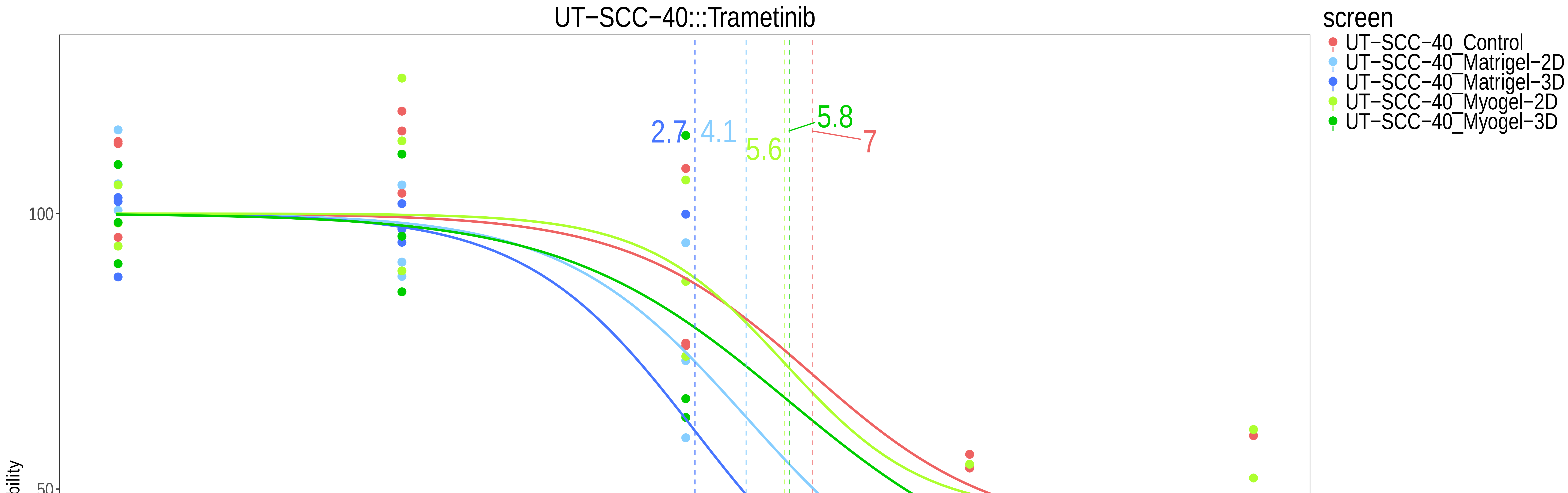

	screen	drug_name	DSS	EC50
1	UT−SCC−40_Control	Trametinib	10.4	7.0
2	UT−SCC−40_Matrigel−2D	Trametinib	15.6	4.1
3	UT−SCC−40_Matrigel−3D	Trametinib	18.5	2.7
4	UT−SCC−40_Myogel−2D	Trametinib	10.7	5.6
5	UT−SCC−40_Myogel−3D	Trametinib	12.7	5.8

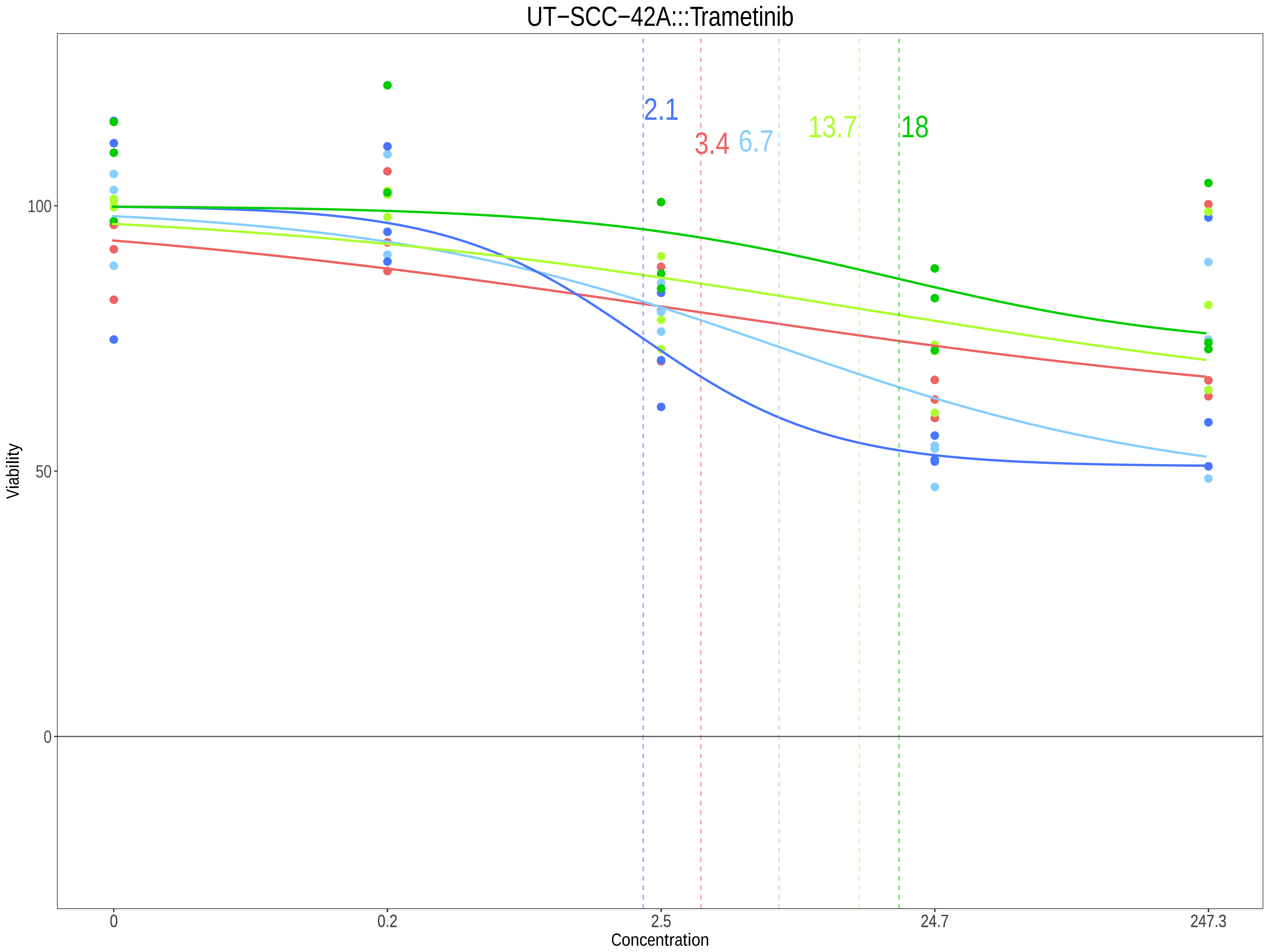

	screen	drug_name	DSS	EC50
1	UT−SCC−42A_Control	Trametinib	6.5	3.4
2	UT−SCC−42A_Matrigel−2D	Trametinib	8.5	6.7
3	UT−SCC−42A_Matrigel−3D	Trametinib	11.9	2.1
4	UT−SCC−42A_Myogel−2D	Trametinib	4.2	13.7
5	UT−SCC−42A_Myogel−3D	Trametinib	2.2	18.0

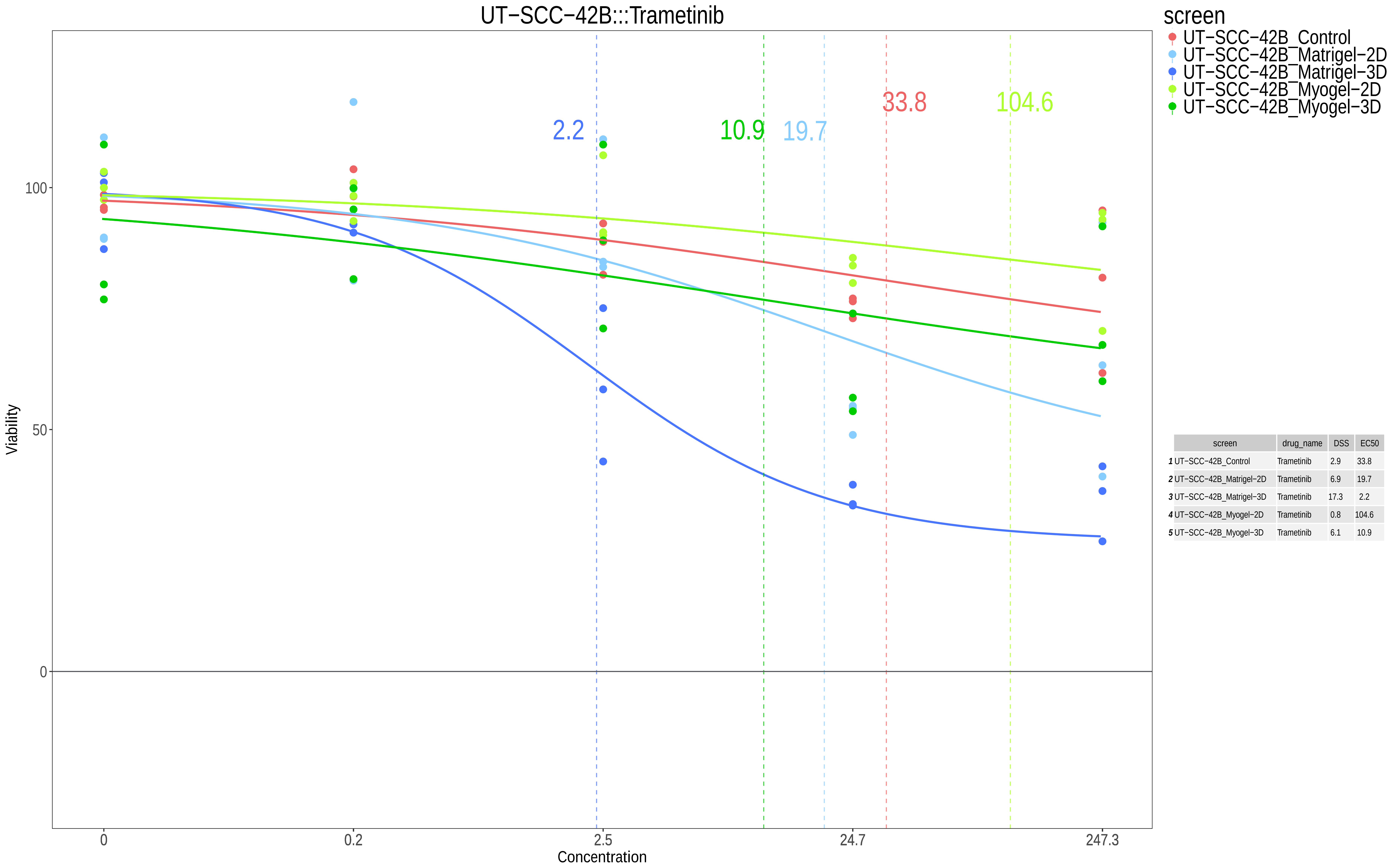

	screen	drug_name	DSS	EC50
1	UT−SCC−42B_Control	Trametinib	2.9	33.8
2	UT−SCC−42B_Matrigel−2D	Trametinib	6.9	19.7
3	UT−SCC−42B_Matrigel−3D	Trametinib	17.3	2.2
4	UT−SCC−42B_Myogel−2D	Trametinib	0.8	104.6
5	UT−SCC−42B_Myogel−3D	Trametinib	6.1	10.9

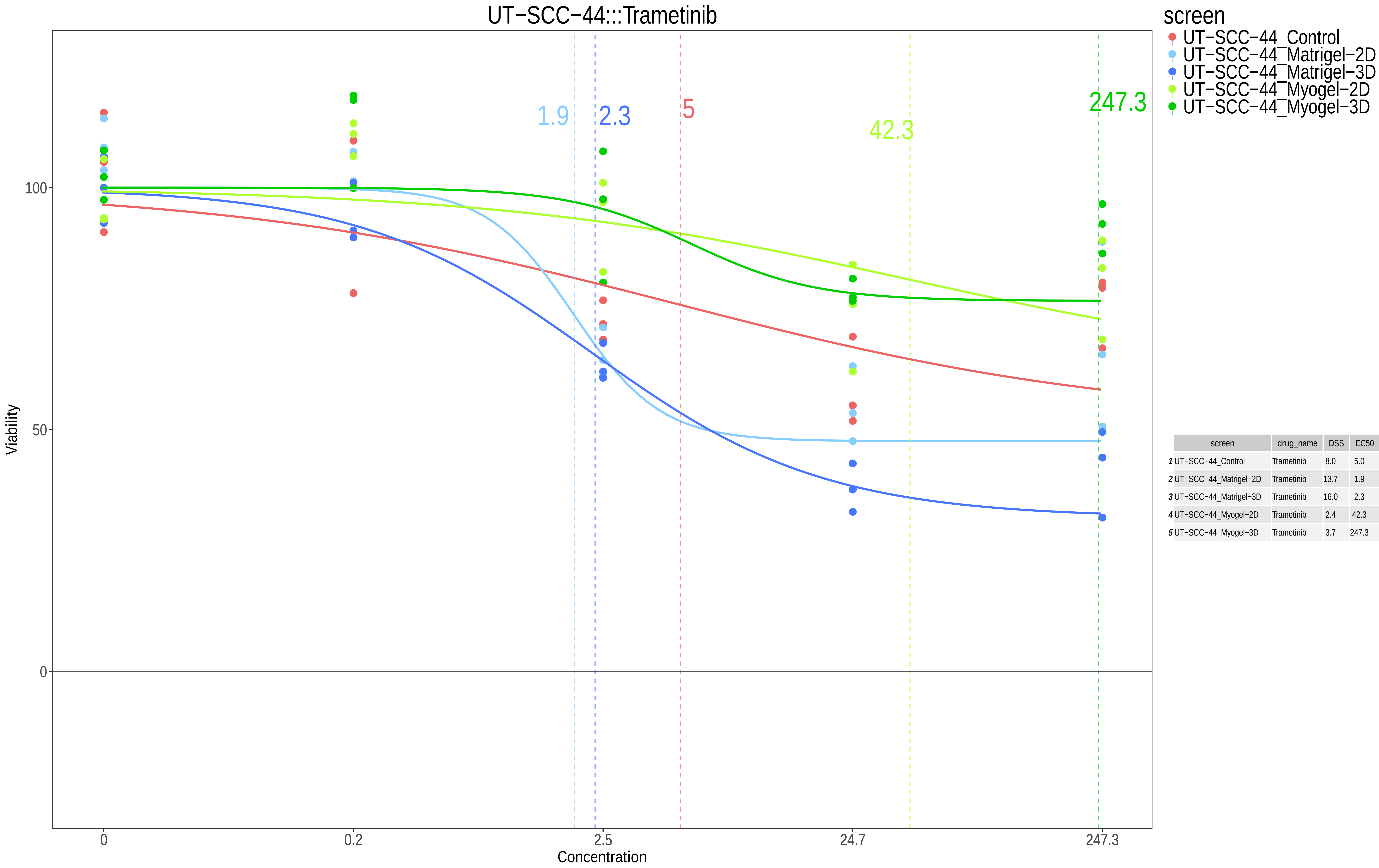

	screen	drug_name	DSS	EC50
1	UT−SCC−44_Control	Trametinib	8.0	5.0
2	UT−SCC−44_Matrigel−2D	Trametinib	13.7	1.9
3	UT−SCC−44_Matrigel−3D	Trametinib	16.0	2.3
4	UT−SCC−44_Myogel−2D	Trametinib	2.4	42.3
5	UT−SCC−44_Myogel−3D	Trametinib	3.7	247.3

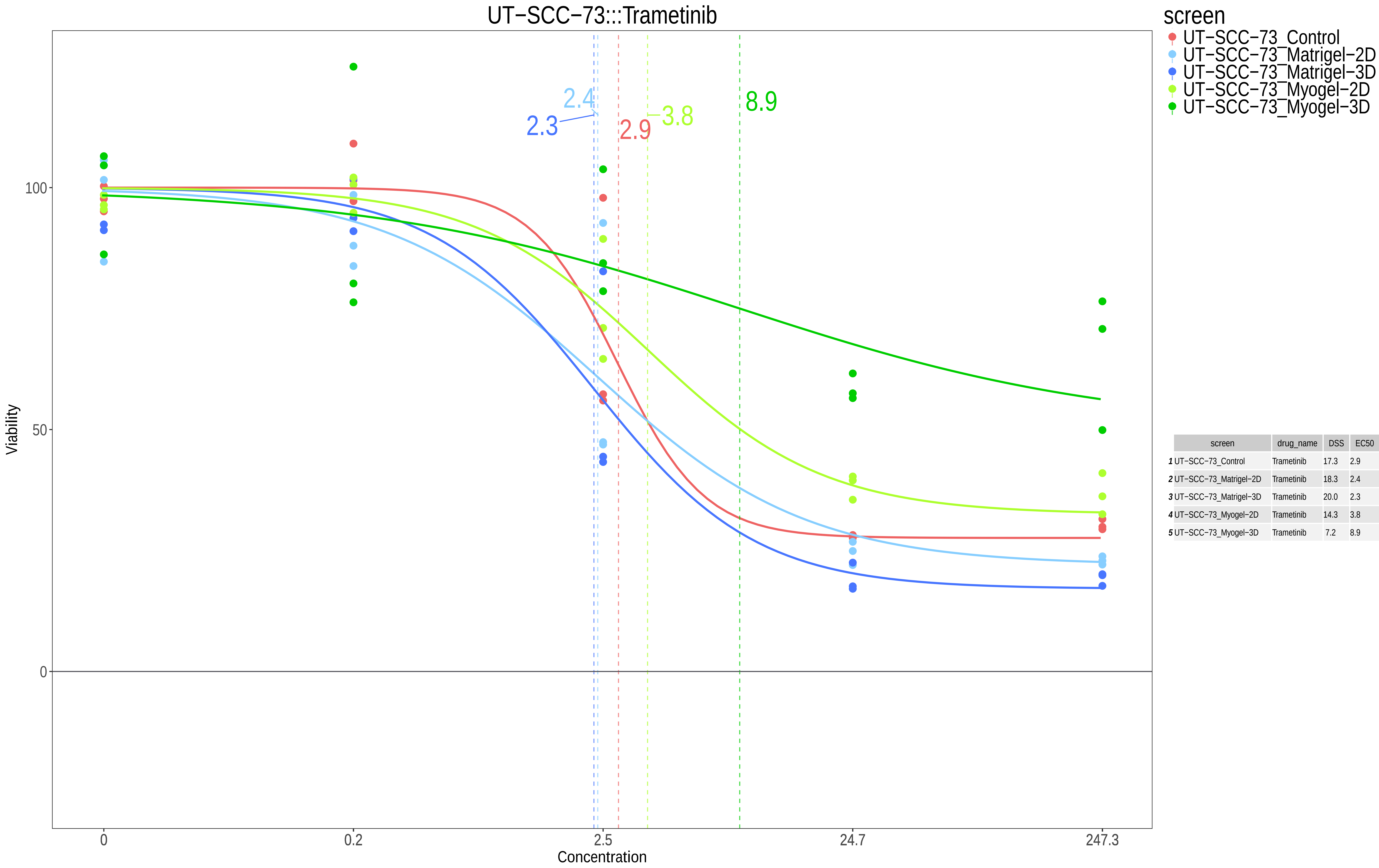

	screen	drug_name	DSS	EC50
1	UT−SCC−73_Control	Trametinib	17.3	2.9
2	UT−SCC−73_Matrigel−2D	Trametinib	18.3	2.4
3	UT−SCC−73_Matrigel−3D	Trametinib	20.0	2.3
4	UT−SCC−73_Myogel−2D	Trametinib	14.3	3.8
5	UT−SCC−73_Myogel−3D	Trametinib	7.2	8.9

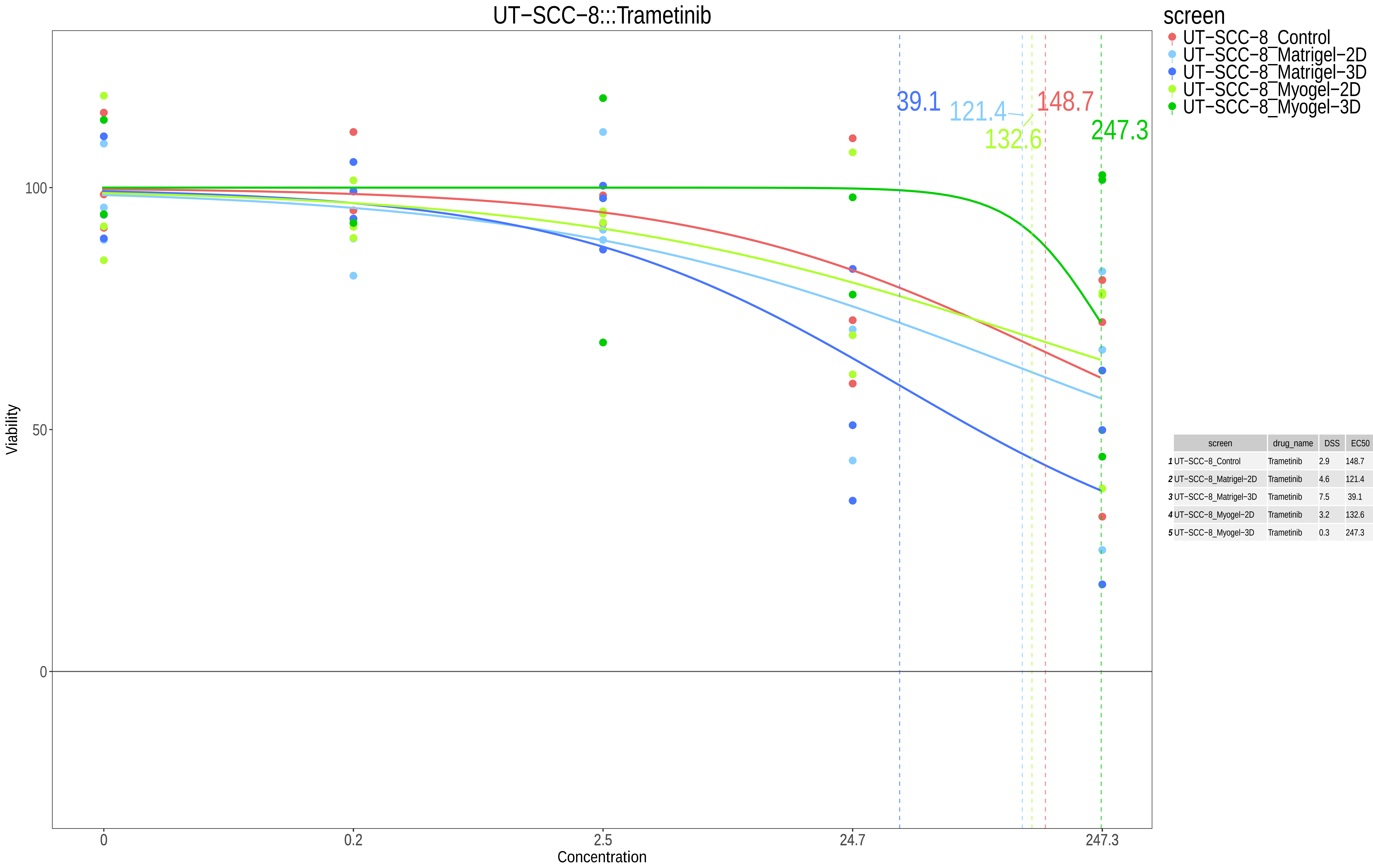

	screen	drug_name	DSS	EC50
1	UT−SCC−8_Control	Trametinib	2.9	148.7
2	UT−SCC−8_Matrigel−2D	Trametinib	4.6	121.4
3	UT−SCC−8_Matrigel−3D	Trametinib	7.5	39.1
4	UT−SCC−8_Myogel−2D	Trametinib	3.2	132.6
5	UT−SCC−8_Myogel−3D	Trametinib	0.3	247.3

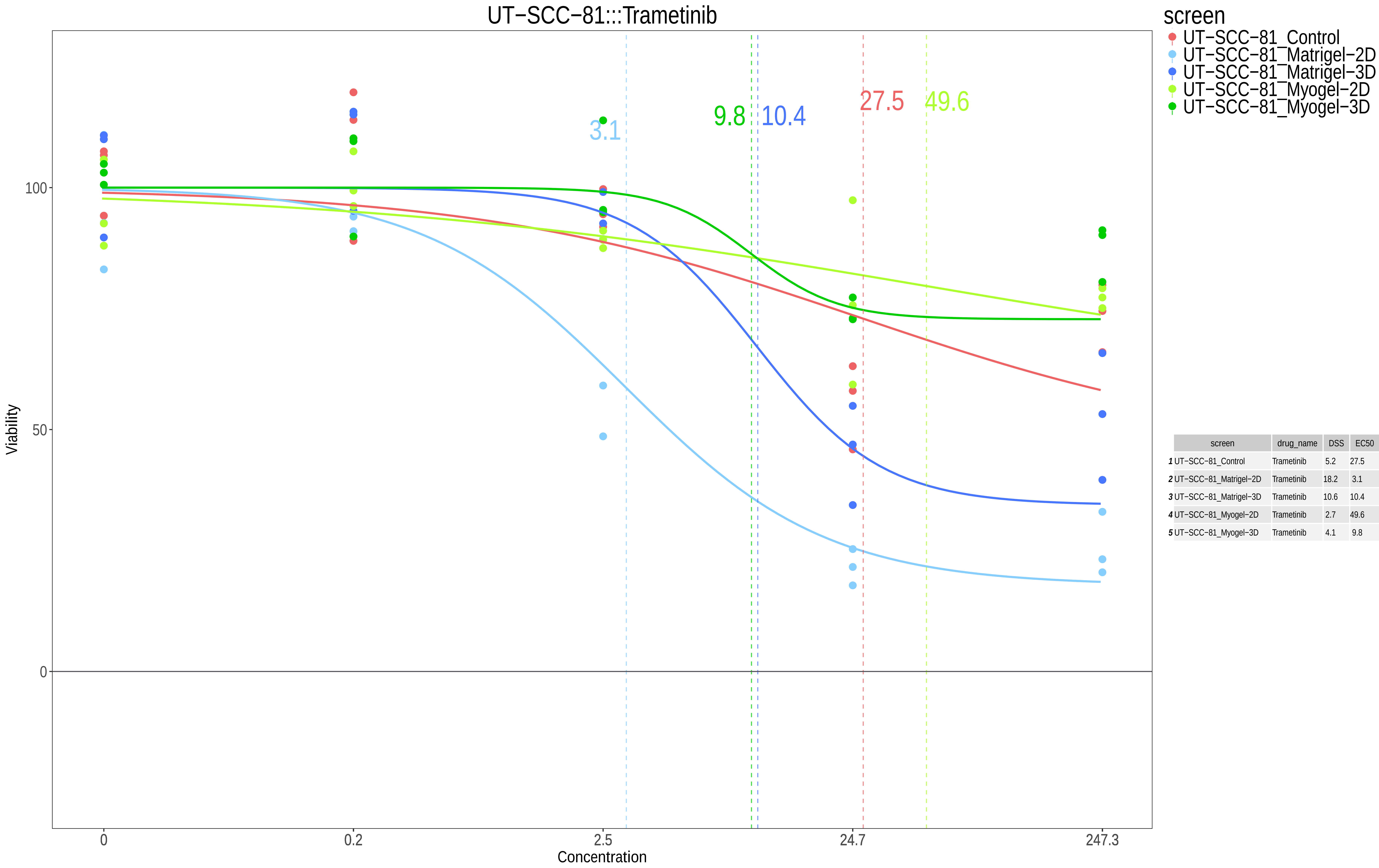

	screen	drug_name	DSS	EC50
1	UT−SCC−81_Control	Trametinib	5.2	27.5
2	UT−SCC−81_Matrigel−2D	Trametinib	18.2	3.1
3	UT−SCC−81_Matrigel−3D	Trametinib	10.6	10.4
4	UT−SCC−81_Myogel−2D	Trametinib	2.7	49.6
5	UT−SCC−81_Myogel−3D	Trametinib	4.1	9.8

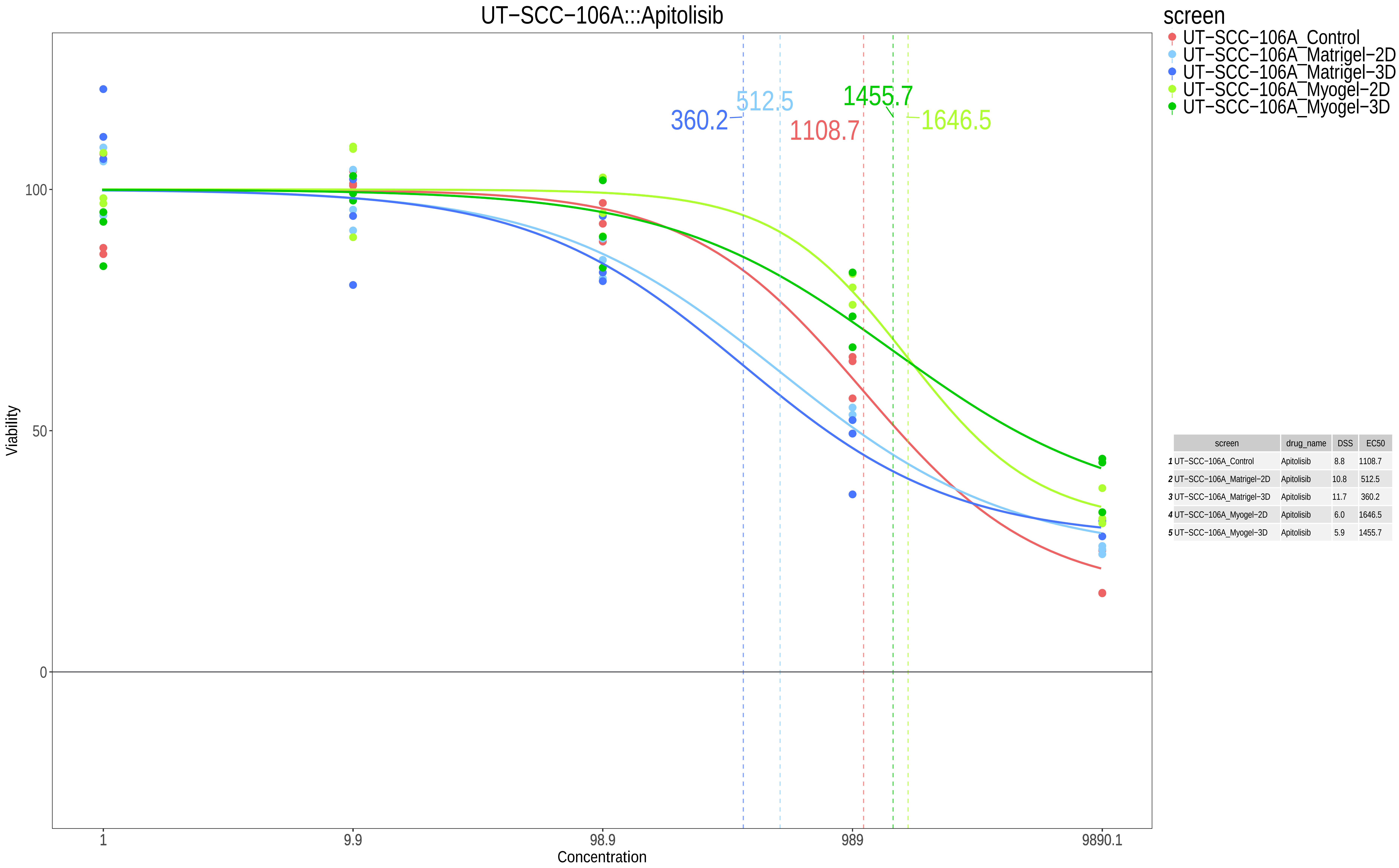

	screen	drug_name	DSS	EC50
1	UT−SCC−106A_Control	Apitolisib	8.8	1108.7
2	UT−SCC−106A_Matrigel−2D	Apitolisib	10.8	512.5
3	UT−SCC−106A_Matrigel−3D	Apitolisib	11.7	360.2
4	UT−SCC−106A_Myogel−2D	Apitolisib	6.0	1646.5
5	UT−SCC−106A_Myogel−3D	Apitolisib	5.9	1455.7

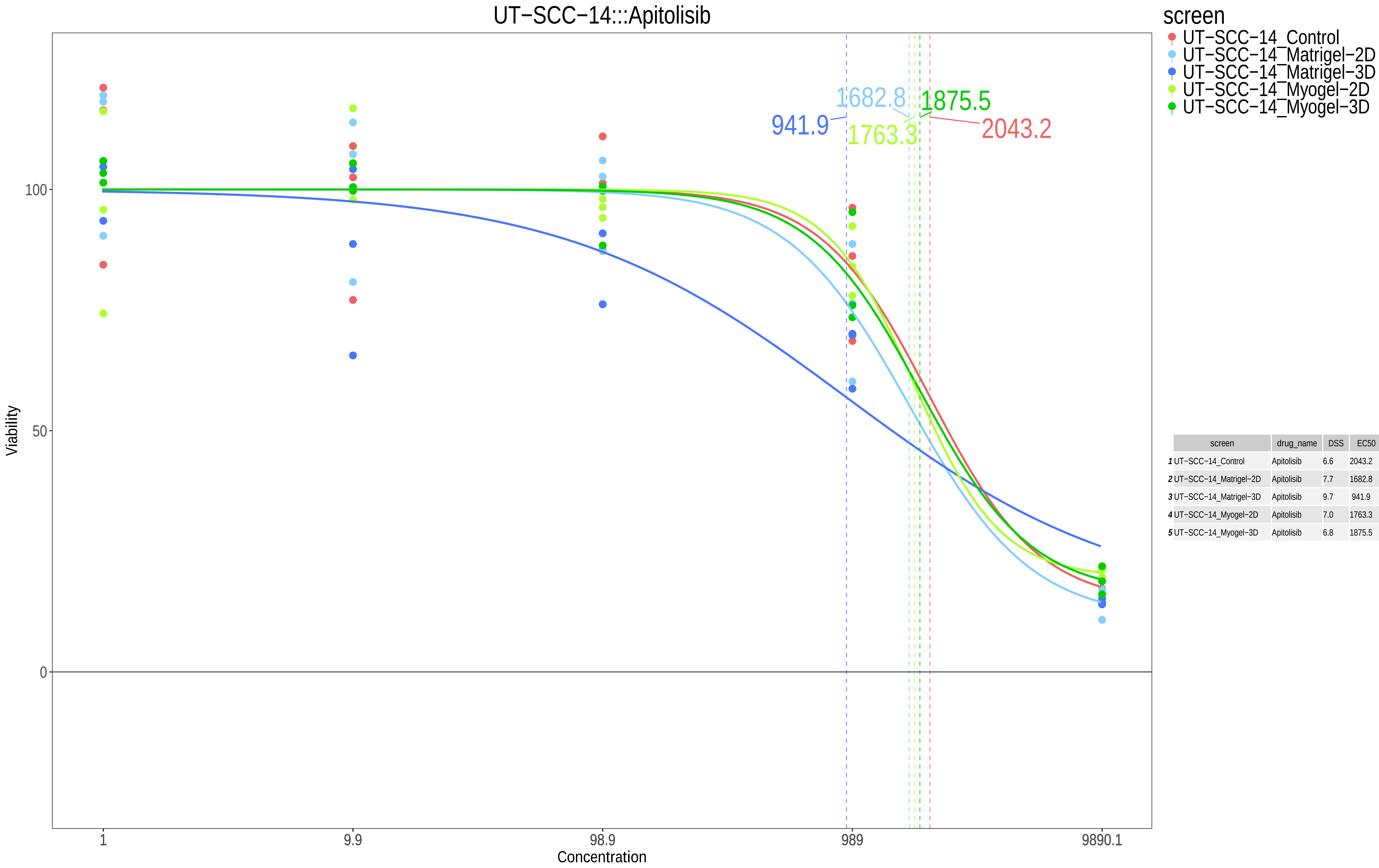

	screen	drug_name	DSS	EC50
1	UT−SCC−14_Control	Apitolisib	6.6	2043.2
2	UT−SCC−14_Matrigel−2D	Apitolisib	7.7	1682.8
3	UT−SCC−14_Matrigel−3D	Apitolisib	9.7	941.9
4	UT−SCC−14_Myogel−2D	Apitolisib	7.0	1763.3
5	UT−SCC−14_Myogel−3D	Apitolisib	6.8	1875.5

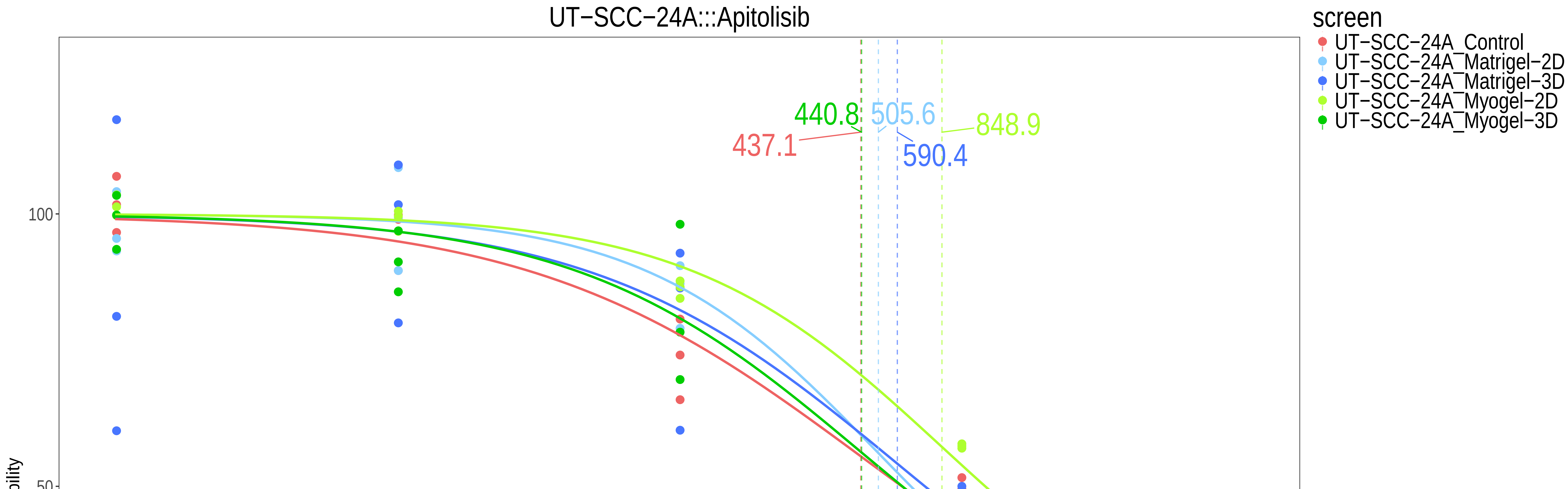

	screen	drug_name	DSS	EC50
1	UT−SCC−24A_Control	Apitolisib	13.3	437.1
2	UT−SCC−24A_Matrigel−2D	Apitolisib	12.7	505.6
3	UT−SCC−24A_Matrigel−3D	Apitolisib	12.4	590.4
4	UT−SCC−24A_Myogel−2D	Apitolisib	10.1	848.9
5	UT−SCC−24A_Myogel−3D	Apitolisib	13.2	440.8

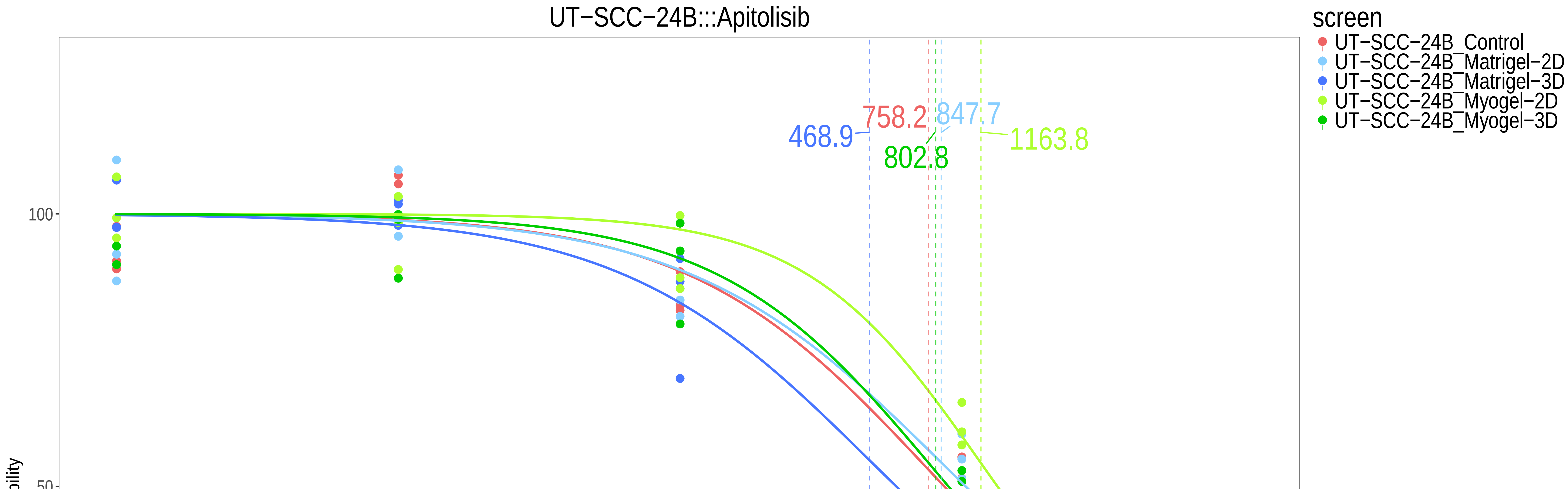

	screen	drug_name	DSS	EC50
1	UT−SCC−24B_Control	Apitolisib	11.6	758.2
2	UT−SCC−24B_Matrigel−2D	Apitolisib	10.8	847.7
3	UT−SCC−24B_Matrigel−3D	Apitolisib	13.4	468.9
4	UT−SCC−24B_Myogel−2D	Apitolisib	9.5	1163.8
5	UT−SCC−24B_Myogel−3D	Apitolisib	11.5	802.8

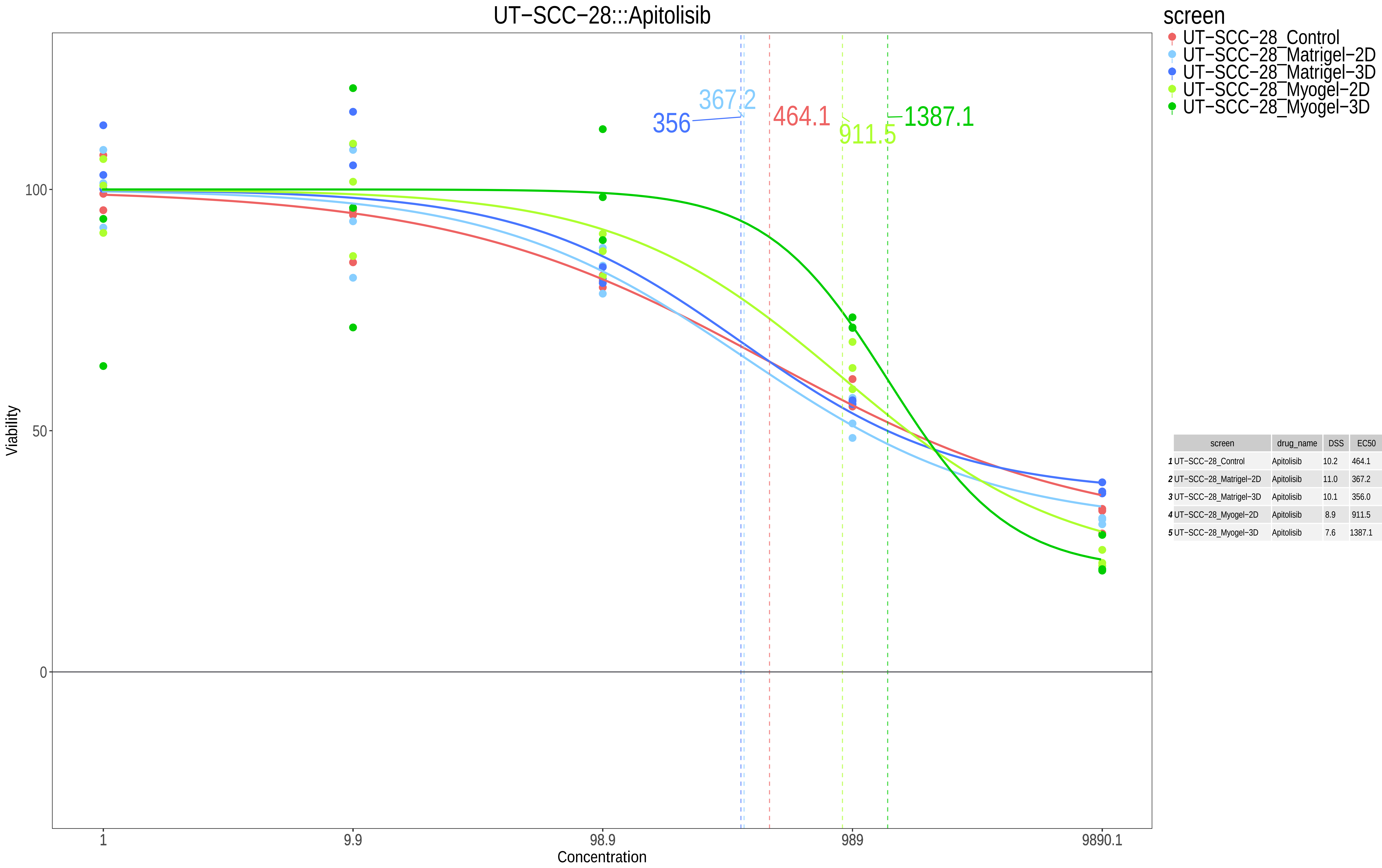

	screen	drug_name	DSS	EC50
1	UT−SCC−28_Control	Apitolisib	10.2	464.1
2	UT−SCC−28_Matrigel−2D	Apitolisib	11.0	367.2
3	UT−SCC−28_Matrigel−3D	Apitolisib	10.1	356.0
4	UT−SCC−28_Myogel−2D	Apitolisib	8.9	911.5
5	UT−SCC−28_Myogel−3D	Apitolisib	7.6	1387.1

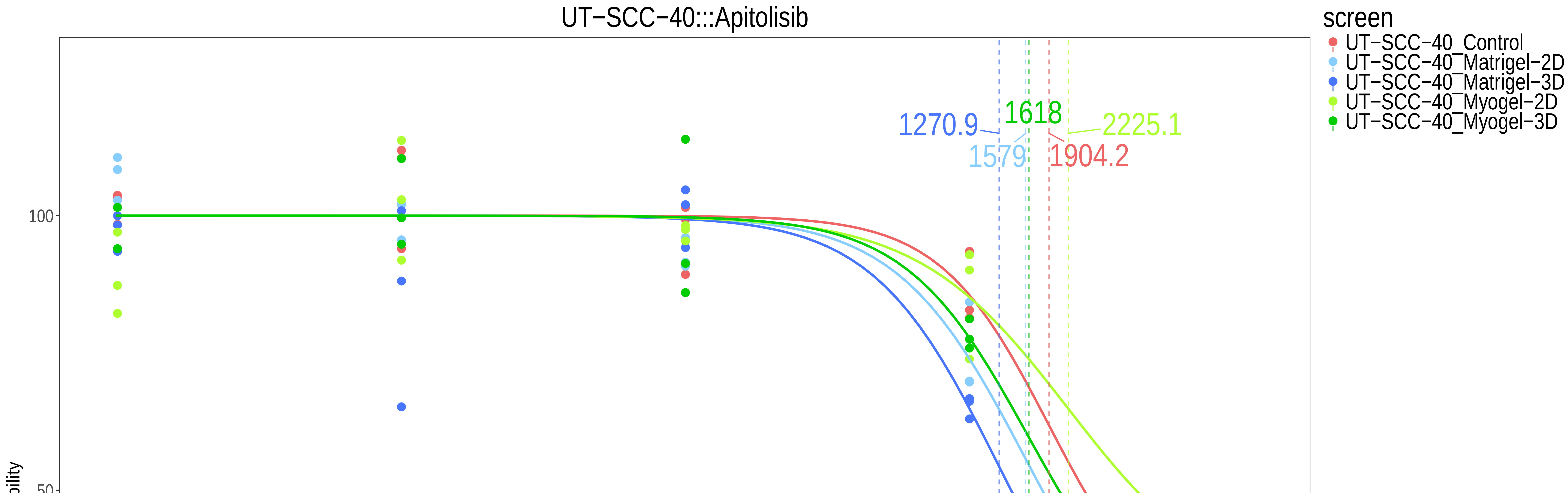

	screen	drug_name	DSS	EC50
1	UT−SCC−40_Control	Apitolisib	6.2	1904.2
2	UT−SCC−40_Matrigel−2D	Apitolisib	8.0	1579.0
3	UT−SCC−40_Matrigel−3D	Apitolisib	9.3	1270.9
4	UT−SCC−40_Myogel−2D	Apitolisib	5.0	2225.1
5	UT−SCC−40_Myogel−3D	Apitolisib	7.2	1618.0

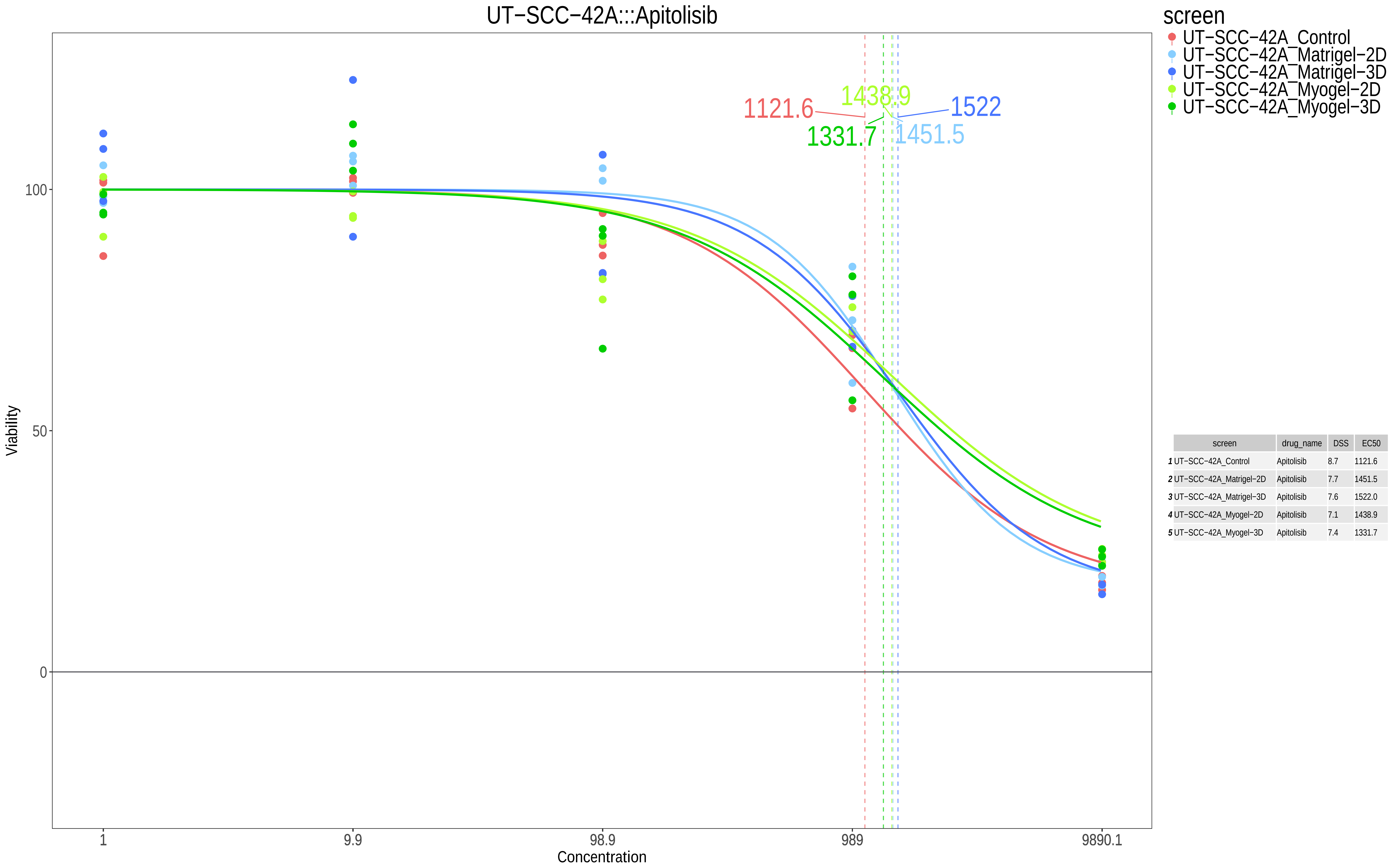

	screen	drug_name	DSS	EC50
1	UT−SCC−42A_Control	Apitolisib	8.7	1121.6
2	UT−SCC−42A_Matrigel−2D	Apitolisib	7.7	1451.5
3	UT−SCC−42A_Matrigel−3D	Apitolisib	7.6	1522.0
4	UT−SCC−42A_Myogel−2D	Apitolisib	7.1	1438.9
5	UT−SCC−42A_Myogel−3D	Apitolisib	7.4	1331.7

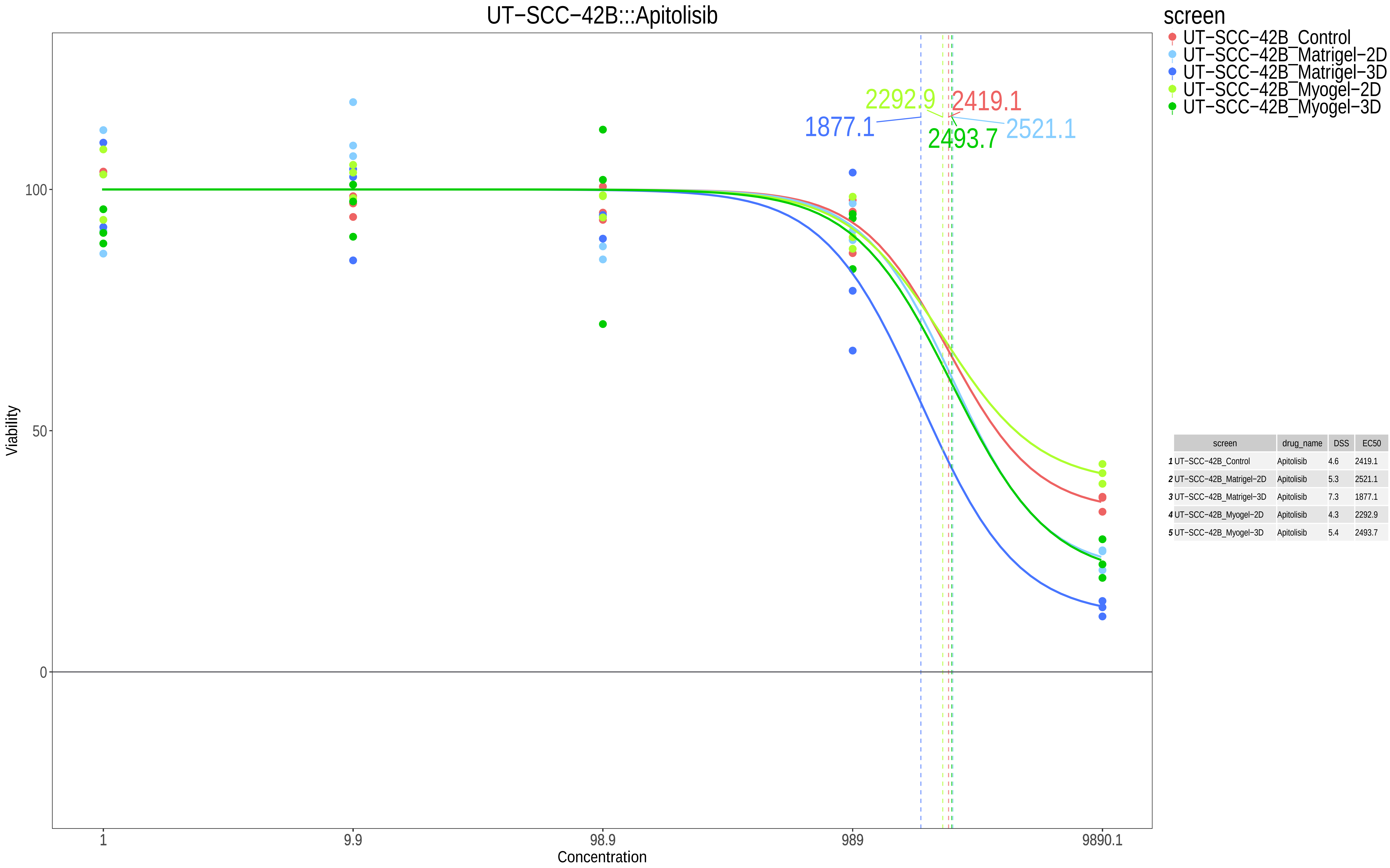

	screen	drug_name	DSS	EC50
1	UT−SCC−42B_Control	Apitolisib	4.6	2419.1
2	UT−SCC−42B_Matrigel−2D	Apitolisib	5.3	2521.1
3	UT−SCC−42B_Matrigel−3D	Apitolisib	7.3	1877.1
4	UT−SCC−42B_Myogel−2D	Apitolisib	4.3	2292.9
5	UT−SCC−42B_Myogel−3D	Apitolisib	5.4	2493.7

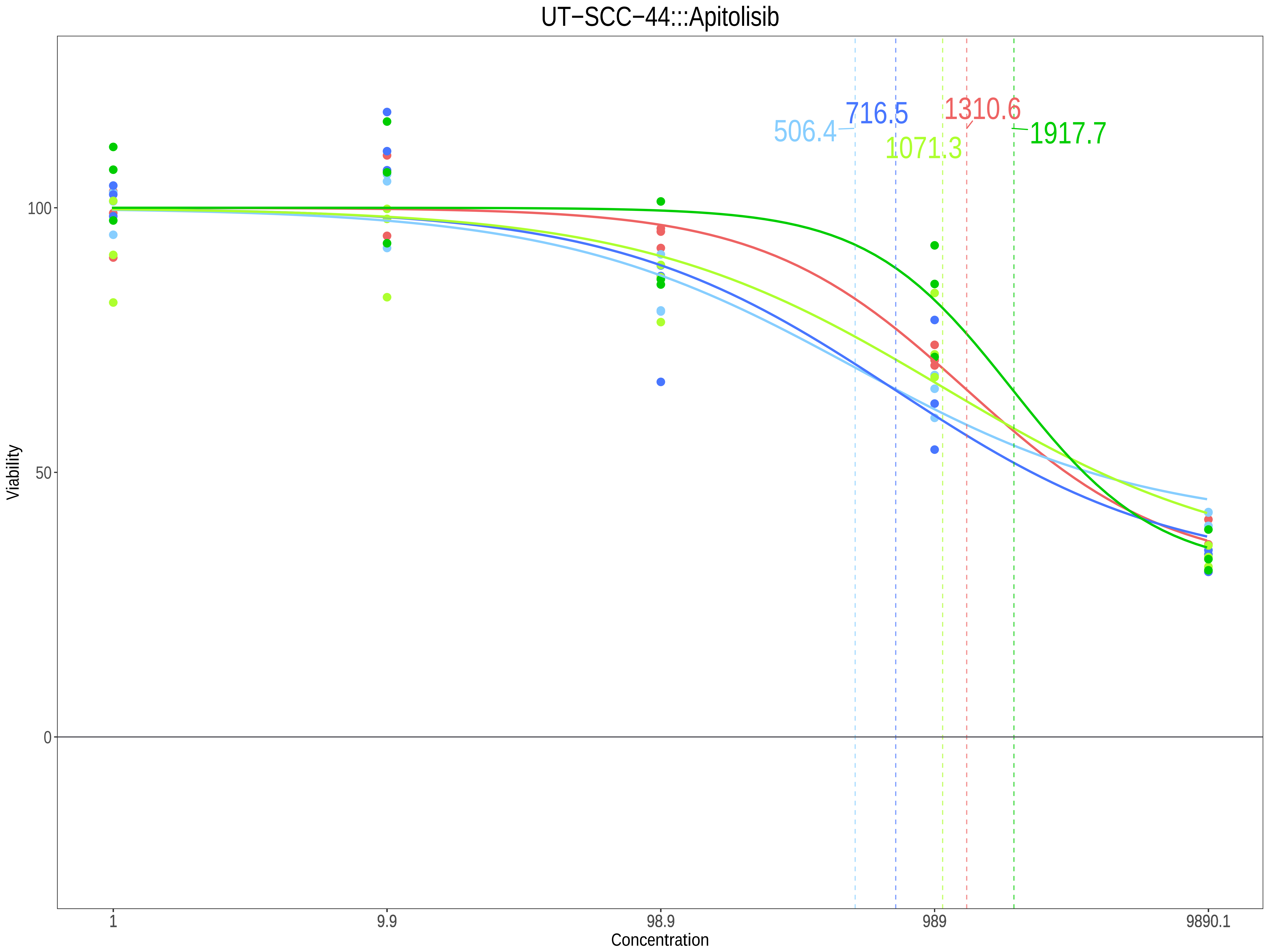

	screen	drug_name	DSS	EC50
1	UT−SCC−44_Control	Apitolisib	6.5	1310.6
2	UT−SCC−44_Matrigel−2D	Apitolisib	8.3	506.4
3	UT−SCC−44_Matrigel−3D	Apitolisib	8.5	716.5
4	UT−SCC−44_Myogel−2D	Apitolisib	7.0	1071.3
5	UT−SCC−44_Myogel−3D	Apitolisib	5.3	1917.7

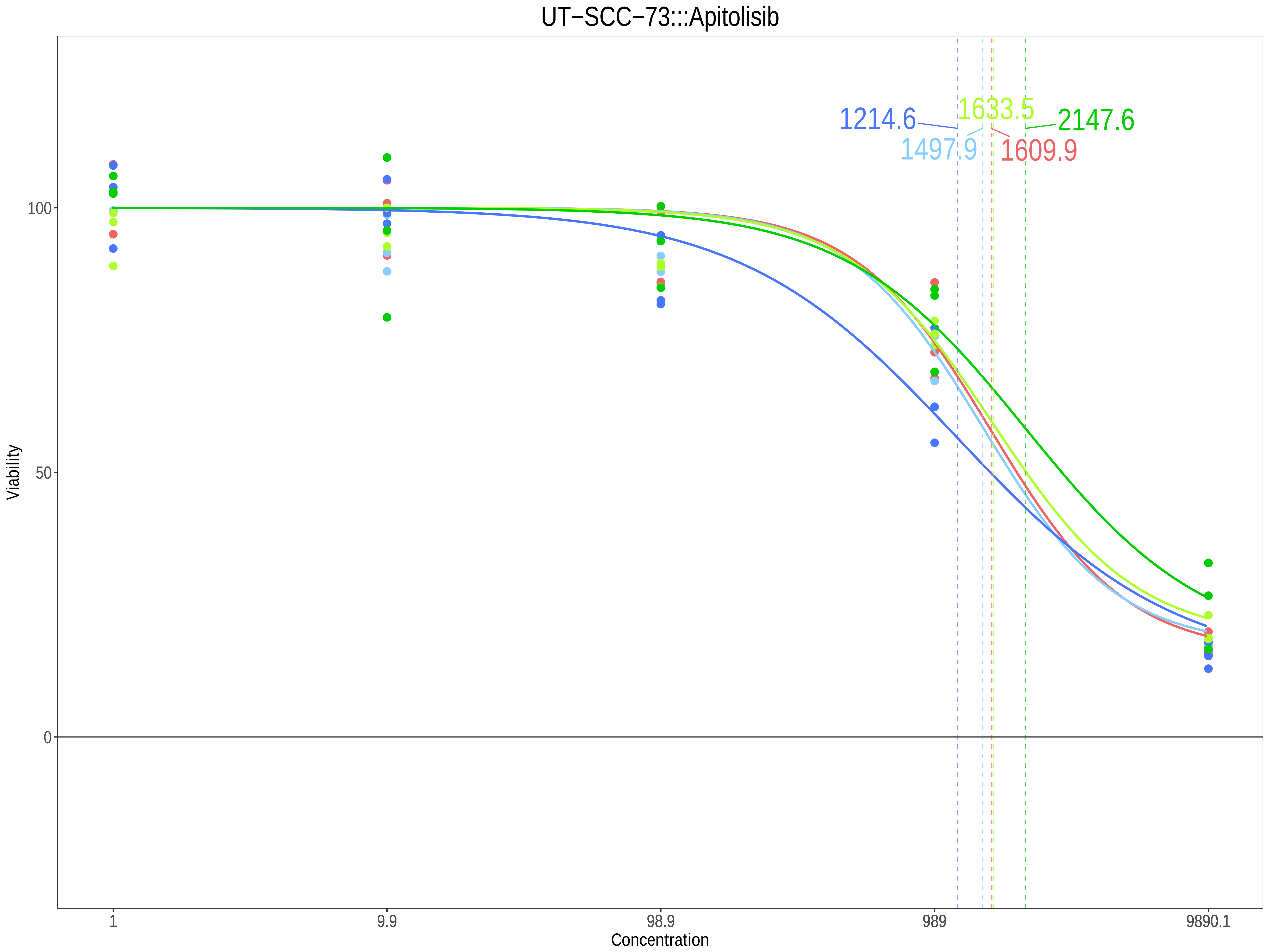

	screen	drug_name	DSS	EC50
1	UT−SCC−73_Control	Apitolisib	7.5	1609.9
2	UT−SCC−73_Matrigel−2D	Apitolisib	7.7	1497.9
3	UT−SCC−73_Matrigel−3D	Apitolisib	8.8	1214.6
4	UT−SCC−73_Myogel−2D	Apitolisib	7.1	1633.5
5	UT−SCC−73_Myogel−3D	Apitolisib	6.1	2147.6

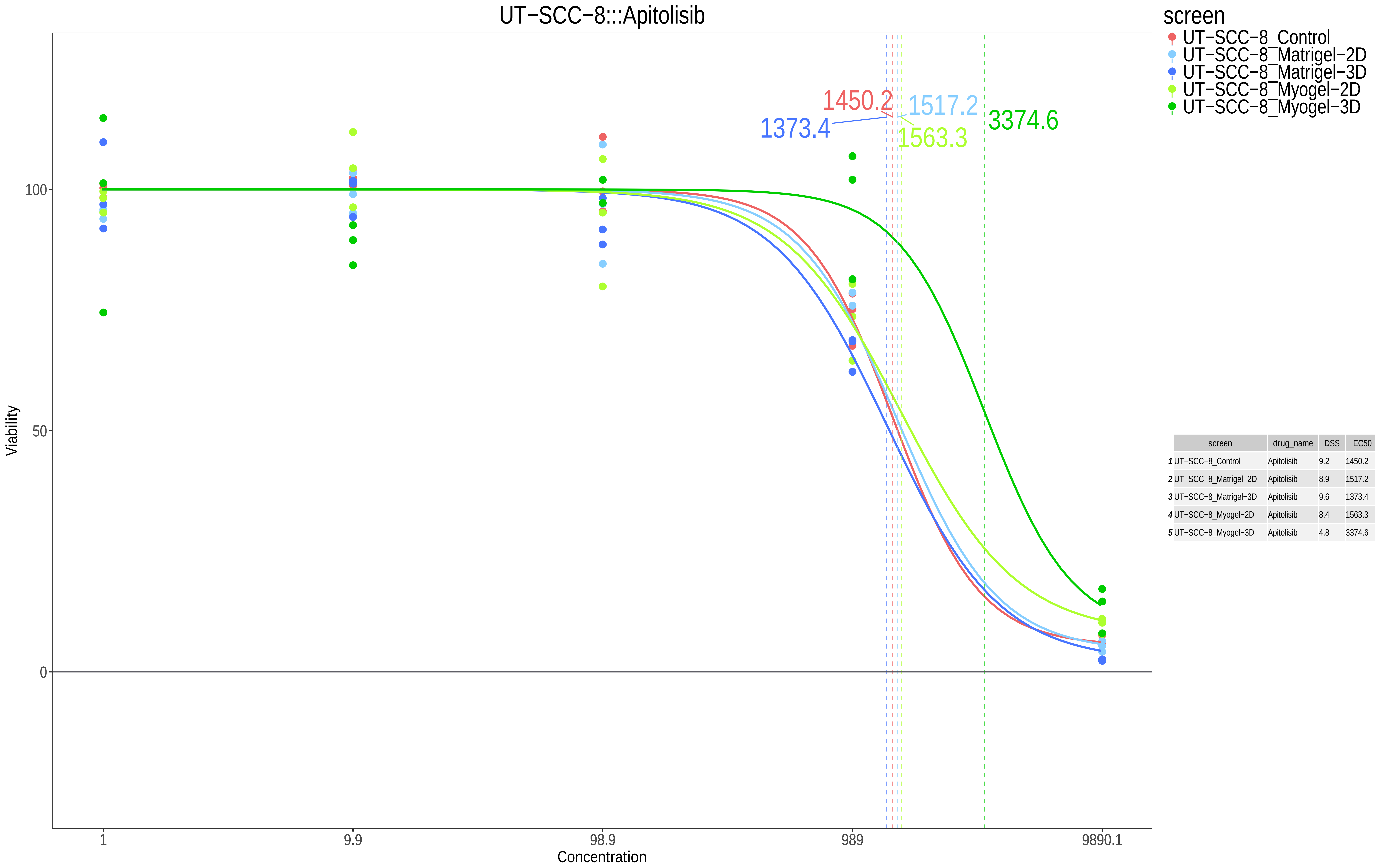

	screen	drug_name	DSS	EC50
1	UT−SCC−8_Control	Apitolisib	9.2	1450.2
2	UT−SCC−8_Matrigel−2D	Apitolisib	8.9	1517.2
3	UT−SCC−8_Matrigel−3D	Apitolisib	9.6	1373.4
4	UT−SCC−8_Myogel−2D	Apitolisib	8.4	1563.3
5	UT−SCC−8_Myogel−3D	Apitolisib	4.8	3374.6

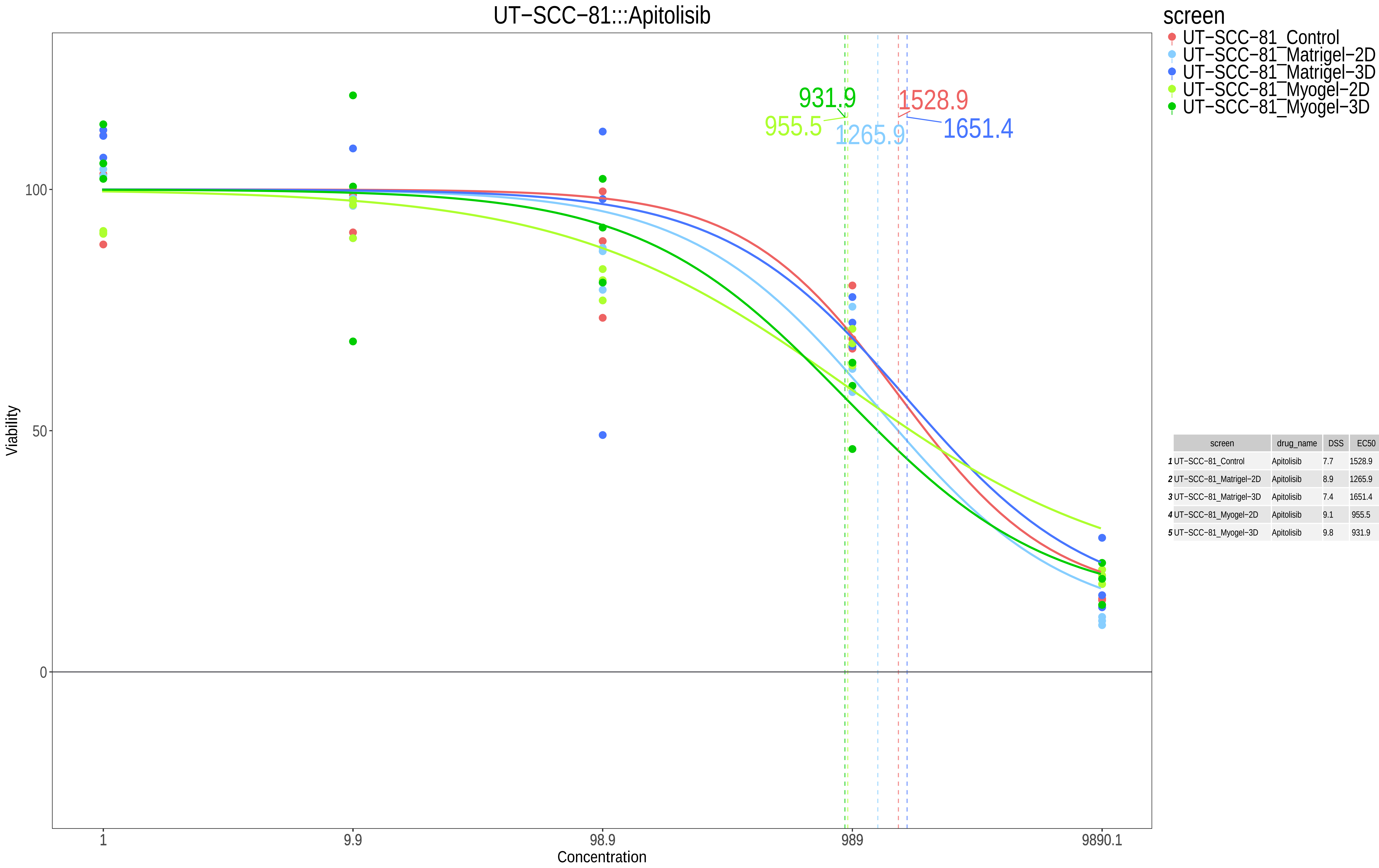

	screen	drug_name	DSS	EC50
1	UT−SCC−81_Control	Apitolisib	7.7	1528.9
2	UT−SCC−81_Matrigel−2D	Apitolisib	8.9	1265.9
3	UT−SCC−81_Matrigel−3D	Apitolisib	7.4	1651.4
4	UT−SCC−81_Myogel−2D	Apitolisib	9.1	955.5
5	UT−SCC−81_Myogel−3D	Apitolisib	9.8	931.9

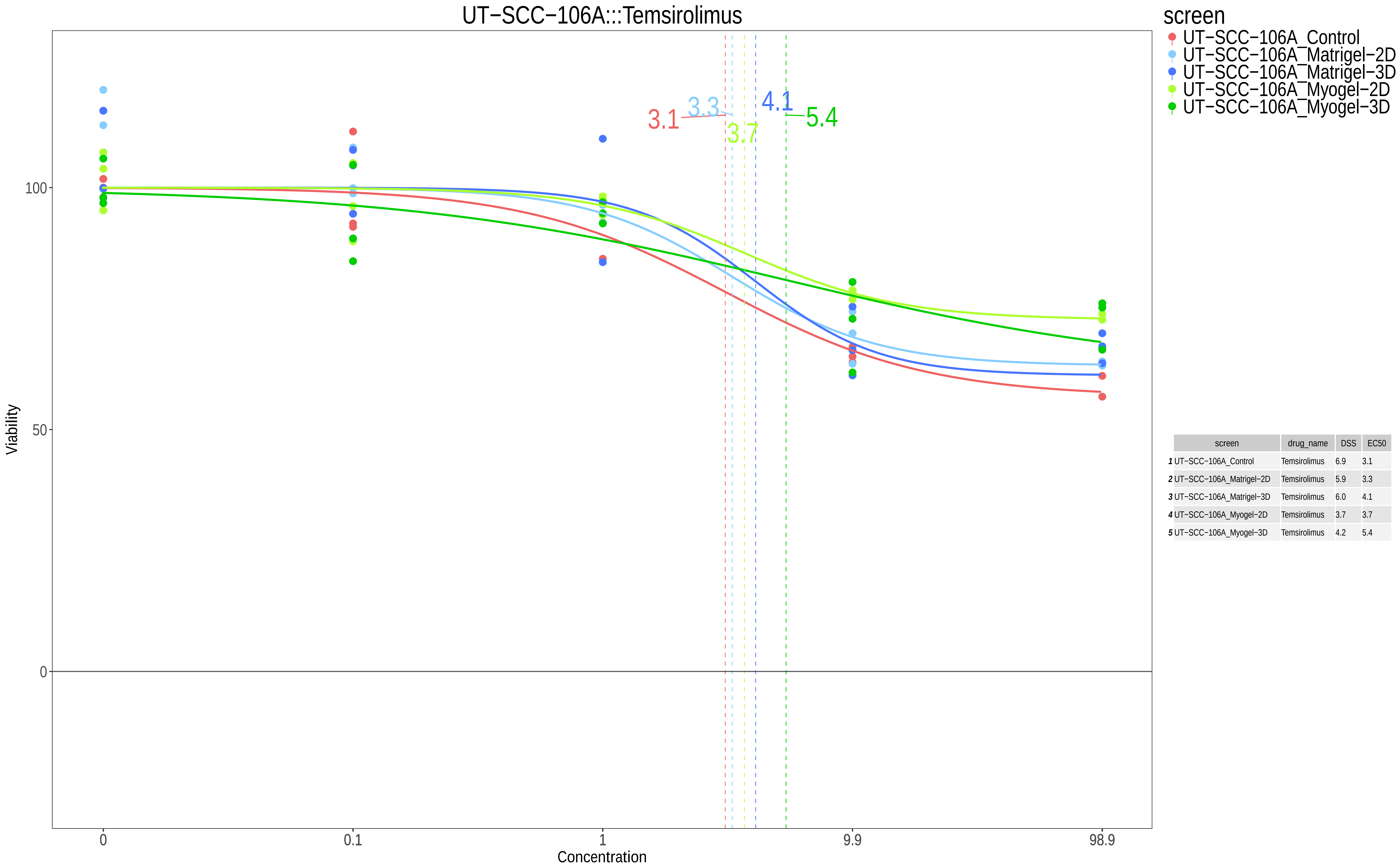

	screen	drug_name	DSS	EC50
1	UT−SCC−106A_Control	Temsirolimus	6.9	3.1
2	UT−SCC−106A_Matrigel−2D	Temsirolimus	5.9	3.3
3	UT−SCC−106A_Matrigel−3D	Temsirolimus	6.0	4.1
4	UT−SCC−106A_Myogel−2D	Temsirolimus	3.7	3.7
5	UT−SCC−106A_Myogel−3D	Temsirolimus	4.2	5.4

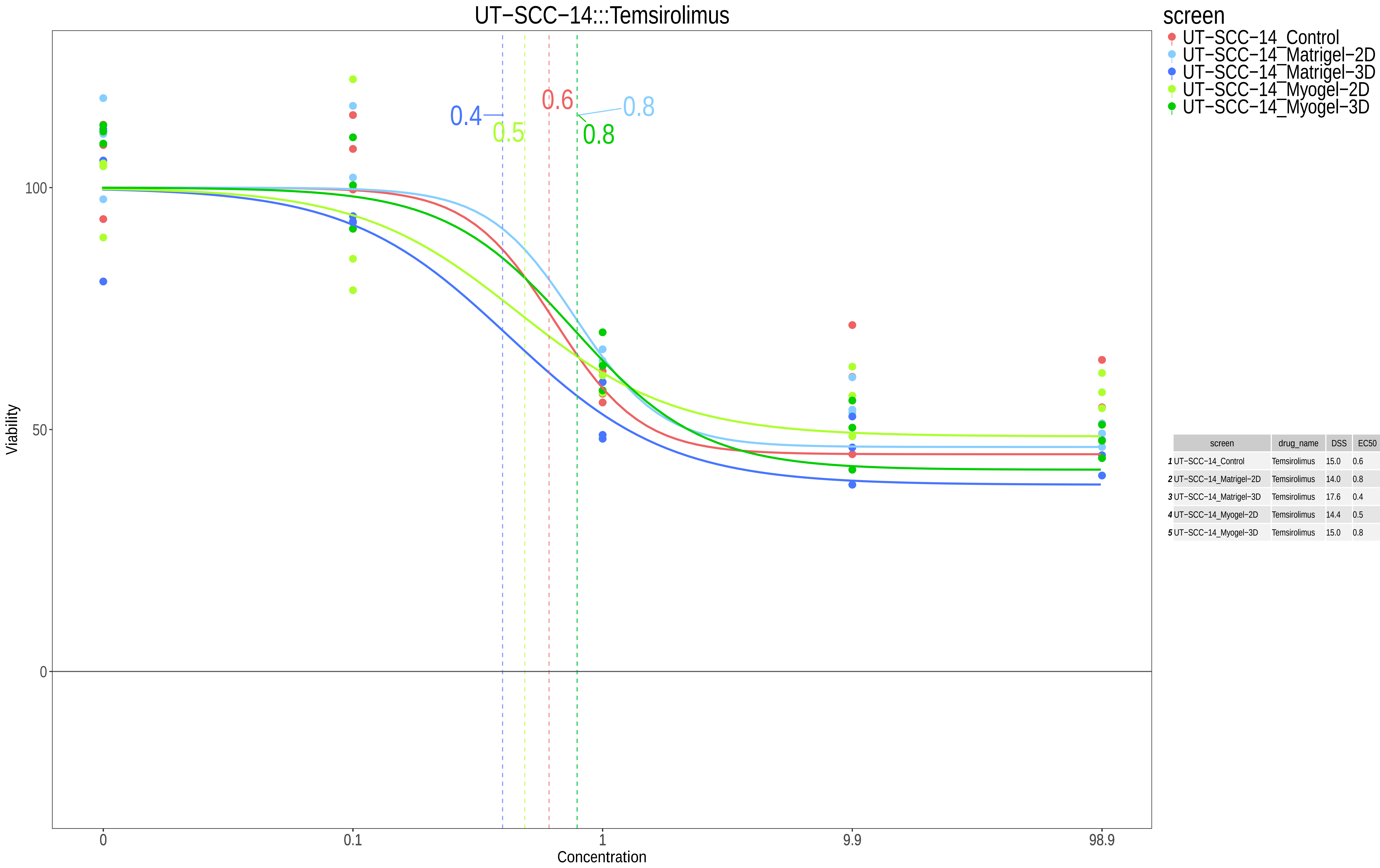

	screen	drug_name	DSS	EC50
1	UT−SCC−14_Control	Temsirolimus	15.0	0.6
2	UT−SCC−14_Matrigel−2D	Temsirolimus	14.0	0.8
3	UT−SCC−14_Matrigel−3D	Temsirolimus	17.6	0.4
4	UT−SCC−14_Myogel−2D	Temsirolimus	14.4	0.5
5	UT−SCC−14_Myogel−3D	Temsirolimus	15.0	0.8

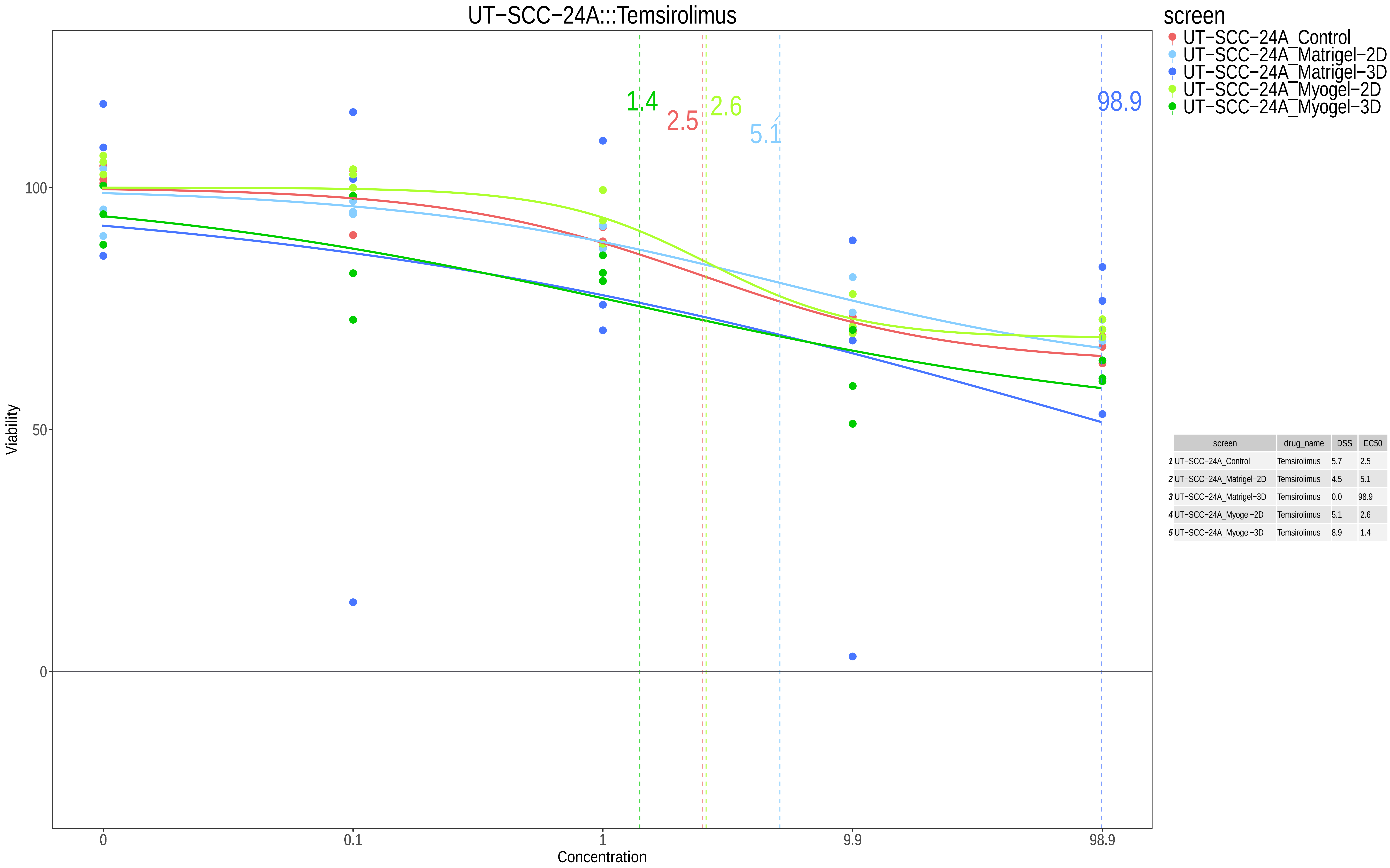

	screen	drug_name	DSS	EC50
1	UT−SCC−24A_Control	Temsirolimus	5.7	2.5
2	UT−SCC−24A_Matrigel−2D	Temsirolimus	4.5	5.1
3	UT−SCC−24A_Matrigel−3D	Temsirolimus	0.0	98.9
4	UT−SCC−24A_Myogel−2D	Temsirolimus	5.1	2.6
5	UT−SCC−24A_Myogel−3D	Temsirolimus	8.9	1.4

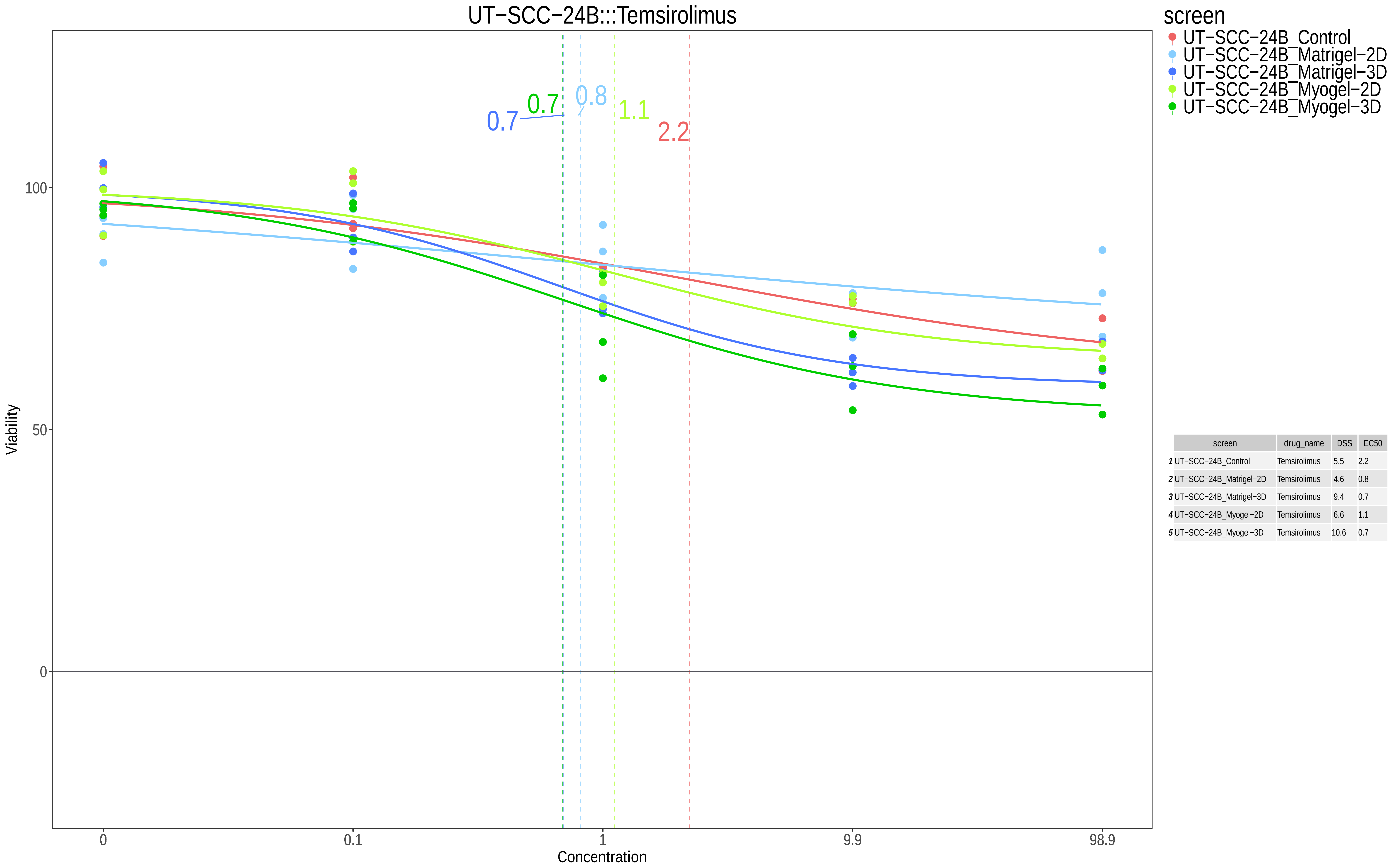

	screen	drug_name	DSS	EC50
1	UT−SCC−24B_Control	Temsirolimus	5.5	2.2
2	UT−SCC−24B_Matrigel−2D	Temsirolimus	4.6	0.8
3	UT−SCC−24B_Matrigel−3D	Temsirolimus	9.4	0.7
4	UT−SCC−24B_Myogel−2D	Temsirolimus	6.6	1.1
5	UT−SCC−24B_Myogel−3D	Temsirolimus	10.6	0.7

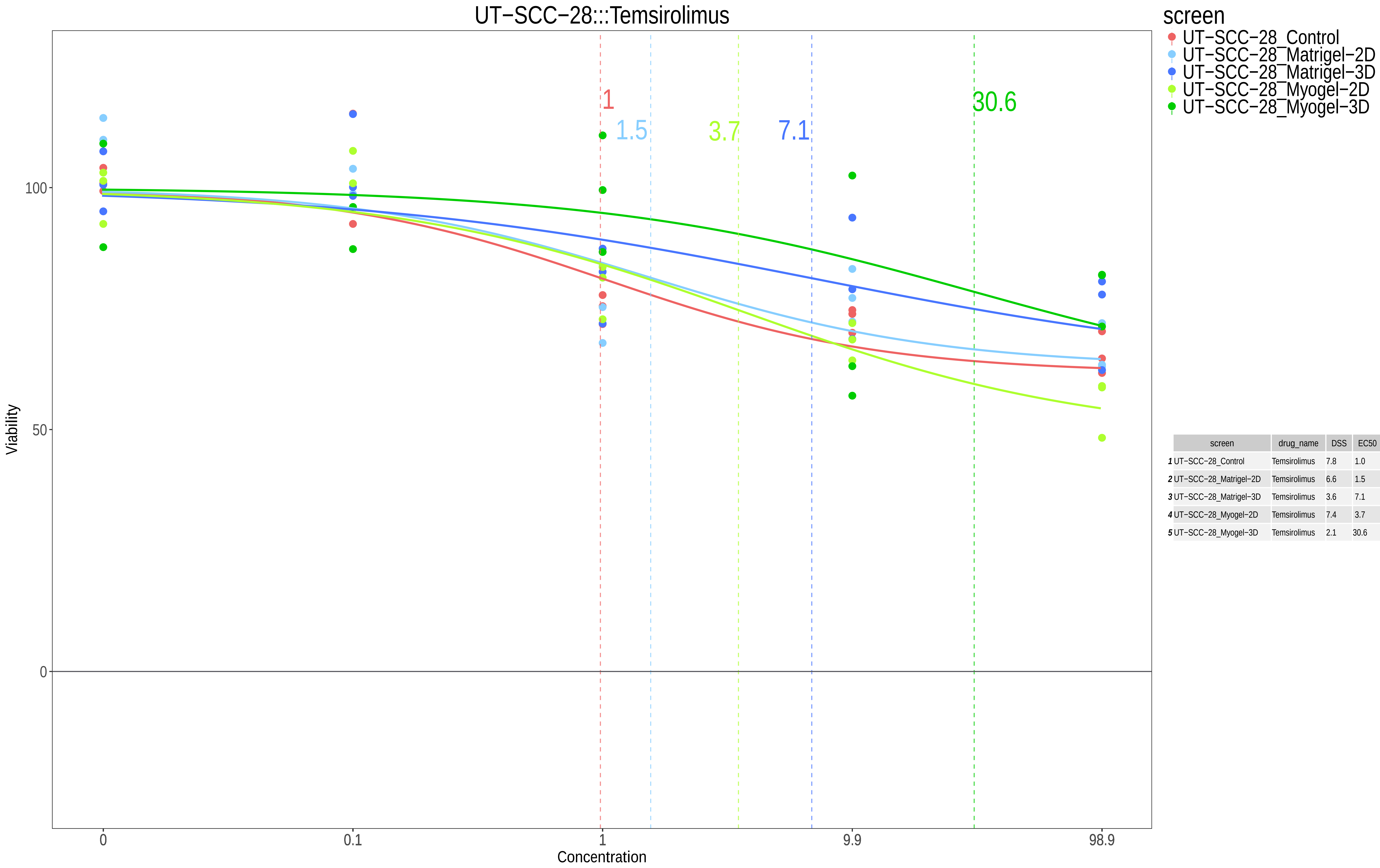

	screen	drug_name	DSS	EC50
1	UT−SCC−28_Control	Temsirolimus	7.8	1.0
2	UT−SCC−28_Matrigel−2D	Temsirolimus	6.6	1.5
3	UT−SCC−28_Matrigel−3D	Temsirolimus	3.6	7.1
4	UT−SCC−28_Myogel−2D	Temsirolimus	7.4	3.7
5	UT−SCC−28_Myogel−3D	Temsirolimus	2.1	30.6

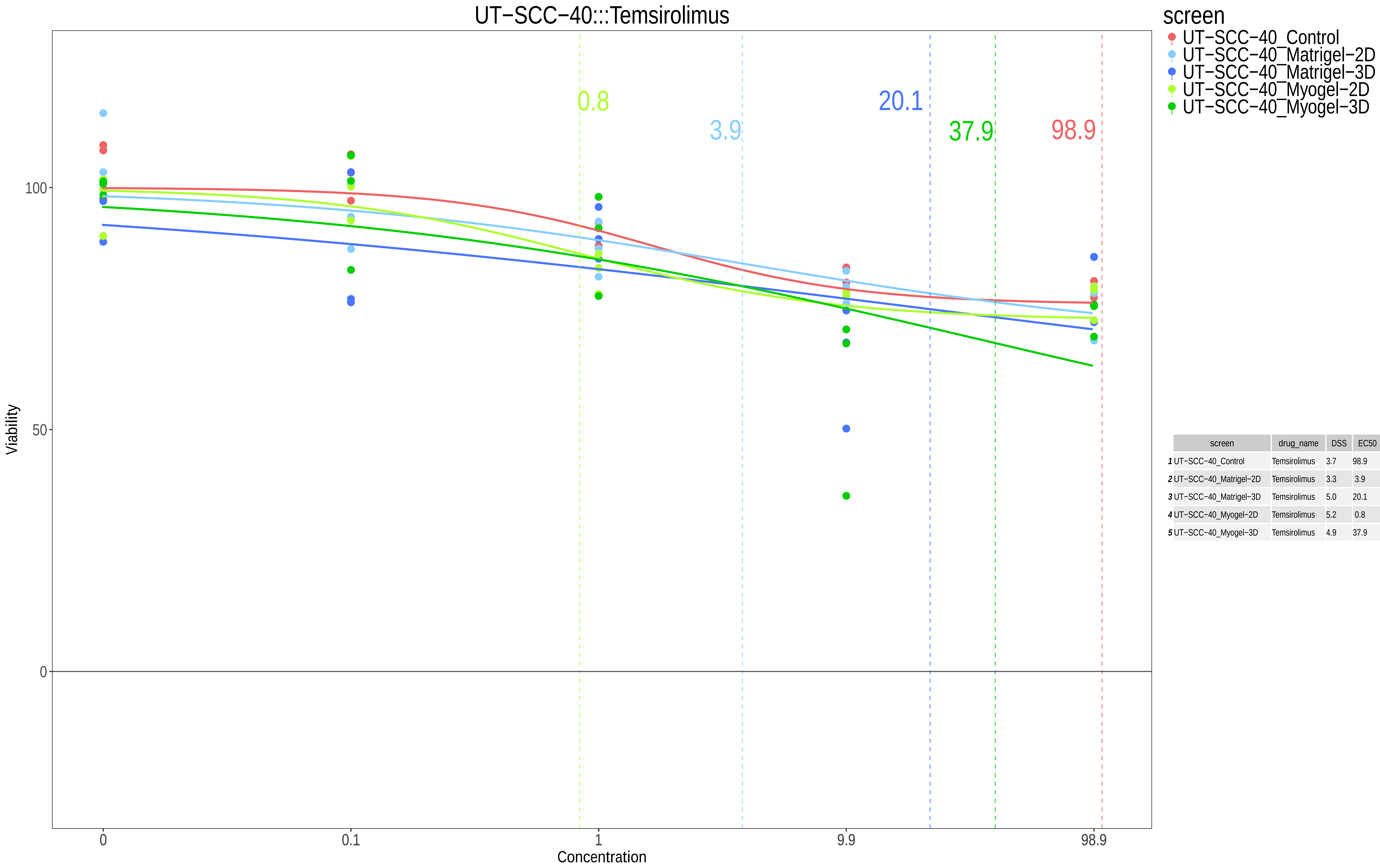

	screen	drug_name	DSS	EC50
1	UT−SCC−40_Control	Temsirolimus	3.7	98.9
2	UT−SCC−40_Matrigel−2D	Temsirolimus	3.3	3.9
3	UT−SCC−40_Matrigel−3D	Temsirolimus	5.0	20.1
4	UT−SCC−40_Myogel−2D	Temsirolimus	5.2	0.8
5	UT−SCC−40_Myogel−3D	Temsirolimus	4.9	37.9

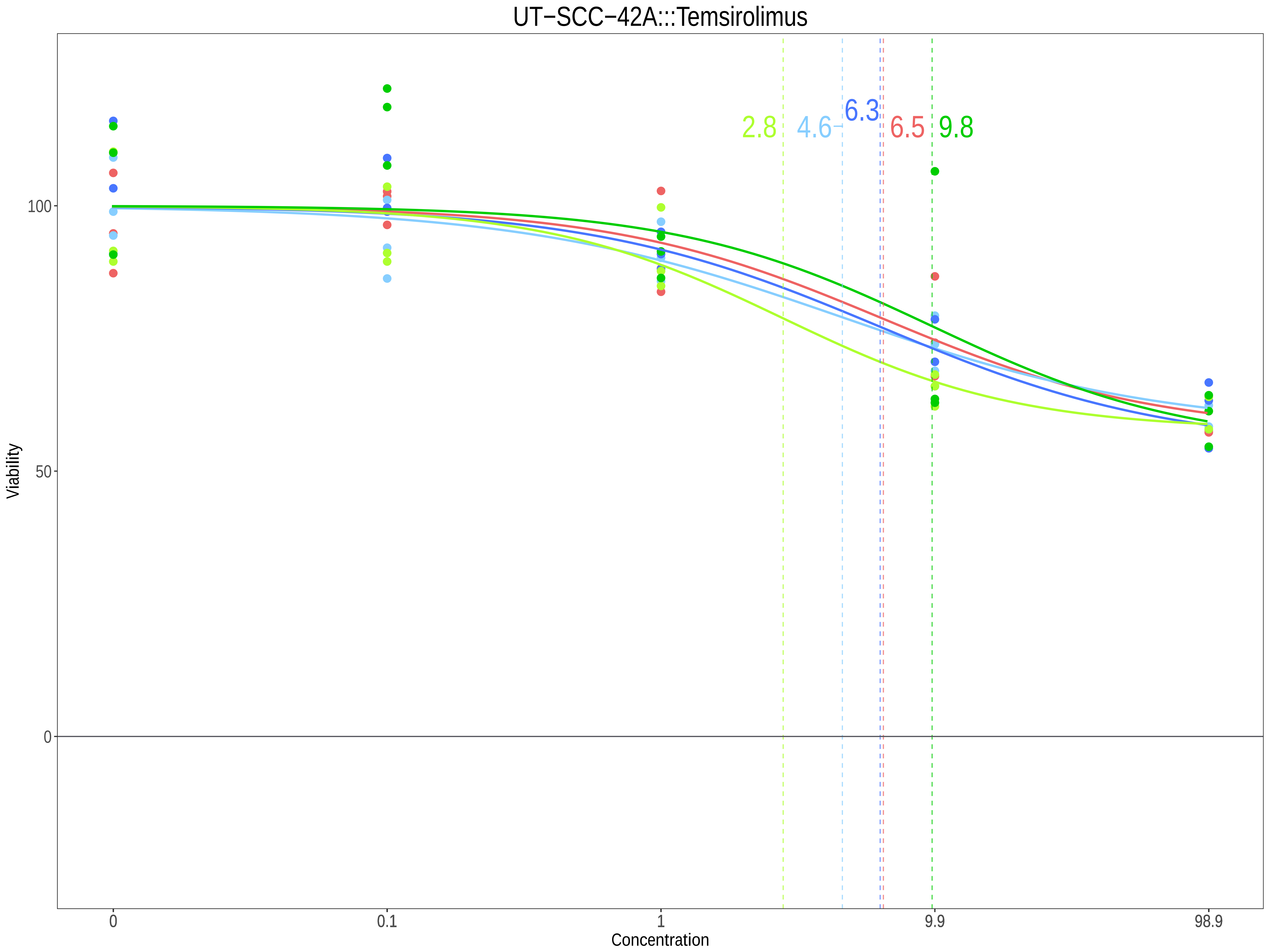

	screen	drug_name	DSS	EC50
1	UT−SCC−42A_Control	Temsirolimus	4.9	6.5
2	UT−SCC−42A_Matrigel−2D	Temsirolimus	5.4	4.6
3	UT−SCC−42A_Matrigel−3D	Temsirolimus	5.4	6.3
4	UT−SCC−42A_Myogel−2D	Temsirolimus	6.9	2.8
5	UT−SCC−42A_Myogel−3D	Temsirolimus	4.5	9.8

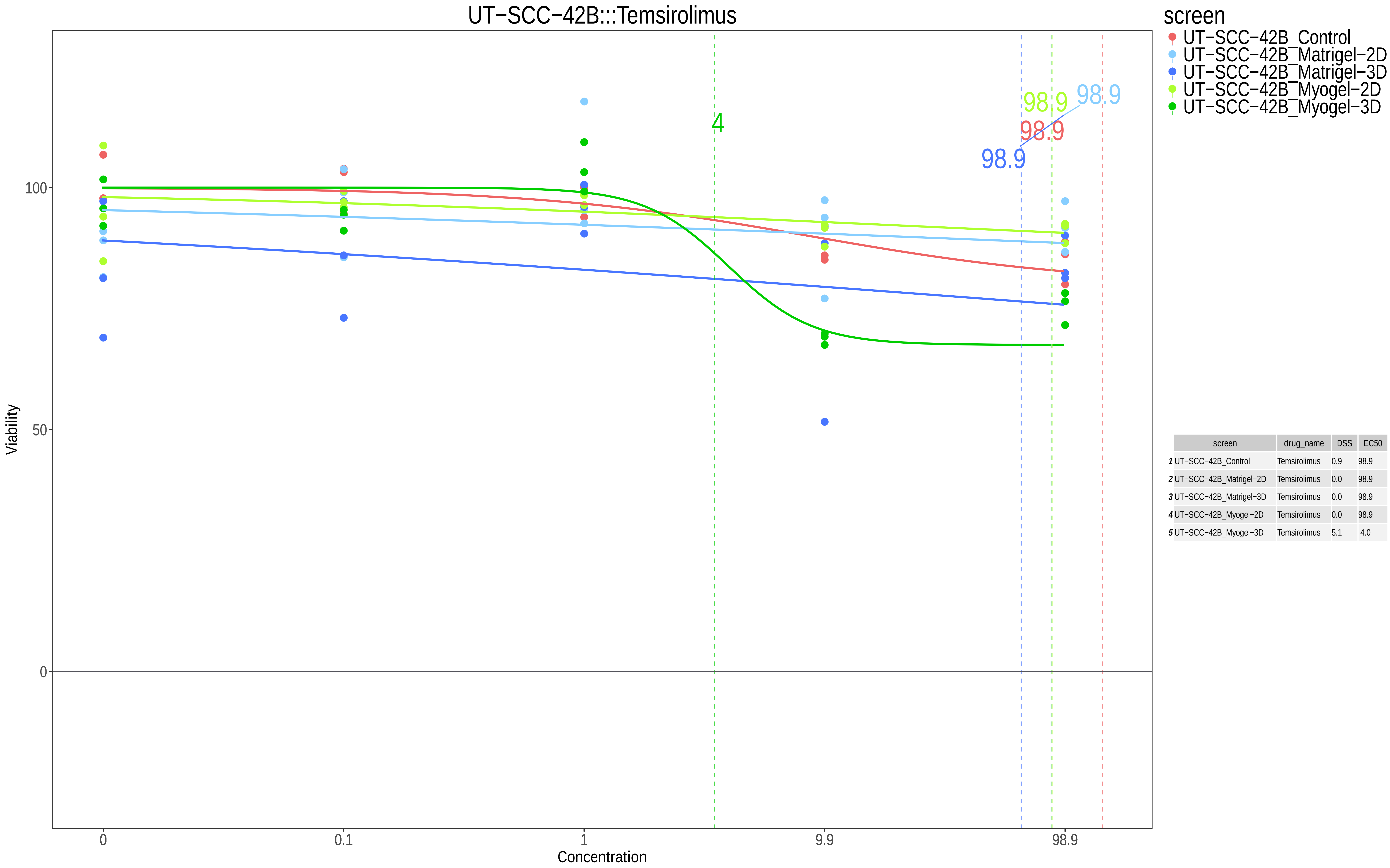

	screen	drug_name	DSS	EC50
1	UT−SCC−42B_Control	Temsirolimus	0.9	98.9
2	UT−SCC−42B_Matrigel−2D	Temsirolimus	0.0	98.9
3	UT−SCC−42B_Matrigel−3D	Temsirolimus	0.0	98.9
4	UT−SCC−42B_Myogel−2D	Temsirolimus	0.0	98.9
5	UT−SCC−42B_Myogel−3D	Temsirolimus	5.1	4.0

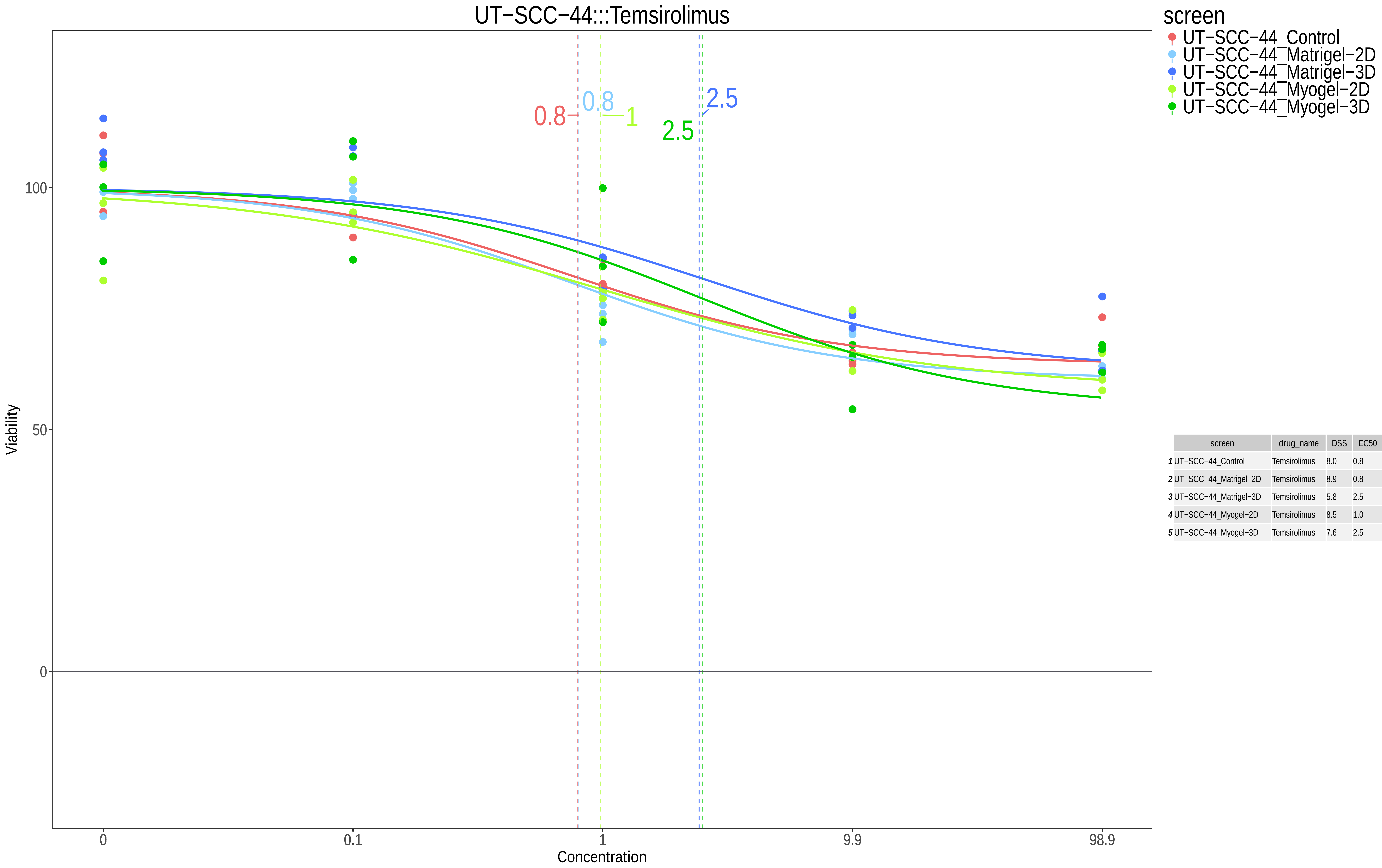

	screen	drug_name	DSS	EC50
1	UT−SCC−44_Control	Temsirolimus	8.0	0.8
2	UT−SCC−44_Matrigel−2D	Temsirolimus	8.9	0.8
3	UT−SCC−44_Matrigel−3D	Temsirolimus	5.8	2.5
4	UT−SCC−44_Myogel−2D	Temsirolimus	8.5	1.0
5	UT−SCC−44_Myogel−3D	Temsirolimus	7.6	2.5

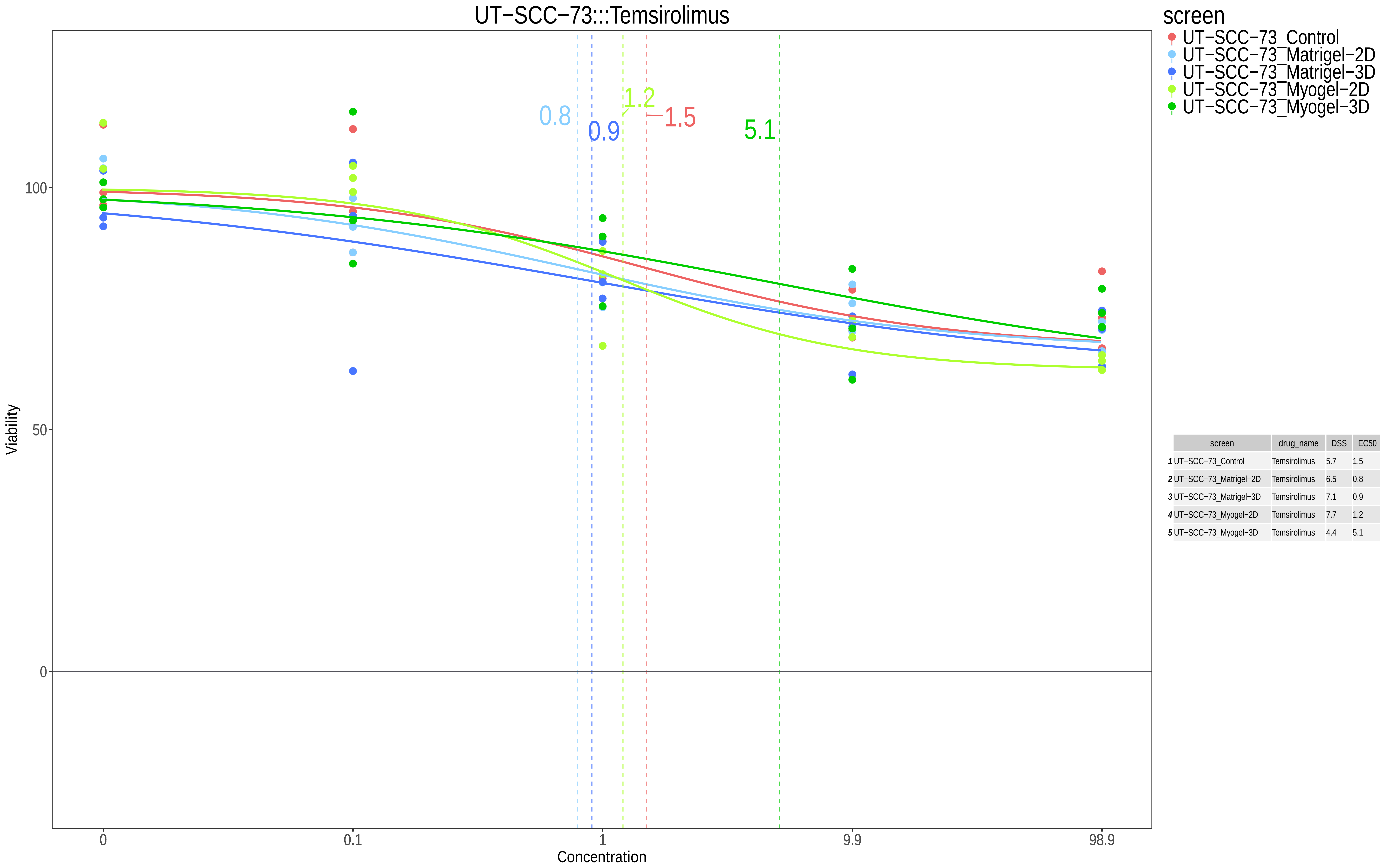

	screen	drug_name	DSS	EC50
1	UT−SCC−73_Control	Temsirolimus	5.7	1.5
2	UT−SCC−73_Matrigel−2D	Temsirolimus	6.5	0.8
3	UT−SCC−73_Matrigel−3D	Temsirolimus	7.1	0.9
4	UT−SCC−73_Myogel−2D	Temsirolimus	7.7	1.2
5	UT−SCC−73_Myogel−3D	Temsirolimus	4.4	5.1

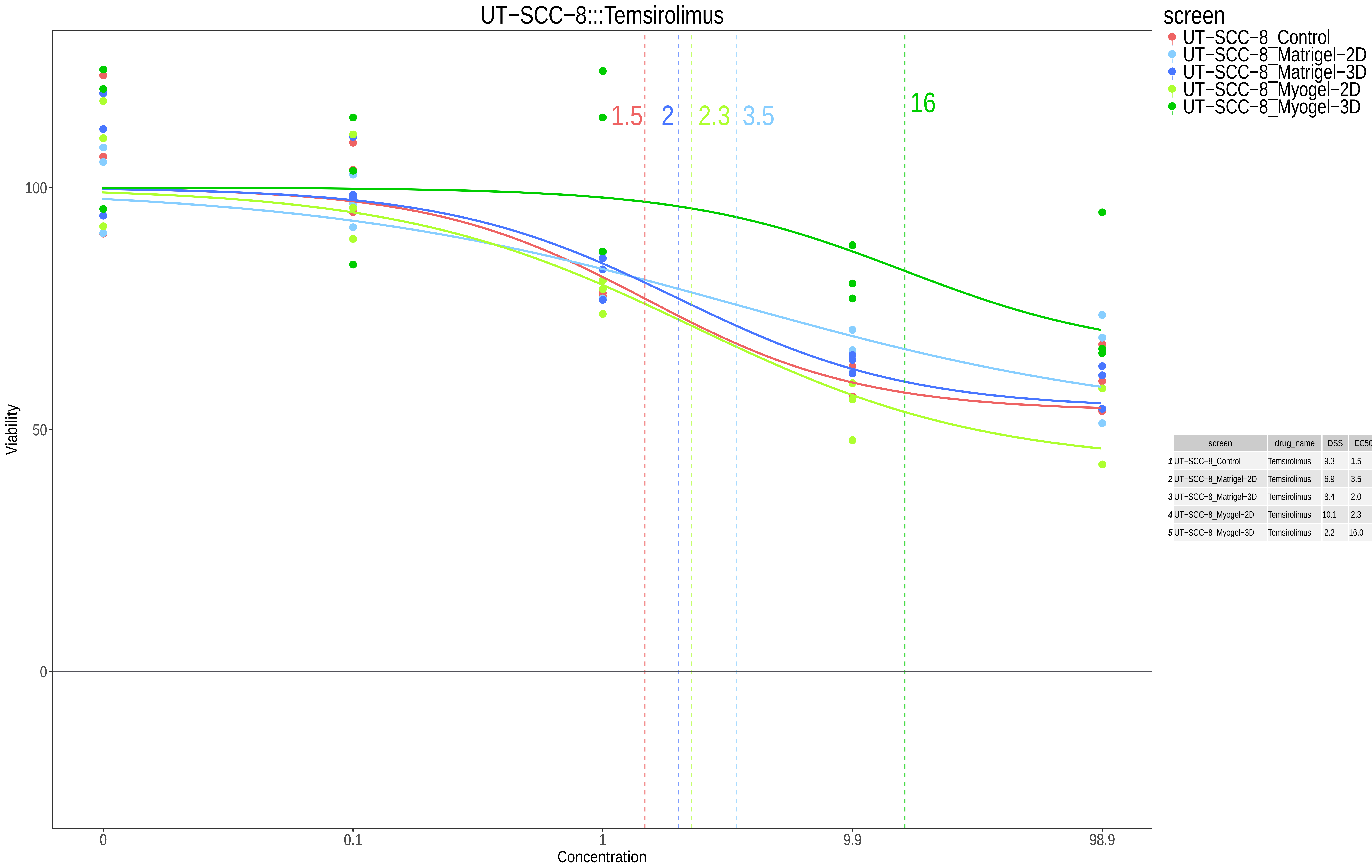

	screen	drug_name	DSS	EC50
1	UT−SCC−8_Control	Temsirolimus	9.3	1.5
2	UT−SCC−8_Matrigel−2D	Temsirolimus	6.9	3.5
3	UT−SCC−8_Matrigel−3D	Temsirolimus	8.4	2.0
4	UT−SCC−8_Myogel−2D	Temsirolimus	10.1	2.3
5	UT−SCC−8_Myogel−3D	Temsirolimus	2.2	16.0

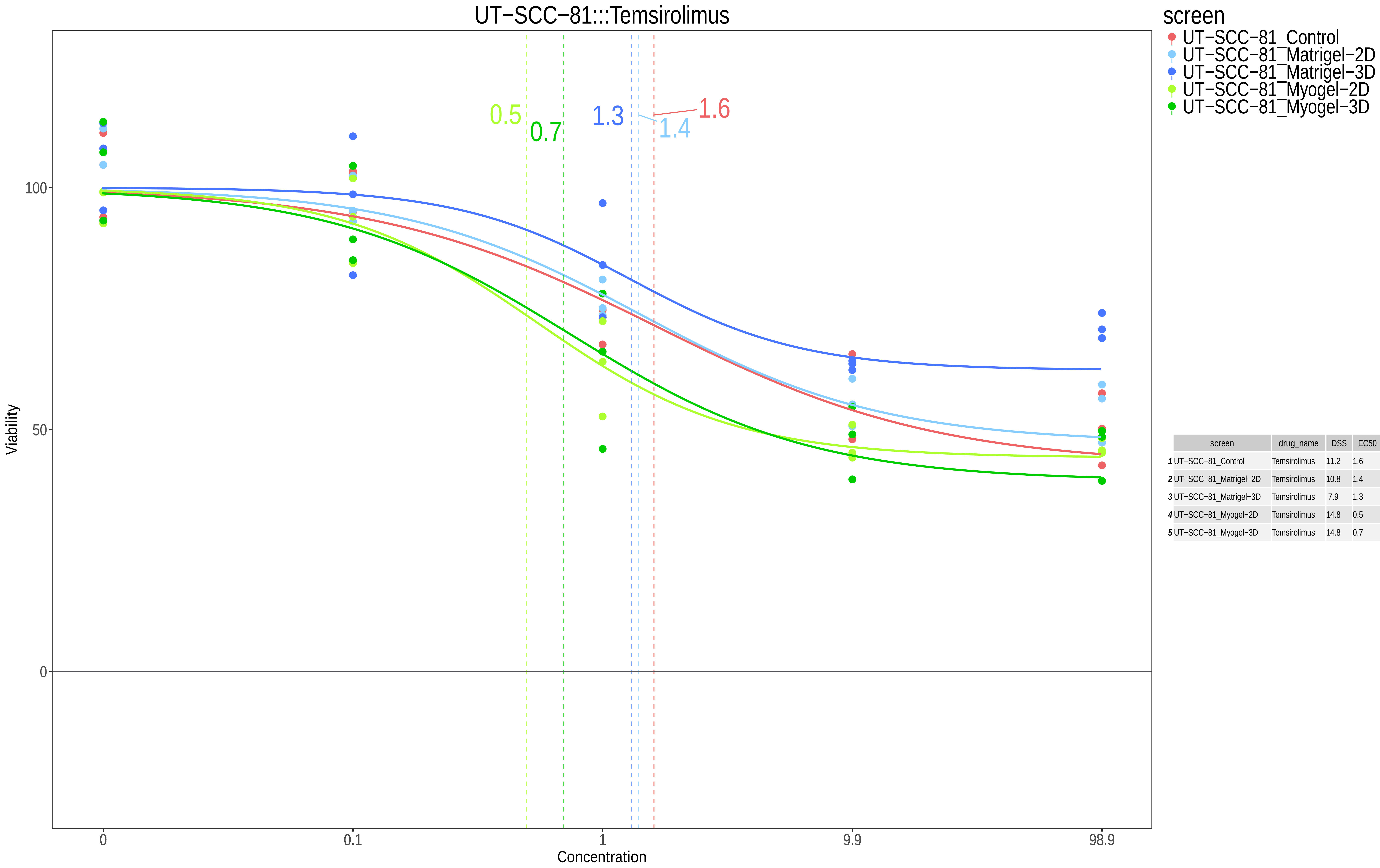

	screen	drug_name	DSS	EC50
1	UT−SCC−81_Control	Temsirolimus	11.2	1.6
2	UT−SCC−81_Matrigel−2D	Temsirolimus	10.8	1.4
3	UT−SCC−81_Matrigel−3D	Temsirolimus	7.9	1.3
4	UT−SCC−81_Myogel−2D	Temsirolimus	14.8	0.5
5	UT−SCC−81_Myogel−3D	Temsirolimus	14.8	0.7

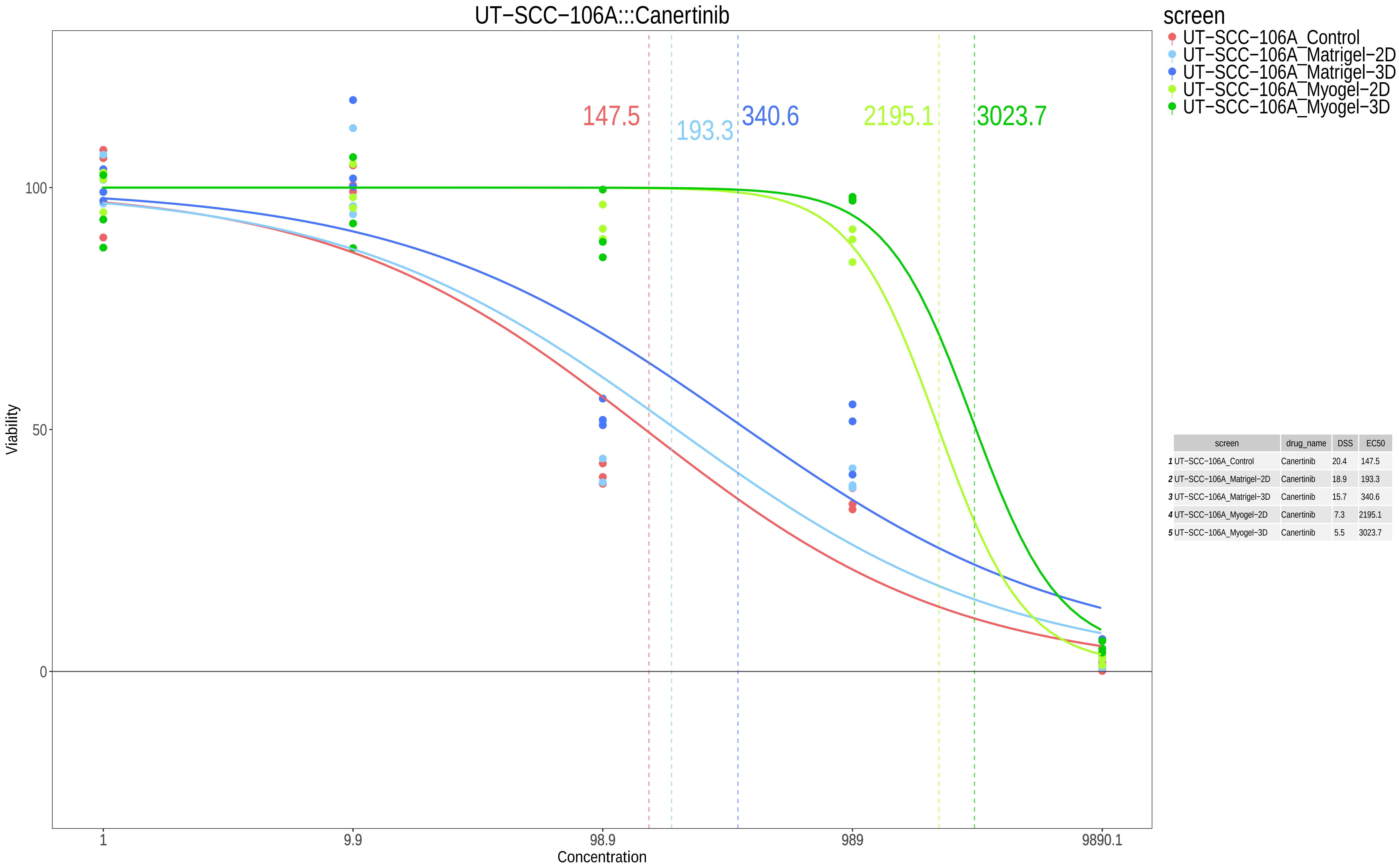

	screen	drug_name	DSS	EC50
1	UT−SCC−106A_Control	Canertinib	20.4	147.5
2	UT−SCC−106A_Matrigel−2D	Canertinib	18.9	193.3
3	UT−SCC−106A_Matrigel−3D	Canertinib	15.7	340.6
4	UT−SCC−106A_Myogel−2D	Canertinib	7.3	2195.1
5	UT−SCC−106A_Myogel−3D	Canertinib	5.5	3023.7

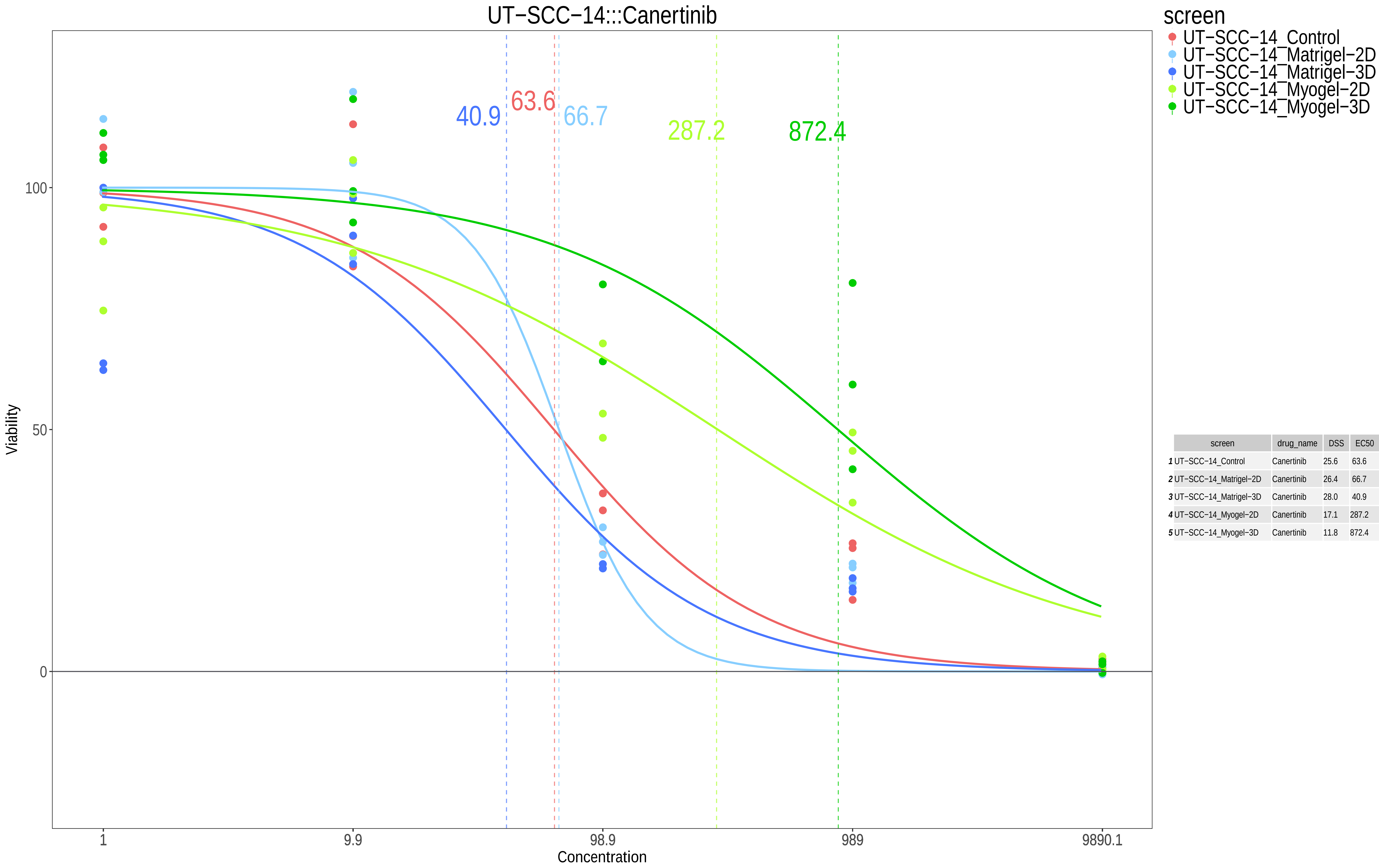

	screen	drug_name	DSS	EC50
1	UT−SCC−14_Control	Canertinib	25.6	63.6
2	UT−SCC−14_Matrigel−2D	Canertinib	26.4	66.7
3	UT−SCC−14_Matrigel−3D	Canertinib	28.0	40.9
4	UT−SCC−14_Myogel−2D	Canertinib	17.1	287.2
5	UT−SCC−14_Myogel−3D	Canertinib	11.8	872.4

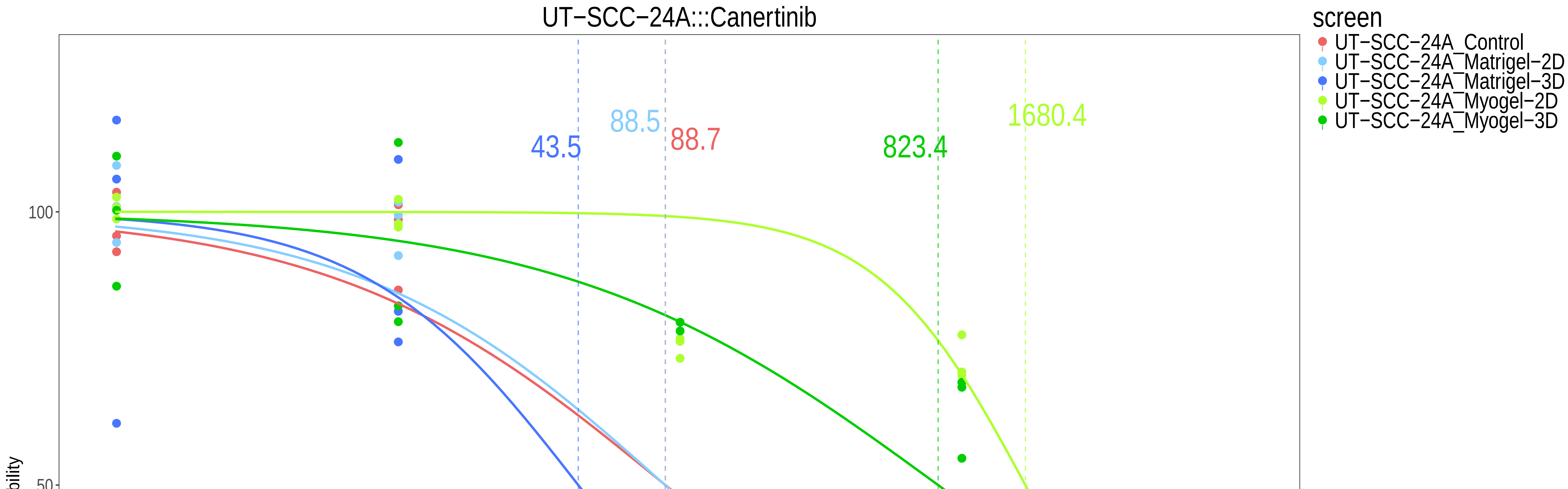

	screen	drug_name	DSS	EC50
1	UT−SCC−24A_Control	Canertinib	23.2	88.7
2	UT−SCC−24A_Matrigel−2D	Canertinib	23.3	88.5
3	UT−SCC−24A_Matrigel−3D	Canertinib	27.8	43.5
4	UT−SCC−24A_Myogel−2D	Canertinib	8.6	1680.4
5	UT−SCC−24A_Myogel−3D	Canertinib	12.2	823.4

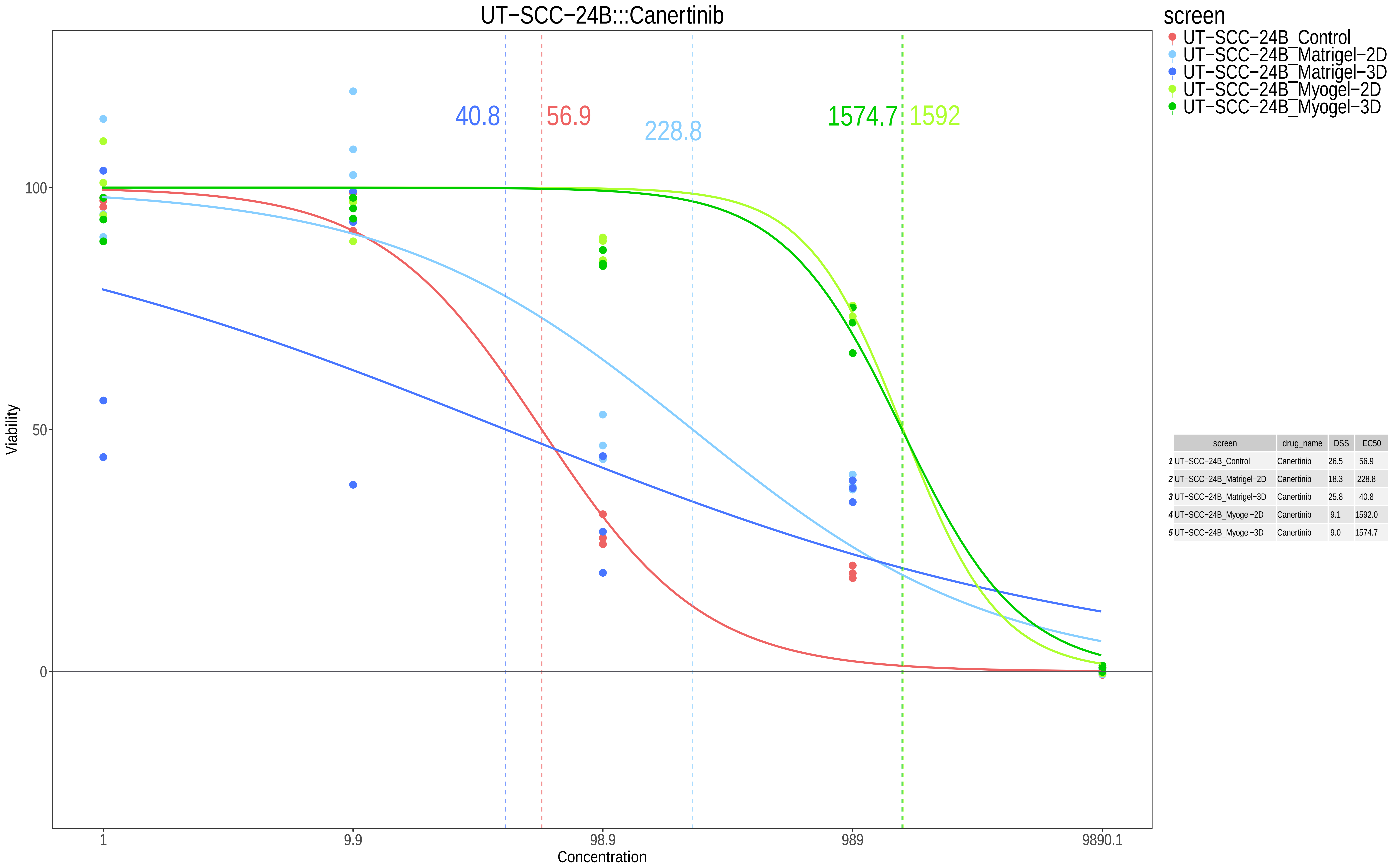

	screen	drug_name	DSS	EC50
1	UT−SCC−24B_Control	Canertinib	26.5	56.9
2	UT−SCC−24B_Matrigel−2D	Canertinib	18.3	228.8
3	UT−SCC−24B_Matrigel−3D	Canertinib	25.8	40.8
4	UT−SCC−24B_Myogel−2D	Canertinib	9.1	1592.0
5	UT−SCC−24B_Myogel−3D	Canertinib	9.0	1574.7

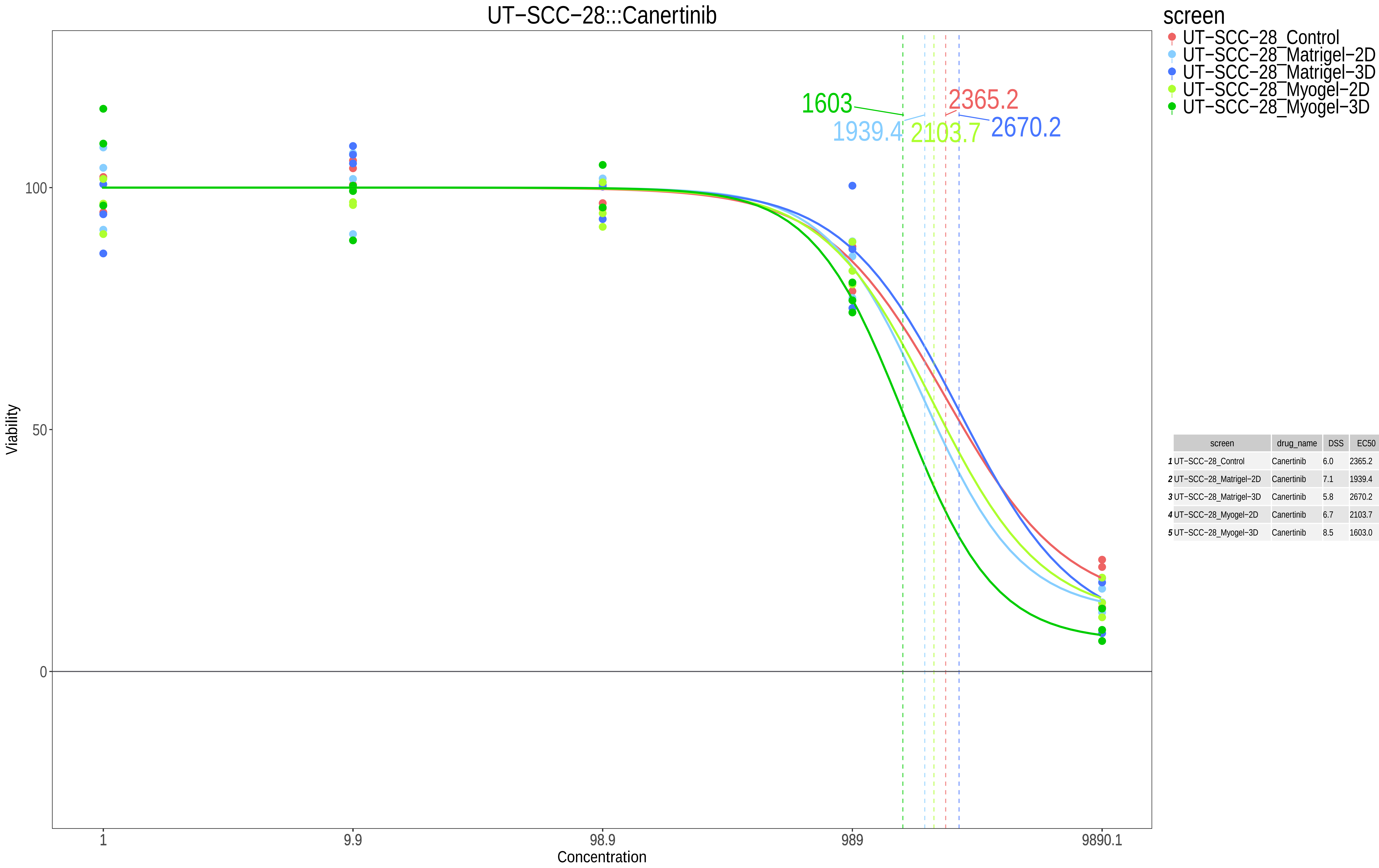

	screen	drug_name	DSS	EC50
1	UT−SCC−28_Control	Canertinib	6.0	2365.2
2	UT−SCC−28_Matrigel−2D	Canertinib	7.1	1939.4
3	UT−SCC−28_Matrigel−3D	Canertinib	5.8	2670.2
4	UT−SCC−28_Myogel−2D	Canertinib	6.7	2103.7
5	UT−SCC−28_Myogel−3D	Canertinib	8.5	1603.0

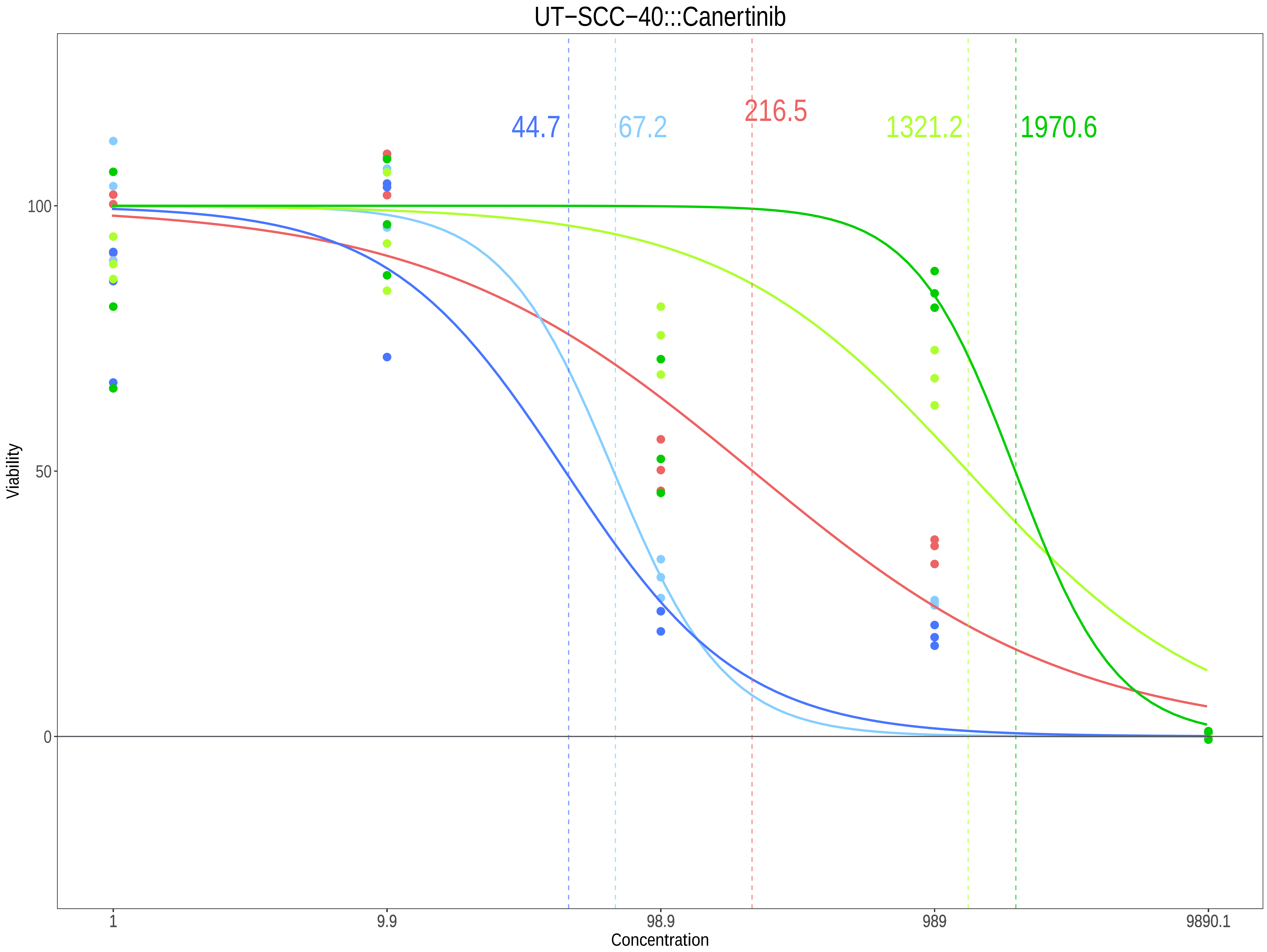

	screen	drug_name	DSS	EC50
1	UT−SCC−40_Control	Canertinib	18.6	216.5
2	UT−SCC−40_Matrigel−2D	Canertinib	26.2	67.2
3	UT−SCC−40_Matrigel−3D	Canertinib	27.9	44.7
4	UT−SCC−40_Myogel−2D	Canertinib	9.7	1321.2
5	UT−SCC−40_Myogel−3D	Canertinib	8.0	1970.6

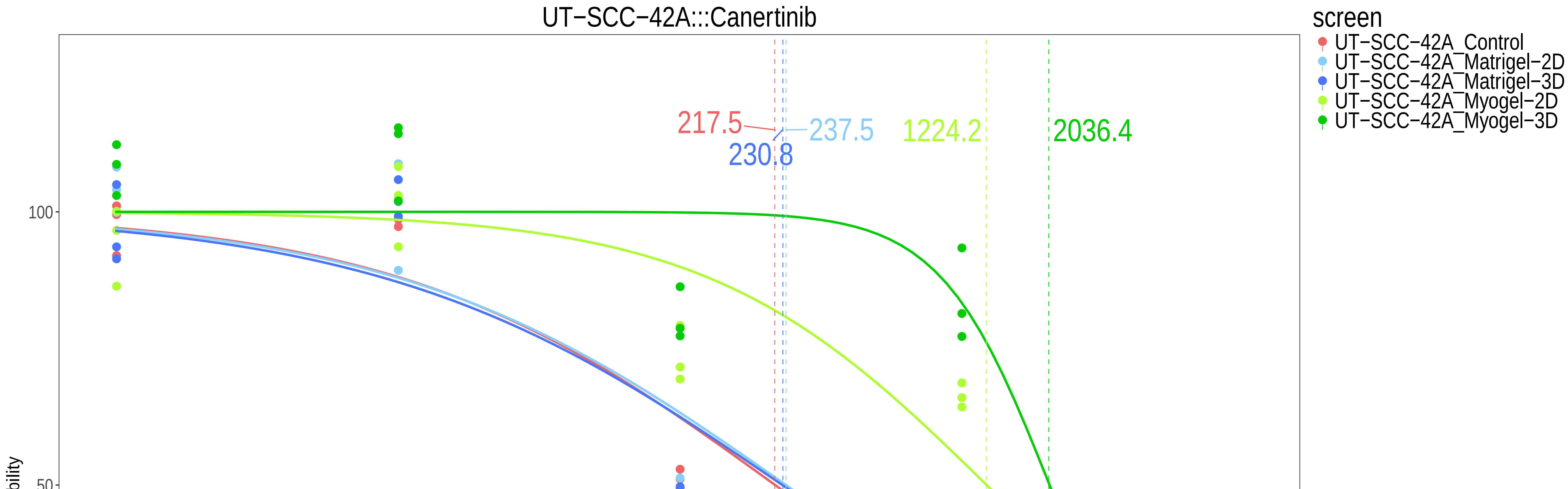

	screen	drug_name	DSS	EC50
1	UT−SCC−42A_Control	Canertinib	18.5	217.5
2	UT−SCC−42A_Matrigel−2D	Canertinib	18.0	237.5
3	UT−SCC−42A_Matrigel−3D	Canertinib	18.1	230.8
4	UT−SCC−42A_Myogel−2D	Canertinib	10.1	1224.2
5	UT−SCC−42A_Myogel−3D	Canertinib	7.7	2036.4

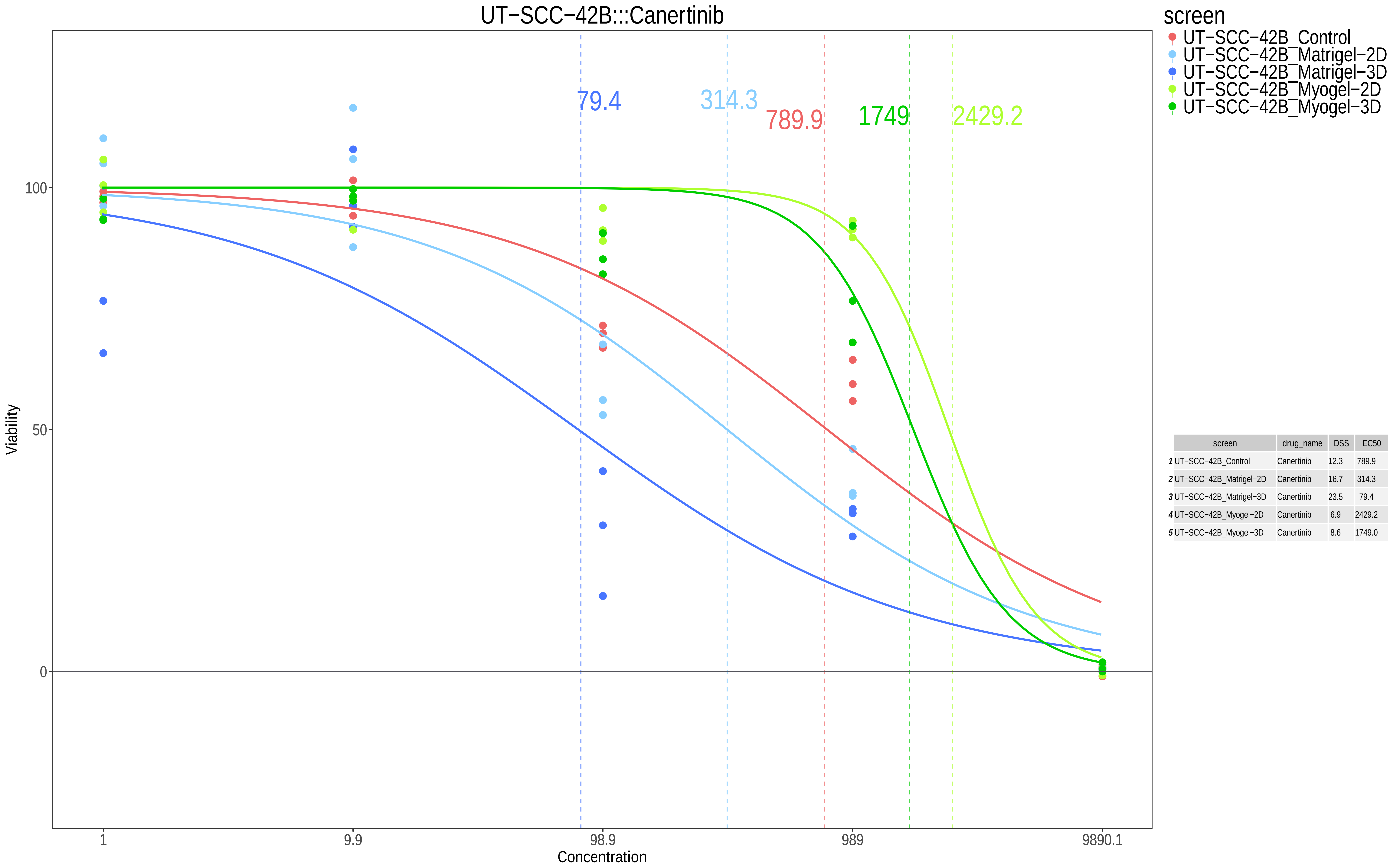

	screen	drug_name	DSS	EC50
1	UT−SCC−42B_Control	Canertinib	12.3	789.9
2	UT−SCC−42B_Matrigel−2D	Canertinib	16.7	314.3
3	UT−SCC−42B_Matrigel−3D	Canertinib	23.5	79.4
4	UT−SCC−42B_Myogel−2D	Canertinib	6.9	2429.2
5	UT−SCC−42B_Myogel−3D	Canertinib	8.6	1749.0

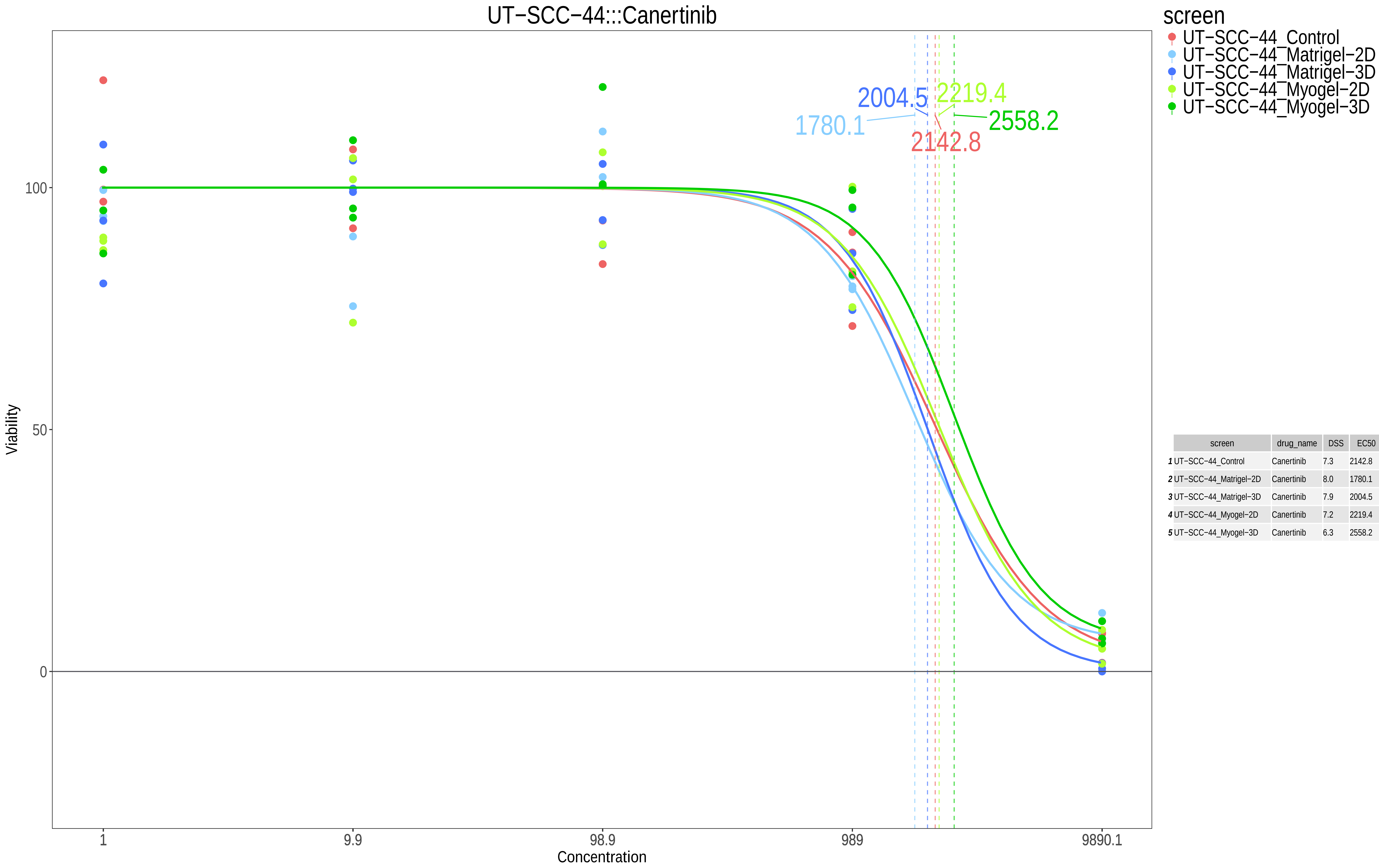

	screen	drug_name	DSS	EC50
1	UT−SCC−44_Control	Canertinib	7.3	2142.8
2	UT−SCC−44_Matrigel−2D	Canertinib	8.0	1780.1
3	UT−SCC−44_Matrigel−3D	Canertinib	7.9	2004.5
4	UT−SCC−44_Myogel−2D	Canertinib	7.2	2219.4
5	UT−SCC−44_Myogel−3D	Canertinib	6.3	2558.2

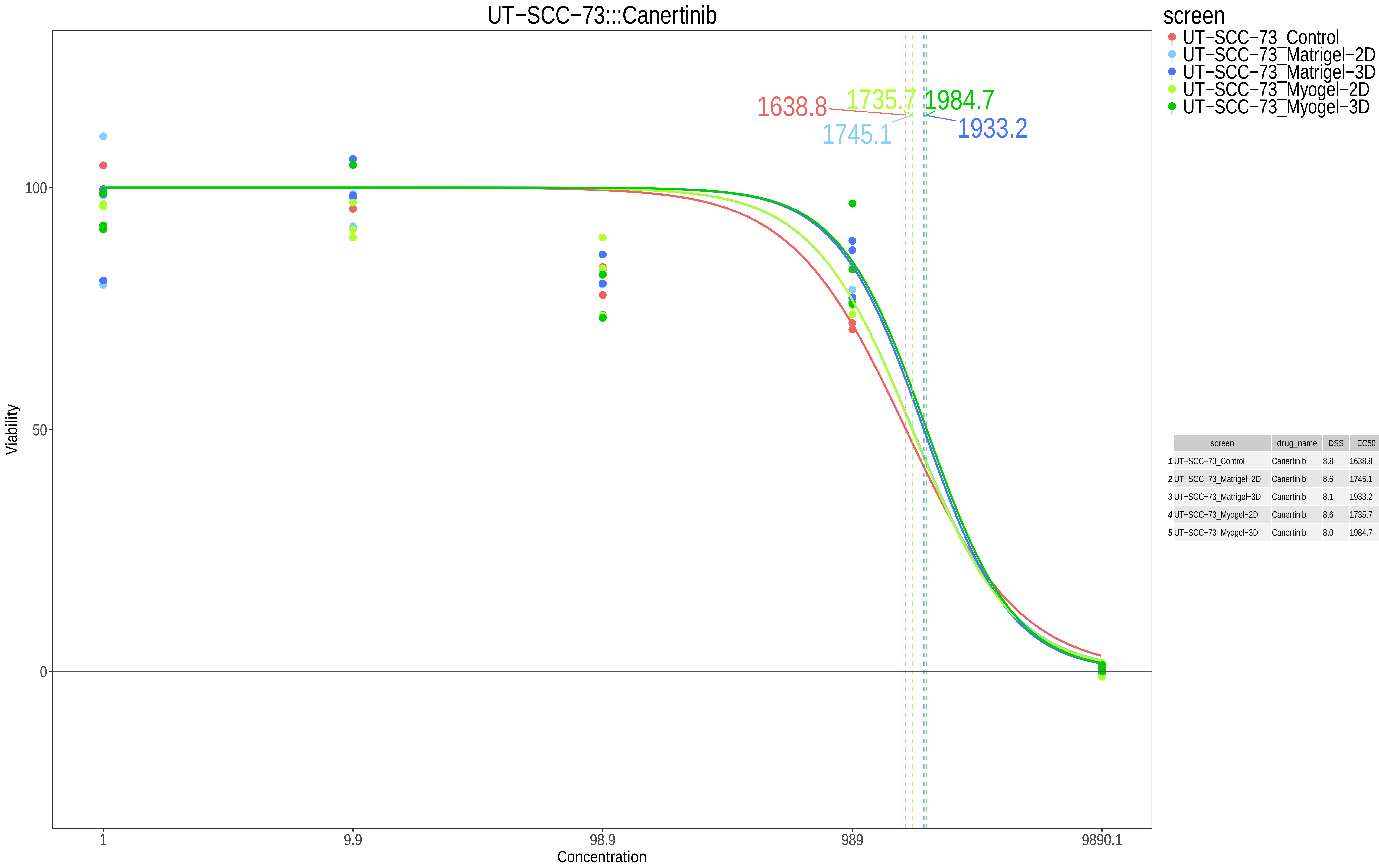

	screen	drug_name	DSS	EC50
1	UT−SCC−73_Control	Canertinib	8.8	1638.8
2	UT−SCC−73_Matrigel−2D	Canertinib	8.6	1745.1
3	UT−SCC−73_Matrigel−3D	Canertinib	8.1	1933.2
4	UT−SCC−73_Myogel−2D	Canertinib	8.6	1735.7
5	UT−SCC−73_Myogel−3D	Canertinib	8.0	1984.7

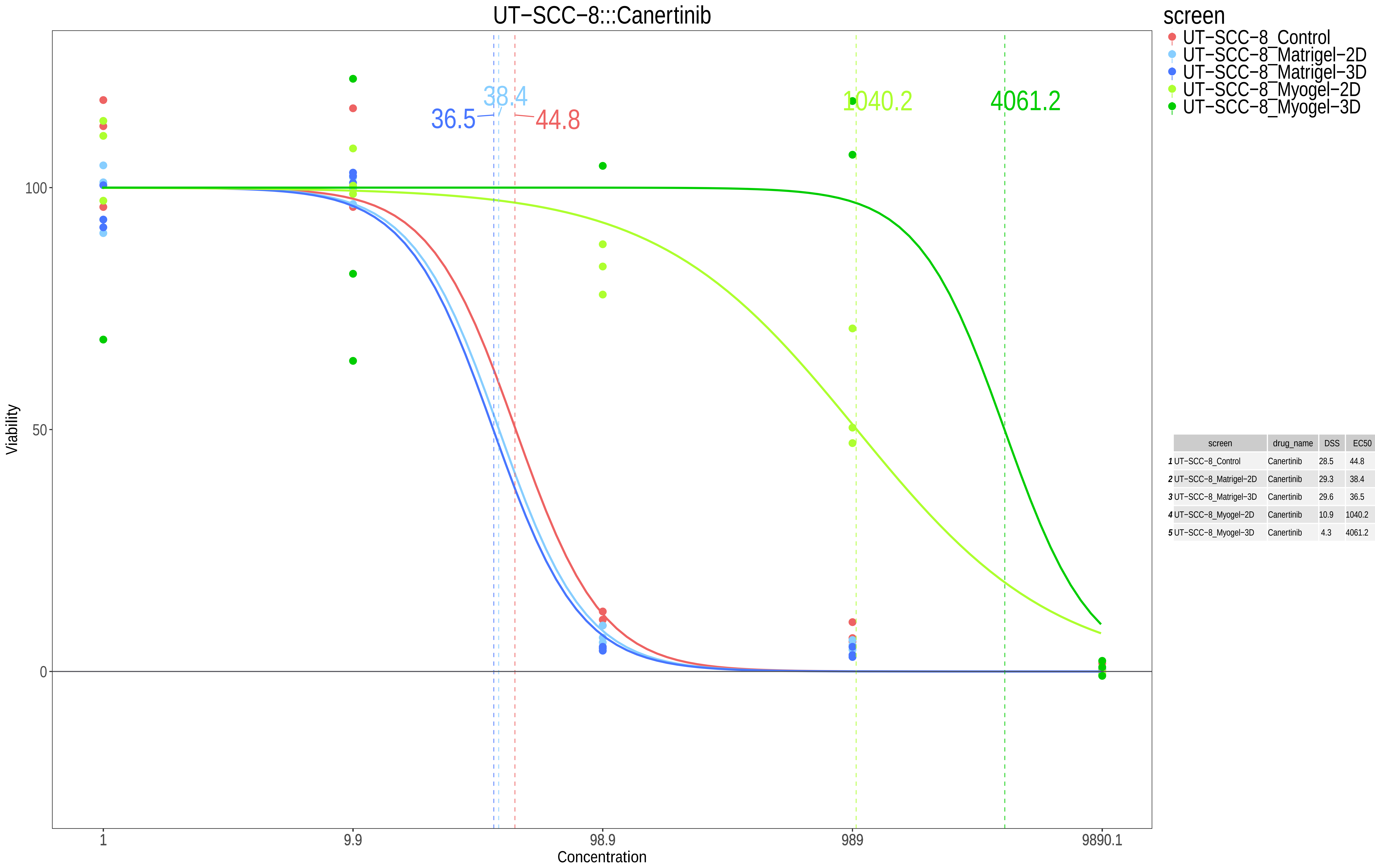

	screen	drug_name	DSS	EC50
1	UT−SCC−8_Control	Canertinib	28.5	44.8
2	UT−SCC−8_Matrigel−2D	Canertinib	29.3	38.4
3	UT−SCC−8_Matrigel−3D	Canertinib	29.6	36.5
4	UT−SCC−8_Myogel−2D	Canertinib	10.9	1040.2
5	UT−SCC−8_Myogel−3D	Canertinib	4.3	4061.2

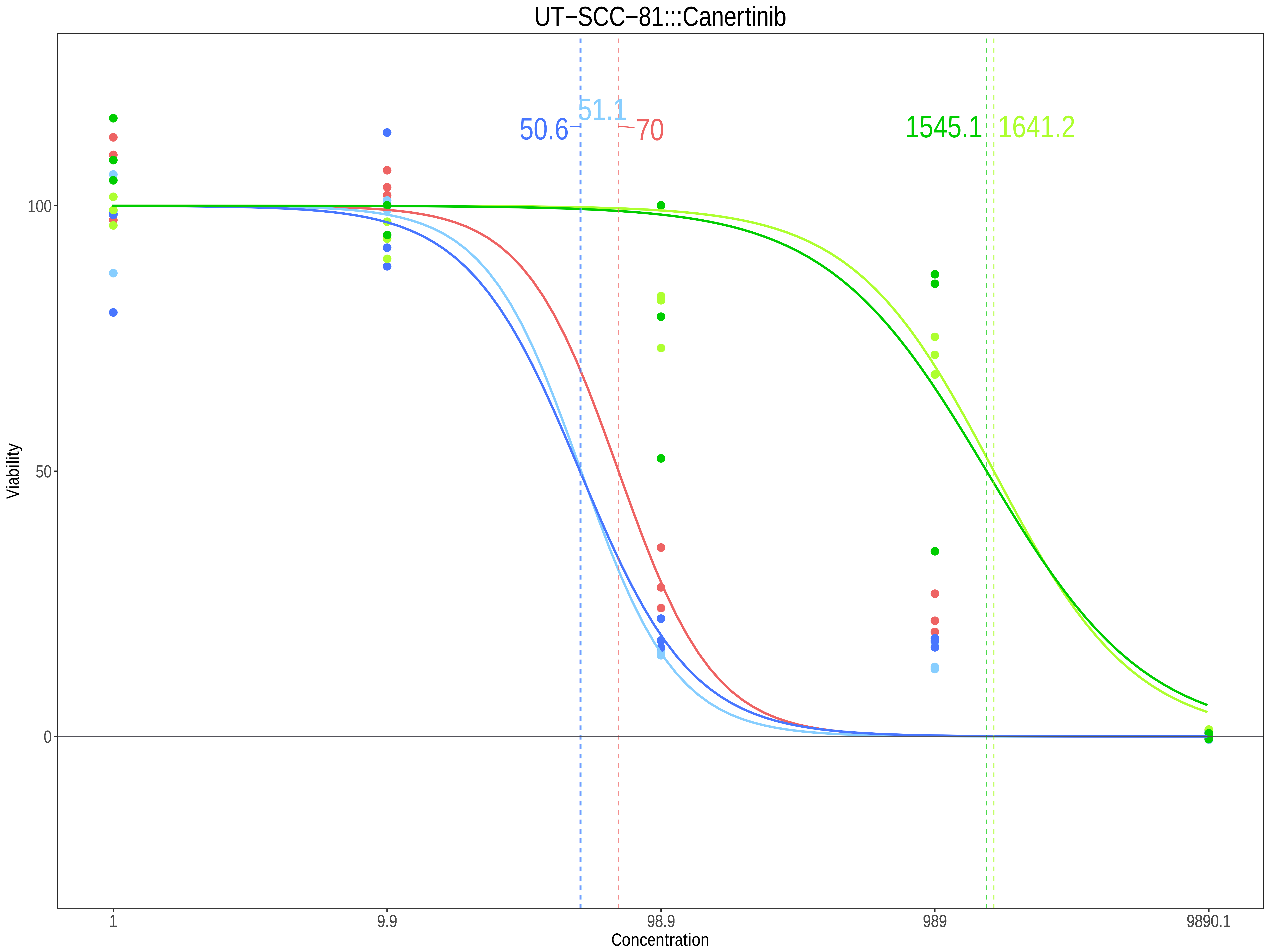

	screen	drug_name	DSS	EC50
1	UT−SCC−81_Control	Canertinib	26.1	70.0
2	UT−SCC−81_Matrigel−2D	Canertinib	27.8	51.1
3	UT−SCC−81_Matrigel−3D	Canertinib	27.7	50.6
4	UT−SCC−81_Myogel−2D	Canertinib	8.8	1641.2
5	UT−SCC−81_Myogel−3D	Canertinib	9.0	1545.1

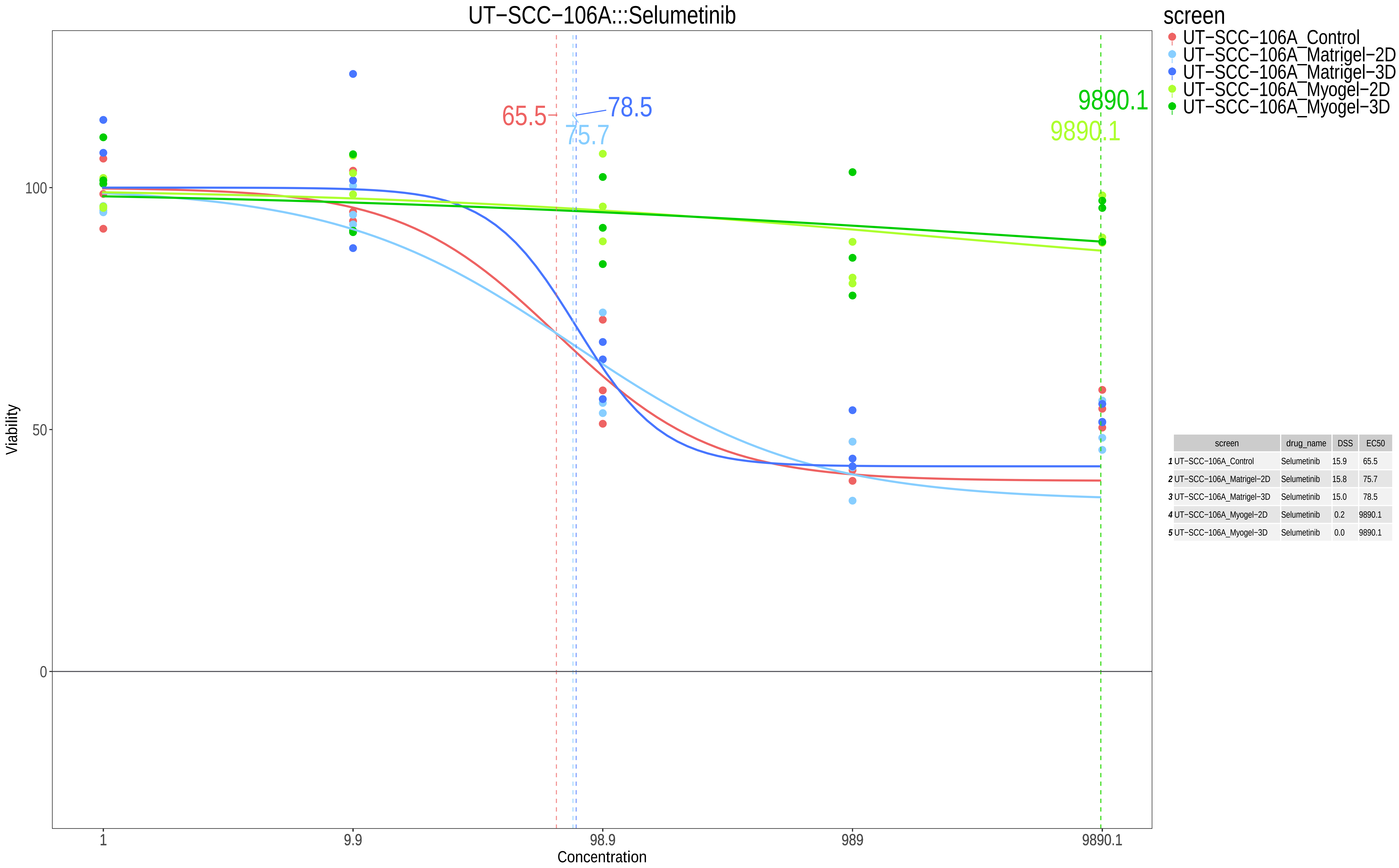

	screen	drug_name	DSS	EC50
1	UT−SCC−106A_Control	Selumetinib	15.9	65.5
2	UT−SCC−106A_Matrigel−2D	Selumetinib	15.8	75.7
3	UT−SCC−106A_Matrigel−3D	Selumetinib	15.0	78.5
4	UT−SCC−106A_Myogel−2D	Selumetinib	0.2	9890.1
5	UT−SCC−106A_Myogel−3D	Selumetinib	0.0	9890.1

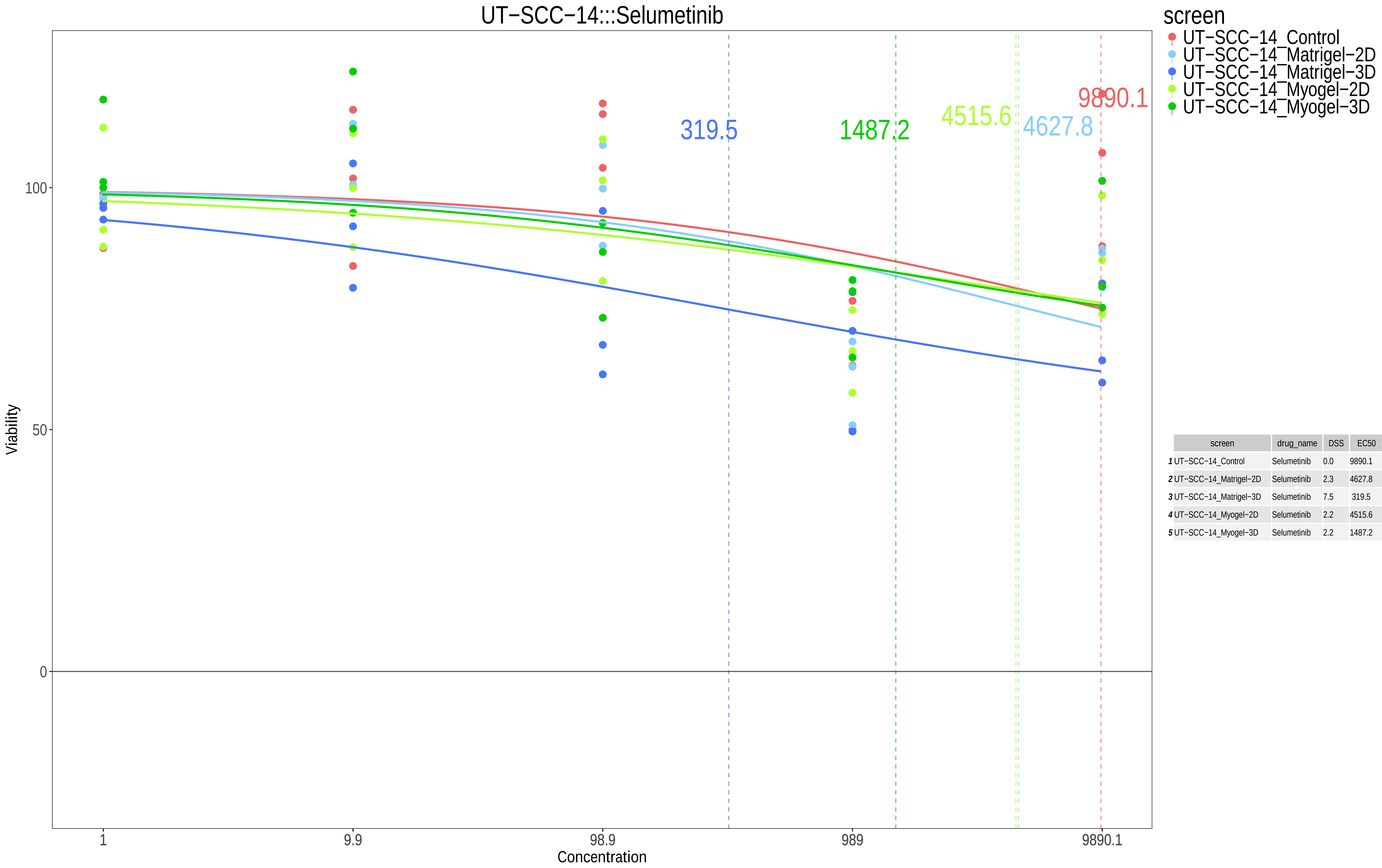

	screen	drug_name	DSS	EC50
1	UT−SCC−14_Control	Selumetinib	0.0	9890.1
2	UT−SCC−14_Matrigel−2D	Selumetinib	2.3	4627.8
3	UT−SCC−14_Matrigel−3D	Selumetinib	7.5	319.5
4	UT−SCC−14_Myogel−2D	Selumetinib	2.2	4515.6
5	UT−SCC−14_Myogel−3D	Selumetinib	2.2	1487.2

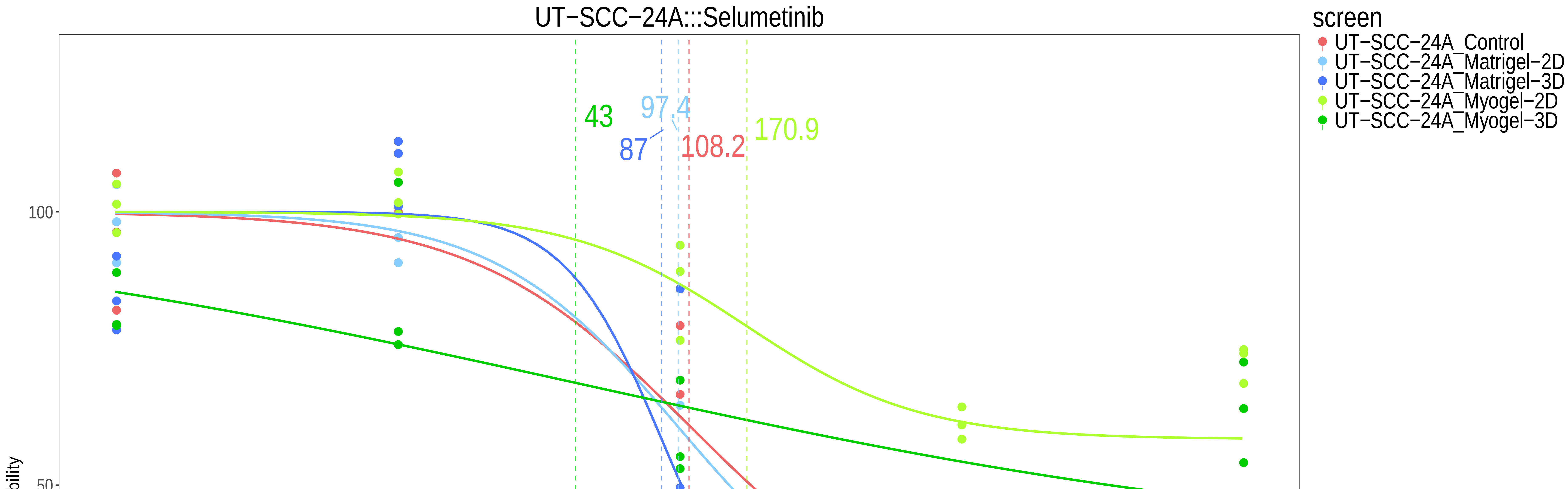

	screen	drug_name	DSS	EC50
1	UT−SCC−24A_Control	Selumetinib	18.0	108.2
2	UT−SCC−24A_Matrigel−2D	Selumetinib	18.7	97.4
3	UT−SCC−24A_Matrigel−3D	Selumetinib	21.3	87.0
4	UT−SCC−24A_Myogel−2D	Selumetinib	8.4	170.9
5	UT−SCC−24A_Myogel−3D	Selumetinib	15.4	43.0

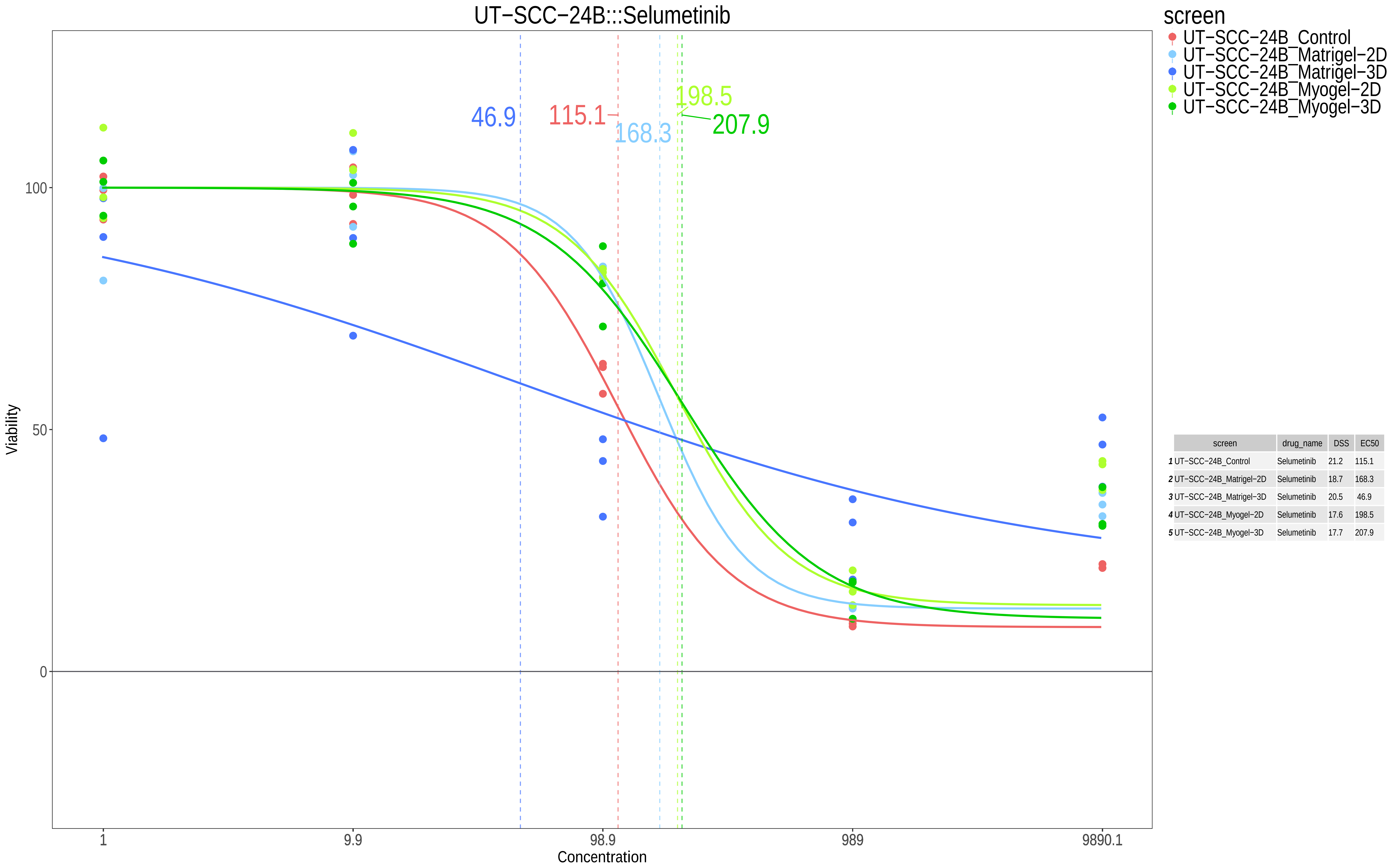

	screen	drug_name	DSS	EC50
1	UT−SCC−24B_Control	Selumetinib	21.2	115.1
2	UT−SCC−24B_Matrigel−2D	Selumetinib	18.7	168.3
3	UT−SCC−24B_Matrigel−3D	Selumetinib	20.5	46.9
4	UT−SCC−24B_Myogel−2D	Selumetinib	17.6	198.5
5	UT−SCC−24B_Myogel−3D	Selumetinib	17.7	207.9

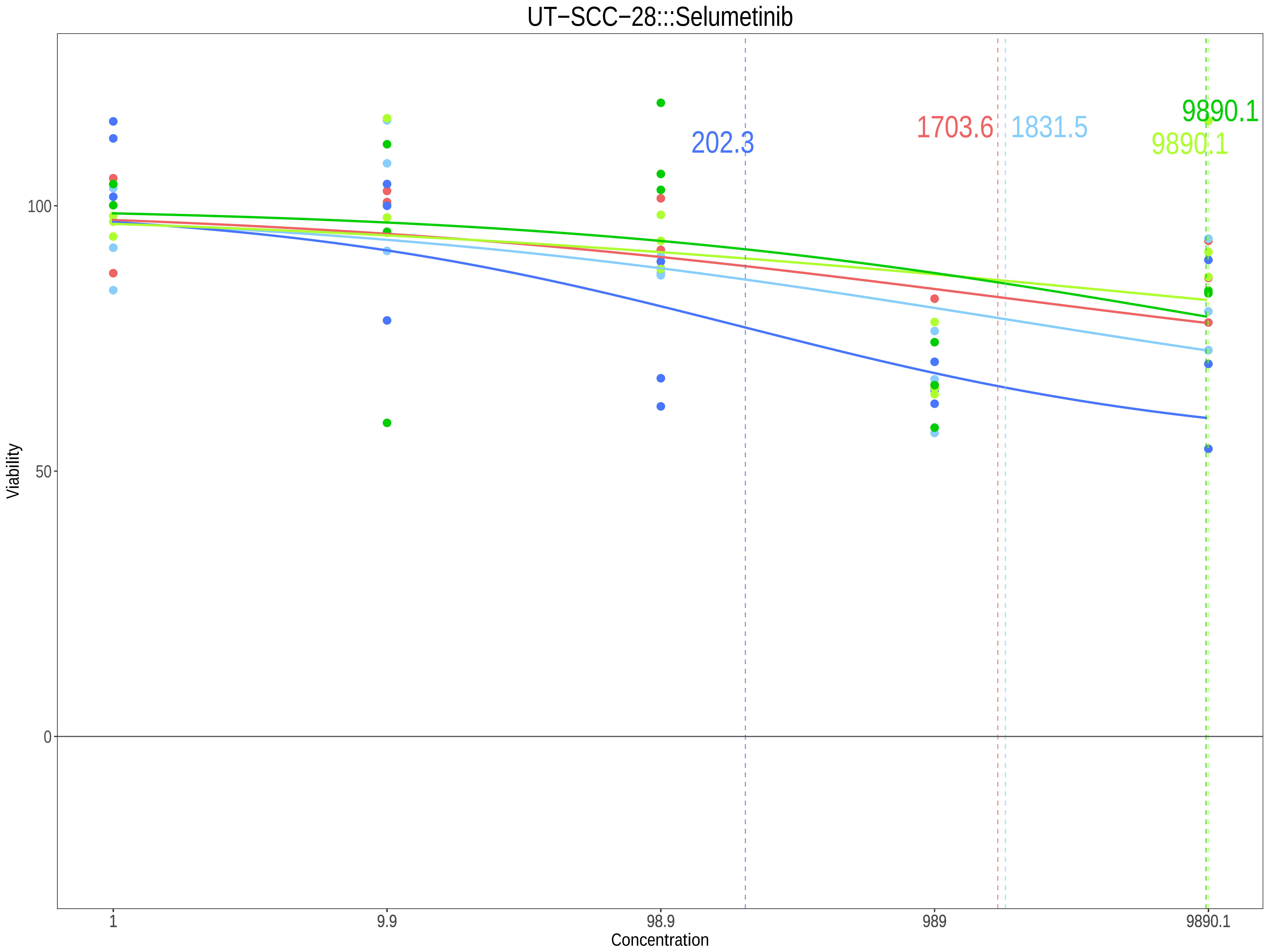

	screen	drug_name	DSS	EC50
1	UT−SCC−28_Control	Selumetinib	2.1	1703.6
2	UT−SCC−28_Matrigel−2D	Selumetinib	3.2	1831.5
3	UT−SCC−28_Matrigel−3D	Selumetinib	7.5	202.3
4	UT−SCC−28_Myogel−2D	Selumetinib	0.0	9890.1
5	UT−SCC−28_Myogel−3D	Selumetinib	0.0	9890.1

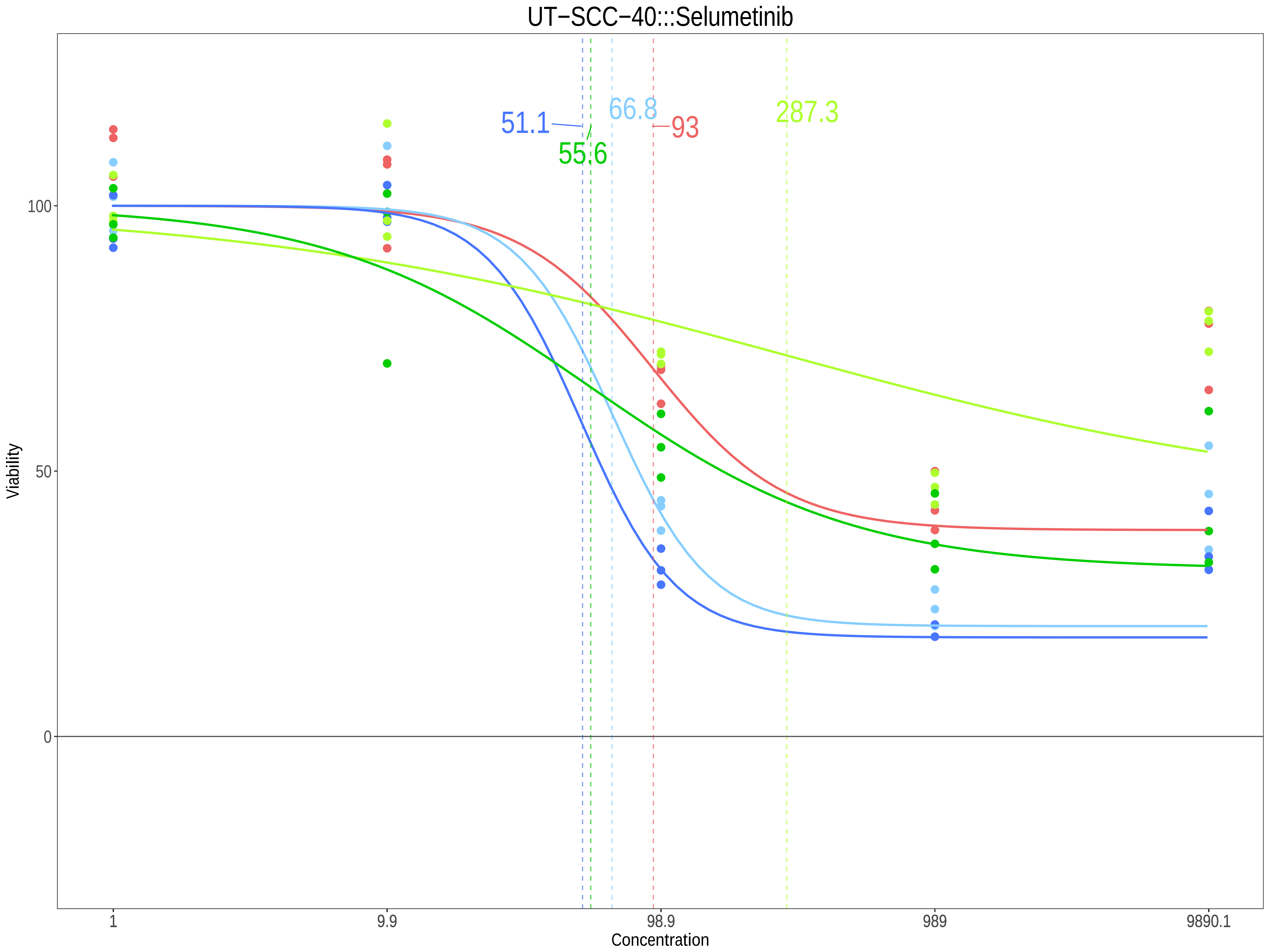

	screen	drug_name	DSS	EC50
1	UT−SCC−40_Control	Selumetinib	15.1	93.0
2	UT−SCC−40_Matrigel−2D	Selumetinib	21.2	66.8
3	UT−SCC−40_Matrigel−3D	Selumetinib	23.0	51.1
4	UT−SCC−40_Myogel−2D	Selumetinib	8.9	287.3
5	UT−SCC−40_Myogel−3D	Selumetinib	17.9	55.6

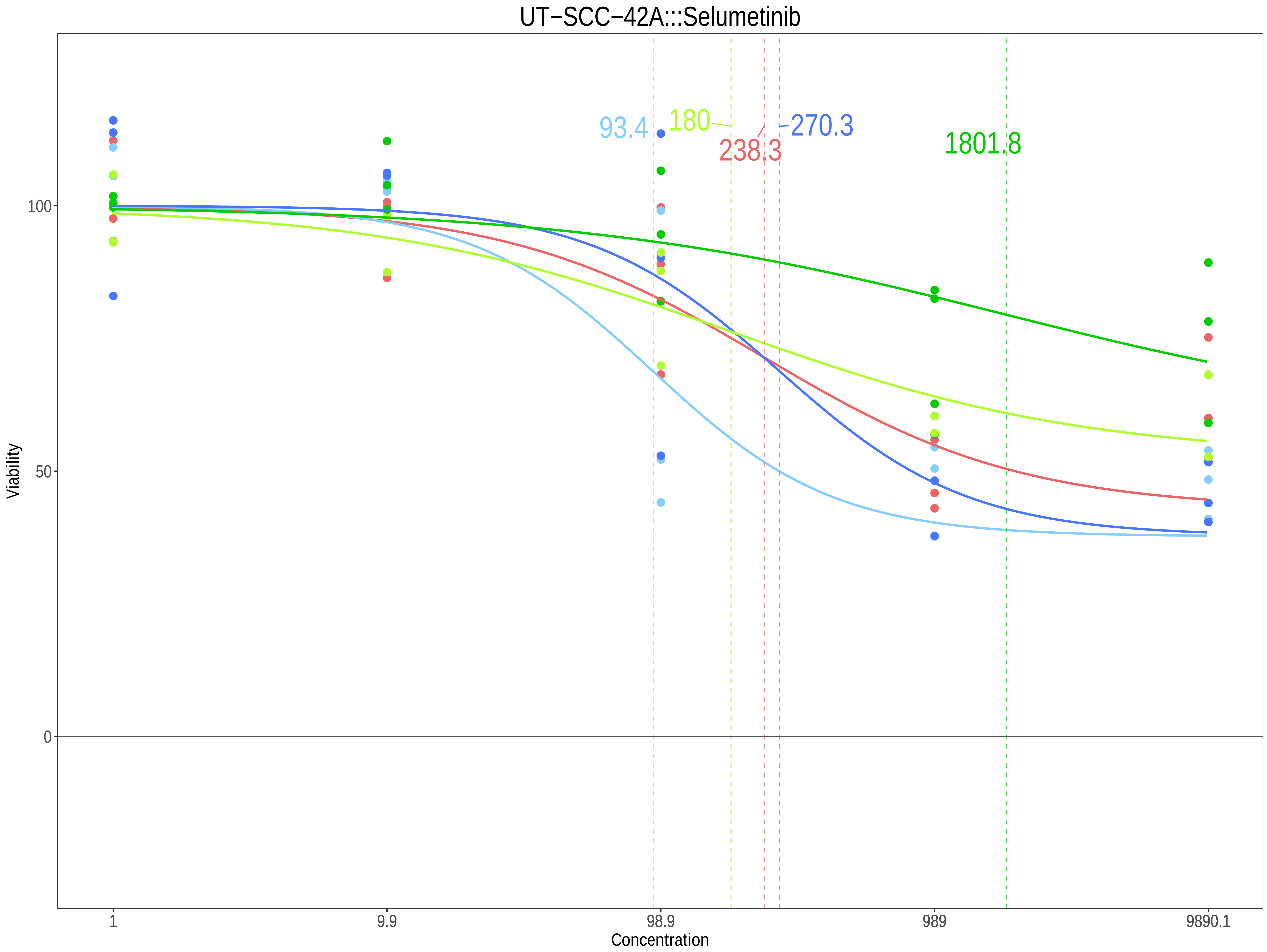

	screen	drug_name	DSS	EC50
1	UT−SCC−42A_Control	Selumetinib	10.2	238.3
2	UT−SCC−42A_Matrigel−2D	Selumetinib	15.0	93.4
3	UT−SCC−42A_Matrigel−3D	Selumetinib	11.2	270.3
4	UT−SCC−42A_Myogel−2D	Selumetinib	8.5	180.0
5	UT−SCC−42A_Myogel−3D	Selumetinib	2.7	1801.8

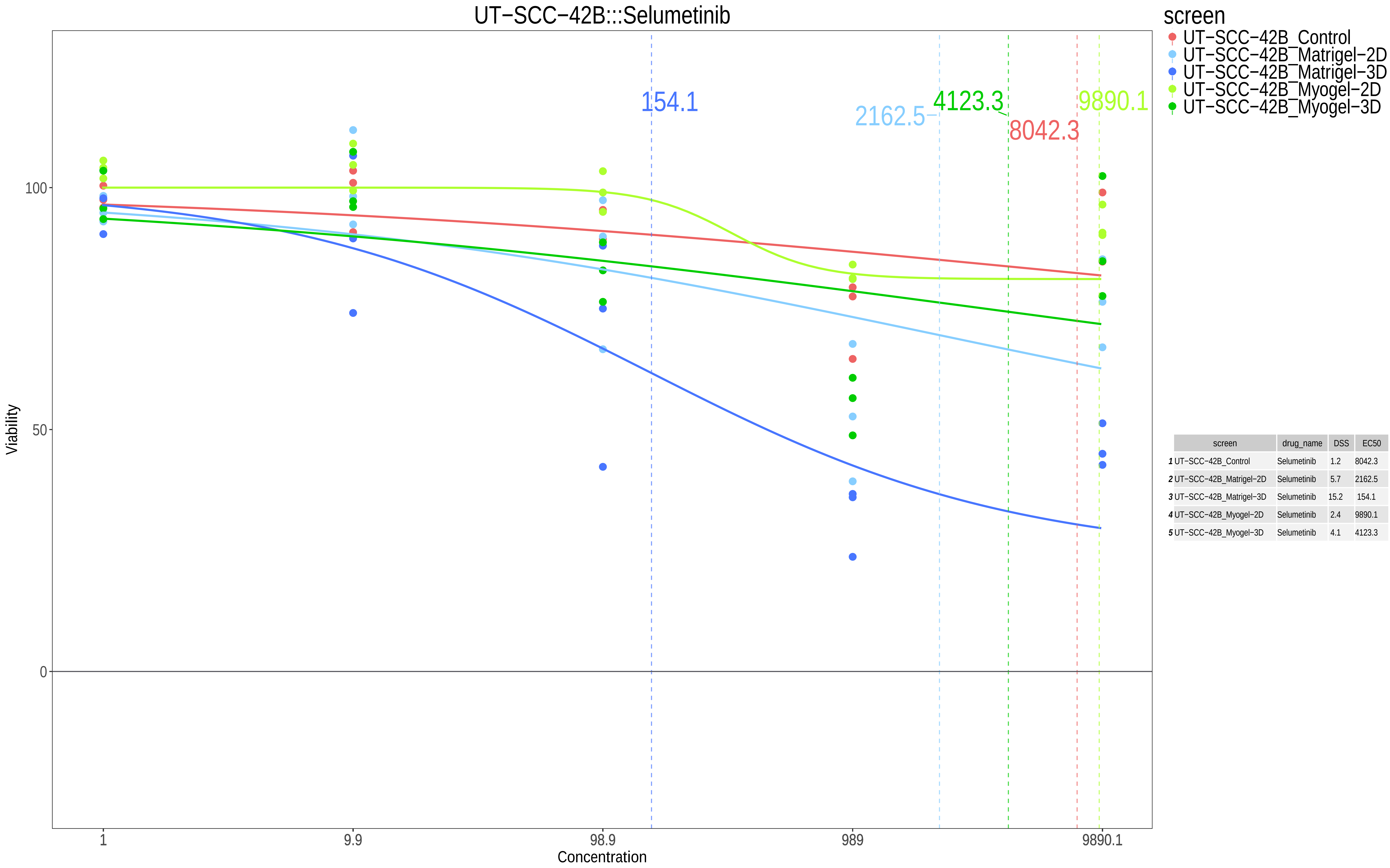

	screen	drug_name	DSS	EC50
1	UT−SCC−42B_Control	Selumetinib	1.2	8042.3
2	UT−SCC−42B_Matrigel−2D	Selumetinib	5.7	2162.5
3	UT−SCC−42B_Matrigel−3D	Selumetinib	15.2	154.1
4	UT−SCC−42B_Myogel−2D	Selumetinib	2.4	9890.1
5	UT−SCC−42B_Myogel−3D	Selumetinib	4.1	4123.3

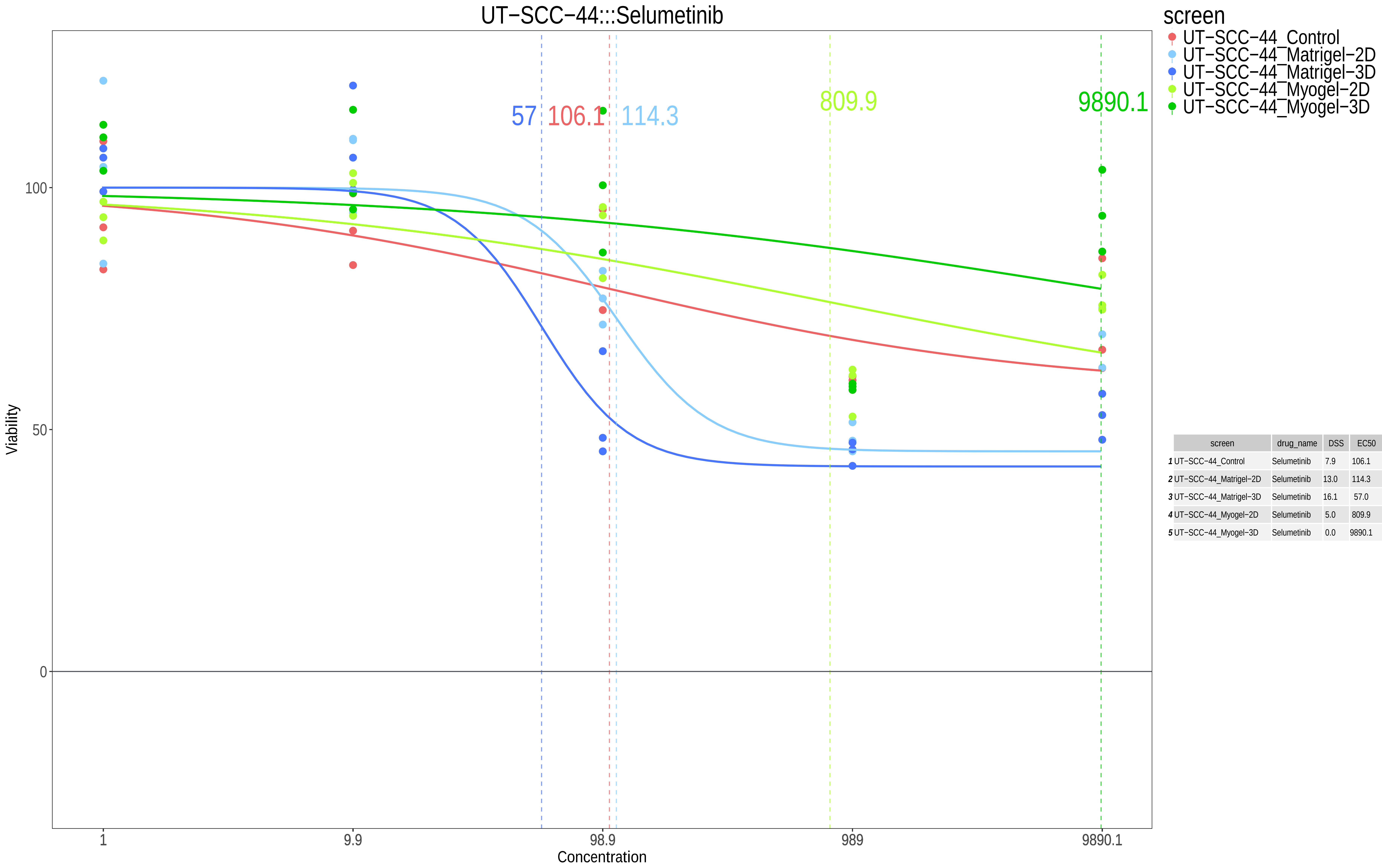

	screen	drug_name	DSS	EC50
1	UT−SCC−44_Control	Selumetinib	7.9	106.1
2	UT−SCC−44_Matrigel−2D	Selumetinib	13.0	114.3
3	UT−SCC−44_Matrigel−3D	Selumetinib	16.1	57.0
4	UT−SCC−44_Myogel−2D	Selumetinib	5.0	809.9
5	UT−SCC−44_Myogel−3D	Selumetinib	0.0	9890.1

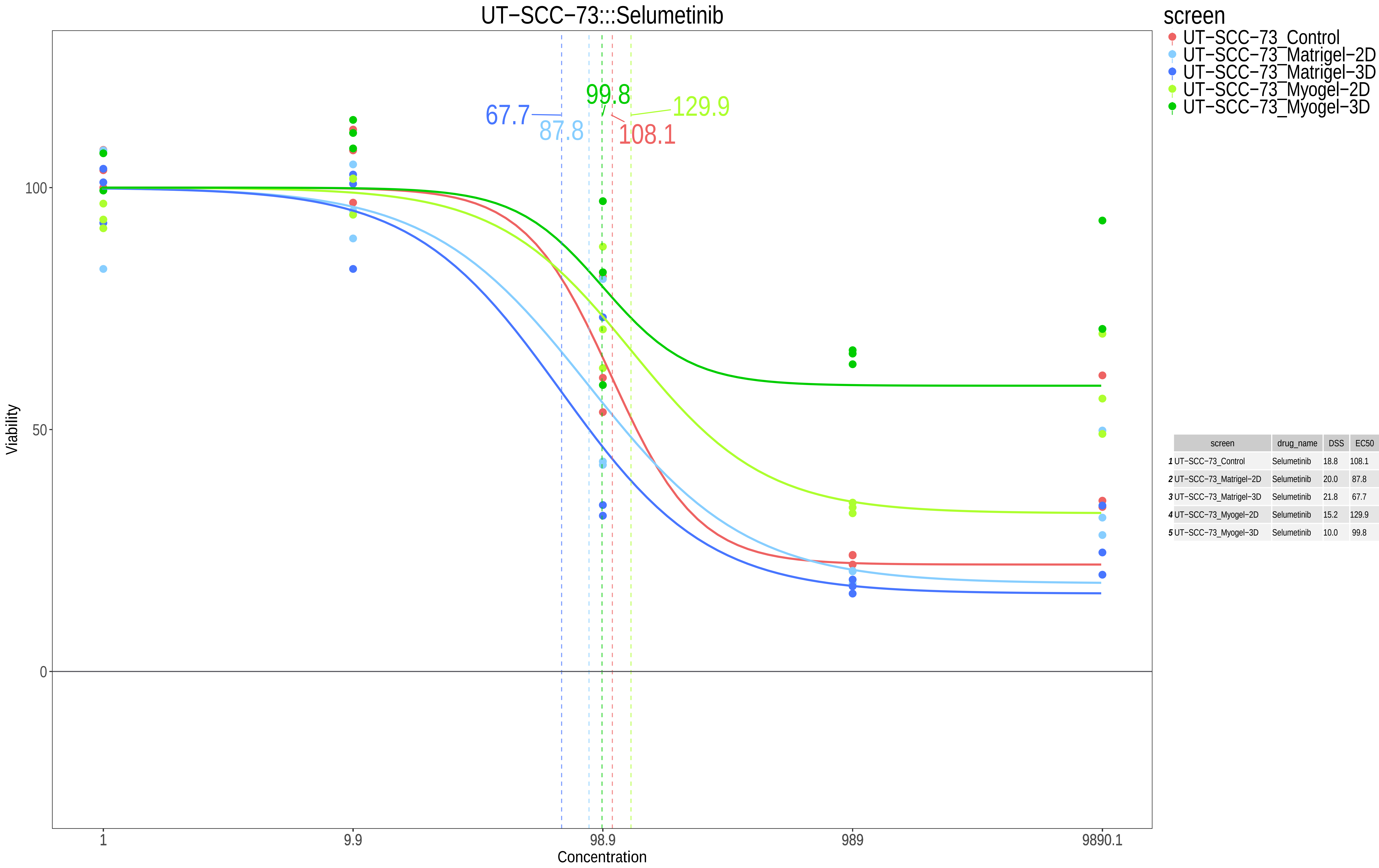

	screen	drug_name	DSS	EC50
1	UT−SCC−73_Control	Selumetinib	18.8	108.1
2	UT−SCC−73_Matrigel−2D	Selumetinib	20.0	87.8
3	UT−SCC−73_Matrigel−3D	Selumetinib	21.8	67.7
4	UT−SCC−73_Myogel−2D	Selumetinib	15.2	129.9
5	UT−SCC−73_Myogel−3D	Selumetinib	10.0	99.8

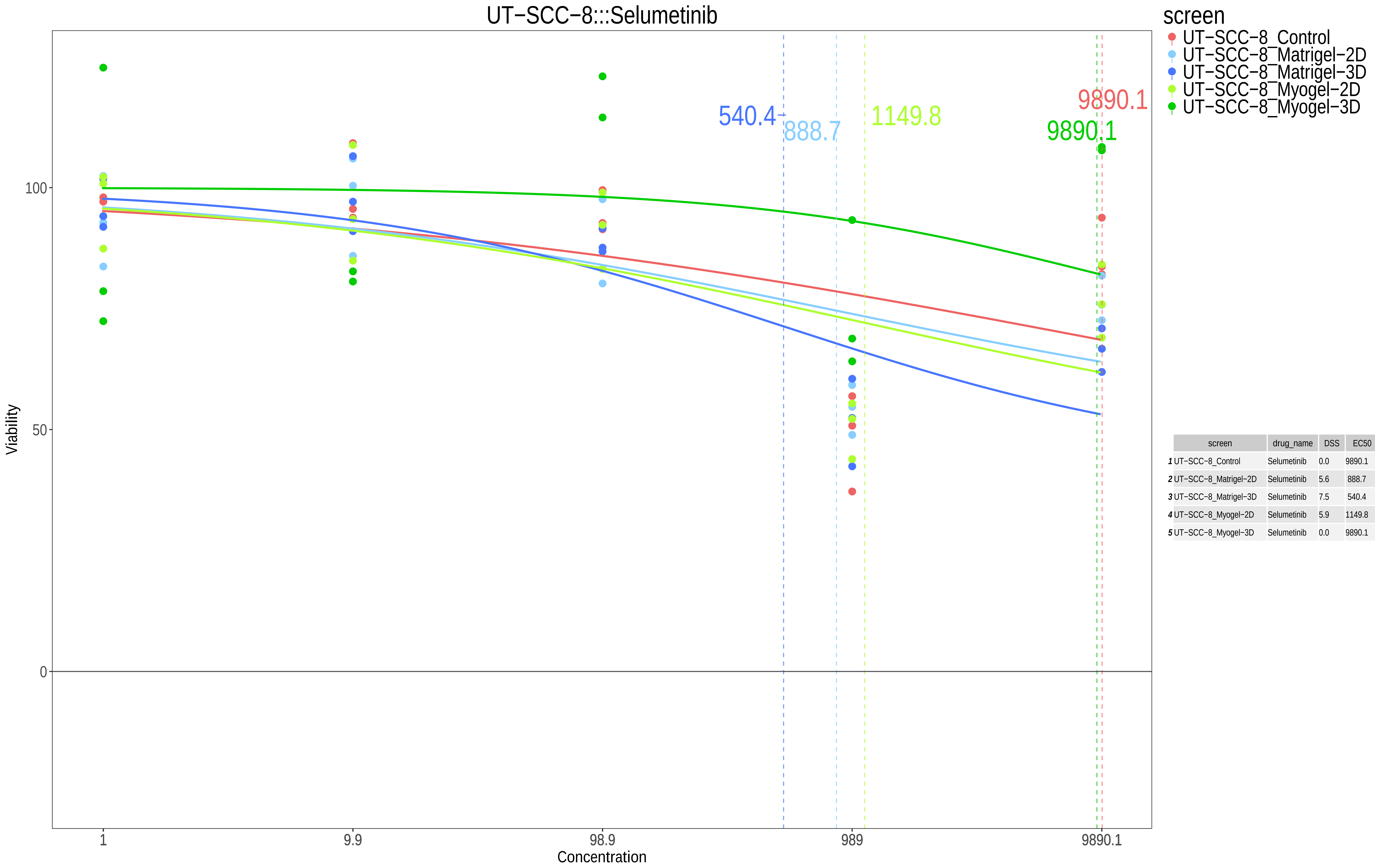

	screen	drug_name	DSS	EC50
1	UT−SCC−8_Control	Selumetinib	0.0	9890.1
2	UT−SCC−8_Matrigel−2D	Selumetinib	5.6	888.7
3	UT−SCC−8_Matrigel−3D	Selumetinib	7.5	540.4
4	UT−SCC−8_Myogel−2D	Selumetinib	5.9	1149.8
5	UT−SCC−8_Myogel−3D	Selumetinib	0.0	9890.1

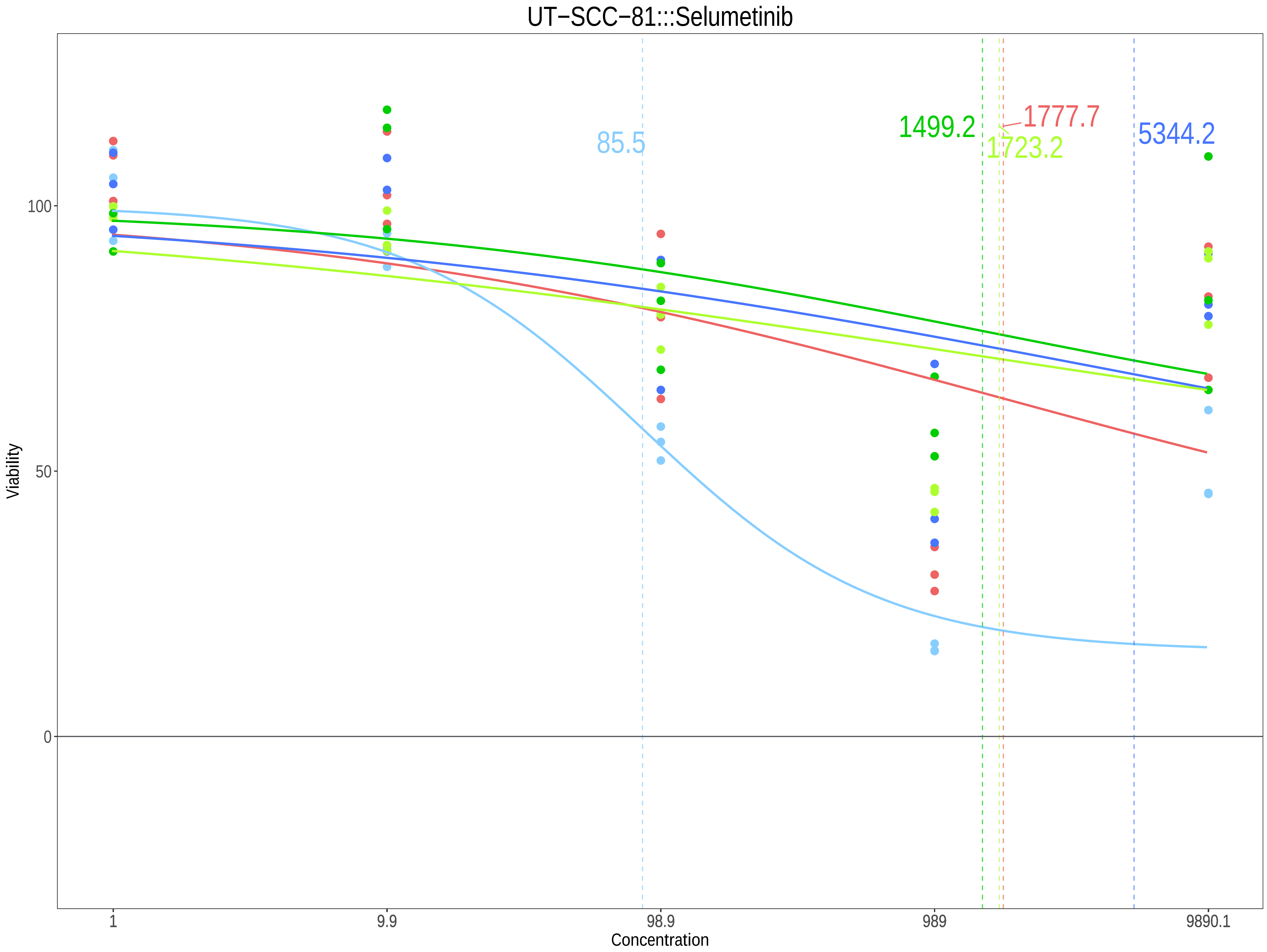

	screen	drug_name	DSS	EC50
1	UT−SCC−81_Control	Selumetinib	7.6	1777.7
2	UT−SCC−81_Matrigel−2D	Selumetinib	20.1	85.5
3	UT−SCC−81_Matrigel−3D	Selumetinib	5.0	5344.2
4	UT−SCC−81_Myogel−2D	Selumetinib	6.5	1723.2
5	UT−SCC−81_Myogel−3D	Selumetinib	4.0	1499.2

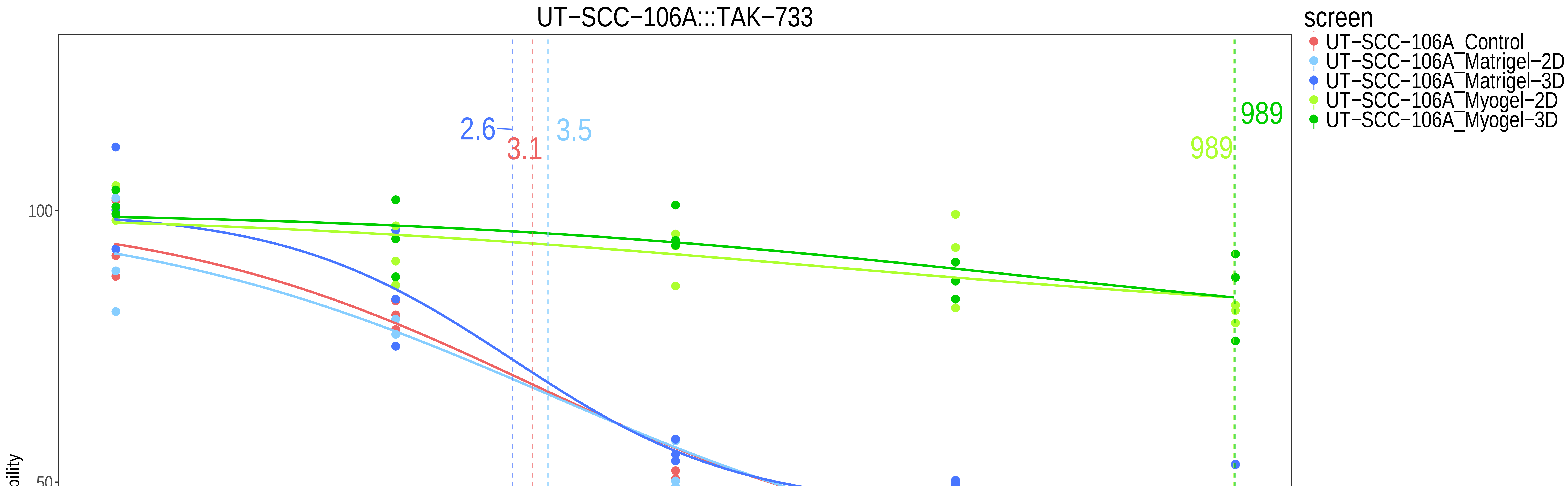

	screen	drug_name	DSS	EC50
1	UT−SCC−106A_Control	TAK−733	18.1	3.1
2	UT−SCC−106A_Matrigel−2D	TAK−733	18.5	3.5
3	UT−SCC−106A_Matrigel−3D	TAK−733	16.8	2.6
4	UT−SCC−106A_Myogel−2D	TAK−733	1.0	989.0
5	UT−SCC−106A_Myogel−3D	TAK−733	0.7	989.0

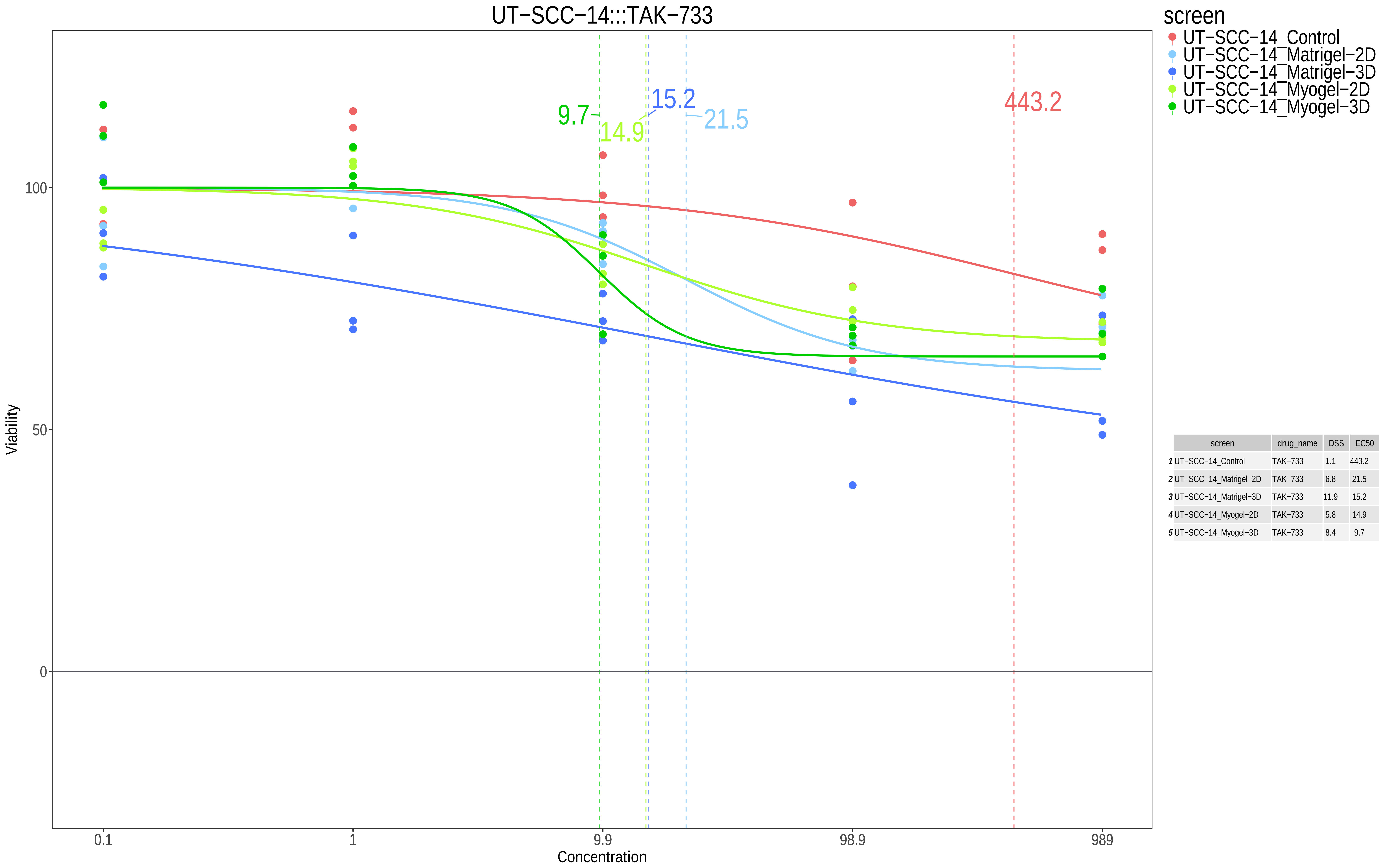

	screen	drug_name	DSS	EC50
1	UT−SCC−14_Control	TAK−733	1.1	443.2
2	UT−SCC−14_Matrigel−2D	TAK−733	6.8	21.5
3	UT−SCC−14_Matrigel−3D	TAK−733	11.9	15.2
4	UT−SCC−14_Myogel−2D	TAK−733	5.8	14.9
5	UT−SCC−14_Myogel−3D	TAK−733	8.4	9.7

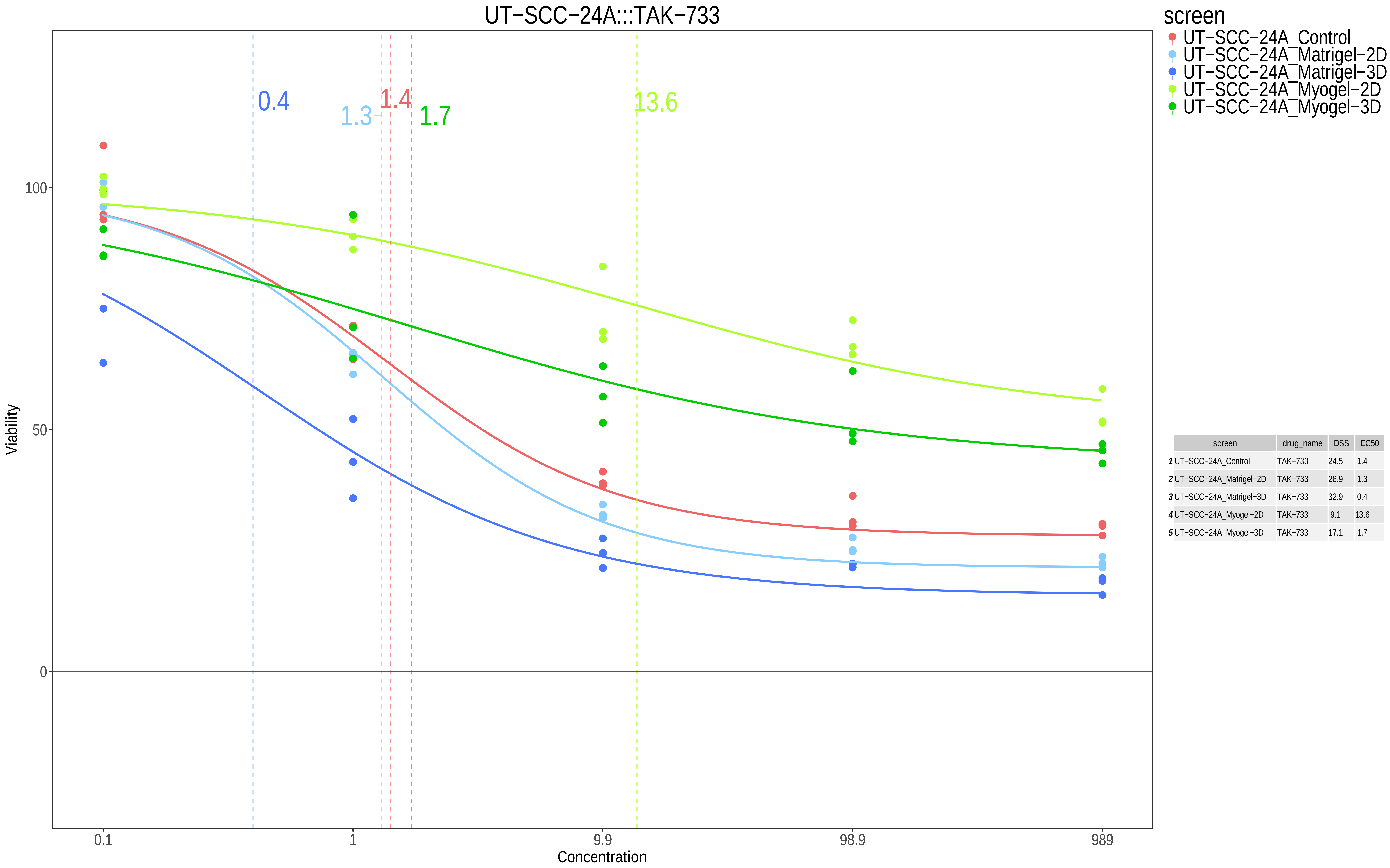

	screen	drug_name	DSS	EC50
1	UT−SCC−24A_Control	TAK−733	24.5	1.4
2	UT−SCC−24A_Matrigel−2D	TAK−733	26.9	1.3
3	UT−SCC−24A_Matrigel−3D	TAK−733	32.9	0.4
4	UT−SCC−24A_Myogel−2D	TAK−733	9.1	13.6
5	UT−SCC−24A_Myogel−3D	TAK−733	17.1	1.7

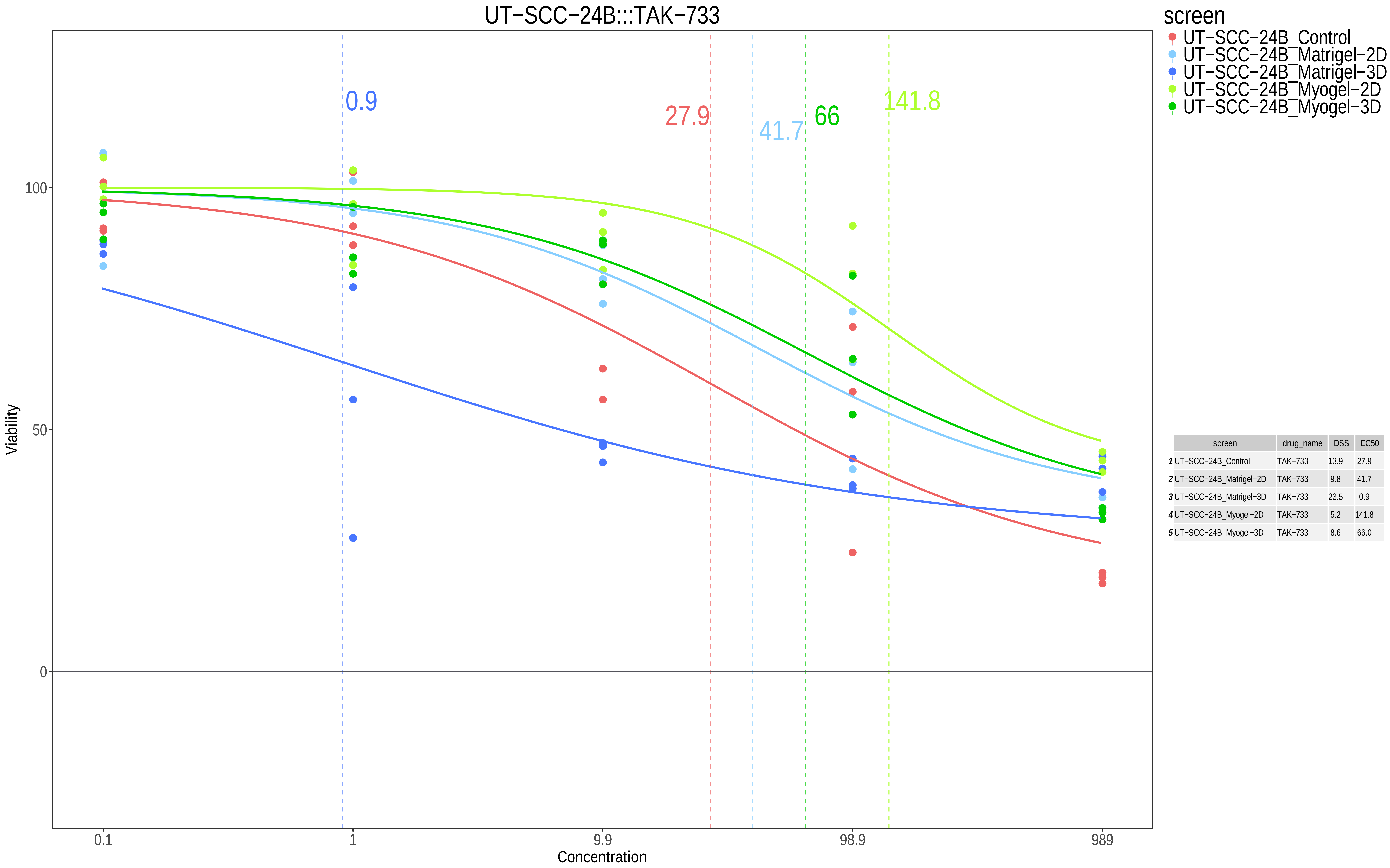

	screen	drug_name	DSS	EC50
1	UT−SCC−24B_Control	TAK−733	13.9	27.9
2	UT−SCC−24B_Matrigel−2D	TAK−733	9.8	41.7
3	UT−SCC−24B_Matrigel−3D	TAK−733	23.5	0.9
4	UT−SCC−24B_Myogel−2D	TAK−733	5.2	141.8
5	UT−SCC−24B_Myogel−3D	TAK−733	8.6	66.0

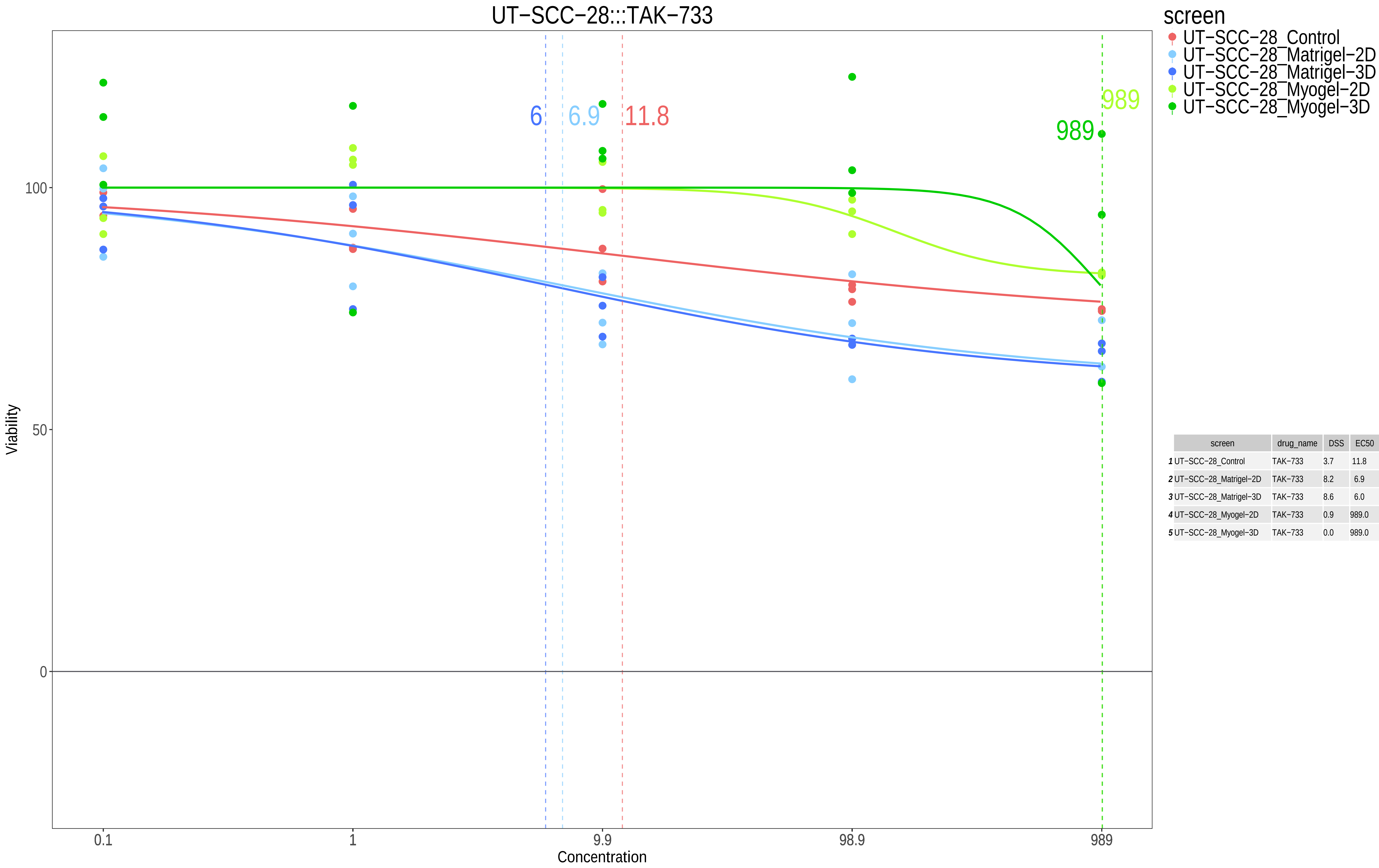

	screen	drug_name	DSS	EC50
1	UT−SCC−28_Control	TAK−733	3.7	11.8
2	UT−SCC−28_Matrigel−2D	TAK−733	8.2	6.9
3	UT−SCC−28_Matrigel−3D	TAK−733	8.6	6.0
4	UT−SCC−28_Myogel−2D	TAK−733	0.9	989.0
5	UT−SCC−28_Myogel−3D	TAK−733	0.0	989.0

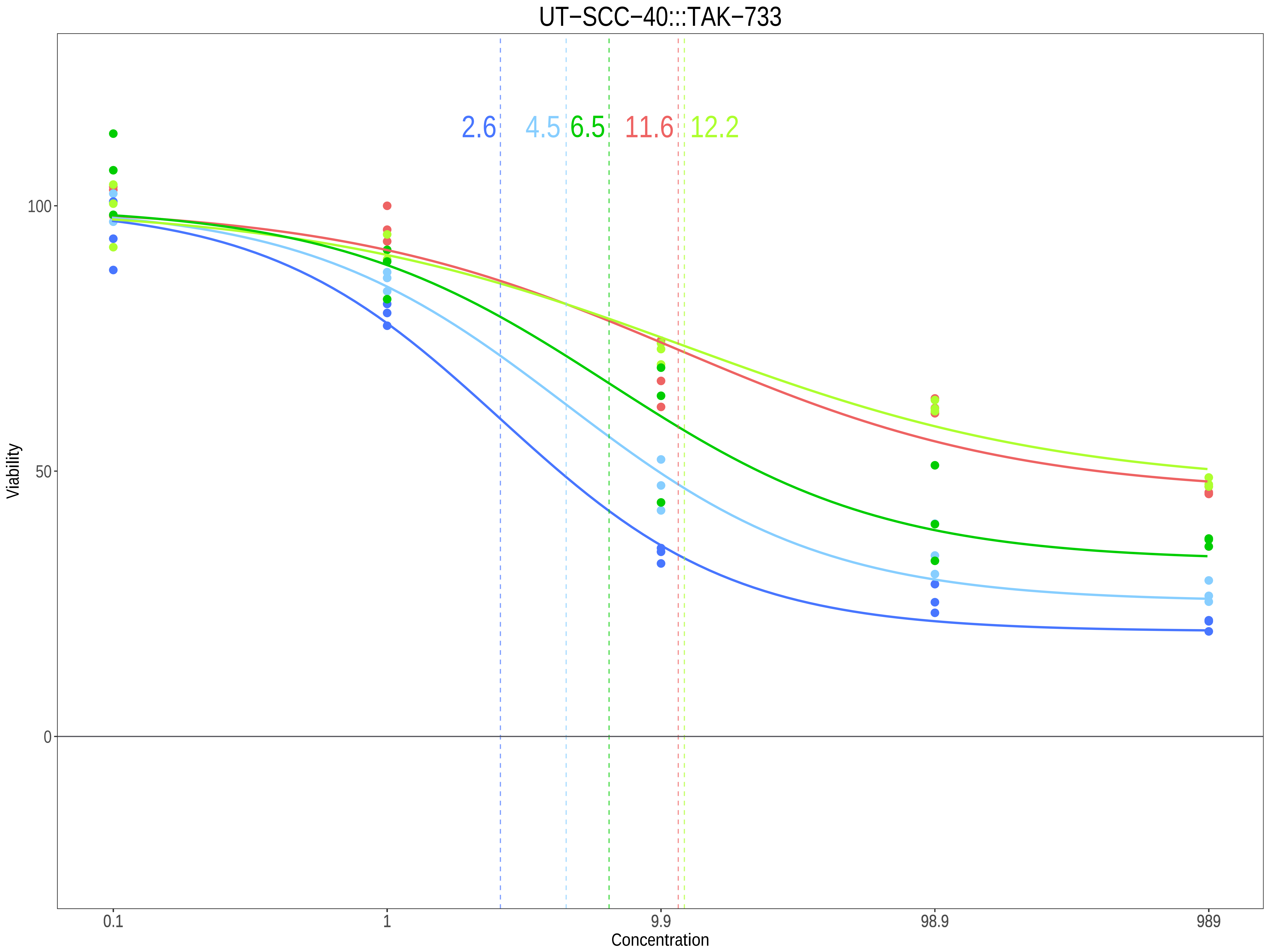

	screen	drug_name	DSS	EC50
1	UT−SCC−40_Control	TAK−733	11.3	11.6
2	UT−SCC−40_Matrigel−2D	TAK−733	20.4	4.5
3	UT−SCC−40_Matrigel−3D	TAK−733	24.6	2.6
4	UT−SCC−40_Myogel−2D	TAK−733	10.7	12.2
5	UT−SCC−40_Myogel−3D	TAK−733	16.8	6.5

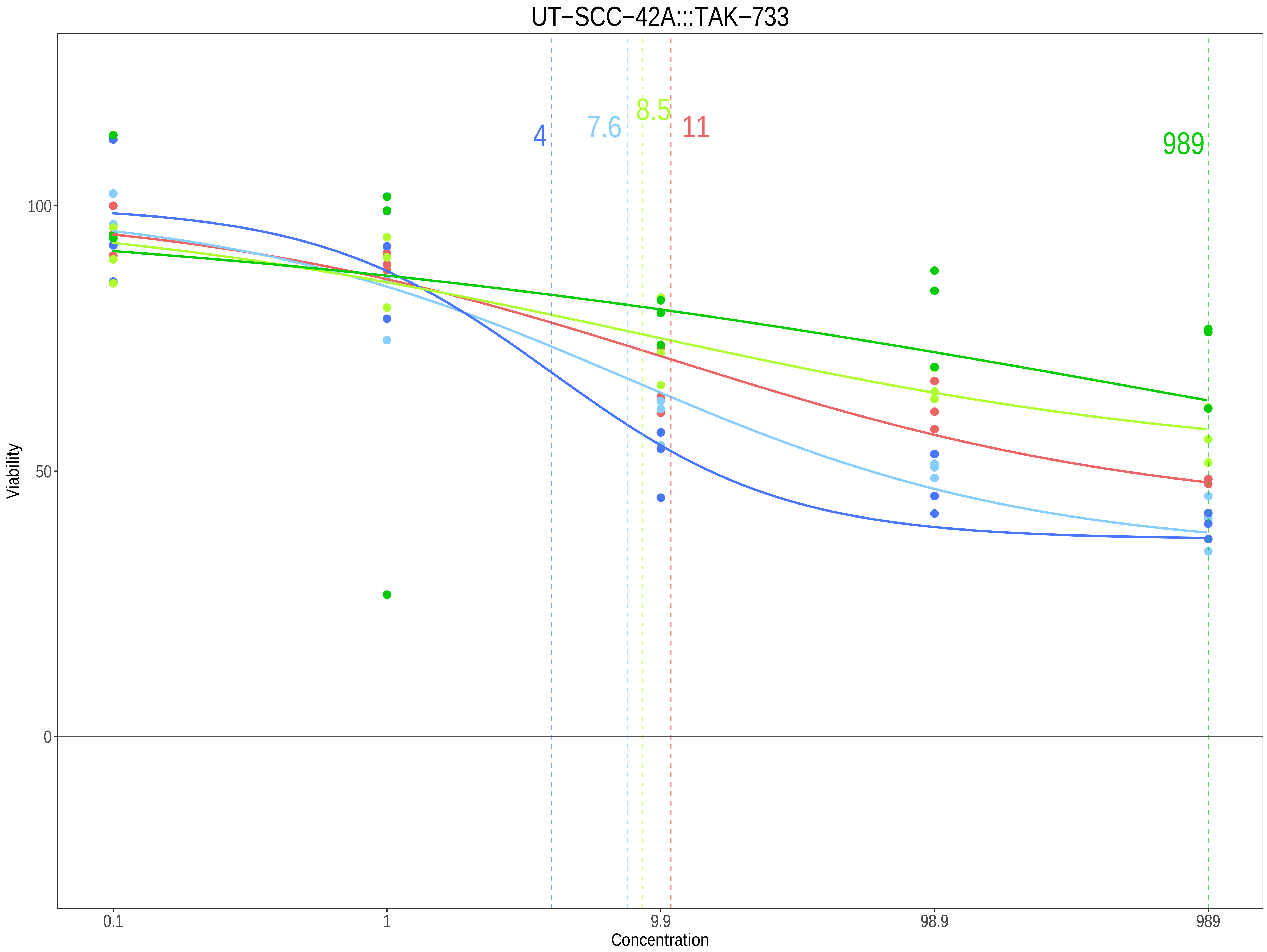

	screen	drug_name	DSS	EC50
1	UT−SCC−42A_Control	TAK−733	11.9	11.0
2	UT−SCC−42A_Matrigel−2D	TAK−733	15.1	7.6
3	UT−SCC−42A_Matrigel−3D	TAK−733	17.7	4.0
4	UT−SCC−42A_Myogel−2D	TAK−733	9.9	8.5
5	UT−SCC−42A_Myogel−3D	TAK−733	0.0	989.0

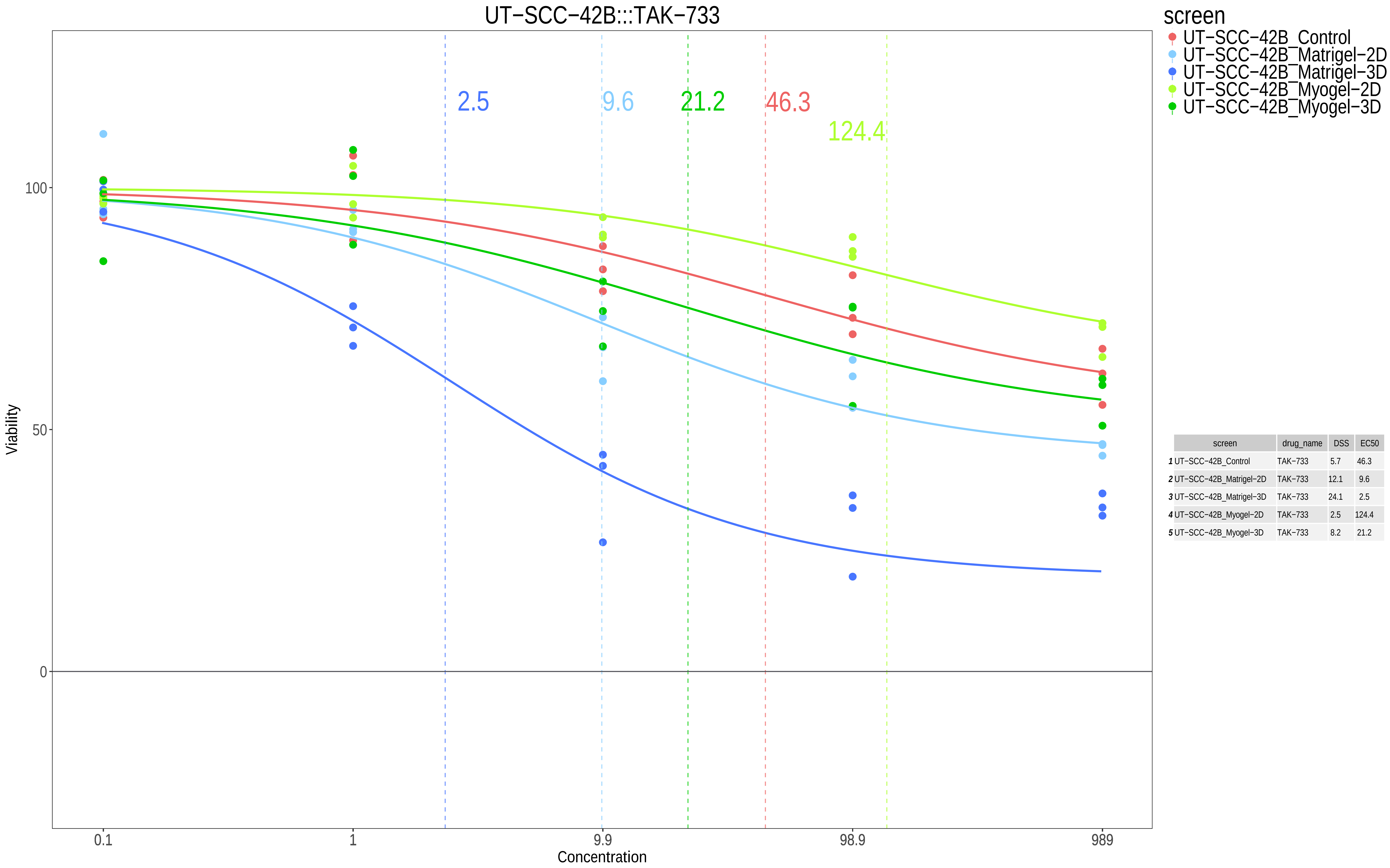

	screen	drug_name	DSS	EC50
1	UT−SCC−42B_Control	TAK−733	5.7	46.3
2	UT−SCC−42B_Matrigel−2D	TAK−733	12.1	9.6
3	UT−SCC−42B_Matrigel−3D	TAK−733	24.1	2.5
4	UT−SCC−42B_Myogel−2D	TAK−733	2.5	124.4
5	UT−SCC−42B_Myogel−3D	TAK−733	8.2	21.2

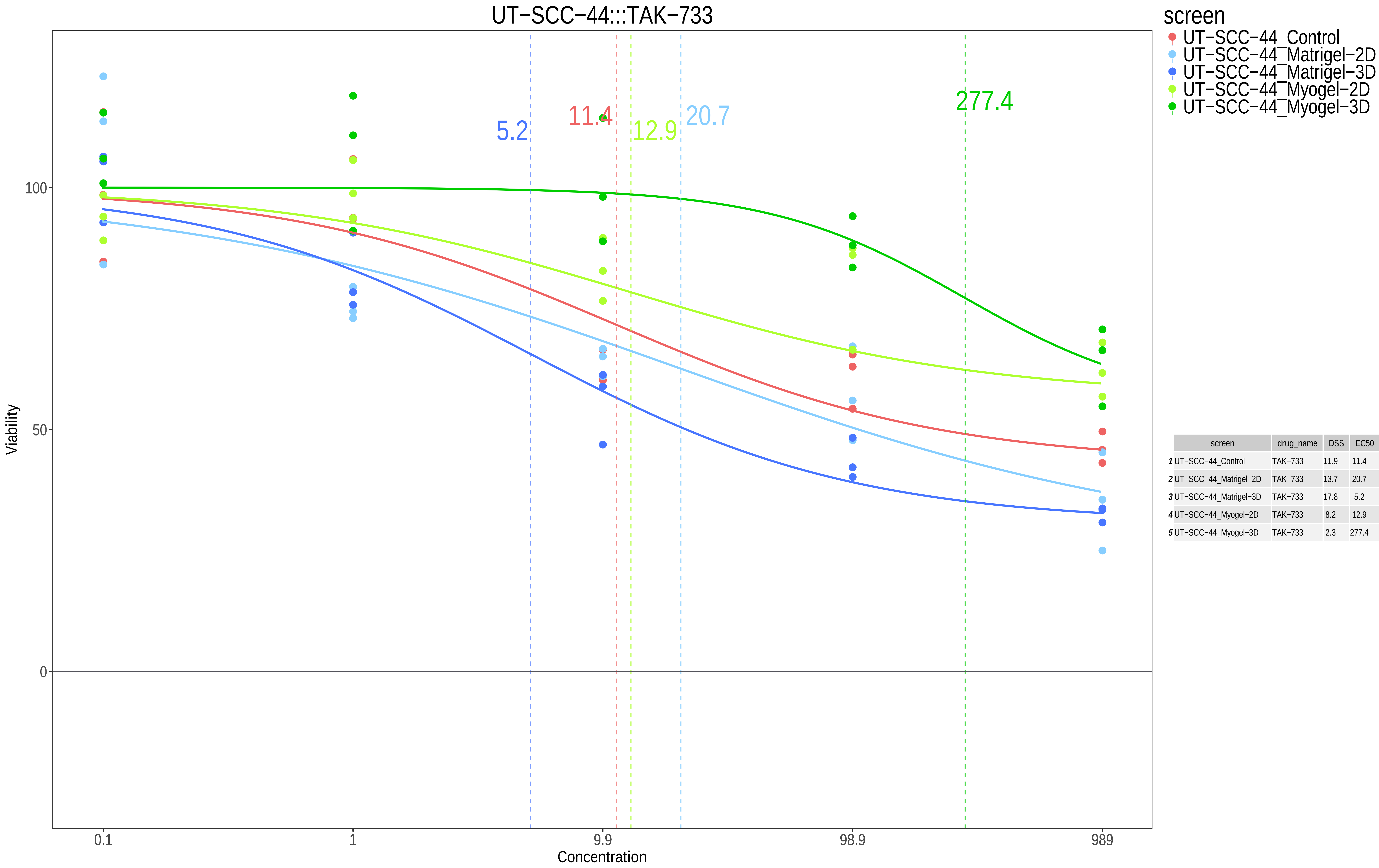

	screen	drug_name	DSS	EC50
1	UT−SCC−44_Control	TAK−733	11.9	11.4
2	UT−SCC−44_Matrigel−2D	TAK−733	13.7	20.7
3	UT−SCC−44_Matrigel−3D	TAK−733	17.8	5.2
4	UT−SCC−44_Myogel−2D	TAK−733	8.2	12.9
5	UT−SCC−44_Myogel−3D	TAK−733	2.3	277.4

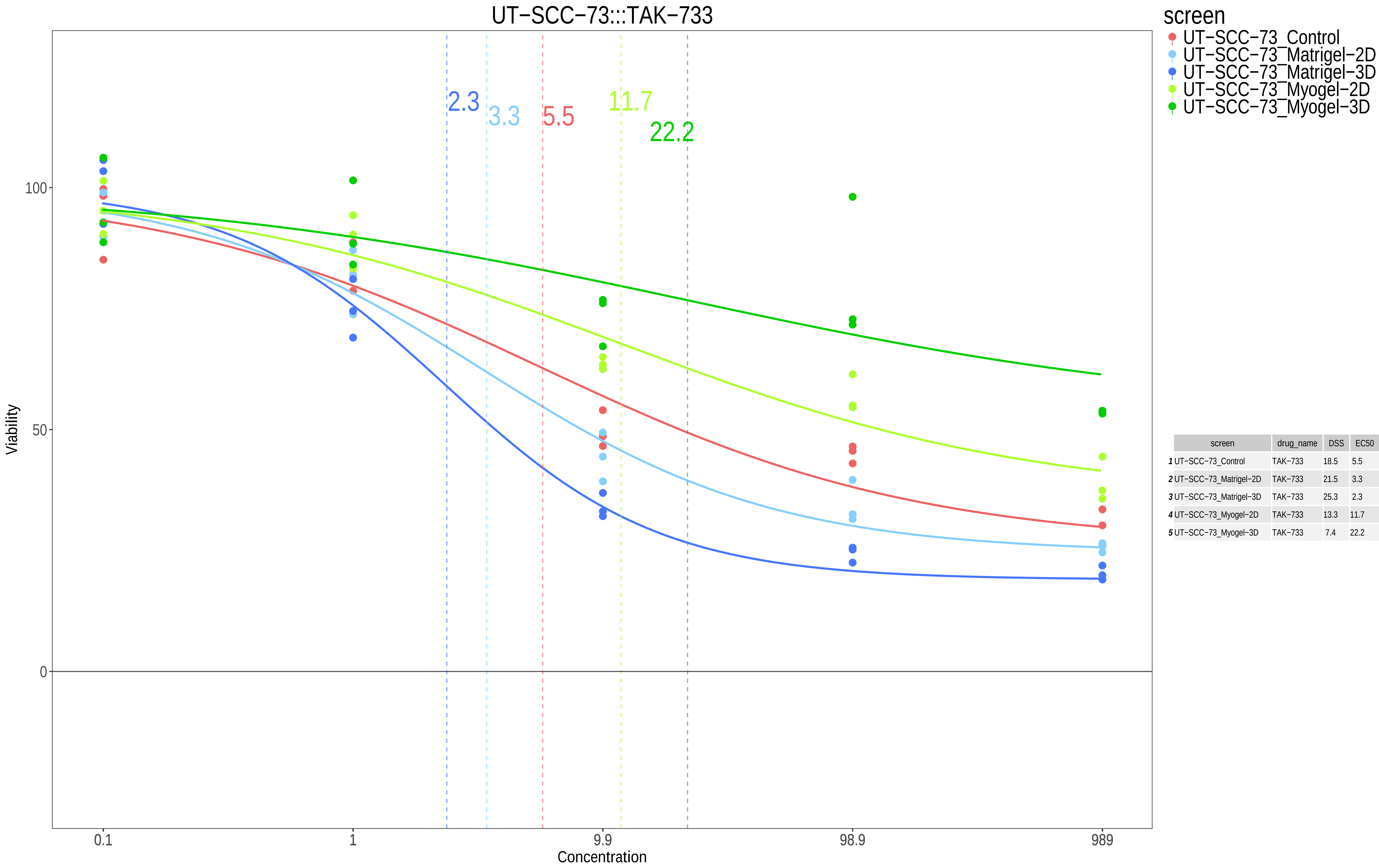

	screen	drug_name	DSS	EC50
1	UT−SCC−73_Control	TAK−733	18.5	5.5
2	UT−SCC−73_Matrigel−2D	TAK−733	21.5	3.3
3	UT−SCC−73_Matrigel−3D	TAK−733	25.3	2.3
4	UT−SCC−73_Myogel−2D	TAK−733	13.3	11.7
5	UT−SCC−73_Myogel−3D	TAK−733	7.4	22.2

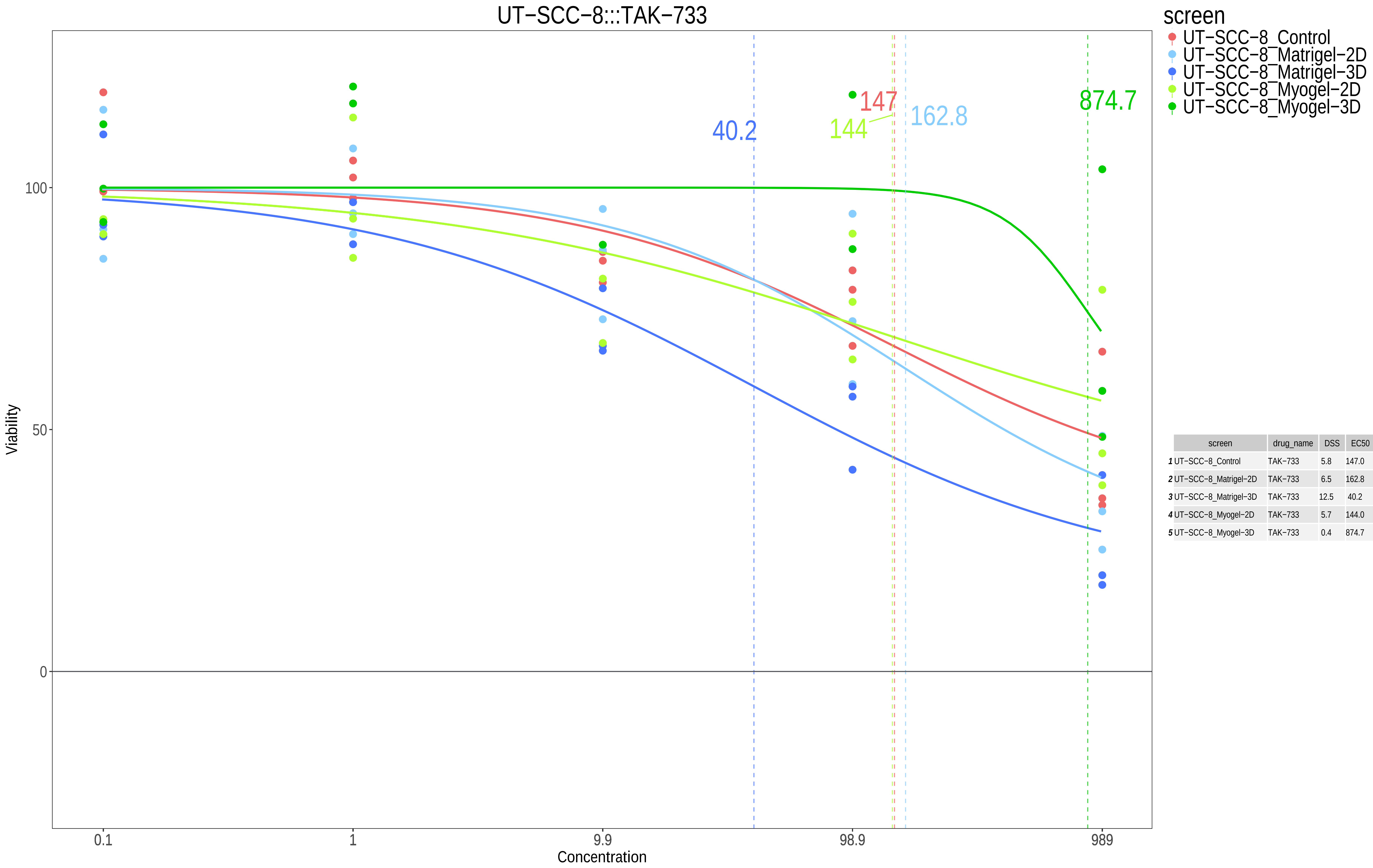

	screen	drug_name	DSS	EC50
1	UT−SCC−8_Control	TAK−733	5.8	147.0
2	UT−SCC−8_Matrigel−2D	TAK−733	6.5	162.8
3	UT−SCC−8_Matrigel−3D	TAK−733	12.5	40.2
4	UT−SCC−8_Myogel−2D	TAK−733	5.7	144.0
5	UT−SCC−8_Myogel−3D	TAK−733	0.4	874.7

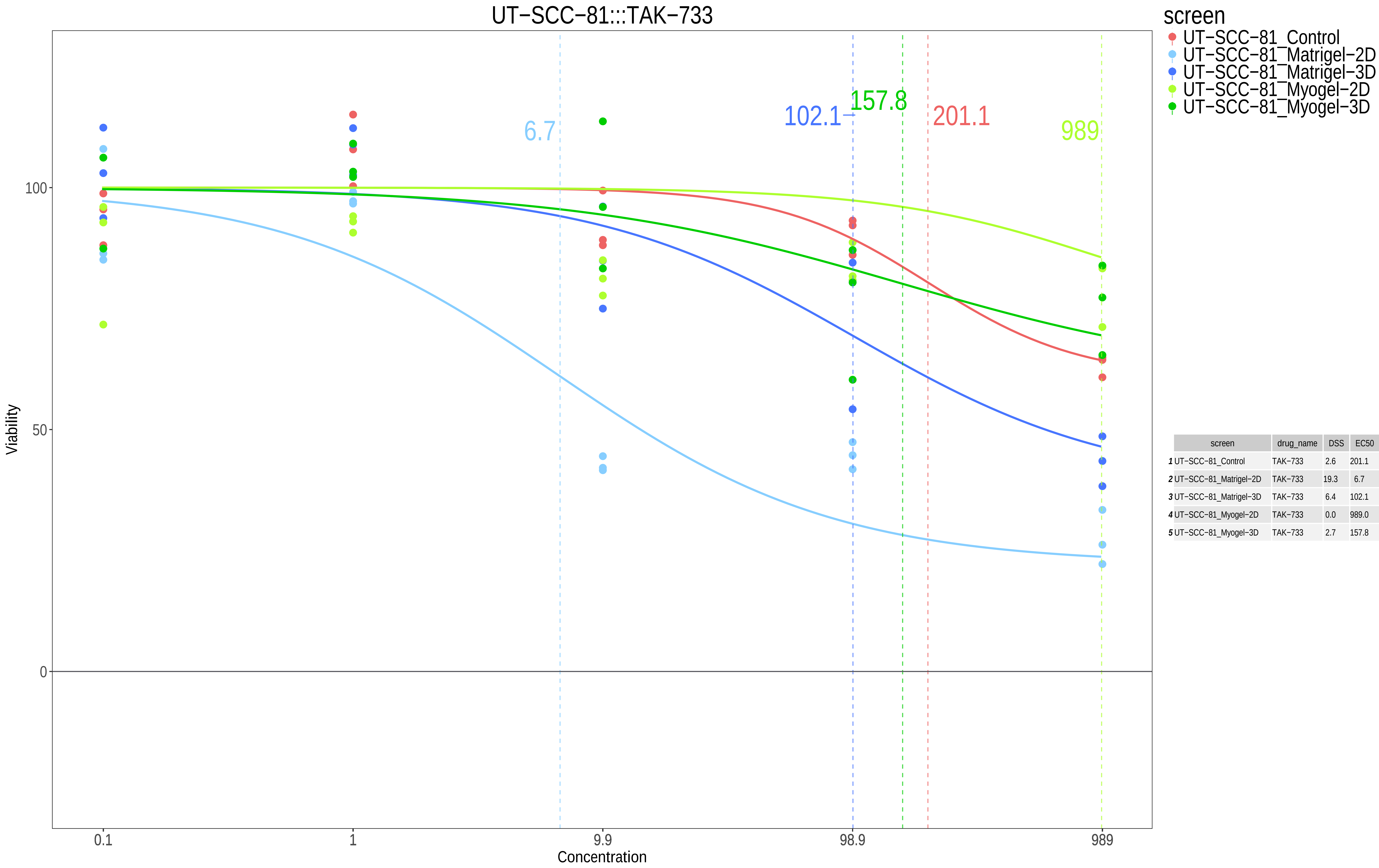

	screen	drug_name	DSS	EC50
1	UT−SCC−81_Control	TAK−733	2.6	201.1
2	UT−SCC−81_Matrigel−2D	TAK−733	19.3	6.7
3	UT−SCC−81_Matrigel−3D	TAK−733	6.4	102.1
4	UT−SCC−81_Myogel−2D	TAK−733	0.0	989.0
5	UT−SCC−81_Myogel−3D	TAK−733	2.7	157.8

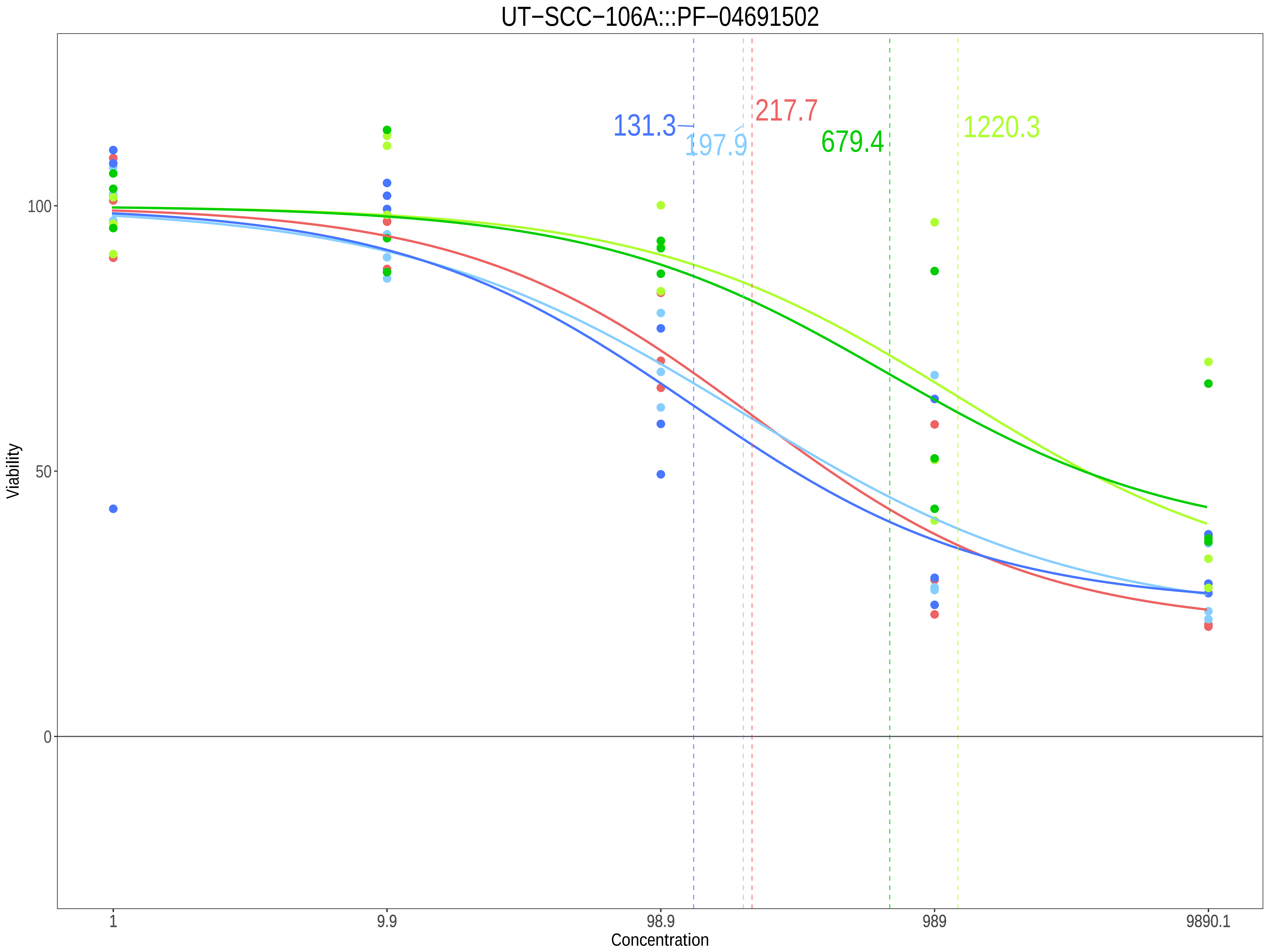

	screen	drug_name	DSS	EC50
1	UT−SCC−106A_Control	PF−04691502	14.7	217.7
2	UT−SCC−106A_Matrigel−2D	PF−04691502	14.7	197.9
3	UT−SCC−106A_Matrigel−3D	PF−04691502	16.0	131.3
4	UT−SCC−106A_Myogel−2D	PF−04691502	7.1	1220.3
5	UT−SCC−106A_Myogel−3D	PF−04691502	7.8	679.4

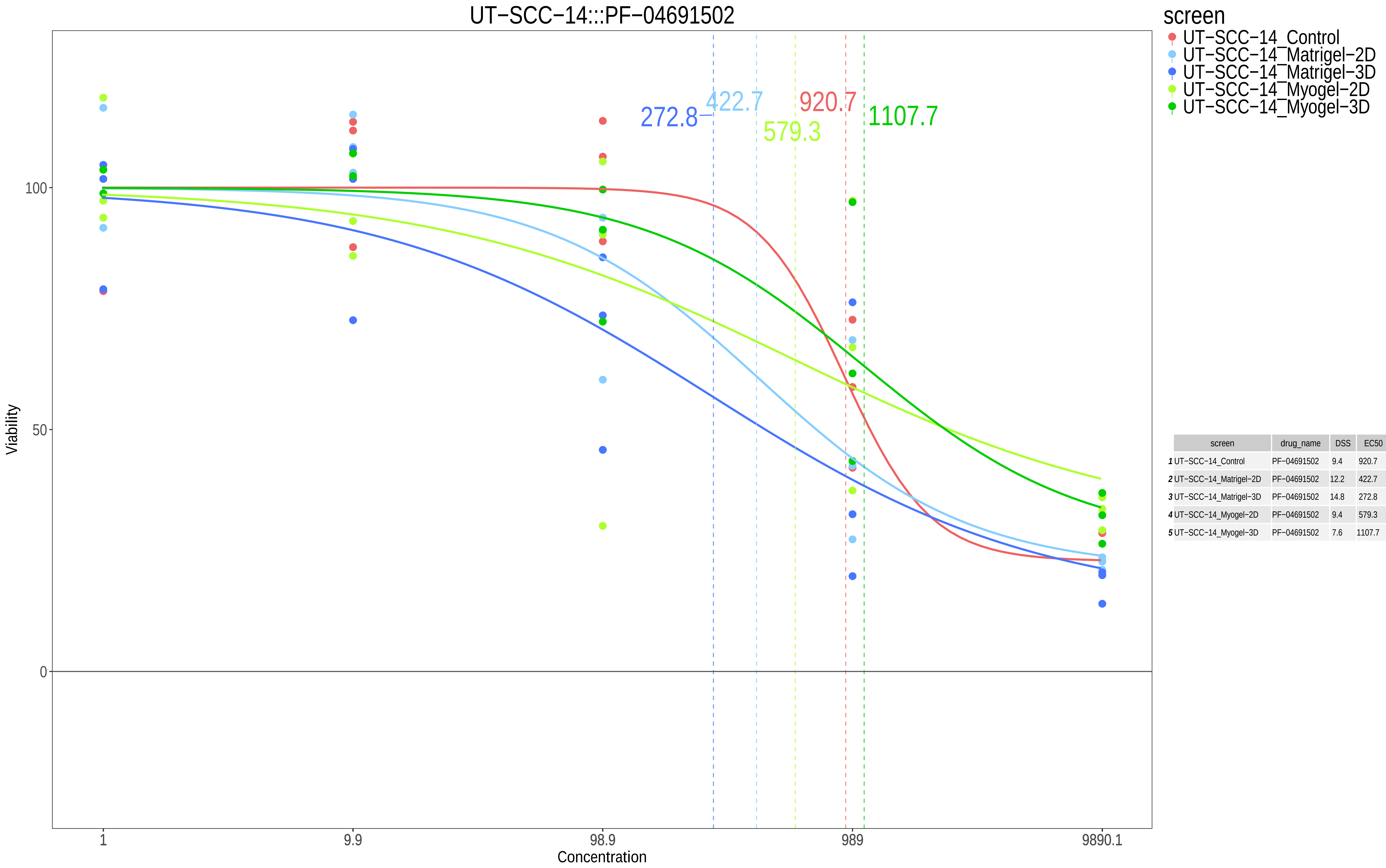

	screen	drug_name	DSS	EC50
1	UT−SCC−14_Control	PF−04691502	9.4	920.7
2	UT−SCC−14_Matrigel−2D	PF−04691502	12.2	422.7
3	UT−SCC−14_Matrigel−3D	PF−04691502	14.8	272.8
4	UT−SCC−14_Myogel−2D	PF−04691502	9.4	579.3
5	UT−SCC−14_Myogel−3D	PF−04691502	7.6	1107.7

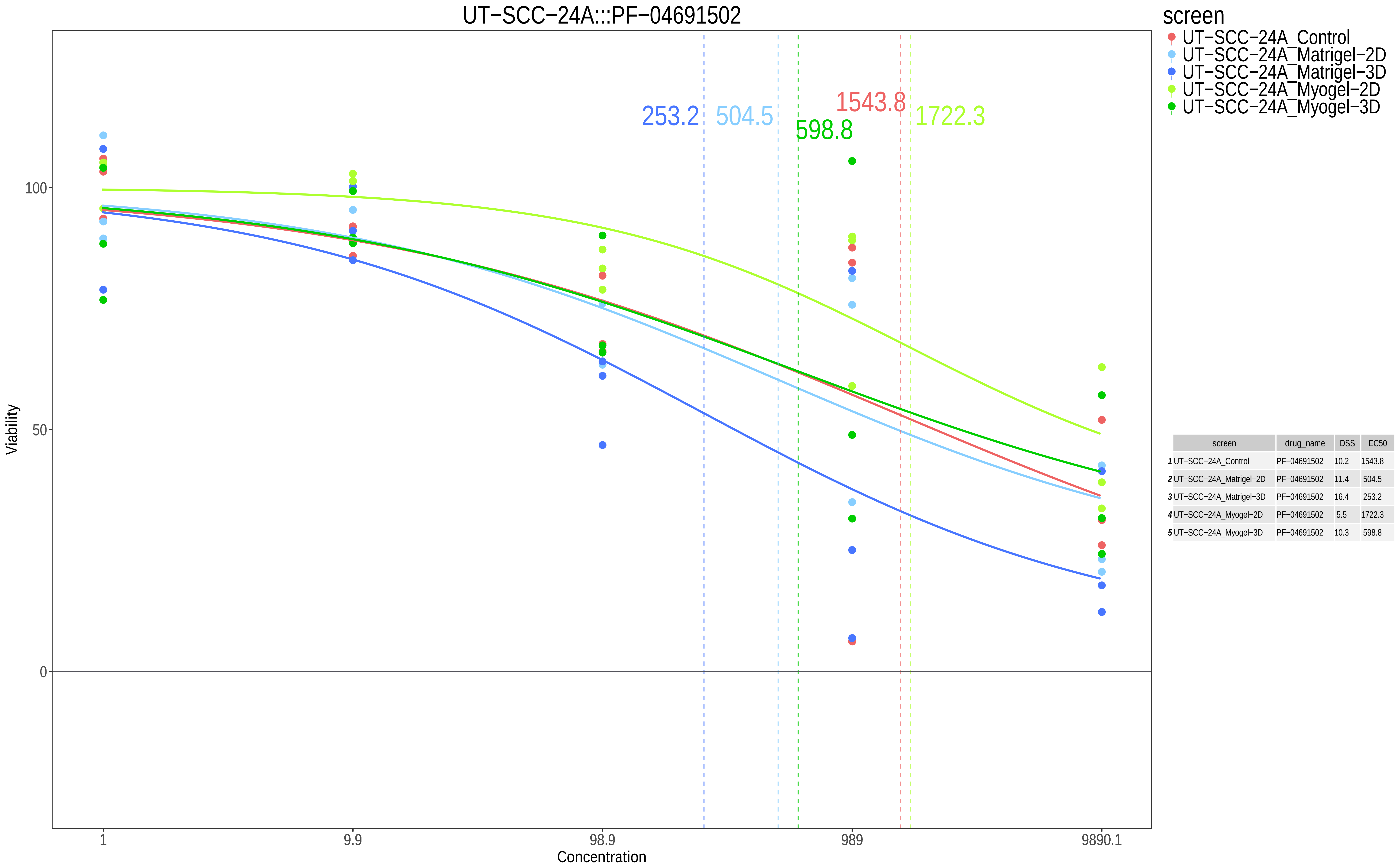

	screen	drug_name	DSS	EC50
1	UT−SCC−24A_Control	PF−04691502	10.2	1543.8
2	UT−SCC−24A_Matrigel−2D	PF−04691502	11.4	504.5
3	UT−SCC−24A_Matrigel−3D	PF−04691502	16.4	253.2
4	UT−SCC−24A_Myogel−2D	PF−04691502	5.5	1722.3
5	UT−SCC−24A_Myogel−3D	PF−04691502	10.3	598.8

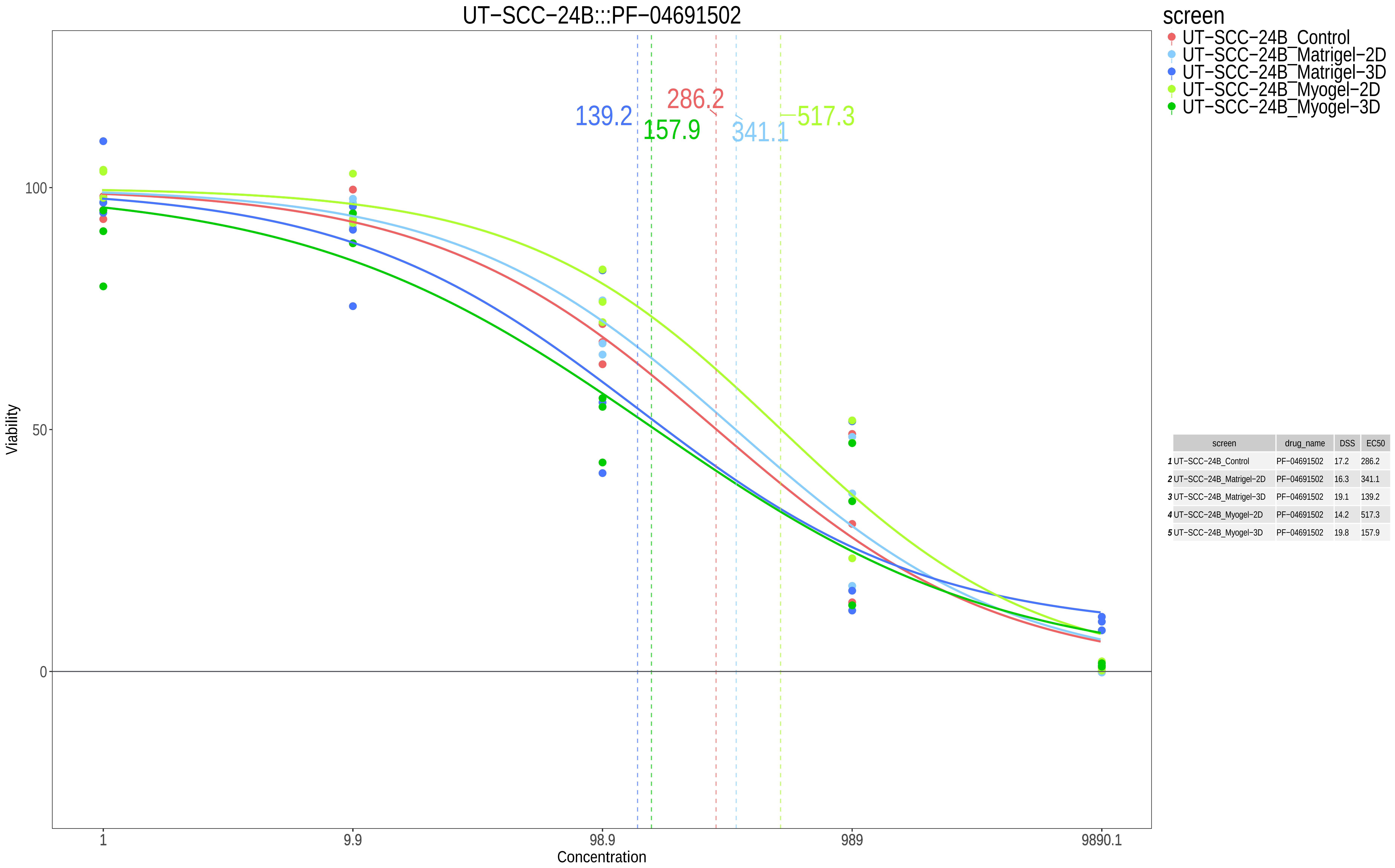

	screen	drug_name	DSS	EC50
1	UT−SCC−24B_Control	PF−04691502	17.2	286.2
2	UT−SCC−24B_Matrigel−2D	PF−04691502	16.3	341.1
3	UT−SCC−24B_Matrigel−3D	PF−04691502	19.1	139.2
4	UT−SCC−24B_Myogel−2D	PF−04691502	14.2	517.3
5	UT−SCC−24B_Myogel−3D	PF−04691502	19.8	157.9

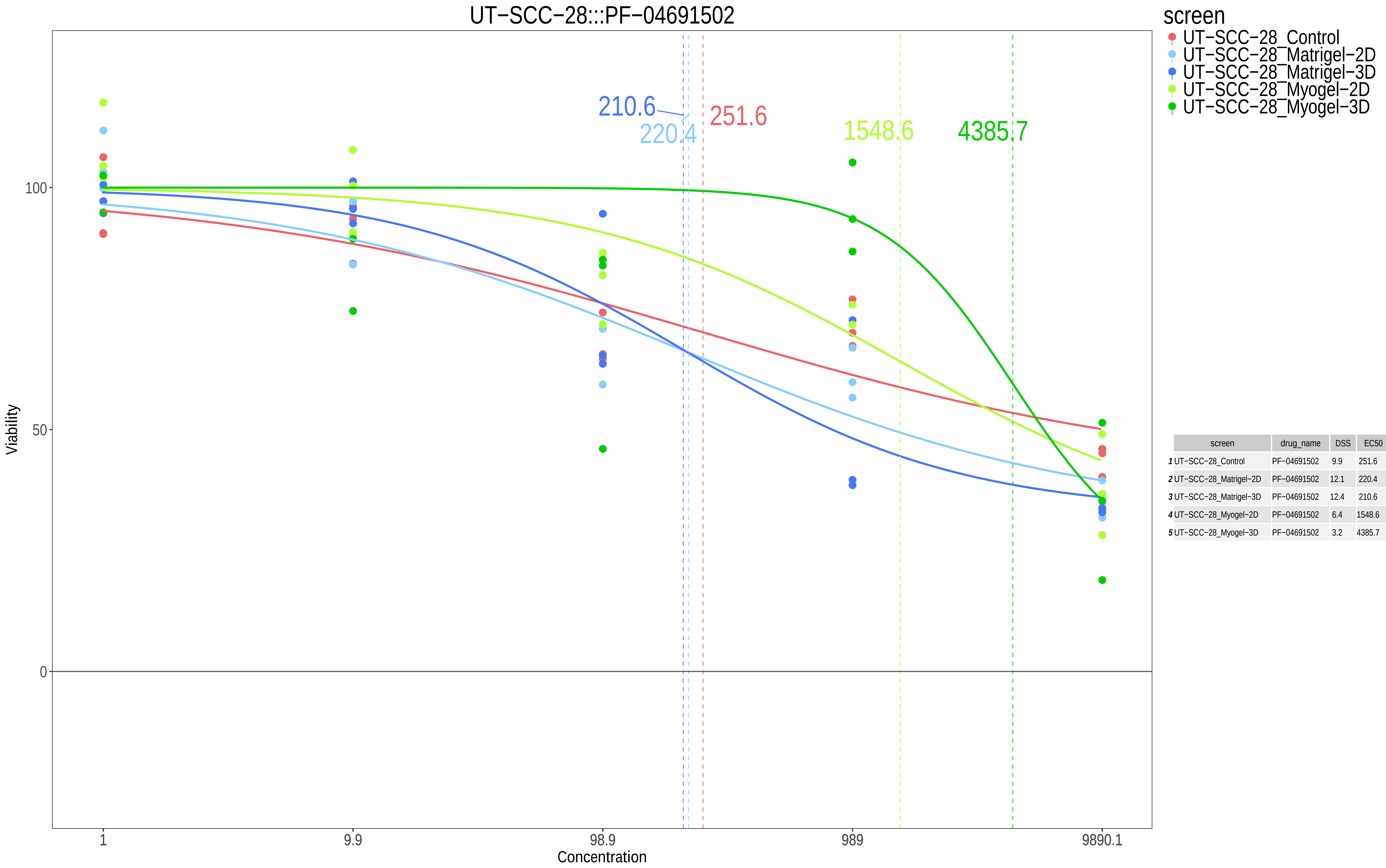

	screen	drug_name	DSS	EC50
1	UT−SCC−28_Control	PF−04691502	9.9	251.6
2	UT−SCC−28_Matrigel−2D	PF−04691502	12.1	220.4
3	UT−SCC−28_Matrigel−3D	PF−04691502	12.4	210.6
4	UT−SCC−28_Myogel−2D	PF−04691502	6.4	1548.6
5	UT−SCC−28_Myogel−3D	PF−04691502	3.2	4385.7

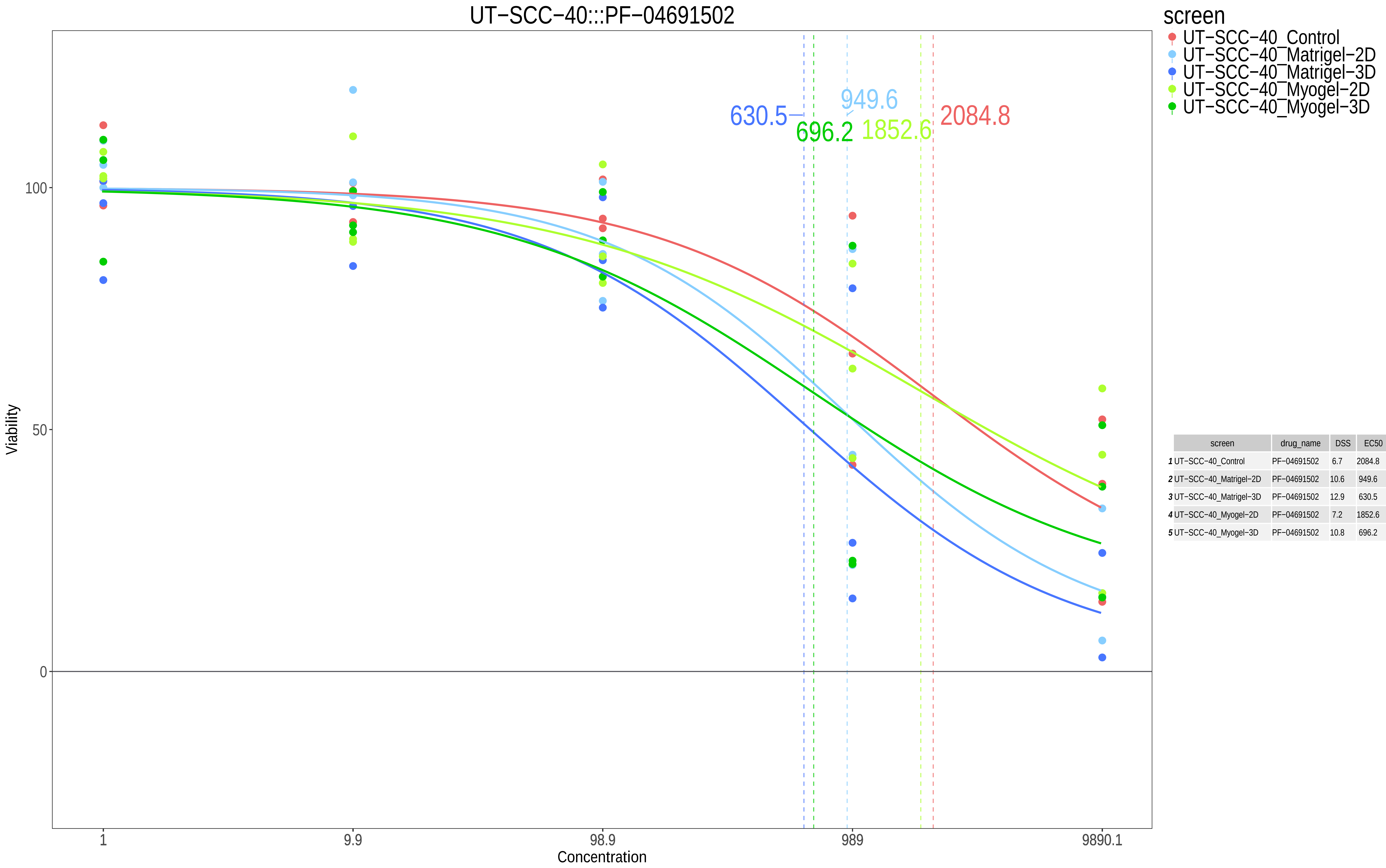

	screen	drug_name	DSS	EC50
1	UT−SCC−40_Control	PF−04691502	6.7	2084.8
2	UT−SCC−40_Matrigel−2D	PF−04691502	10.6	949.6
3	UT−SCC−40_Matrigel−3D	PF−04691502	12.9	630.5
4	UT−SCC−40_Myogel−2D	PF−04691502	7.2	1852.6
5	UT−SCC−40_Myogel−3D	PF−04691502	10.8	696.2

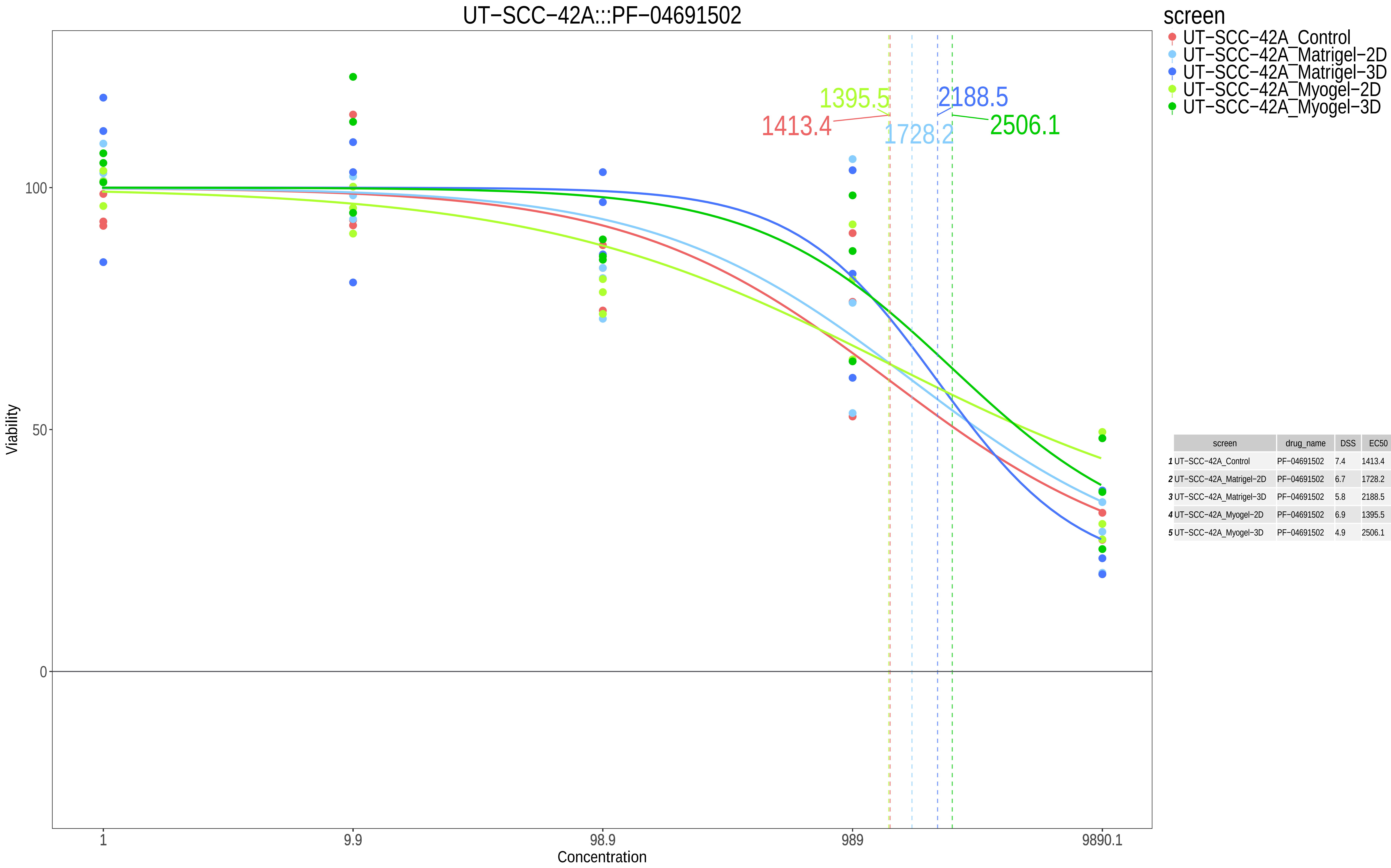

	screen	drug_name	DSS	EC50
1	UT−SCC−42A_Control	PF−04691502	7.4	1413.4
2	UT−SCC−42A_Matrigel−2D	PF−04691502	6.7	1728.2
3	UT−SCC−42A_Matrigel−3D	PF−04691502	5.8	2188.5
4	UT−SCC−42A_Myogel−2D	PF−04691502	6.9	1395.5
5	UT−SCC−42A_Myogel−3D	PF−04691502	4.9	2506.1

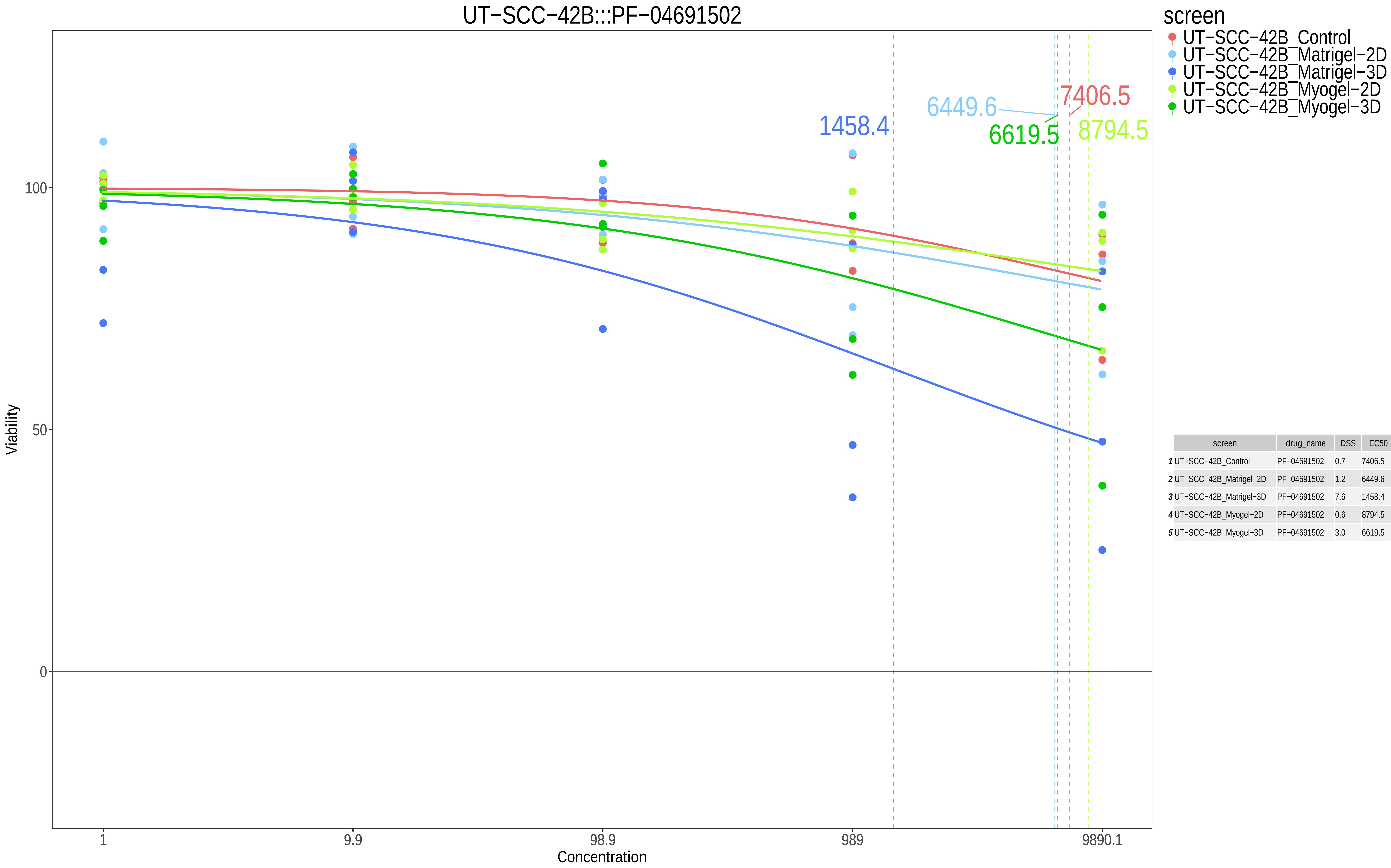

	screen	drug_name	DSS	EC50
1	UT−SCC−42B_Control	PF−04691502	0.7	7406.5
2	UT−SCC−42B_Matrigel−2D	PF−04691502	1.2	6449.6
3	UT−SCC−42B_Matrigel−3D	PF−04691502	7.6	1458.4
4	UT−SCC−42B_Myogel−2D	PF−04691502	0.6	8794.5
5	UT−SCC−42B_Myogel−3D	PF−04691502	3.0	6619.5

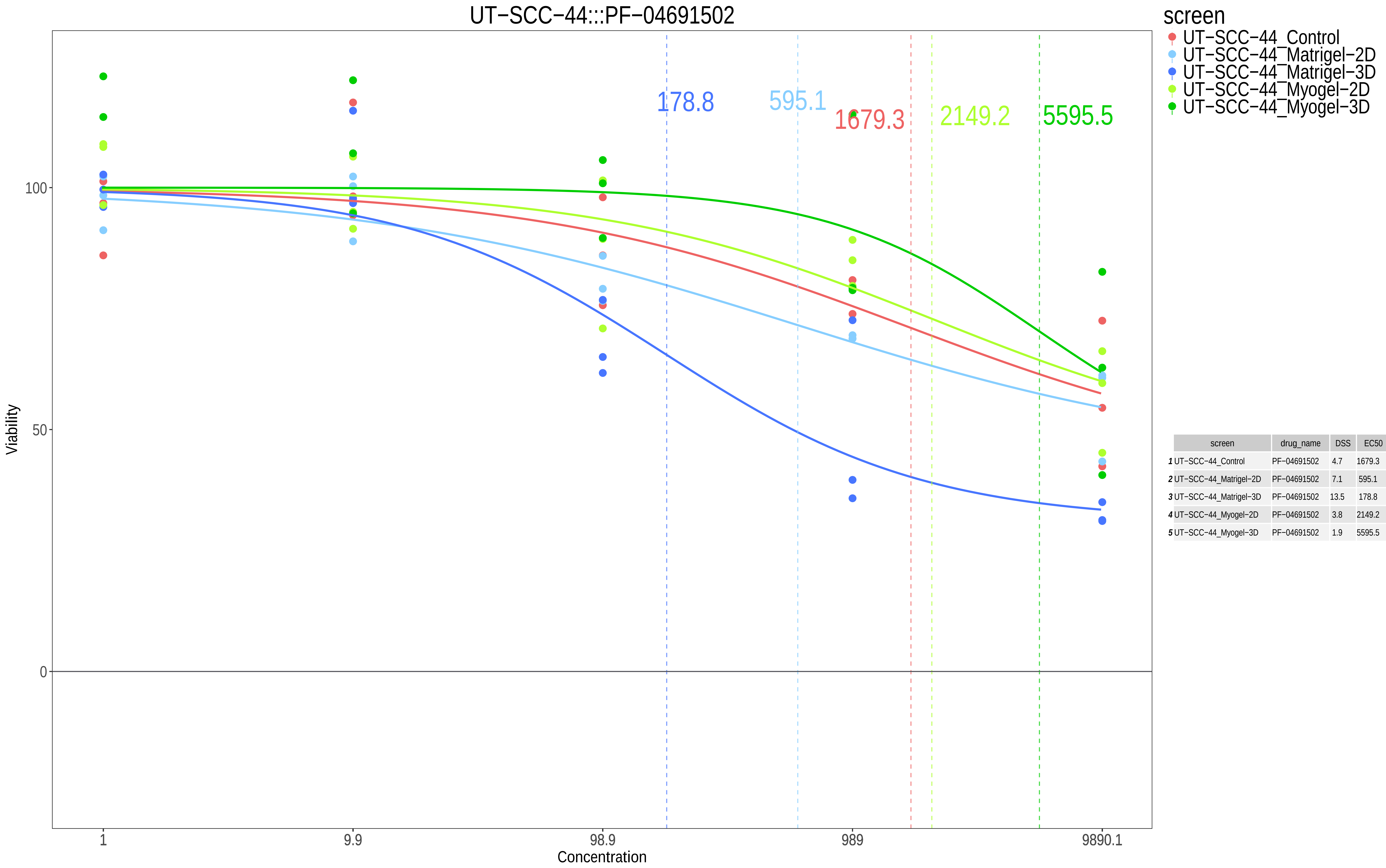

	screen	drug_name	DSS	EC50
1	UT−SCC−44_Control	PF−04691502	4.7	1679.3
2	UT−SCC−44_Matrigel−2D	PF−04691502	7.1	595.1
3	UT−SCC−44_Matrigel−3D	PF−04691502	13.5	178.8
4	UT−SCC−44_Myogel−2D	PF−04691502	3.8	2149.2
5	UT−SCC−44_Myogel−3D	PF−04691502	1.9	5595.5

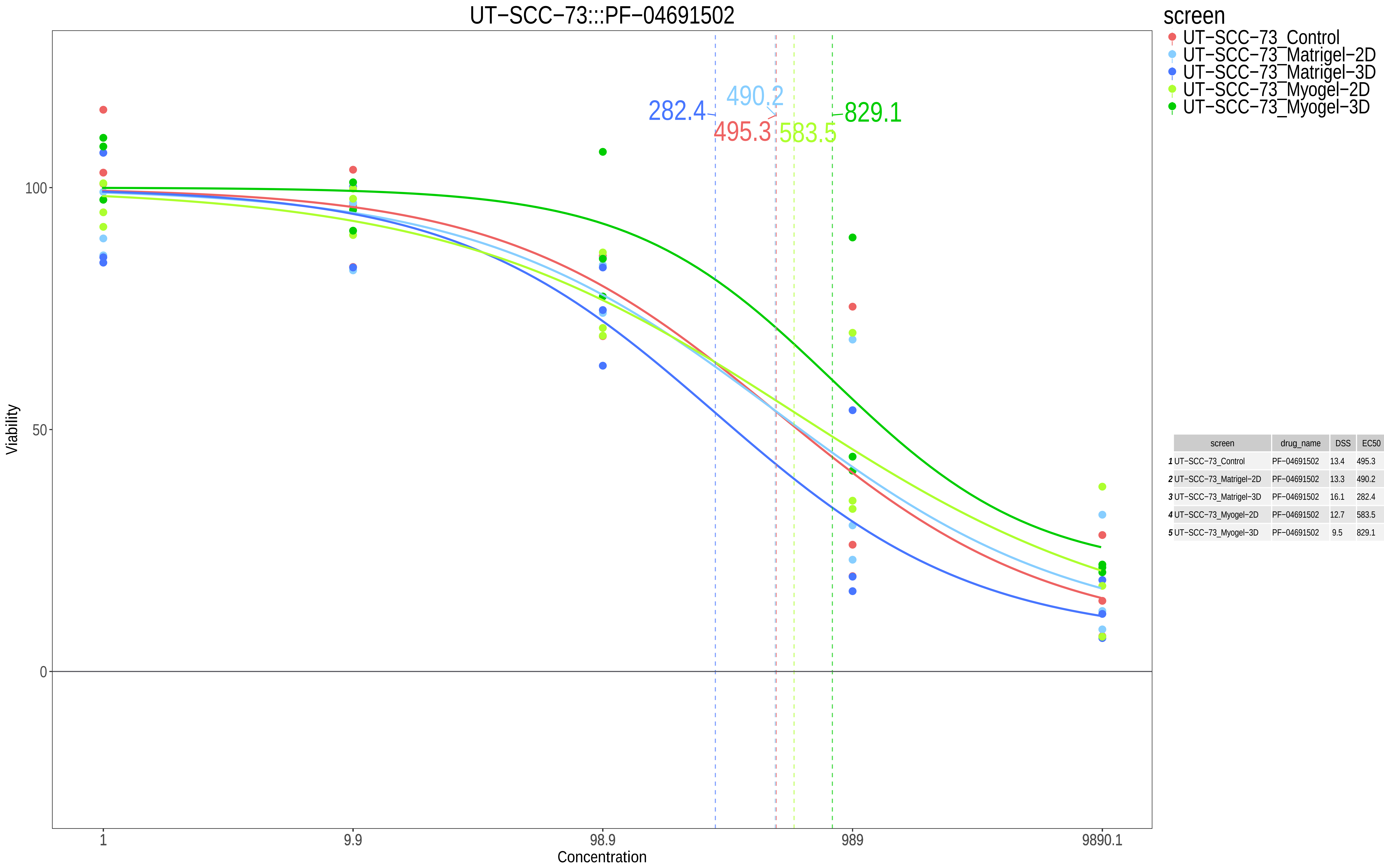

	screen	drug_name	DSS	EC50
1	UT−SCC−73_Control	PF−04691502	13.4	495.3
2	UT−SCC−73_Matrigel−2D	PF−04691502	13.3	490.2
3	UT−SCC−73_Matrigel−3D	PF−04691502	16.1	282.4
4	UT−SCC−73_Myogel−2D	PF−04691502	12.7	583.5
5	UT−SCC−73_Myogel−3D	PF−04691502	9.5	829.1

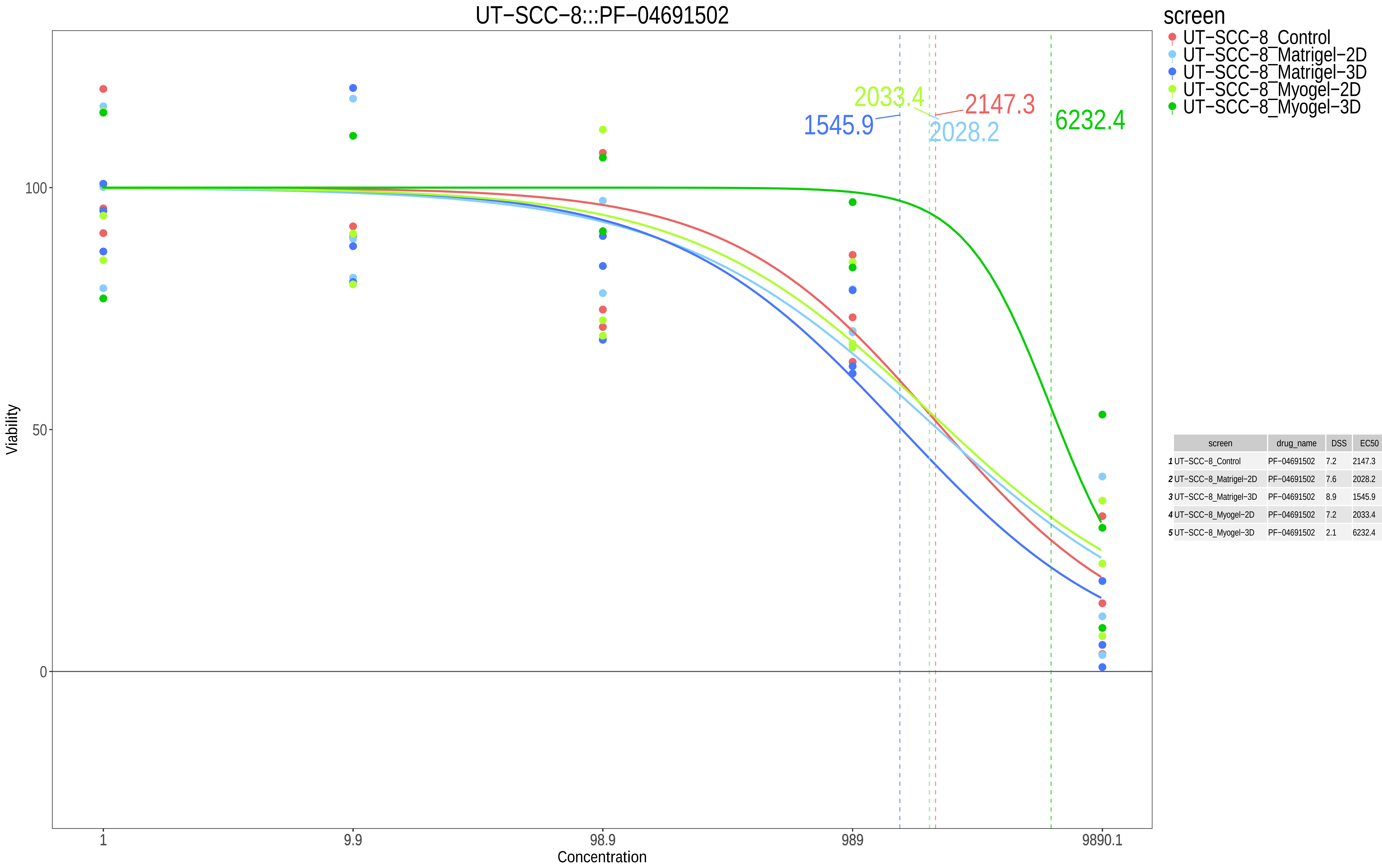

	screen	drug_name	DSS	EC50
1	UT−SCC−8_Control	PF−04691502	7.2	2147.3
2	UT−SCC−8_Matrigel−2D	PF−04691502	7.6	2028.2
3	UT−SCC−8_Matrigel−3D	PF−04691502	8.9	1545.9
4	UT−SCC−8_Myogel−2D	PF−04691502	7.2	2033.4
5	UT−SCC−8_Myogel−3D	PF−04691502	2.1	6232.4

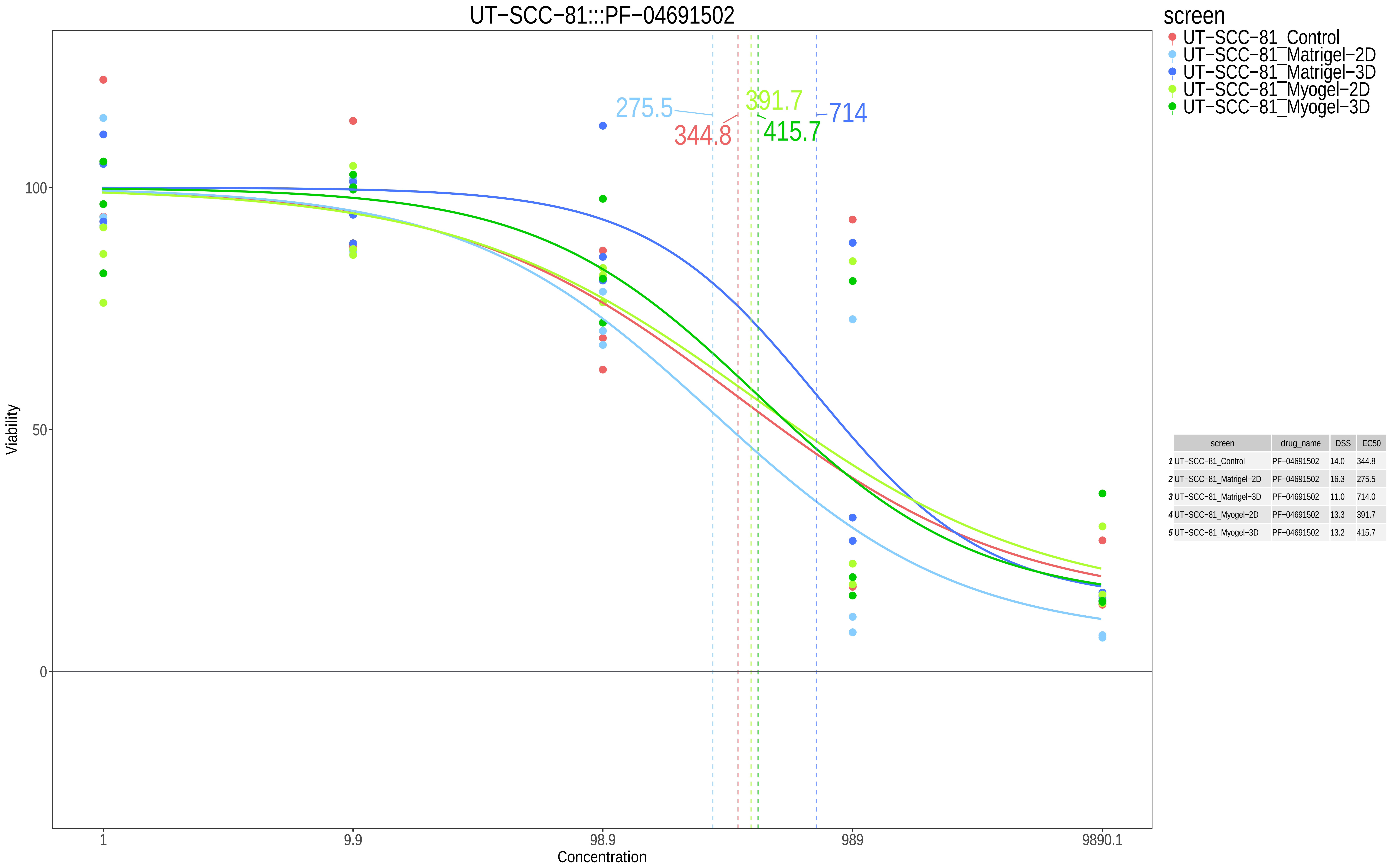

	screen	drug_name	DSS	EC50
1	UT−SCC−81_Control	PF−04691502	14.0	344.8
2	UT−SCC−81_Matrigel−2D	PF−04691502	16.3	275.5
3	UT−SCC−81_Matrigel−3D	PF−04691502	11.0	714.0
4	UT−SCC−81_Myogel−2D	PF−04691502	13.3	391.7
5	UT−SCC−81_Myogel−3D	PF−04691502	13.2	415.7

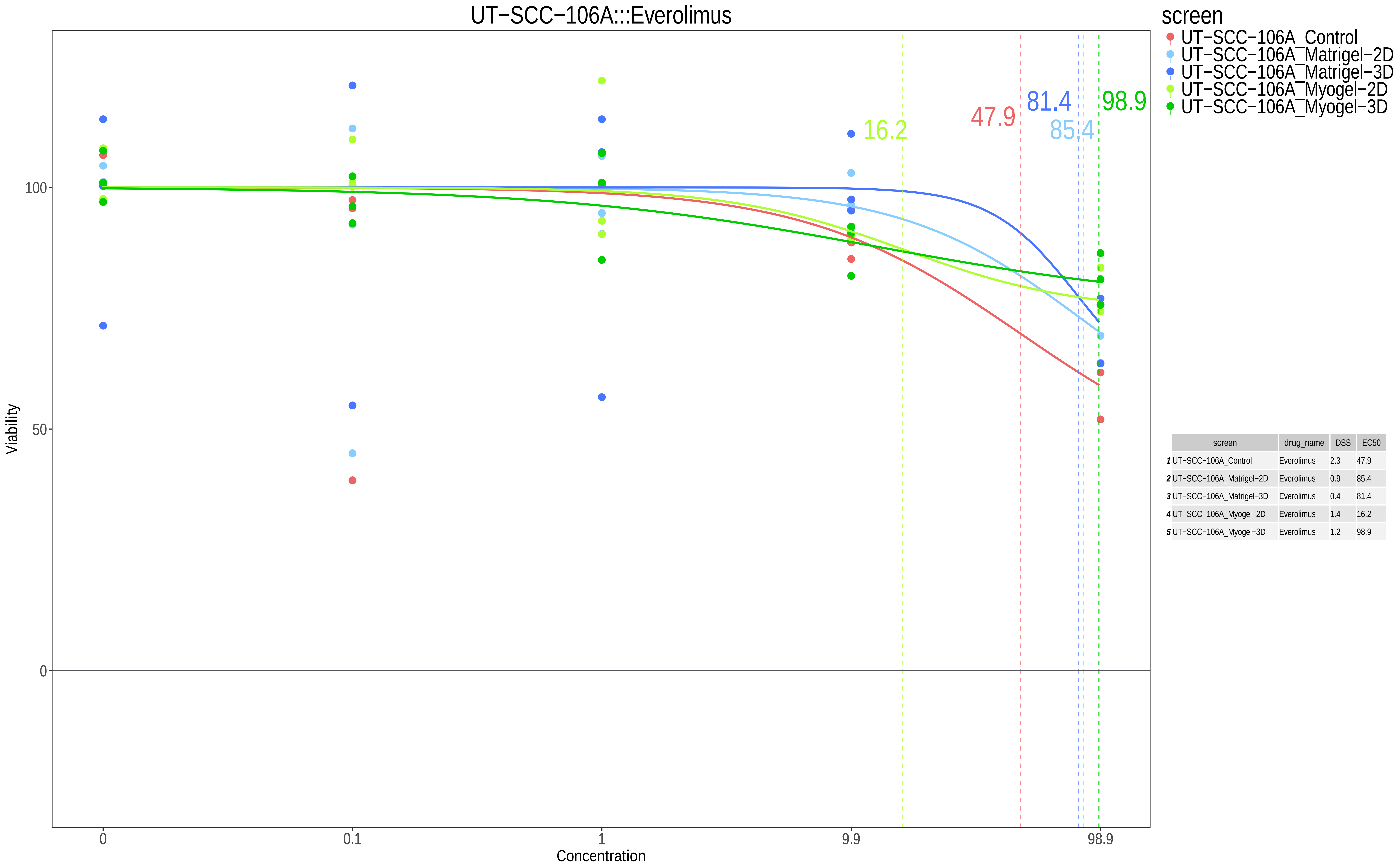

	screen	drug_name	DSS	EC50
1	UT−SCC−106A_Control	Everolimus	2.3	47.9
2	UT−SCC−106A_Matrigel−2D	Everolimus	0.9	85.4
3	UT−SCC−106A_Matrigel−3D	Everolimus	0.4	81.4
4	UT−SCC−106A_Myogel−2D	Everolimus	1.4	16.2
5	UT−SCC−106A_Myogel−3D	Everolimus	1.2	98.9

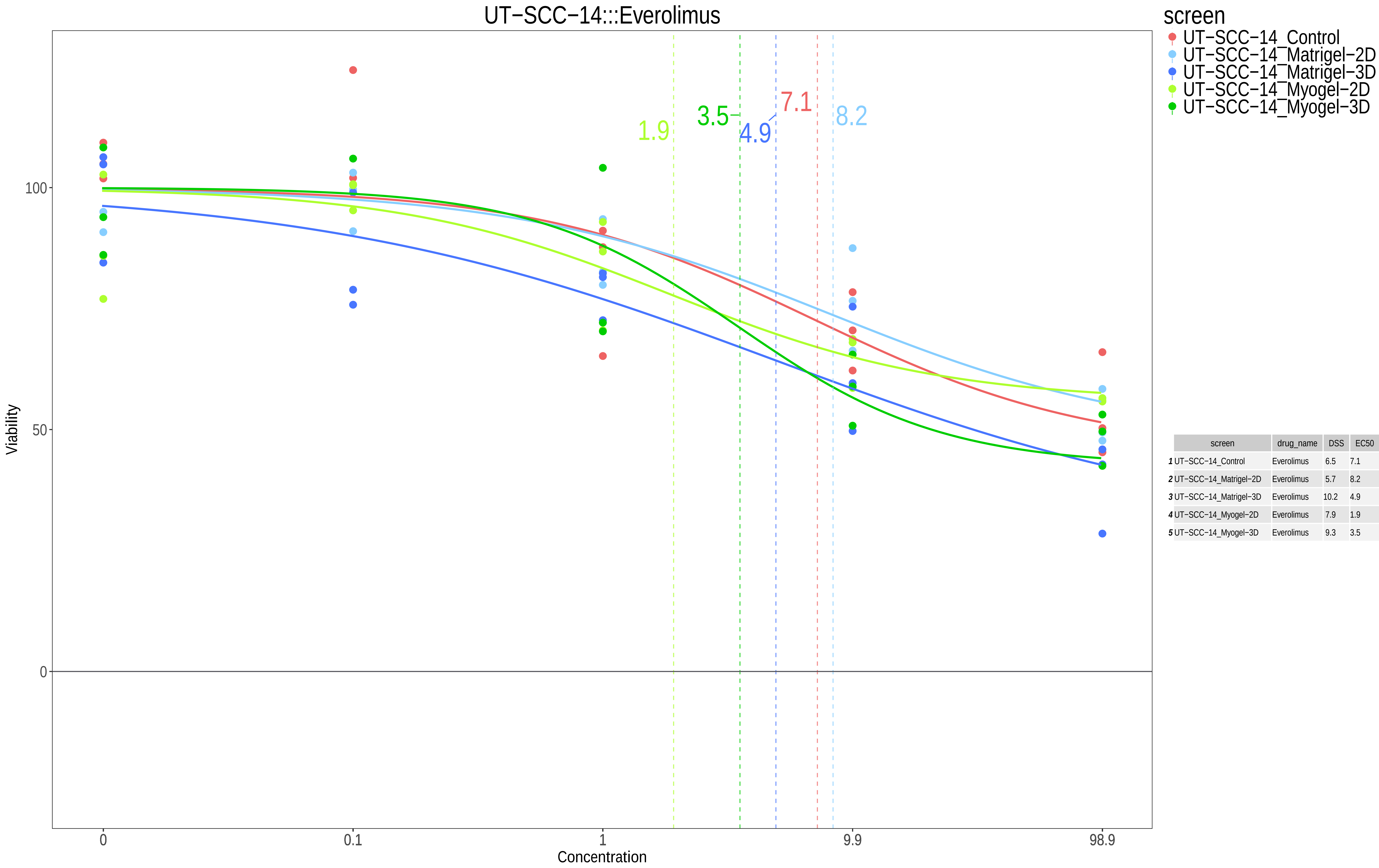

	screen	drug_name	DSS	EC50
1	UT−SCC−14_Control	Everolimus	6.5	7.1
2	UT−SCC−14_Matrigel−2D	Everolimus	5.7	8.2
3	UT−SCC−14_Matrigel−3D	Everolimus	10.2	4.9
4	UT−SCC−14_Myogel−2D	Everolimus	7.9	1.9
5	UT−SCC−14_Myogel−3D	Everolimus	9.3	3.5

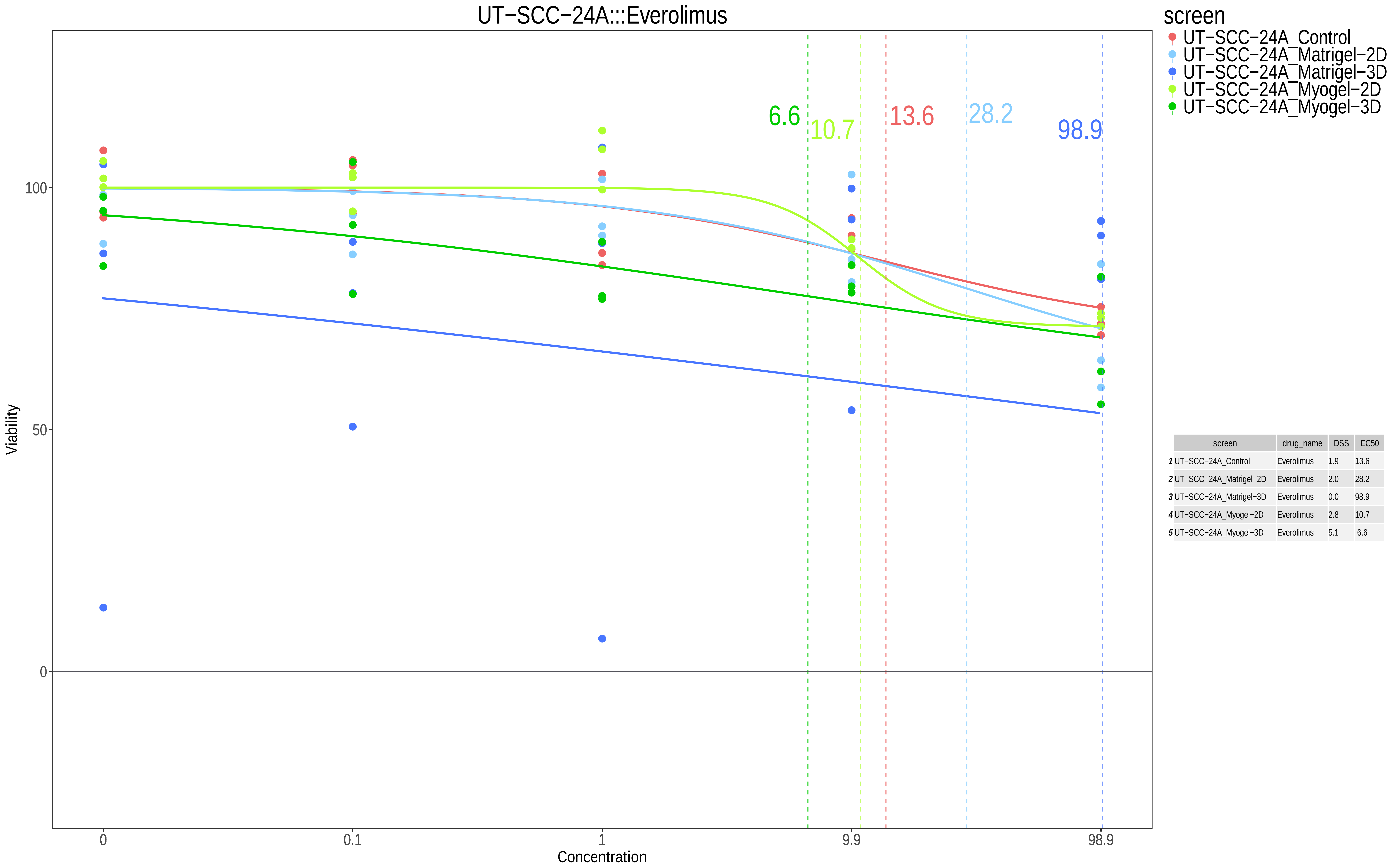

	screen	drug_name	DSS	EC50
1	UT−SCC−24A_Control	Everolimus	1.9	13.6
2	UT−SCC−24A_Matrigel−2D	Everolimus	2.0	28.2
3	UT−SCC−24A_Matrigel−3D	Everolimus	0.0	98.9
4	UT−SCC−24A_Myogel−2D	Everolimus	2.8	10.7
5	UT−SCC−24A_Myogel−3D	Everolimus	5.1	6.6

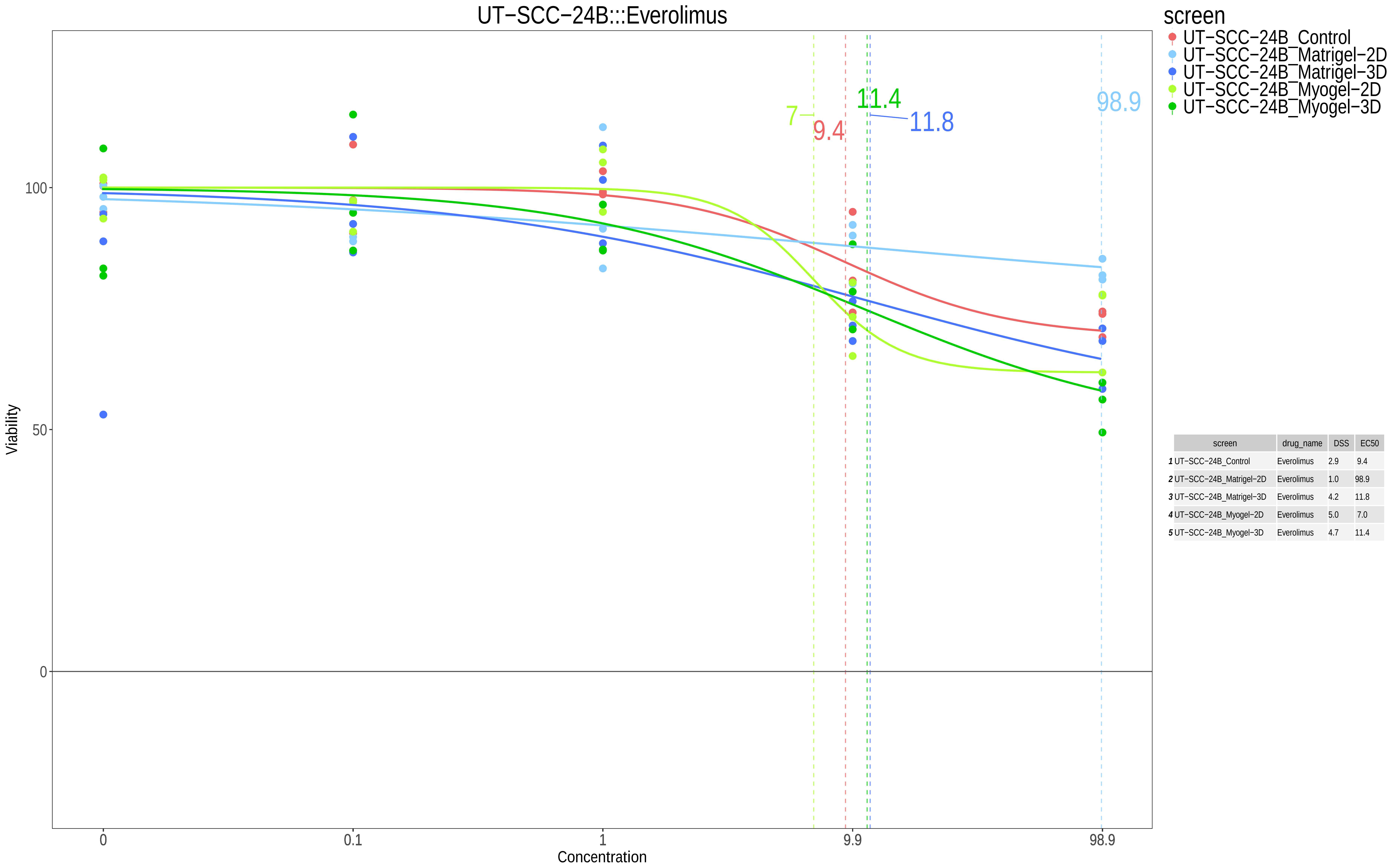

	screen	drug_name	DSS	EC50
1	UT−SCC−24B_Control	Everolimus	2.9	9.4
2	UT−SCC−24B_Matrigel−2D	Everolimus	1.0	98.9
3	UT−SCC−24B_Matrigel−3D	Everolimus	4.2	11.8
4	UT−SCC−24B_Myogel−2D	Everolimus	5.0	7.0
5	UT−SCC−24B_Myogel−3D	Everolimus	4.7	11.4

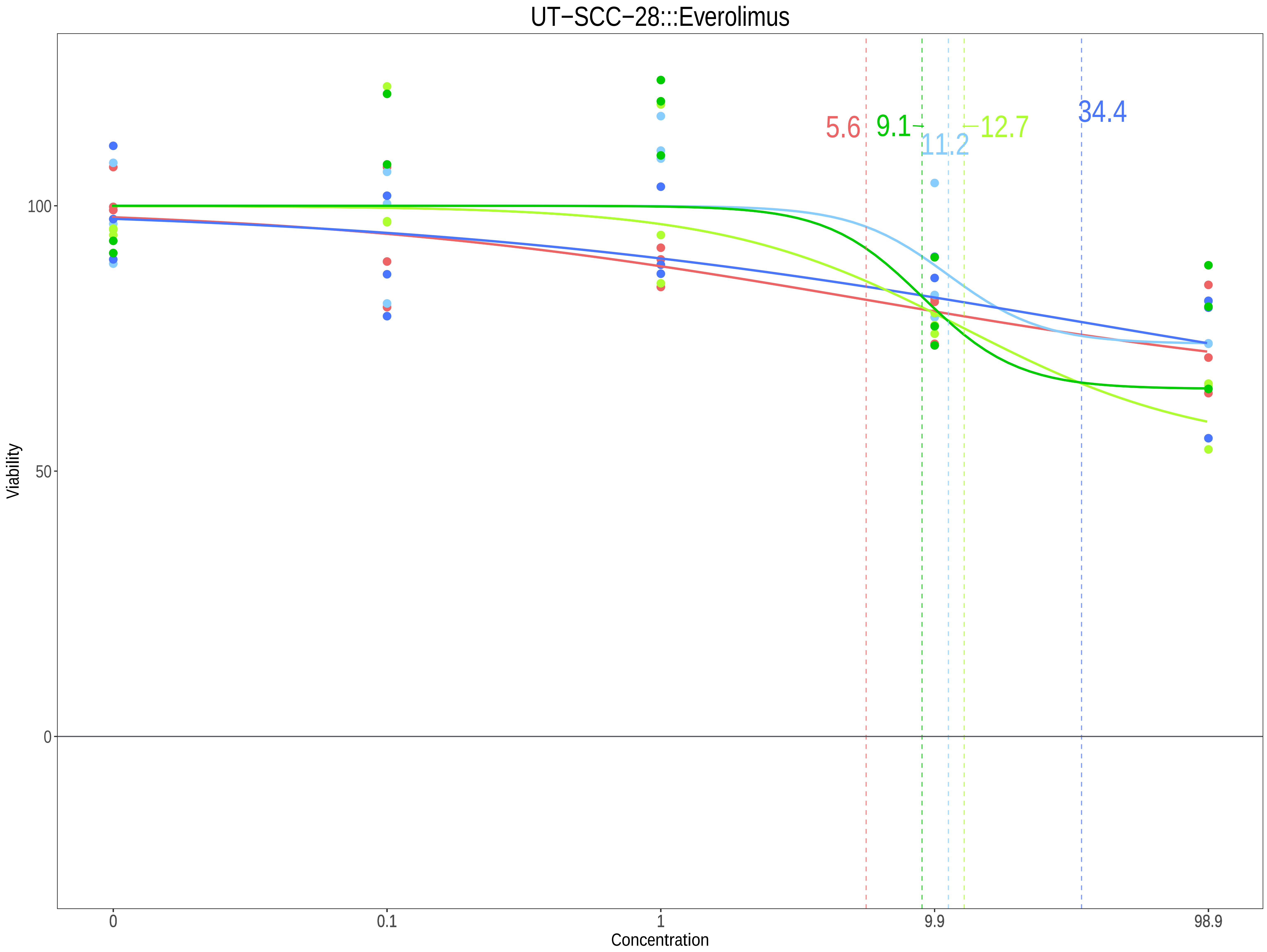

	screen	drug_name	DSS	EC50
1	UT−SCC−28_Control	Everolimus	3.5	5.6
2	UT−SCC−28_Matrigel−2D	Everolimus	2.4	11.2
3	UT−SCC−28_Matrigel−3D	Everolimus	2.5	34.4
4	UT−SCC−28_Myogel−2D	Everolimus	4.0	12.7
5	UT−SCC−28_Myogel−3D	Everolimus	3.9	9.1

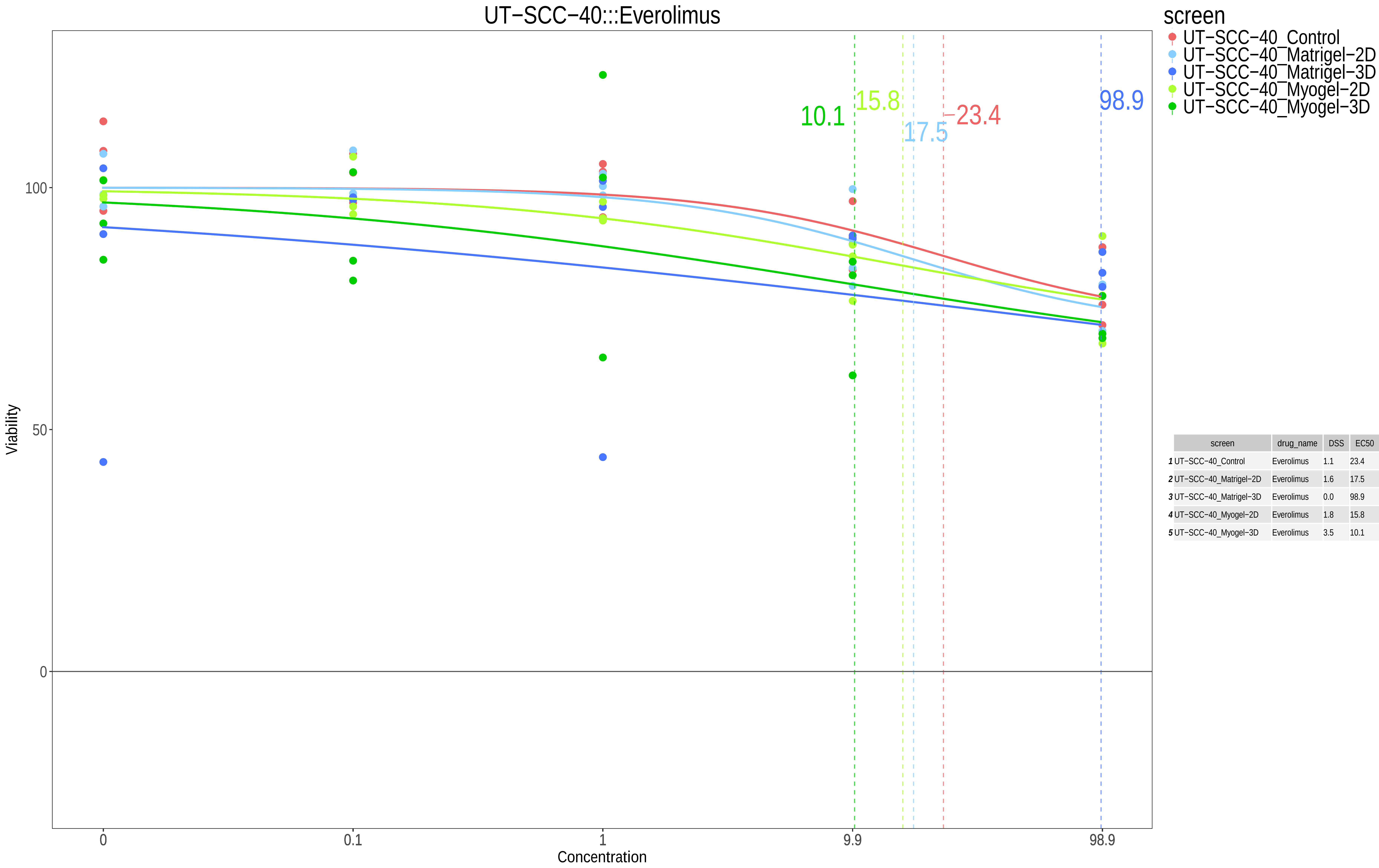

	screen	drug_name	DSS	EC50
1	UT−SCC−40_Control	Everolimus	1.1	23.4
2	UT−SCC−40_Matrigel−2D	Everolimus	1.6	17.5
3	UT−SCC−40_Matrigel−3D	Everolimus	0.0	98.9
4	UT−SCC−40_Myogel−2D	Everolimus	1.8	15.8
5	UT−SCC−40_Myogel−3D	Everolimus	3.5	10.1

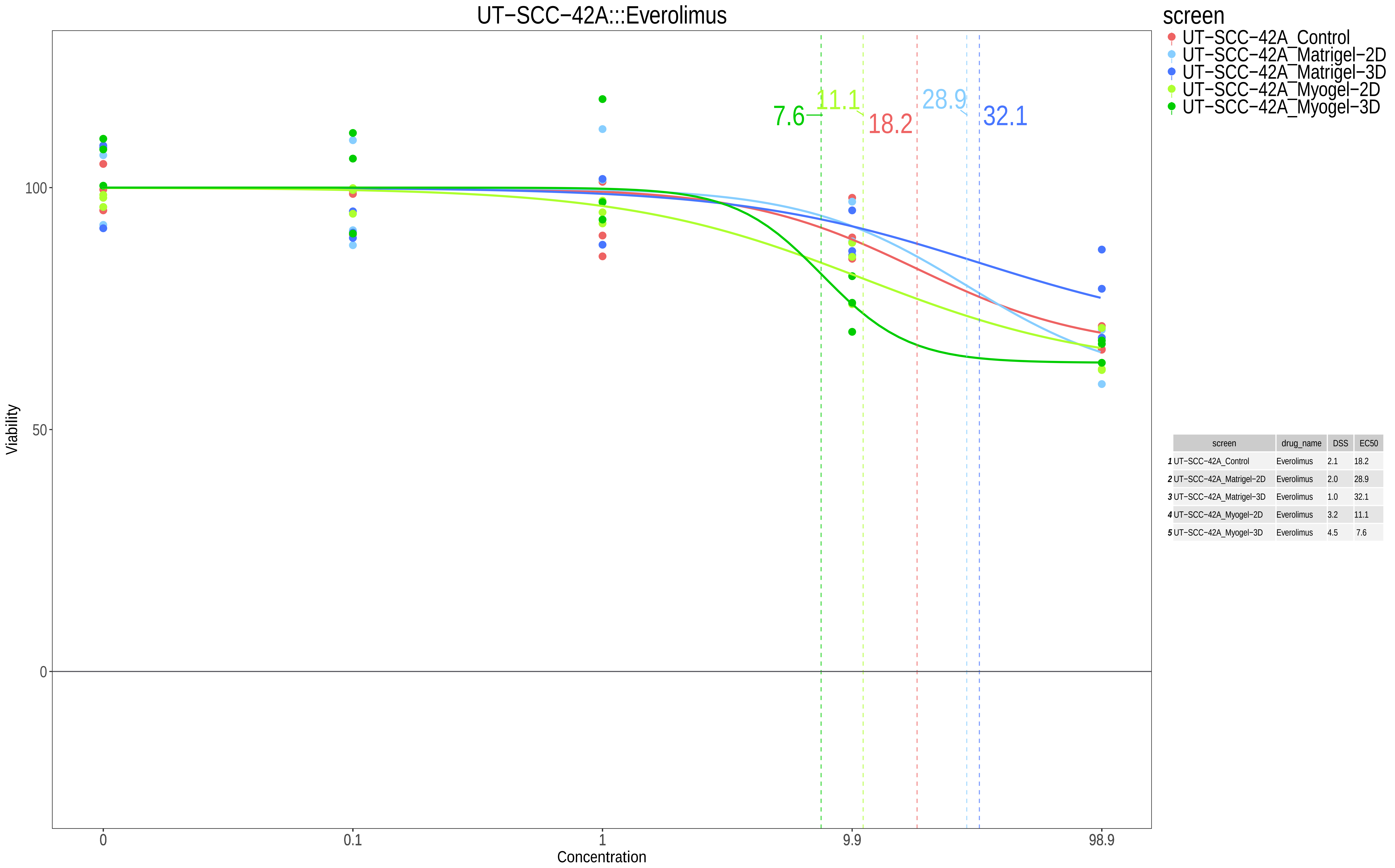

	screen	drug_name	DSS	EC50
1	UT−SCC−42A_Control	Everolimus	2.1	18.2
2	UT−SCC−42A_Matrigel−2D	Everolimus	2.0	28.9
3	UT−SCC−42A_Matrigel−3D	Everolimus	1.0	32.1
4	UT−SCC−42A_Myogel−2D	Everolimus	3.2	11.1
5	UT−SCC−42A_Myogel−3D	Everolimus	4.5	7.6

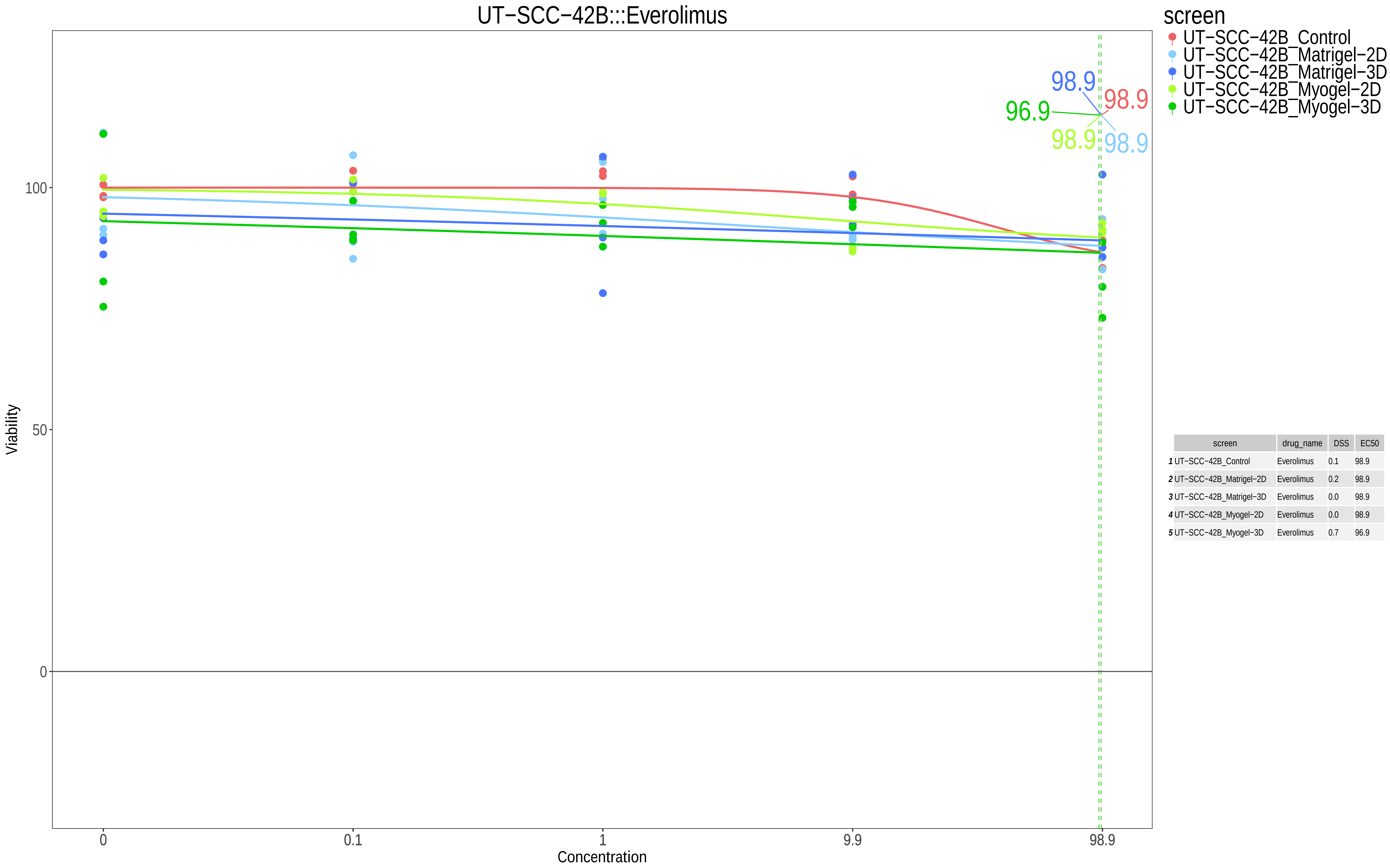

	screen	drug_name	DSS	EC50
1	UT−SCC−42B_Control	Everolimus	0.1	98.9
2	UT−SCC−42B_Matrigel−2D	Everolimus	0.2	98.9
3	UT−SCC−42B_Matrigel−3D	Everolimus	0.0	98.9
4	UT−SCC−42B_Myogel−2D	Everolimus	0.0	98.9
5	UT−SCC−42B_Myogel−3D	Everolimus	0.7	96.9

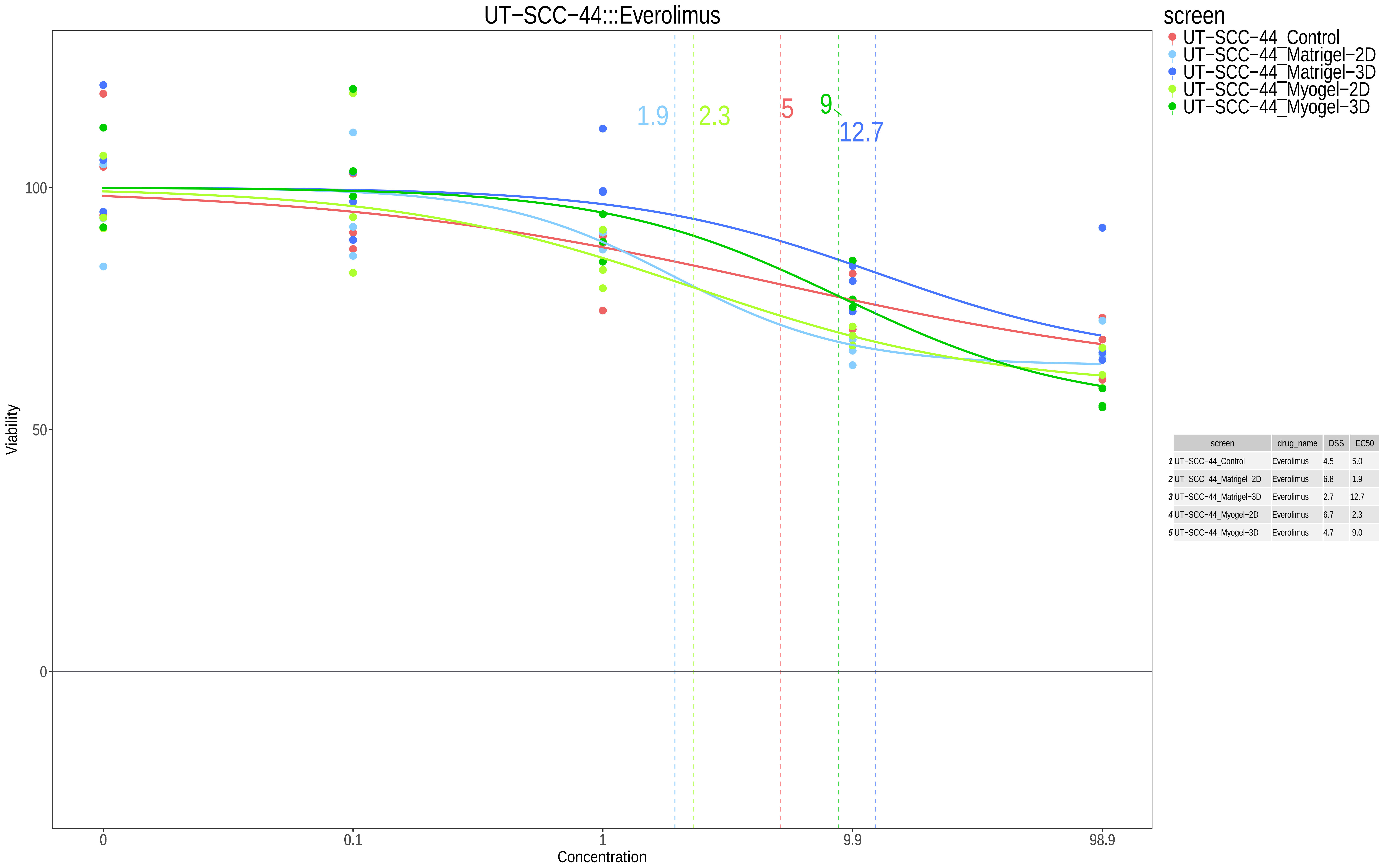

	screen	drug_name	DSS	EC50
1	UT−SCC−44_Control	Everolimus	4.5	5.0
2	UT−SCC−44_Matrigel−2D	Everolimus	6.8	1.9
3	UT−SCC−44_Matrigel−3D	Everolimus	2.7	12.7
4	UT−SCC−44_Myogel−2D	Everolimus	6.7	2.3
5	UT−SCC−44_Myogel−3D	Everolimus	4.7	9.0

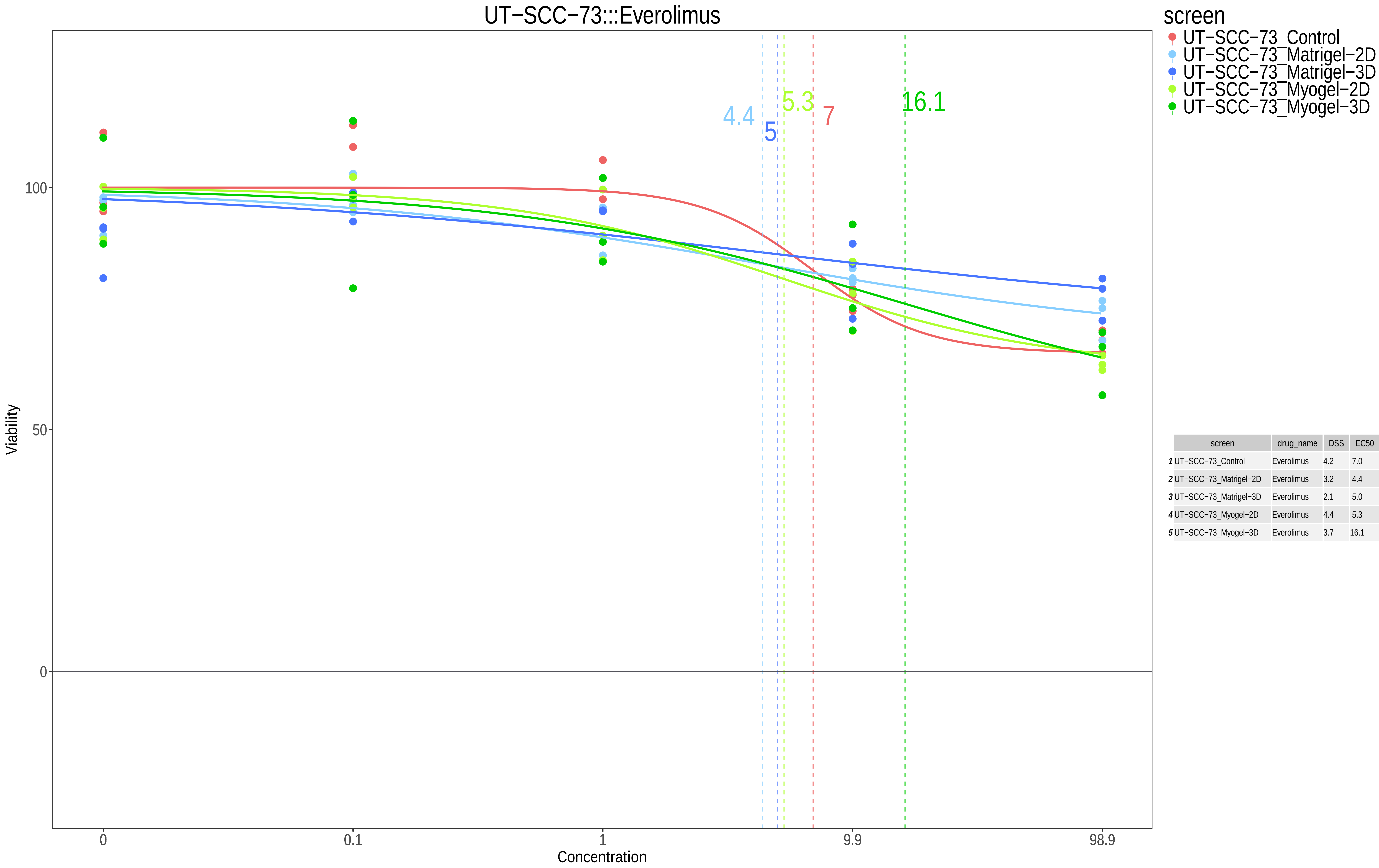

	screen	drug_name	DSS	EC50
1	UT−SCC−73_Control	Everolimus	4.2	7.0
2	UT−SCC−73_Matrigel−2D	Everolimus	3.2	4.4
3	UT−SCC−73_Matrigel−3D	Everolimus	2.1	5.0
4	UT−SCC−73_Myogel−2D	Everolimus	4.4	5.3
5	UT−SCC−73_Myogel−3D	Everolimus	3.7	16.1

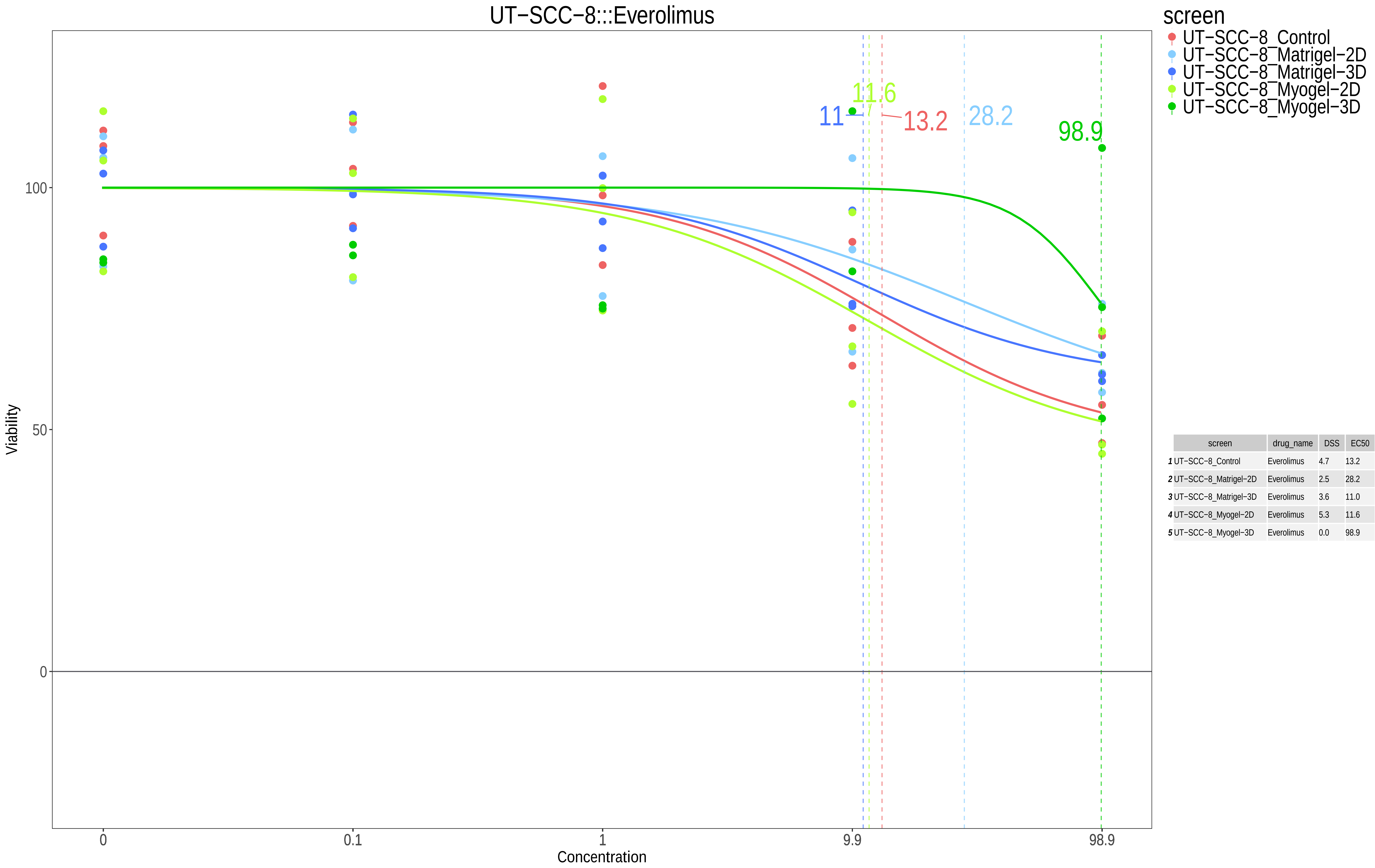

	screen	drug_name	DSS	EC50
1	UT−SCC−8_Control	Everolimus	4.7	13.2
2	UT−SCC−8_Matrigel−2D	Everolimus	2.5	28.2
3	UT−SCC−8_Matrigel−3D	Everolimus	3.6	11.0
4	UT−SCC−8_Myogel−2D	Everolimus	5.3	11.6
5	UT−SCC−8_Myogel−3D	Everolimus	0.0	98.9

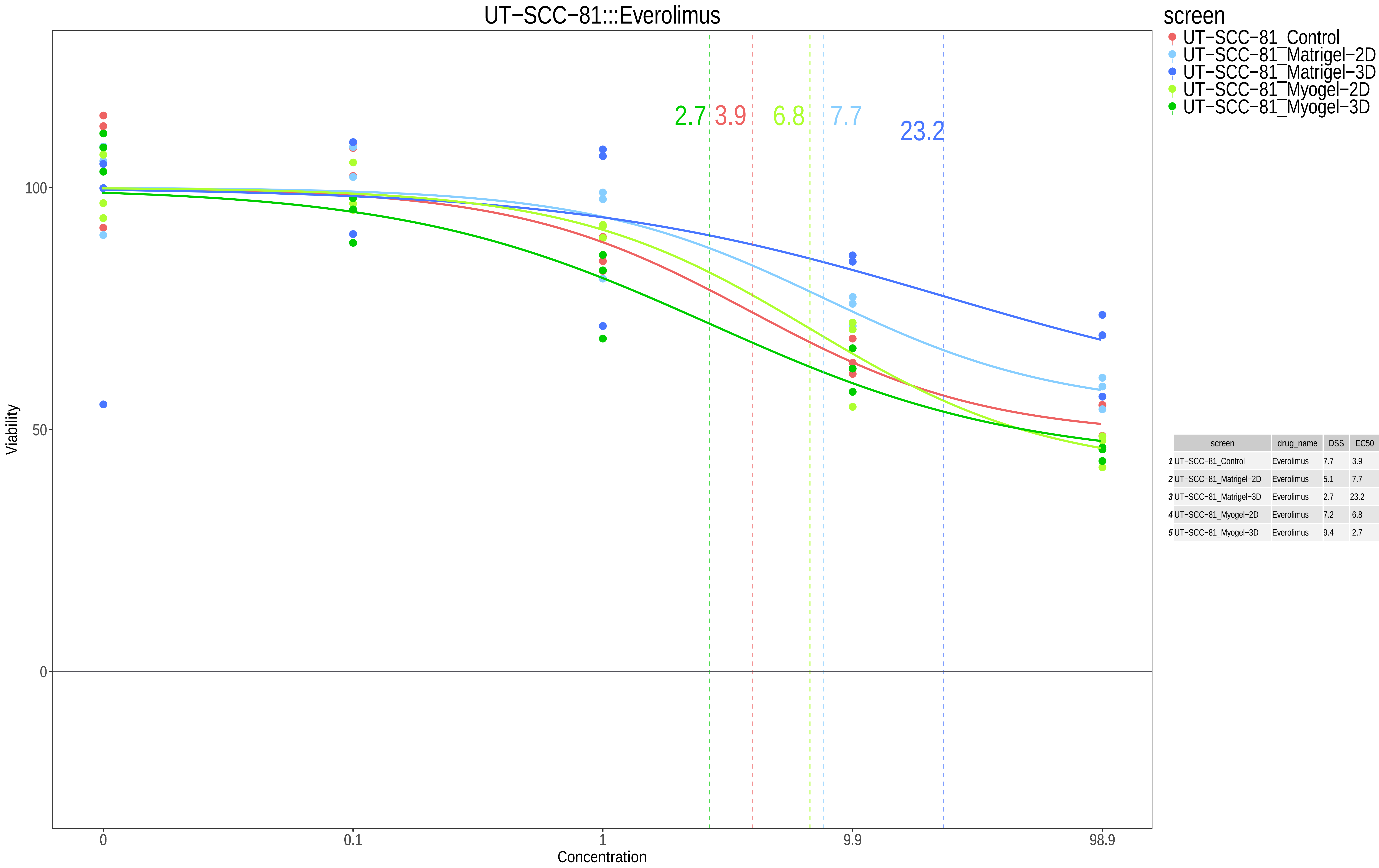

	screen	drug_name	DSS	EC50
1	UT−SCC−81_Control	Everolimus	7.7	3.9
2	UT−SCC−81_Matrigel−2D	Everolimus	5.1	7.7
3	UT−SCC−81_Matrigel−3D	Everolimus	2.7	23.2
4	UT−SCC−81_Myogel−2D	Everolimus	7.2	6.8
5	UT−SCC−81_Myogel−3D	Everolimus	9.4	2.7

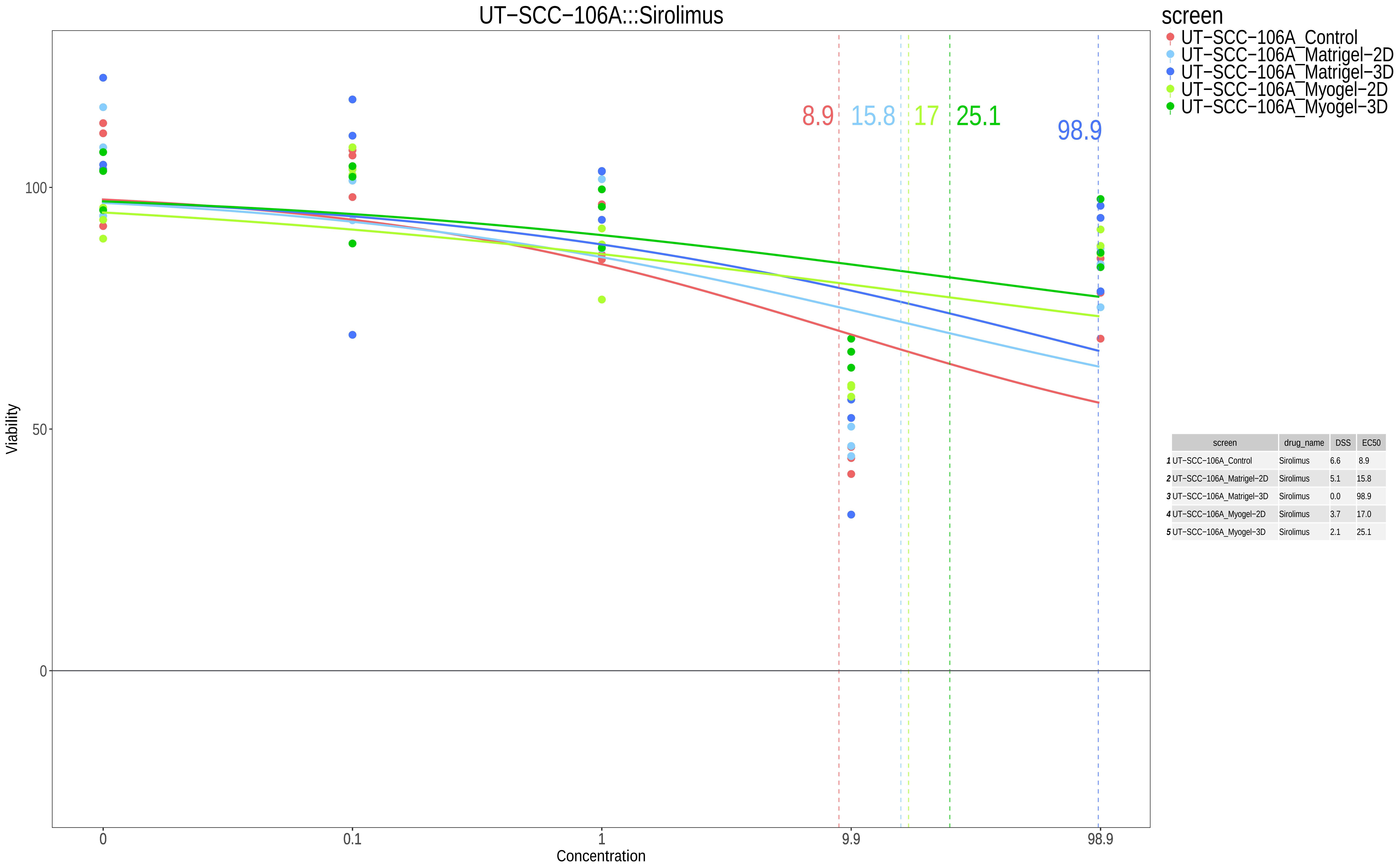

	screen	drug_name	DSS	EC50
1	UT−SCC−106A_Control	Sirolimus	6.6	8.9
2	UT−SCC−106A_Matrigel−2D	Sirolimus	5.1	15.8
3	UT−SCC−106A_Matrigel−3D	Sirolimus	0.0	98.9
4	UT−SCC−106A_Myogel−2D	Sirolimus	3.7	17.0
5	UT−SCC−106A_Myogel−3D	Sirolimus	2.1	25.1

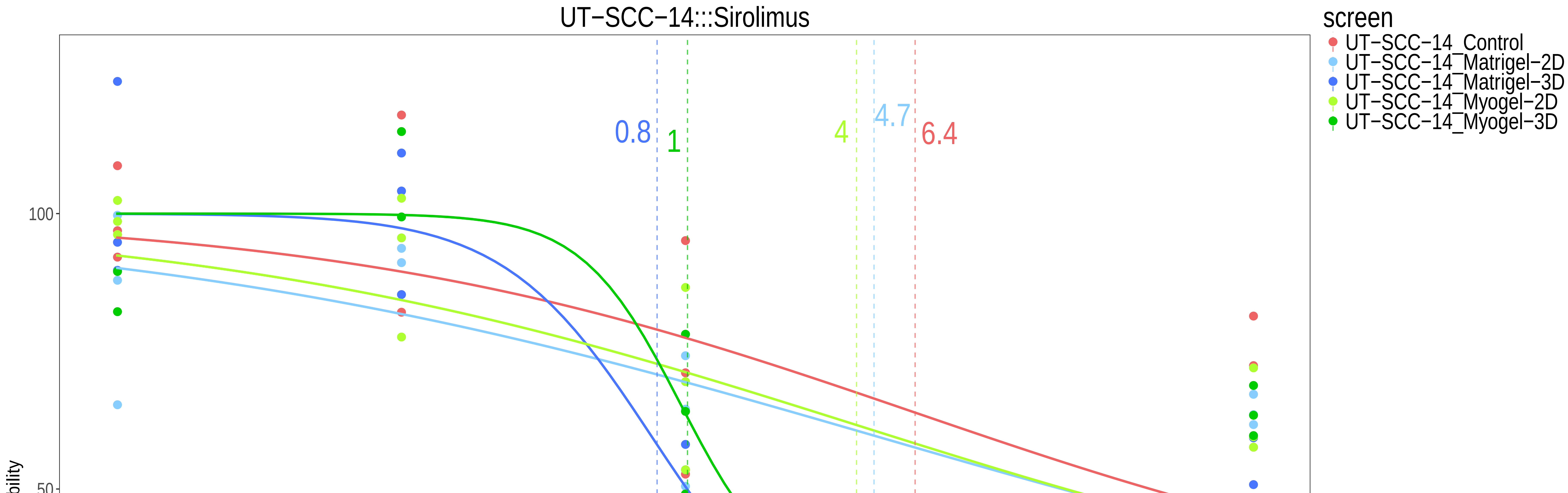

	screen	drug_name	DSS	EC50
1	UT−SCC−14_Control	Sirolimus	9.6	6.4
2	UT−SCC−14_Matrigel−2D	Sirolimus	12.8	4.7
3	UT−SCC−14_Matrigel−3D	Sirolimus	20.7	0.8
4	UT−SCC−14_Myogel−2D	Sirolimus	12.1	4.0
5	UT−SCC−14_Myogel−3D	Sirolimus	17.2	1.0

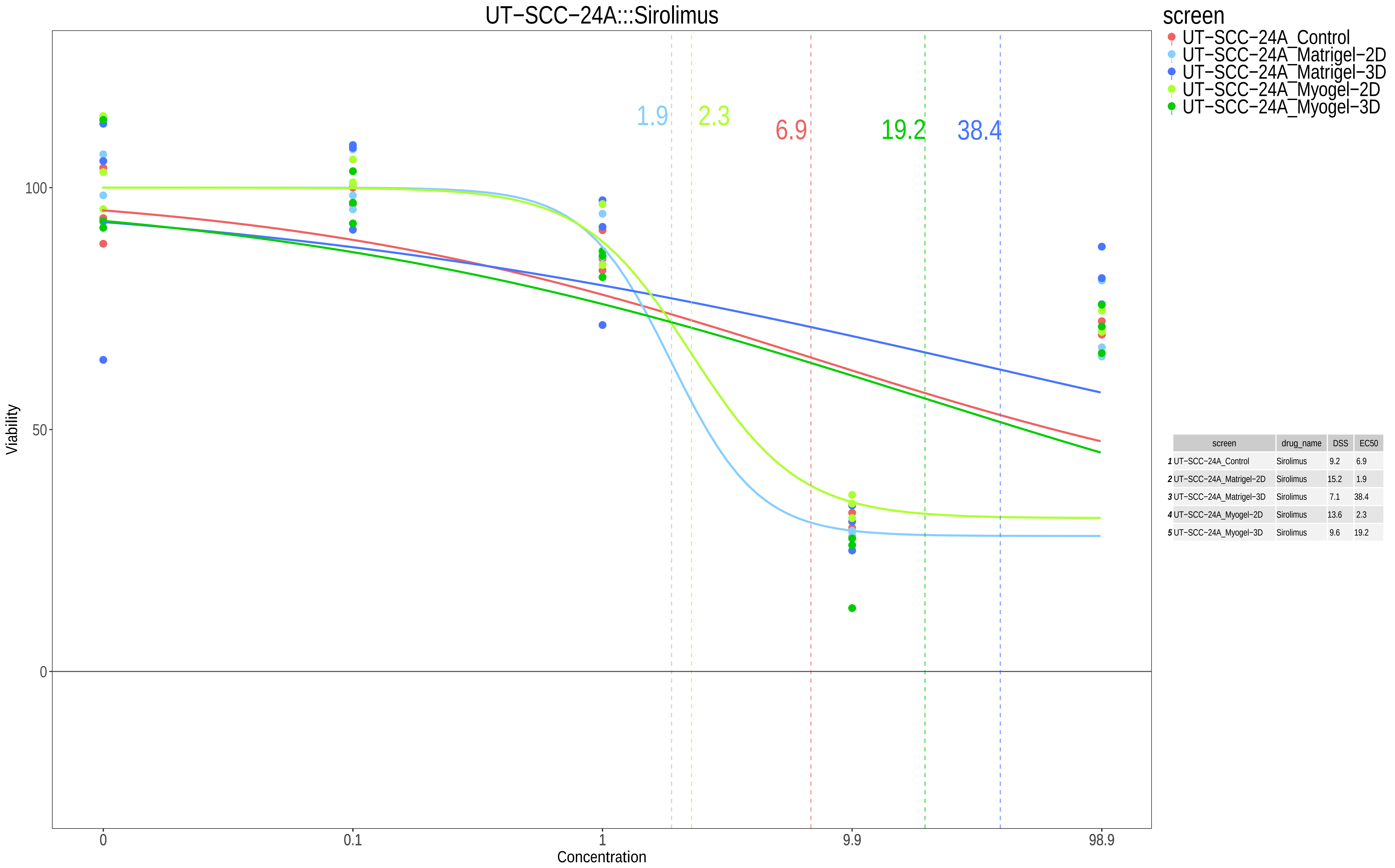

	screen	drug_name	DSS	EC50
1	UT−SCC−24A_Control	Sirolimus	9.2	6.9
2	UT−SCC−24A_Matrigel−2D	Sirolimus	15.2	1.9
3	UT−SCC−24A_Matrigel−3D	Sirolimus	7.1	38.4
4	UT−SCC−24A_Myogel−2D	Sirolimus	13.6	2.3
5	UT−SCC−24A_Myogel−3D	Sirolimus	9.6	19.2

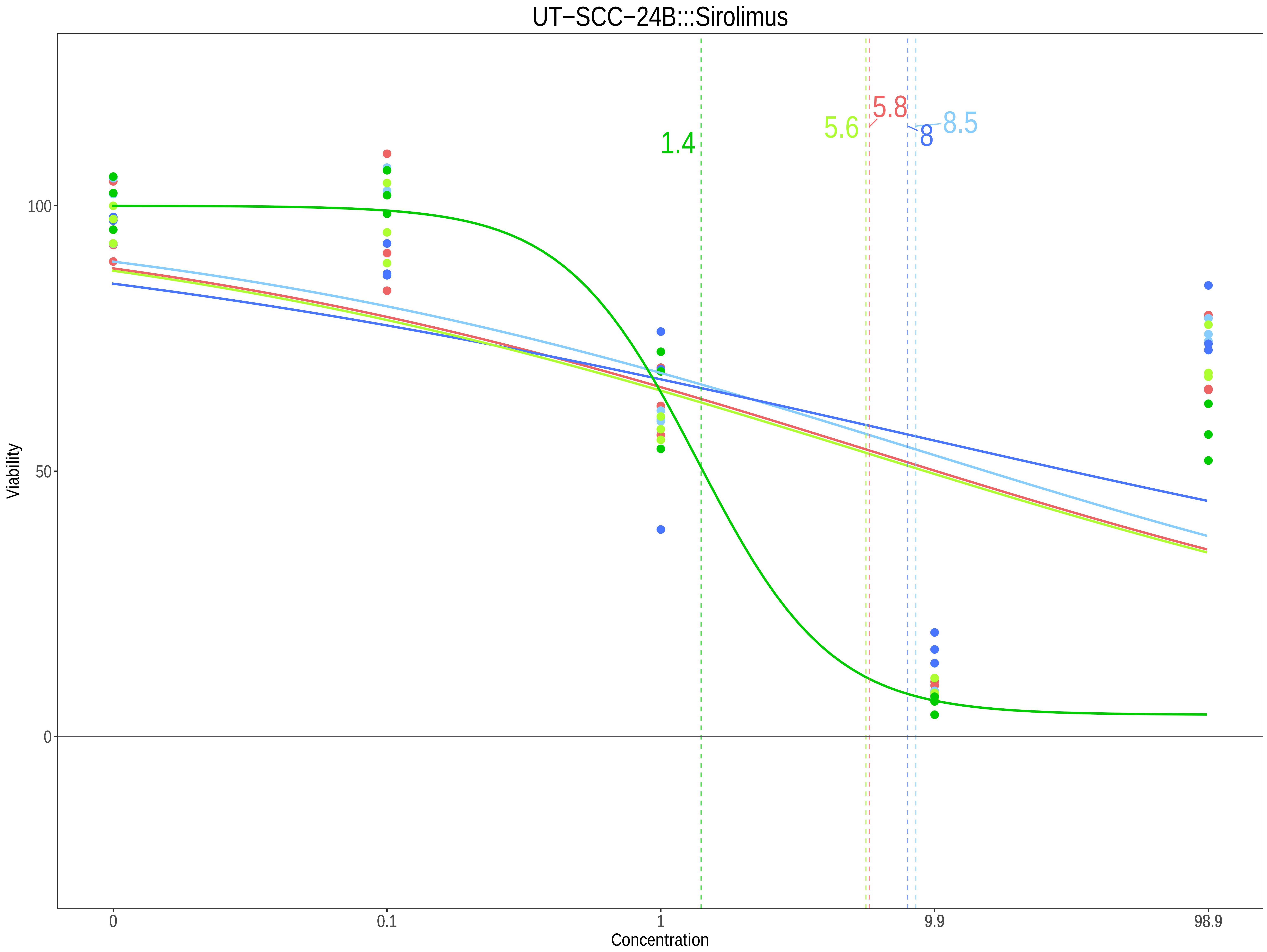

	screen	drug_name	DSS	EC50
1	UT−SCC−24B_Control	Sirolimus	14.5	5.8
2	UT−SCC−24B_Matrigel−2D	Sirolimus	13.2	8.5
3	UT−SCC−24B_Matrigel−3D	Sirolimus	13.5	8.0
4	UT−SCC−24B_Myogel−2D	Sirolimus	14.8	5.6
5	UT−SCC−24B_Myogel−3D	Sirolimus	21.3	1.4

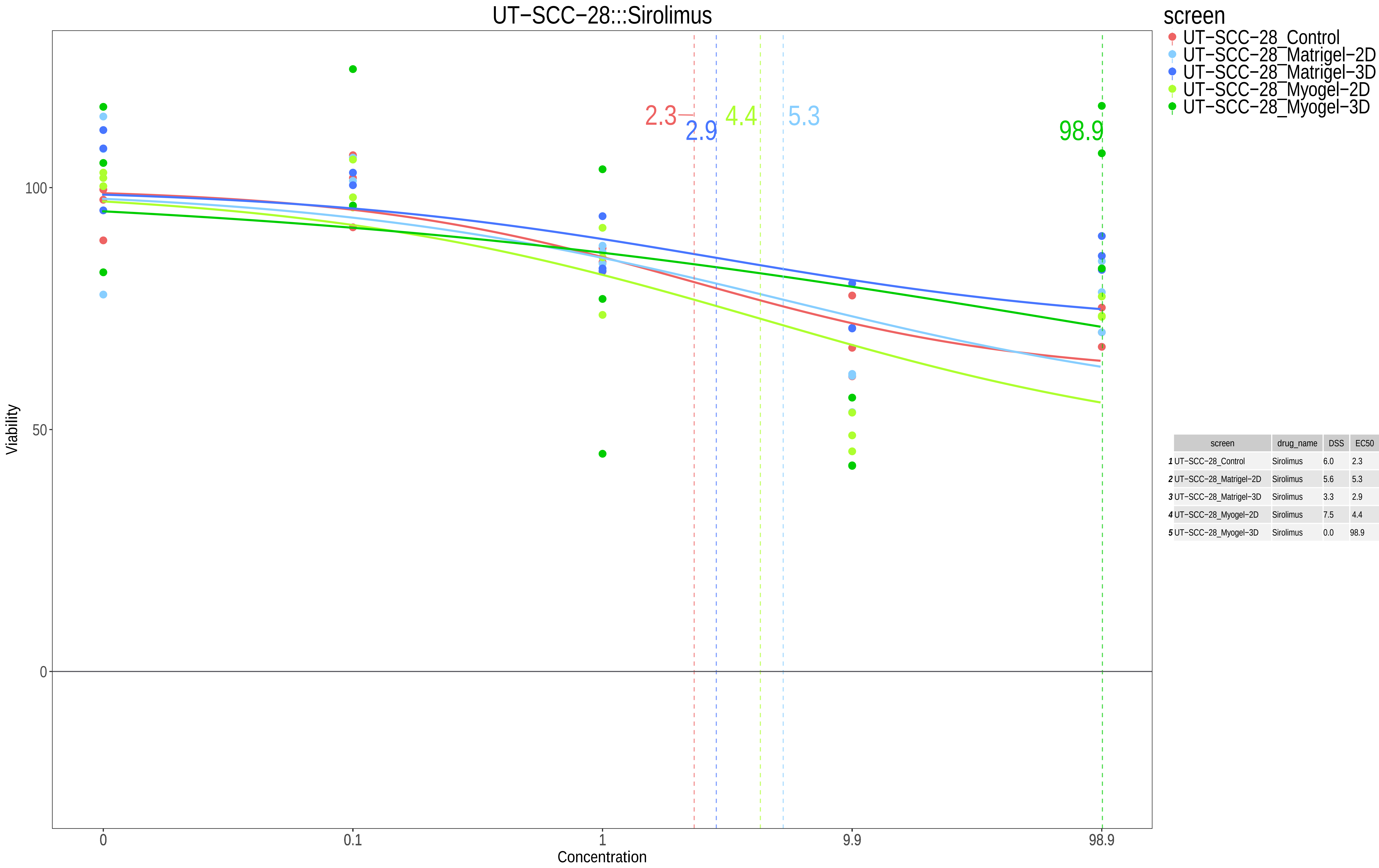

	screen	drug_name	DSS	EC50
1	UT−SCC−28_Control	Sirolimus	6.0	2.3
2	UT−SCC−28_Matrigel−2D	Sirolimus	5.6	5.3
3	UT−SCC−28_Matrigel−3D	Sirolimus	3.3	2.9
4	UT−SCC−28_Myogel−2D	Sirolimus	7.5	4.4
5	UT−SCC−28_Myogel−3D	Sirolimus	0.0	98.9

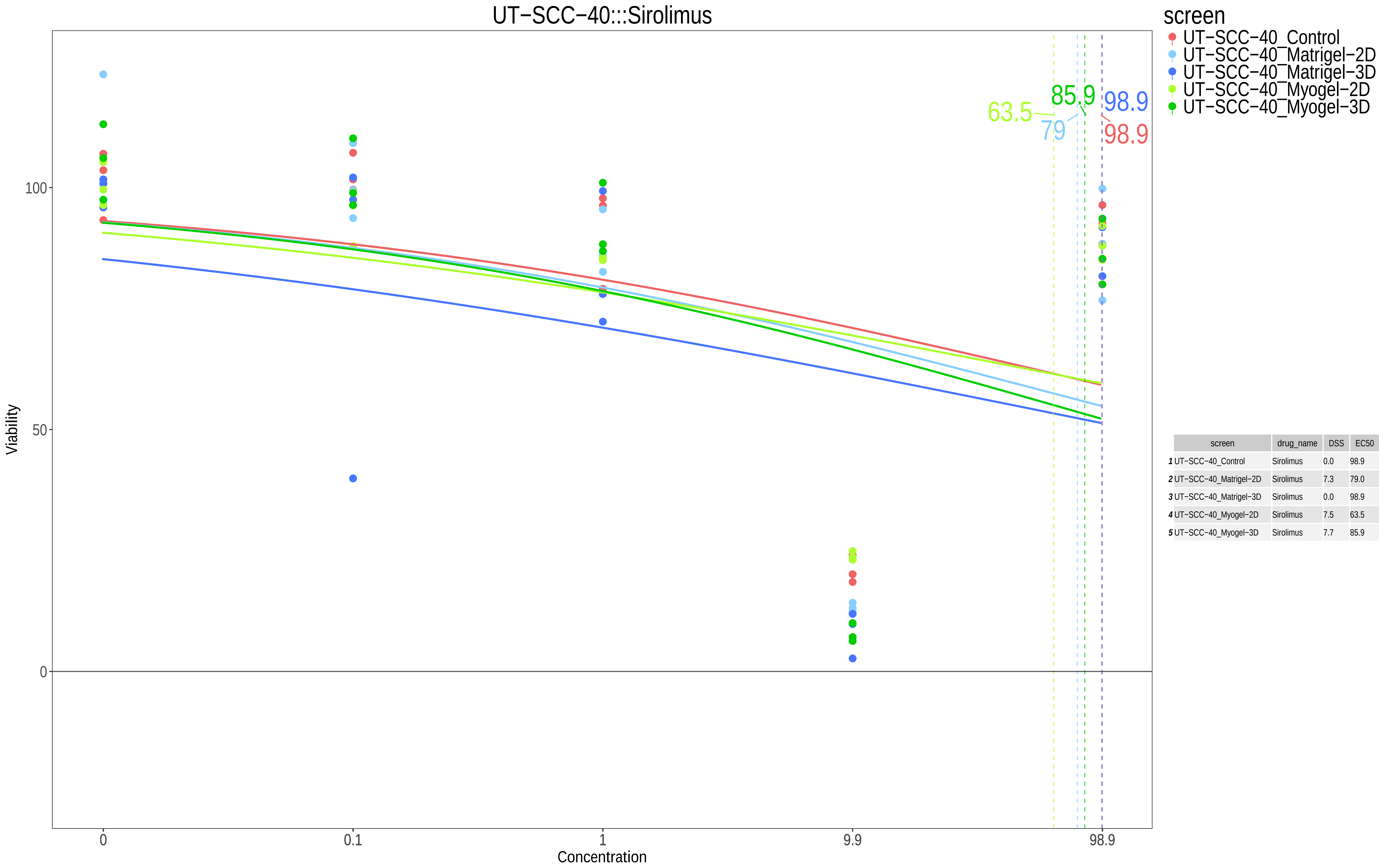

	screen	drug_name	DSS	EC50
1	UT−SCC−40_Control	Sirolimus	0.0	98.9
2	UT−SCC−40_Matrigel−2D	Sirolimus	7.3	79.0
3	UT−SCC−40_Matrigel−3D	Sirolimus	0.0	98.9
4	UT−SCC−40_Myogel−2D	Sirolimus	7.5	63.5
5	UT−SCC−40_Myogel−3D	Sirolimus	7.7	85.9

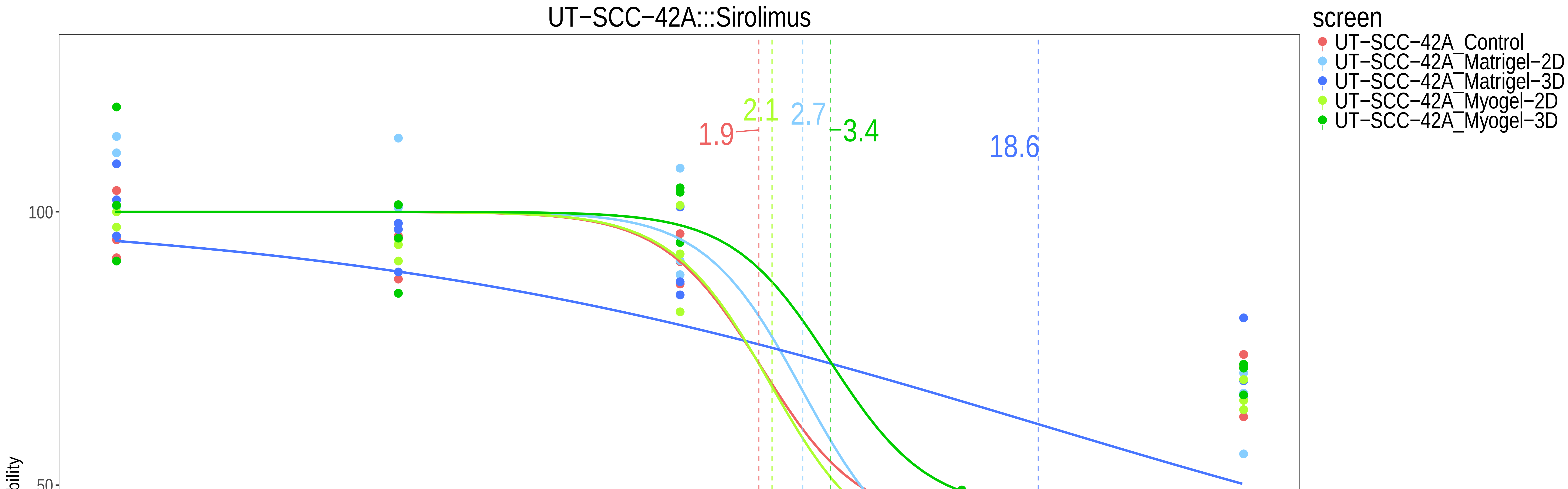

	screen	drug_name	DSS	EC50
1	UT−SCC−42A_Control	Sirolimus	11.9	1.9
2	UT−SCC−42A_Matrigel−2D	Sirolimus	12.5	2.7
3	UT−SCC−42A_Matrigel−3D	Sirolimus	8.1	18.6
4	UT−SCC−42A_Myogel−2D	Sirolimus	12.9	2.1
5	UT−SCC−42A_Myogel−3D	Sirolimus	9.7	3.4

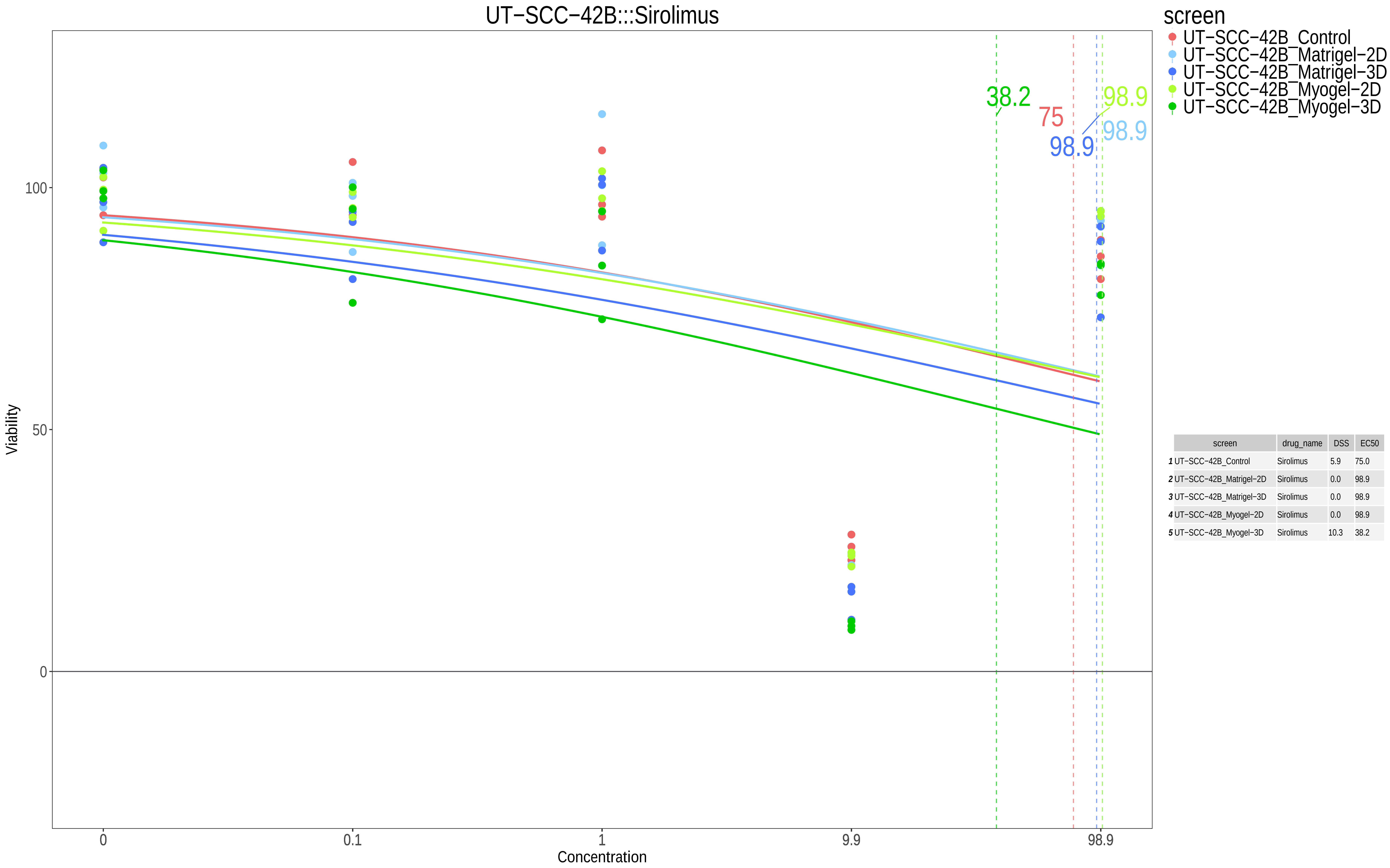

	screen	drug_name	DSS	EC50
1	UT−SCC−42B_Control	Sirolimus	5.9	75.0
2	UT−SCC−42B_Matrigel−2D	Sirolimus	0.0	98.9
3	UT−SCC−42B_Matrigel−3D	Sirolimus	0.0	98.9
4	UT−SCC−42B_Myogel−2D	Sirolimus	0.0	98.9
5	UT−SCC−42B_Myogel−3D	Sirolimus	10.3	38.2

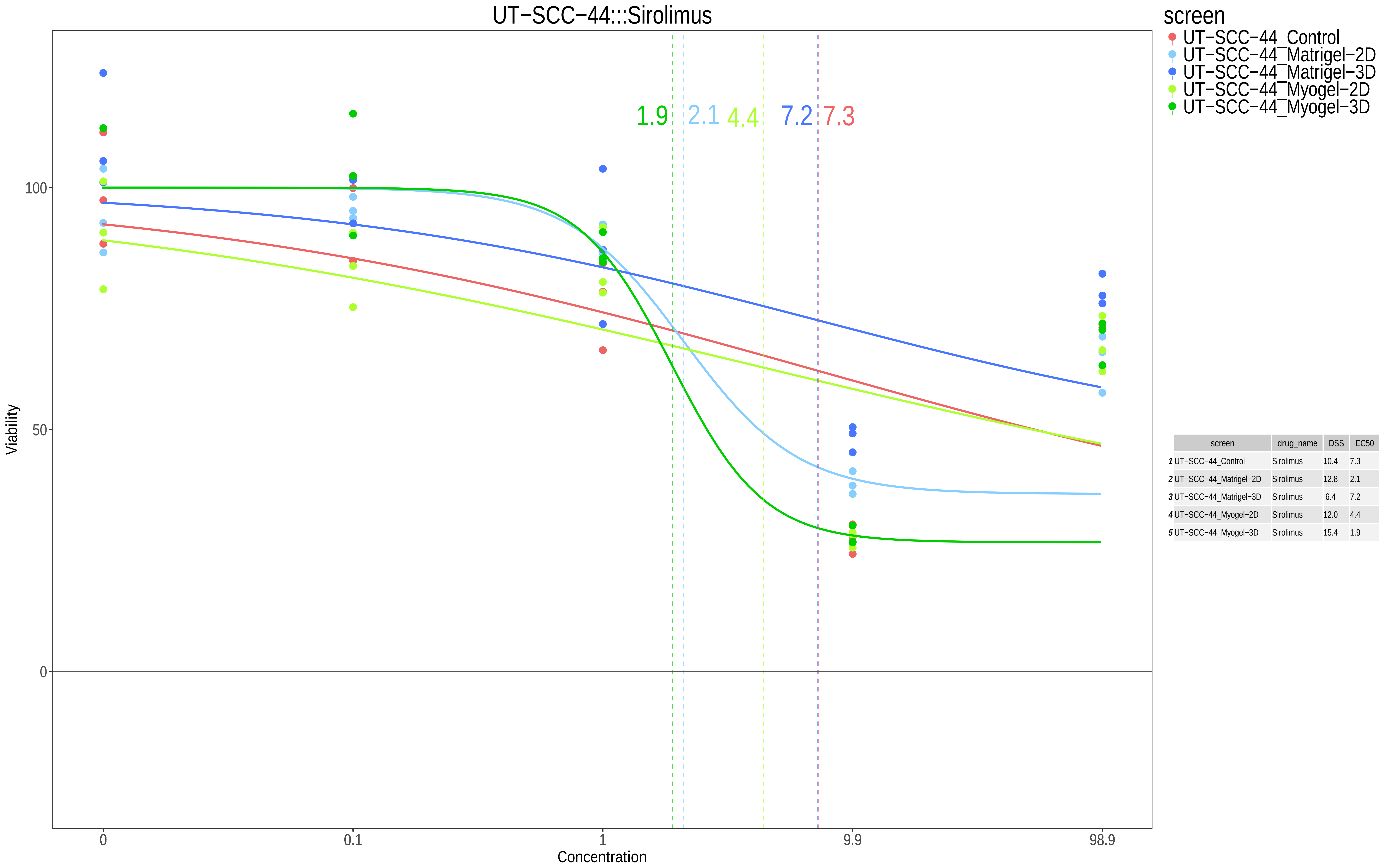

	screen	drug_name	DSS	EC50
1	UT−SCC−44_Control	Sirolimus	10.4	7.3
2	UT−SCC−44_Matrigel−2D	Sirolimus	12.8	2.1
3	UT−SCC−44_Matrigel−3D	Sirolimus	6.4	7.2
4	UT−SCC−44_Myogel−2D	Sirolimus	12.0	4.4
5	UT−SCC−44_Myogel−3D	Sirolimus	15.4	1.9

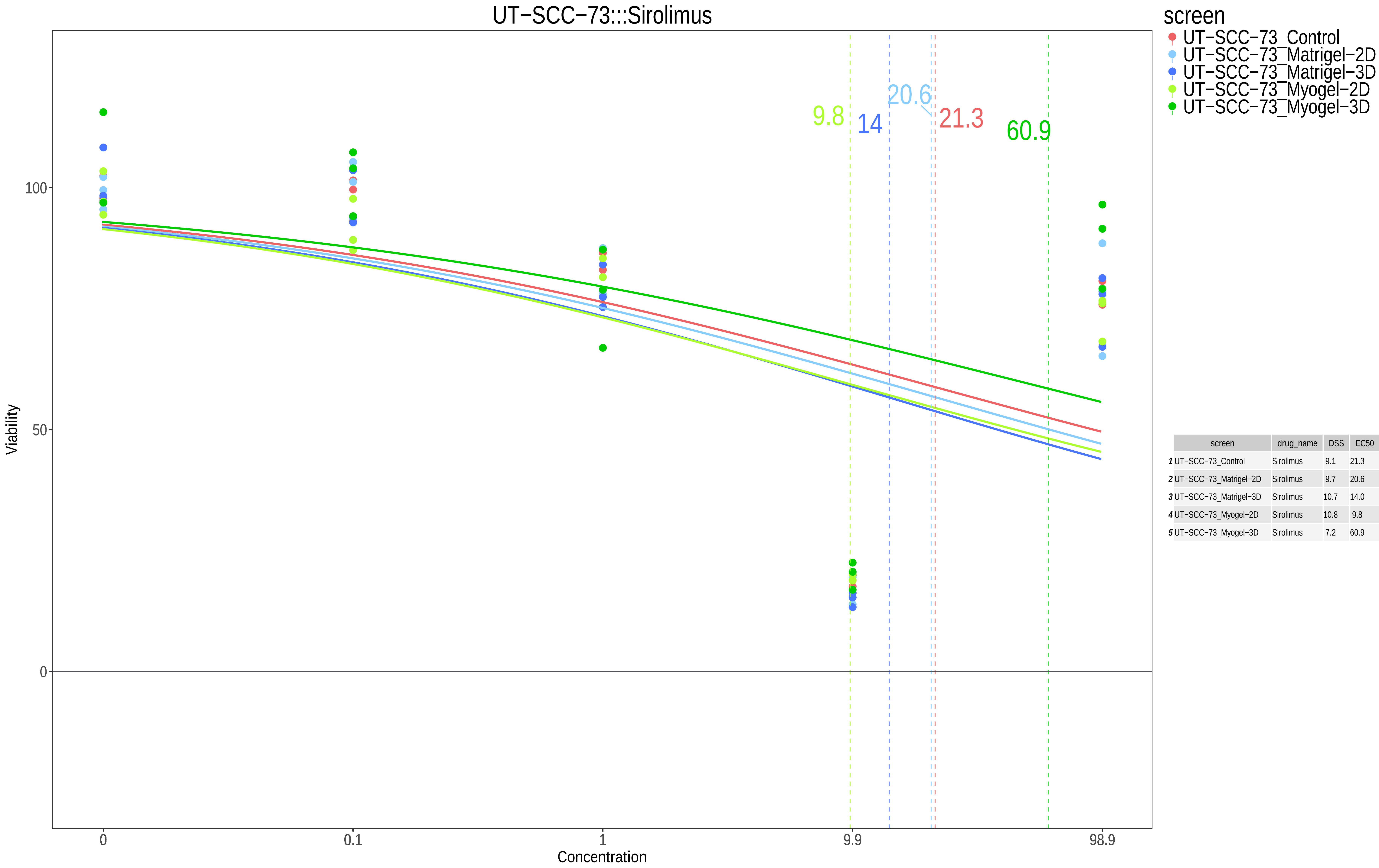

	screen	drug_name	DSS	EC50
1	UT−SCC−73_Control	Sirolimus	9.1	21.3
2	UT−SCC−73_Matrigel−2D	Sirolimus	9.7	20.6
3	UT−SCC−73_Matrigel−3D	Sirolimus	10.7	14.0
4	UT−SCC−73_Myogel−2D	Sirolimus	10.8	9.8
5	UT−SCC−73_Myogel−3D	Sirolimus	7.2	60.9

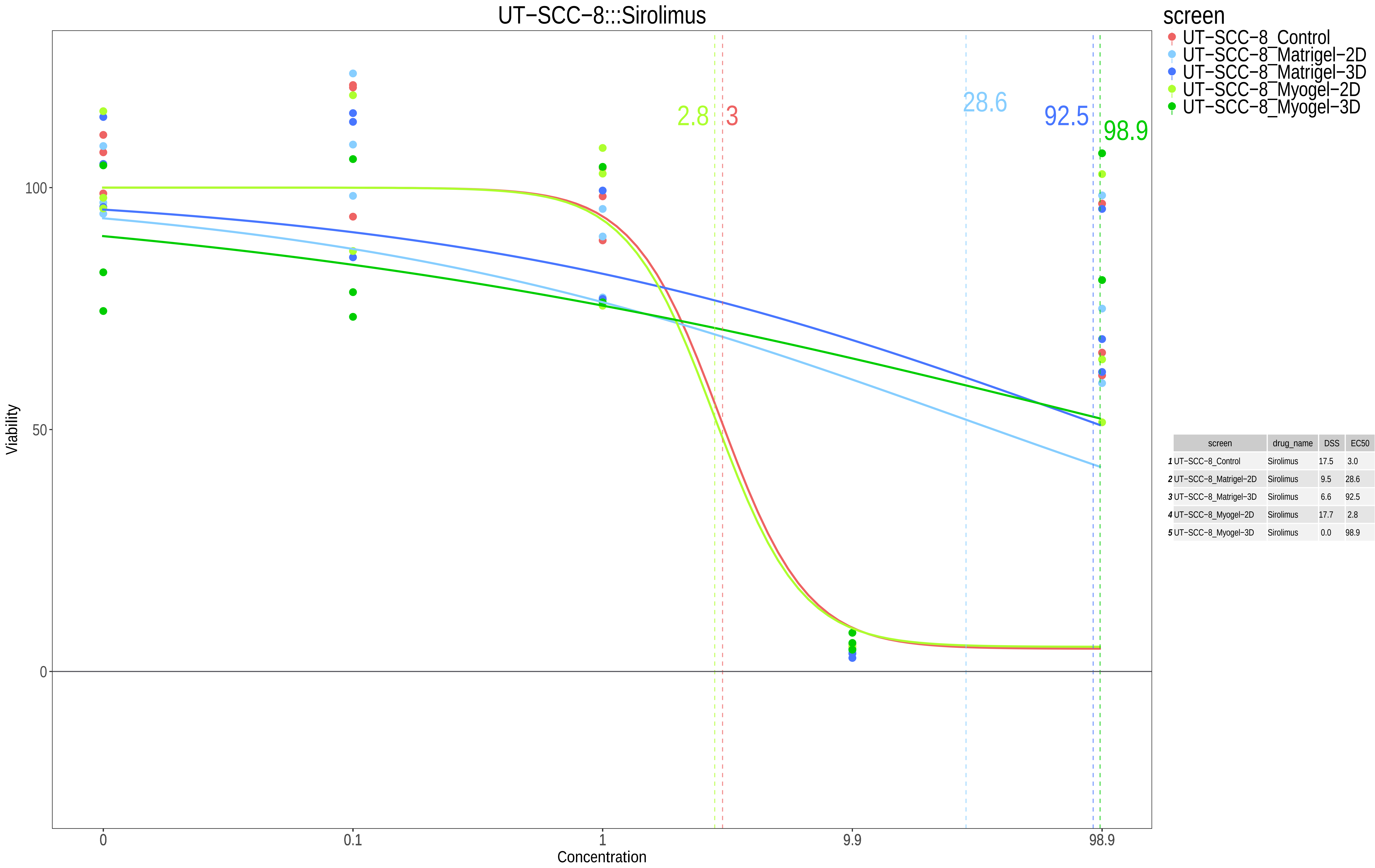

	screen	drug_name	DSS	EC50
1	UT−SCC−8_Control	Sirolimus	17.5	3.0
2	UT−SCC−8_Matrigel−2D	Sirolimus	9.5	28.6
3	UT−SCC−8_Matrigel−3D	Sirolimus	6.6	92.5
4	UT−SCC−8_Myogel−2D	Sirolimus	17.7	2.8
5	UT−SCC−8_Myogel−3D	Sirolimus	0.0	98.9

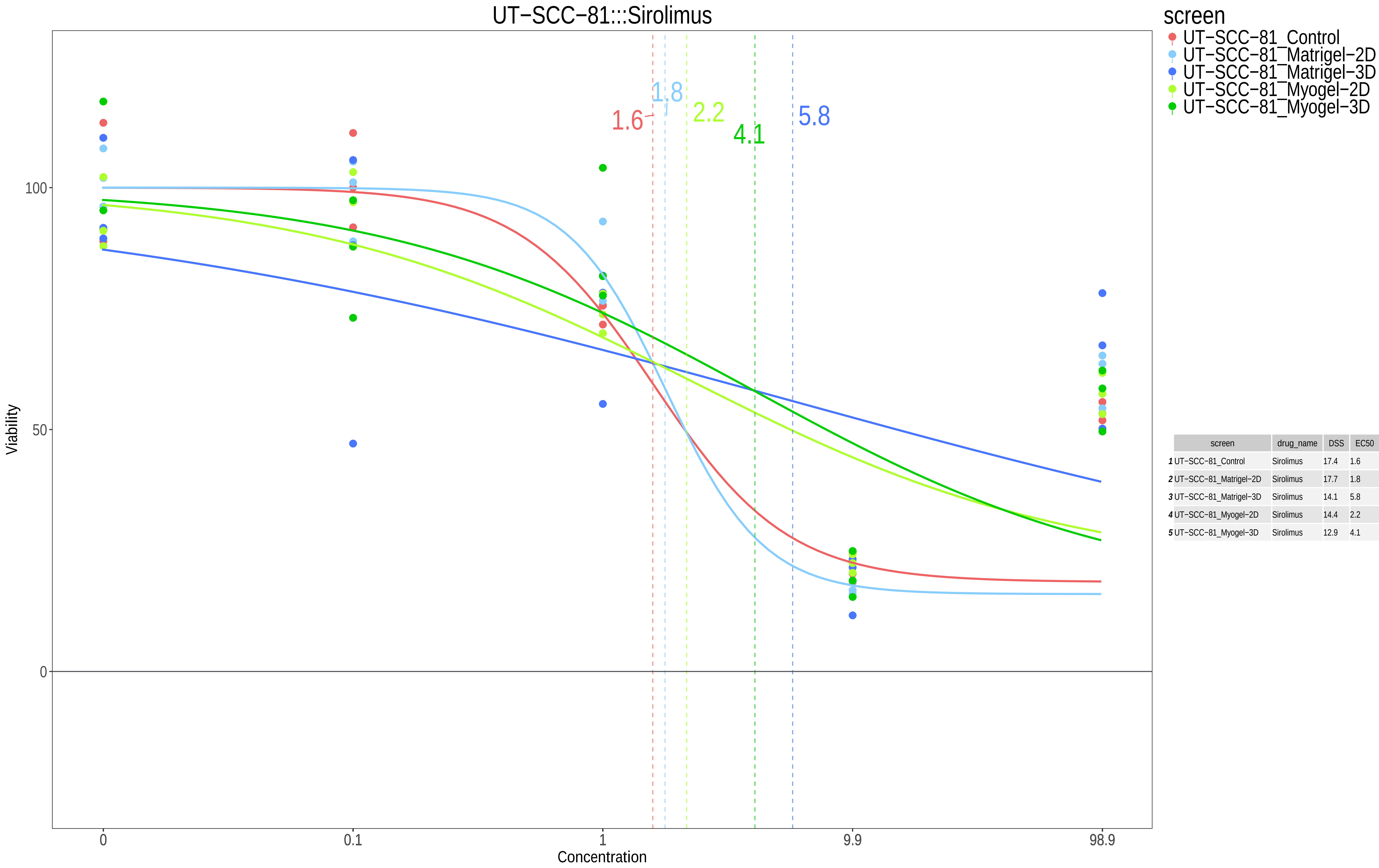

	screen	drug_name	DSS	EC50
1	UT−SCC−81_Control	Sirolimus	17.4	1.6
2	UT−SCC−81_Matrigel−2D	Sirolimus	17.7	1.8
3	UT−SCC−81_Matrigel−3D	Sirolimus	14.1	5.8
4	UT−SCC−81_Myogel−2D	Sirolimus	14.4	2.2
5	UT−SCC−81_Myogel−3D	Sirolimus	12.9	4.1

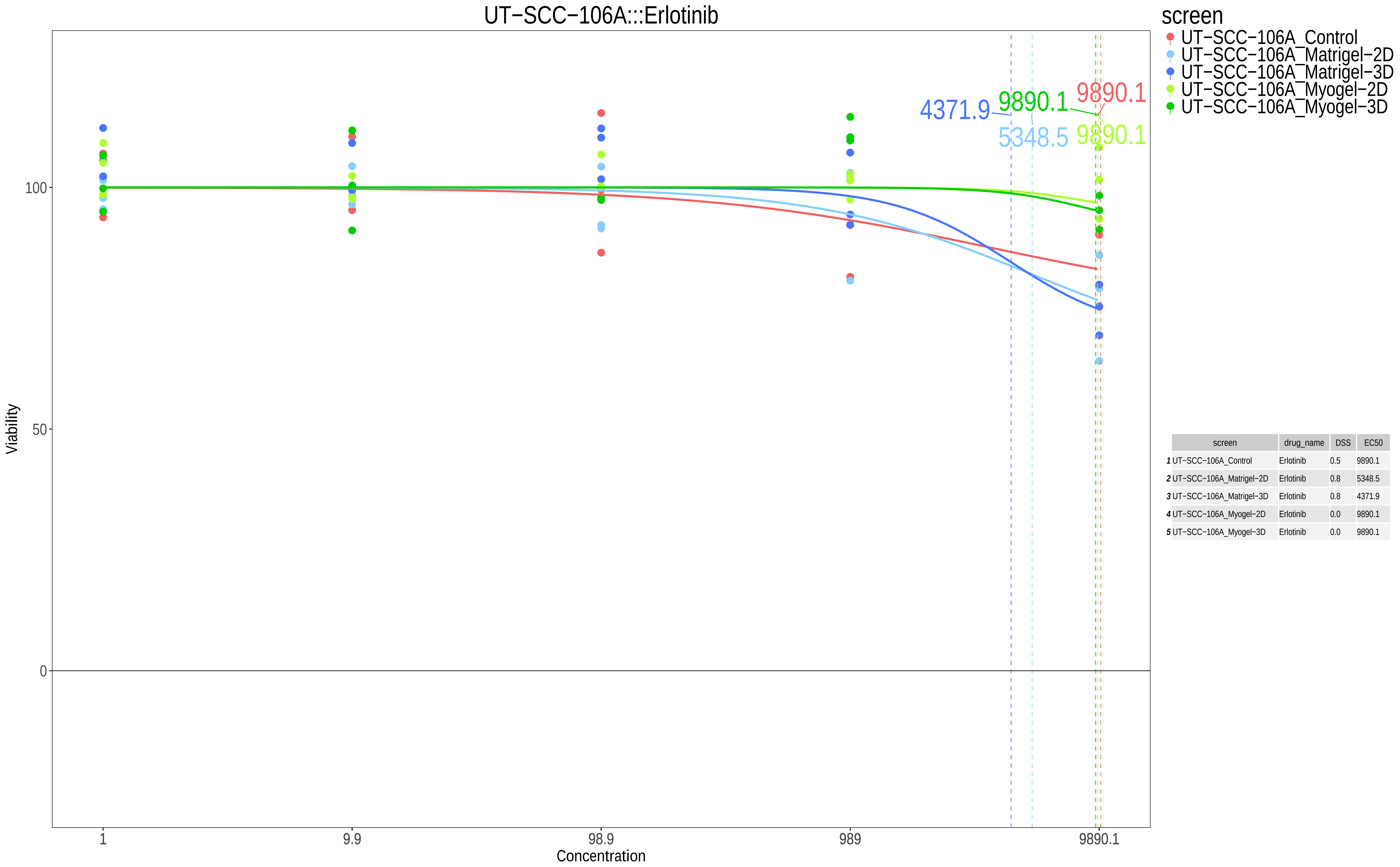

	screen	drug_name	DSS	EC50
1	UT−SCC−106A_Control	Erlotinib	0.5	9890.1
2	UT−SCC−106A_Matrigel−2D	Erlotinib	0.8	5348.5
3	UT−SCC−106A_Matrigel−3D	Erlotinib	0.8	4371.9
4	UT−SCC−106A_Myogel−2D	Erlotinib	0.0	9890.1
5	UT−SCC−106A_Myogel−3D	Erlotinib	0.0	9890.1

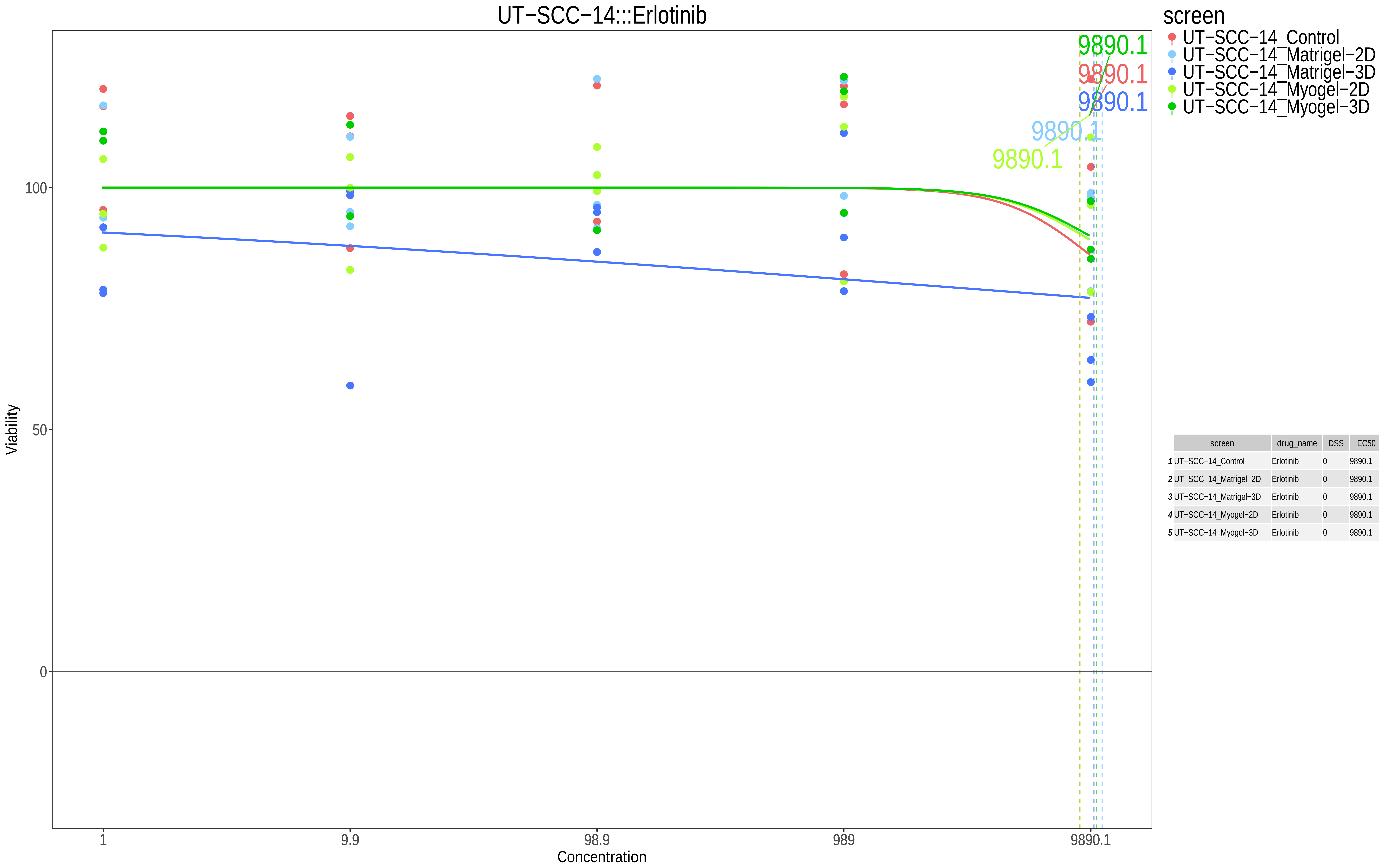

	screen	drug_name	DSS	EC50
1	UT−SCC−14_Control	Erlotinib	0	9890.1
2	UT−SCC−14_Matrigel−2D	Erlotinib	0	9890.1
3	UT−SCC−14_Matrigel−3D	Erlotinib	0	9890.1
4	UT−SCC−14_Myogel−2D	Erlotinib	0	9890.1
5	UT−SCC−14_Myogel−3D	Erlotinib	0	9890.1

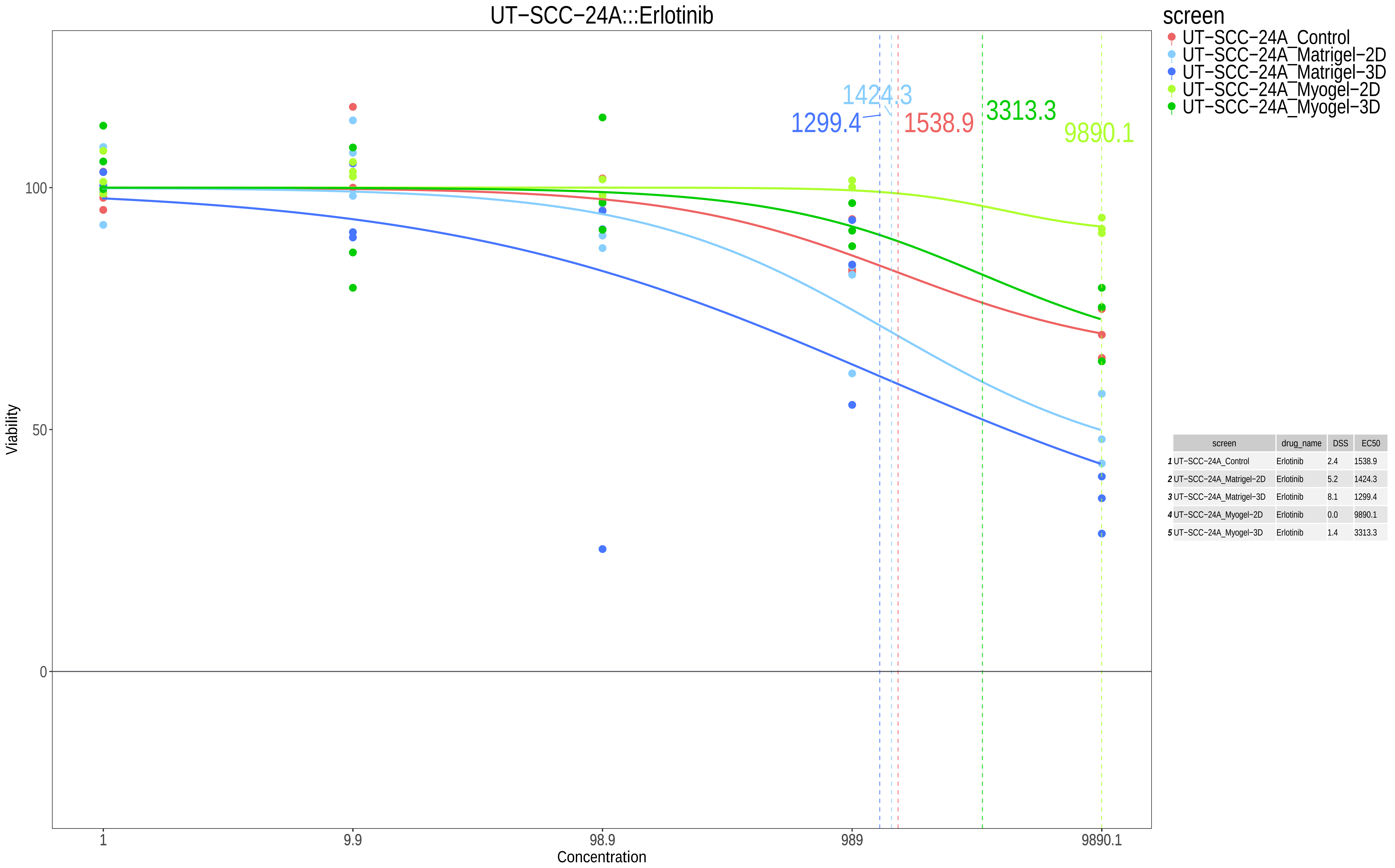

	screen	drug_name	DSS	EC50
1	UT−SCC−24A_Control	Erlotinib	2.4	1538.9
2	UT−SCC−24A_Matrigel−2D	Erlotinib	5.2	1424.3
3	UT−SCC−24A_Matrigel−3D	Erlotinib	8.1	1299.4
4	UT−SCC−24A_Myogel−2D	Erlotinib	0.0	9890.1
5	UT−SCC−24A_Myogel−3D	Erlotinib	1.4	3313.3

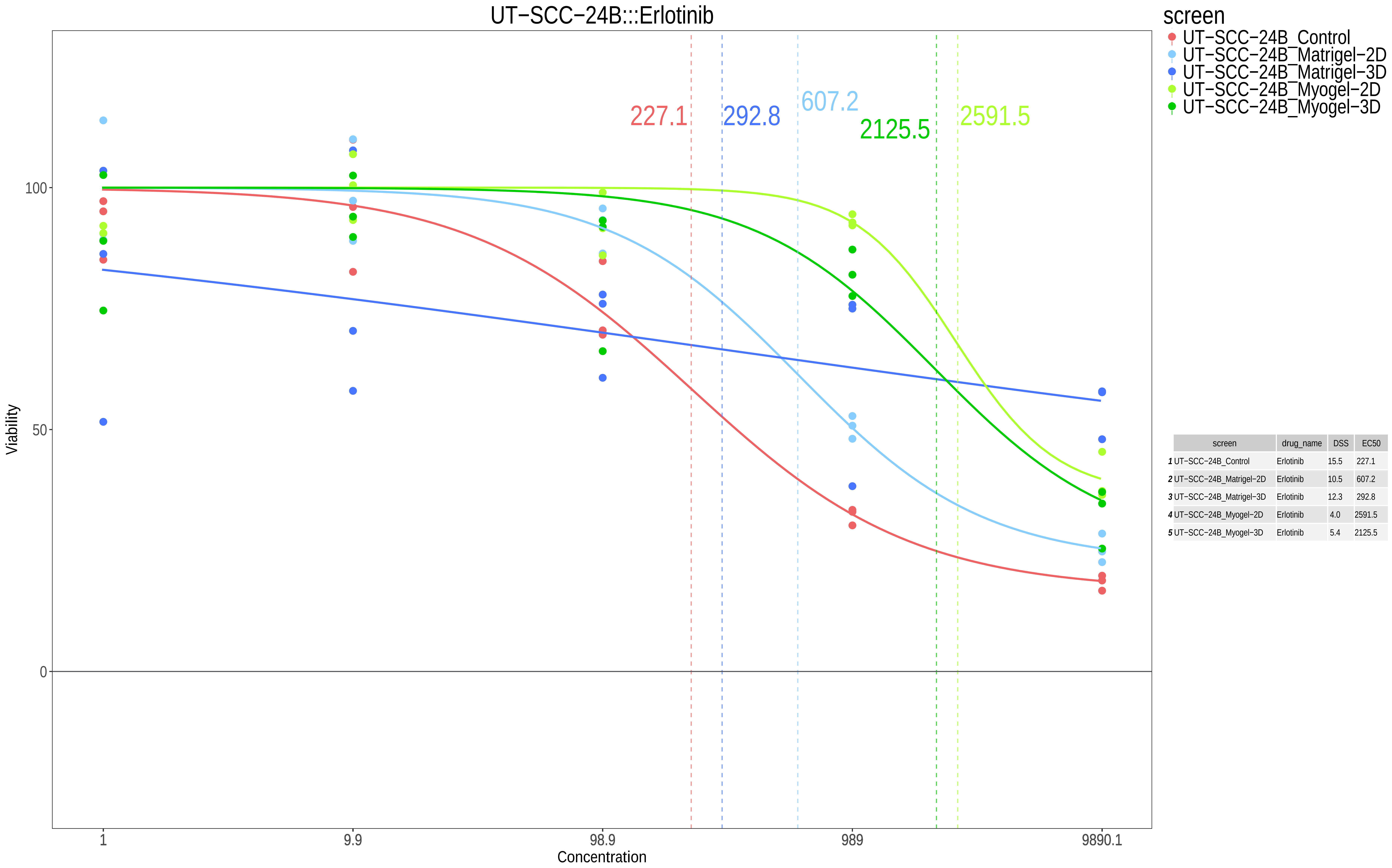

	screen	drug_name	DSS	EC50
1	UT−SCC−24B_Control	Erlotinib	15.5	227.1
2	UT−SCC−24B_Matrigel−2D	Erlotinib	10.5	607.2
3	UT−SCC−24B_Matrigel−3D	Erlotinib	12.3	292.8
4	UT−SCC−24B_Myogel−2D	Erlotinib	4.0	2591.5
5	UT−SCC−24B_Myogel−3D	Erlotinib	5.4	2125.5

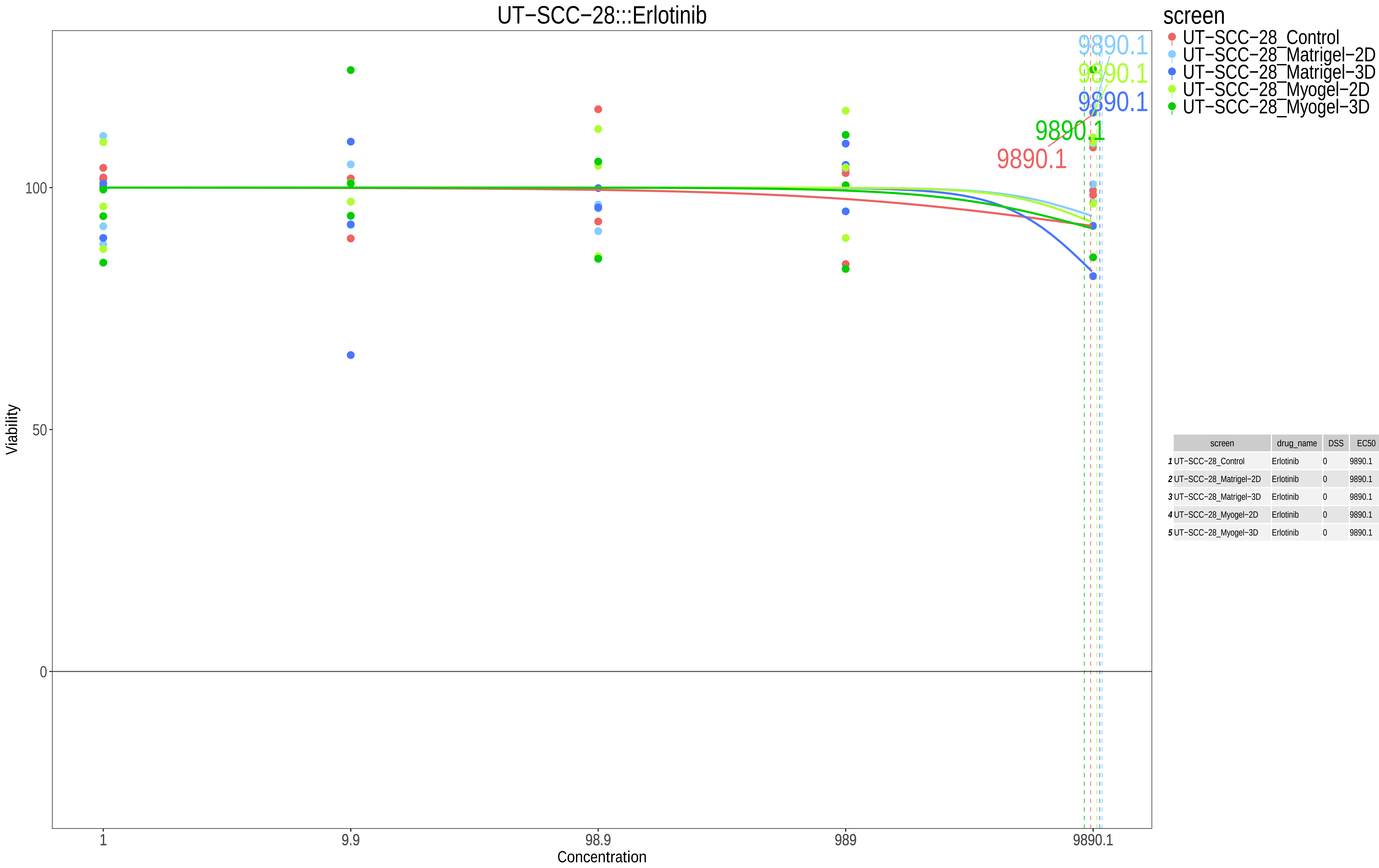

	screen	drug_name	DSS	EC50
1	UT−SCC−28_Control	Erlotinib	0	9890.1
2	UT−SCC−28_Matrigel−2D	Erlotinib	0	9890.1
3	UT−SCC−28_Matrigel−3D	Erlotinib	0	9890.1
4	UT−SCC−28_Myogel−2D	Erlotinib	0	9890.1
5	UT−SCC−28_Myogel−3D	Erlotinib	0	9890.1

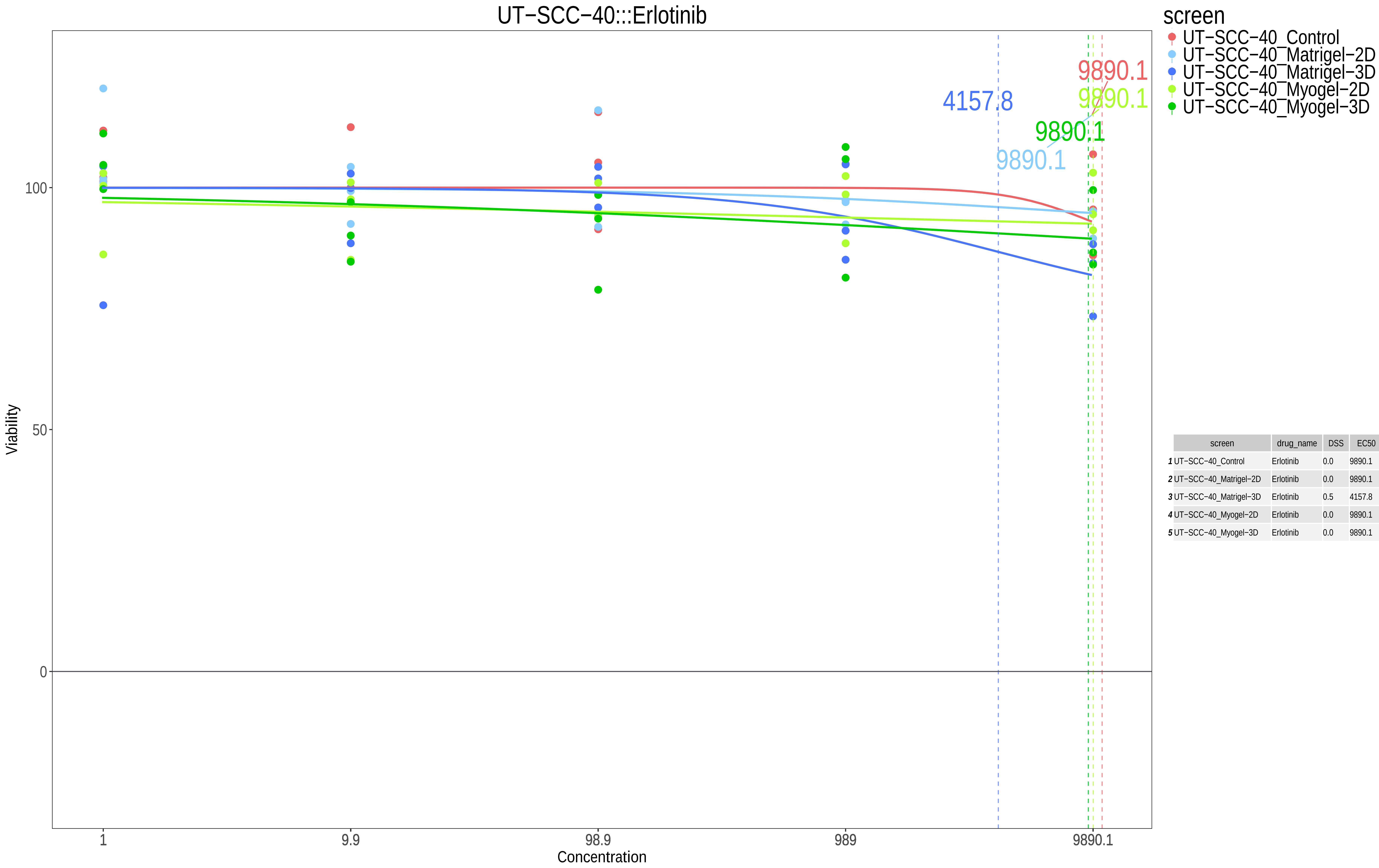

	screen	drug_name	DSS	EC50
1	UT−SCC−40_Control	Erlotinib	0.0	9890.1
2	UT−SCC−40_Matrigel−2D	Erlotinib	0.0	9890.1
3	UT−SCC−40_Matrigel−3D	Erlotinib	0.5	4157.8
4	UT−SCC−40_Myogel−2D	Erlotinib	0.0	9890.1
5	UT−SCC−40_Myogel−3D	Erlotinib	0.0	9890.1

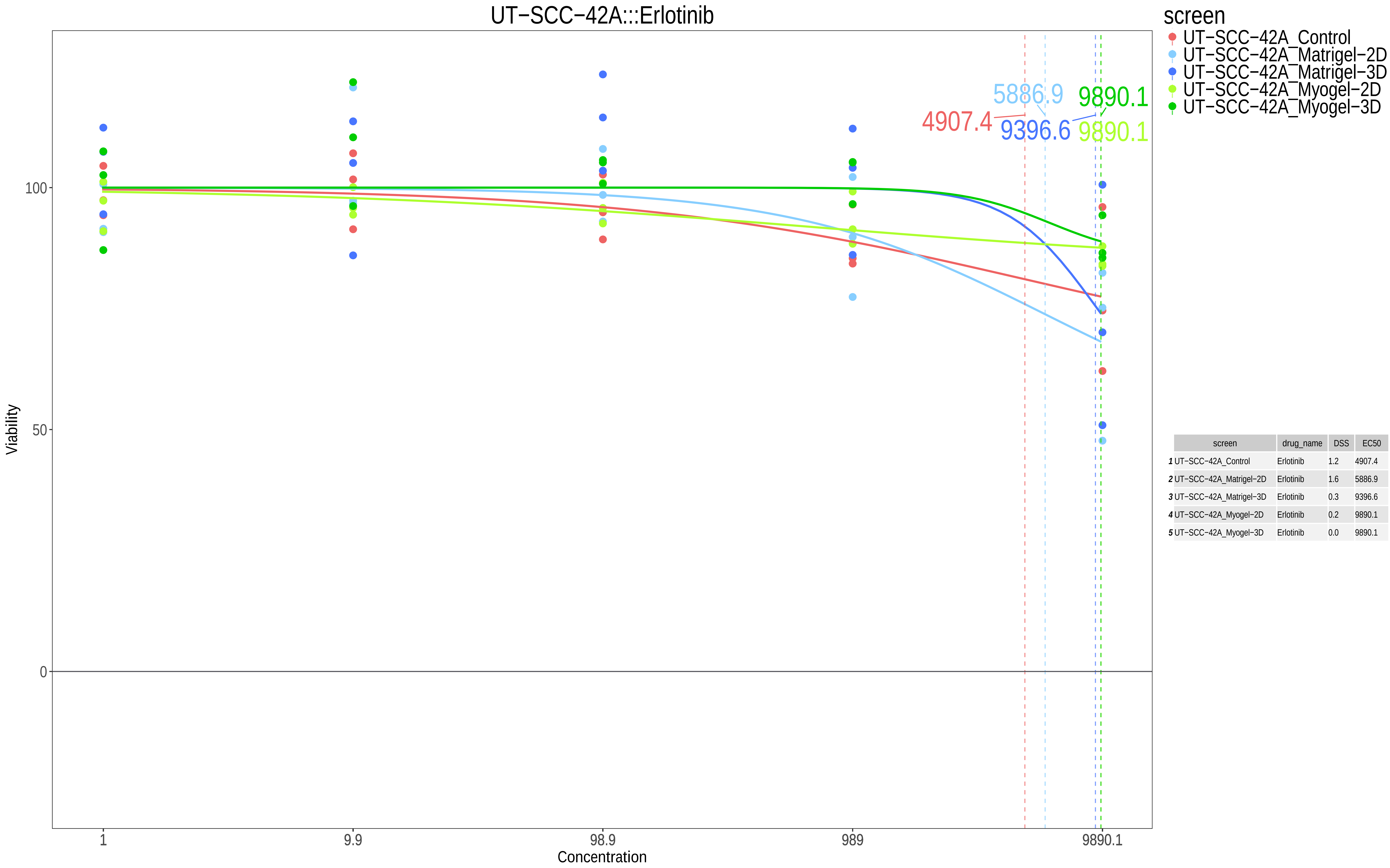

	screen	drug_name	DSS	EC50
1	UT−SCC−42A_Control	Erlotinib	1.2	4907.4
2	UT−SCC−42A_Matrigel−2D	Erlotinib	1.6	5886.9
3	UT−SCC−42A_Matrigel−3D	Erlotinib	0.3	9396.6
4	UT−SCC−42A_Myogel−2D	Erlotinib	0.2	9890.1
5	UT−SCC−42A_Myogel−3D	Erlotinib	0.0	9890.1

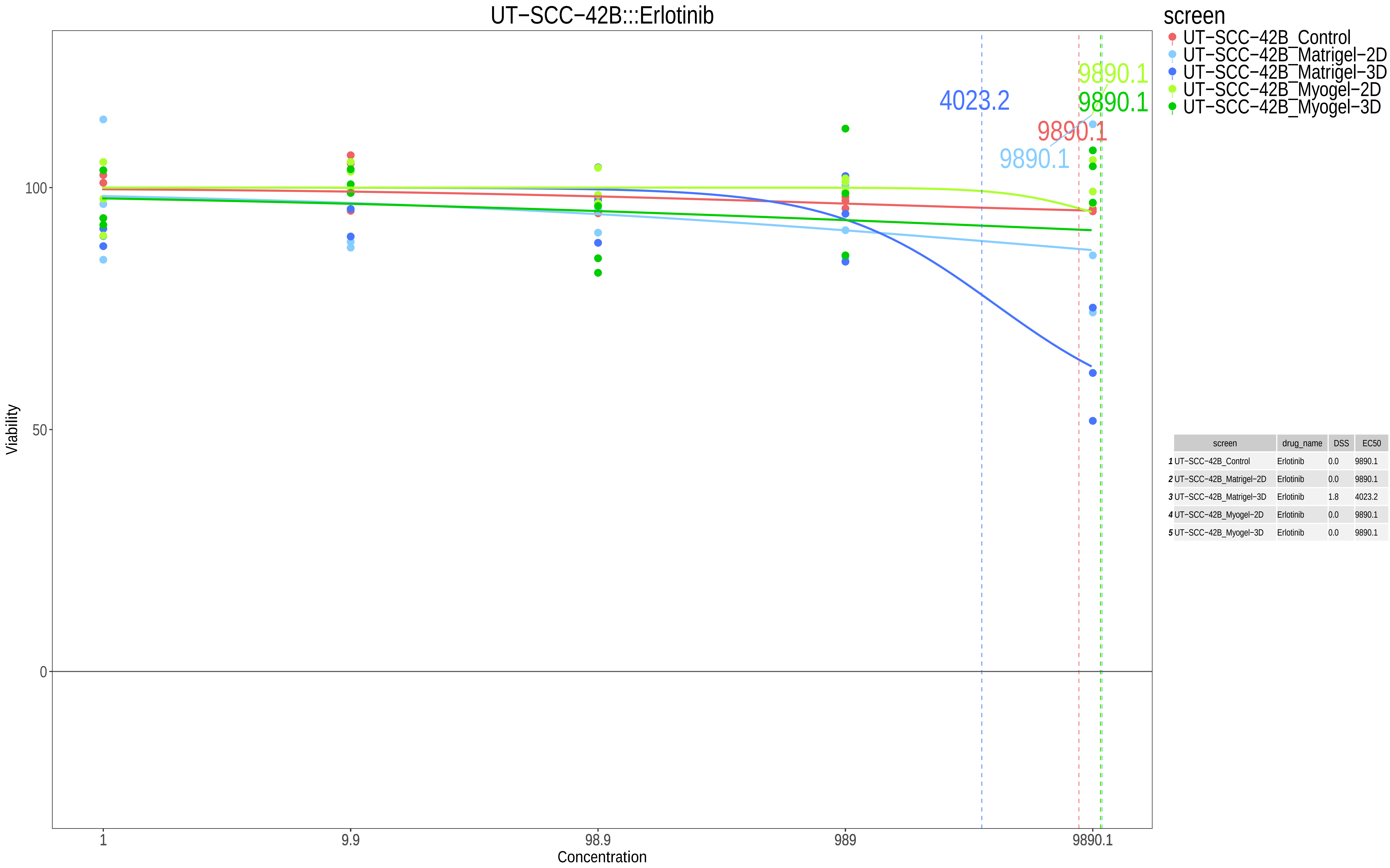

	screen	drug_name	DSS	EC50
1	UT−SCC−42B_Control	Erlotinib	0.0	9890.1
2	UT−SCC−42B_Matrigel−2D	Erlotinib	0.0	9890.1
3	UT−SCC−42B_Matrigel−3D	Erlotinib	1.8	4023.2
4	UT−SCC−42B_Myogel−2D	Erlotinib	0.0	9890.1
5	UT−SCC−42B_Myogel−3D	Erlotinib	0.0	9890.1

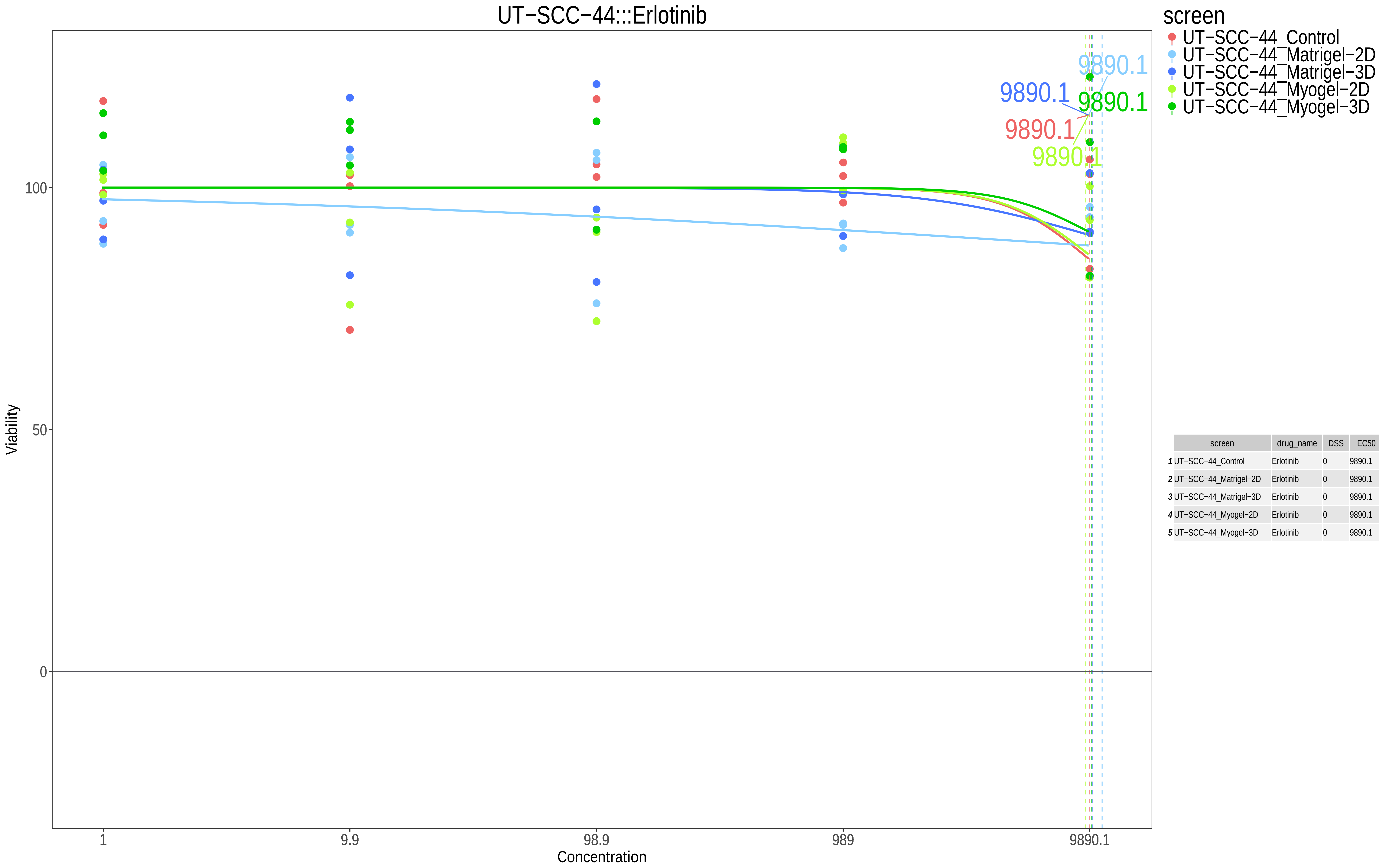

	screen	drug_name	DSS	EC50
1	UT−SCC−44_Control	Erlotinib	0	9890.1
2	UT−SCC−44_Matrigel−2D	Erlotinib	0	9890.1
3	UT−SCC−44_Matrigel−3D	Erlotinib	0	9890.1
4	UT−SCC−44_Myogel−2D	Erlotinib	0	9890.1
5	UT−SCC−44_Myogel−3D	Erlotinib	0	9890.1

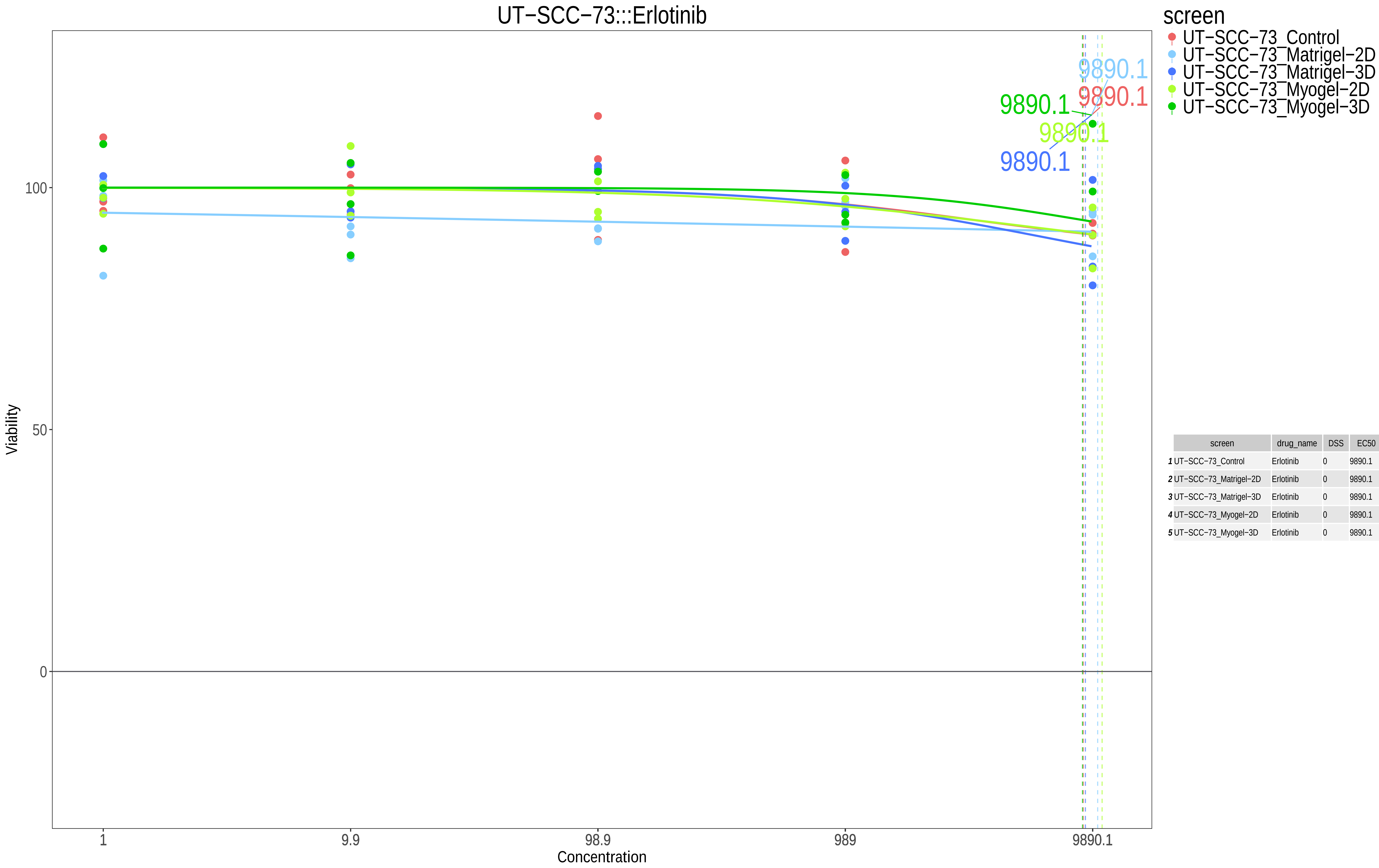

	screen	drug_name	DSS	EC50
1	UT−SCC−73_Control	Erlotinib	0	9890.1
2	UT−SCC−73_Matrigel−2D	Erlotinib	0	9890.1
3	UT−SCC−73_Matrigel−3D	Erlotinib	0	9890.1
4	UT−SCC−73_Myogel−2D	Erlotinib	0	9890.1
5	UT−SCC−73_Myogel−3D	Erlotinib	0	9890.1

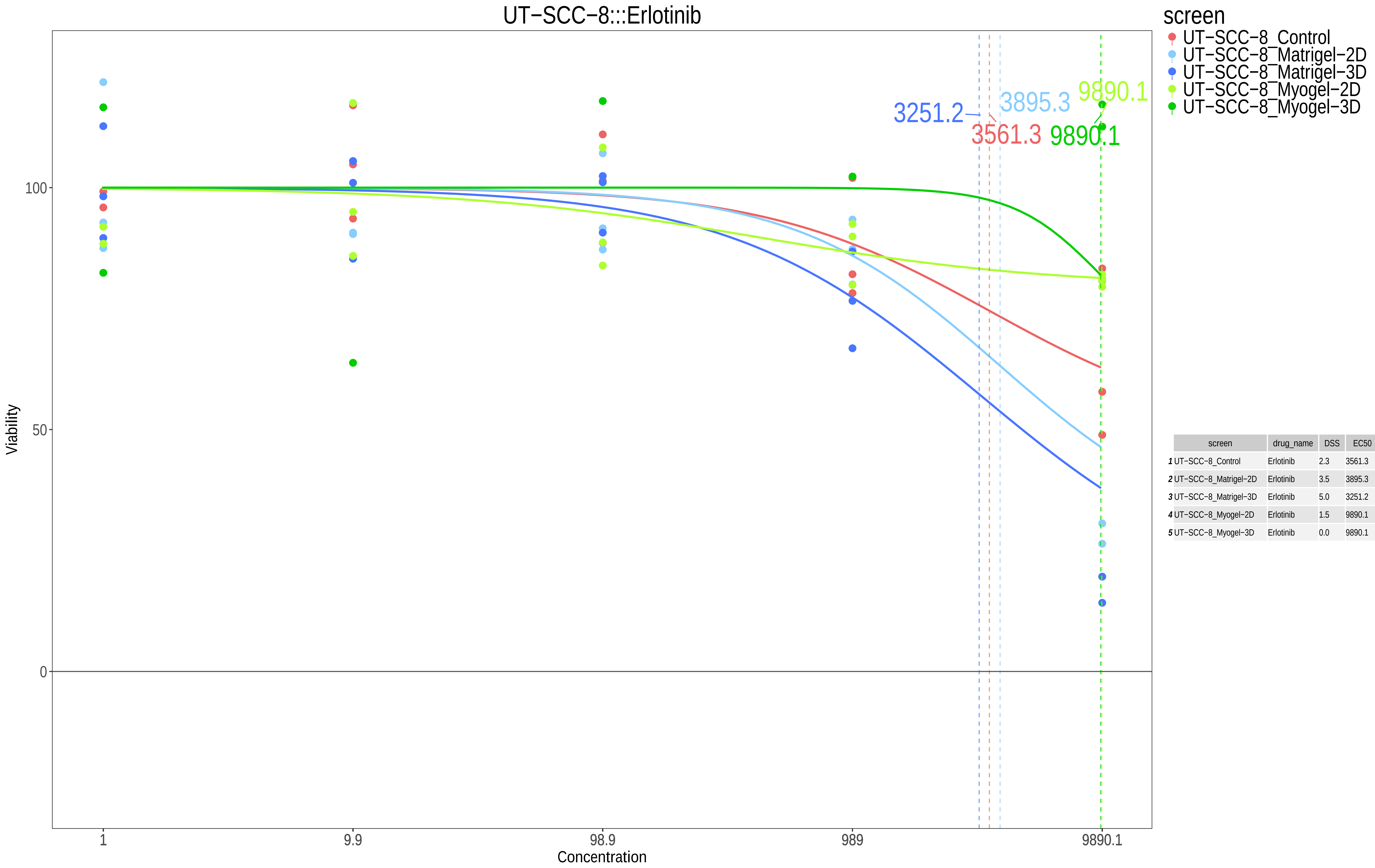

	screen	drug_name	DSS	EC50
1	UT−SCC−8_Control	Erlotinib	2.3	3561.3
2	UT−SCC−8_Matrigel−2D	Erlotinib	3.5	3895.3
3	UT−SCC−8_Matrigel−3D	Erlotinib	5.0	3251.2
4	UT−SCC−8_Myogel−2D	Erlotinib	1.5	9890.1
5	UT−SCC−8_Myogel−3D	Erlotinib	0.0	9890.1

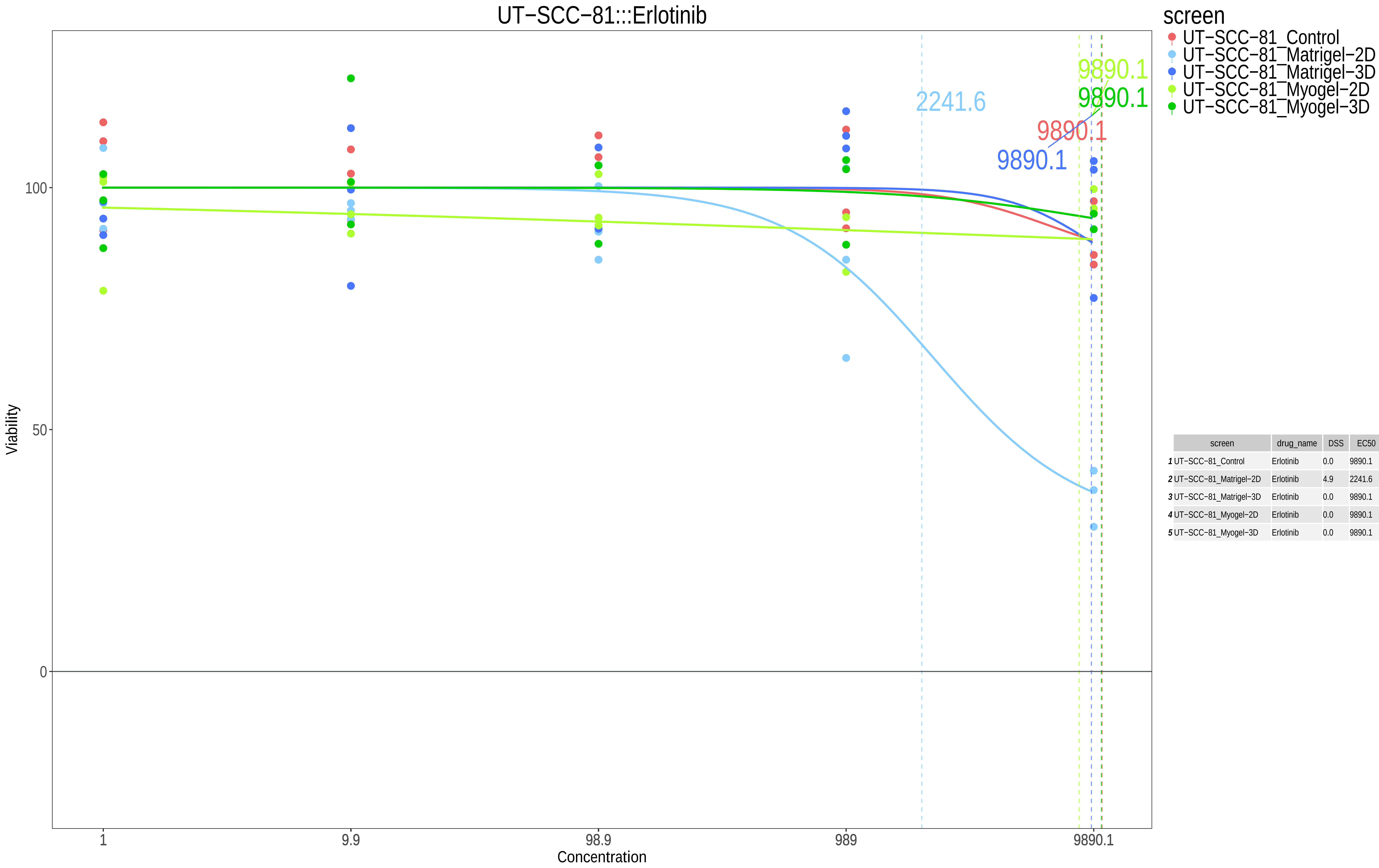

	screen	drug_name	DSS	EC50
1	UT−SCC−81_Control	Erlotinib	0.0	9890.1
2	UT−SCC−81_Matrigel−2D	Erlotinib	4.9	2241.6
3	UT−SCC−81_Matrigel−3D	Erlotinib	0.0	9890.1
4	UT−SCC−81_Myogel−2D	Erlotinib	0.0	9890.1
5	UT−SCC−81_Myogel−3D	Erlotinib	0.0	9890.1

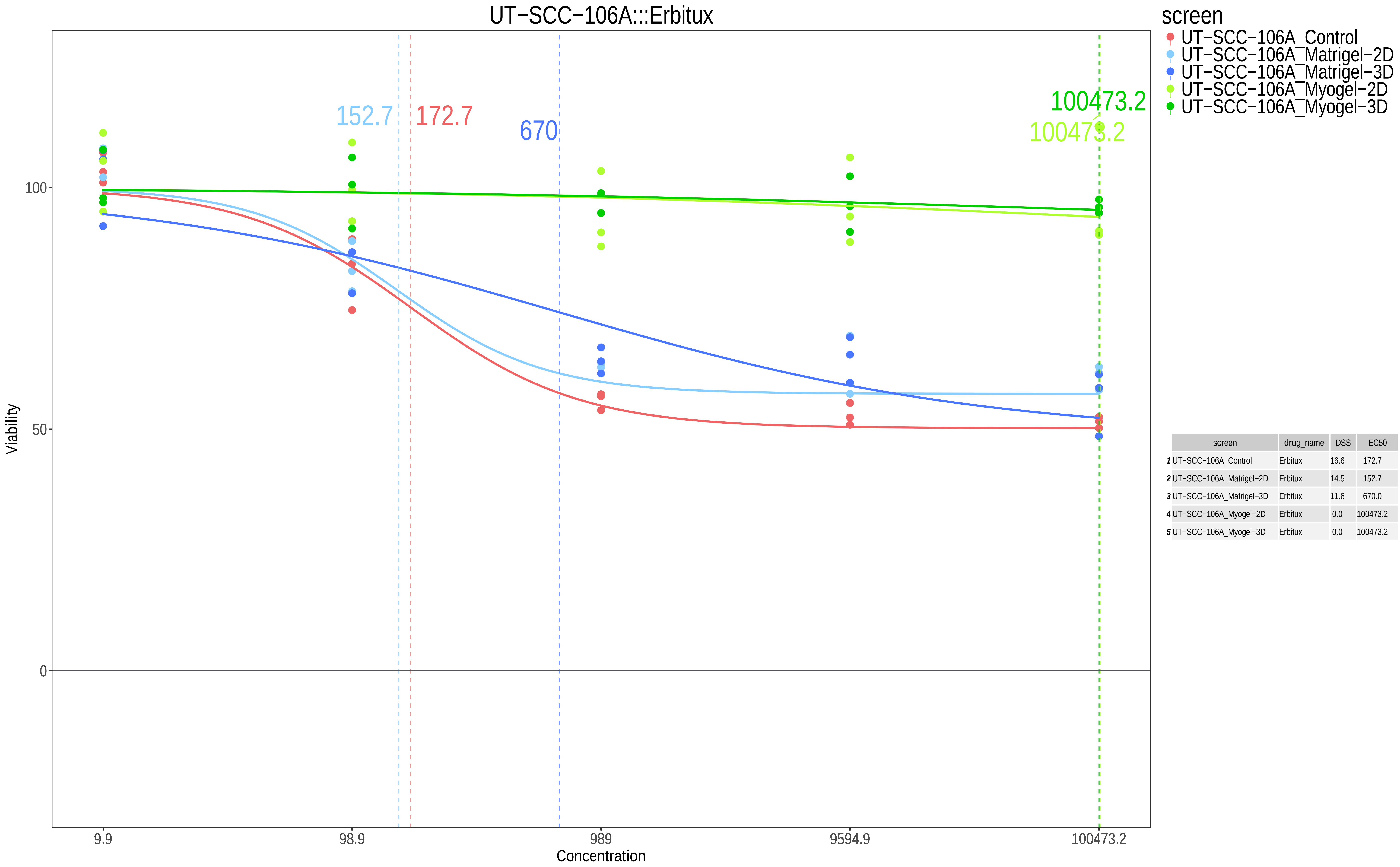

	screen	drug_name	DSS	EC50
1	UT−SCC−106A_Control	Erbitux	16.6	172.7
2	UT−SCC−106A_Matrigel−2D	Erbitux	14.5	152.7
3	UT−SCC−106A_Matrigel−3D	Erbitux	11.6	670.0
4	UT−SCC−106A_Myogel−2D	Erbitux	0.0	100473.2
5	UT−SCC−106A_Myogel−3D	Erbitux	0.0	100473.2

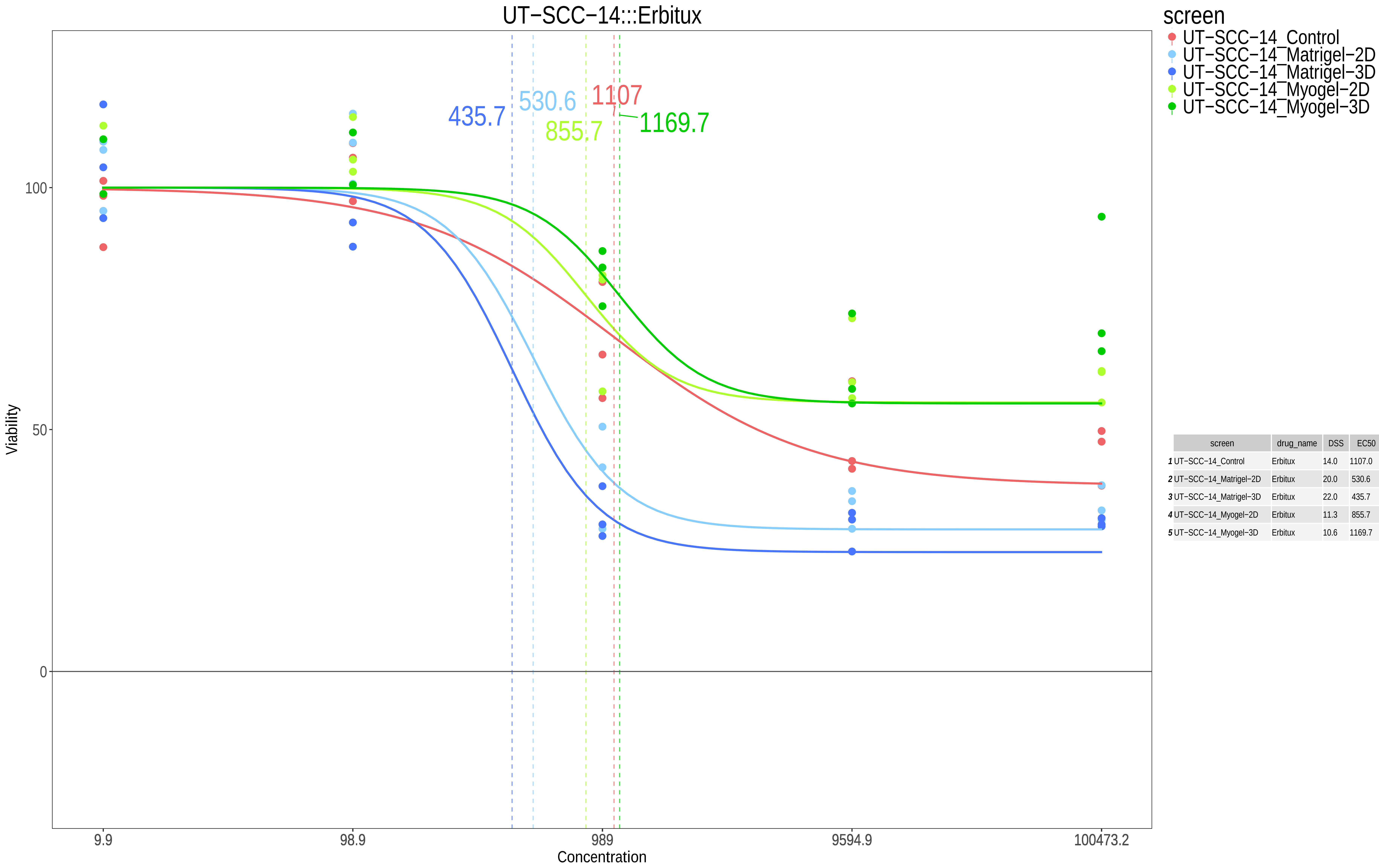

	screen	drug_name	DSS	EC50
1	UT−SCC−14_Control	Erbitux	14.0	1107.0
2	UT−SCC−14_Matrigel−2D	Erbitux	20.0	530.6
3	UT−SCC−14_Matrigel−3D	Erbitux	22.0	435.7
4	UT−SCC−14_Myogel−2D	Erbitux	11.3	855.7
5	UT−SCC−14_Myogel−3D	Erbitux	10.6	1169.7

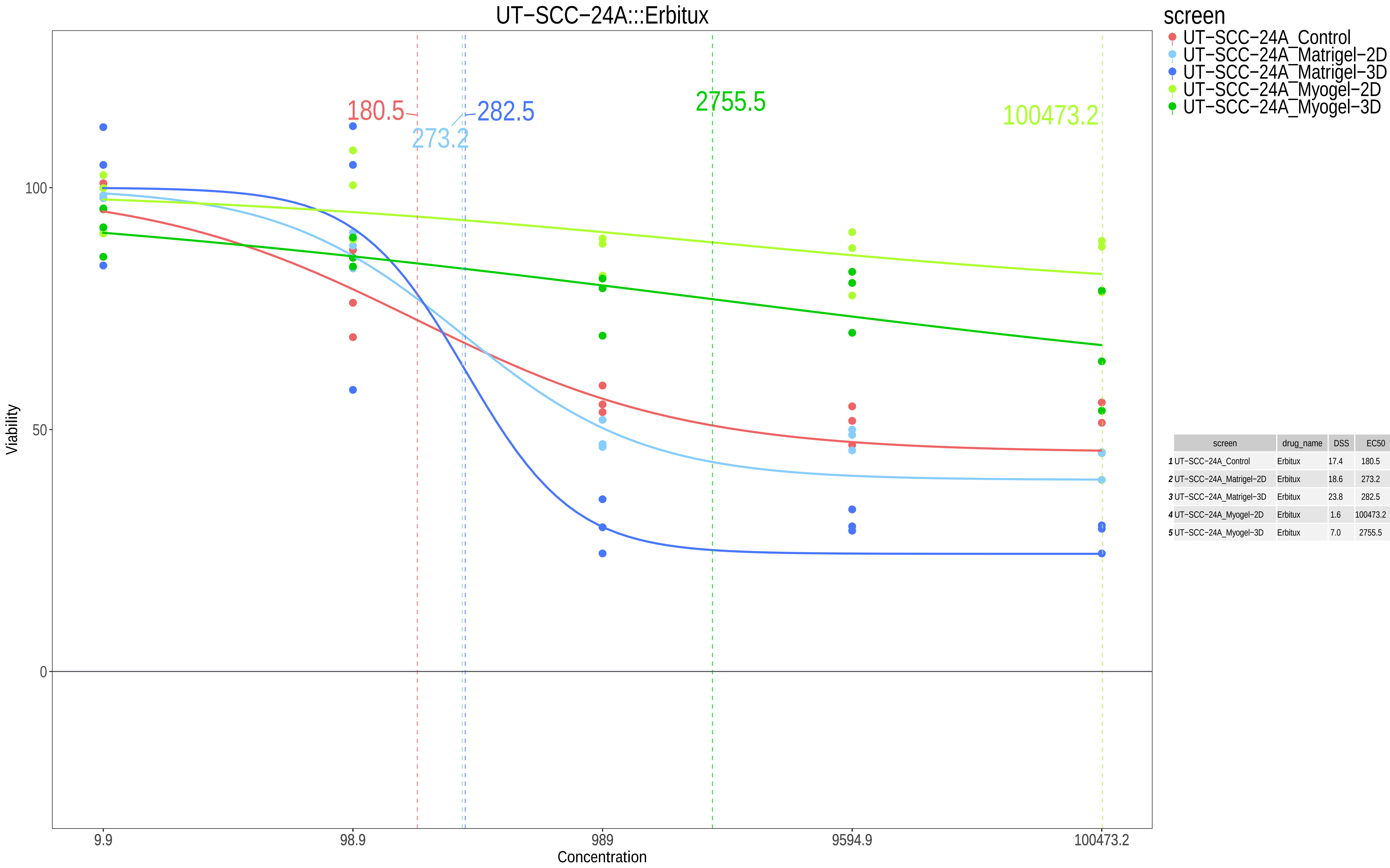

	screen	drug_name	DSS	EC50
1	UT−SCC−24A_Control	Erbitux	17.4	180.5
2	UT−SCC−24A_Matrigel−2D	Erbitux	18.6	273.2
3	UT−SCC−24A_Matrigel−3D	Erbitux	23.8	282.5
4	UT−SCC−24A_Myogel−2D	Erbitux	1.6	100473.2
5	UT−SCC−24A_Myogel−3D	Erbitux	7.0	2755.5

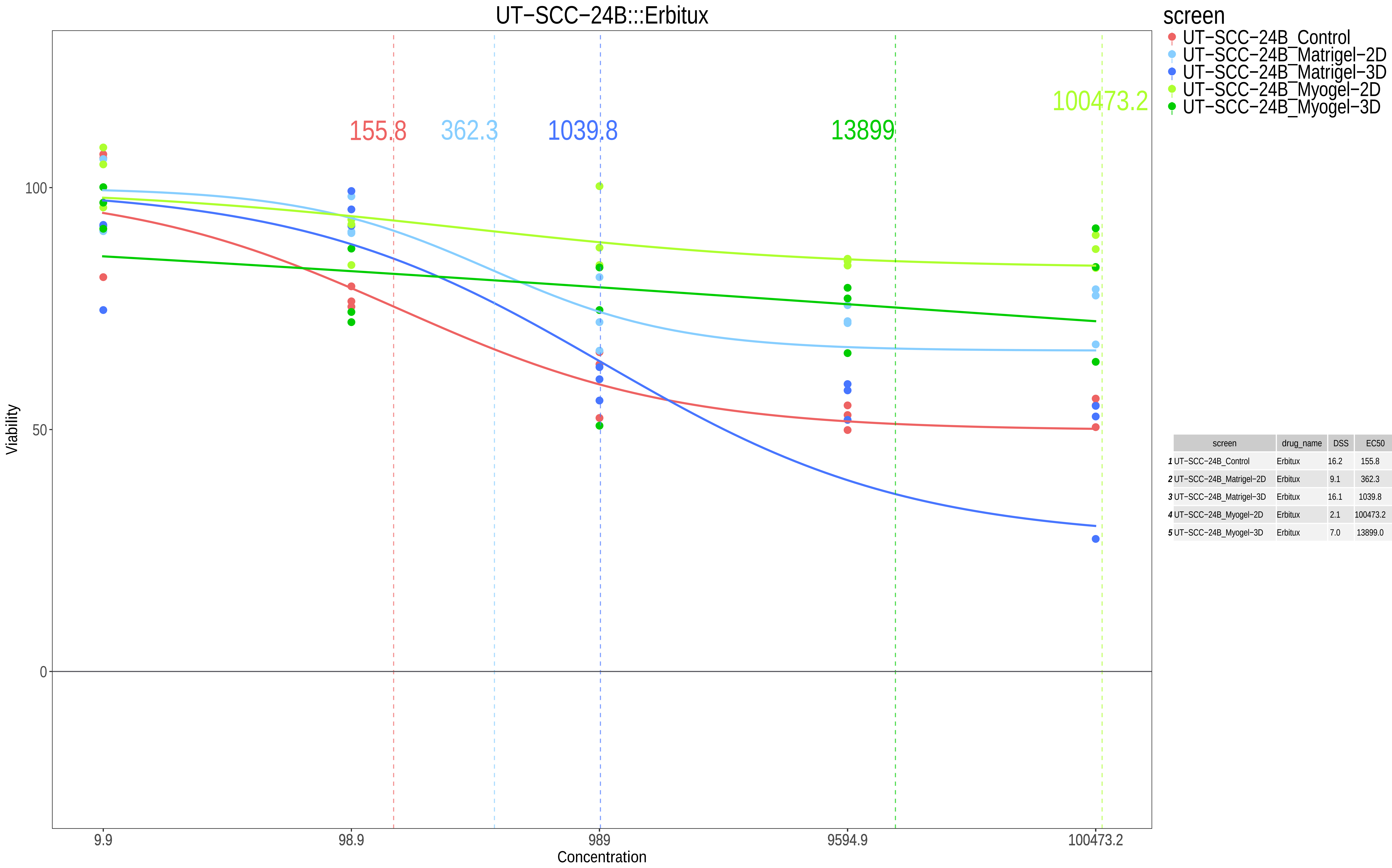

	screen	drug_name	DSS	EC50
1	UT−SCC−24B_Control	Erbitux	16.2	155.8
2	UT−SCC−24B_Matrigel−2D	Erbitux	9.1	362.3
3	UT−SCC−24B_Matrigel−3D	Erbitux	16.1	1039.8
4	UT−SCC−24B_Myogel−2D	Erbitux	2.1	100473.2
5	UT−SCC−24B_Myogel−3D	Erbitux	7.0	13899.0

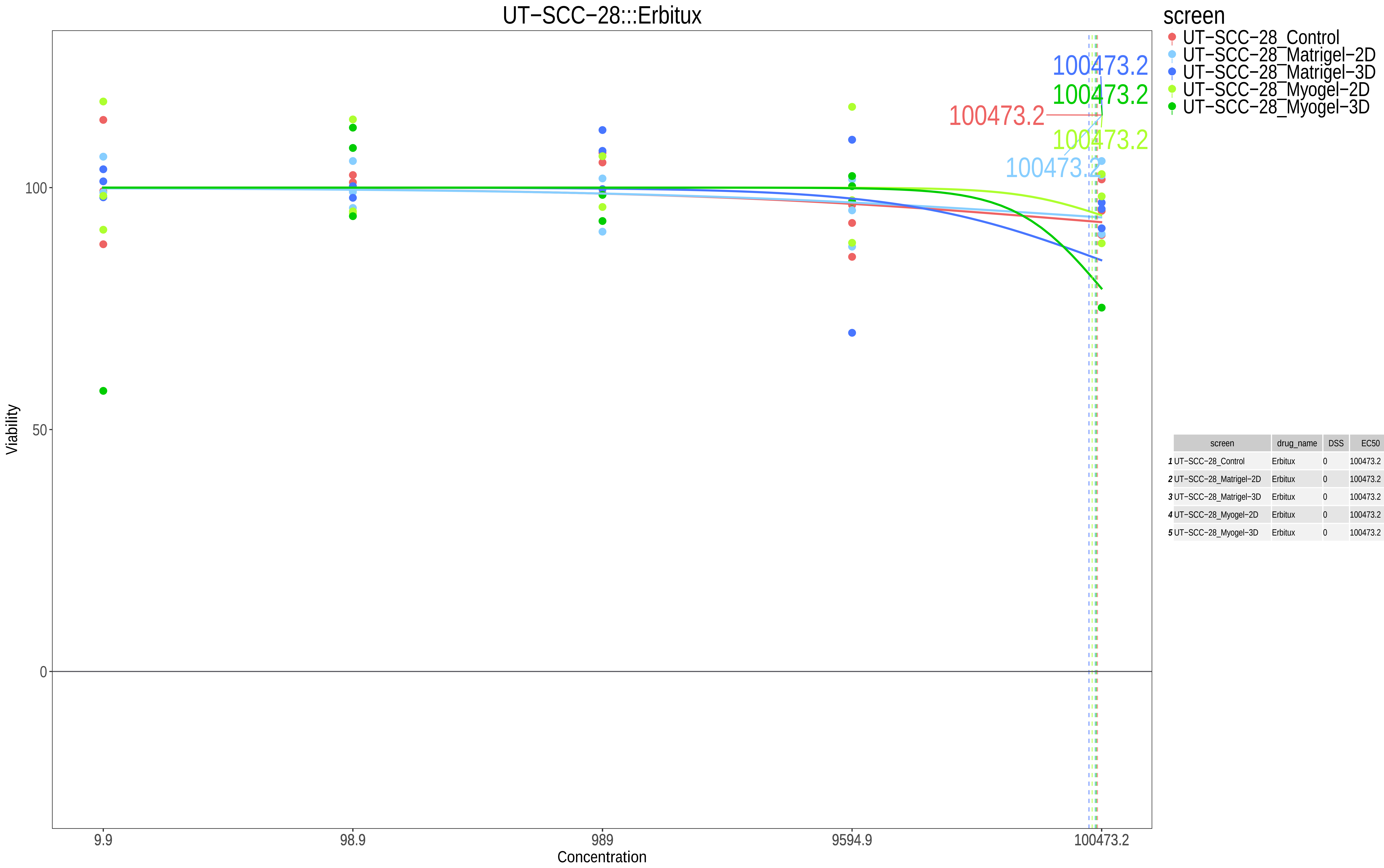

	screen	drug_name	DSS	EC50
1	UT−SCC−28_Control	Erbitux	0	100473.2
2	UT−SCC−28_Matrigel−2D	Erbitux	0	100473.2
3	UT−SCC−28_Matrigel−3D	Erbitux	0	100473.2
4	UT−SCC−28_Myogel−2D	Erbitux	0	100473.2
5	UT−SCC−28_Myogel−3D	Erbitux	0	100473.2

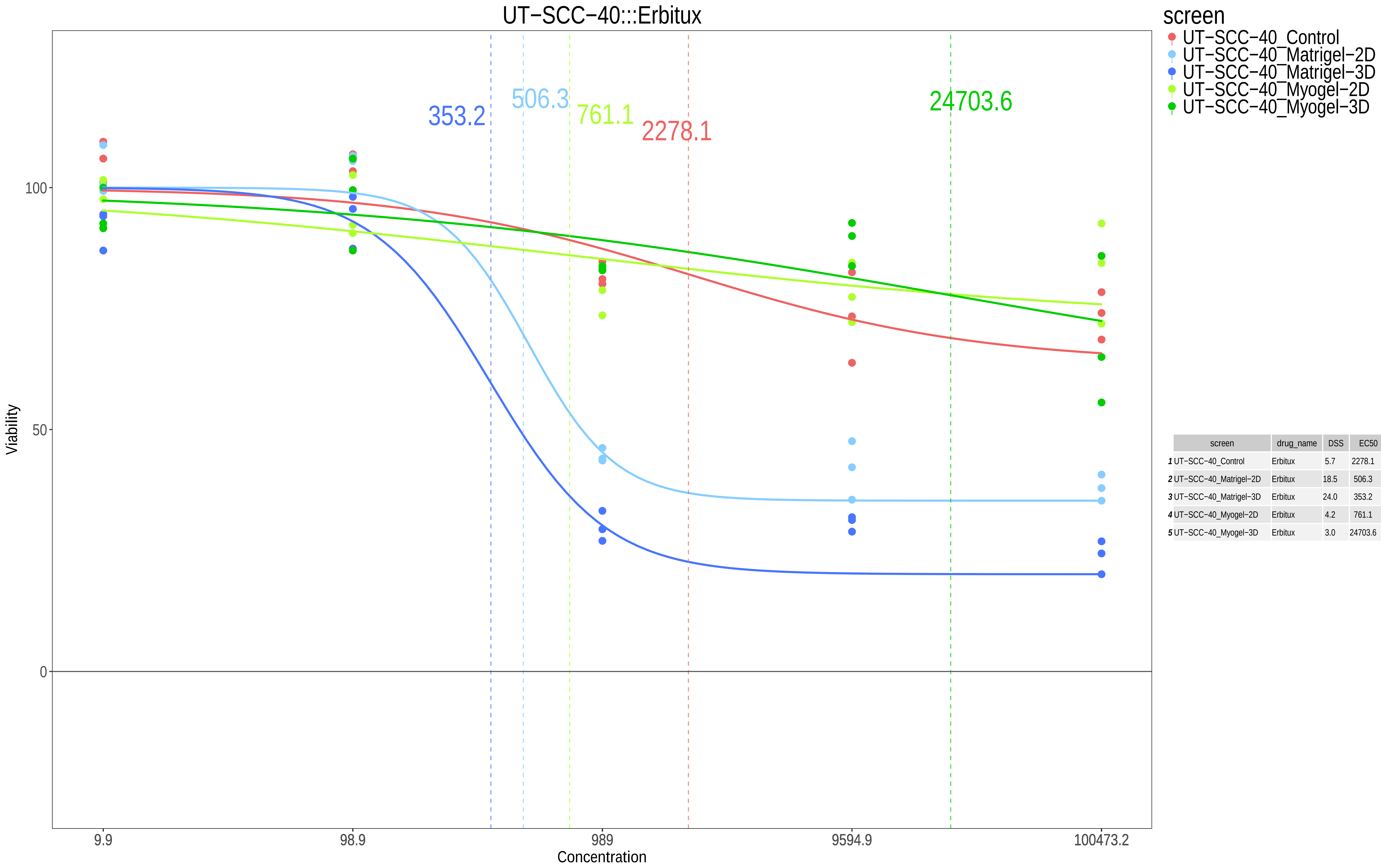

	screen	drug_name	DSS	EC50
1	UT−SCC−40_Control	Erbitux	5.7	2278.1
2	UT−SCC−40_Matrigel−2D	Erbitux	18.5	506.3
3	UT−SCC−40_Matrigel−3D	Erbitux	24.0	353.2
4	UT−SCC−40_Myogel−2D	Erbitux	4.2	761.1
5	UT−SCC−40_Myogel−3D	Erbitux	3.0	24703.6

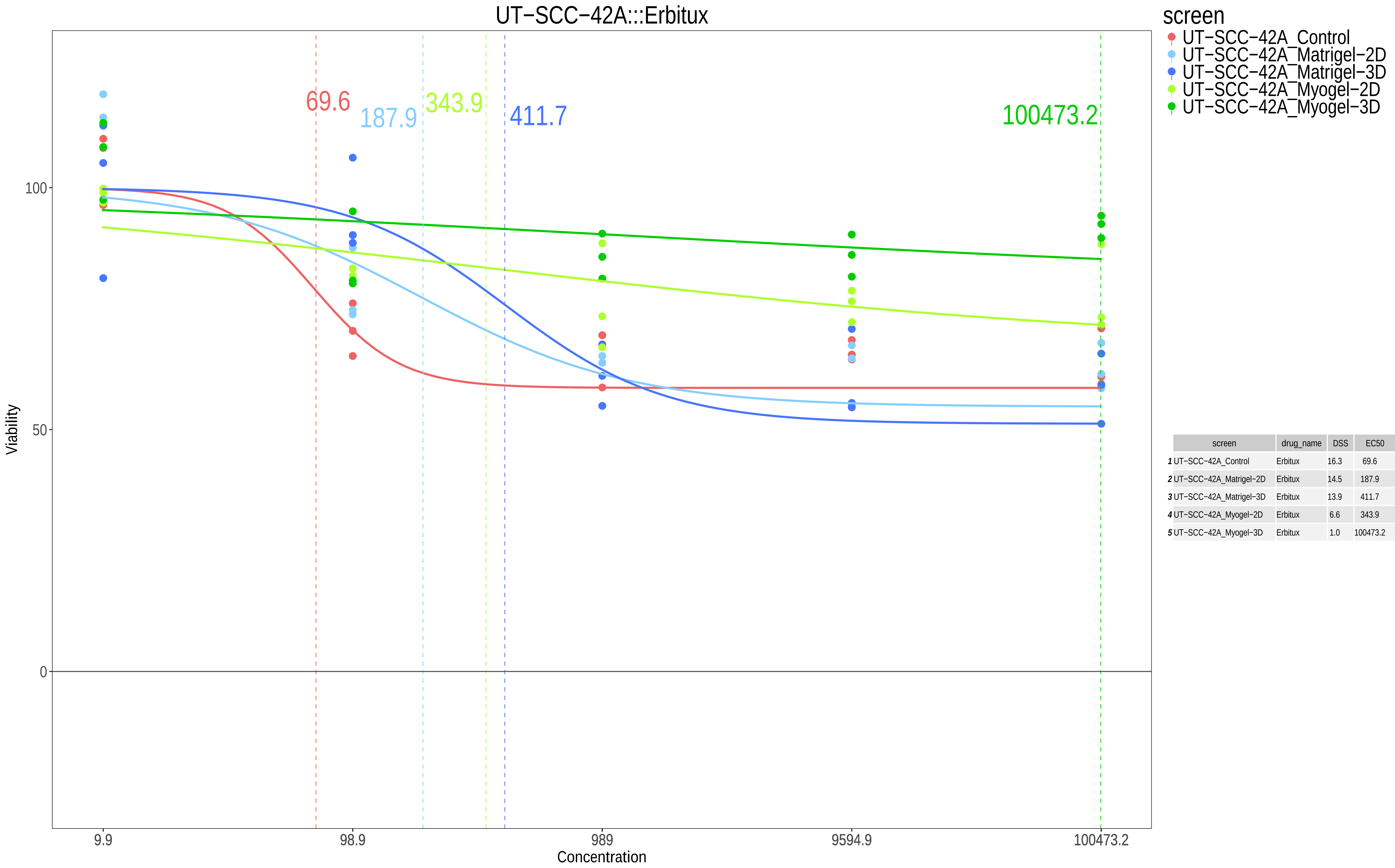

	screen	drug_name	DSS	EC50
1	UT−SCC−42A_Control	Erbitux	16.3	69.6
2	UT−SCC−42A_Matrigel−2D	Erbitux	14.5	187.9
3	UT−SCC−42A_Matrigel−3D	Erbitux	13.9	411.7
4	UT−SCC−42A_Myogel−2D	Erbitux	6.6	343.9
5	UT−SCC−42A_Myogel−3D	Erbitux	1.0	100473.2

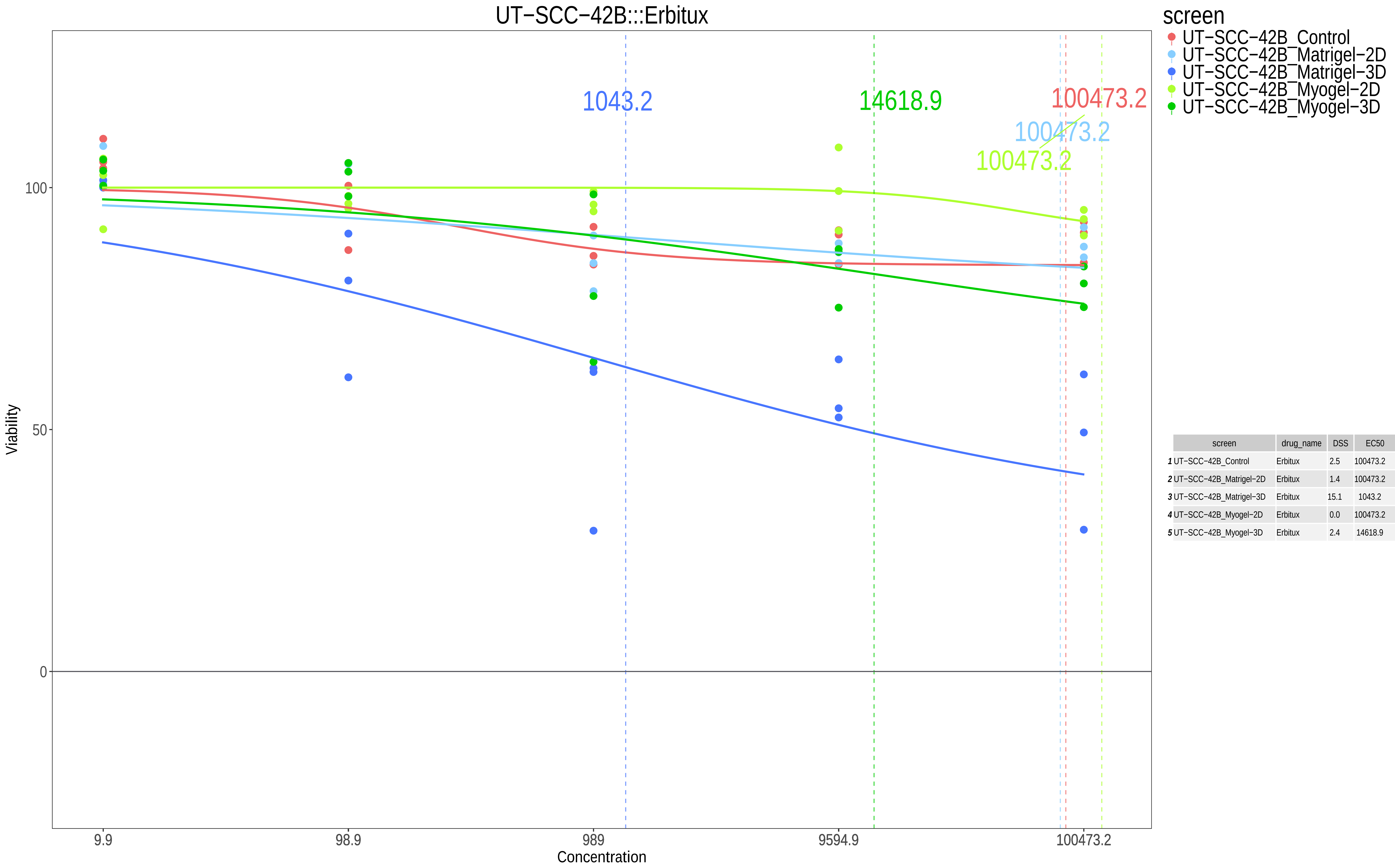

	screen	drug_name	DSS	EC50
1	UT−SCC−42B_Control	Erbitux	2.5	100473.2
2	UT−SCC−42B_Matrigel−2D	Erbitux	1.4	100473.2
3	UT−SCC−42B_Matrigel−3D	Erbitux	15.1	1043.2
4	UT−SCC−42B_Myogel−2D	Erbitux	0.0	100473.2
5	UT−SCC−42B_Myogel−3D	Erbitux	2.4	14618.9

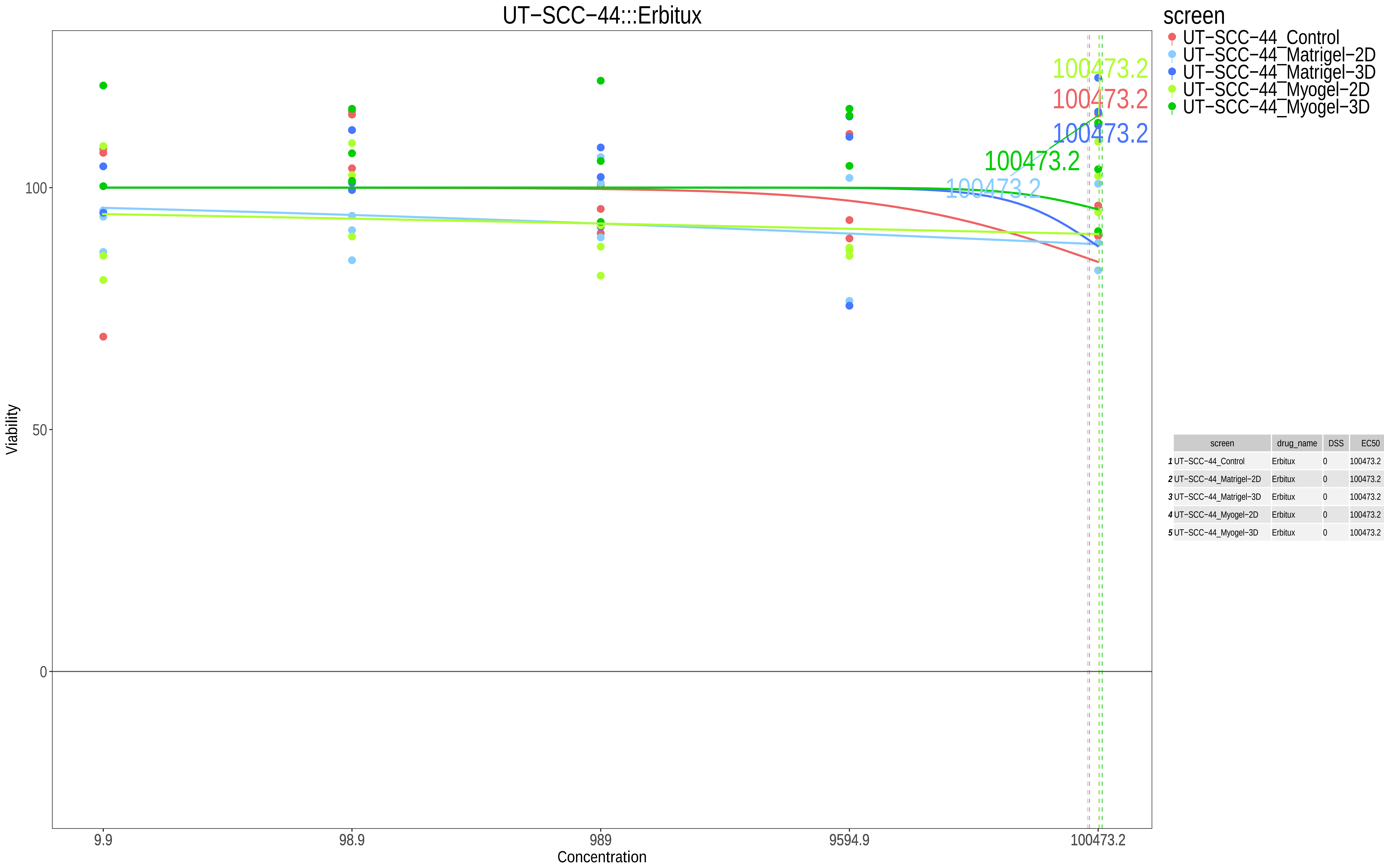

	screen	drug_name	DSS	EC50
1	UT−SCC−44_Control	Erbitux	0	100473.2
2	UT−SCC−44_Matrigel−2D	Erbitux	0	100473.2
3	UT−SCC−44_Matrigel−3D	Erbitux	0	100473.2
4	UT−SCC−44_Myogel−2D	Erbitux	0	100473.2
5	UT−SCC−44_Myogel−3D	Erbitux	0	100473.2

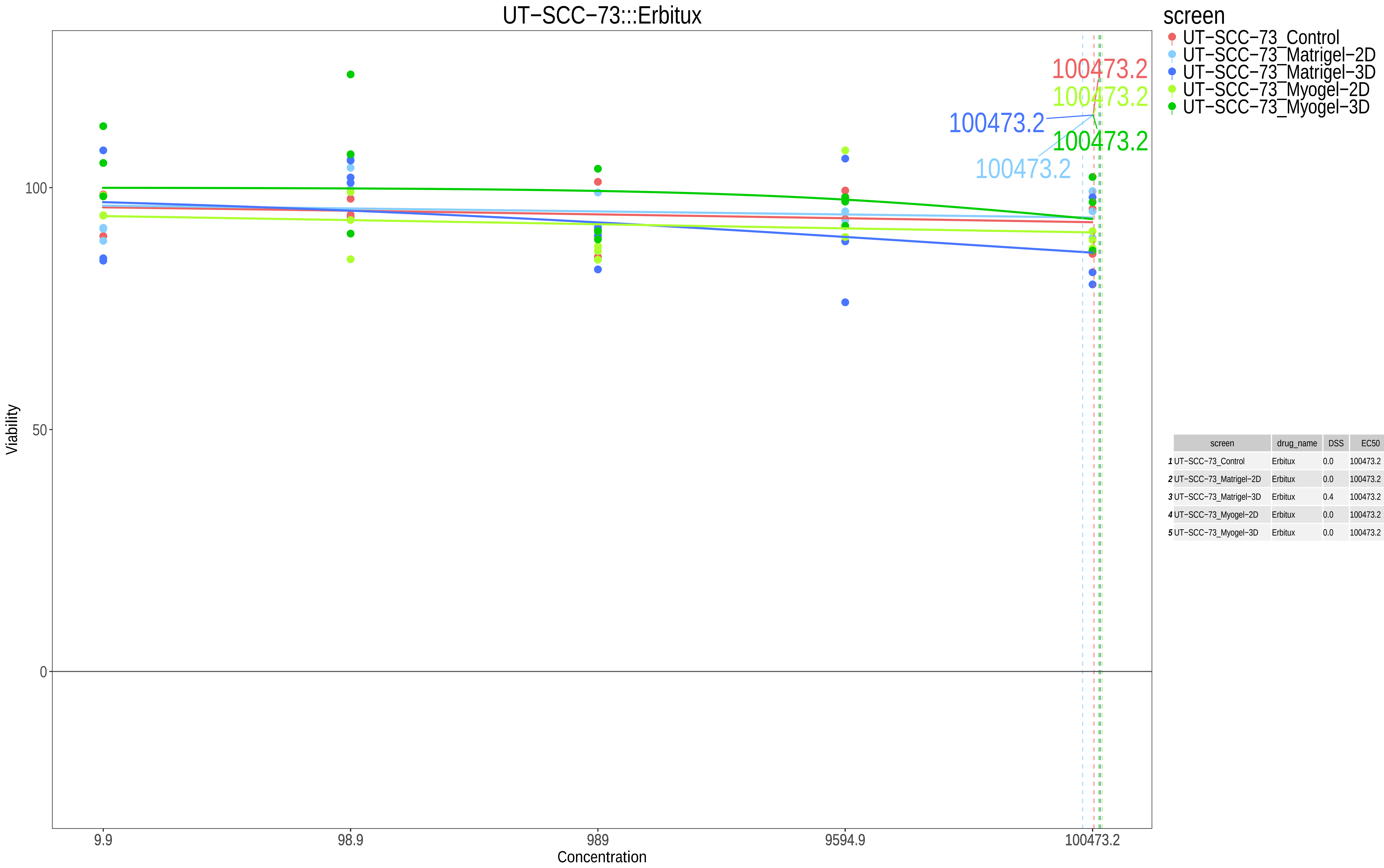

	screen	drug_name	DSS	EC50
1	UT−SCC−73_Control	Erbitux	0.0	100473.2
2	UT−SCC−73_Matrigel−2D	Erbitux	0.0	100473.2
3	UT−SCC−73_Matrigel−3D	Erbitux	0.4	100473.2
4	UT−SCC−73_Myogel−2D	Erbitux	0.0	100473.2
5	UT−SCC−73_Myogel−3D	Erbitux	0.0	100473.2

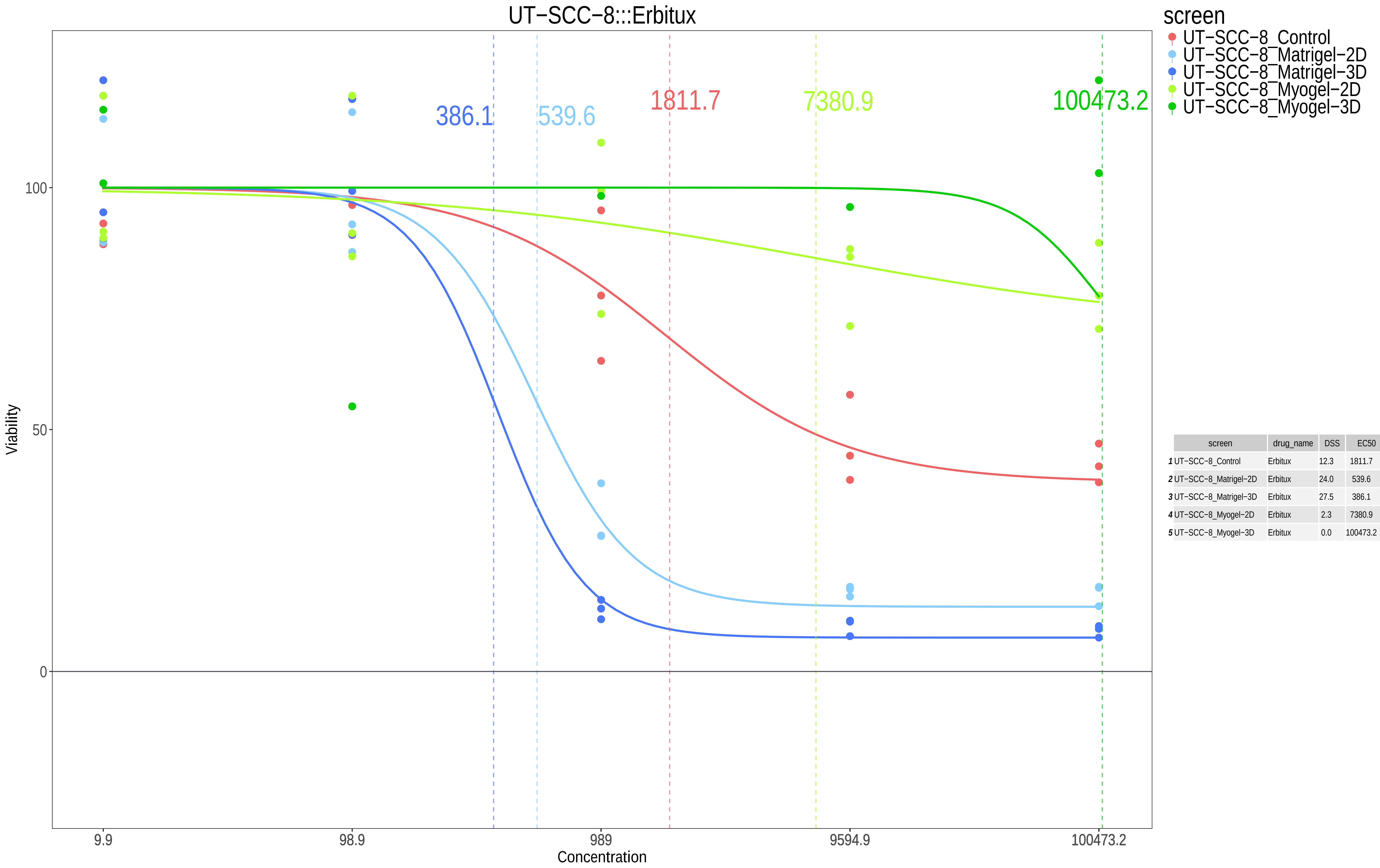

	screen	drug_name	DSS	EC50
1	UT−SCC−8_Control	Erbitux	12.3	1811.7
2	UT−SCC−8_Matrigel−2D	Erbitux	24.0	539.6
3	UT−SCC−8_Matrigel−3D	Erbitux	27.5	386.1
4	UT−SCC−8_Myogel−2D	Erbitux	2.3	7380.9
5	UT−SCC−8_Myogel−3D	Erbitux	0.0	100473.2

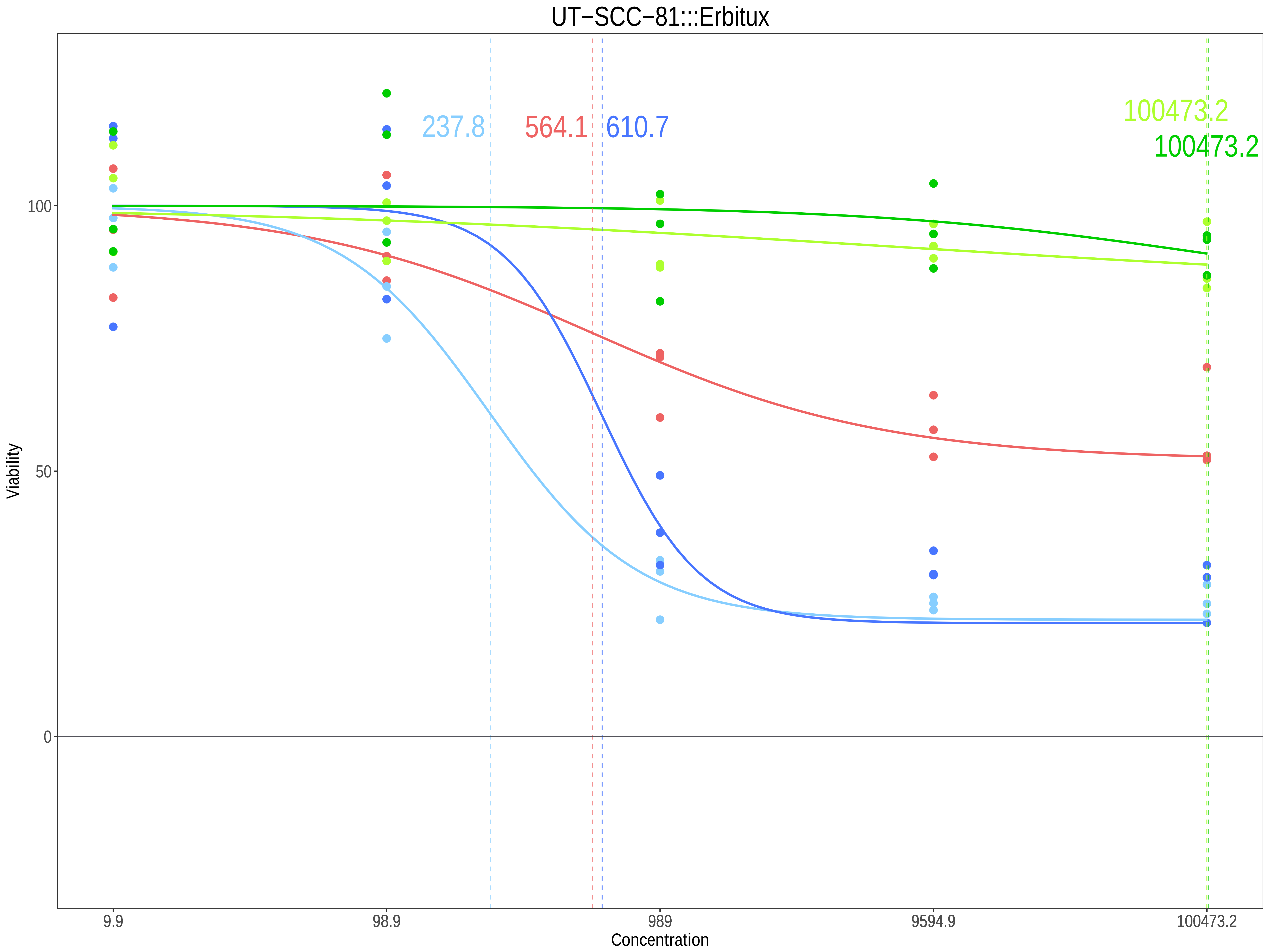

	screen	drug_name	DSS	EC50
1	UT−SCC−81_Control	Erbitux	12.0	564.1
2	UT−SCC−81_Matrigel−2D	Erbitux	25.0	237.8
3	UT−SCC−81_Matrigel−3D	Erbitux	21.5	610.7
4	UT−SCC−81_Myogel−2D	Erbitux	0.1	100473.2
5	UT−SCC−81_Myogel−3D	Erbitux	0.0	100473.2
