# Supplementary material for: Human Tumor–Derived Matrix Improves the Predictability of Head and Neck Cancer Drug Testing
Source: Cancers (Basel). 2019 Dec 30;12(1):92. doi: 10.3390/cancers12010092 (PMC7017272; doi:10.3390/cancers12010092)
Supplement: Supplementary file 1 [file cancers-12-00092-s001.zip › cancers-664648-supplement-final/Supplementary Table 1.pdf]

Supplementary Table 1: Response rates for the drugs tested on the cell lines under all culturing conditions. Response rates for each anticancer compound were calculated using DSS ≥ 5 as the cut-off point.

		Response rate %				
Drug No	Preferred name	Control	Myogel 2D	Myogel 3D	Matrigel 2D	Matrigel 3D
1	Afatinib	75	16,7	16,7	75	75
2	Canertinib	100	100	91,7	100	100
3	Gefitinib	16,7	8,3	8,3	41,7	50
4	Erlotinib	8,3	0	8,3	16,7	25
5	Refametinib	91,7	91,7	75	100	100
6	Binimetinib	50	41,7	33,3	75	91,7
7	Selumetinib	66,7	66,7	33,3	83,3	100
8	Trametinib	66,7	25	41,7	83,3	100
9	Pimasertib	91,7	100	100	100	100
10	TAK-733	75	66,7	50	100	100
11	Dactolisib	8,3	8,3	8,3	8,3	0
12	PF-04691502	83,3	83,3	58,3	91,7	100
13	Apitolisib	91,7	91,7	91,7	100	100
14	Omipalisib	100	100	100	100	100
15	Everolimus	16,7	41,7	25	25	8,3
16	Temsirolimus	75	83,3	50	66,7	66,7
17	Ridaforolimus	83,3	58,3	58,3	58,3	41,7
18	Sirolimus	91,7	83,3	75	91,7	66,7
19	Erbitux	66,7	16,7	25	66,7	75
