# Supplementary material for: Human Tumor–Derived Matrix Improves the Predictability of Head and Neck Cancer Drug Testing
Source: Cancers (Basel). 2019 Dec 30;12(1):92. doi: 10.3390/cancers12010092 (PMC7017272; doi:10.3390/cancers12010092)
Supplement: Supplementary file 1 [file cancers-12-00092-s001.zip › cancers-664648-supplement-final/Supplementary Table 2.pdf]

Supplementary Table 2: Clinical and pathological characteristics of the HNSCC cell lines. TNM is based on pathology report.

Cell line	Sex[a]	Age[b]	TNM	Specimen site	Type[c]	Grade	Passage
**UT-SCC-8**	M	42	T2N0M0	larynx	pri	G1	46
**UT-SCC-14**	M	25	T3N1M0	tongue	pri(per)	G2	31
**UT-SCC-24A**	M	41	T2N0M0	tongue	pri	G2	9
**UT-SCC-24B**	M	41	T2N1M0	neck	met(per)	G2	36
**UT-SCC-28**	F	48	T2N0M0	floor of mouth	pri(per)	G1	32
**UT-SCC-42A**	M	43	T4N3M0	larynx	pri	G3	14
**UT-SCC-42B**	M	43	T4N3M0	neck	met	G3	17
**UT-SCC-40**	M	65	T3N0M0	tongue	pri	G1	9
**UT-SCC-44**	F	71	T4N2BM0	gingiva of mandib.	pri(per)	G3	31
**UT-SCC-73**	F	86	T1N0M0	tongue	pri	G2	16
**UT-SCC-81**	M	48	T2N0M0	tongue	pri	G1	16
**UT-SCC-106A**	M	37	T1AN0M0	larynx	pri	G1	17

[a]M=male , F=female, [b] Age in years, [C] Pri=primary tumor, met=metastasis, per= persistent disease
