# Supplementary material for: Human Tumor–Derived Matrix Improves the Predictability of Head and Neck Cancer Drug Testing
Source: Cancers (Basel). 2019 Dec 30;12(1):92. doi: 10.3390/cancers12010092 (PMC7017272; doi:10.3390/cancers12010092)
Supplement: Supplementary file 1 [file cancers-12-00092-s001.zip › cancers-664648-supplement-final/Supplementary Table 3.pdf]

Supplementary Table 3: List of the anticancer compounds screened against the cell lines tested in this study. Each drug was tested over a 10 000-fold concentration range.

Drug No	FIMM batch ID	Drug name	Start conc. (nM)	Max conc. (nM)	Res. code	Alias	Trade names	Mechanism/Targets	Supplier	Supplier Ref
1	FIMM003710-001	Afatinib	0,099	989,011	BIBW2992		Gilotrif, Giotrif	EGFR inhibitor	Selleck	S1011
2	FIMM003781-001	Canertinib	0,989	9890,110	CI-1033	PD 183805		pan-HER inhibitor	LC Laboratorie	C-1201
3	FIMM000160-003	Gefitinib	0,989	9890,110	ZD-1839		Iressa	EGFR inhibitor	LC Laboratorie	G-4408
4	FIMM000183-004	Erlotinib	0,989	9890,110	CP-358774	OSI-774	Tarceva	EGFR inhibitor	Medchem Expr	HY-50896
5	FIMM003754-001	Refametinib	0,989	9890,110	BAY 869766	RDEA119		MEK1/2 inhibitor	ChemieTek	CT-R119
6	FIMM100407-001	Binimetinib	0,099	989,011	NVP-MEK162	MEK162, ARRY-438162, ARRY-162		MEK1/2 inhibitor	ChemieTek	CT-A162
7	FIMM003708-002	Selumetinib	0,989	9890,110	AZD6244	ARRY-142886		MEK1/2 inhibitor	Medchem Expr	HY-50706
8	FIMM003751-001	Trametinib	0,025	247,253	GSK1120212	JTP-74057	Mekinist	MEK1/2 inhibitor	ChemieTek	CT-GSK112
9	FIMM003729-001	Pimasertib	0,989	9890,110	AS703026	MSC1936369B		MEK inhibitor	Selleck	S1475
10	FIMM100412-001	TAK-733	0,099	989,011	TAK-733			MEK inhibitor	Selleck	S2617
11	FIMM023832-001	Dactolisib	0,099	989,011	NVP-BEZ235			mTOR/(PI3K) inhibitor	LC Laboratorie	N-4288
12	FIMM003735-001	PF-04691502	0,989	9890,110	PF-04691502			PI3K/mTOR inhibitor	ChemieTek	CT-PF1502
13	FIMM100390-001	Apitolisib	0,989	9890,110	GDC-0980			PI3K/mTOR inhibitor	ChemieTek	CT-G0980
14	FIMM100414-002	Omipalisib	0,099	989,011	GSK2126458			PI3K/mTOR inhibitor	Medchem Expr	HY-10297
15	FIMM003755-001	Everolimus	0,010	98,901	NVP-RAD001	RAD001, SDZ-RAD	Afinitor, Certican, Zort	binds FKBP12, causes inhibit	LC Laboratorie	E-4040
16	FIMM003786-001	Temsirolimus	0,010	98,901	CCI-779		Torisel	binds FKBP12, causes inhibit	LC Laboratorie	T-8040
17	FIMM100397-001	Ridaforolimus	0,010	98,901	MK-8669	AP 23573, Deforolimus		binds FKBP12, causes inhibit	Active Biochem	A-1004
18	FIMM023795-004	Sirolimus	0,010	98,901		Rapamycin	Rapamune	binds FKBP12, causes inhibit	LC Laboratorie	R-5000
19		Cetuximab	0,005*	49,451*		CETUXIMAB,ERBITUX	Erbitux	EGFR inhibitor	Merck KGaA	

*µg/ml
