# Supplementary material for: Human Tumor–Derived Matrix Improves the Predictability of Head and Neck Cancer Drug Testing
Source: Cancers (Basel). 2019 Dec 30;12(1):92. doi: 10.3390/cancers12010092 (PMC7017272; doi:10.3390/cancers12010092)
Supplement: Supplementary file 1 [file cancers-12-00092-s001.zip › cancers-664648-supplement-final/Supplementary Table 4.pdf]

Supplementary Table 4: Z' factor values for each screen.

Plate	Z_Prime	SSMD	Signal_Vs_BG	Mean_DMSO	SD_DMSO	CV_DMSO	Mean_BzCl	SD_BzCl	CV_BzCl	Mean_Cells	SD_Cells	CV_Cells
UT-SCC-106A_Control	0.74	12	27.3	55167.6	4129.5	7.9	2023.0	549.5	28.6	54895.6	3837.1	7.0
UT-SCC-106A_Matrigel-2D	0.66	9	28.7	52187.1	5296.7	10.7	1819.3	484.3	28.1	48519.8	4244.0	8.8
UT-SCC-106A_Matrigel-3D	0.57	7	46.8	47096.3	6399.5	14.3	1005.8	201.9	21.2	46828.6	5221.5	11.2
UT-SCC-106A_Myogel-2D	0.85	23	23.8	52553.2	1990.2	4.0	2210.9	510.2	24.3	51013.1	3571.2	7.0
UT-SCC-106A_Myogel-3D	0.79	16	25.4	46618.0	2654.9	6.0	1834.2	536.3	30.8	44748.0	3340.5	7.5
UT-SCC-14_Control	0.39	5	25.8	35739.9	6455.3	19.0	1383.2	506.9	38.6	29221.7	6082.9	20.9
UT-SCC-14_Matrigel-2D	0.64	9	34.7	53531.0	5767.5	11.4	1542.6	460.8	31.5	47586.5	7841.1	16.6
UT-SCC-14_Matrigel-3D	0.65	8	60.2	68240.7	7565.1	11.7	1134.1	370.2	34.4	49331.0	11024.0	22.5
UT-SCC-14_Myogel-2D	0.50	6	26.9	51797.0	7565.5	15.4	1922.2	681.6	37.4	41982.4	7562.5	18.1
UT-SCC-14_Myogel-3D	0.53	6	34.1	44686.9	6363.2	15.0	1309.6	465.5	37.5	38201.3	6579.7	17.3
UT-SCC-24A_Control	0.74	12	29.8	32745.5	2415.4	7.8	1100.4	286.6	27.5	31825.3	4621.7	14.6
UT-SCC-24A_Matrigel-2D	0.76	13	37.2	53148.5	3813.0	7.6	1428.3	384.2	28.4	53836.4	6485.9	12.1
UT-SCC-24A_Matrigel-3D	0.56	7	58.2	66306.4	9310.1	14.8	1140.0	269.6	24.9	54323.2	11987.4	22.2
UT-SCC-24A_Myogel-2D	0.87	28	24.5	39808.8	1198.4	3.2	1626.5	455.2	29.5	37641.6	4106.9	11.0
UT-SCC-24A_Myogel-3D	0.65	9	38.3	46043.9	4836.6	11.1	1203.3	370.0	32.4	43890.3	7997.2	18.3
UT-SCC-24B_Control	0.70	10	29.0	58178.4	5138.9	9.3	2005.3	472.1	24.8	51954.3	4155.3	8.0
UT-SCC-24B_Matrigel-2D	0.80	15	36.1	67507.1	4001.1	6.2	1868.9	472.3	26.6	64867.7	5012.3	7.8
UT-SCC-24B_Matrigel-3D	0.78	14	53.4	151639.7	9965.6	6.9	2841.1	1014.1	37.6	121465.2	29700.3	24.6
UT-SCC-24B_Myogel-2D	0.65	9	28.1	60286.5	6242.7	10.9	2144.7	574.1	28.2	54159.2	5664.4	10.5
UT-SCC-24B_Myogel-3D	0.78	14	44.7	85469.9	5608.1	6.9	1913.9	488.5	26.9	74002.2	9971.2	13.6
UT-SCC-28_Control	0.73	12	23.6	40594.9	2910.4	7.6	1721.0	569.7	34.9	23917.0	19528.4	82.2
UT-SCC-28_Matrigel-2D	0.62	8	31.9	23178.2	2679.3	12.2	726.2	201.6	29.3	14451.0	11976.3	83.4
UT-SCC-28_Matrigel-3D	0.61	8	44.0	27467.7	3367.7	12.9	624.8	105.2	17.8	26802.2	4062.0	15.3
UT-SCC-28_Myogel-2D	0.75	14	22.8	54988.6	3517.9	6.7	2409.2	841.0	36.8	31066.9	25363.9	82.2
UT-SCC-28_Myogel-3D	0.59	7	49.3	46959.8	5893.1	13.2	952.9	335.3	37.1	28844.5	24294.6	84.8
UT-SCC-40_Control	0.72	12	26.6	130838.5	10104.3	8.1	4925.8	1477.9	31.6	122169.3	14706.0	12.1
UT-SCC-40_Matrigel-2D	0.76	13	33.4	143446.7	9726.2	7.1	4301.0	1317.4	32.3	136242.4	15533.0	11.5
UT-SCC-40_Matrigel-3D	0.82	17	58.6	185182.0	10421.9	5.9	3160.3	792.3	26.4	152268.0	28643.4	18.9

QC Summary:

Plate	Z_Prime	SSMD	Signal_Vs_BG	Mean_DMSO	SD_DMSO	CV_DMSO	Mean_BzCl	SD_BzCl	CV_BzCl	Mean_Cells	SD_Cells	CV_Cells
UT−SCC−40_Myogel−2D	0.77	15	27.3	118144.6	7271.9	6.5	4334.4	1435.1	34.9	100160.2	15487.6	15.6
UT−SCC−40_Myogel−3D	0.65	9	35.9	136799.7	14485.7	11.2	3814.2	1120.5	31.0	116320.4	19111.3	16.5
UT−SCC−42A_Control	0.74	12	25.6	44923.1	3317.2	7.8	1756.7	494.4	29.7	39500.2	3885.1	9.9
UT−SCC−42A_Matrigel−2D	0.80	16	35.5	65905.5	3810.2	6.1	1857.8	543.8	30.9	66308.5	6762.7	10.3
UT−SCC−42A_Matrigel−3D	0.56	7	53.3	83235.4	11520.2	14.6	1560.7	362.5	24.5	79112.8	10131.4	12.9
UT−SCC−42A_Myogel−2D	0.79	16	22.8	56193.3	3211.9	6.0	2467.1	590.4	25.2	51566.5	4621.0	9.0
UT−SCC−42A_Myogel−3D	0.75	12	43.2	60761.6	4542.3	7.9	1407.7	408.4	30.6	54713.7	8206.3	15.1
UT−SCC−42B_Control	0.79	16	23.2	81476.5	4424.3	5.7	3518.3	991.7	29.7	82019.3	6126.6	7.5
UT−SCC−42B_Matrigel−2D	0.79	15	31.5	85145.6	5241.3	6.5	2702.9	660.1	25.7	88538.5	8406.1	9.6
UT−SCC−42B_Matrigel−3D	0.73	11	48.7	114434.2	9579.6	8.8	2350.9	600.1	26.9	114206.4	7139.0	6.3
UT−SCC−42B_Myogel−2D	0.85	23	25.7	83350.5	3198.4	4.0	3241.4	813.9	26.5	82323.2	5737.4	7.0
UT−SCC−42B_Myogel−3D	0.79	15	38.3	110304.0	6749.7	6.5	2879.6	615.5	22.5	104734.6	9465.0	9.1
UT−SCC−44_Control	0.74	13	25.8	49137.1	3419.7	7.3	1902.6	694.8	38.5	26324.6	21790.3	83.3
UT−SCC−44_Matrigel−2D	0.67	10	36.1	26186.4	2515.4	10.1	725.3	253.5	36.8	13661.3	11278.5	83.1
UT−SCC−44_Matrigel−3D	0.57	7	47.0	52063.9	6863.4	13.9	1108.1	368.8	35.1	47355.0	10305.7	21.9
UT−SCC−44_Myogel−2D	0.59	8	25.4	44267.6	5138.5	12.2	1745.6	644.3	38.9	24351.7	20158.3	83.3
UT−SCC−44_Myogel−3D	0.72	11	37.5	42862.0	3455.2	8.5	1144.5	377.8	34.8	26631.3	22071.8	83.4
UT−SCC−73_Control	0.72	11	29.0	98840.7	7840.9	8.4	3403.1	955.2	29.6	92441.2	11584.3	12.6
UT−SCC−73_Matrigel−2D	0.84	20	36.4	174020.0	7849.8	4.8	4786.3	1191.3	26.2	153438.1	15344.3	10.1
UT−SCC−73_Matrigel−3D	0.77	13	53.3	177872.2	12358.9	7.3	3336.7	791.2	25.0	165495.4	16661.2	10.1
UT−SCC−73_Myogel−2D	0.78	15	25.7	117984.2	7211.4	6.4	4582.8	1118.0	25.7	102948.8	11238.4	11.0
UT−SCC−73_Myogel−3D	0.76	14	28.5	77649.6	5144.2	7.0	2727.4	870.5	33.6	75583.4	12228.8	16.3
UT−SCC−8_Control	0.65	9	32.6	96179.4	9989.8	10.9	2952.2	808.9	28.9	127958.3	15246.2	12.0
UT−SCC−8_Matrigel−2D	0.71	10	37.2	91434.7	8154.7	9.4	2459.1	406.2	17.4	115774.1	11314.9	9.8
UT−SCC−8_Matrigel−3D	0.74	11	52.0	157895.6	12904.6	8.6	3035.9	662.0	23.0	175337.9	21284.1	12.2
UT−SCC−8_Myogel−2D	0.59	7	33.8	71010.2	8956.3	13.3	2103.8	437.5	21.9	98255.7	12907.0	13.2
UT−SCC−8_Myogel−3D	0.48	6	34.0	41815.0	6635.5	16.7	1228.5	451.5	38.7	47124.0	12146.0	25.9
UT−SCC−81_Control	0.80	16	29.6	101928.5	5711.0	5.9	3445.1	927.8	28.4	92447.2	12923.7	14.1

QC Summary:

Plate	Z_Prime	SSMD	Signal_Vs_BG	Mean_DMSO	SD_DMSO	CV_DMSO	Mean_BzCl	SD_BzCl	CV_BzCl	Mean_Cells	SD_Cells	CV_Cells
UT−SCC−81_Matrigel−2D	0.77	13	38.5	129109.5	8876.6	7.2	3351.1	942.4	29.6	115134.5	13812.3	12.1
UT−SCC−81_Matrigel−3D	0.41	5	39.4	167197.9	31069.0	19.6	4243.8	1153.6	28.7	154083.1	29626.6	19.4
UT−SCC−81_Myogel−2D	0.79	16	25.3	101680.4	5843.8	6.1	4013.5	1150.4	30.2	92773.5	10061.0	10.9
UT−SCC−81_Myogel−3D	0.48	6	34.7	96046.6	15460.2	17.0	2770.9	635.0	24.2	99982.1	12515.9	12.6

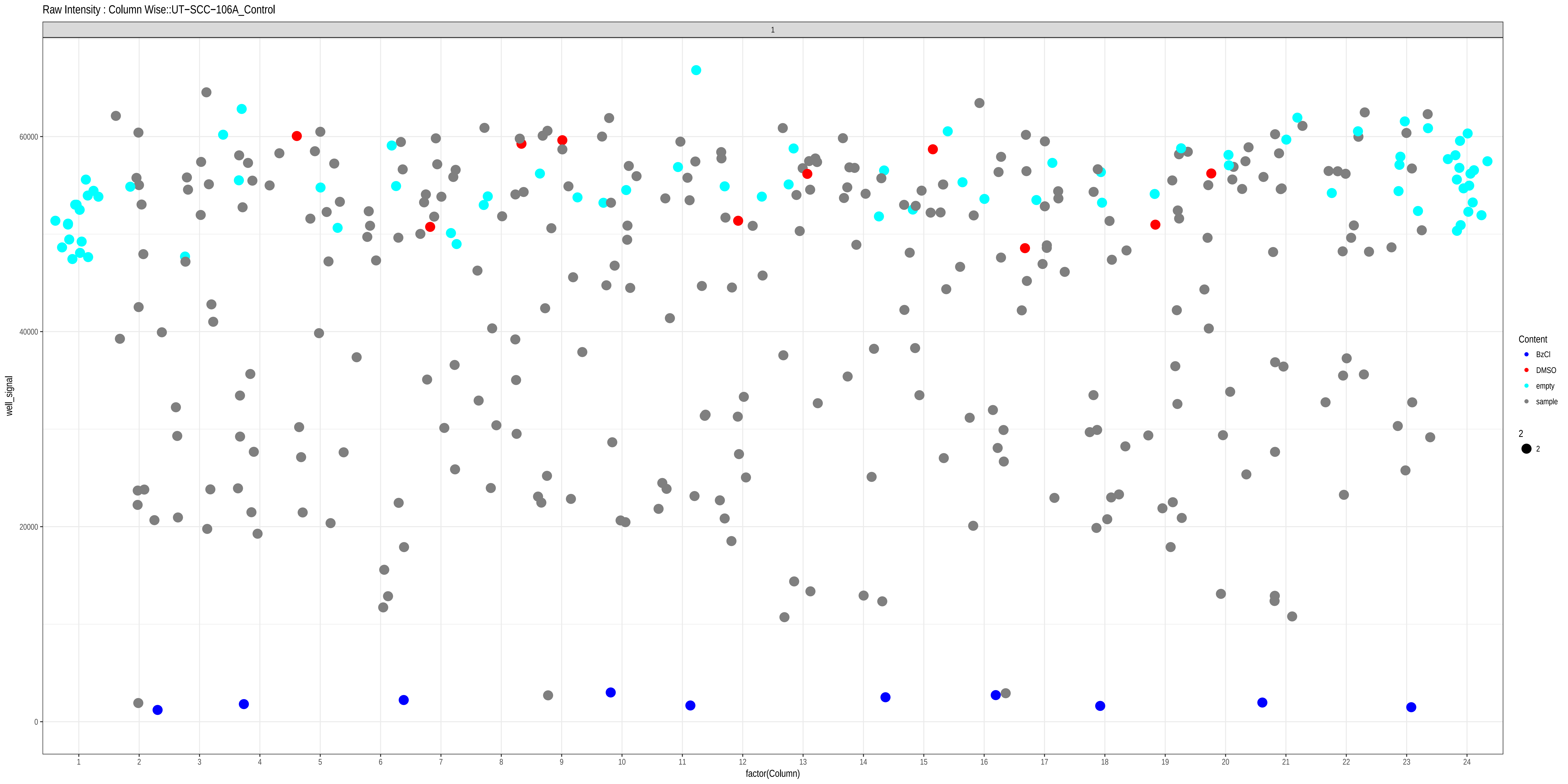
Raw Intensity : Column Wise::UT−SCC−106A_Control
1
well_signal
60000
40000
20000
0
factor(Column)
1
2
3
4
5
6
7
8
9
10
11
12
13
14
15
16
17
18
19
20
21
22
23
24
Content
BzCl
DMSO
empty
sample
2
2

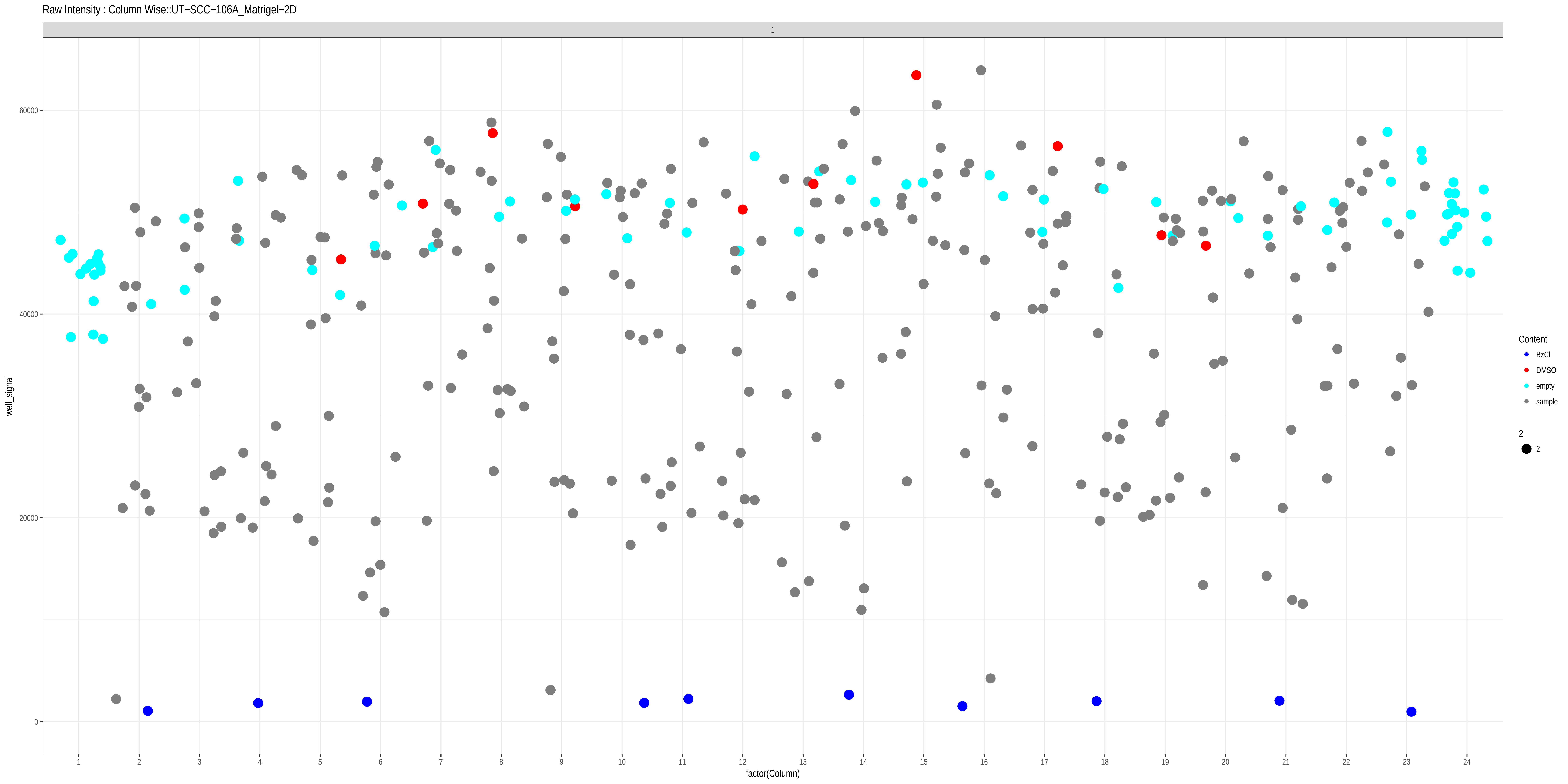

Raw Intensity : Column Wise::UT−SCC−106A_Matrigel−2D
1
well_signal
60000
40000
20000
0
1 2 3 4 5 6 7 8 9 10 11 12 13 14 15 16 17 18 19 20 21 22 23 24
factor(Column)
Content
BzCl
DMSO
empty
sample
2
2

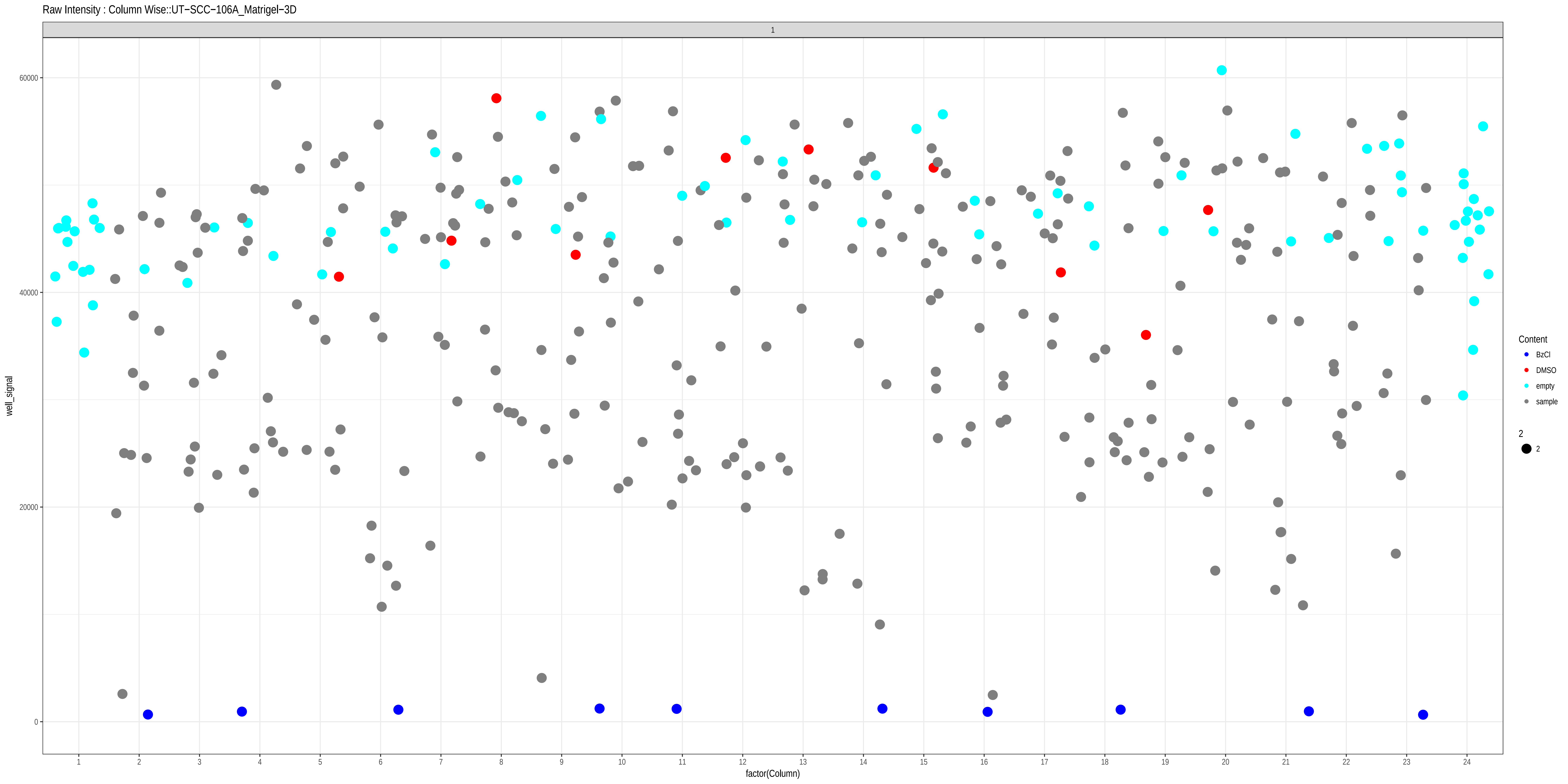
Raw Intensity : Column Wise::UT−SCC−106A_Matrigel−3D
1
60000
40000
20000
0
well_signal
1
2
3
4
5
6
7
8
9
10
11
12
13
14
15
16
17
18
19
20
21
22
23
24
factor(Column)
Content
BzCl
DMSO
empty
sample
2
2

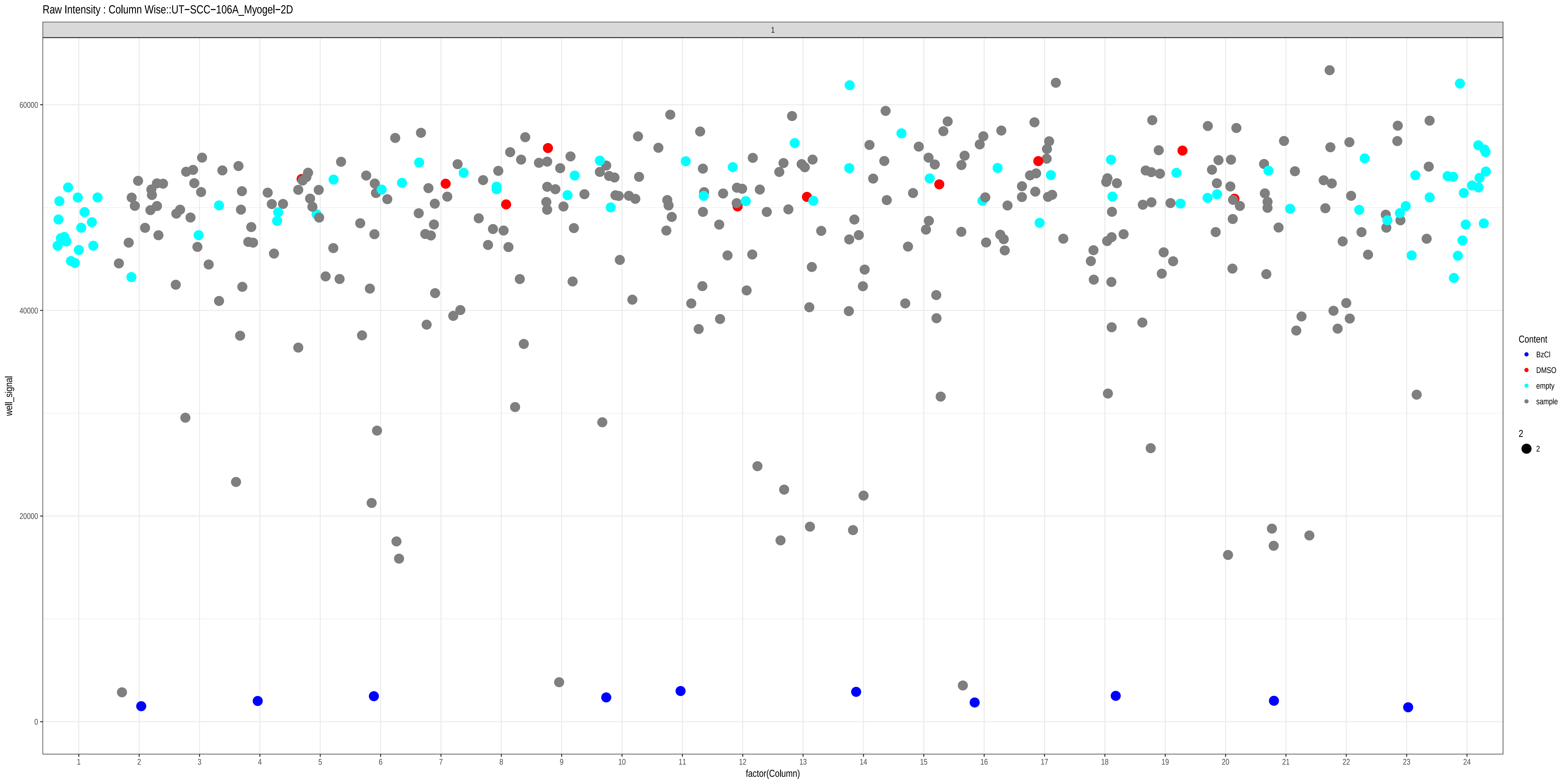
Raw Intensity : Column Wise::UT−SCC−106A_Myogel−2D
1
well_signal
60000
40000
20000
0
factor(Column)
1
2
3
4
5
6
7
8
9
10
11
12
13
14
15
16
17
18
19
20
21
22
23
24
Content
BzCl
DMSO
empty
sample
2
2

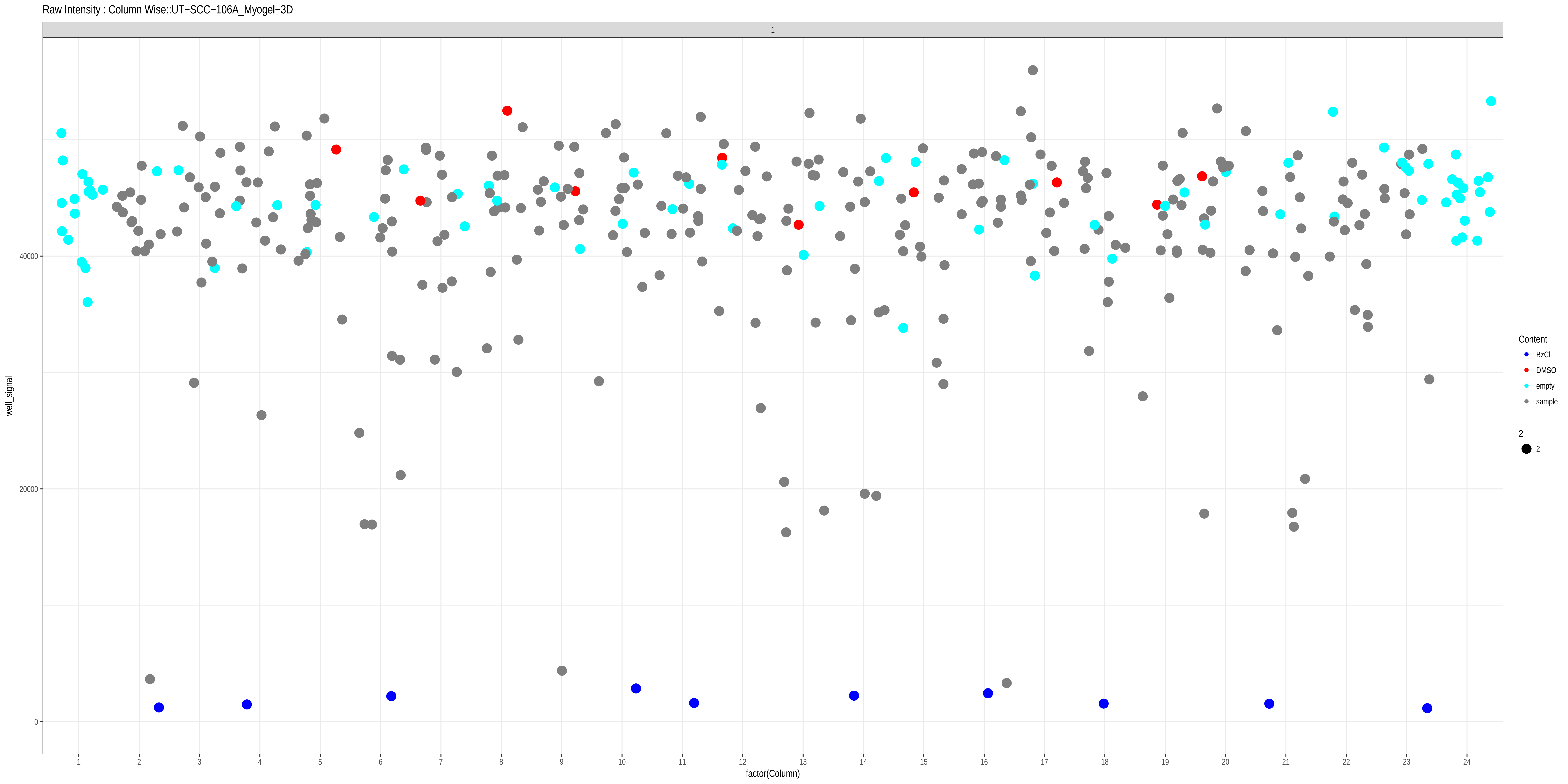
Raw Intensity : Column Wise::UT−SCC−106A_Myogel−3D
1
well_signal
40000
20000
0
1
2
3
4
5
6
7
8
9
10
11
12
13
14
15
16
17
18
19
20
21
22
23
24
factor(Column)
Content
BzCl
DMSO
empty
sample
2
2

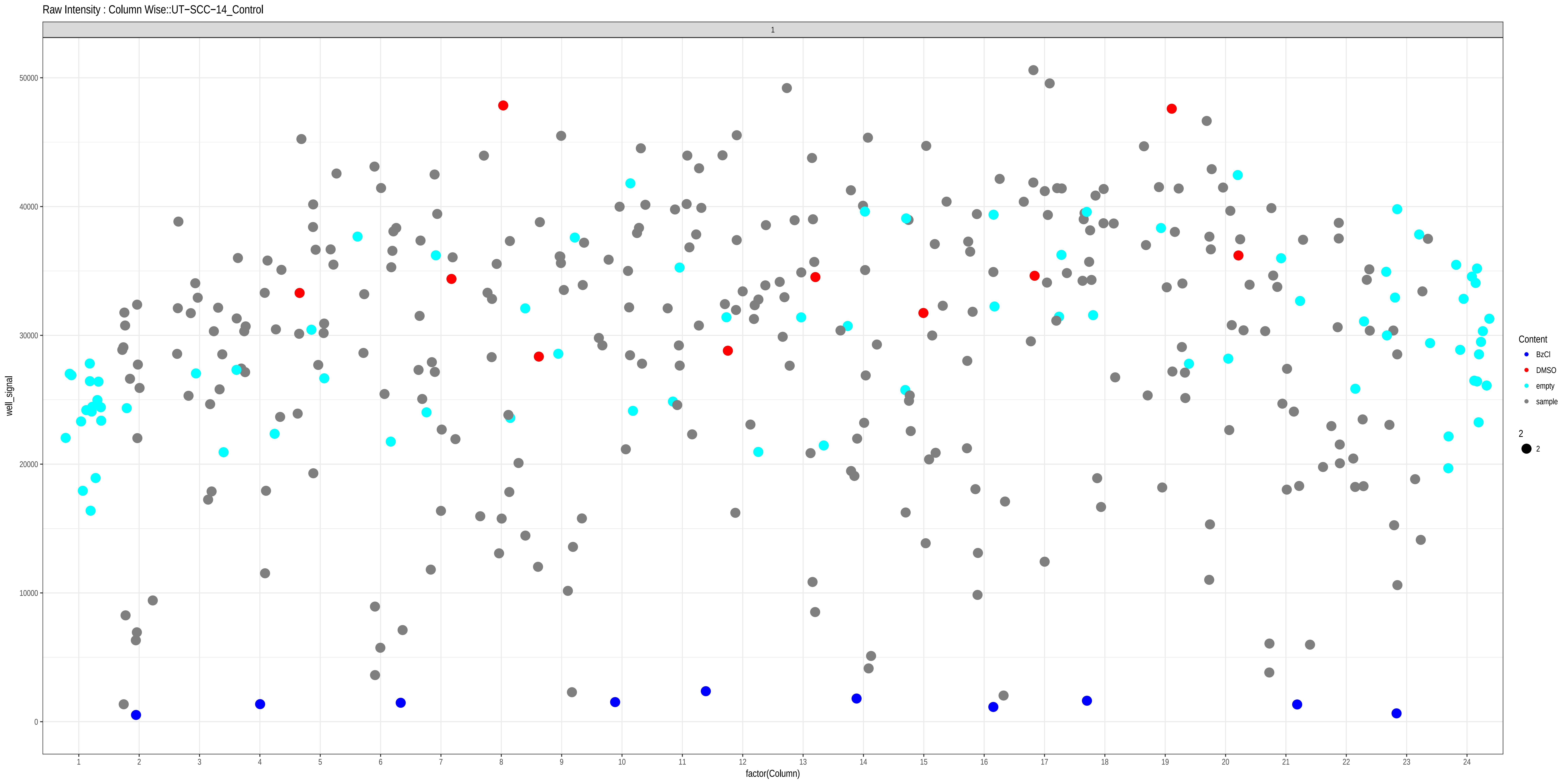

Raw Intensity : Column Wise::UT−SCC−14_Control
1
well_signal
0
10000
20000
30000
40000
50000
factor(Column)
1
2
3
4
5
6
7
8
9
10
11
12
13
14
15
16
17
18
19
20
21
22
23
24
Content
BzCl
DMSO
empty
sample
2
2

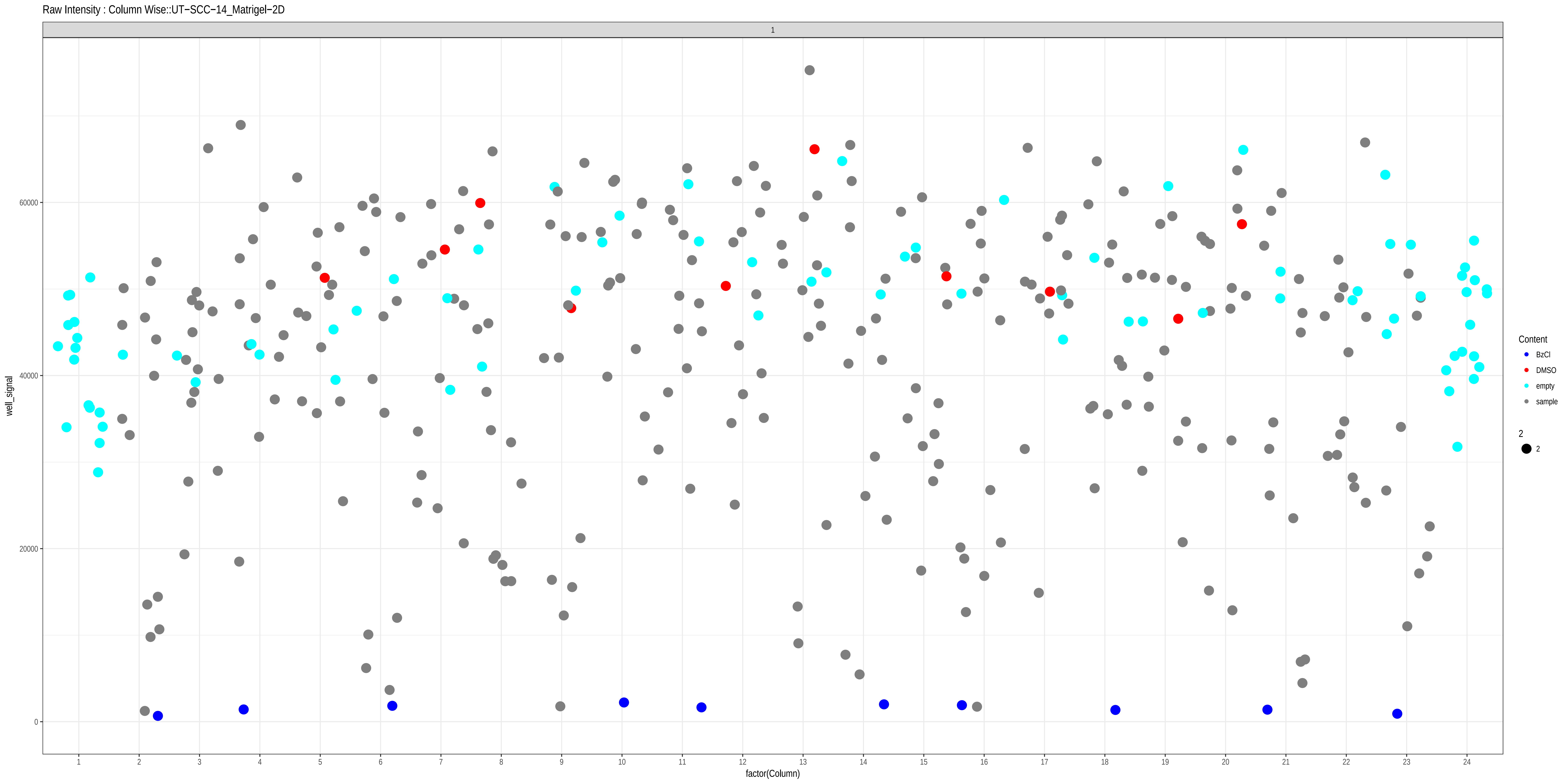
Raw Intensity : Column Wise::UT−SCC−14_Matrigel−2D
1
well_signal
60000
40000
20000
0
factor(Column)
1
2
3
4
5
6
7
8
9
10
11
12
13
14
15
16
17
18
19
20
21
22
23
24
Content
BzCl
DMSO
empty
sample
2
2

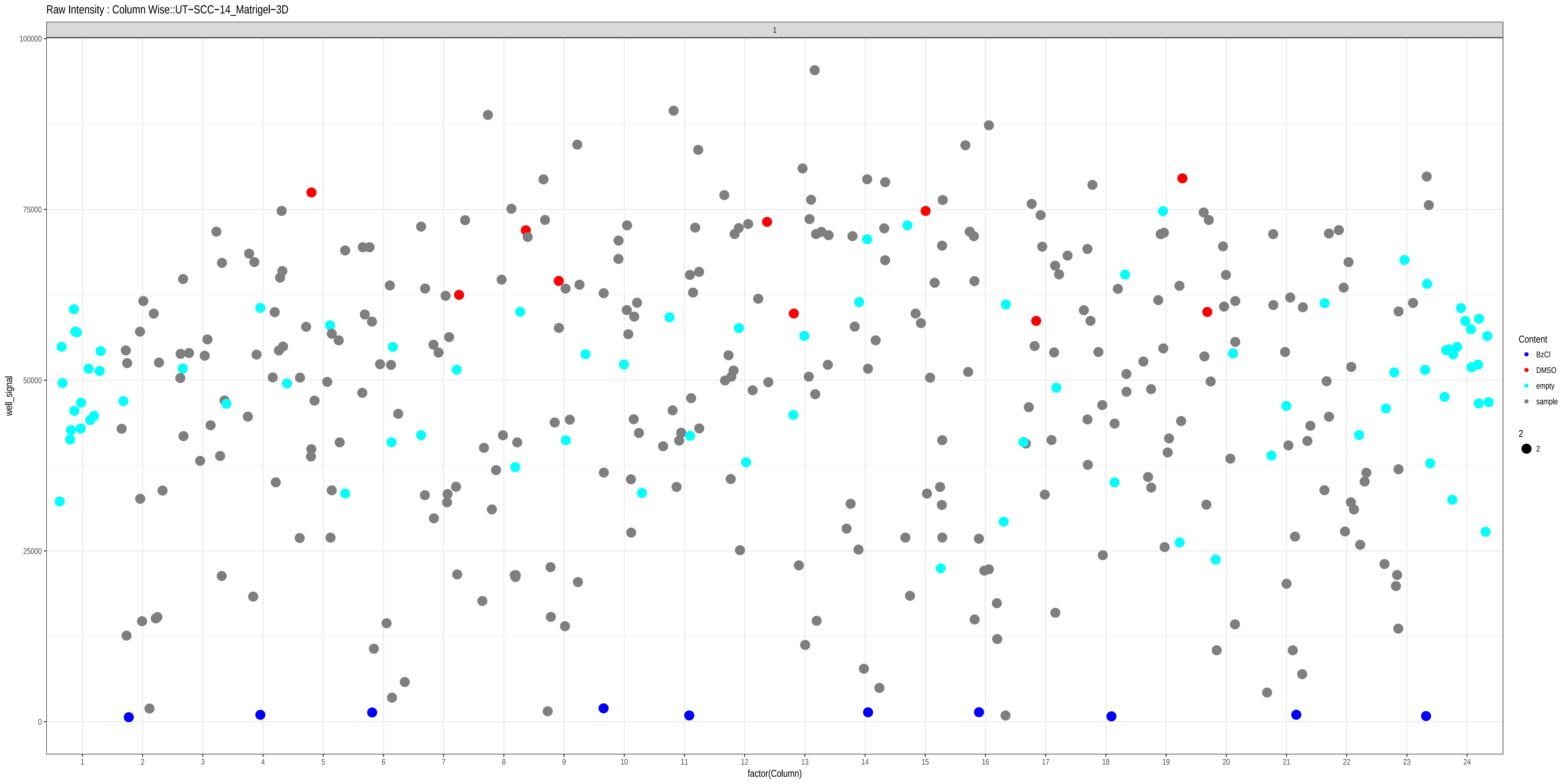
Raw Intensity : Column Wise::UT−SCC−14_Matrigel−3D
1
100000
75000
50000
25000
0
well_signal
1
2
3
4
5
6
7
8
9
10
11
12
13
14
15
16
17
18
19
20
21
22
23
24
factor(Column)
Content
BzCl
DMSO
empty
sample
2
2

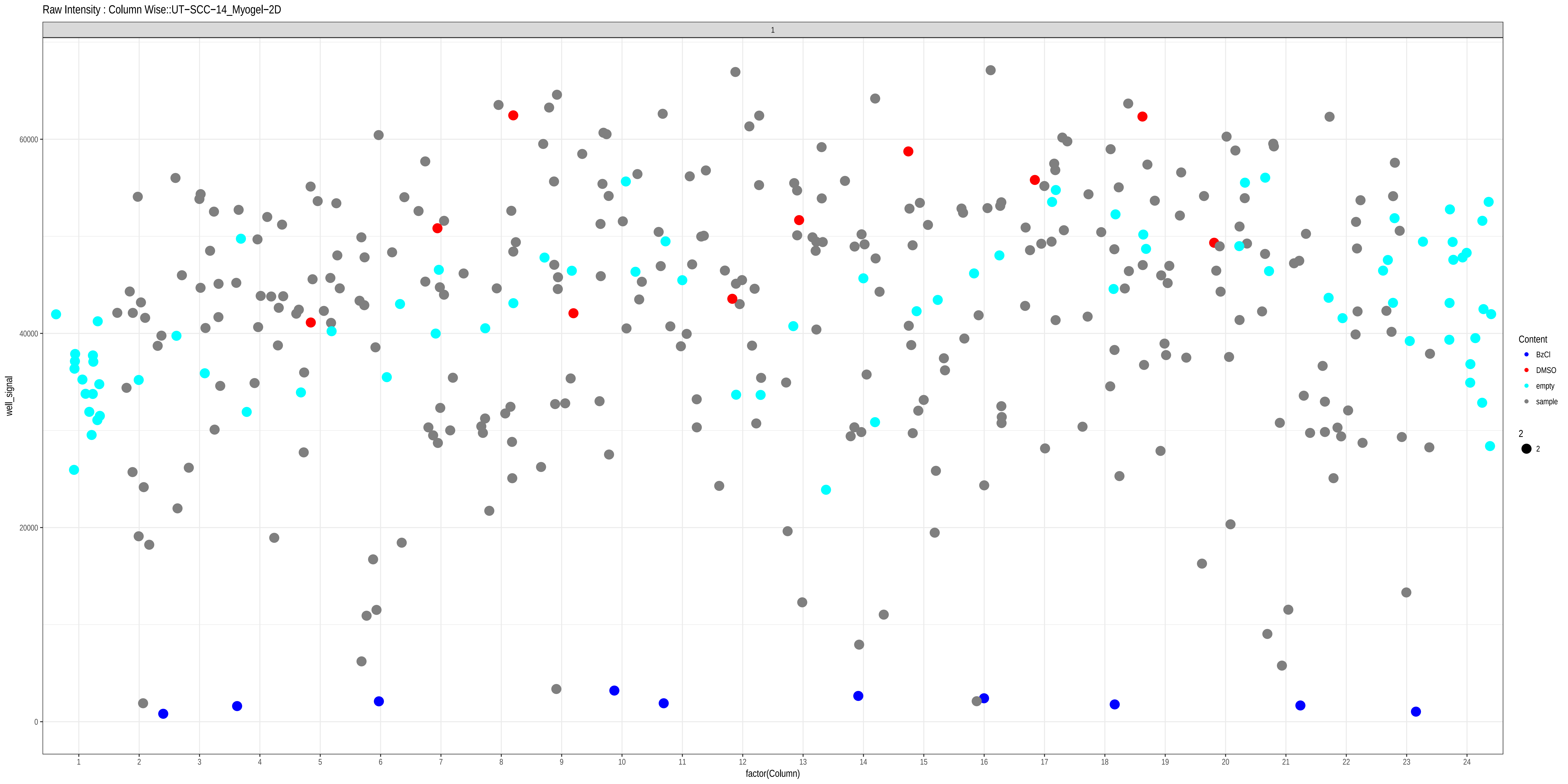
Raw Intensity : Column Wise::UT−SCC−14_Myogel−2D
1
60000
40000
20000
0
well_signal
1
2
3
4
5
6
7
8
9
10
11
12
13
14
15
16
17
18
19
20
21
22
23
24
factor(Column)
Content
BzCl
DMSO
empty
sample
2
2

Raw Intensity : Column Wise::UT−SCC−14_Myogel−3D

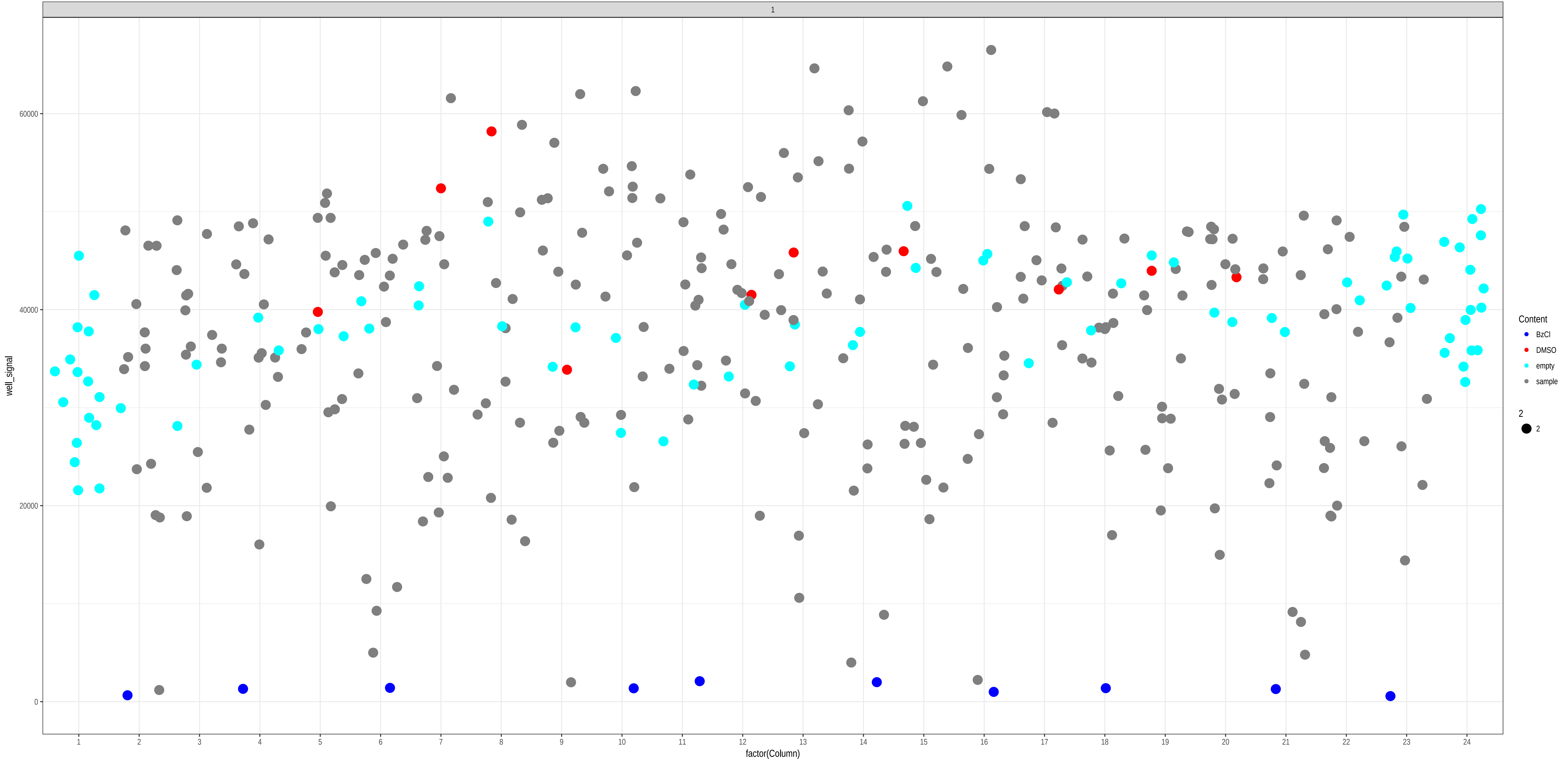

Raw Intensity : Column Wise::UT−SCC−24A_Control

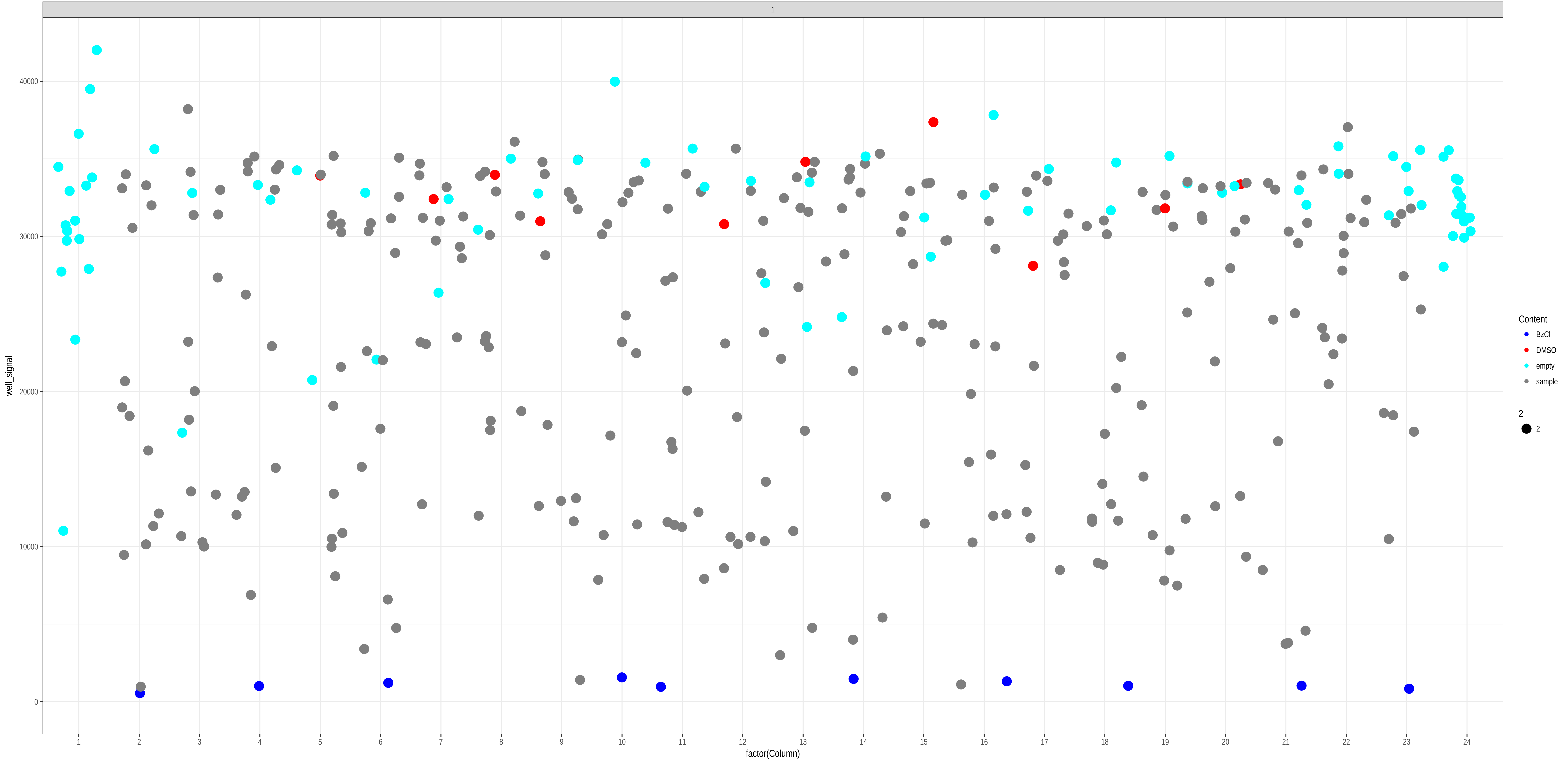

Raw Intensity : Column Wise::UT−SCC−24A_Matrigel−2D

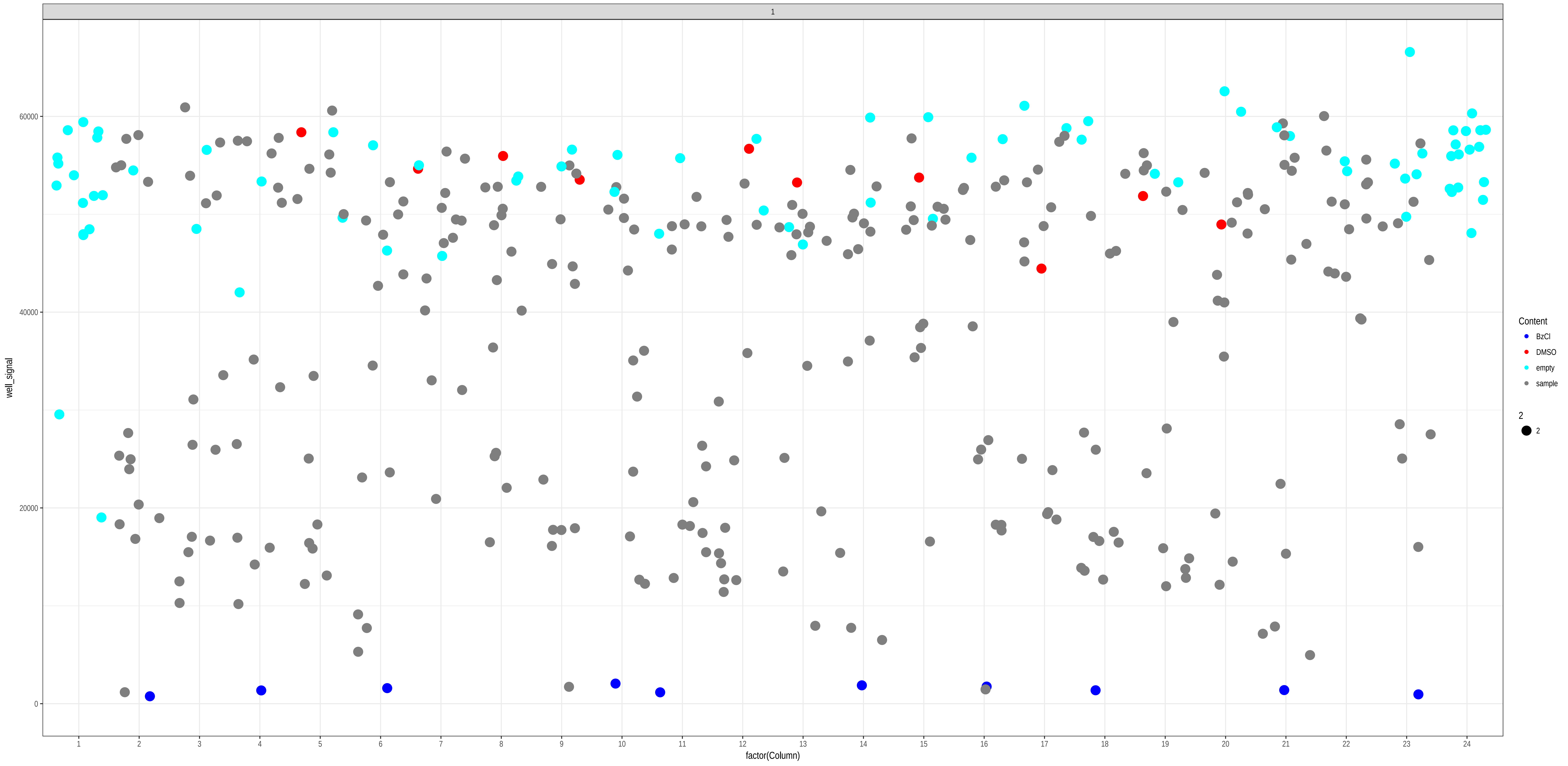

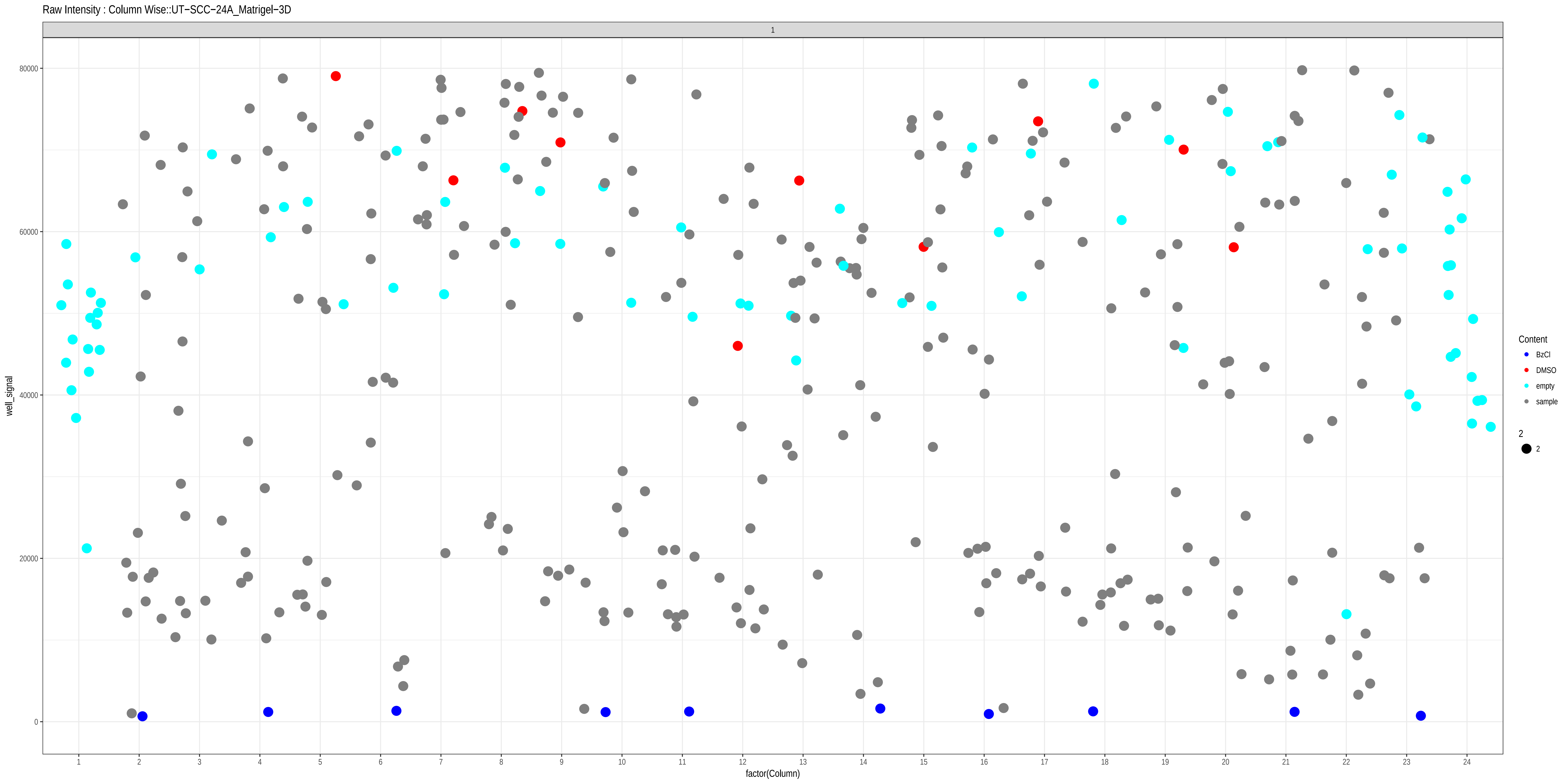
Raw Intensity : Column Wise::UT−SCC−24A_Matrigel−3D
1
80000
60000
40000
20000
0
well_signal
1 2 3 4 5 6 7 8 9 10 11 12 13 14 15 16 17 18 19 20 21 22 23 24
factor(Column)
Content
BzCl
DMSO
empty
sample
2
2

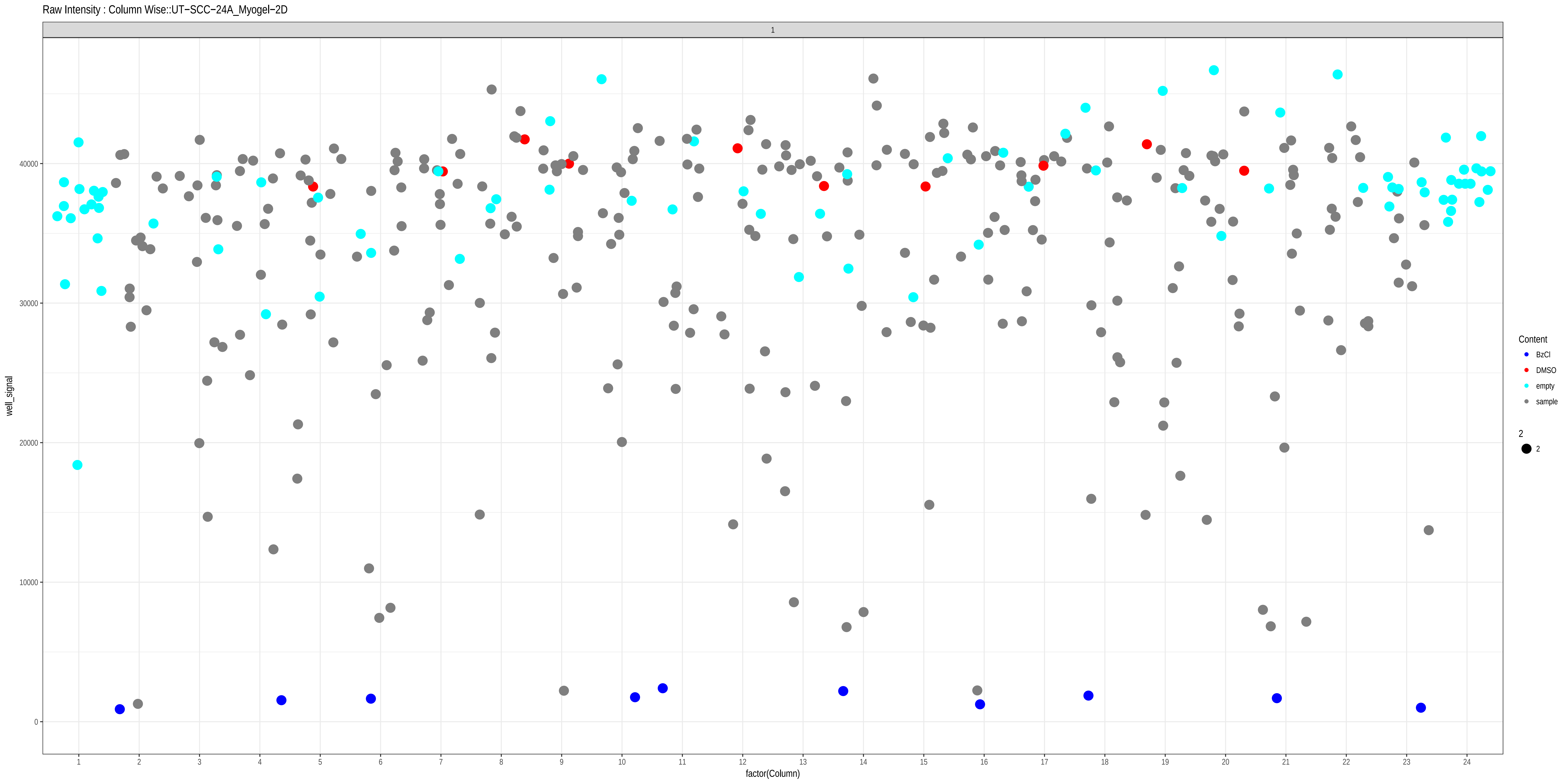

Raw Intensity : Column Wise::UT−SCC−24A_Myogel−2D
1
well_signal
0
10000
20000
30000
40000
factor(Column)
1
2
3
4
5
6
7
8
9
10
11
12
13
14
15
16
17
18
19
20
21
22
23
24
Content
BzCl
DMSO
empty
sample
2
2

Raw Intensity : Column Wise::UT−SCC−24A_Myogel−3D

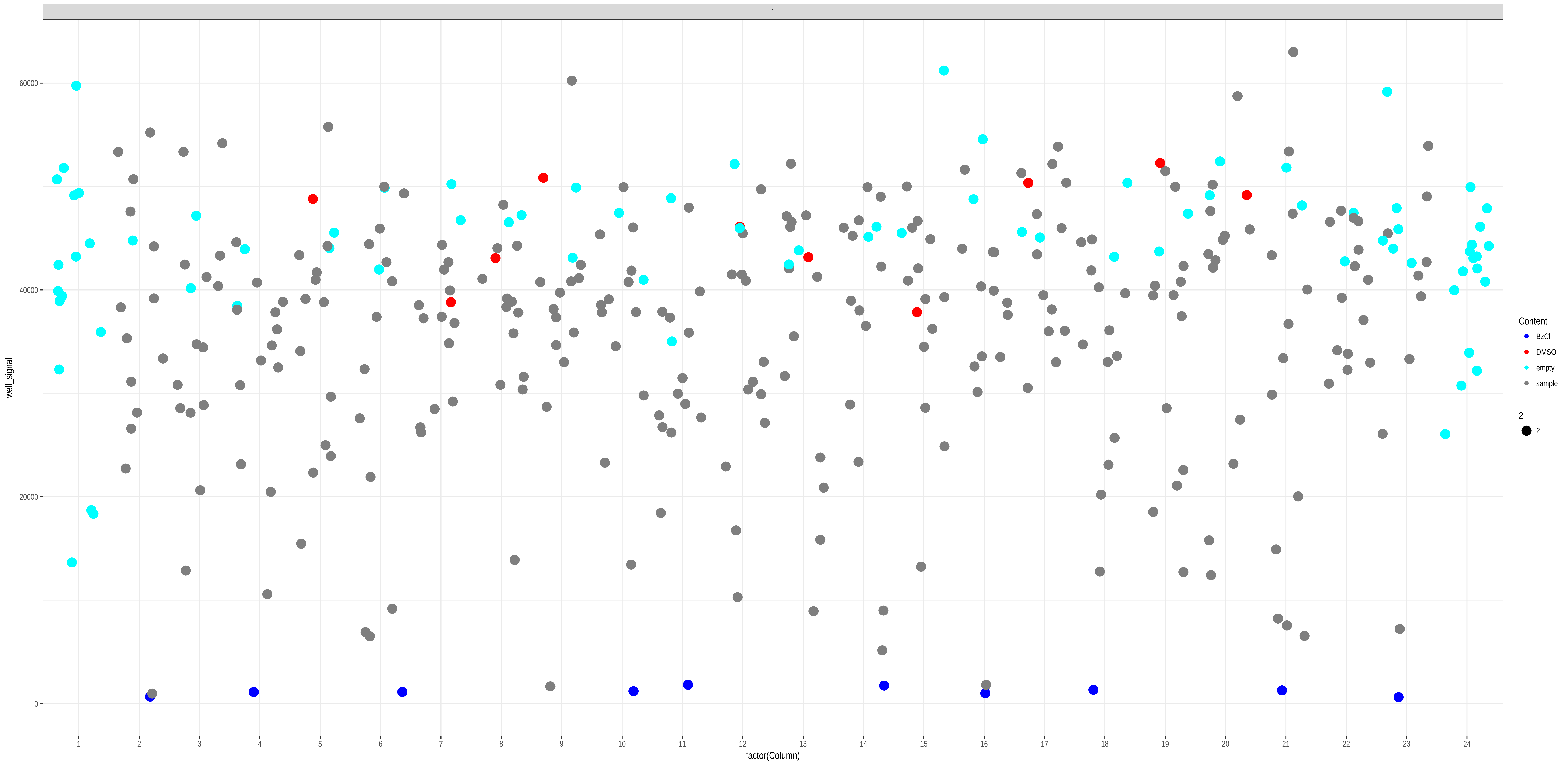

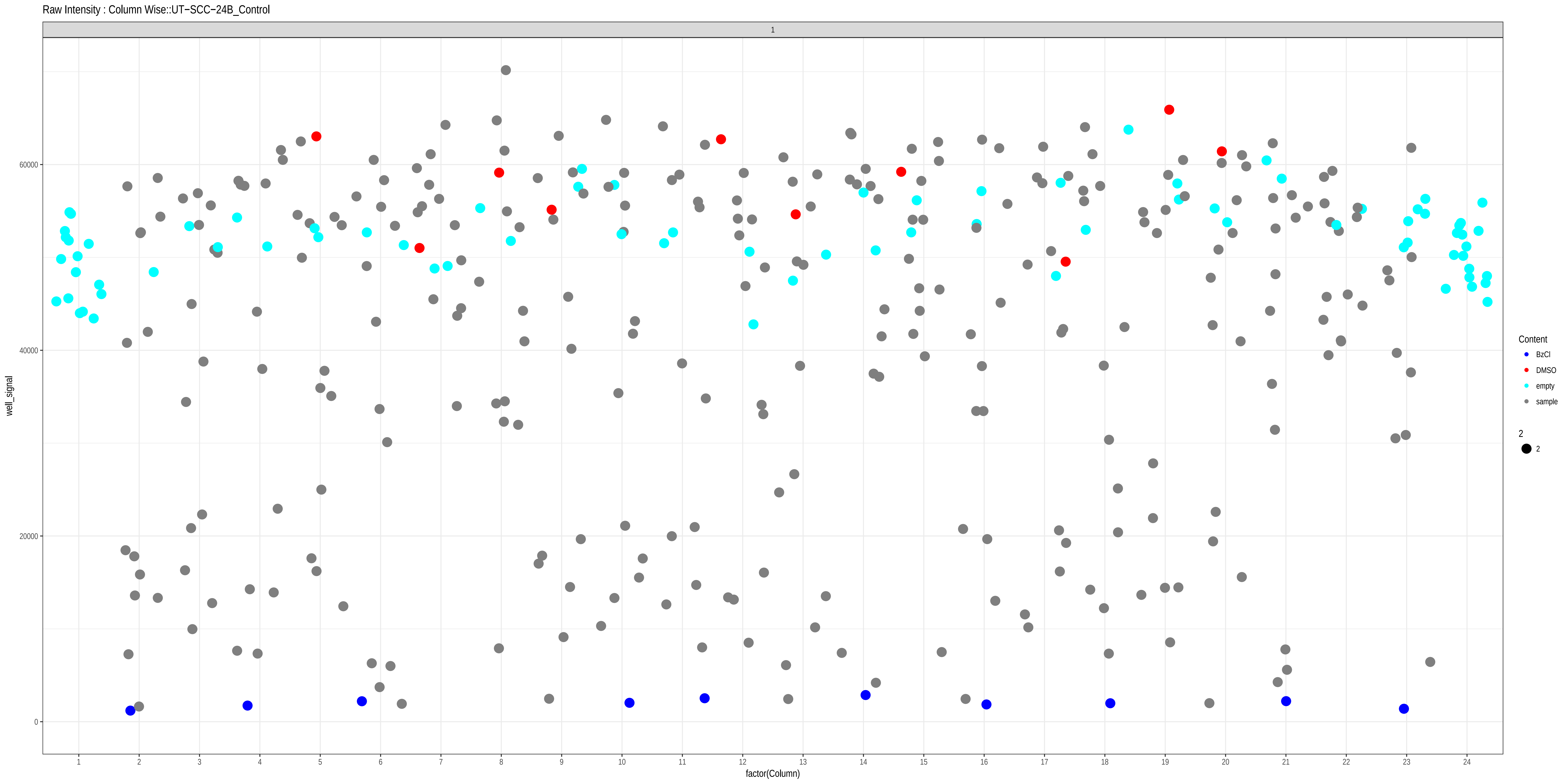
Raw Intensity : Column Wise::UT−SCC−24B_Control
1
well_signal
60000
40000
20000
0
factor(Column)
1
2
3
4
5
6
7
8
9
10
11
12
13
14
15
16
17
18
19
20
21
22
23
24
Content
BzCl
DMSO
empty
sample
2
2

Raw Intensity : Column Wise::UT−SCC−24B_Matrigel−2D

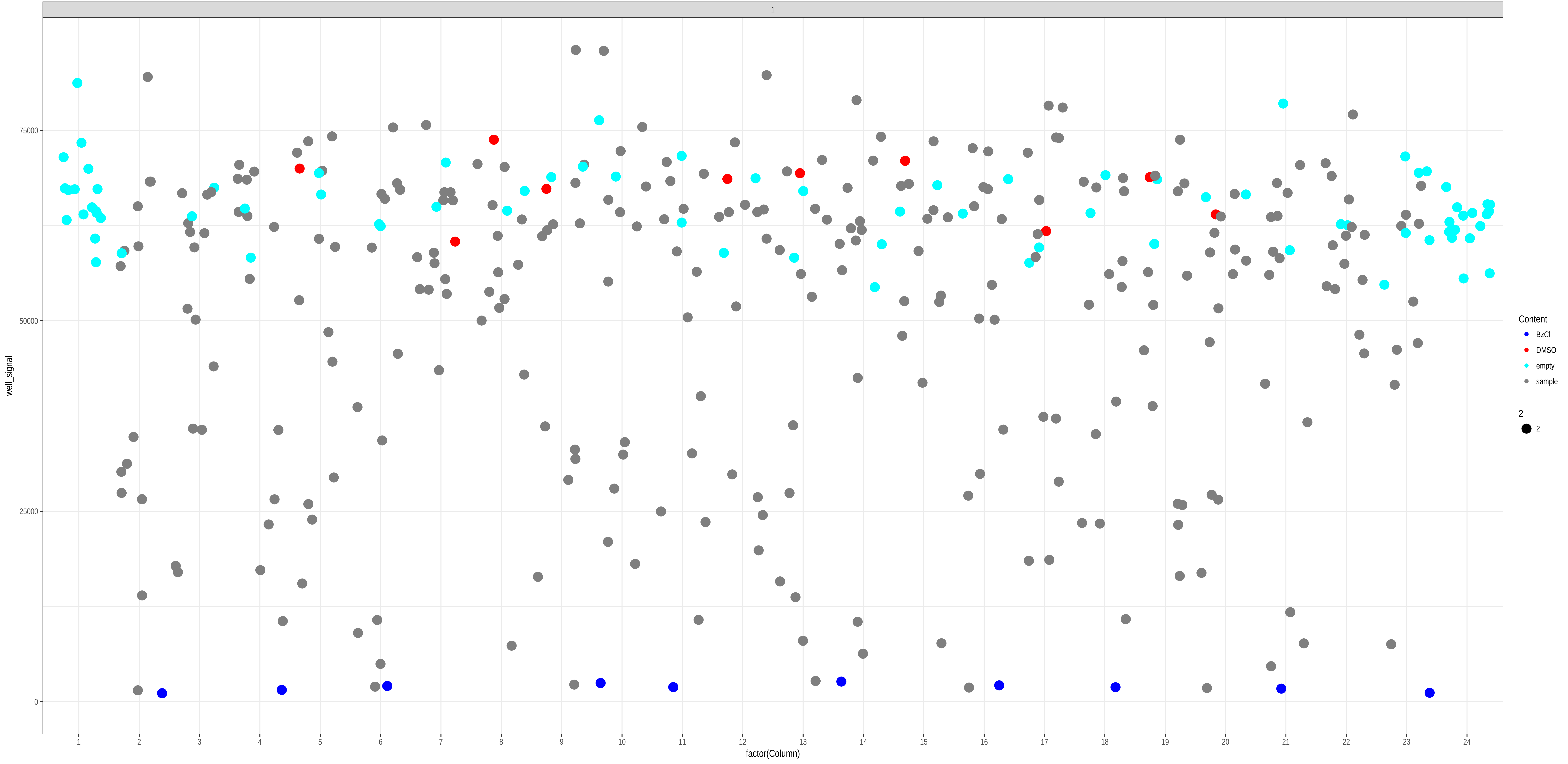

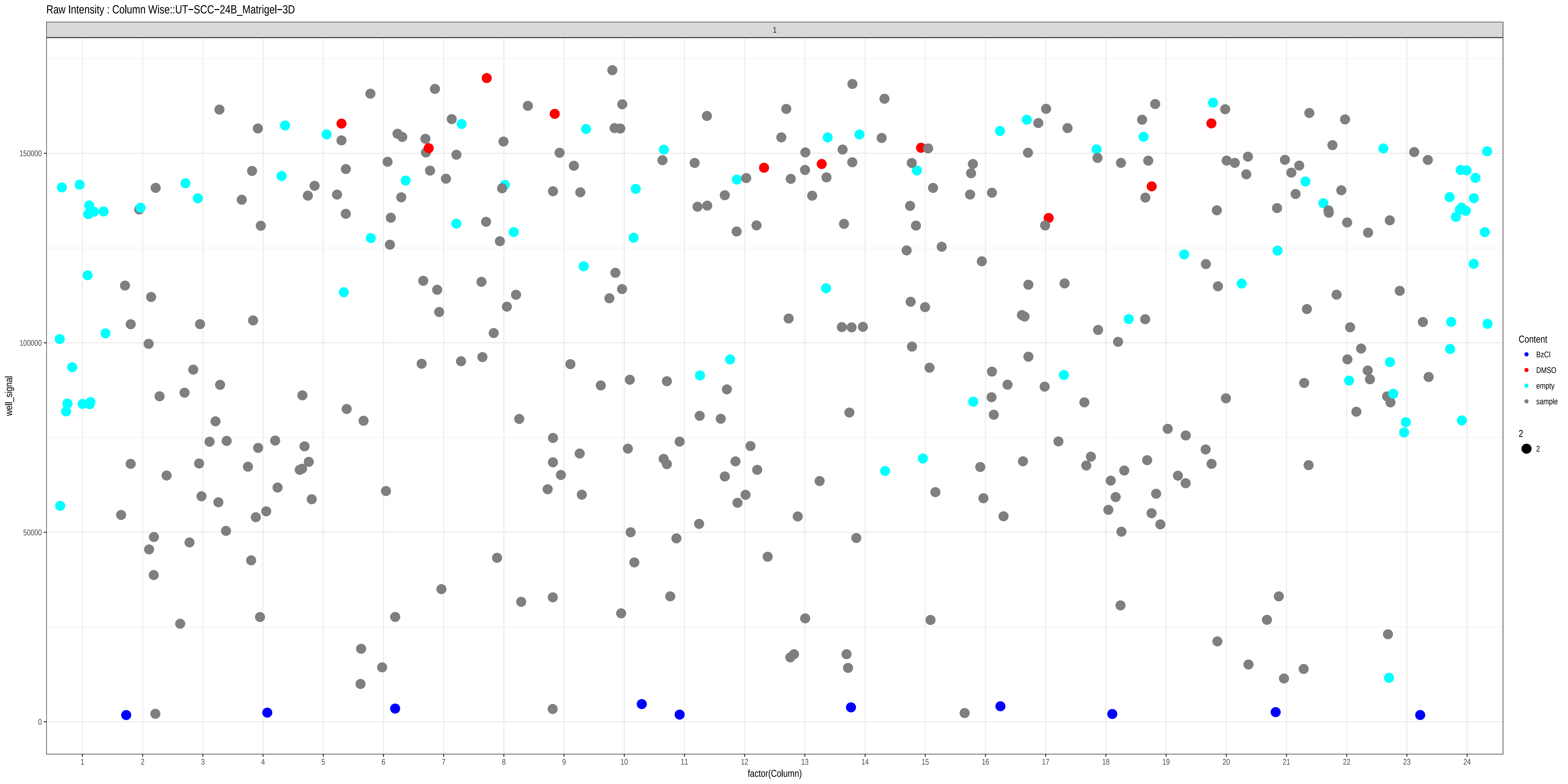

Raw Intensity : Column Wise::UT−SCC−24B_Matrigel−3D
1
well_signal
150000
100000
50000
0
1
2
3
4
5
6
7
8
9
10
11
12
13
14
15
16
17
18
19
20
21
22
23
24
factor(Column)
Content
BzCl
DMSO
empty
sample
2
2

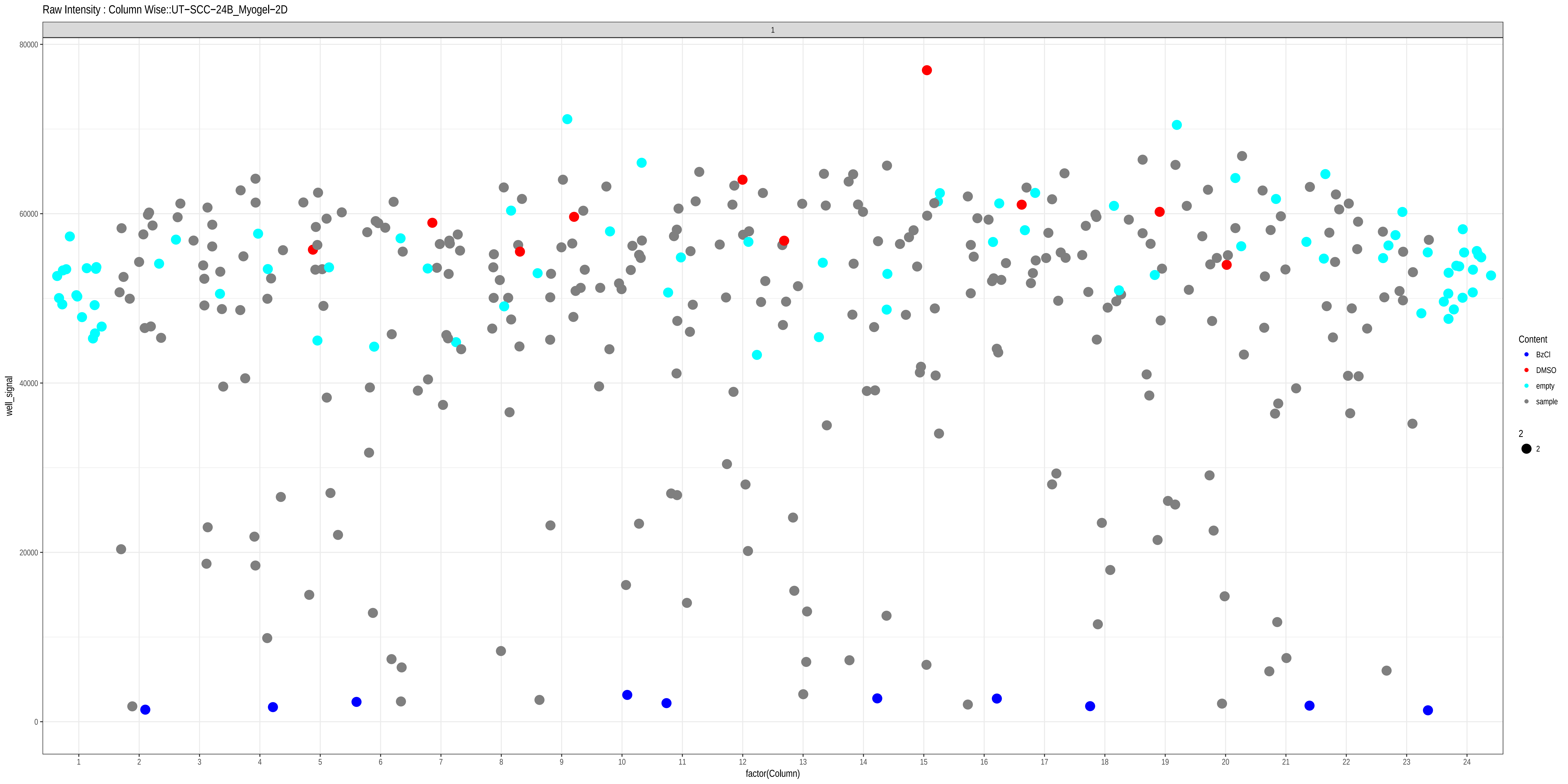

Raw Intensity : Column Wise::UT−SCC−24B_Myogel−2D
1
80000
60000
40000
20000
0
well_signal
1
2
3
4
5
6
7
8
9
10
11
12
13
14
15
16
17
18
19
20
21
22
23
24
factor(Column)
Content
BzCl
DMSO
empty
sample
2
2

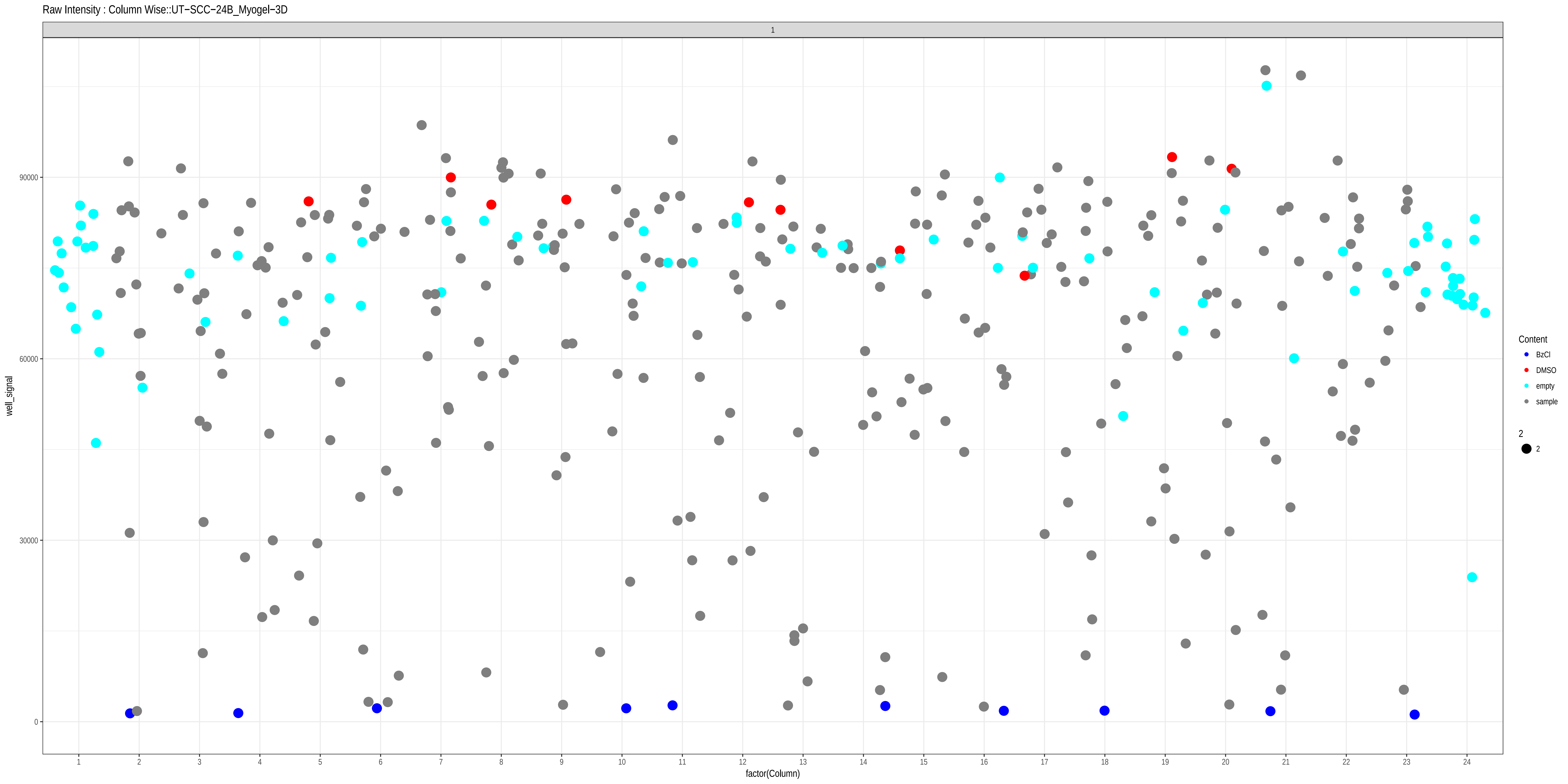
Raw Intensity : Column Wise::UT−SCC−24B_Myogel−3D
1
well_signal
90000
60000
30000
0
factor(Column)
1
2
3
4
5
6
7
8
9
10
11
12
13
14
15
16
17
18
19
20
21
22
23
24
Content
BzCl
DMSO
empty
sample
2
2

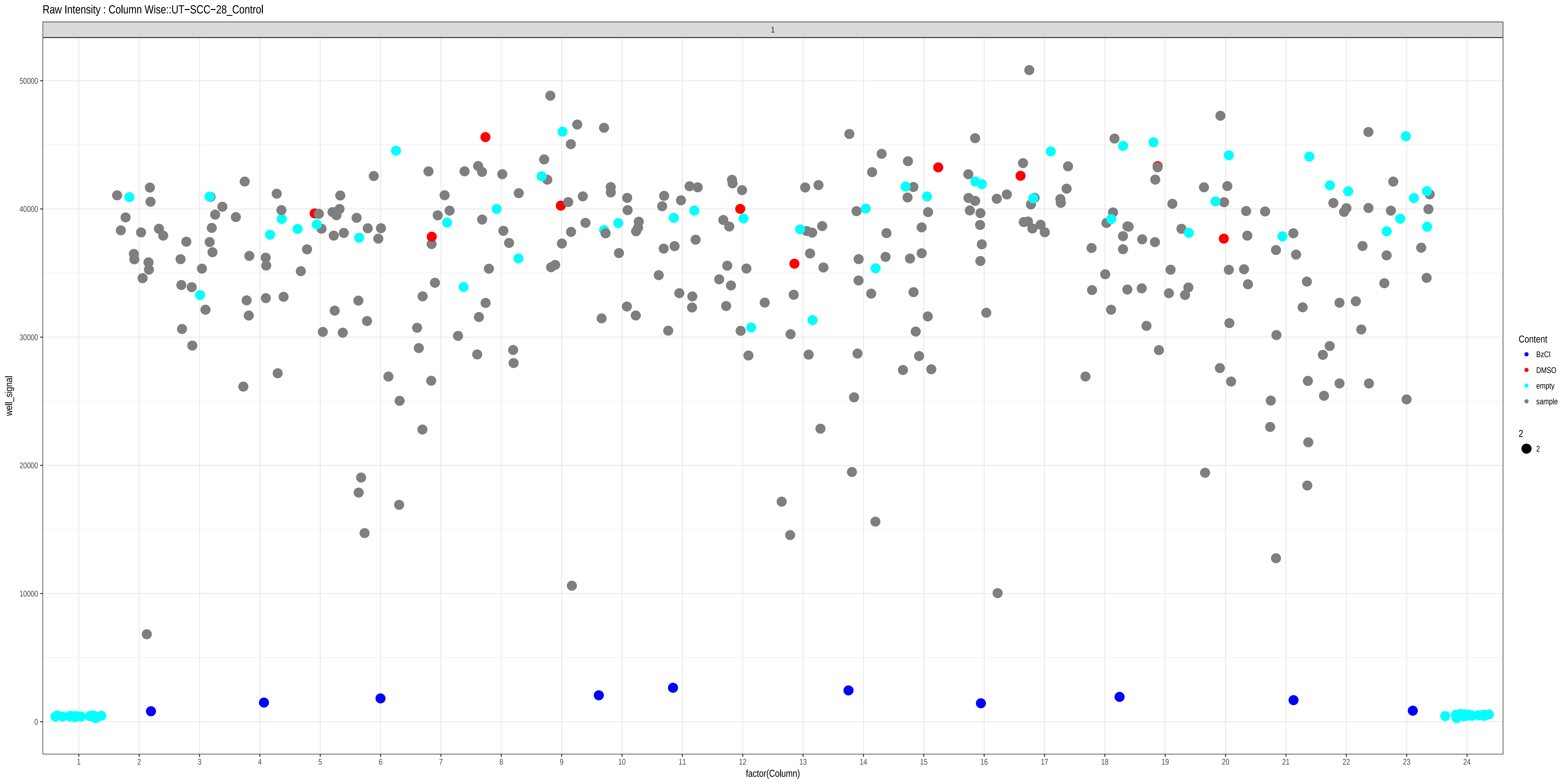
Raw Intensity : Column Wise::UT−SCC−28_Control
1
50000
40000
30000
20000
10000
0
well_signal
1 2 3 4 5 6 7 8 9 10 11 12 13 14 15 16 17 18 19 20 21 22 23 24
factor(Column)
Content
BzCl
DMSO
empty
sample
2
2

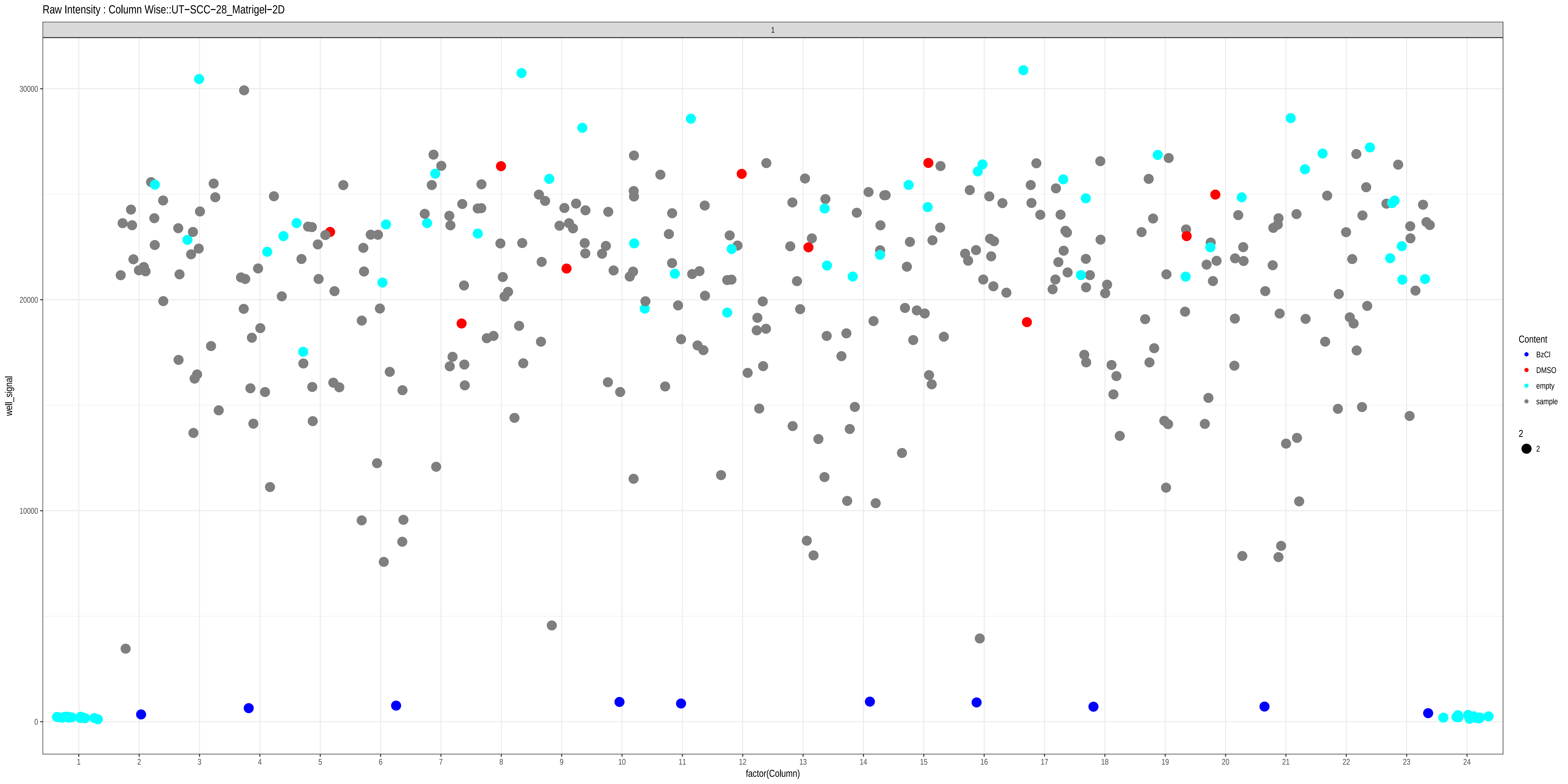

Raw Intensity : Column Wise::UT−SCC−28_Matrigel−2D
1
well_signal
30000
20000
10000
0
1 2 3 4 5 6 7 8 9 10 11 12 13 14 15 16 17 18 19 20 21 22 23 24
factor(Column)
Content
BzCl
DMSO
empty
sample
2
2

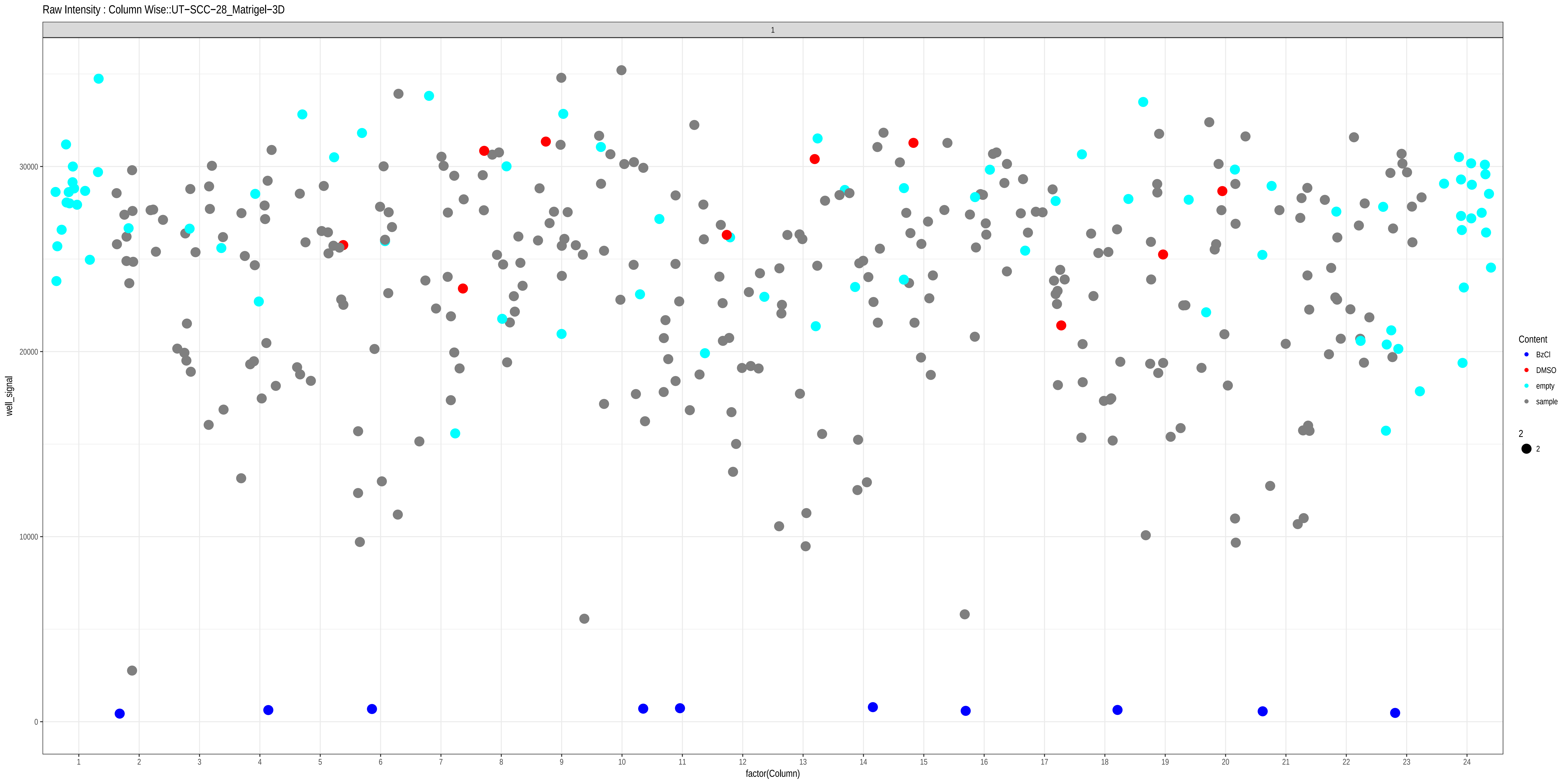

Raw Intensity : Column Wise::UT−SCC−28_Matrigel−3D
1
well_signal
factor(Column)
0
10000
20000
30000
1
2
3
4
5
6
7
8
9
10
11
12
13
14
15
16
17
18
19
20
21
22
23
24
Content
BzCl
DMSO
empty
sample
2
2

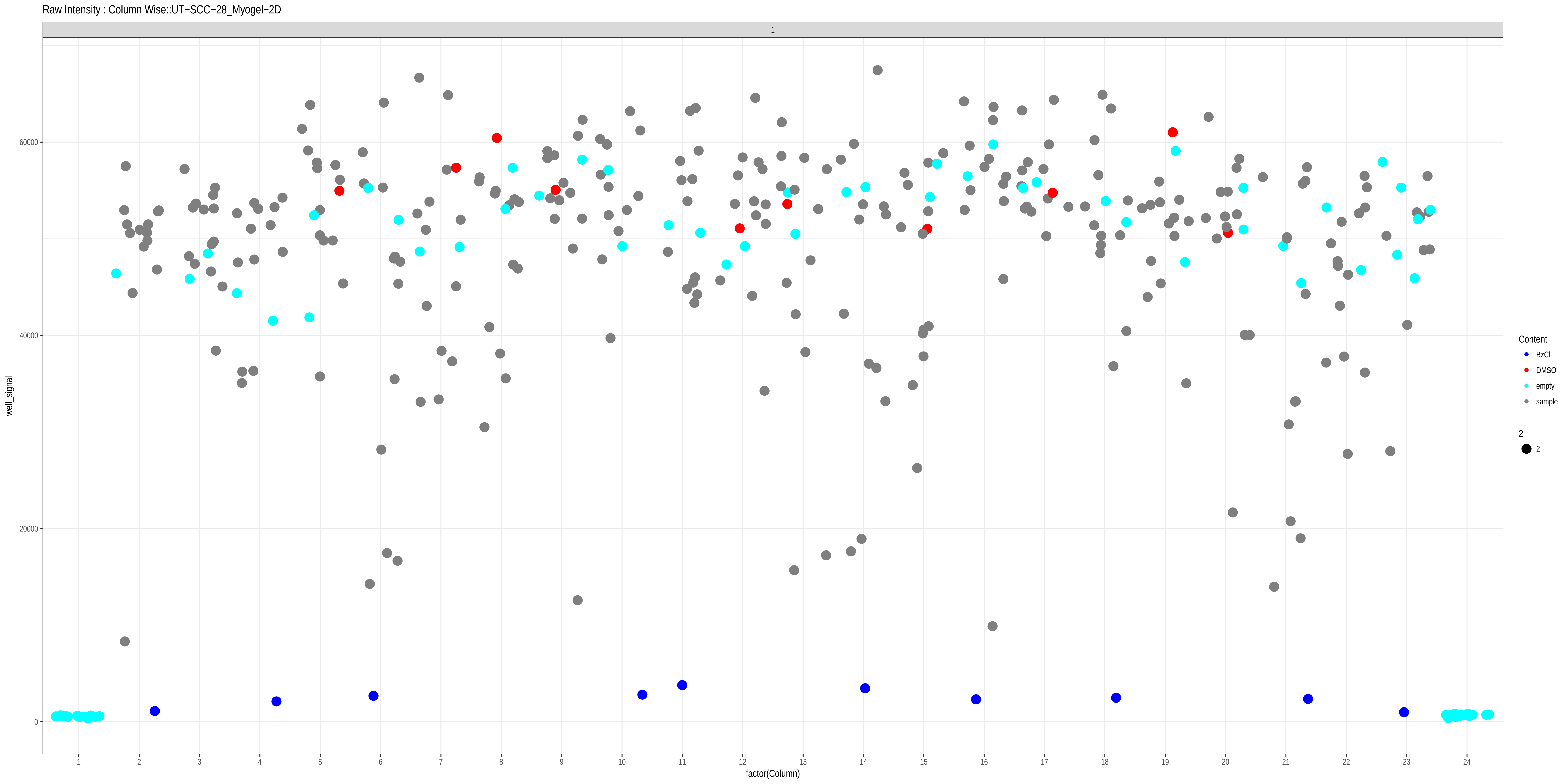

Raw Intensity : Column Wise::UT−SCC−28_Myogel−2D
1
well_signal
60000
40000
20000
0
factor(Column)
1
2
3
4
5
6
7
8
9
10
11
12
13
14
15
16
17
18
19
20
21
22
23
24
Content
BzCl
DMSO
empty
sample
2
2

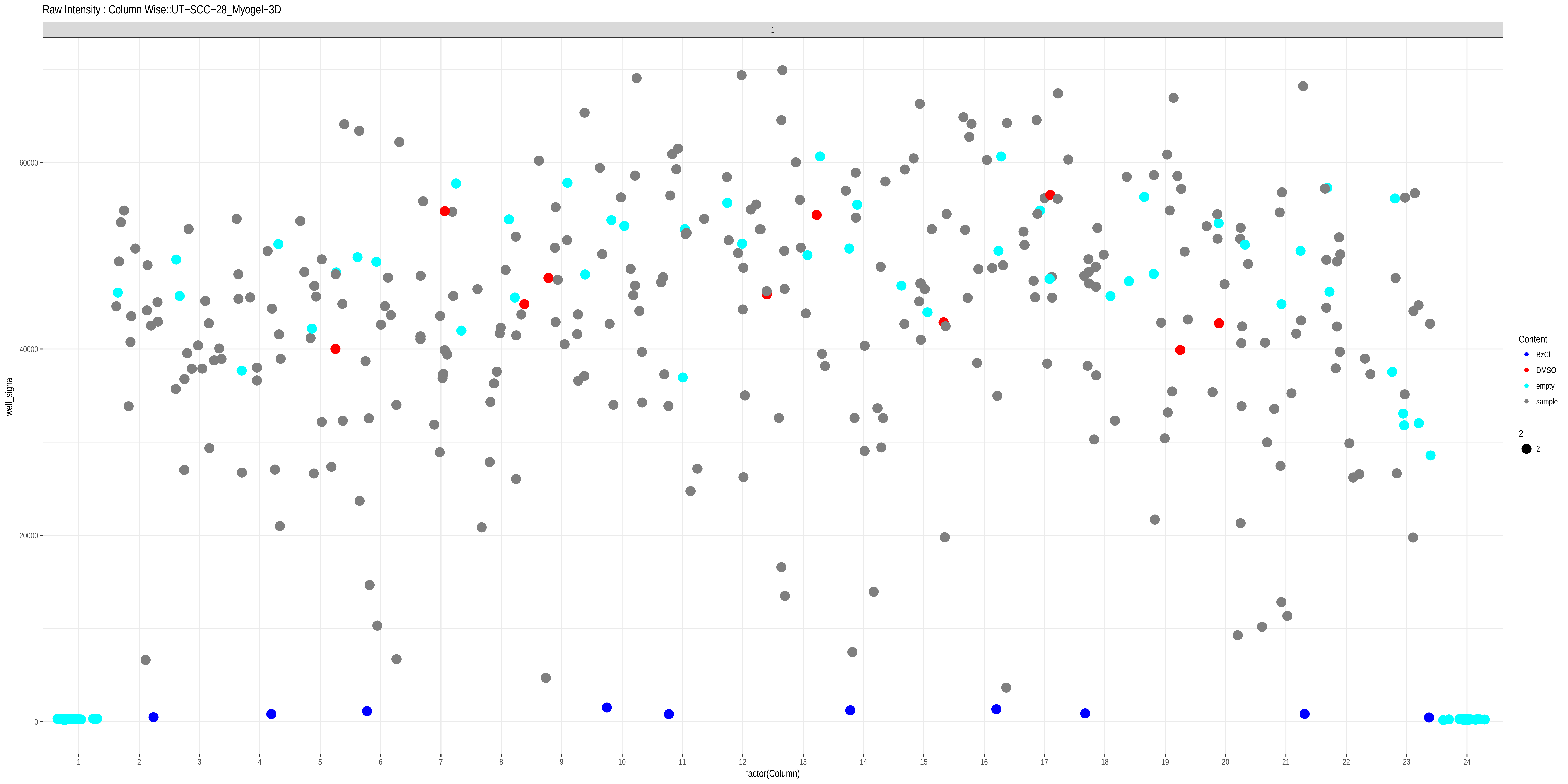

Raw Intensity : Column Wise::UT−SCC−28_Myogel−3D
1
well_signal
60000
40000
20000
0
factor(Column)
1
2
3
4
5
6
7
8
9
10
11
12
13
14
15
16
17
18
19
20
21
22
23
24
Content
BzCl
DMSO
empty
sample
2
2

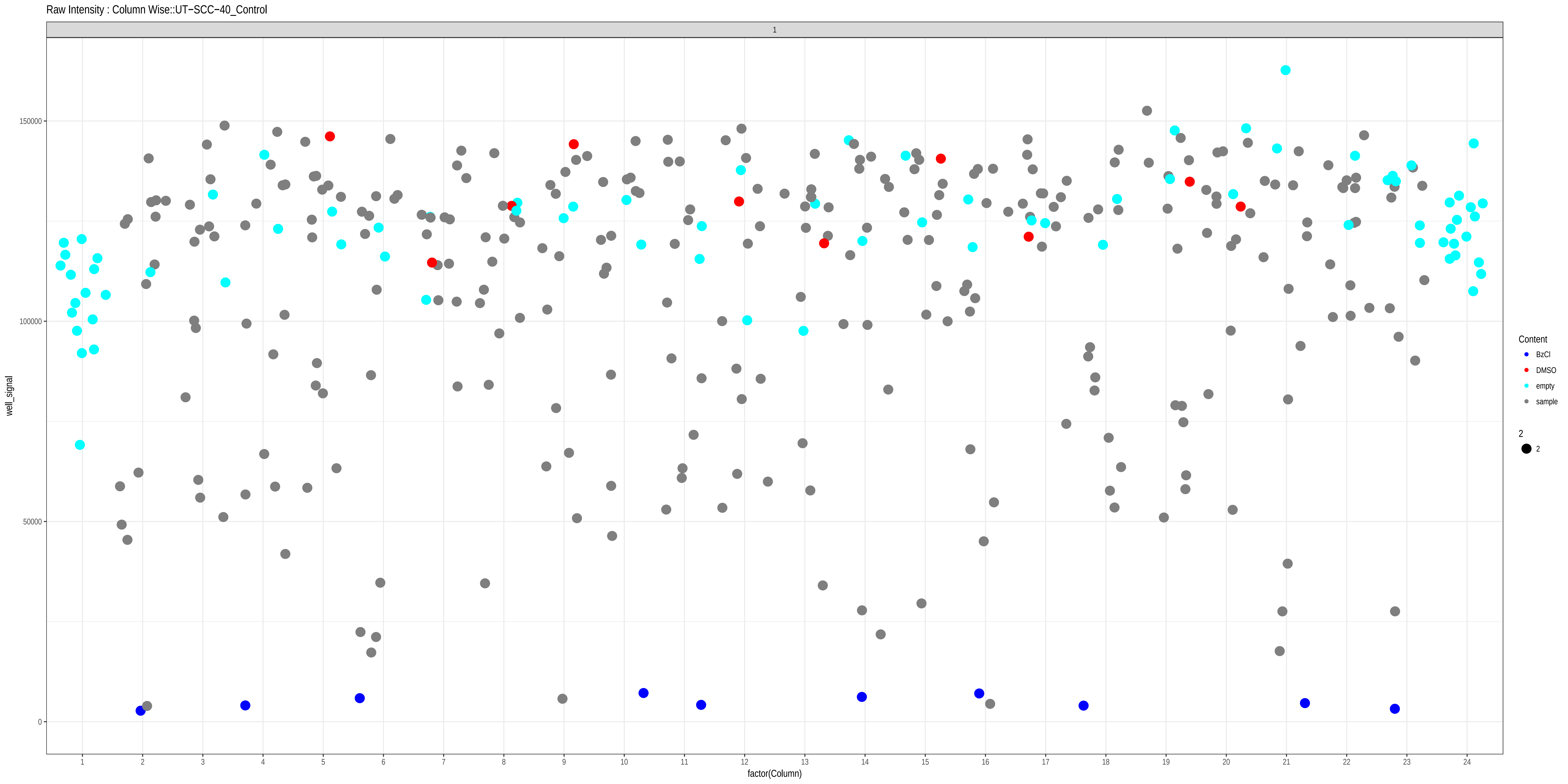
Raw Intensity : Column Wise::UT−SCC−40_Control
1
well_signal
150000
100000
50000
0
1
2
3
4
5
6
7
8
9
10
11
12
13
14
15
16
17
18
19
20
21
22
23
24
factor(Column)
Content
BzCl
DMSO
empty
sample
2
2

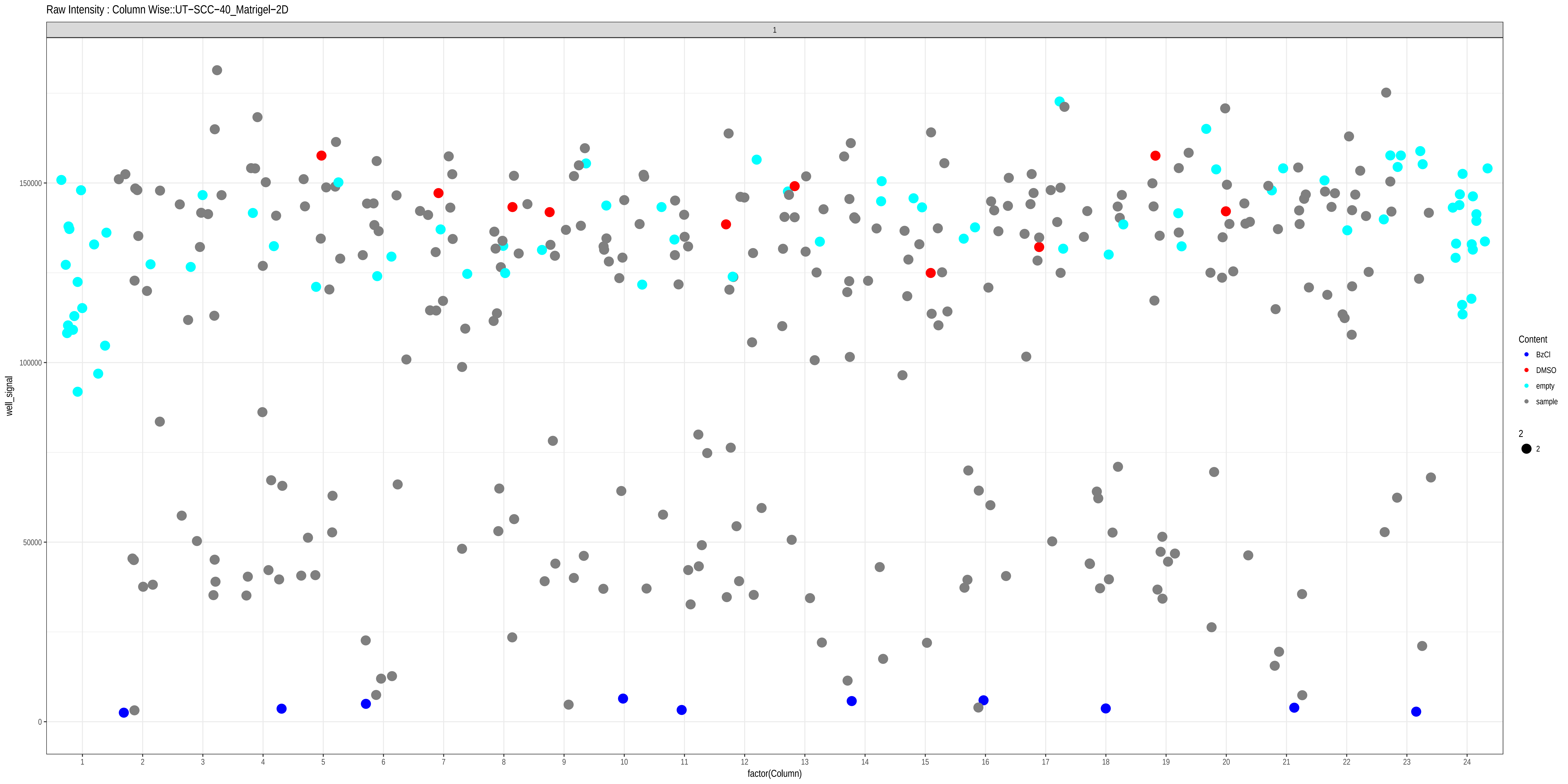
Raw Intensity : Column Wise::UT−SCC−40_Matrigel−2D
1
well_signal
150000
100000
50000
0
factor(Column)
1
2
3
4
5
6
7
8
9
10
11
12
13
14
15
16
17
18
19
20
21
22
23
24
Content
BzCl
DMSO
empty
sample
2
2

Raw Intensity : Column Wise::UT−SCC−40_Matrigel−3D

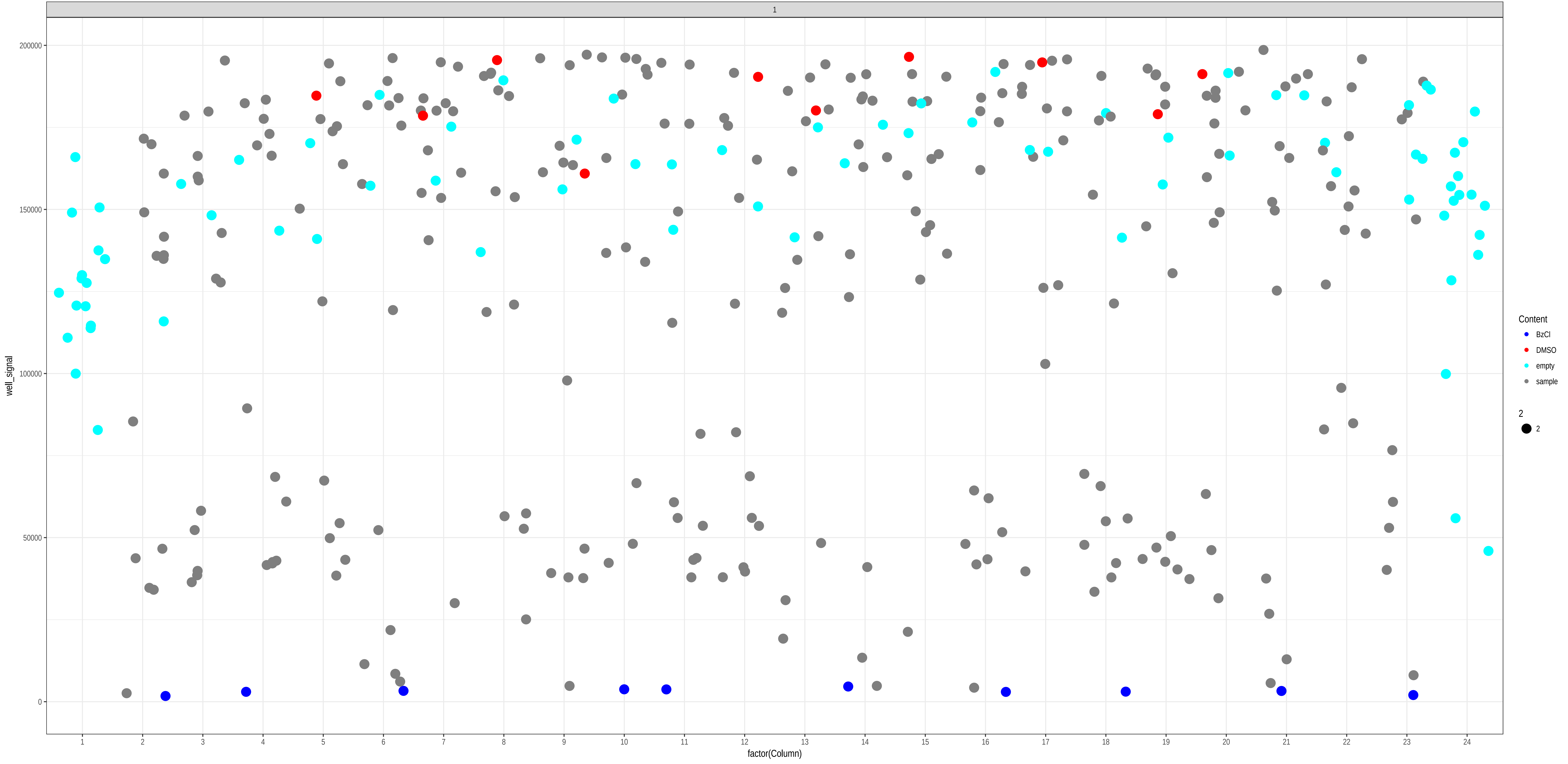

Raw Intensity : Column Wise::UT−SCC−40_Myogel−2D

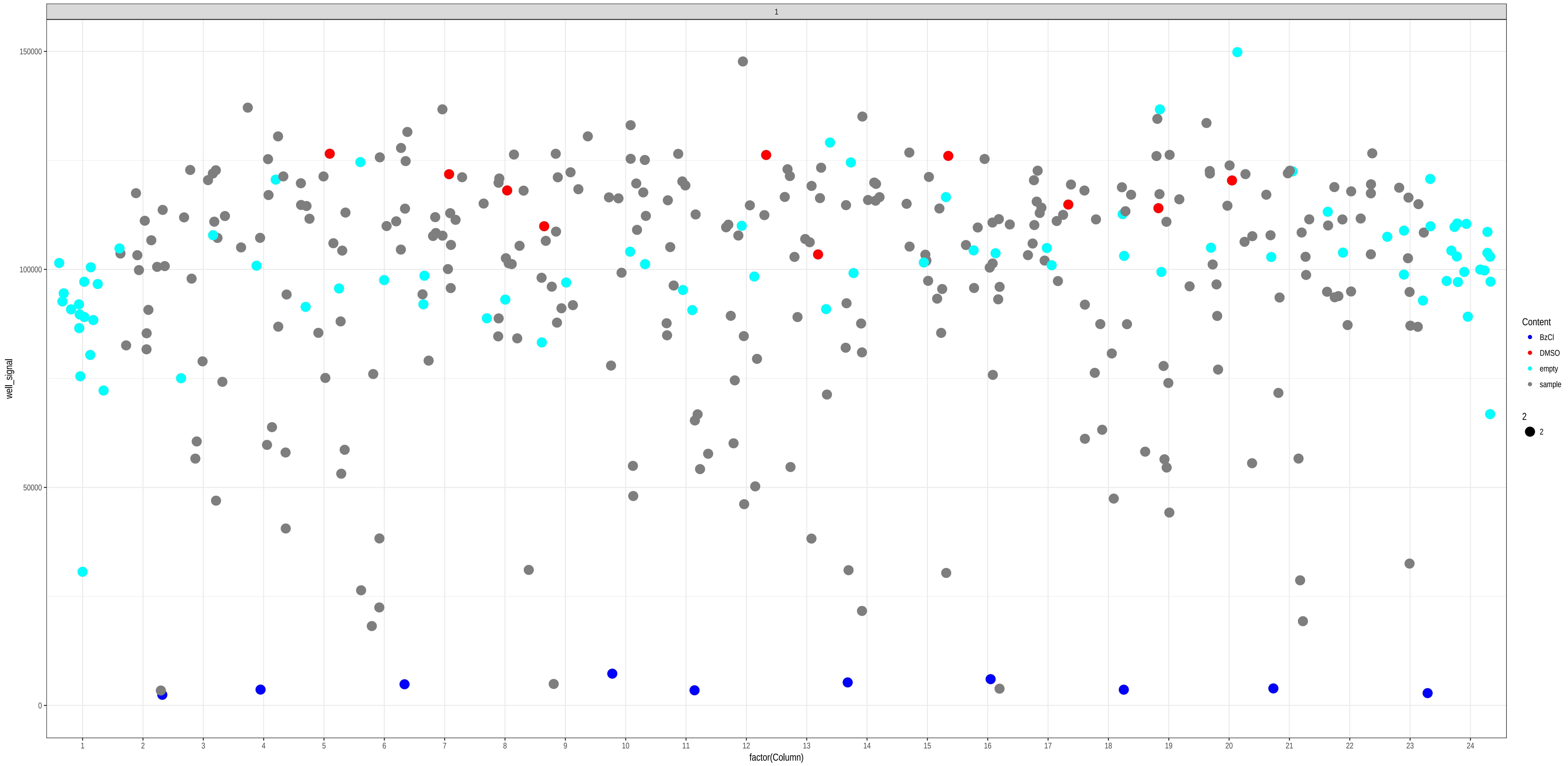

Raw Intensity : Column Wise::UT−SCC−40_Myogel−3D

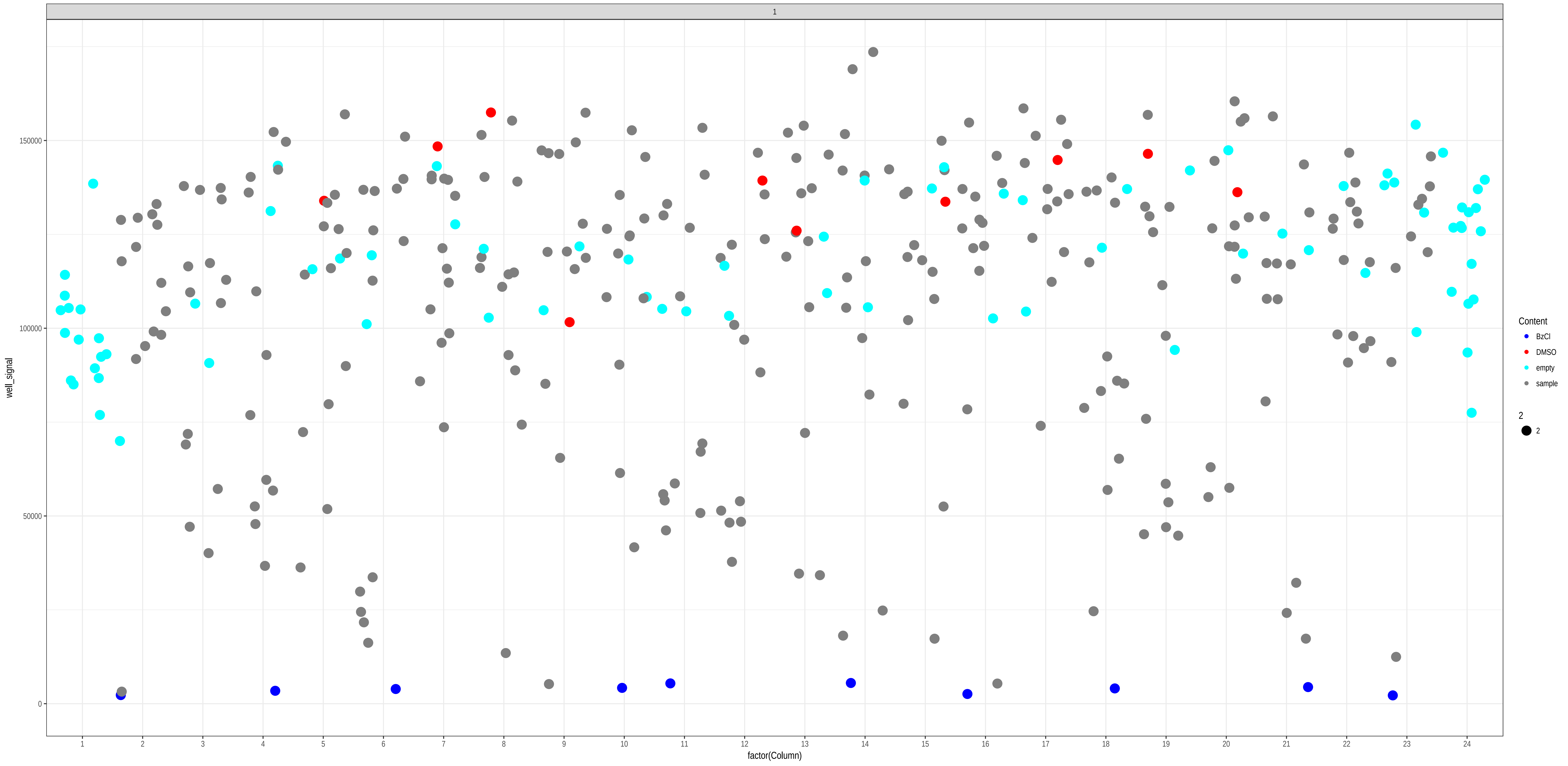

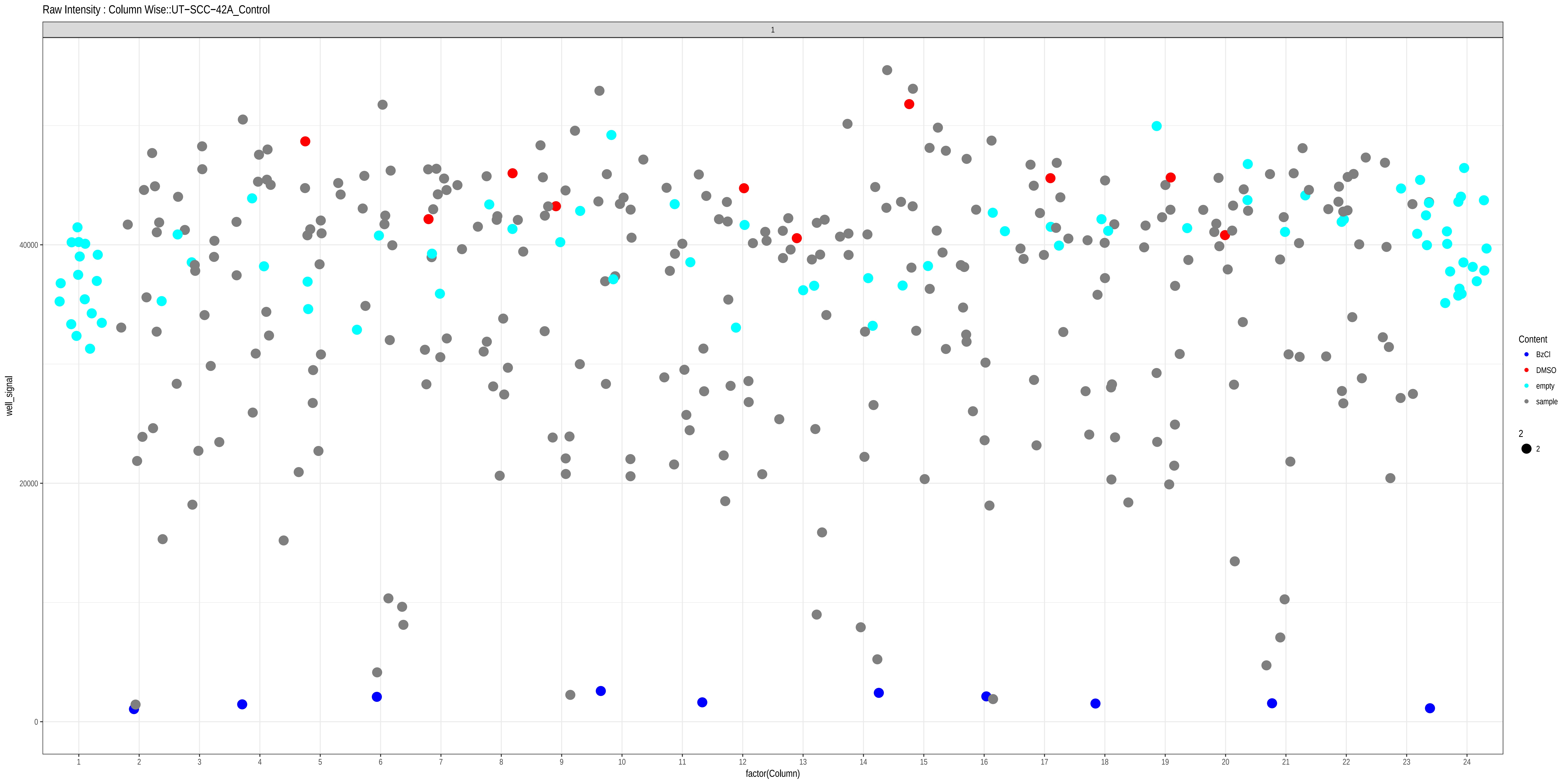

Raw Intensity : Column Wise::UT−SCC−42A_Control
1
well_signal
0
20000
40000
factor(Column)
1
2
3
4
5
6
7
8
9
10
11
12
13
14
15
16
17
18
19
20
21
22
23
24
Content
BzCl
DMSO
empty
sample
2
2

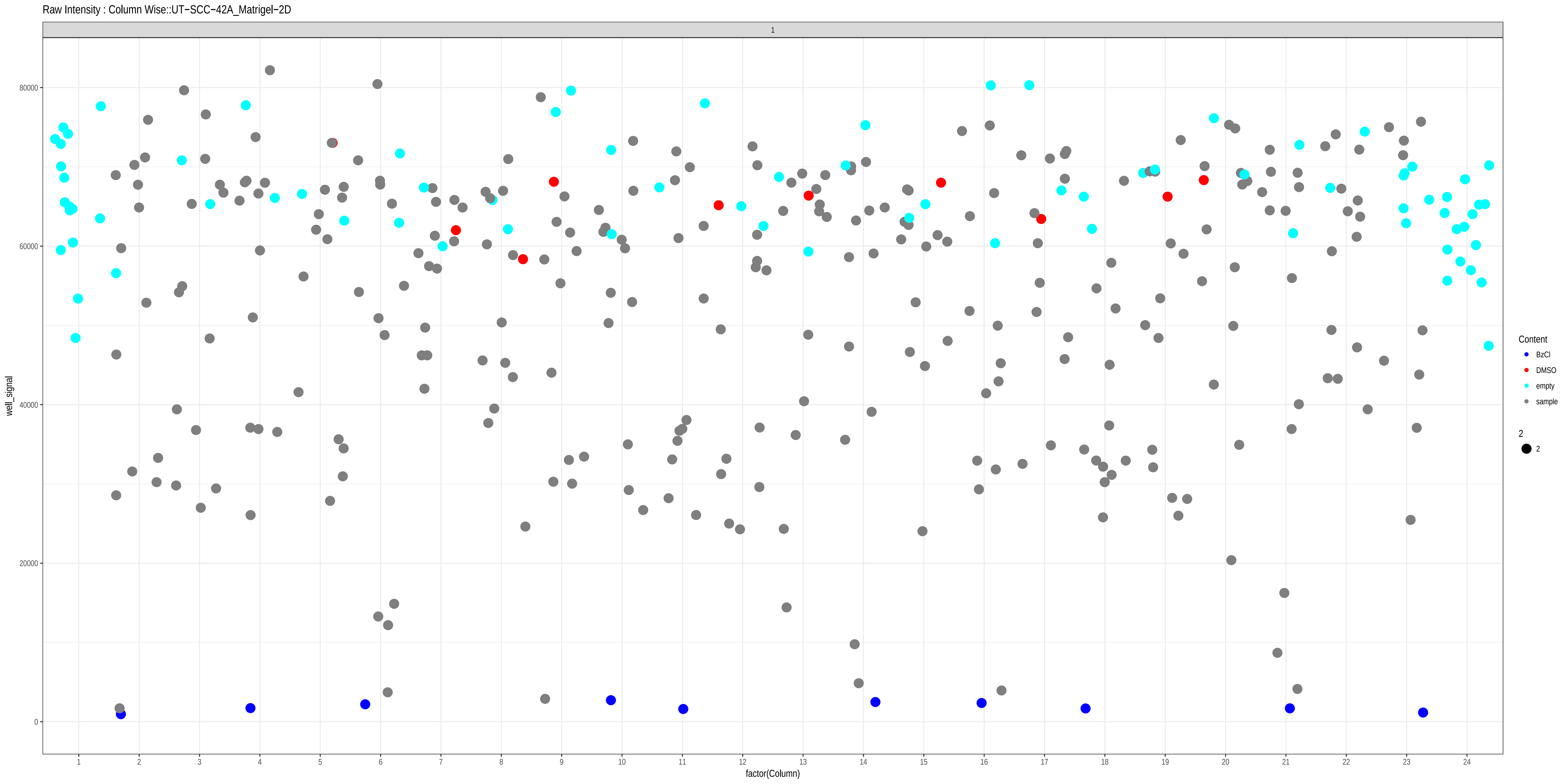

Raw Intensity : Column Wise::UT−SCC−42A_Matrigel−2D
1
well_signal
80000
60000
40000
20000
0
1
2
3
4
5
6
7
8
9
10
11
12
13
14
15
16
17
18
19
20
21
22
23
24
factor(Column)
Content
BzCl
DMSO
empty
sample
2
2

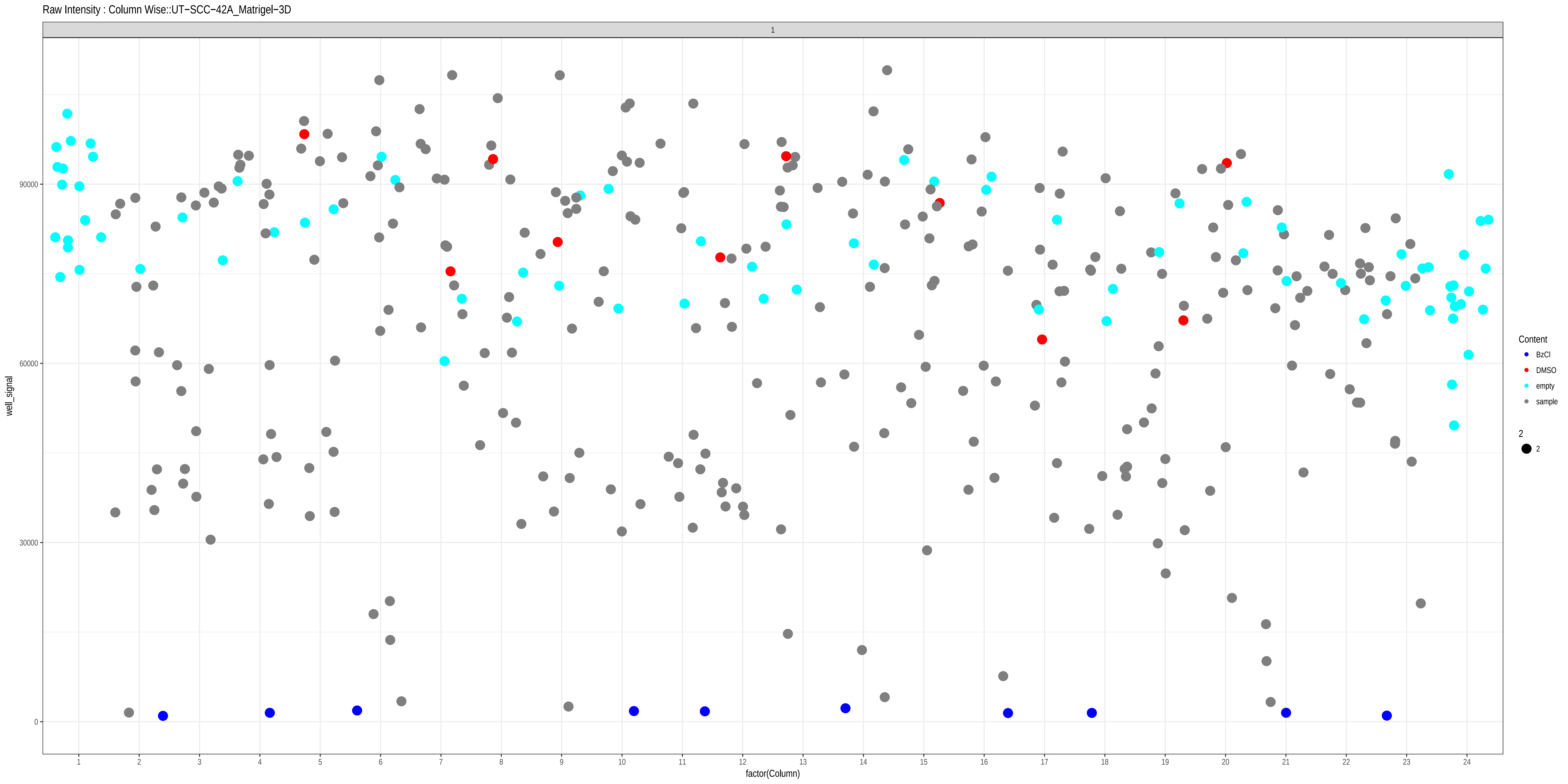
Raw Intensity : Column Wise::UT−SCC−42A_Matrigel−3D
1
well_signal
90000
60000
30000
0
1 2 3 4 5 6 7 8 9 10 11 12 13 14 15 16 17 18 19 20 21 22 23 24
factor(Column)
Content
BzCl
DMSO
empty
sample
2
2

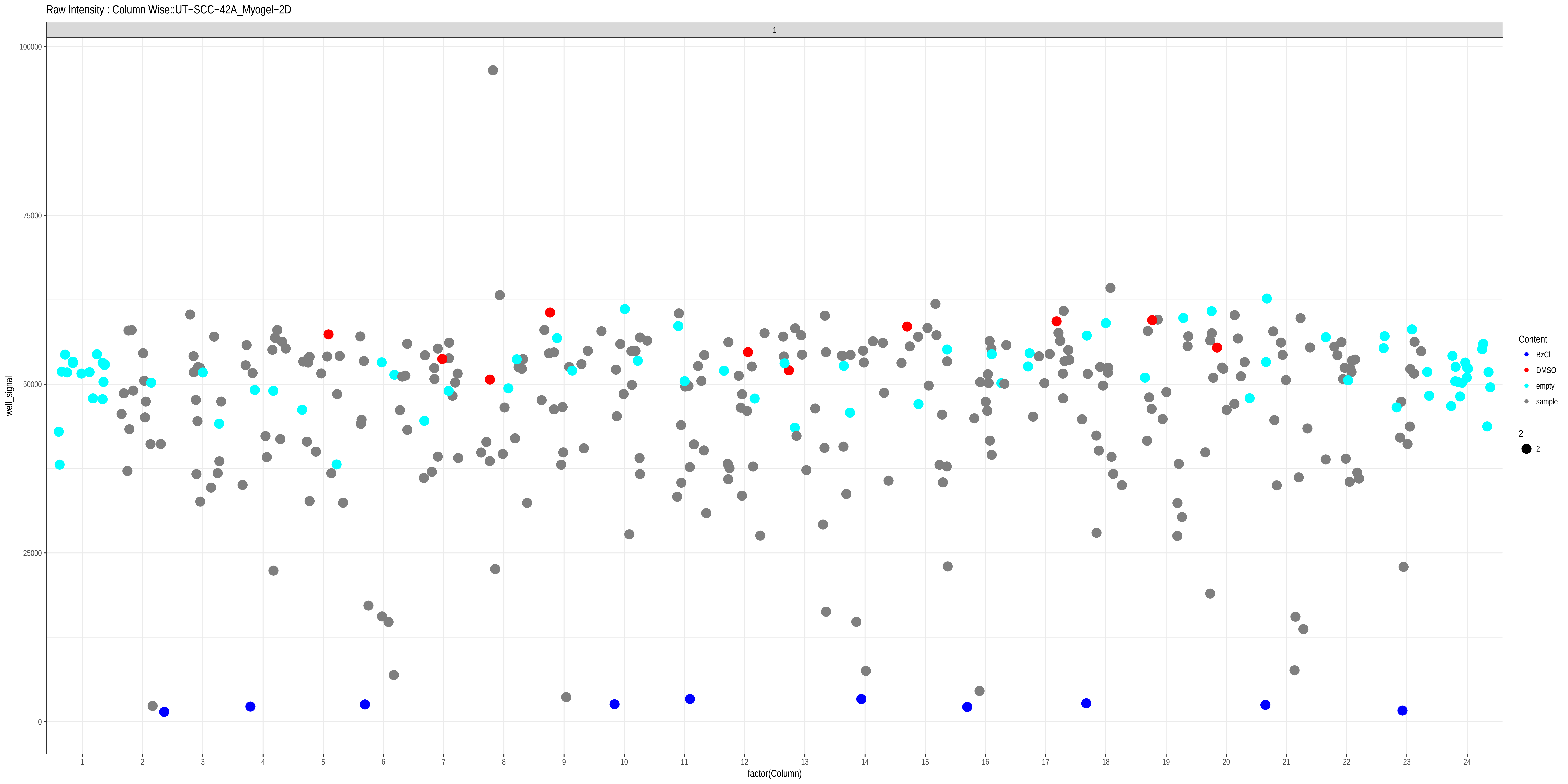
Raw Intensity : Column Wise::UT−SCC−42A_Myogel−2D
1
100000
75000
50000
25000
0
well_signal
1 2 3 4 5 6 7 8 9 10 11 12 13 14 15 16 17 18 19 20 21 22 23 24
factor(Column)
Content
BzCl
DMSO
empty
sample
2
2

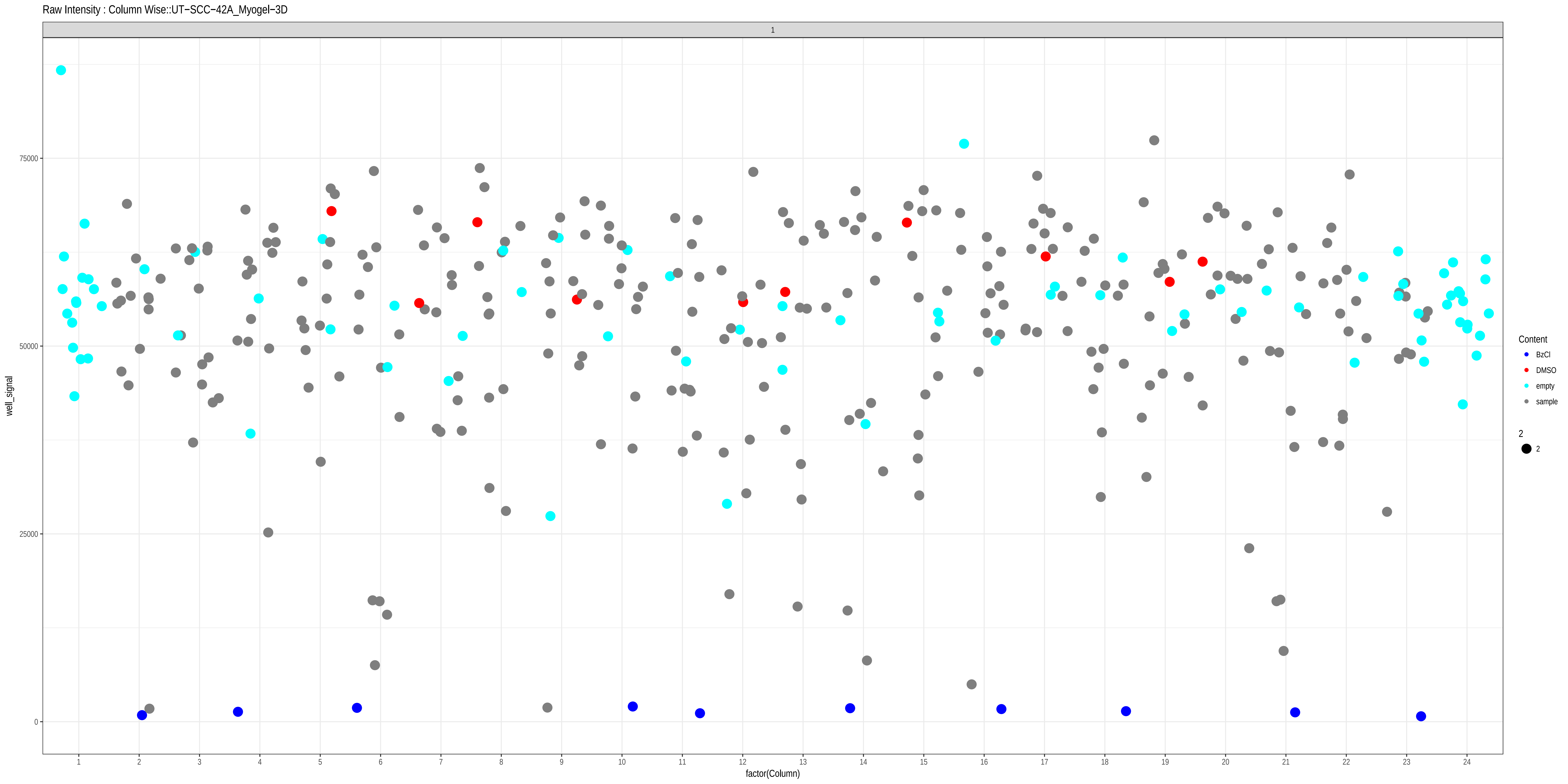

Raw Intensity : Column Wise::UT−SCC−42A_Myogel−3D
1
well_signal
75000
50000
25000
0
1 2 3 4 5 6 7 8 9 10 11 12 13 14 15 16 17 18 19 20 21 22 23 24
factor(Column)
Content
BzCl
DMSO
empty
sample
2
2

Raw Intensity : Column Wise::UT−SCC−42B_Control

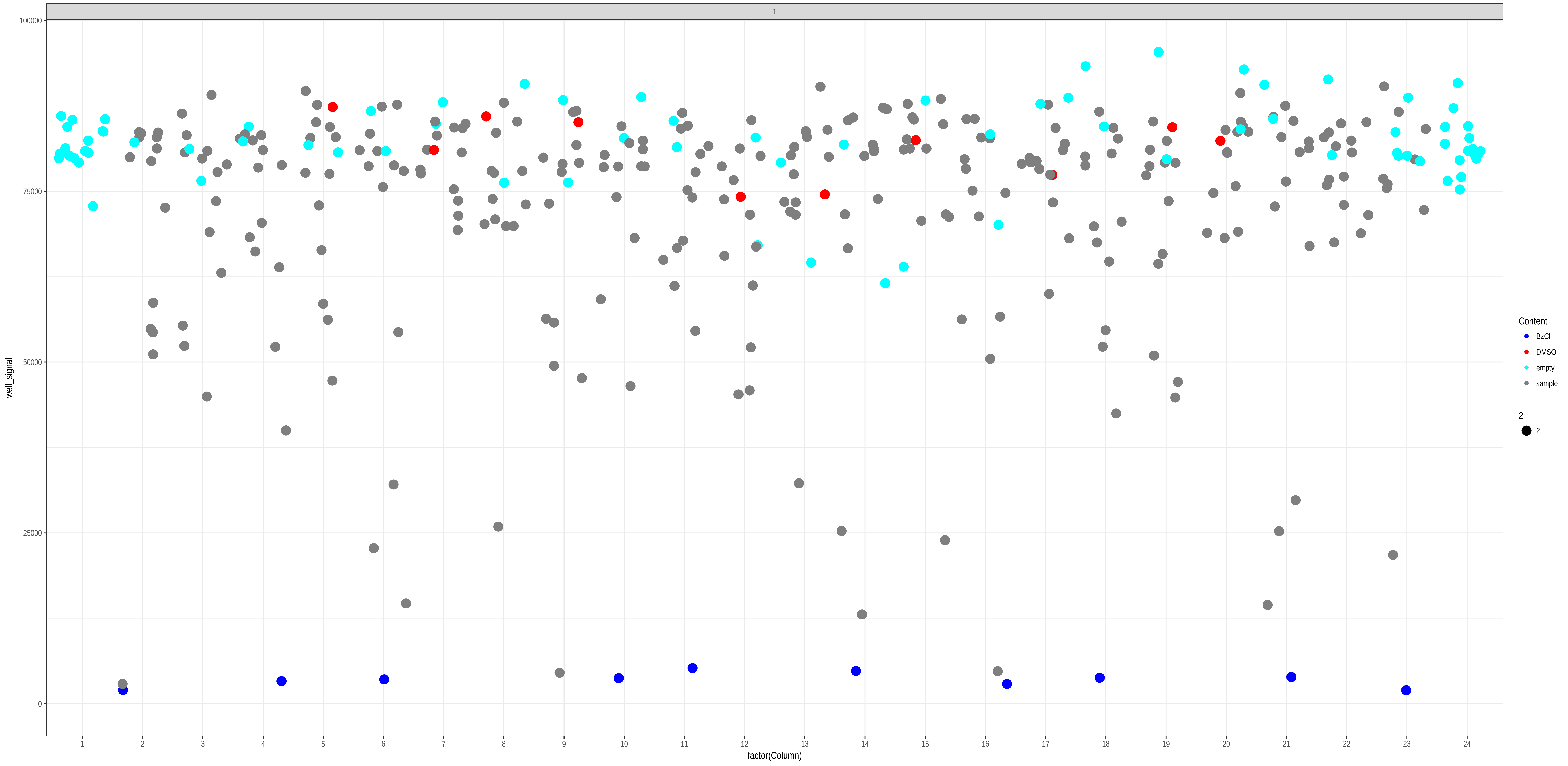

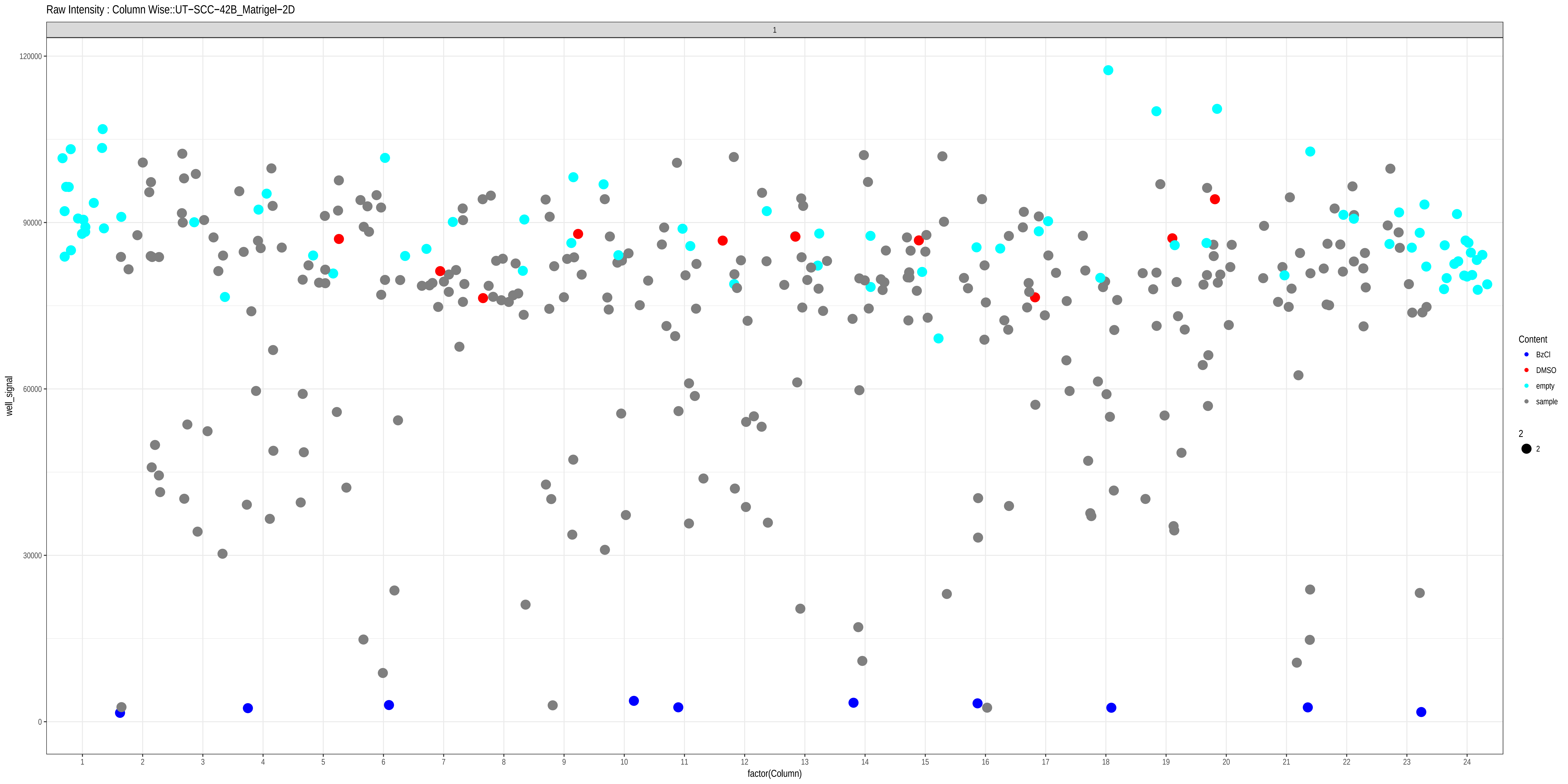

Raw Intensity : Column Wise::UT−SCC−42B_Matrigel−2D
1
120000
90000
60000
30000
0
well_signal
1
2
3
4
5
6
7
8
9
10
11
12
13
14
15
16
17
18
19
20
21
22
23
24
factor(Column)
Content
BzCl
DMSO
empty
sample
2
2

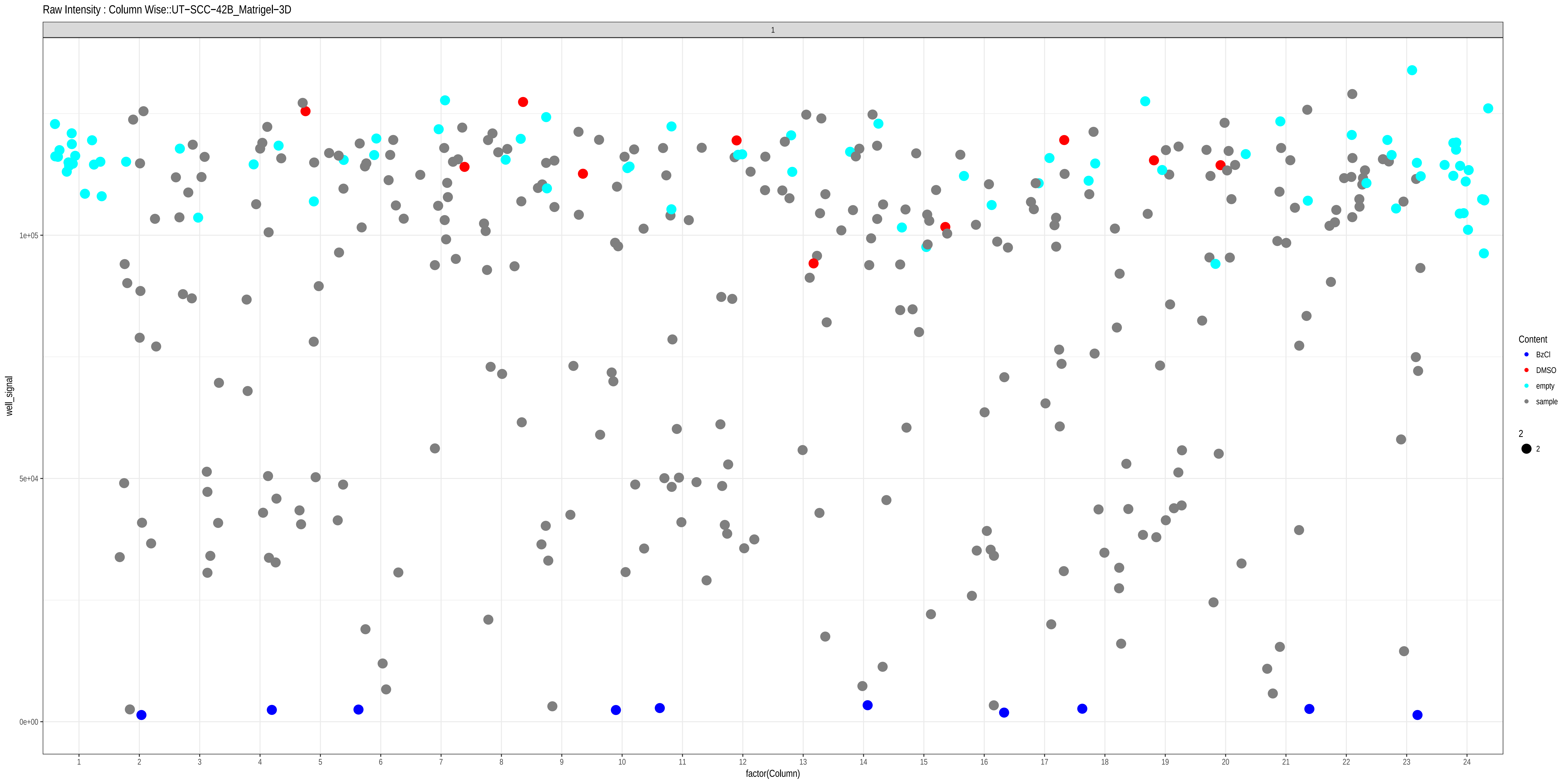

Raw Intensity : Column Wise::UT−SCC−42B_Matrigel−3D
1
well_signal
1e+05
5e+04
0e+00
1
2
3
4
5
6
7
8
9
10
11
12
13
14
15
16
17
18
19
20
21
22
23
24
factor(Column)
Content
BzCl
DMSO
empty
sample
2
2

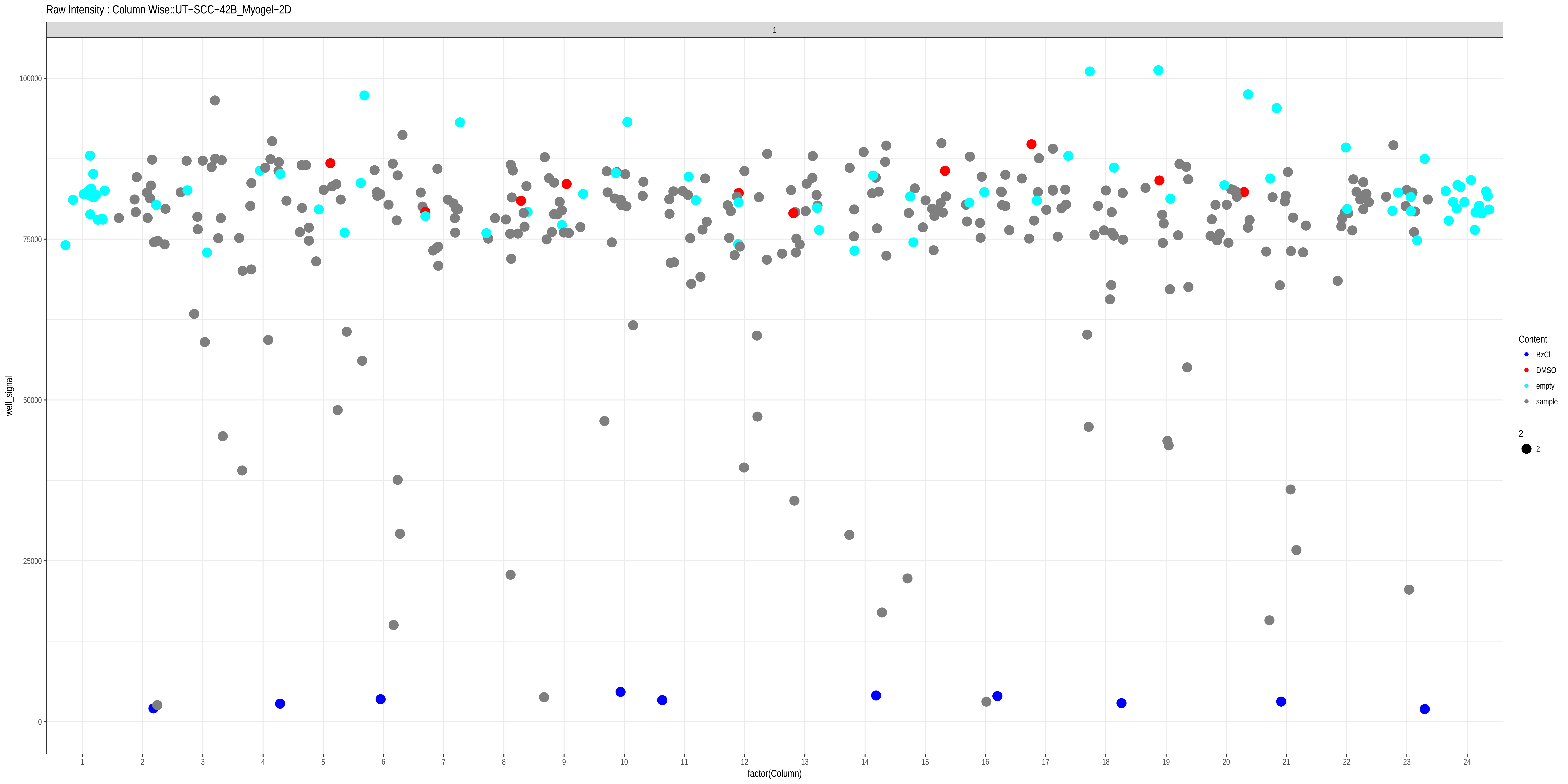
Raw Intensity : Column Wise::UT−SCC−42B_Myogel−2D
1
100000
75000
50000
25000
0
well_signal
1 2 3 4 5 6 7 8 9 10 11 12 13 14 15 16 17 18 19 20 21 22 23 24
factor(Column)
Content
BzCl
DMSO
empty
sample
2
2

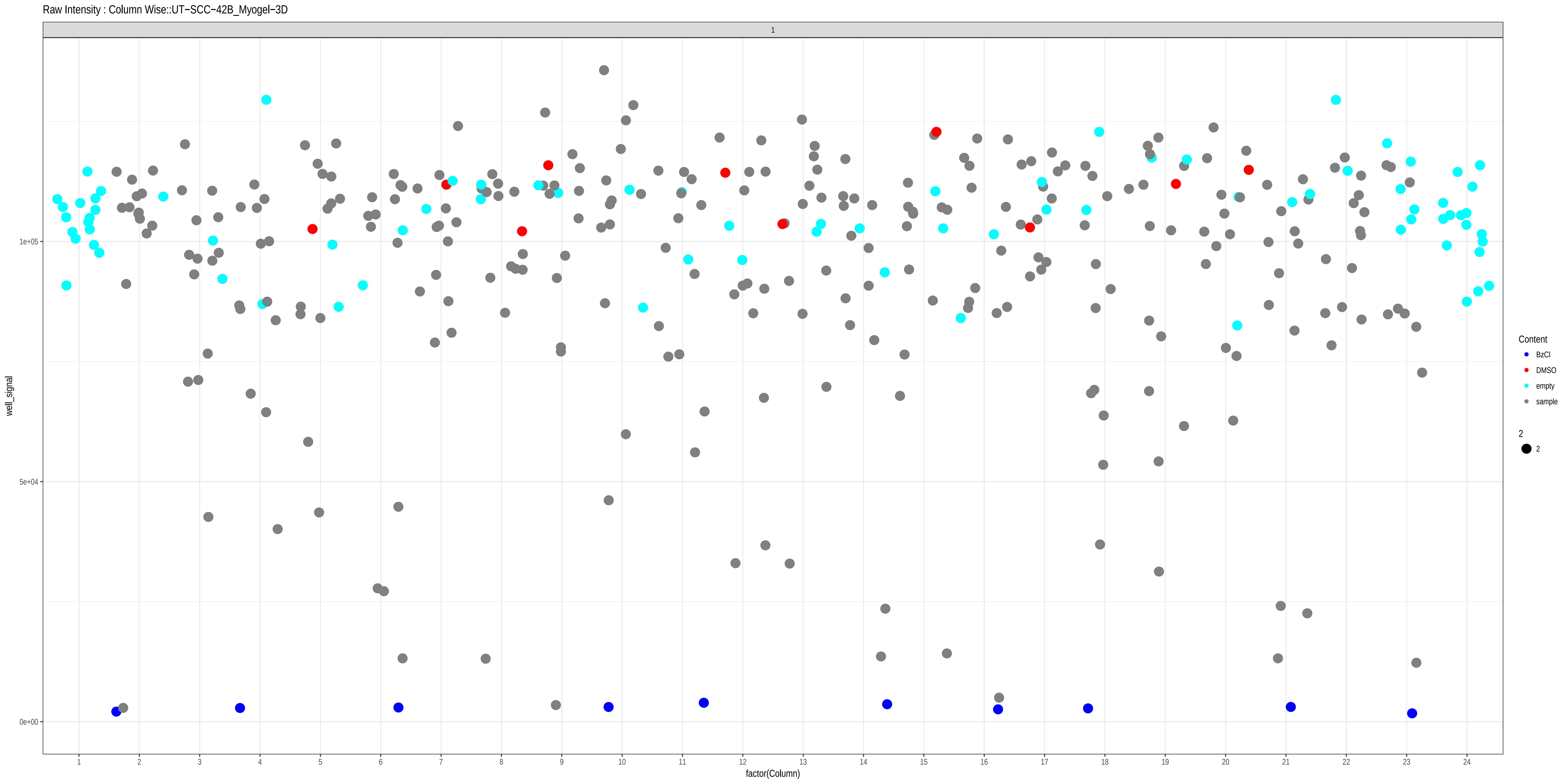

Raw Intensity : Column Wise::UT−SCC−42B_Myogel−3D
1
well_signal
1e+05
5e+04
0e+00
factor(Column)
1
2
3
4
5
6
7
8
9
10
11
12
13
14
15
16
17
18
19
20
21
22
23
24
Content
BzCl
DMSO
empty
sample
2
2

Raw Intensity : Column Wise::UT−SCC−44_Control

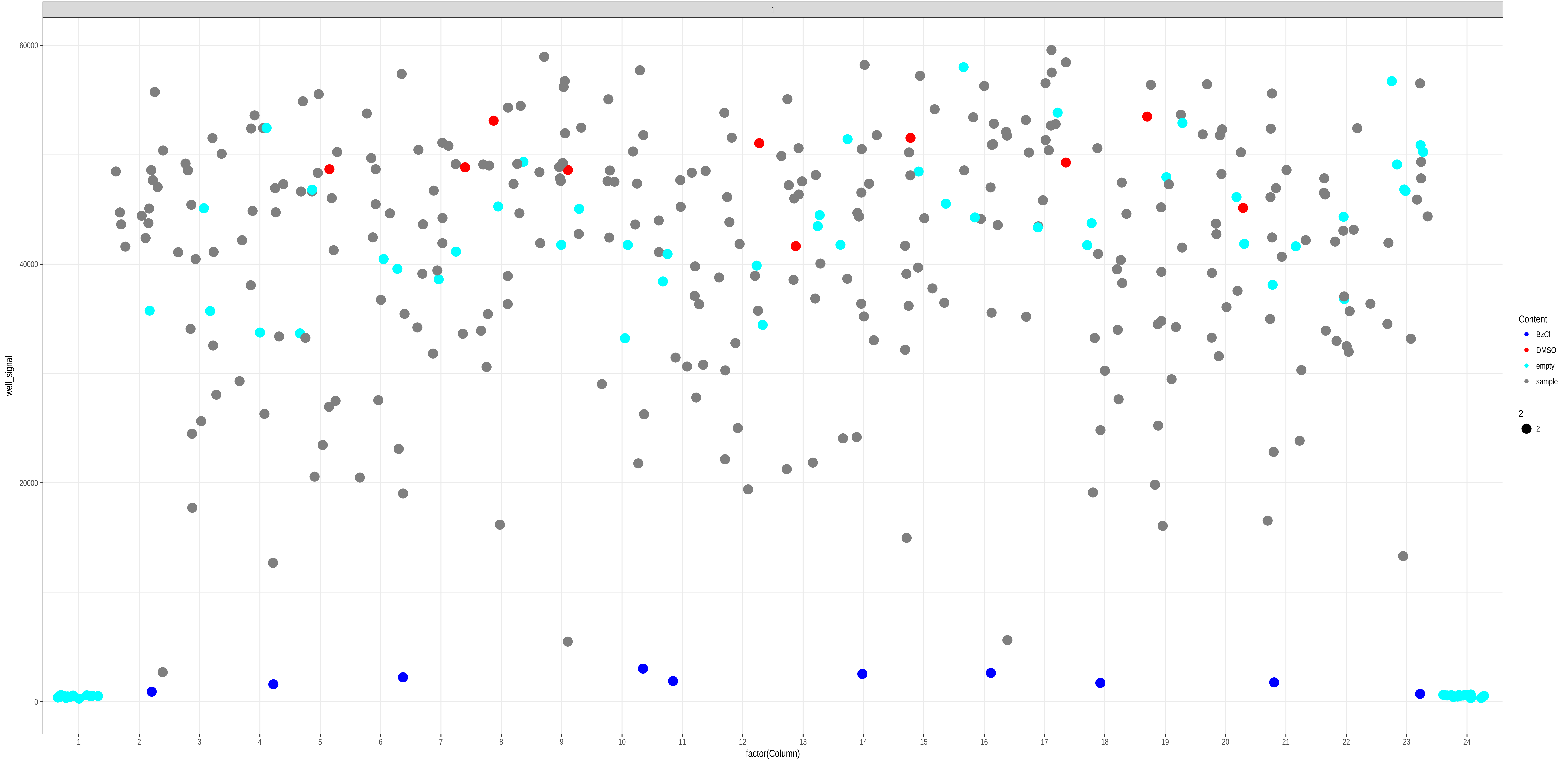

Raw Intensity : Column Wise::UT−SCC−44_Matrigel−2D

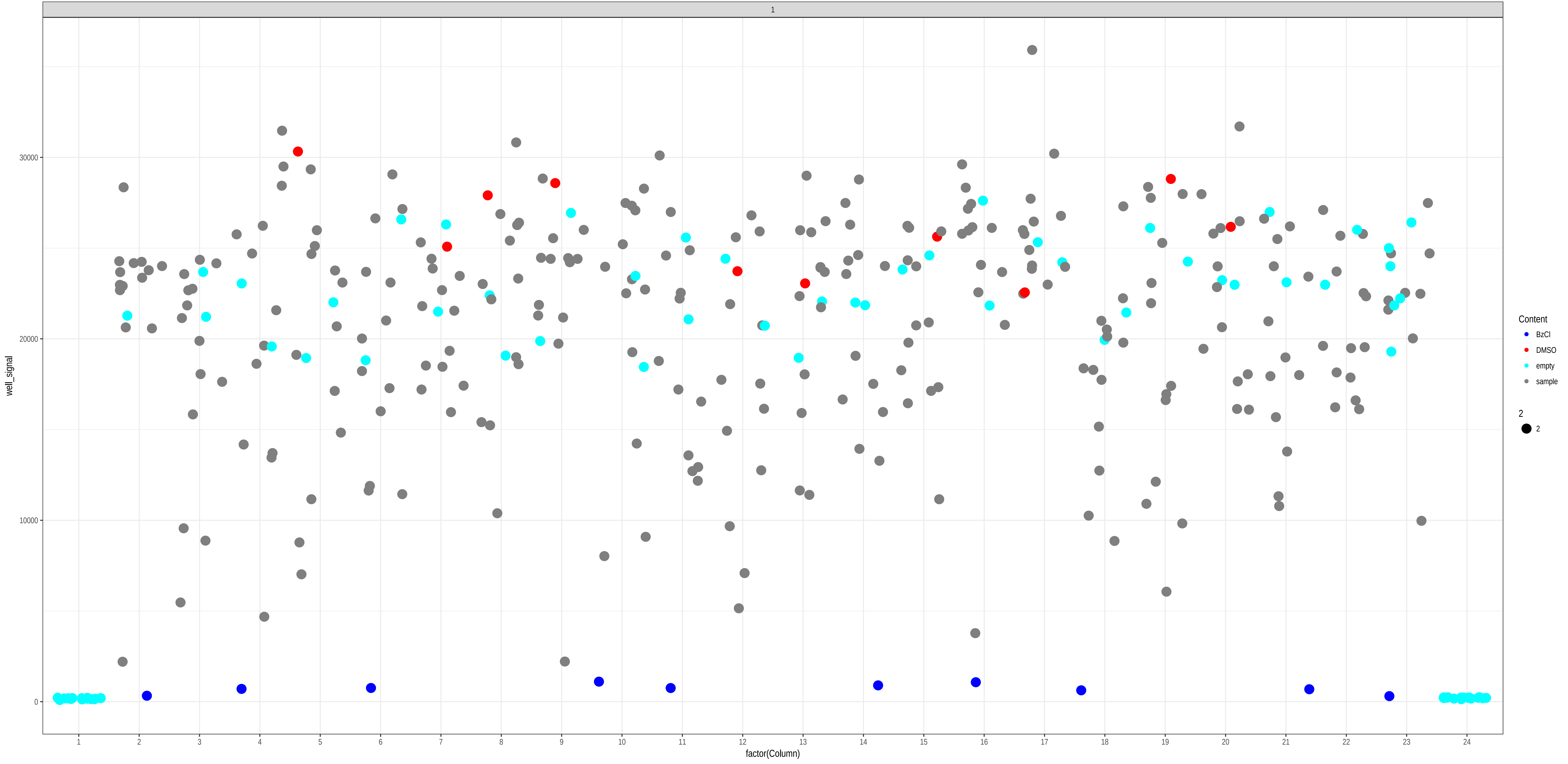

Raw Intensity : Column Wise::UT−SCC−44_Matrigel−3D

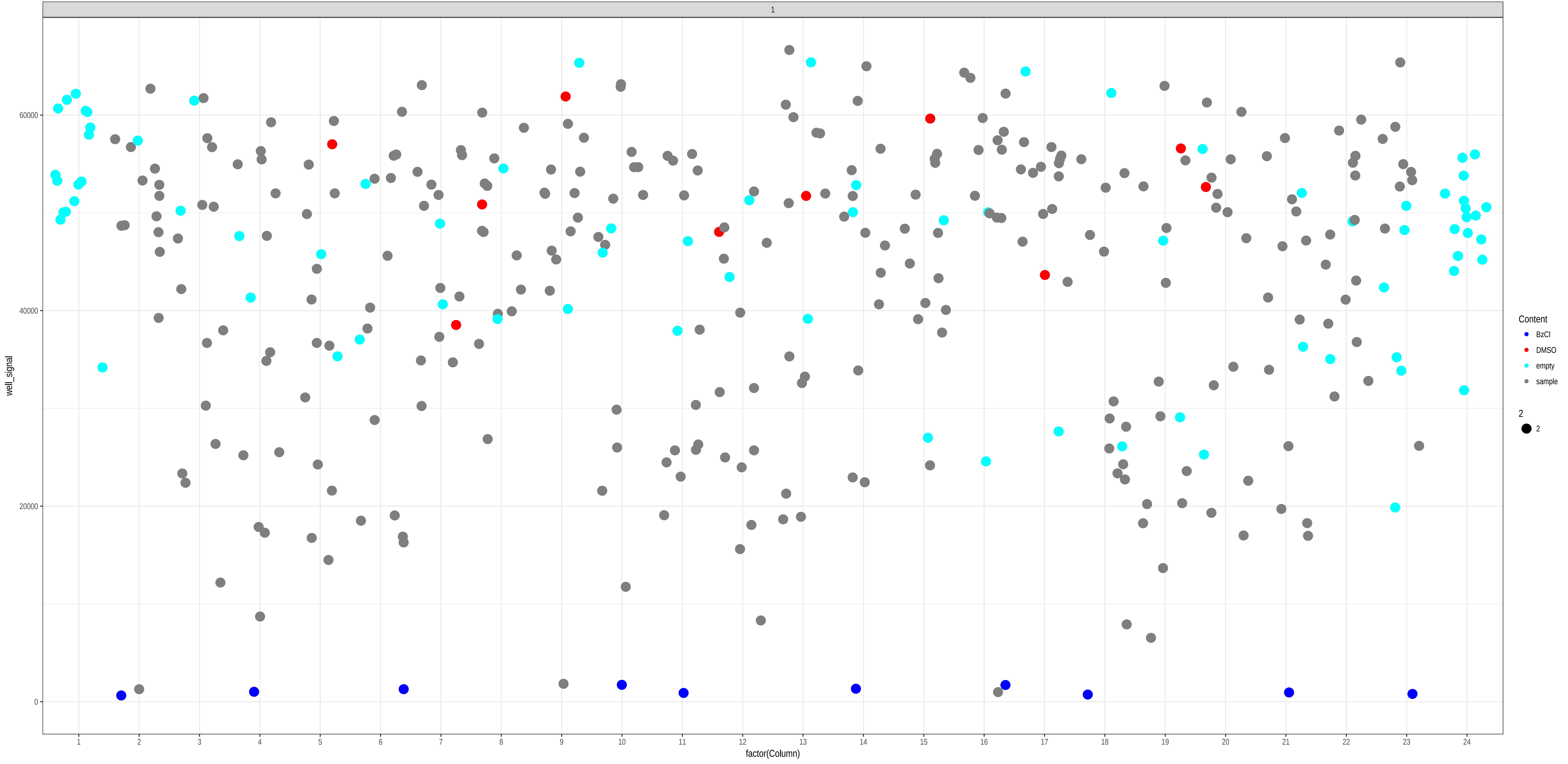

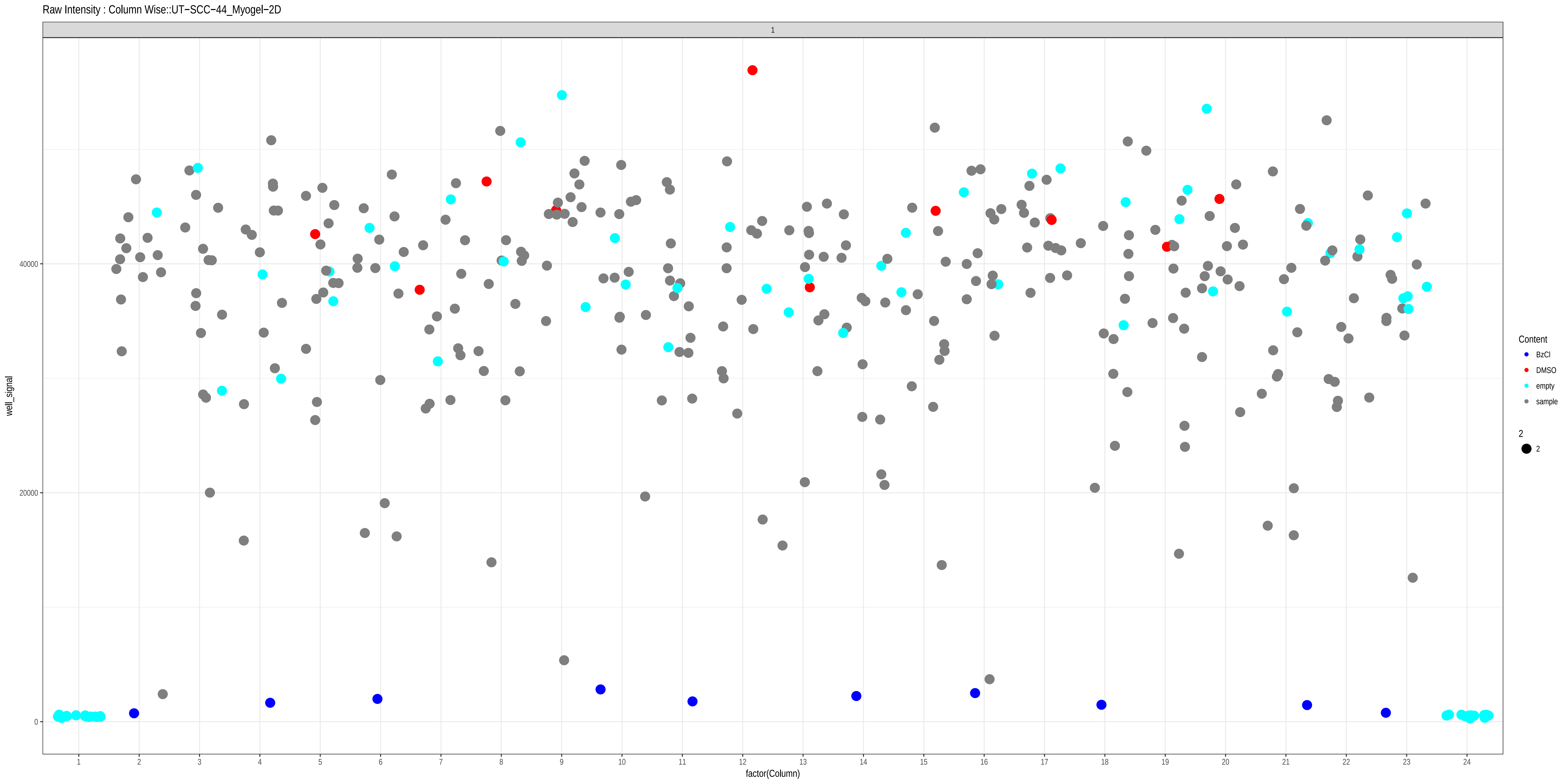
Raw Intensity : Column Wise::UT−SCC−44_Myogel−2D
1
well_signal
40000
20000
0
factor(Column)
1
2
3
4
5
6
7
8
9
10
11
12
13
14
15
16
17
18
19
20
21
22
23
24
Content
BzCl
DMSO
empty
sample
2
2

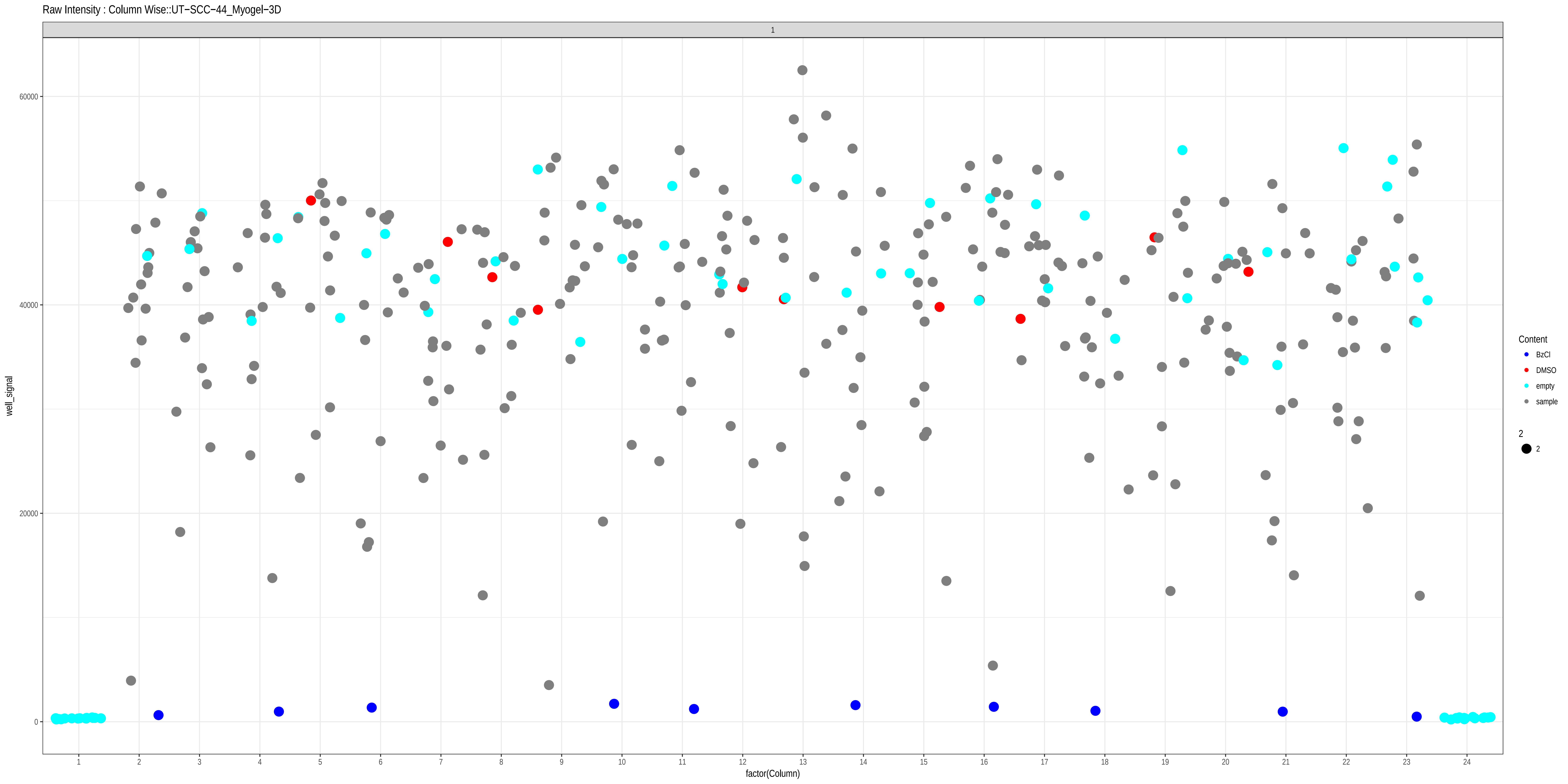

Raw Intensity : Column Wise::UT−SCC−44_Myogel−3D
1
60000
40000
20000
0
well_signal
1
2
3
4
5
6
7
8
9
10
11
12
13
14
15
16
17
18
19
20
21
22
23
24
factor(Column)
Content
BzCl
DMSO
empty
sample
2
2

Raw Intensity : Column Wise::UT-SCC-73_Control

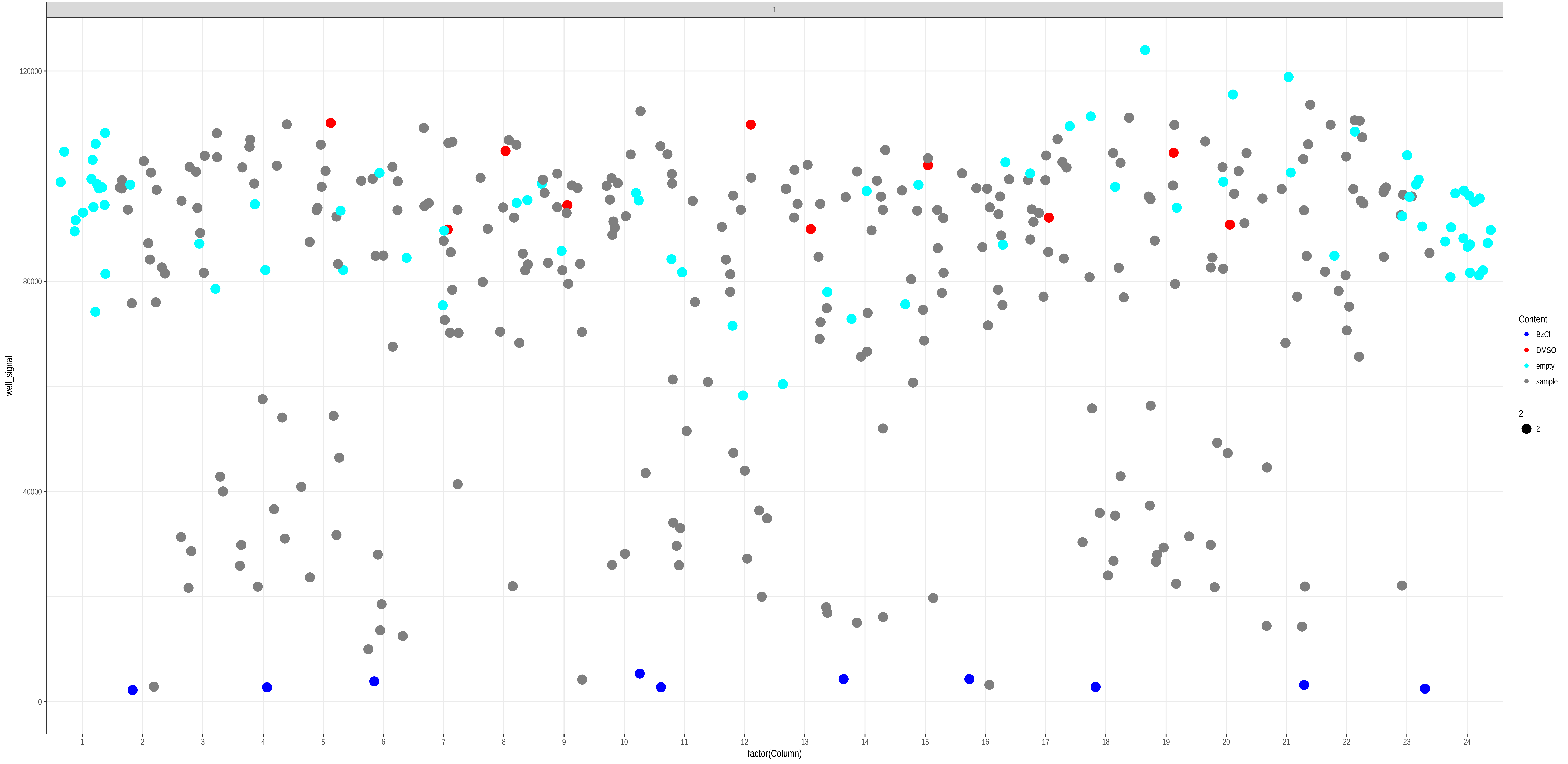

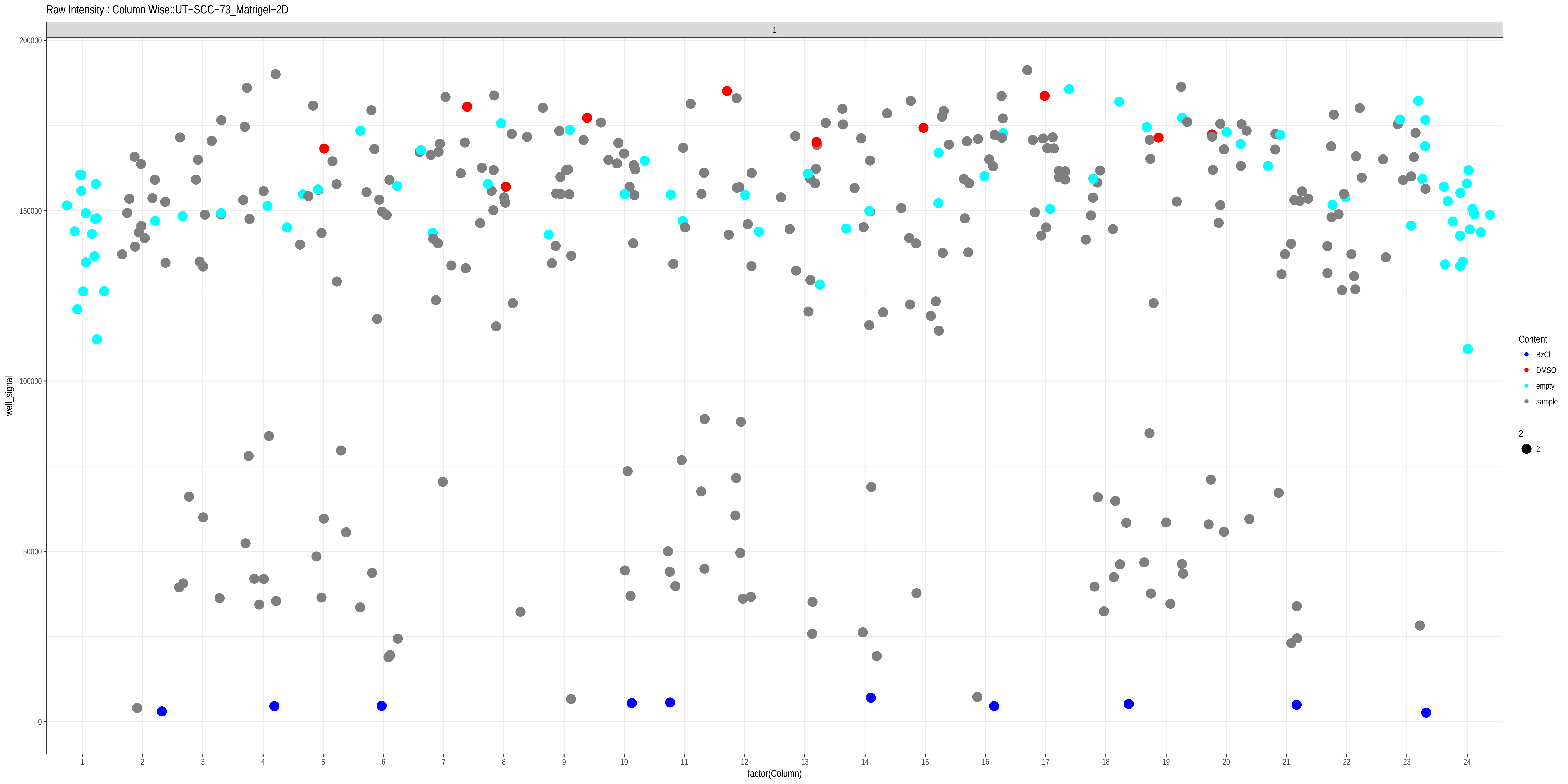

Raw Intensity : Column Wise::UT−SCC−73_Matrigel−2D
1
200000
150000
100000
50000
0
well_signal
1
2
3
4
5
6
7
8
9
10
11
12
13
14
15
16
17
18
19
20
21
22
23
24
factor(Column)
Content
BzCl
DMSO
empty
sample
2
2

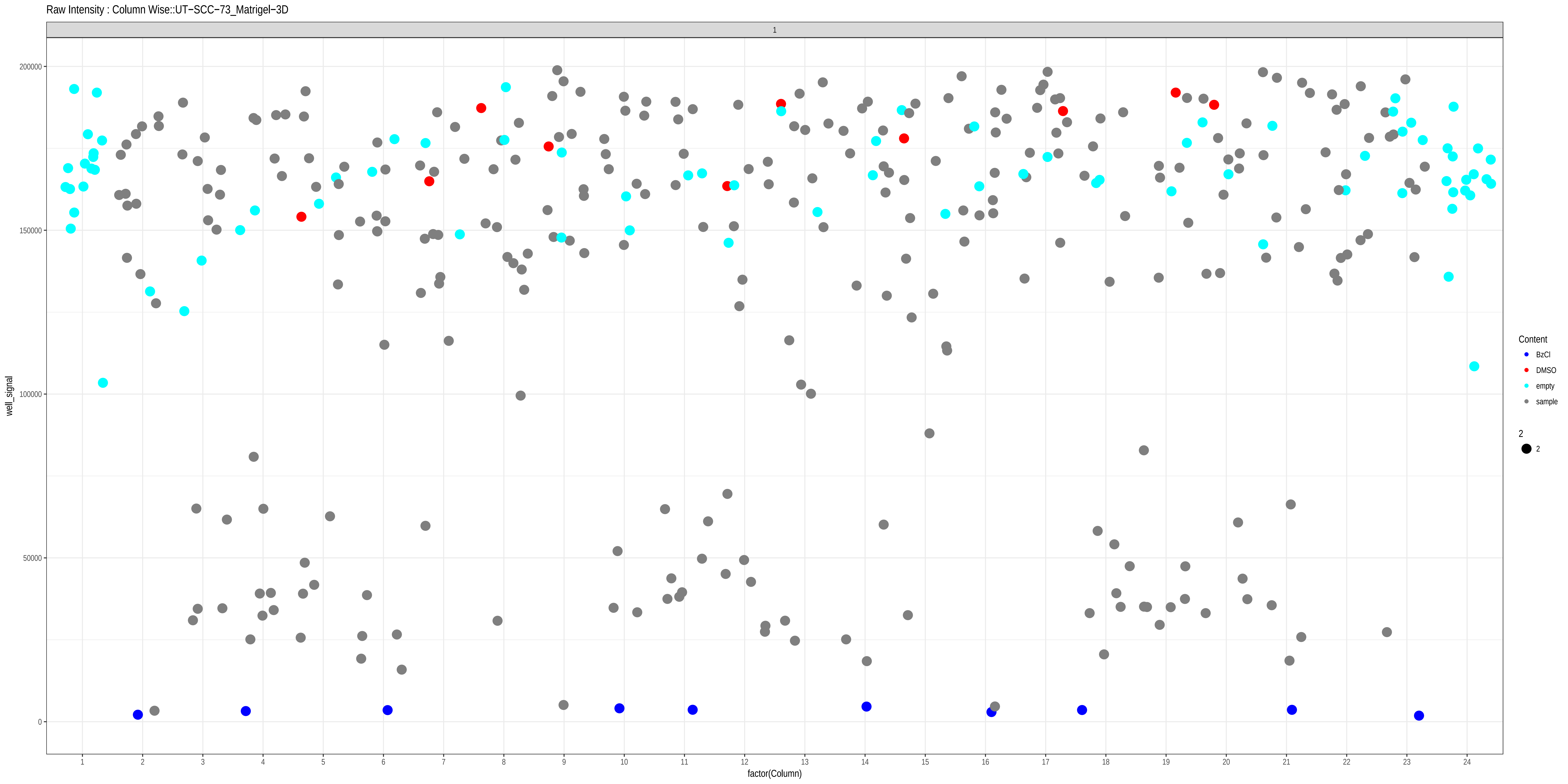

Raw Intensity : Column Wise::UT−SCC−73_Matrigel−3D
1
well_signal
200000
150000
100000
50000
0
factor(Column)
1
2
3
4
5
6
7
8
9
10
11
12
13
14
15
16
17
18
19
20
21
22
23
24
Content
BzCl
DMSO
empty
sample
2
2

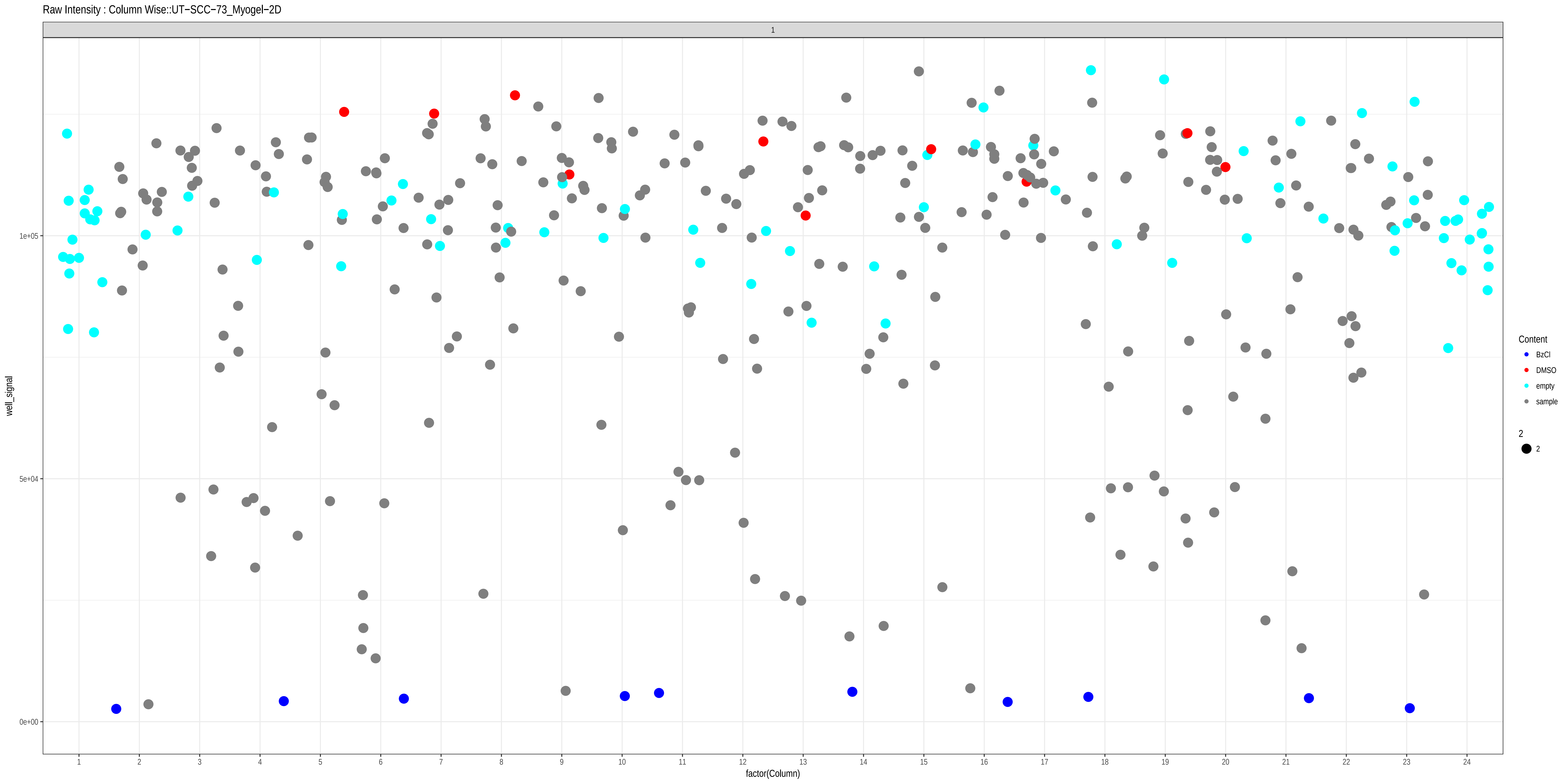
Raw Intensity : Column Wise::UT−SCC−73_Myogel−2D
1
well_signal
1e+05
5e+04
0e+00
factor(Column)
1
2
3
4
5
6
7
8
9
10
11
12
13
14
15
16
17
18
19
20
21
22
23
24
Content
BzCl
DMSO
empty
sample
2
2

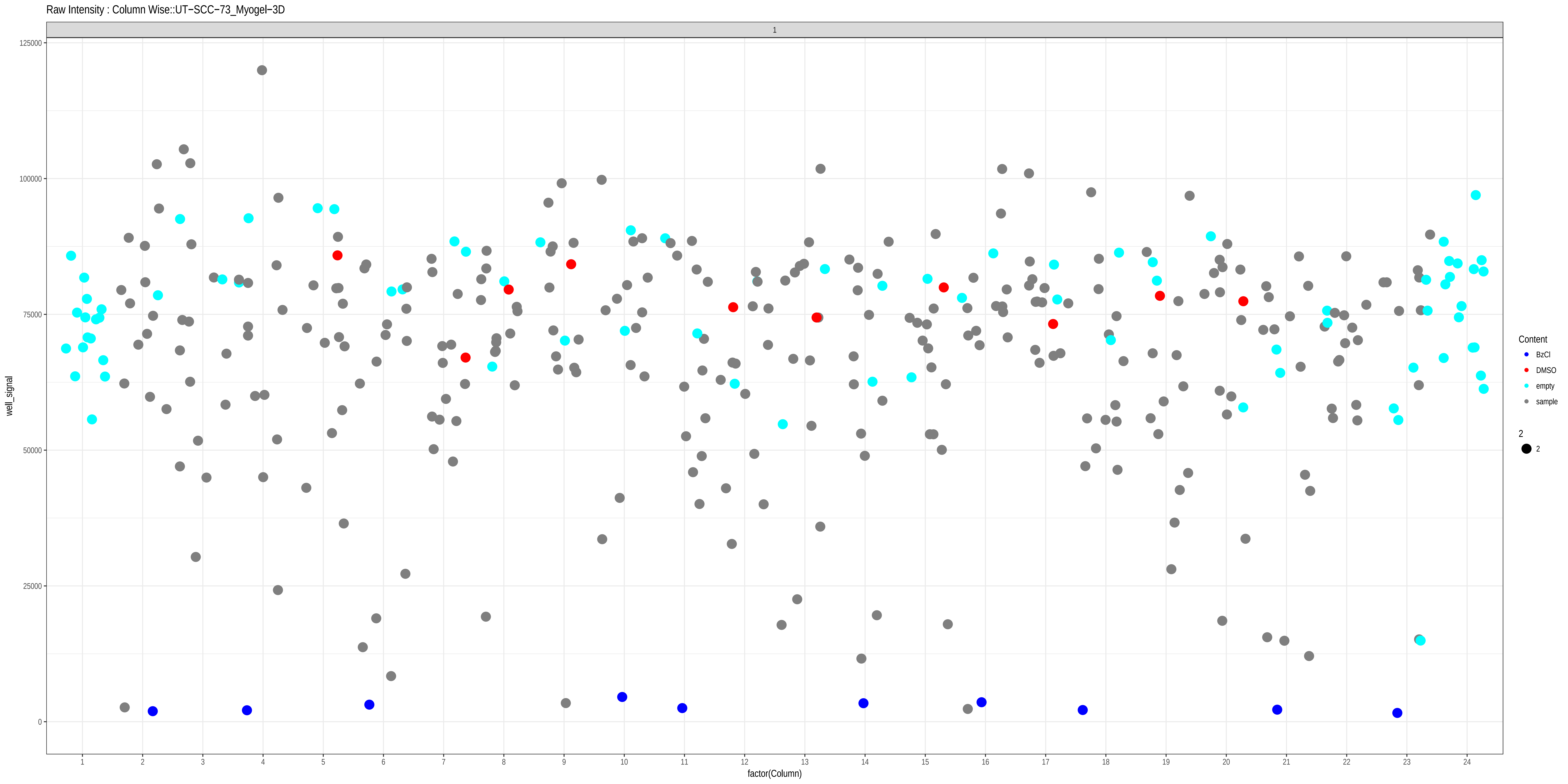

Raw Intensity : Column Wise::UT−SCC−73_Myogel−3D
1
well_signal
125000
100000
75000
50000
25000
0
factor(Column)
1
2
3
4
5
6
7
8
9
10
11
12
13
14
15
16
17
18
19
20
21
22
23
24
Content
BzCl
DMSO
empty
sample
2
2

Raw Intensity : Column Wise::UT−SCC−8_Control

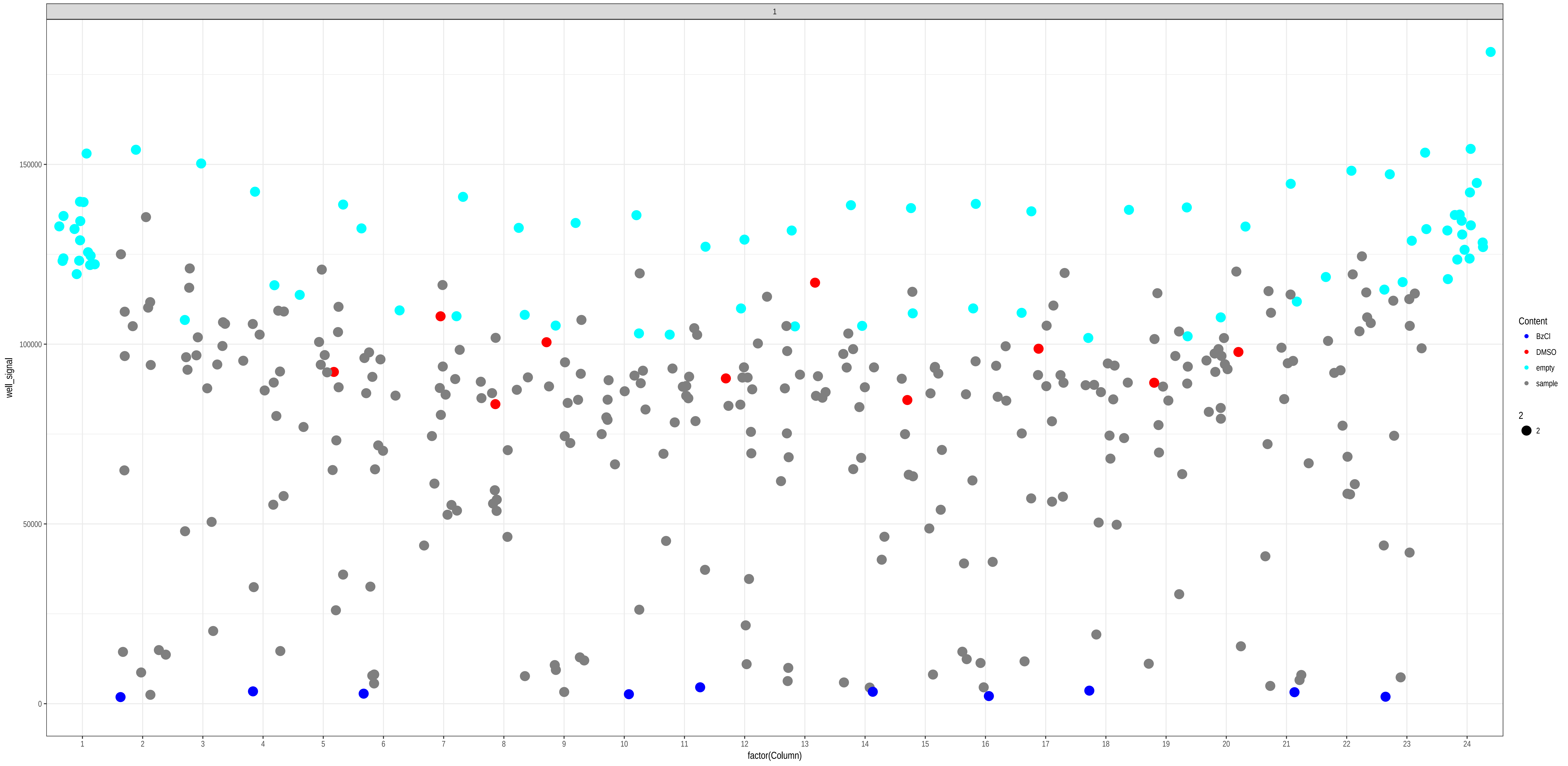

Raw Intensity : Column Wise::UT−SCC−8_Matrigel−2D

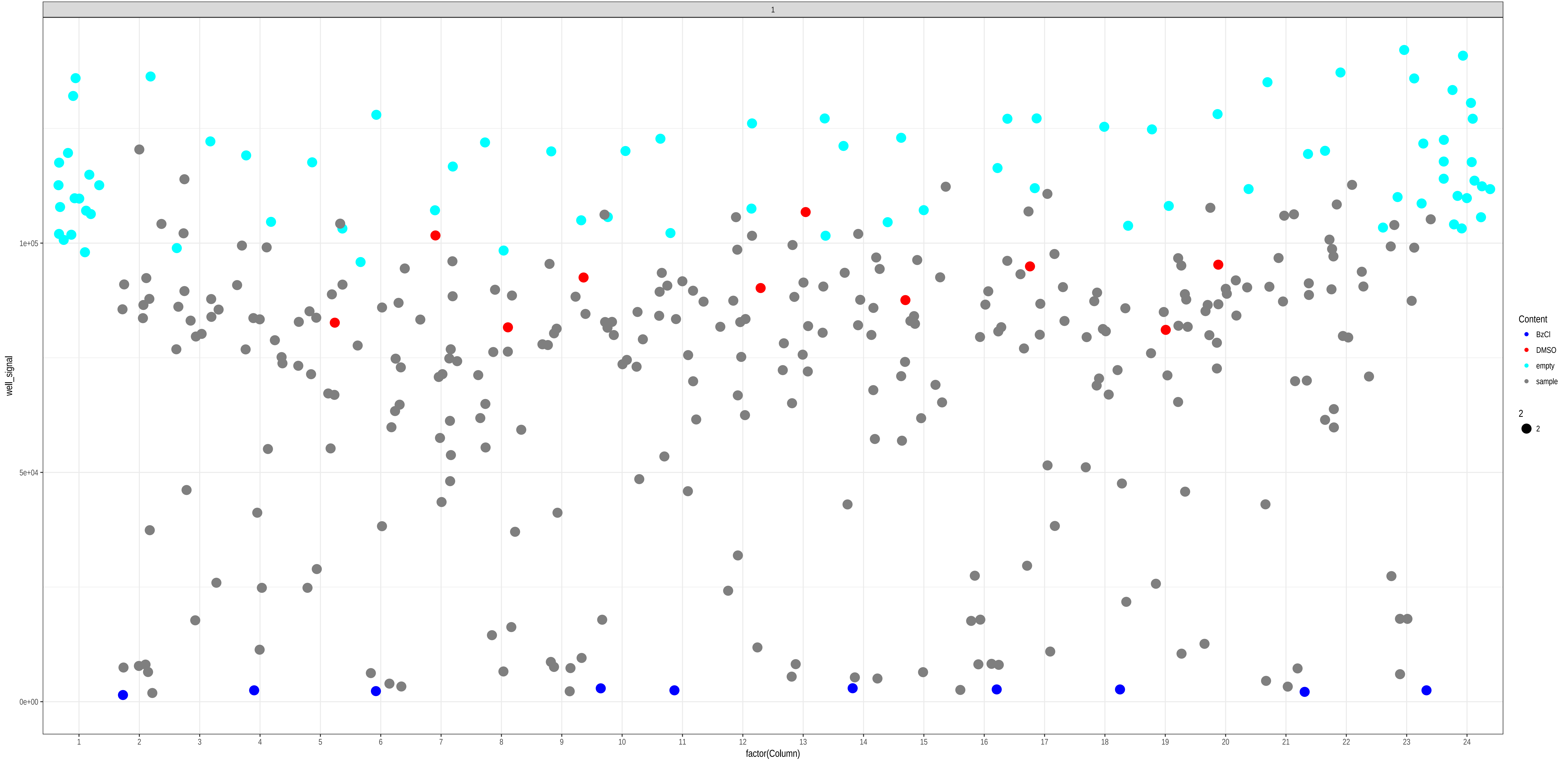

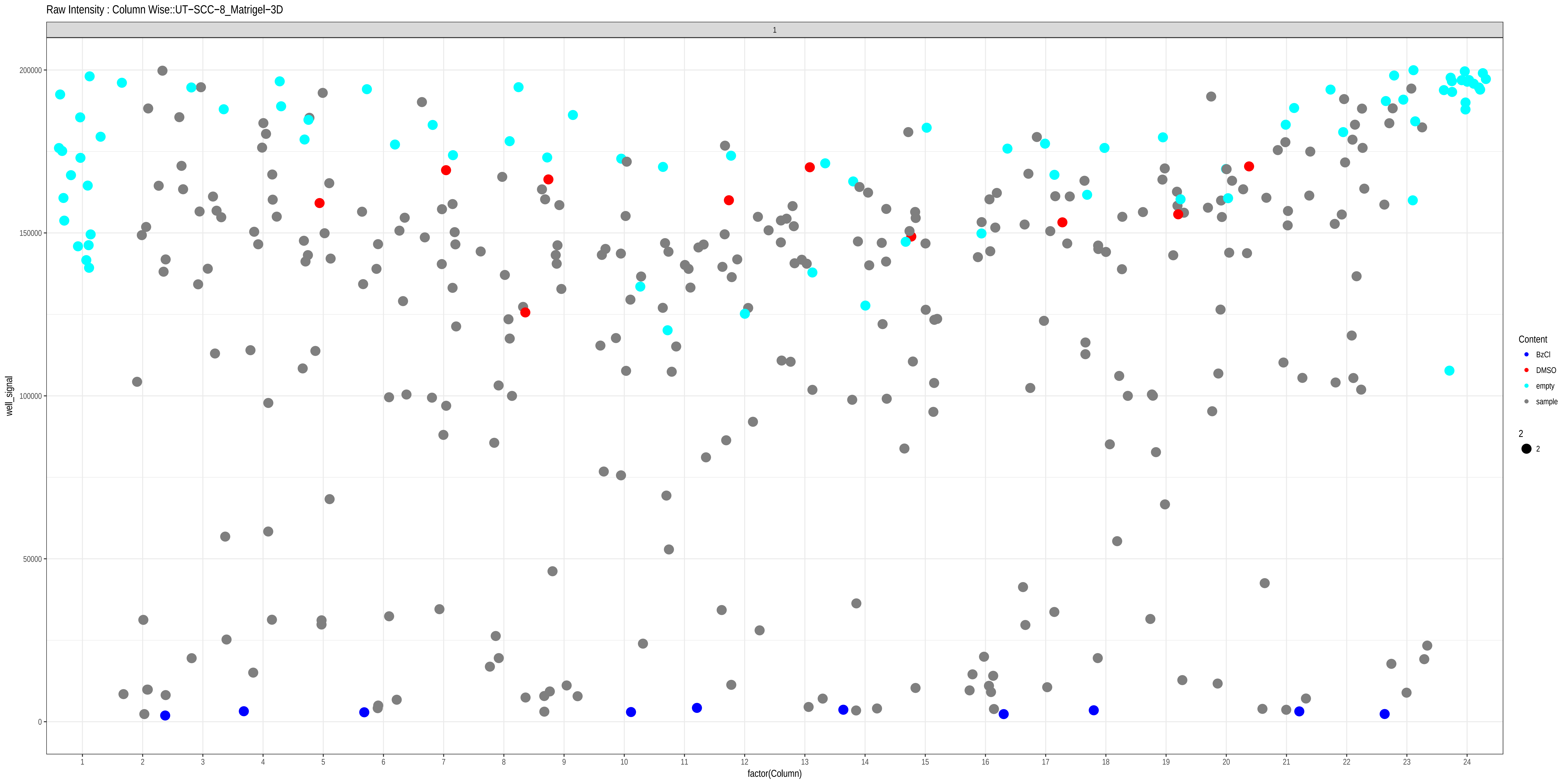
Raw Intensity : Column Wise::UT−SCC−8_Matrigel−3D
1
well_signal
200000
150000
100000
50000
0
1
2
3
4
5
6
7
8
9
10
11
12
13
14
15
16
17
18
19
20
21
22
23
24
factor(Column)
Content
BzCl
DMSO
empty
sample
2
2

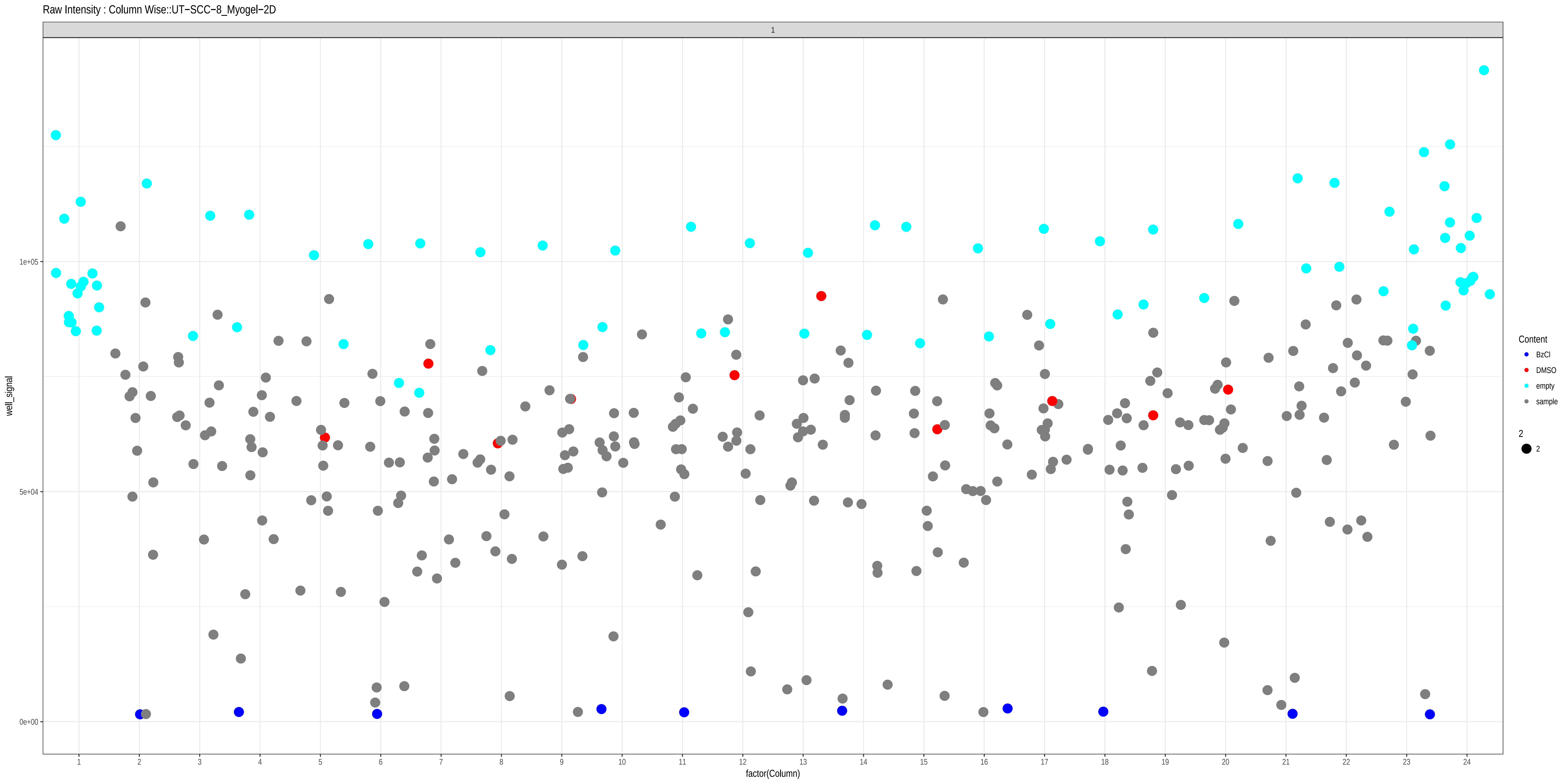
Raw Intensity : Column Wise::UT−SCC−8_Myogel−2D
1
well_signal
1e+05
5e+04
0e+00
factor(Column)
1
2
3
4
5
6
7
8
9
10
11
12
13
14
15
16
17
18
19
20
21
22
23
24
Content
BzCl
DMSO
empty
sample
2
2

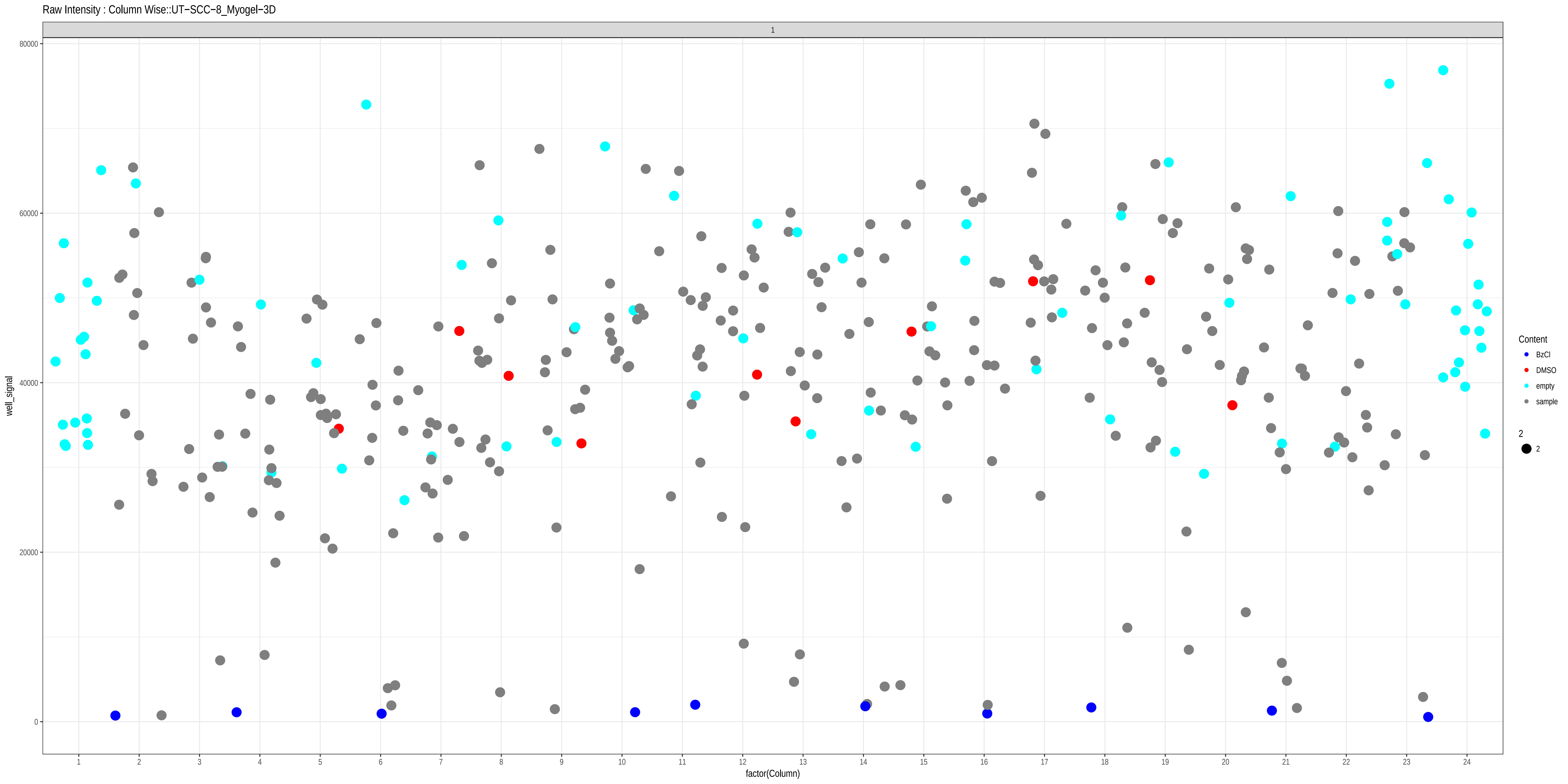

Raw Intensity : Column Wise::UT−SCC−8_Myogel−3D
1
80000
60000
40000
20000
0
well_signal
1
2
3
4
5
6
7
8
9
10
11
12
13
14
15
16
17
18
19
20
21
22
23
24
factor(Column)
Content
BzCl
DMSO
empty
sample
2
2

Raw Intensity : Column Wise::UT−SCC−81_Control

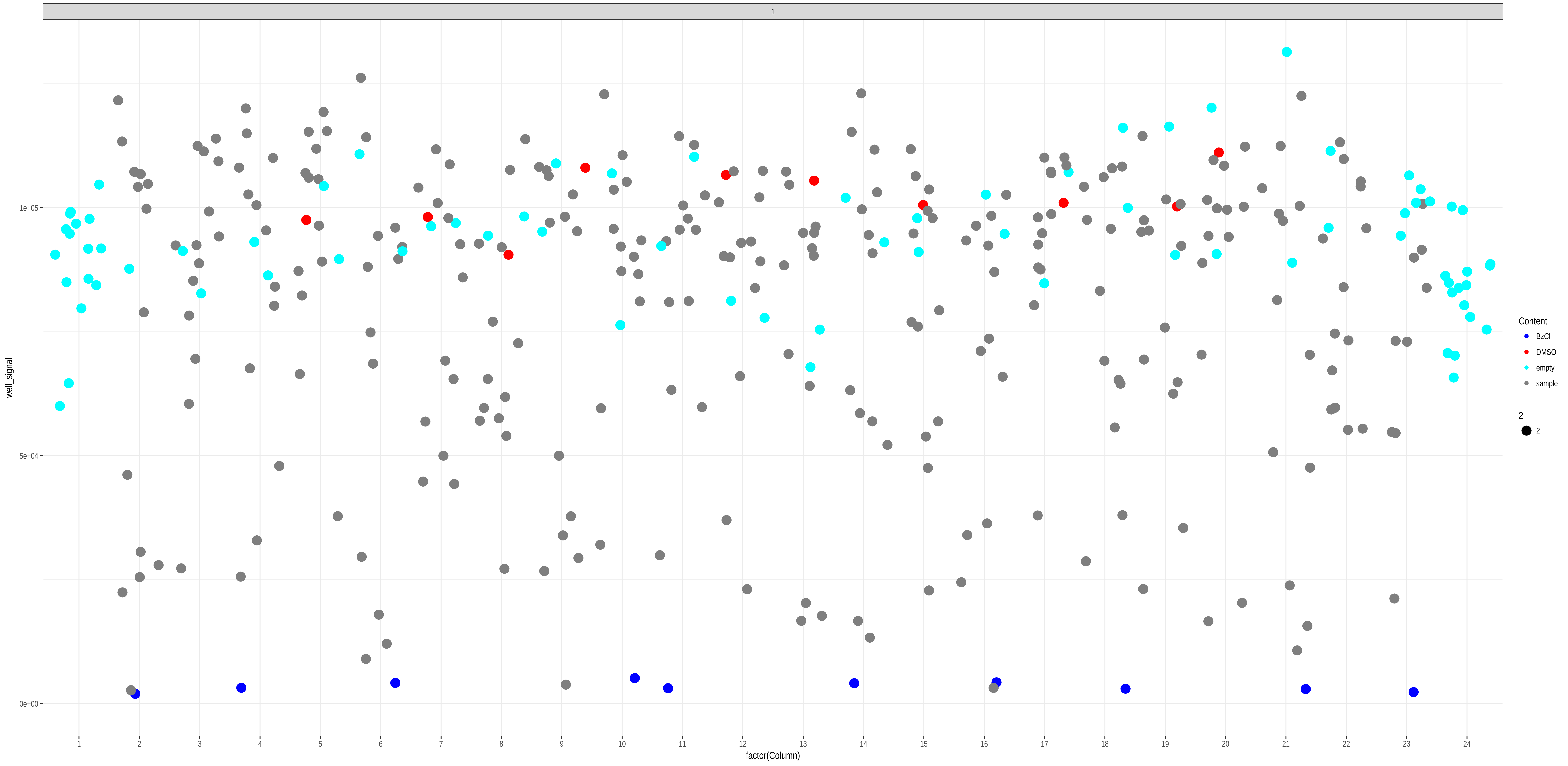

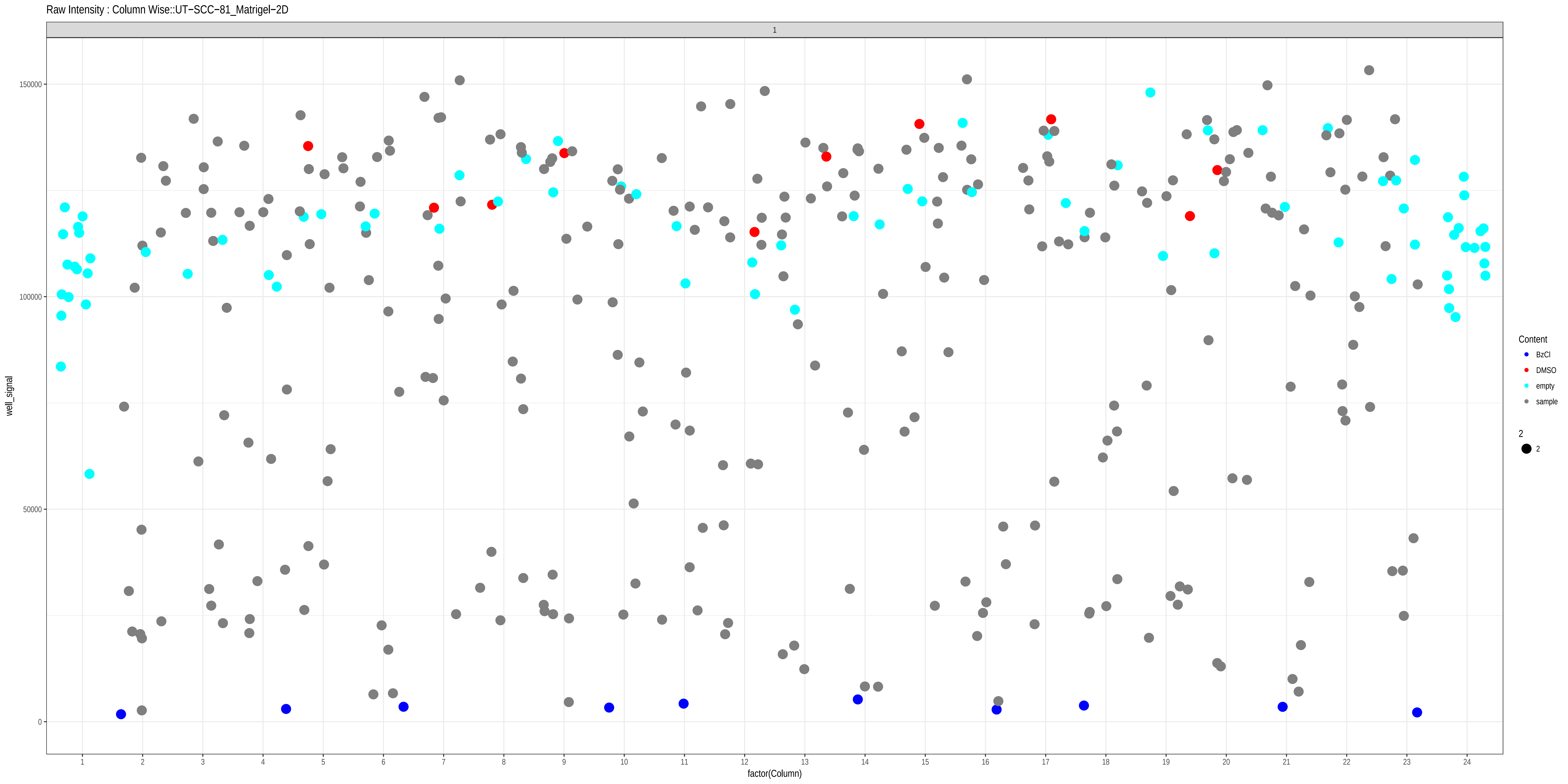

Raw Intensity : Column Wise::UT−SCC−81_Matrigel−2D
1
well_signal
150000
100000
50000
0
factor(Column)
1
2
3
4
5
6
7
8
9
10
11
12
13
14
15
16
17
18
19
20
21
22
23
24
Content
BzCl
DMSO
empty
sample
2
2

Raw Intensity : Column Wise::UT−SCC−81_Matrigel−3D

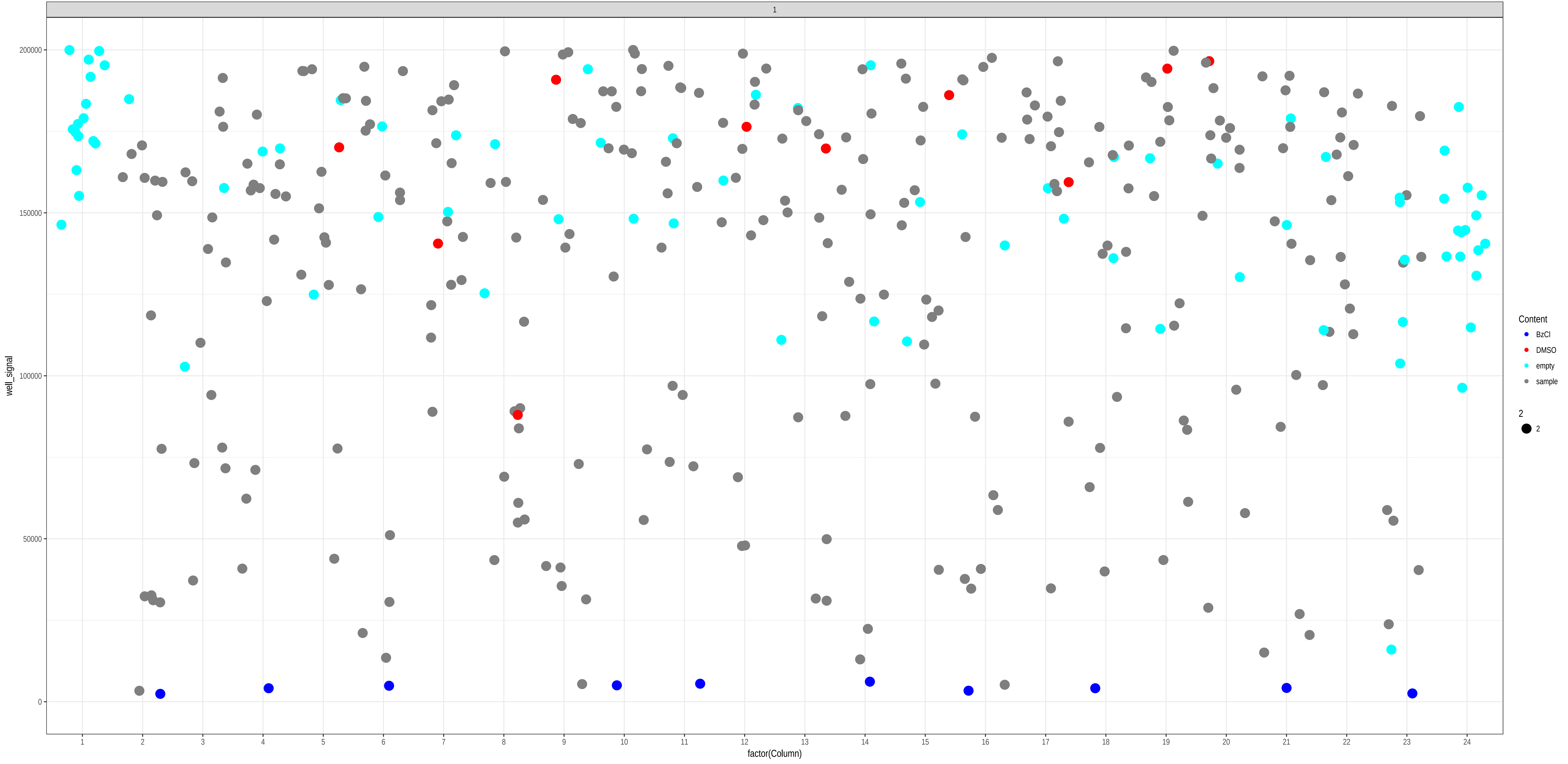

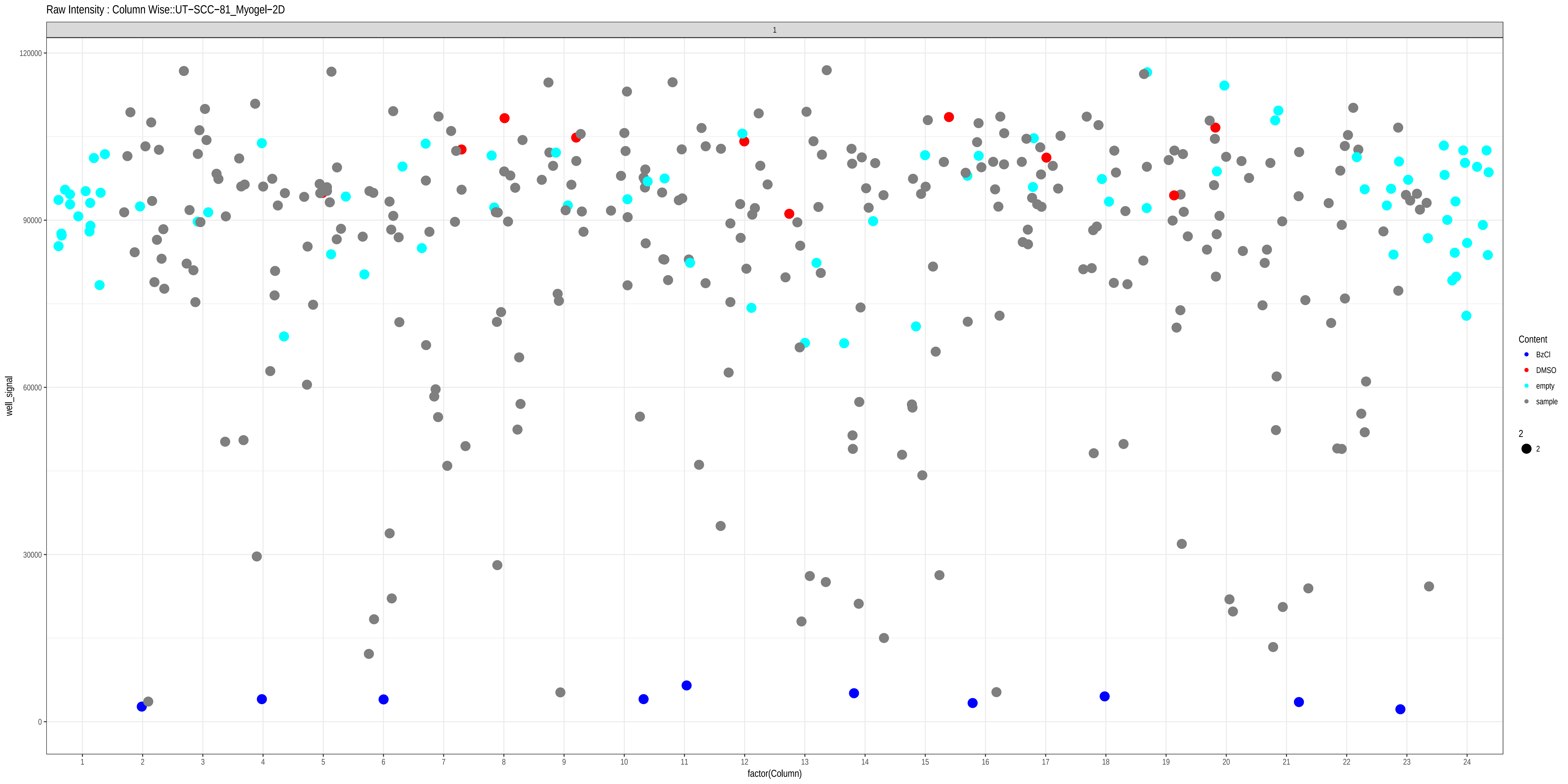

Raw Intensity : Column Wise::UT−SCC−81_Myogel−2D
1
well_signal
120000
90000
60000
30000
0
factor(Column)
1
2
3
4
5
6
7
8
9
10
11
12
13
14
15
16
17
18
19
20
21
22
23
24
Content
BzCl
DMSO
empty
sample
2
2

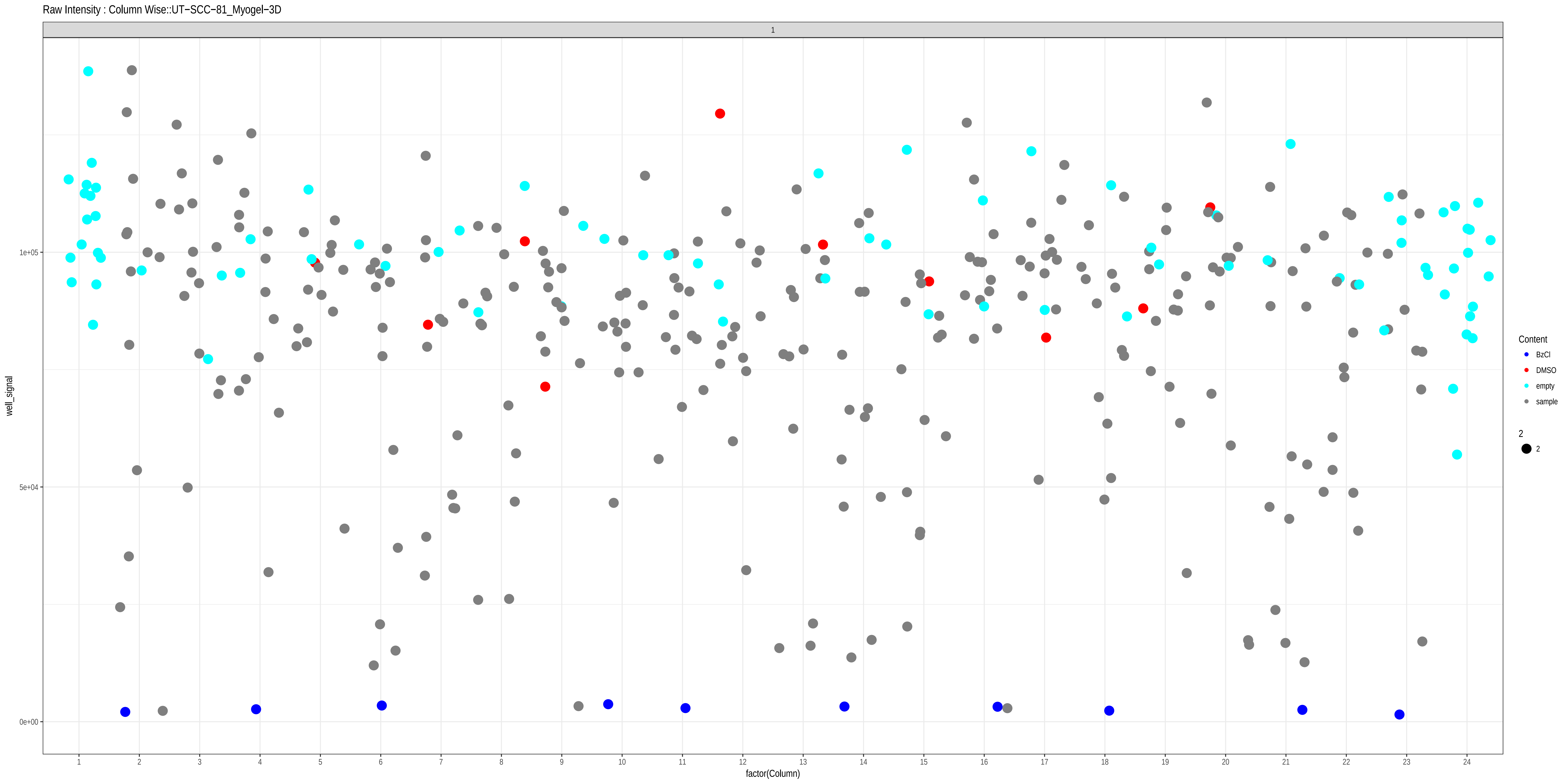
Raw Intensity : Column Wise::UT−SCC−81_Myogel−3D
1
well_signal
1e+05
5e+04
0e+00
factor(Column)
1
2
3
4
5
6
7
8
9
10
11
12
13
14
15
16
17
18
19
20
21
22
23
24
Content
BzCl
DMSO
empty
sample
2
2
