# Supplementary figures and images for: Human Tumor–Derived Matrix Improves the Predictability of Head and Neck Cancer Drug Testing
Source: Cancers (Basel). 2019 Dec 30;12(1):92. doi: 10.3390/cancers12010092 (PMC7017272; doi:10.3390/cancers12010092)

Supplementary Figure 1: Heat maps for drug responses (DSS)

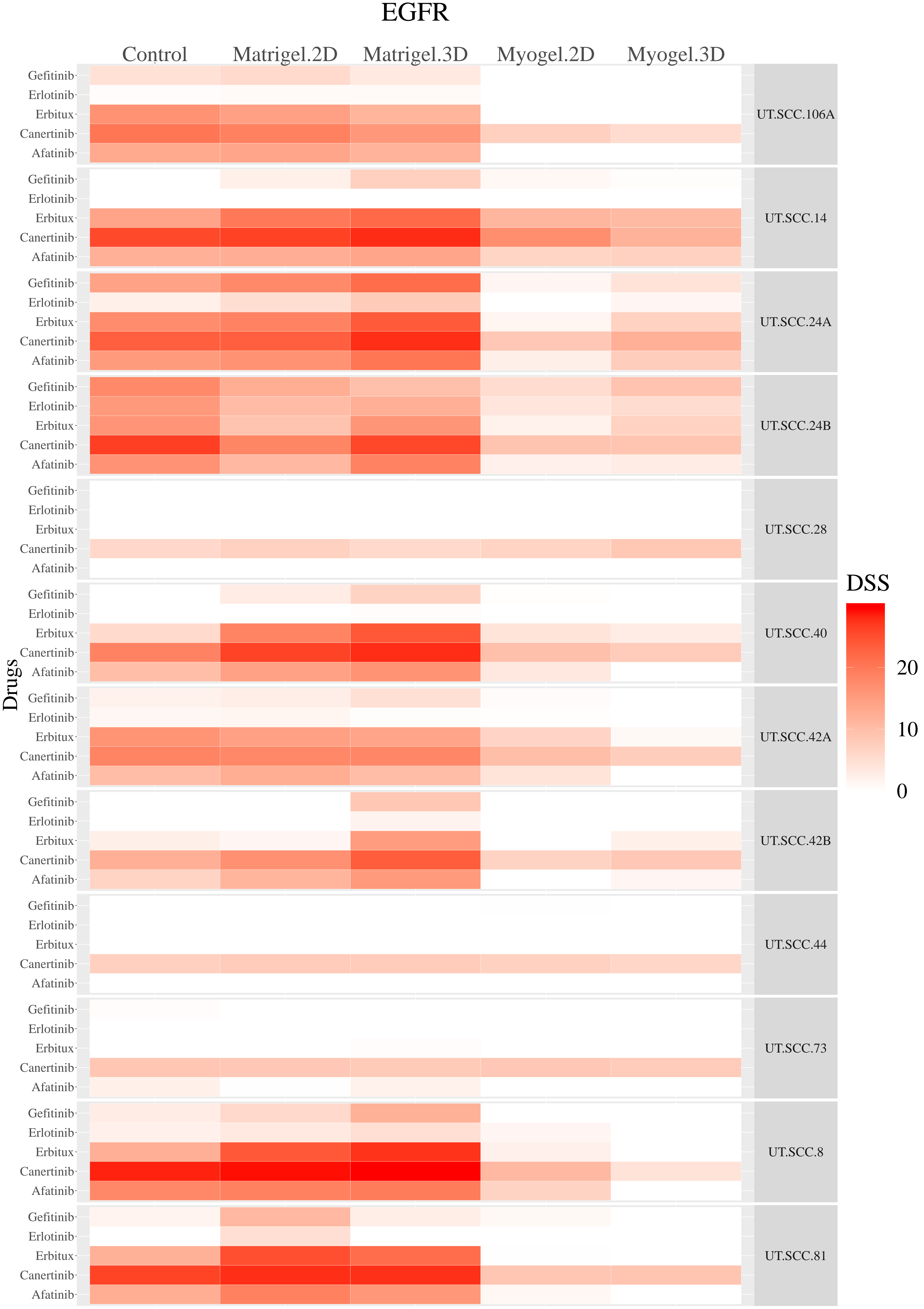

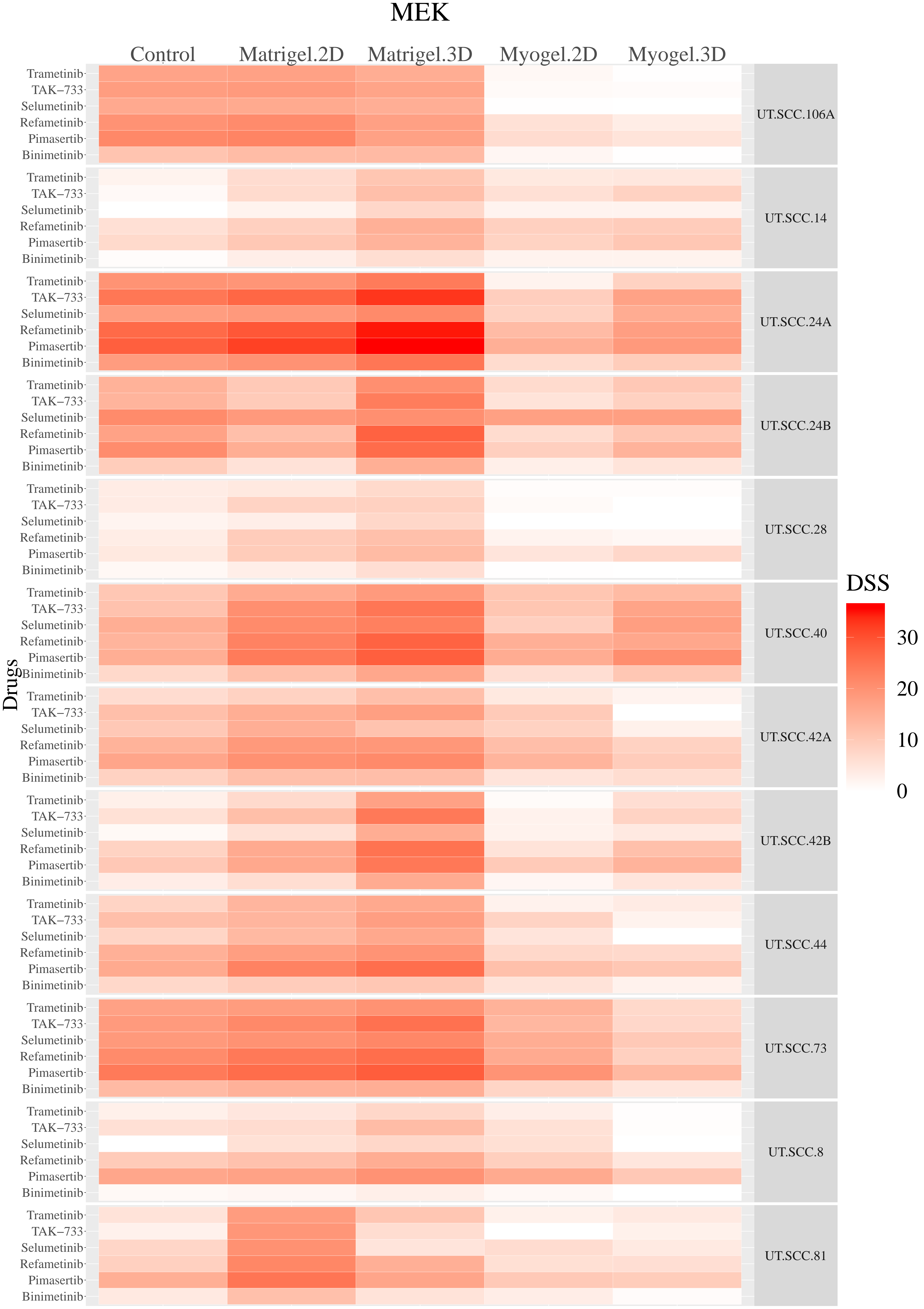

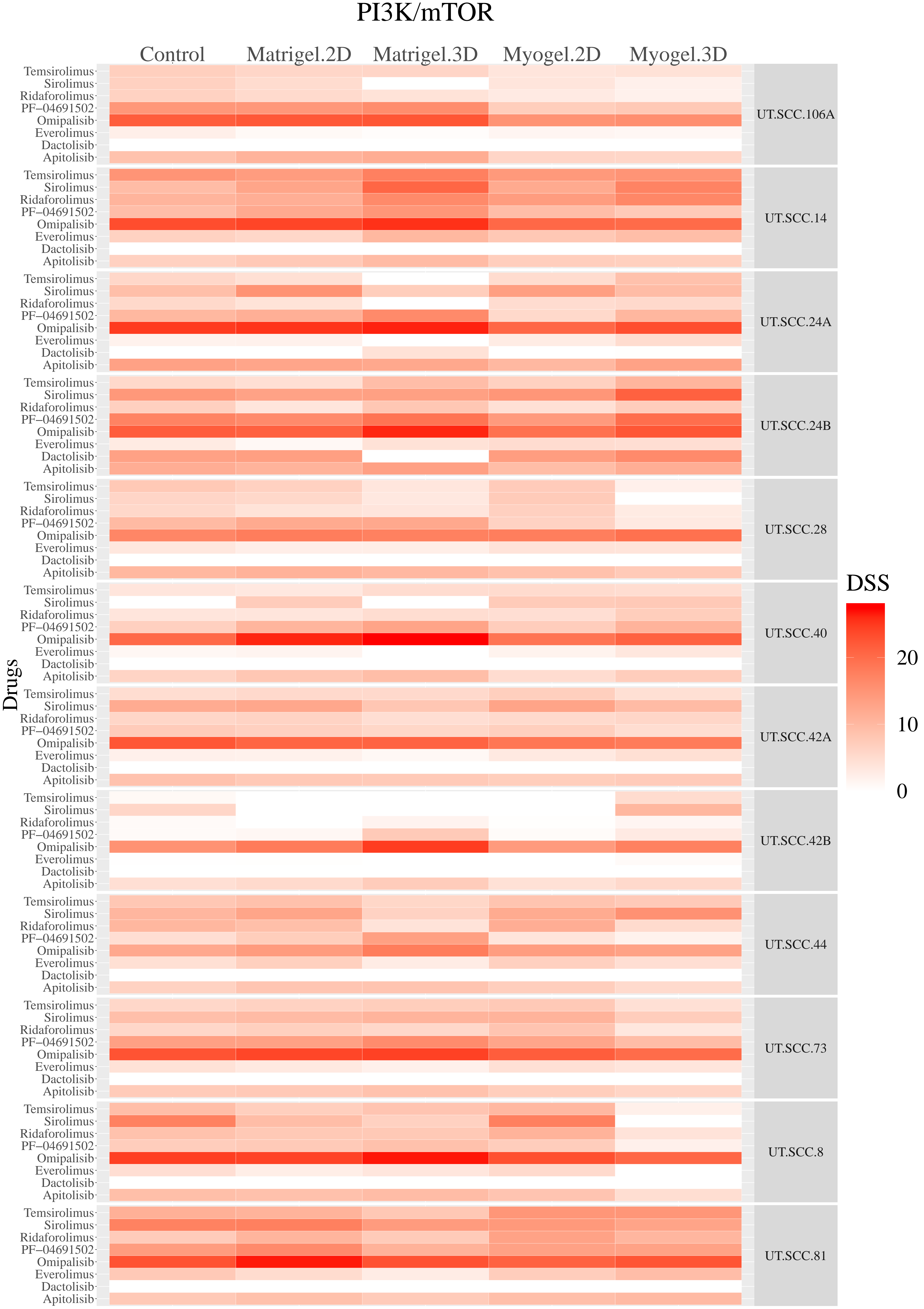

Supplement: Supplementary file 1 [file cancers-12-00092-s001.zip › cancers-664648-supplement-final/Supplementary Figure 1.pdf]
